# Palladium(II) Complexes of Substituted Salicylaldehydes: Synthesis, Characterization and Investigation of Their Biological Profile

**DOI:** 10.3390/ph15070886

**Published:** 2022-07-18

**Authors:** Ariadni Zianna, George Geromichalos, Augusta-Maria Fiotaki, Antonios G. Hatzidimitriou, Stavros Kalogiannis, George Psomas

**Affiliations:** 1 Laboratory of Inorganic Chemistry, Department of Chemistry, Aristotle University of Thessaloniki, GR-54124 Thessaloniki, Greece; hatzidim@chem.auth.gr; 2 Department of Nutritional Sciences and Dietetics, International Hellenic University, Sindos, GR-57400 Thessaloniki, Greece; fiotaki30@gmail.com (A.-M.F.); kalogian@ihu.gr (S.K.)

**Keywords:** palladium(II), substituted salicylaldehydes, antioxidant activity, antimicrobial activity, interaction with DNA, interaction with serum albumins, in silico molecular docking, in silico predictive tools

## Abstract

Five palladium(II) complexes of substituted salicylaldehydes (X-saloH, X = 4-Et_2_N (for **1**), 3,5-diBr (for **2**), 3,5-diCl (for **3**), 5-F (for **4**) or 4-OMe (for **5**)) bearing the general formula [Pd(X-salo)_2_] were synthesized and structurally characterized. The crystal structure of complex [Pd(4-Et_2_N-salo)_2_] was determined by single-crystal X-ray crystallography. The complexes can scavenge 1,1-diphenyl-picrylhydrazyl and 2,2′-azinobis(3-ethylbenzothiazoline-6-sulfonic acid) radicals and reduce H_2_O_2_. They are active against two Gram-positive (*Staphylococcus aureus* and *Bacillus subtilis*) and two Gram-negative (*Escherichia coli* and *Xanthomonas campestris*) bacterial strains. The complexes interact strongly with calf-thymus DNA via intercalation, as deduced by diverse techniques and via the determination of their binding constants. Complexes interact reversibly with bovine and human serum albumin. Complementary insights into their possible mechanisms of bioactivity at the molecular level were provided by molecular docking calculations, exploring in silico their ability to bind to calf-thymus DNA, *Escherichia coli* and *Staphylococcus aureus* DNA-gyrase, 5-lipoxygenase, and membrane transport lipid protein 5-lipoxygenase-activating protein, contributing to the understanding of the role complexes **1**–**5** can play both as antioxidant and antibacterial agents. Furthermore, in silico predictive tools have been employed to study the chemical reactivity, molecular properties and drug-likeness of the complexes, and also the drug-induced changes of gene expression profile (as protein- and mRNA-based prediction results), the sites of metabolism, the substrate/metabolite specificity, the cytotoxicity for cancer and non-cancer cell lines, the acute rat toxicity, the rodent organ-specific carcinogenicity, the anti-target interaction profiles, the environmental ecotoxicity, and finally the activity spectra profile of the compounds.

## 1. Introduction

Palladium(II) complexes are considered important for the synthesis of novel metallodrugs, mainly because of their electronic and structural similarities to platinum(II) complexes [1]. In the literature, a number of reported Pd(II) complexes may serve as anti-viral, anti-fungal, anti-microbial or anti-tumor agents [2], while others have been studied regarding their interaction with DNA and bovine serum albumin (BSA), cytotoxic and antioxidant activity [3,4,5].

Since increased bacterial resistance to current antibiotics brings an urgent need for the discovery of new complexes with antibacterial properties, in recent years, many efforts towards the development of new palladium(II) drugs were shown to direct to a different target: multi-resistant bacterial strains, [6,7]. Recent examples of palladium complexes bearing antimicrobial properties include mixed-ligand Pd(II) complexes of tetracycline and salicylaldehyde [8], Pd(II) complexes bearing azomethine chelates [9], palladium(II) complexes with Schiff bases [10], *N*-heterocyclic carbene-Pd(II)-triphenylophosphine complexes [11], as well as a palladium(II) complex with ibuprofen [6].

A possible drawback of Pd(II) complexes is the ligand exchange at the Pd center (105 times greater than Pt), which causes rapid hydrolytic processes leading to the dissociation of the complex and the formation of very reactive species, which leads to reduced biological activity [3]. The above-mentioned drawback could be overcome by using ligands with strong chelating ability, such as substituted salicylaldehydes (X-saloH). Substituted salicylaldehydes are able to coordinate with metal ions, mainly in a chelating bidentate (O,O′-) manner, through their carbonyl and phenolato oxygen atoms, while there have been very few examples of different coordination modes [12,13,14,15,16].

A number of metal complexes of X-saloH with interesting biological features can be found in the literature. Mixed-ligand Cu(II) complexes of X-saloH and 2,2′-bipyridine or 1,10-phenanthroline as co-ligands presented antiproliferative properties [17], while other Cu(II) complexes may act as artificial nucleases [18]. Furthermore, Co(II) complexes of salicylaldehydes exhibited antimicrobial [19] or anticancer properties [20]. In addition, previous studies conducted by our laboratory showed that metal complexes of substituted salicylaldehydes showed interesting results regarding their affinity to calf-thymus (CT) DNA and their binding to serum albumins (SAs) [21,22,23] as well as their antimicrobial and antioxidant properties [13,14,23].

**Figure 1:**
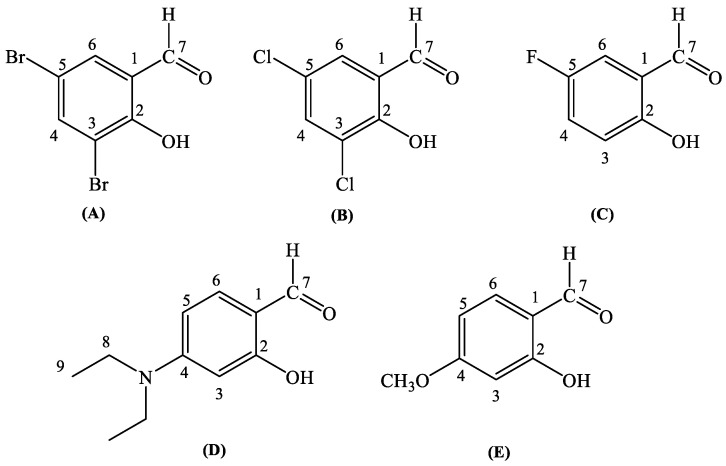
Syntax formula and H-atom numbering for (**A**) 3,5-diBr-saloH, (**B**) 3,5-diCl-saloH, (**C**) 5-F-saloH, (**D**) 4-Et_2_N-saloH, and (**E**) 4-OMe-saloH.

In order to gain more insight into the structure–activity relationship, we have synthesized and characterized five novel palladium(II) complexes with X-saloH as ligands, namely [Pd(4-Et_2_N-salo)_2_] (**1**), [Pd(3,5-diBr-salo)_2_] (**2**), [Pd(3,5-diCl-salo)_2_] (**3**), [Pd(5-F-salo)_2_] (**4**) and [Pd(4-OMe-salo)_2_] (**5**), where 4-Et_2_N-saloH = 4-diethylamino-salicylaldehyde, 3,5-diBr-saloH = 3,5-dibromo-salicylaldehyde, 3,5-diCl-saloH = 3,5-dichloro-salicylaldehyde, 5-F-saloH = 5-fluoro-salicylaldehyde and 4-OMe-saloH = 4-methoxy-salicylaldehyde (Figure 1). All complexes were characterized by physicochemical and spectroscopic techniques (IR, UV-vis and ^1^H NMR). The crystal structure of complex **1** was determined by single-crystal X-ray crystallography.

The study of the potential antioxidant activity of X-saloH and their Pd(II) complexes was focused on their ability to scavenge free radicals 1,1-diphenyl-picrylhydrazyl (DPPH), 2,2′-azinobis(3-ethylbenzothiazoline-6-sulfonic acid) (ABTS) free radicals and to reduce H_2_O_2_. The antimicrobial activity of X-saloH and complexes **1**–**5** was studied in vitro against two Gram-positive (*Staphylococcus aureus* ATCC 6538 (*S. aureus*) and *Bacillus subtilis* ATCC 6633 (*B. subtilis*)) and two Gram-negative microorganisms (*Escherichia coli* NCTC 29,212 (*E. coli*)) and *Xanthomonas campestris* ATCC 1395 (*X. campestris*)) by determining the minimum inhibitory concentration (MIC).

The interaction of the complexes with CT DNA was investigated in vitro regarding the binding affinity for CT DNA by UV-vis spectroscopy, by viscosity measurements, via their ability to displace ethidium bromide (EB) from the DNA-EB conjugate. Moreover, the in vitro affinity of the complexes to bind to human serum albumin (HSA) and BSA was evaluated by fluorescence emission spectroscopy, the SA-binding constants were determined and the SA-binding site was investigated.

In order to explore the capacity of the studied compounds to act as antibacterial and antioxidant agents, thus suggesting a mechanistic mode of action, as well as to elucidate their binding mode on CT DNA, molecular docking simulations on the crystal structure of *E. coli* and *S. aureus* DNA-gyrases, CT DNA, 5-lipoxygenase (5-LOX) and membrane transport lipid protein 5-lipoxygenase-activating protein (FLAP) proteins were employed.

In order to decipher the discrepancy between in vitro and in silico results, a computational calculation approach to the electronic structure of complexes **1**–**5** was adopted. Additionally, a variety of computational tools were employed to predict the complete biological activity profile of complexes **1**–**5**. Predictive tools include general pharmacological potential (prediction of activity spectra), prediction of target proteins affected by the compound, prediction of drug-induced changes of gene expression profile, protein and mRNA based prediction results, prediction substrate/metabolite specificity and sites of metabolism, prediction of cytotoxicity for tumor and non-tumor cell lines, rodent organ-specific carcinogenicity prediction and calculation of ADMET (absorption, distribution, metabolism, excretion, and toxicity) parameters, pharmacokinetic properties, drug-like nature and medicinal chemistry friendliness of the compound.

## 2. Results and Discussion

### 2.1. Synthesis and Characterization

The novel neutral palladium complexes were synthesized according to our published procedure [22] from the reaction of Pd(CH_3_COO)_2_ with the corresponding deprotonated X-salo^−^ ligand, and possess a 1:2 Pd^2+^-to-(X-salo^−^) composition. In all complexes, the X-salo^−^ ligands are coordinated with Pd(II) in a bidentate chelating mode through their carbonyl and deprotonated phenolato oxygen atoms. Evidence of the coordination mode of the ligands in the complexes has also arisen from the interpretation of the IR, ^1^H NMR and UV-vis spectra. The crystal structure of complex **1** was further verified by single-crystal X-ray diffraction analysis.

All complexes are soluble in DMF and DMSO, but insoluble in H_2_O, Et_2_O and most organic solvents. Conductivity measurements have shown that complexes **1**–**5** are non-electrolytes in DMSO solution, since the values of the Λ_M_ of the complexes in a 1 mM DMSO solution were found to be <15 mho∙cm^2^∙mol^−1^ [24].

In the IR spectra, free X-saloH present two peaks attributed to the stretching (~3200 cm^−1^) and bending (1400 cm^−1^) vibrations of its phenolic OH. In the IR spectrum of complexes **1**–**5**, these two peaks vanish because of the deprotonation and the coordination of the phenolato group of the X-salo^−^ to the Pd ion. The peak at ~1679 cm^−1^, which is attributed to aldehyde bond *v*(HC=O) of the uncoordinated X-saloH, is shifted in the IR spectra of complexes **1**–**5** towards 1620 cm^−1^, indicating coordination with the carbonyl oxygen. Finally, the bands due to the C-O stretching vibrations at 1258–1285 cm^−1^ of free X-saloH exhibit positive shifts towards 1315–1324 cm^−1^ in the IR spectra of complexes **1**–**5** because of the coordination of the phenolato oxygen of the ligand [25]. The findings of the IR spectroscopy are in agreement with the determined X-ray crystal structure.

^1^H NMR spectroscopy has also been used in order to confirm the deprotonation of the salicylaldehyde as well as the stability of the complexes in the solution. The ^1^H NMR spectra are consistent with the obtained structures of **1**–**5**. In the ^1^H NMR spectra of the free X-saloH, the -OH signal appears as a broad peak at δ = 12 ppm. The absence of this signal in the ^1^H NMR spectra of the complexes proves the deprotonation. The ^1^H NMR spectra of the title complexes and the protons attributable to the aldehydo group are found at δ~9.90 ppm. All sets of signals related to the presence of the ligands in the corresponding compounds are present and are slightly shifted as expected upon binding to palladium ion. The absence of an additional set of signals related to dissociated ligands suggests that all complexes remain intact in solution. The ^1^H NMR spectra were also recorded for different time intervals (up to 72 h) and remained unchanged.

The UV-visible (UV-vis) spectra of the complexes were recorded as a nujol mull corresponding to the solid state, and in DMSO or buffer solutions used in biological experiments (150 mM NaCl and 15 mM trisodium citrate at pH values regulated in the range 6–8 by HCl solution). The spectra in solid and solution states were found to be similar, suggesting that the complexes do not dissociate in the pH range 6–8 [22]. In particular, in square-planar palladium(II) complexes, three d-d type bands are expected due to transitions from the ground state to the excited ^1^A_2g_, ^1^B_1g_ and ^1^E_1g_, at 460–520, 405–420 and 320–380 nm, respectively. In all complexes **1**–**5**, the first two transition bands seem to overlap, and as a result, two main bands are observed.

### 2.2. Structure of the Complexes

#### 2.2.1. Description of the Crystal Structure of [Pd(4-Et_2_N-salo)_2_]·CH_3_CN, (**1**)·CH_3_CN

**Figure 2:**
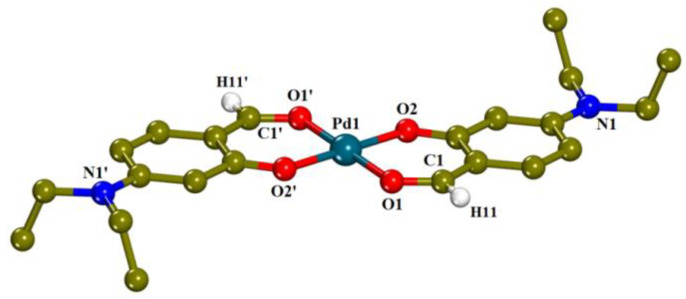
Crystal structure of complex **1**. Aromatic and ethyl hydrogen atoms and solvate molecules are omitted for clarity. Selected bond distances and angles: Pd1—O1 = 1.987(2) Å, Pd—O2= 1.985(2) Å; O1—Pd1—O2 = 94.83(9)°, O1′—Pd1—O2 = 85.17(9)°. (Symmetry code: (′) −*x* + 1, −*y* + 1, −*z* + 1).

The molecular structure of complex **1** is presented in Figure 2. The unit cell consists of two neutral complex moieties and two totally acetonitrile solvate molecules disordered over four positions with occupation factors of 0.5. Crystallographic data for complex **1** are presented in Table S1. The coordination sphere around the palladium ion consists of two deprotonated 4-Et_2_N-salo^−^ ligands, coordinated with the metal ion in a bidentate chelating manner via the carbonyl oxygen (O1) and the phenolic oxygen (O2), leading to a square planar geometry around Pd1. The Pd-O distances (Pd1—O1 = 1.987(2) Å and Pd—O2 = 1.985(2) Å) are almost equal. To the best of our knowledge, the crystal structures of only two palladium complexes with (substituted) salicylaldehydes have been reported up to date, i.e., [Pd(3-OMe-salo)_2_] [22] and [Pd(salo)_2_] [26], which present similar arrangement of the ligands around Pd(II) ion.

#### 2.2.2. Proposed Structures for Complexes **2**–**5**

Based on characterization studies conducted by IR and UV-vis spectroscopy, elemental analysis and molar conductivity, as well as the comparison with the crystal structures of complex **1** and reported similar palladium complexes [22,26], we may suggest that complexes **2**–**5** also present square planar geometry with the two deprotonated X-salo^−^ ligands bidentately bound to Pd, most likely in *trans* positions.

### 2.3. Antioxidant Activity

Generally, antioxidants found in food are rich in organic compounds (phenolic and cinnamic acids, flavones and flavonoids, etc.). The carboxylic group in these acids or a near hydroxyl group and an oxo group for flavonoids and flavones enables them to coordinate with metal ions through their oxygen atoms forming stable complexes. The combination of the redox properties of metal ions and various ligands is an interesting method to develop antioxidant compounds [27].

For the above reasons, the scavenging activity of X-saloH and palladium(II) complexes **1**–**5** has been evaluated towards DPPH and ABTS, as well as their ability to reduce H_2_O_2_ and has been also compared with that of the well-known antioxidant agents nordihydroguaiaretic acid (NDGA), butylated hydroxytoluene (ΒHΤ), 6-hydroxy-2,5,7,8-tetramethylchromane-2-carboxylic acid (Trolox) and L-ascorbic acid, which are the most commonly used standard reference antioxidant agents [28,29]. The Pd(II) starting material did not show any noteworthy activity. The results are summarized in Table 1.

The DPPH radical assay was developed in the 1950s [30] and this method has often been used to assess the antioxidant capacity of several metal complexes [27]. The DPPH-scavenging ability of compounds has often been related to their ability to prevent aging, cancer and inflammation [31]. Complexes **1**–**5** presented a low ability to scavenge DPPH and are in general as active as the corresponding X-saloH and less active than the reference compounds BHT and NDGA. Time did not seem to influence the DPPH-scavenging ability of X-saloH and complexes **1**–**5**.

The ability of a compound to scavenge the cationic ABTS radicals (ABTS^+●^) has often been used to evaluate its total antioxidant activity [31]. Almost all complexes **1**–**5** are more active ABTS scavengers than the corresponding X-saloH ligands. Complexes **1** and **5** presented the highest ABTS-scavenging activity among the compounds which was close to that of the reference compound Trolox.

H_2_O_2_ has the ability to penetrate biological membranes. It is not very reactive itself, but it can sometimes be toxic because it may give rise to hydroxyl radical in the cells. For this reason, removing H_2_O_2_ is very important for the protection of living systems [32]. When a scavenger is incubated with H_2_O_2_ using a peroxidase assay system, the loss of H_2_O_2_ can be measured [33]. Complexes **1**–**5** presented a moderate ability to reduce H_2_O_2_. In addition, it may be noted that the free X-saloH compounds show significantly high potency towards the reduction of H_2_O_2_ which is higher than that of their corresponding complexes and even the reference compound L-ascorbic acid.

On average, complexes 1–5 presented similar or lower antioxidant activity when compared to their Zn(II) [13,14] or Mn(II) [23] analogs.

### 2.4. Antimicrobial Activity

According to the literature, substituted salicylaldehydes may present significant antimicrobial properties, but the mechanism of action of these agents remains a mystery [34]. An explanation for this may be the formation of Schiff bases with amino groups of microbial cells. Benzaldehyde and salicylaldehyde have been found to present zero-to-low activity, but substitutions such as halogenation, hydroxylation and nitro-substitution may often result in highly active compounds. However, the activity cannot be foreseen and may vary from microbe to microbe [34,35].

Pelttari et al. tested among other substituted salicylaldehydes the antimicrobial activity of 5-Cl-saloH and 5-Br-saloH against a number of microbes [35]. They found that in most cases, 5-Br-saloH was highly active, presenting significantly higher activity than 5-Cl-saloH. Both of the 5-halogenated salicylaldehydes had relatively equally low activity against *P. aeruginosa* and *A. niger* [35]. Ntanatsidis et al. found that 5-Br-saloH had better antimicrobial potency than 5-Cl-saloH against *X. campestris*, while 5-Cl-saloH and 5-Br-saloH had equal activity against *S. aureus* [23]. Regarding the case of *E. coli*, the doubly halogenated 3,5-diCl-saloH was much more active than its singly halogenated analogous compound 5-Cl-saloH, while 3,5-diBr-saloH was less active than the highly effective 5-Br-saloH. In the case of 3,5-dihalogenated salicylaldehydes, the highest activity was usually displayed by 3,5-diCl-saloH. It can be noted that the increase of activity from the difluoro-saloH to the dichloro-saloH one may be attributed to a change in the inductive effect of the substituents [35]. 3,5-diBr-saloH presented the best antimicrobial potency, followed by 3,5-diCl-saloH and 5-F-saloH, while 4-Et_2_N-saloH and 4-OMe-saloH presented the lowest antimicrobial potency against all microbes (>200 μg/mL). These findings suggest that substitution in position 4 does not seem to favor antimicrobial potency. Peltarri et al. also found that 4-Et_2_N-saloH presented minimum activity [35].

The antimicrobial activities of X-saloH and complexes **1**–**5** in the present study were evaluated against two Gram-negative (*X. campestris* and *E. coli*) and two Gram-positive (*B. subtilis* and *S. aureus*) bacterial strains and the results are presented in Table 2. The MIC values of all ligands are within the range 25–200 μg/mL (89–1427 μΜ), the MIC value of the starting Pd(II) salt (Pd(CH_3_COO)_2_) was >200 μg/mL, and the MIC values of the complexes are found in the range of 25–100 μg/mL (51–407 μM). As can be seen in most cases, complexes **1**–**5** presented better antimicrobial activity than the corresponding X-saloH, especially when the MIC values are considered in the molar scale, suggesting that the antimicrobial potency of the X-saloH is improved upon coordination with Pd(II). Coordination to metal ions seems in most cases to improve the antimicrobial activity of the salicylaldehydes [13,14,23]. Considering the potential selectivity of the compounds, they do not show significant differentiation among the Gram-(+) or the Gram-(−) bacterial species. In addition, complexes **2** and **3** bearing the dihalogeno-substituted salo ligands were more active than the other complexes. The highest antimicrobial activity is provided by complex **2** against all tested microbes (MIC = 25 μg/mL, 38 μM).

### 2.5. Interaction with CT DNA

The importance to explore the binding affinity of the studied complexes to CT DNA is obvious in order to analyze the mechanism implicated in their pharmacological effects. DNA is among the common biological targets of anticancer drugs since one of the main mechanisms of action of these drugs is the damage of DNA [36]. So, an attempt to reveal possible interaction with double-stranded DNA is of valuable importance. Metal complexes may interact in various modes with DNA. Covalent interactions take place when one at least labile ligand of the complex is replaced by DNA-base nitrogen, while when non-covalent binding occurs, the metal complexes may interact with DNA via three main types of weak interactions: (i) π–π stacking interactions of the complexes between DNA-base pairs which leads to intercalation; (ii) electrostatic interaction outside of the helix (Coulomb forces); and (iii) van der Waals forces, hydrogen bonding, and hydrophobic interactions with a major or minor DNA-groove [37].

**Figure 3:**
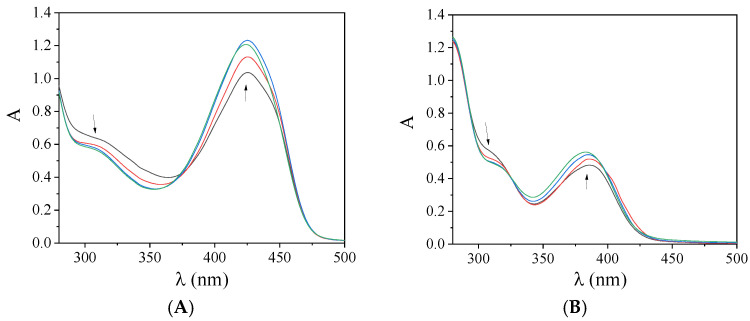
UV-vis spectra of DMSO solution of (**A**) **2** (10^−4^ M), (**B**) **5** (10^−4^ M) in the presence of increasing amounts of CT DNA. The arrows show the changes upon increasing amounts of CT DNA.

The preliminary study of the interaction between a complex and CT DNA was performed by monitoring the changes of the λ_max_ of the complexes in the presence of increasing amounts of CT DNA [38]. Within this context, the UV-vis spectra of X-saloH and complexes **1**–**5** (10^−5^–10^−4^ M) were recorded in the presence of CT DNA (Figure 3 and Figure S1). In the UV-vis spectra of the compounds, two main bands are observed. Upon addition of CT DNA, in most cases, hypochromism is observed for band I with λ_max_ in the range 314–339 nm, while hyperchromism accompanied by a slight blue shift is mostly observed for band II in the region 380–428 nm (Table 3). The obtained data may reveal the interaction of the complexes with CT DNA [39], but may not clarify the interaction mode further leading to the necessity for more experiments, such as DNA-viscosity measurements and competitive studies with ethidium bromide.

The DNA-binding constants (K_b_) of the compounds, as determined by the Wolfe–Shimer equation (Equation (S1)) [40] and the plots [DNA]/(ε_A_–ε_f_) *versus* [DNA] (Figure S2), may show the magnitude of their interaction with CT DNA. The K_b_ constants of complexes **1**–**5** (Table 3) are relatively high (of the order 10^5^–10^6^ M^−1^) with complex **3** showing the highest K_b_ constant (=1.90 (±0.12) × 10^6^ M^−1^) among them. The values of K_b_ are in the range found for analogous Pd(II) complexes of X-saloH [22] and, in most cases, higher than that of the classical intercalator EB (=1.23 (±0.07) × 10^5^ M^−1^) [41].

**Figure 4:**
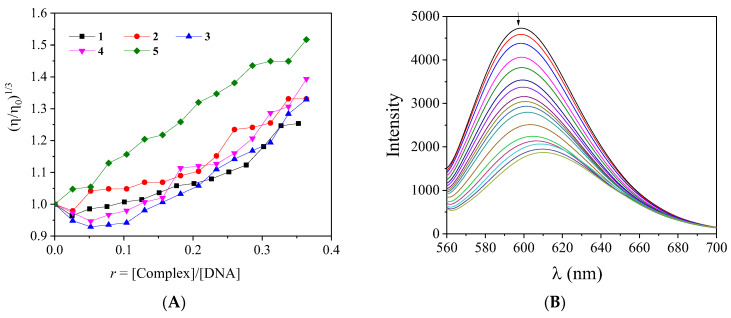
(**A**) Relative viscosity (η/η_0_)^1/3^ of CT DNA (0.1 mM) in buffer solution (150 mM NaCl and 15 mM trisodium citrate at pH 7.0) in the presence of complexes **1**–**5**, at increasing amounts (*r* = [compound]/[DNA] = 0–0.36). (**B**) Fluorescence emission spectra (λ_excitation_ = 540 nm) for EB-DNA adduct ([EB] = 20 μM, [DNA] = 26 μM) in buffer solution (150 mM NaCl and 15 mM trisodium citrate at pH = 7.0) in the absence and in the presence of increasing amounts of complex **2** (up to *r* = 0.35). The arrow shows the changes of intensity upon increasing amounts of **2**.

The viscosity of DNA is usually related to its length changes induced by the interaction with a compound [42]. The viscosity of a CT DNA solution (0.1 mM) was monitored in the presence of increasing amounts (up to *r* = [compound]/[DNA] = 0.36) of the compounds at RT. For all complexes **1**–**5**, the relative DNA-viscosity exhibited an increase which was more pronounced for complex **5** (Figure 4A). These experimental findings may be considered evidence of the existence of an intercalative binding mode to DNA; in the case of intercalation, the DNA viscosity increases as a result of the increase of the separation distance of DNA bases in order to make the necessary room for the accommodation of the intercalating compound [42].

EB is categorized as a typical DNA-intercalation indicator since it can intercalate in-between adjacent DNA-base pairs. A solution containing the EB-DNA adduct, when excited with λ_ex_ = 540 nm, presents an intense fluorescence emission band at 592 nm [43]. When a compound with the ability to intercalate to DNA equally or stronger than EB is added to this solution, changes to the emission band may occur and can be monitored in order to investigate the competition of the compound with EB for the DNA-intercalation site. X-saloH and complexes **1**–**5** do not show any fluorescence emission bands at room temperature in solution or in the presence of CT DNA or EB under the same experimental conditions. So, any changes observed in the fluorescence emission spectra of the EB-DNA solution, when the compounds are added, are useful to examine the EB-displacing ability of the complexes as possible indirect evidence of their intercalating ability [43,44].

The fluorescence emission spectra of pre-treated EB-DNA ([EB] = 20 µM, [DNA] = 26 µM) were recorded in the presence of increasing amounts of the compounds (representatively shown for complex **2** in Figure 4B) and showed a significant decrease of the fluorescence emission band of the DNA-EB compound at 592 nm. The most pronounced decrease was up to 64.8% for complex **2** (Figure S3, Table 4). The observed quenching may reveal the significant ability of the complexes to displace EB for the EB-DNA and, thus, an intercalative mode of interaction of the complexes with CT DNA is indirectly proposed [44].

The Stern–Volmer (K_SV_) constants (Table 4) of the complexes were calculated from the Stern–Volmer equation and the Stern–Volmer plots (Figure S4) and have shown relatively high values, indicating a tight binding to DNA. Complexes **1** and **2** exhibit the highest K_SV_ constants (=1.00 × 10^5^–1.03 × 10^5^ M^−1^) among the complexes. The EB-DNA quenching constants (k_q_) of the compounds (Table 4) were calculated with Equation (S3) (considering τ_o_ = 23 ns as the fluorescence lifetime [45]) and are higher than 10^10^ M^−1^s^−1^ [44], so a static quenching mechanism is proposed for the quenching of the fluorescence induced by the compounds [22], suggesting their interaction with the fluorophore.

### 2.6. Interaction with Serum Albumins

#### 2.6.1. Affinity of the Compounds for BSA and HSA

Serum albumins are among the important proteins of the circulatory system. Their main role is to carry drugs and other bioactive small molecules through the bloodstream [46,47]. BSA is the most widely studied albumin and is structurally homologous to HSA having two and one tryptophan residues, respectively [48]. The tryptophan residues of both albumins are responsible for the intense fluorescence emission band with λ_em,max_= 342 nm for BSA and 351 nm for HSA, respectively, when their solutions are excited at 295 nm [43]. The solutions of some of complexes **1**–**5** exhibited a maximum emission in the region 395–415 nm under the same experimental conditions and in such cases, the SA-fluorescence emission spectra were corrected before the calculations processing. The inner-filter effect was also taken into consideration and was calculated with Equation (S5) [49]; it was found to be rather low, with a minimal effect on the measurements.

**Figure 5:**
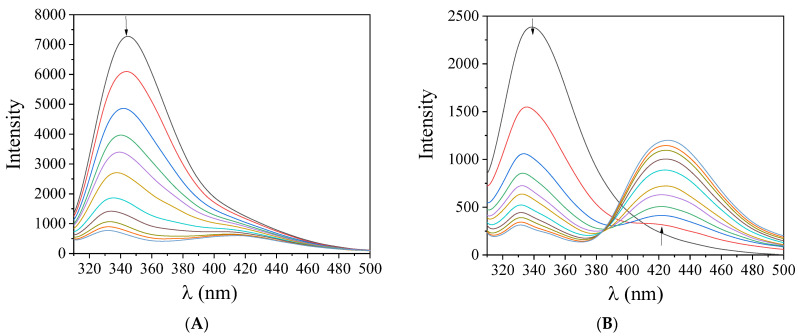
Fluorescence emission spectra (λ_excitation_ = 295 nm) of a buffer solution (150 mM NaCl and 15 mM trisodium citrate at pH 7.0) containing (**A**) BSA (3 μM) upon addition of increasing amounts of complex **2**, and (**B**) HSA (3 μM) upon addition of increasing amounts of complex **3**. The arrows show the changes in intensity upon increasing amounts of the complex.

When the compounds were added to an SA solution (3 μM), a significant quenching of the fluorescence emission bands (at λ_em_ = 342 nm for BSA and at λ_em_ = 351 nm for HSA) of the albumins was observed (Figure 5). The quenching induced by the compounds was more pronounced in the case of BSA (Table 5 and Figure S5). The observed quenching may be ascribed to changes in the tryptophan environment of SA due to the possible denaturation of their secondary structure, resulting from the binding of the complexes to SA [43].

The SA-quenching constants (k_q_) for complexes **1**–**5** (Table 5) (calculated from the corresponding Stern–Volmer plots (Figures S6 and S7) with the Stern–Volmer quenching equation (Equations (S2) and (S3))) are much higher than 10^10^ M^−1^s^−1^, which is an indication of a static quenching mechanism [44], verifying the interaction of the compounds with the albumins. The k_q_ constants of complexes **1**–**5** are similar to those reported for similar Pd(II) and other metal complexes with substituted salicylaldehydes such as ligands [13,14,21,22,23].

The SA-binding constants (K) of the complexes (calculated from the corresponding Scatchard plots (Figures S8 and S9) with the Scatchard equation (Equation (S5))) (Table 5) are relatively high, suggesting a tight interaction of the compounds with the albumins. Therefore, the compounds may get transported by the albumins toward their potential biological targets. On the other hand, the K values are significantly lower than the constant of avidin, K_avidin_~10^15^ M^−1^ (it is considered as the limit between reversible and irreversible interactions), suggesting the interaction is reversible so that the complexes may get released when they approach their targets [50].

#### 2.6.2. Location of the Albumin-Binding Site

According to crystallography, albumins consist of three domains (I, II and III) which are subdivided into two subdomains (A and B) [51]. There are at least four sites in the albumin where drugs and metal ions can be bound, with the most important sites being Sudlow’s site 1 (or drug site I) in subdomain IIA and Sudlow’s site 2 (or drug site II) in subdomain IIIA [51]. Warfarin and ibuprofen are the most prevalent markers of the albumin-binding site since they show a selective binding affinity for drug sites I and II, respectively [52].

**Figure 6:**
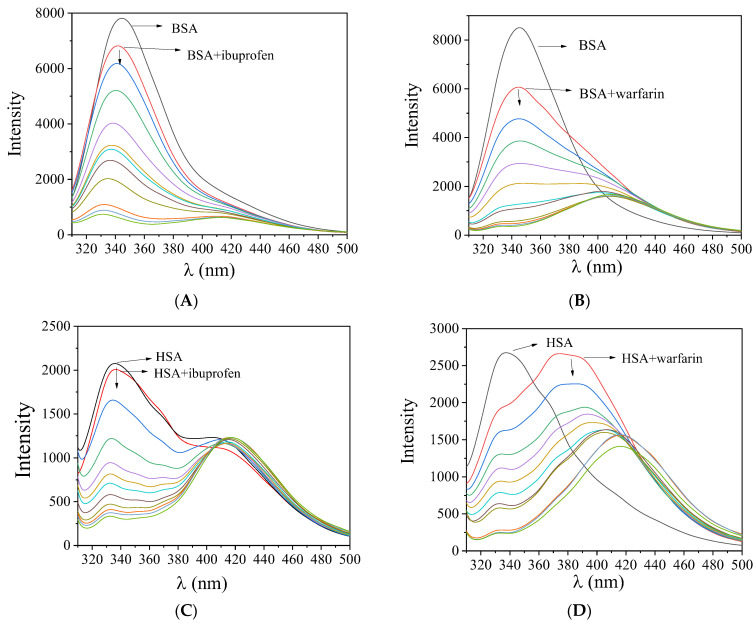
Fluorescence emission spectra (λ_excitation_ = 295 nm) for BSA (3 μM) in the presence of (**A**) ibuprofen and (**B**) warfarin in buffer solution (150 mM NaCl and 15 mM trisodium citrate at pH 7.0) upon addition of increasing amounts of complex **1** and **2**, respectively. Fluorescence emission spectra (λ_excitation_ = 295 nm) for HSA (3 μM) in the presence of (**C**) ibuprofen and (**D**) warfarin in buffer solution (150 mM NaCl and 15mM trisodium citrate at pH 7.0) upon addition of increasing amounts of complex **2**. The arrows show the changes of intensity upon increasing amounts of complex.

The drug sites where the compounds may bind to the albumins were specified via competitive experiments with warfarin and ibuprofen monitored by fluorescence emission spectroscopy. The addition of the compounds into a pre-treated solution containing SA and the site-marker (warfarin or ibuprofen) resulted in a significant quenching of the initial fluorescence emission band (representatively shown in Figure 6). In order to specify the preferable drug site, the SA-binding constants of the compounds in the presence of warfarin or ibuprofen were calculated (with the Scatchard equation (Equation (S5)) and plots (Figures S10–S13)) (Table 6) and are compared with those determined in the absence of any site-marker. A decrease in the value of K in presence of the site-marker shows that the binding of the compound to albumin is influenced by the presence of this marker due to competition for the same binding site [52,53].

In the case of BSA, complexes **1**–**3** did not show any preference towards drug site I or II since the decreased SA-binding constants are rather close and may not suggest any site selectivity, which is firmly shown by complex **4** towards drug site I and complex **5** for drug site II. In the case of HSA, complexes **1**–**4** showed a preference to bind at drug site I, while complex **5** did not seem to show firmly a selectivity between Sudlow’s sites 1 and 2 [52,53].

### 2.7. In Silico Molecular Docking Studies

Molecular docking calculations were employed to evaluate the ability of complexes **1**–**5** to bind to CT DNA, *E. coli* and *S. aureus* DNA-gyrases, 5-LOX, and FLAP bio-macromolecules, in order to explain the in vitro activity of these compounds. The best-scored pose of docked compounds in each target macromolecule was selected for the evaluation of binding interactions. Binding free energy for each pose was also computed and poses with the lowest free binding energy were selected for further visualization studies. Binding energies for the best docking poses of all macromolecules are shown in Table 7. From these data, it is obvious that compound **1** seems to succeed in better binding (lower binding energy) with CT DNA and 5-LOX, and **3** with *E. coli* and *S. aureus* DNA-gyrases, and FLAP. The conducted in silico studies were found in excellent agreement with the observed in vitro activities of the compounds (details are reported in each section).

#### 2.7.1. Docking Calculations on CT DNA

The order of decreasing binding capacity (from lower to higher global binding energy) of compounds **1**–**5** to CT DNA target the bio-macromolecule (crystal structure PDB ID number: 1BNA) was calculated to be **1** ≥ **3** > **5** > **2** > **4** (Table 7).

**Figure 7:**
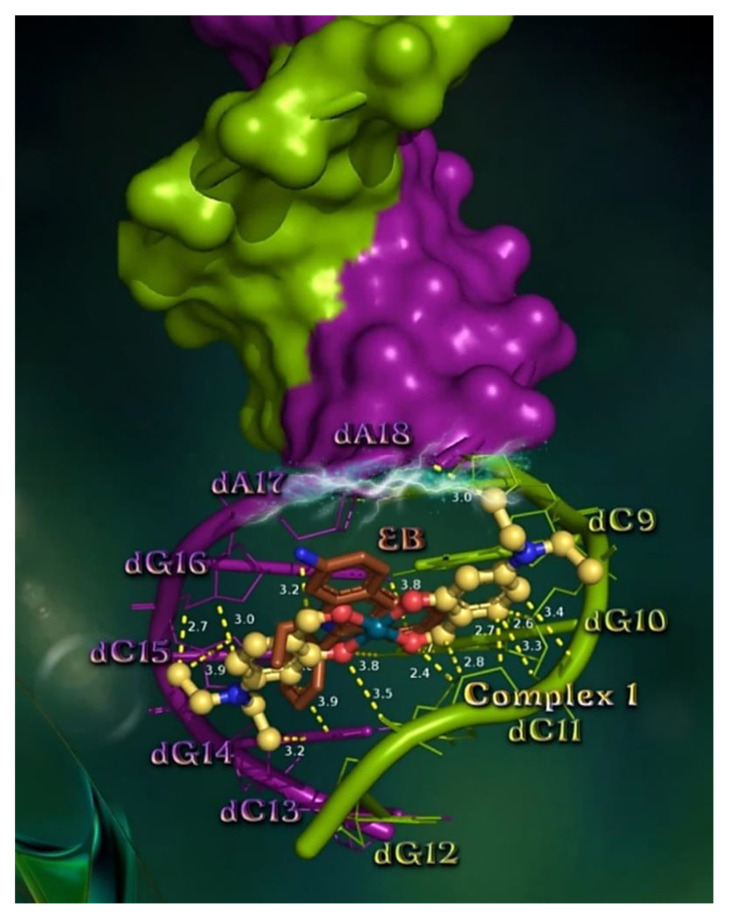
Binding pose architecture of complex **1** and EB in the crystal structure of CT DNA (PDB: 1bna) depicting its stabilization in the binding cavity of the minor groove of DNA. The ligand binding site illustrates the binding interactions of **1** in the crystal structure of CT DNA (lower panel). DNA-structure is illustrated as both opaque surface and cartoon representation, in split pea green and deep purple (clones A and B, respectively). Base pair nucleotides are rendered in line representations and color-coded according to DNA strand color. Docked molecules are rendered in ball-and-stick (**1**) and stick (EB) modes colored according to atom type in yellow, orange and brown C atoms, respectively. Dotted lines in yellow indicate hydrogen bond, polar, π–polar type, π–alkyl hydrophobic type and classic hydrophobic interactions, between the docked molecule and the nucleotides in the binding pocket of DNA. Heteroatom color-code: O: red, N: blue, and Pd: deep teal. Hydrogen atoms are omitted from all molecules for clarity. Nucleotides are numbered according to PyMol software. The final structure was ray-traced and illustrated with the aid of PyMol Molecular Graphics System. Binding interactions with nucleotide molecules are shown in Table S2.

The best-scored pose of docked compound **1** (the one exhibiting the best binding) was selected for evaluating the binding interactions. The binding of **1**, as well as the DNA intercalator EB, in the crystal structure of the CT DNA are illustrated in Figure 7, depicting a docking orientation that stabilizes the compound in the binding cavity of the minor groove. Our model for the predicted binding pose of **1** on CT DNA suggests intercalation of the compound with A (in split pea green) and B (in deep purple) helices of DNA inside the minor groove, anchored between purines and pyrimidines of both DNA strands via intrastrand penetration (of the same strand) as well as interstrand penetration (between opposite complementary strands) at the same place occupied by EB, sharing common contacts with it. It should be noted that complex **1** is inserted in the minor groove of DNA in such a way that its plane forms a 32° dihedral angle with the nucleotide planes. Complex **1** seems to be anchored in the minor groove of CT DNA via hydrogen bond (Hb), hydrophobic (Hph), polar (P), π–polar type, and π–alkyl hydrophobic interactions. The binding contacts of **1** include nucleotides dC9, dG10, dC11, and dG12 of one strand (A), and dA17, dG16, dC15, dG14, and dC13 of the other (B). The interactions of **1** inside the minor groove are cited in detail in Table S2. Complex **1**, despite its relative bulk size and the fact that it is inserted in the more regionally restricted minor groove of DNA, adopts an orientation that allows it to enter the minor groove by its whole structure due to its flat conformation, not leaving any of its ligands protruding out of the minor groove of the double-helical DNA structure, thus attaining a deep penetration making numerous contacts with the nucleotides of the binding pocket, with a consequence of low binding energy. The molecule is predicted to be inserted between the hydrogen-bonded G-C base pair nucleobases in almost a parallel position, inducing distortion of the G-C base pair interstrand connection, resulting in a perturbation in the canonical structure of the double helix, influencing thus the functional role of the DNA. The three sequential base pair impairments (dG14≡dC11, dG10≡dC15, and dG16≡dC9) induced by interruption of interstrand Hb base pairings are shown in Table S2.

Complex **1** is anchored inside the minor groove with the involvement of critical Hb, P, and π–polar interactions with nucleotides of the three base pairs, interrupting beyond the interstrand and also the intrastrand Hb stabilization of the double-strand and single-strand of DNA. In this way, it achieves additional interruption of the intrastrand CpG base pair steps of the same strand, thus contributing to the destabilization in the overall helical model. Specifically, **1** is placed in a position to influence the CpG intrastrand base pair steps dG16pdC15 and dC15pdG14 of one strand, and dC9pdG10 and dG10pdC11 of the other, affecting the single-stranded geometric stabilization of neighboring nucleotides.

The above in silico study is in accordance with the in vitro DNA experiments indicating the following order of activity: DNA-binding constants (K_b_) showing the magnitude of the interaction of complexes with DNA: **3** > **5** >> **1** > **2** > **4**, and quenching constants (k_q_) revealing the possible intercalative mode of interaction of complexes with EB-DNA: **1** ≥ **2** > **3** > **4** > **5**. Furthermore, the higher values of Stern–Volmer constants (K_SV_) indicate tight binding to DNA: **1** ≈ **2** >> **3** > **4** > **5**. The values of K_b_ (Table 3) and calculated ΔG_bind_ binding energies (Table 7) are in good agreement. Complexes **3** and **5** were revealed to have the highest K_b_ constant among the complexes, followed by **1**, **2** and **4**, with the latter having the lowest K_b_ constant; that is, the weakest binding. From the ΔG_bind_ binding energies, it is deduced that complexes **3** and **1** exhibit the lowest energy corresponding to the highest binding capacity which is in agreement with the highest K_b_ constant of **3**. On the other hand, the highest energy (lowest binding capacity) is calculated for complex **4**, which also exhibits the lowest K_b_ constant. Furthermore, complex **2** possesses the second higher energy and at the same time the second lower K_b_ constant. The above results indicate the excellent agreement between in vitro and in silico studies, indicating the possibility of an intercalative mode of action.

Apart from the role of DNA in the mitosis phase of the cell cycle and the propagation of the cells, there also exist numerous bindings of proteins to DNA which are important in many biological processes to control transcription and replication. Some proteins may bind to single-stranded DNA and others to double-stranded DNA. Complex **1**, by its interrupting activity on the canonical base pairing of DNA, may play a role in the inhibition of many processes promoting a plethora of diseases.

#### 2.7.2. Docking Calculations on *E. coli* and *S. aureus* DNA-Gyrase

DNA-gyrase is a topoisomerase type II enzyme that has attracted attention since its discovery in 1976, when it was first isolated from *E. coli* [54]. DNA-gyrase catalyzes changes in DNA-topology by breaking and rejoining double-stranded DNA, introducing negative supercoils of the closed-circular DNA in front of the replication fork [55]. It is the only enzyme that can actively underwind (i.e., negative supercoiling) the double helix [56]. Since this function is essential for DNA replication and transcription, DNA-gyrase is really a suitable target for antibacterial agents. In order to achieve a rational approach in the mechanism of the antibacterial activity of complexes **1**–**5,** its role in the inhibition of DNA-gyrase was probed via a computational approach.

**Figure 8:**
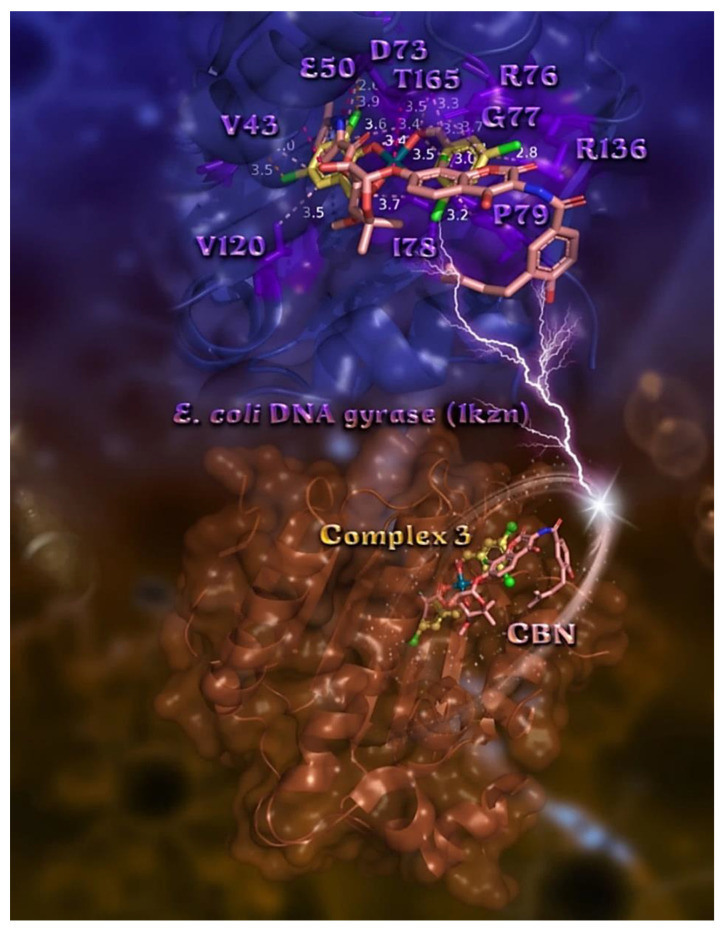
(**Lower panel**) Docking pose orientation of best (lowest ΔG_bind_ energy) bound complex **3** (rendered in a ball-and-stick mode for lower panel and stick in the upper panel, and colored according to atom type in yellow-orange C atoms) superimposed with the co-crystallized drug CBN (salmon C atoms rendered in stick representation) in the crystal structure of *E. coli* DNA-gyrase (PDB ID: 1kzn). The target protein is illustrated as a cartoon colored in brown with depth cue in the ray-tracing rendering of the whole structure with an additional depiction of semi-transparent surface colored according to the cartoon. (**Upper panel**) A close-up view of the ATP-binding site architecture of the best (lower energy ranking) binding pose of **3** superimposed with CBN. The target protein is illustrated as both semi-transparent cartoon and surface colored in deep blue. Selected critical contacting amino acid residues of the binding pocket are rendered in a stick model and colored in purple-blue. Binding contacts of **3** are shown as yellow dotted lines. Heteroatom color-code: O: red, N: blue, Cl: chartreuse green, and Pd: deep teal. Hydrogen atoms are omitted from all molecules for sake of clarity. The final structure was ray-traced and illustrated with the aid of PyMol Molecular Graphics Systems. Binding interactions are shown in Table S3.

**Figure 9:**
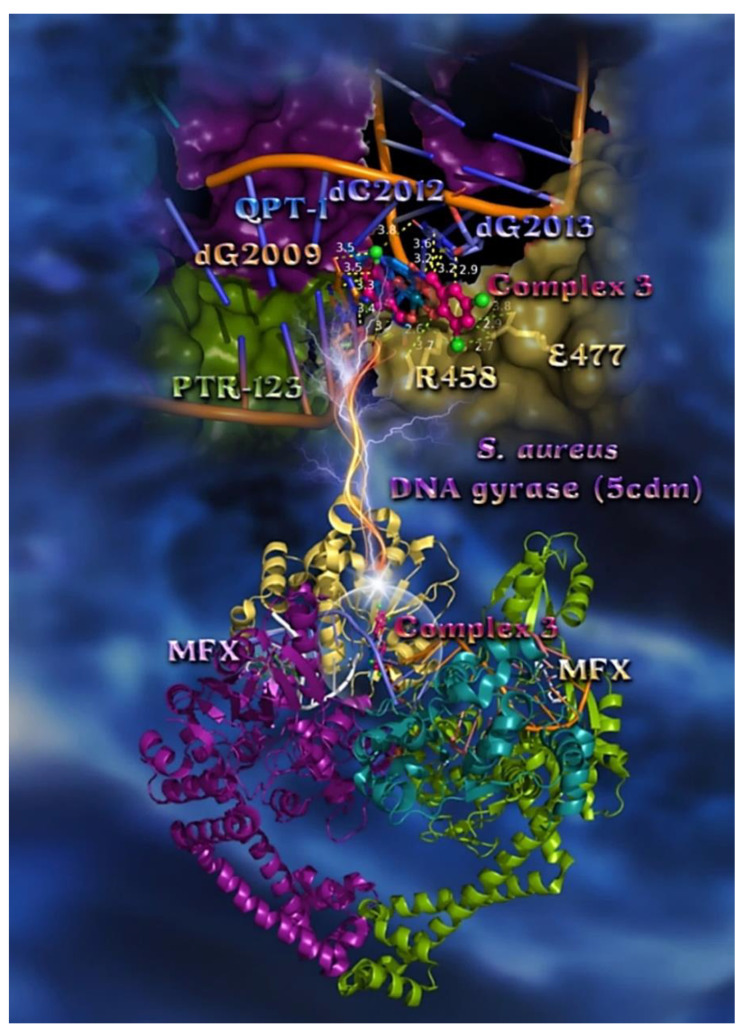
(**Lower panel**) Docking pose orientation of best (lowest ΔG_bind_ energy) bound complex **3** in the crystal structure of *S. aureus* DNA-gyrase (PDB ID: 5cdm). Superimposed are also illustrated the active against *S. aureus* DNA-gyrase drugs MFX (docked) and QPT-1 (co-crystallized). MFX is docked in two binding poses (one lower and one higher binding energy) at the same binding pocket of the protein at the edge of DNA, while complex **3** is anchored at the same binding pocket with the co-crystallized drug QPT-1. The target protein is illustrated as a cartoon with sub-domains color-coded in split pea green, deep teal, deep purple, and yellow-orange colors for chains A, B, C, and D, respectively. An artificially nicked double-stranded DNA interacting with DNA gyrase is also depicted in a cartoon colored in salmon and orange for complementary strands E and N, and white and slate blue for complementary strands F and I. Docked molecules rendered in ball-and-stick and stick (complex **3** and drugs, respectively) model are colored according to atom type in hot pink (**3**), marine blue (QPT-1), and white and light pink (lowest and higher binding energy poses of MFX, respectively). The L-peptide linking amino acid residue o-phosphotyrosine (in split pea green sticks) is also shown in the structure. (**Upper panel**) A close-up view of the binding of **3** in both DNA and DNA-gyrase complex structure, depicting the extent of the binding pocket as determined by the computation process and the crystal structure as well. Double-stranded DNA and DNA-gyrase are depicted in a cartoon and the opaque surface, respectively, colored in the same scheme as in the lower panel. Molecular docking simulations of all molecules were performed individually. Binding contacts are shown as dotted yellow lines. Heteroatom color-code: O: red, N: blue, Cl: chartreuse green, and Pd: deep teal. Hydrogen atoms are omitted from all molecules for clarity. The final structure was ray-traced and illustrated with the aid of PyMol Molecular Graphics Systems. Binding interactions are shown in Table S3.

The order of decreasing binding capacity (from lower to higher global binding energy) of compounds **1**–**5** to *E. coli* and *S. aureus* DNA-gyrases target enzymes (PDB: 1KZN, and 5CDM, respectively) was calculated to be **3** > **4** > **2** > **5** ≥ **1** > CBN (*E. coli* DNA-gyrase) and QPT-1 > MFX > **3** ≥ **1** > **5** > **2** > **4** (*S. aureus* DNA-gyrase) (Table 7). The bindings of the best-docked complex **3** in the crystal structure of *E. coli* and *S. aureus* DNA-gyrases, as well as their co-crystallized drugs chlorobiocin (CBN), moxifloxacin (MFX), and QPT-1, are depicted in Figure 8 and Figure 9, where the best-fitted docking pose of each molecule inside the ATP-binding site of DNA-gyrase is shown.

The docking pose orientation of best bound (lower energy) complex **3** in the crystal structure of *E. coli* DNA-gyrase superimposed with CBN is depicted in the lower panel of Figure 8. Based on the binding energies (Table 7) and the binding interactions in its binding pocket (Table S3), complex **3** ensures better binding capacity compared to CBN. Compound **3** is shown to be stabilized inside the ATP-binding site of DNA-gyrase and anchored in the same place as CBN. For the docking experiments, we chose to use the DNA-gyrase in a complex with the bound co-crystallized drug CBN, which includes only subunit B, exhibiting the crucial ATPase activity (subunit A is mainly involved in DNA breakage and reunion) [57]. Both molecules, complexes **3** and CBN, are shown to be stabilized inside the same binding pocket of the protein occupied by the co-crystallized drug CBN [58]. The ligand-binding site of both compounds depicts the extent of the pocket as determined by the computation process, labeling the critical residues interacting with the molecules as shown in the upper panel of Figure 8. The docking procedure predicts the formation of a variety of interactions between both complexes and the amino acid residues Val (V43), Glu (E50), Asp (D73), Arg (R76), Gly (G77), Val (V120), and Thr (T165) (upper panel of Figure 8 and Table S3). Stabilization of **3** may be attributed to Hb, π–alkyl Hph, π–cation and π–anion charged electrostatic interactions, π–polar, and halogen bond contacts inside the ATP-binding site of DNA-gyrase protein. Further stabilization of **3** in the protein is achieved as hydrophobic protein atoms of Ile (I78) and Pro (P79) residues enclose the hydrophobic region of phenyl rings of **3**, making Hph contact them. All these contacts were found to be common with those of CBN.

The terminal methyl-pyrrole ring of CBN is deep buried in the binding pocket while the other terminal moiety 4-hydroxy-3-(3-methylbut-2-enyl)benzoyl]amino is anchored in the entrance of the pocket. On the other hand, complex **3** seems to be inserted deeper in the ATP-binding pocket of the enzyme, with one 3,5-diCl-salo ligand and phenolato and carboxylato oxygen atoms anchored at the entrance of the pocket, but much deeper compared to CBN. This 3,5-diCl-salo ligand of **3** lying at the entrance of the pocked is stabilized with π–polar and π–alkyl interactions with E50, R76, G77, P79, and R136 residues, and halogen bond interactions with P79 by one of its Cl atoms and a cation–dipole halogen bond of the other Cl atom with R76. On the other hand, both Cl atoms of the second 3,5-diCl-salo ligand were found to be buried deep inside the catalytic pocket, contributing to the stabilization of **3** within the protein’s binding pocket with the participation of two Dinitz multipolar halogen bond interactions with the carbonyl oxygens of D73 and V43.

The best-fitted docking poses of complex **3**, exhibiting the highest in silico binding capacity in the crystal structure of *S. aureus* DNA-gyrase superimposed with MFX and QPT-1, are depicted in the lower panel of Figure 9. Binding interactions of **3** in its binding pocket are reported in Table S3. A close-up view of the binding of **3** in both DNA and DNA-gyrase complex structures, at exactly the same place occupied by the co-crystallized drug QPT-1 and depicting the extent of the binding pocket as determined by the computation process and the crystal structure, is illustrated in the upper panel of Figure 9. On the contrary, MFX is shown to be stabilized in a binding cleft located at the edge of the artificially nicked DNA strand. MFX is docked in two binding poses (one lower and one higher binding energy) at the same binding pocket of the protein at the edge of DNA, while complex **3** is anchored at the same binding pocket as the co-crystallized drug QPT-1. Further stabilization of **3** is achieved with the participation of interaction with L-peptide-linking amino acid residue o-phosphotyrosine. Complex **3** is shown to penetrate deep in the double helix of DNA stabilized at the site of the artificially nicked double-stranded DNA, being inserted with its plane in parallel to the nucleotide plane of dG2009 of clone N, and making contacts with dG2009 and with dG2013 of clone I by π–π sandwich, π–polar, and halogen bonds, interrupting the interstrand GC base pairing dG2009≡dC2012 of clones N and I, respectively, and dG2013≡dC8 of clones I and F, respectively. The docking procedure predicts the formation of a variety of interactions between **3** and the amino acid residues R458 and E477 via Hb, π–alkyl, π–anion, and halogen bond contacts, all belonging to chain D of the protein.

The in vitro antimicrobial activity of complexes **1**–**5** against *E. coli* and *S. aureus* strains are ordered as **2** > **3** >> **1** > **5** > **4** for *E. coli* and **2** > **3** >> **1** > **5** > **4**, for *S. aureus* (Table 2), with compounds **2** and **3** being four-to-five times higher in activity compared to the rest of the compounds. It is obvious that there is a consistency between in vitro and in silico studies, indicating that complex **3** possesses the highest binding capacity and second-best antimicrobial activity. At the same time, complex **4** in the *S. aureus* strain was found to possess the worst binding capacity in the in silico studies and the least activity in vitro. From the above results, it may be deduced that the predicted binding energy values correlated well with the observed experimental values.

#### 2.7.3. Docking Calculations on 5-LOX and FLAP Target Proteins

Τhe ability of compounds **1**–**5** to bind to 5-LOX and FLAP was explored in order to explain the described antioxidant activity. LOXs form a heterogeneous class of enzymes that catalyze the peroxidation of polyunsaturated fatty acids such as arachidonic acid (AA). 5-LOX is the most predominant isoform associated with the formation of 5-hydroperoxyeicosatetraenoic acid (5-HpETE), the precursor of non-peptido leukotriene B4 (LTB4) and cysteinyl leukotrienes (LTs) (LTC4, LTD4, and LTE4). 5-LOX is the central enzyme in cellular leukotriene biosynthesis and requires a set of stimulatory factors for full activity and is supported by two accessory proteins, FLAP and coactosin-like protein (CLP) [59]. The integral membrane protein FLAP is essential for leukotriene biosynthesis. Inhibition of leukotriene biosynthesis has been extensively studied as a potential for the development of novel therapies for inflammation and allergic disorders such as respiratory diseases and, in particular, asthma, ulcerative colitis, rhinitis and cancer. Consequently, 5-LOX has become a target for the development of therapeutic agents for the treatment of various inflammatory disorders [60]. Furthermore, FLAP inhibitors are anticipated to reduce proinflammatory leukotriene mediators such as the potent chemotactic agent LTB4. FLAP inhibitors have been proven safe and efficacious in asthma clinical trials but have not been brought to the market as yet. Additionally, accumulating evidence indicates that leukotrienes mediate the pathophysiology of acute brain injuries and chronic neurodegenerative diseases. These findings have generated a renewed interest in FLAP inhibitors as interventional therapies for these disorders. The catalytic iron center of activated 5-LOX converts AA in a two-step concerted reaction. Inhibition of LOX enzymes is generally thought to be implicated in the apoptotic and antiproliferative activity of compounds [61] and can especially down-regulate the progression of colorectal and pancreatic cancer. Since the mechanism of the enzyme inhibition may include the reduction of lipidperoxy or lipidoxy radicals [62], LOX inhibition may correlate with the ability of the studied complexes to scavenge the free stable radical DPPH.

In order to explain the described antioxidant activity of compounds **1**–**5** with the aid of a computational approach, molecular docking studies of the compounds on 5-LOX and FLAP target proteins were employed. The order of decreasing binding capacity (from lower to higher global binding energy) of compounds **1**–**5** and Trolox to the crystal structure of the 5-LOX target enzyme bound with the redox-type inhibitor NDGA (PDB: 6N2W) was calculated to be **1** > **3** > **5** ≥ Trolox > **2** > **4** (Table 7). The corresponding values of compounds **1**–**5** to the FLAP target protein bound with its inhibitor MK-591 (PDB: 2Q7M) was calculated to be **3** > **1** > **2** > **4** > **5**. It is obvious that complexes **1** and **3** display the best binding capacity to 5-LOX and FLAP target proteins, respectively. At the same time, it is deduced that complex **1** exhibits the best scavenging activity against ABTS, while complex **3** displays the second-best in vitro H_2_O_2_ reducing activity among the studied compounds (Table 1), revealing consistency between in silico and in vitro experiments. Thus, the best-scored pose of docked compounds **1** and **3** was selected for evaluation of the binding interactions in 5-LOX and FLAP target proteins, respectively.

##### Docking Calculations on 5-LOX

**Figure 10:**
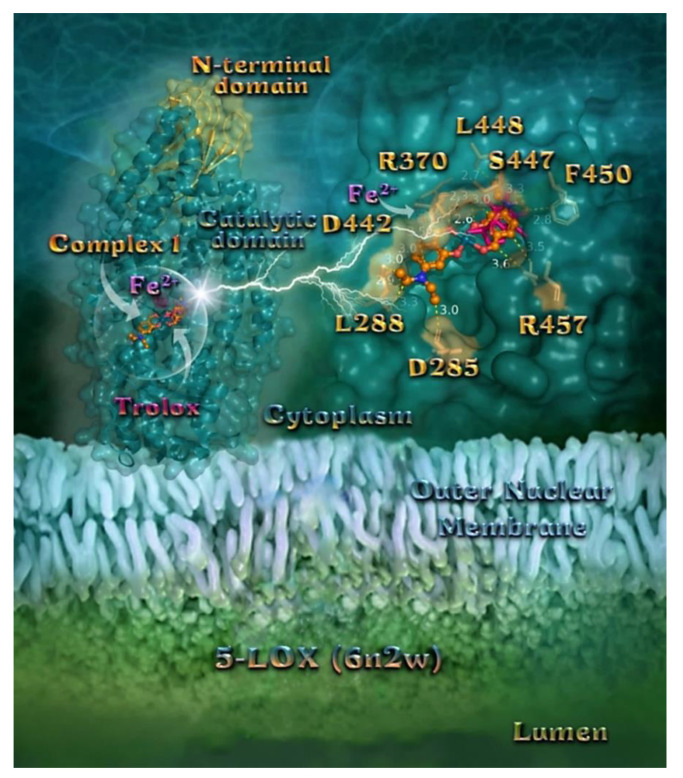
(**Left panel**) Docking pose orientation of complex **1** in the crystal structure of 5-LOX bound with the redox-type inhibitor NDGA (PDB: 6n2w). The target protein is illustrated as a semi-transparent surface in deep teal (catalytic domain) and yellow-orange (N-terminal domain). In superposition are shown the lowest ΔG_bind_ energy binding pose of **1**, as well as the co-crystallized inhibitor NDGA and Trolox, rendered in a ball-and-stick model (**1**) and stick (NDGA and Trolox) and colored according to atom type in orange, white, and hot pink C atoms, respectively. Fe^2+^ is indicated as a violet dotted sphere. Molecular protein structure is shown to be anchored in the outer nuclear membrane colored in a light blue/green model. (**Right panel**) A close-up view of the binding pocket of **1** in the crystal structure of 5-LOX bound with Trolox (6n2w). The target protein is depicted as a semi-transparent deep teal surface with the additional depiction of selected contacting residues of the binding pocket highlighted in yellow-orange. Complex **1** and Trolox are rendered in ball-and-stick and stick models, respectively, colored according to atom type in orange and hot pink C atoms, respectively. Molecular docking simulations of all molecules were performed individually. Heteroatom color-code: O: red, N: blue, Cl: chartreuse green, Pd: grey. Hydrogen atoms are omitted from all molecules for clarity. The final structure was ray-traced and illustrated with the aid of PyMol Molecular Graphics Systems. Binding interactions are shown in Table S4.

The superposition of complex **1,** along with the co-crystallized inhibitors NDGA and Trolox, is illustrated in Figure 10. Docked molecules were revealed to be anchored in the active site of 5-LOX, at the catalytic domain of the enzyme (left panel of Figure 10). A close-up view of the ligand binding pocket of **1** in the crystal structure of 5-LOX (right panel of Figure 10) shows that **1** is stabilized in the binding pocket at the same place occupied by Trolox. Fe^2+^ ion in the active center of the enzyme is depicted in violet dotted sphere representation. Non-heme iron is harbored in the primarily α-helical catalytic domain of the enzyme. The docked molecule is anchored in the active site of 5-LOX. In the lower position of the protein structure is illustrated the larger catalytic domain, while in the upper position is located the N-terminal β-barrel “C2-like” domain of the enzyme which confers Ca^2+^-dependent membrane binding. The binding interactions of **1** on the catalytic domain of 5-LOX are reported in Table S4. The docking orientation of **1** stabilizes the compound in a binding cavity of the enzyme via Hb, polar, Hph, π–alkyl Hph, π–cation and π–anion electrostatic, and π–polar interactions. Binding contacts of **1** include the residues Asp D285, Leu L288, Arg R370, Asp D442, Ser S447, Leu L448, Phe F450, and Arg R457. Common binding contacts of **1** with those of Trolox appeared to be R370, S447, F450, and R457 residues. The 5-LOX pathway’s end product is LTB4, a mediator of several inflammatory and allergic diseases, including atherosclerosis, cancer, and cardiovascular diseases. Thus, reducing the LTs via inhibiting 5-LOX may help to reduce the potential risk of cardiovascular and gastrointestinal disease caused by selective COX-2 and COX-1 inhibitors, respectively [63,64].

##### Docking Calculations on FLAP

**Figure 11:**
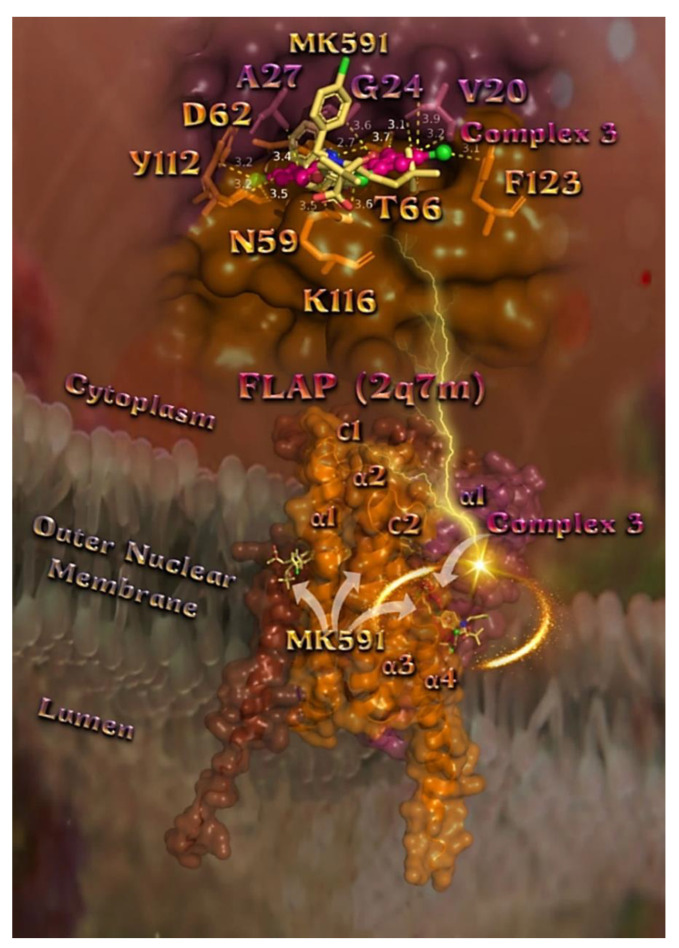
(**Lower panel**) Docking pose orientation of best-bound complex **3** on the crystal structure of FLAP (PDB: 2q7m) bound with FLAP inhibitor MK-591. The target protein is a homotrimer folded into three domains, illustrated as a cartoon and colored by the chain in orange, raspberry, and chocolate for the three domains d (catalytic), e (C-terminal), and f (N-terminal), respectively, between which the catalytic center is located, with the additional depiction of semi-transparent surface colored according to cartoon. FLAP trimer protein is localized at the outer nuclear membrane with each monomer composed of four transmembrane α-helices connected by two cytoplasmic loops and one lumenal loop. Complex **3** is docked at the same binding pocket with co-crystallized drug MK-591. Complex **3** and MK-591 are rendered in ball-and-stick and stick mode, respectively and colored according to atom type in hot pink and yellow-orange, respectively. The lowest energy binding pose of **3** is stabilized at the center of the catalytic site in an intermonomeric cleft between domains d and e formed by helices α1 of chain e (raspberry), and α2, α4 of chain d (orange). Lumen indicates the perinuclear space between outer and inner nuclear membranes. (**Upper panel**) A close-up view of the binding pocket of **3** in the crystal structure of FLAP. The target protein is depicted as a semi-transparent surface colored according to the cartoon. Complex **3** is illustrated to be anchored at the same place with MK-591 (both rendered and colored as in the lower panel), with the additional depiction of selected contacting residues of the binding pocket rendered in a stick model and colored according to the chain. Binding contacts are shown as dotted yellow lines. Heteroatom color-code: O: red, N: blue, Cl: chartreuse green, Pd: grey. Hydrogen atoms are omitted from all molecules for clarity. The final structure was ray-traced and illustrated with the aid of PyMol Molecular Graphics Systems. Binding contacts shown as dotted yellow lines are mentioned in Table S4.

The superposition of complex **3** along with the co-crystallized inhibitor MK-591 is illustrated in Figure 11. Both compounds seem to be stabilized at the same binding pocket sharing common binding contacts. Complex **3** binds to a membrane-embedded pocket in FLAP, suggesting how it might prevent the binding of AA to the active site of the enzyme. Complex **3** in its lowest energy binding pose is stabilized at the center of the catalytic site in an intermonomeric cleft between domains d and e formed by helices α1 of chain e (raspberry), and α2, α4 of chain d (orange). Complex **3** seems to be anchored in a binding crevice of the protein via Hb, polar, Hph, Hb σ-hole, halogen bond, halogen–π, π–alkyl Hph and π–polar interactions. It is shown to intercalate between monomers in the surface grooves forming interactions with residues Val (V20), Val (V21), Gly (G24), and Ala (A27) of helix α1 belonging to chain E, Asn (N59), Asp (D62), and Thr (T66) of helix α2 of chain D, Lys (K116) and Phe (F123) of helix α4 of chain D, and Tyr (Y112) of the C2 loop connecting helices α3 and α4, all belonging to chain D. The structure model shows that the binding site of complex **3** is located within the nuclear membrane, which provides an appropriate environment for the lateral diffusion of AA molecules to FLAP. The structures show that inhibitors bind in membrane-embedded pockets of FLAP, which suggests how these inhibitors prevent AA from binding to FLAP and subsequently being transferred to 5-LOX, thereby preventing leukotriene biosynthesis.

### 2.8. Chemical Reactivity

In order to decipher the discrepancy between in vitro and in silico results, a computational calculation approach to the electronic structure of complexes **1**–**5** was adopted. To this end, DFT single-point energy calculations were carried out at two different levels of theory, namely B3LYP with the inclusion of6-31G*(d,p) and LANL2DZ double-ζ basis set (for *cis* and *trans* orientations), and also the greater computational cost ωB97X-D with cc-pVTZ triple-ζ basis set (for *trans* orientations) to describe the accurate structural and electronic properties of the compounds implemented by the Spartan’ 14 program suite. For complexes **2**–**5**, *trans* conformation for each structure was revealed to be more stable (lower energy). The electronic distribution information of the compounds was theoretically determined through orbital energy calculations of the highest occupied and lowest unoccupied molecular orbitals (HOMOs and LUMOs, respectively), which determine nucleophilic and electrophilic activity. The high HOMO energy corresponds to the more reactive molecule in the reactions with electrophiles, while low LUMO energy for molecular reactions with nucleophiles [65]. The binding of **1** to nucleobases is shown to be governed among others by hydrogen bond contact with the amino group of dG10 and dG16 guanine (acting as an H-bond donor). Similar binding contacts are revealed also for the rest complexes (data not shown). The energy gap between HOMO and LUMO of the reactants theoretically can be used to understand the biological activity of the compounds since the lower HOMO–LUMO gap explains the eventual charge transfer interactions taking place within the molecules [66,67].

The frontier molecular orbitals (FMOs) play an important role in the electronic and optical properties and their energy gap is a critical parameter in determining molecular electrical transport properties [68]. The negative magnitude of FMOs indicates the stability of the synthesized complexes. The energy gap ΔΕ_LUMO__-__HOMO_ between the HOMO orbital of each complex and the LUMO orbital of dG is examined. Since the LUMO value is the same, the higher the energy of HOMO the more closed the energy gap with the LUMO of dG, resulting in enhanced binding. From Table 8, it is deduced that the E_HOMO_ energy (higher to lower) for all complexes followed the order: **1** > **5** > **4** > **3** ≥ **2**. It is thus obvious that the gap energy ΔΕ_LUMO__-__HOMO_ would follow the reverse order: **1** < **5** < **4** < **3** ≤ **2**.

The same order is revealed by examining the chemical reactivity descriptors of these compounds. Chemical global reactivity indices such as chemical hardness (*η*), softness (*s*), electronegativity (*χ*), and electronic chemical potential (*μ*) to deduce the relations among energy, structure, and reactivity characteristics of the complexes have been evaluated (Table 8) from HOMO and LUMO frontier molecular orbitals energy values using DFT calculations, according to Koopman’s theorem [69]. The aforementioned as well as other electronic indices such as atomic charges, dipole moments, total energies, and heats of formation are generally used for the analysis of structure–activity relationships [70].

Chemical hardness (*η* = (E_LUMO_ − E_HOMO_)/2, where E_LUMO_ and E_HOMO_ are the LUMO and HOMO energies of the complex, respectively) is associated with the stability and reactivity of a chemical system [71]. In a molecule, it measures the resistance to change in the electron distribution or charge transfer. On the basis of frontier molecular orbitals, chemical hardness corresponds to the gap between the HOMO and LUMO orbitals. The larger the HOMO–LUMO energy gap (larger *η*), the harder, the stabler, the less polarizable and less reactive the molecule will be [72]. The soft systems have a small HOMO–LUMO gap, large, and highly polarizable. Thus, the computed chemical hardness values of the complexes are ranked as: *η***2** ≈ *η***3** < *η***4** < *η***1** = *η***5**, demonstrating that **5** and **1** are less reactive than **4,** which is less reactive than **2** and **3**. Complexes **2** and **3** were revealed to be more reactive than **4**, **1** and **5** which are characterized by their resistance toward deformation of the electron cloud of the chemical system under small perturbations and are less polarizable.

The softness (*s* = 1/(2*η*)) values for the complexes are ranked as: *s***2** ≈ *s***3** > *s***4** > *s***1** = *s***5**, resulting again in higher reactivity for **2** and **3** (the small magnitude of these values also excluded the possibility of their soft nature).

The concept of electronegativity (*χ* = −(E_HOMO_ + E_LUMO_)/2) put forward by Pauling is defined as “the power of an atom in a molecule to attract electrons towards itself” [73]. The higher the electronegativity of the species is, the greater its electron accepting power, or rather, its electrophilicity. The *χ* values of the complexes are ranked in the order (from higher electronegativity or electrophilicity to lower values): *χ***2** > *χ***3** > *χ***4** > *χ***5** > *χ***1**.

The electronic chemical potential (*μ*), defined as the negative electronegativity of a molecule, is determined as *μ* = (E_HOMO_ + E_LUMO_)/2 and describes the escaping tendency of electrons from an equilibrium (stable) system. The negative values of *μ* indicate a stable complex that does not undergo decomposition spontaneously into its elements. Thus, the μ values of the complexes are in the order (more stable, i.e., more negative values, to less stable, i.e., less negative values): *μ***2** > *μ***3** > *μ***4** > *μ***5** > *μ***1**, suggesting that these complexes do not decompose into elements, implying less stability for **1**.

The global electrophilicity index (*ω* = *μ*^2^/(2*η*)) is related to the ability of an electrophile to acquire additional electronic charge from the environment and the resistance to exchange electronic charge with the environment, providing information about both electron transfer (chemical potential) and stability (hardness). The electrophilicity index (*ω*) is one of the most important quantum chemical descriptors to ascertain the toxicity of molecules in terms of their reactivity and site selectivity [74], thereby quantifying the biological activity of drug–receptor interaction. The global electrophilicity index (*ω*) assesses the lowering of energy due to maximal electron flow between the donor and acceptor calculated from the HOMO–LUMO energy values. The highest electrophilic deactivation for compound **1** is observed (Table 8) (lower to higher *ω* index order: *ω***1** < *ω***5** < *ω***4** < *ω***3** < *ω***2**), thus implying less toxicity between all the compounds. In contrast, the substitution of the 4-Et_2_N-salo ligand with 3,5-diBr-salo shows the highest electrophilic activation. Since low *ω* has been correlated with good nucleophiles [75], the lowest *ω* is in agreement with the lowest ΔΕ_LUMO-HOMO_ value for complex **1**. The order of increasing size for F < Cl < Br in the ligand moieties of **4**, **3**, and **2** complexes, respectively, results in a corresponding increase in the electrophilic activation (*ω***4** < *ω***3** < *ω***2**).

The computed frontier molecular orbitals HOMO and LUMO, as well as the molecular electrostatic potential (MEP) and local ionization potential (LIP) maps, are illustrated in Figure S14. For the optimized structural models, the LUMO in the complexes is localized mainly around Pd ion and on coordinated phenolato and carboxylato oxygen atoms. On the contrary, HOMOs are diffused on both aromatic rings of the X-salo ligand moieties and secondarily and to a lower degree are localized around Pd ion. These localizations of HOMO provide an indication of electrophilic reactivity. The MEP is a plot of electrostatic potential mapped onto the constant electron density surface. The electron density isosurface is a surface on which the molecule’s electron density has a particular value and that encloses a specified fraction of the molecule’s electron probability density. An MEP map provides a picture of the overall polarity of the compounds (providing an indicator for charge distribution in the molecules). Therefore, the overall high and low electron density regions are better characterized by MEPs. MEPs have been used primarily for predicting sites and relative reactivity towards electrophilic attack, in studies of biological recognition and hydrogen bonding interactions [70]. Actually, it is known as a reliable descriptor of long-range intermolecular interactions such as hydrogen bonding [71]. MEP values defined for the sites of the molecules that are possible nucleophilic sites are well known to be reliable measures of their relative hydrogen bond accepting strengths [72]. Negative electrostatic potential corresponds to an attraction of the proton by the concentrated electron density in the molecules (from lone pairs of oxygen atoms ligated to Pd(II)) (colored in shades of red on the EPS surface), while the positive electrostatic potential corresponds to the repulsion of the proton by atomic nuclei in regions where low electron density exists and the nuclear charge is incompletely shielded (and is colored in shades of blue). Potential increases in the order red < orange < yellow < green < blue.

The magnitudes of MEP values near the coordinated Pd ion phenolato and carboxylato oxygen atoms are examined. For complexes **1**–**5**, the computed lower values (negative) of MEP located at the coordinated oxygen atoms range from 50.9 kcal/mol to −54.9 kcal/mol for **1**, −31.3 kcal/mol to −40.2 kcal/mol for **2**, −26.4 kcal/mol to −40.8 kcal/mol for **3**, −34.4 kcal/mol to −40.8 kcal/mol for **4**, and −40.6 kcal/mol to −49.0 kcal/mol for **5**. For **5**, the next lower values are located at non-coordinated methoxy oxygens with −23.1 kcal/mol and −23.6 kcal/mol.

LIP is an indicator of electrophilic addition [76]. A LIP map is a map showing the energy required to remove an electron (the ionization potential) as a function of its location on the electron density surface. The local ionization potential reflects the relative ease of electron removal (ionization) at any location around the complex molecule. By convention, red regions on a local ionization potential map indicate areas from which electron removal (ionization) is relatively easy, meaning that they are subject to electrophilic attack. These are easily distinguished from regions where ionization is relatively difficult (by convention, colored blue). For all complexes, the areas demarking localization of HOMO are also found with lower values of LIP which are most easily ionized. The sites in the molecules representing an indicator of electrophilic addition revealed to be the coordinated oxygen atoms to Pd ion (in the LIP map sites with red/yellow color). The lowest LIP values over the Pd region for complexes **1**–**5** are computed to be 1.5548 kcal/mol (for **1**), 1.8479 kcal/mol (for **2**), 1.8455 kcal/mol (for **3**), 1.7429 kcal/mol (for **4**), and 1.6444 kcal/mol (for **5**). The next higher values of LIP are located over the ο- and π-positions of aromatic carbon atoms as to coordinated phenolato oxygens with values ranging from 1.9074 kcal/mol to 2.1428 kcal/mol for **1**, 2.4877 kcal/mol to 2.5597 kcal/mol for **2**, 2.5105 kcal/mol to 2.6495 kcal/mol for **3**, 2.2731 kcal/mol to 2.4380 kcal/mol for **4**, and 2.0732 kcal/mol to 2.2445 kcal/mol for **5**, with higher values over the π-positions (except for **5** in which higher values are reported for the ο-positions). Additionally, intermediate LIP values were found over the aromatic carbon atoms bearing Br atoms for **2** (2.1523 kcal/mol to 2.2326 kcal/mol), Cl atoms for **3** (2.3693 kal/mol to 2.4934 kcal/mol), and F atoms for **4** (2.3852 kcal/mol and 2.4818 kcal/mol). Among all complexes, the highest LIP values were found over the methyl carbon atoms of **5** (3.4122 kcal/mol), nitrogen atoms of **1** (3.2539 kcal/mol), and also the halogen atoms Br of **2** (3.0478 kcal/mol to 3.0747 kcal/mol), Cl of **3** (3.2396 kcal/mol to 3.2685 kcal/mol), and F of **4** (3.8085 kcal/mol and 3.8188 kcal/mol).

The thermodynamic parameters ZPE (zero-point energy) and vibrational (v) corrections (as either Temp. Correction (Hv) or Entropy Correction (Hv-TSv)) (the total correction to the electronic energy to find the Gibbs Energy), as well as the entropy S°, enthalpy H° (which is the sum of electronic energy and zero-point energy adjusted for finite temperature), Gibbs energy G° (which is the sum of enthalpy and entropy), and heat capacity at a constant volume Cv for the *trans* conformation of complexes **1**–**5**, computed at DFT/B3LYP with the inclusion of 6-31G*(d,p)/LANL2DZ double-ζ basis set, are reported in Table 9.

Additional QSAR parameters from the CPK model were taken into consideration, such as CPK Area (surface area of a space-filling (CPK) model), CPK Volume (volume of a space-filling (CPK) model), PSA (polar surface area of a space-filling (CPK) model defined as the area due to electronegative atoms (N, O) and hydrogens attached to the atoms, CPK ovality (a measure of deviation from a spherical shape, where 1.0 = a sphere and values > 1.0 indicate deviation), and from computed wavefunction. The polarizability is reported in Table 10.

### 2.9. Molecular Pharmacokinetic Properties, Drug-Likeness, Target Proteins and Toxicity Predictions

#### 2.9.1. Molecular Properties Prediction/Drug-Likeness

The molecular physicochemical pharmacokinetics properties of complexes **1**–**5** related to Lipinski’s Rule of Five (Ro5) with the employment of the Molinspiration property engine to analyze the drug-like properties of the compounds are reported in Table S5. For all complexes **1**–**5**, excellent approximation was revealed to Ro5 with complexes **4**, **5** exhibiting no violations (highest drug-likeness score), and complexes **1**–**3** displaying only one, while still obeying Ro5 rule. It has been reported that compounds which are violating two or more of Lipinski’s rule parameters may create problems in the bioavailability [77].

Theoretical miLogP values for complexes **1**–**5** were found to be below 5, suggesting, according to Ro5, good permeability across the cell membrane. According to Veber’s rule, all complexes exhibit TPSA (Topological Polar Surface Area) values lower than 140 Å^2^ and the number of rotatable bonds below 10 and is thus predicted to have good oral bioavailability. The Ghose filter is also fulfilled for all complexes (atoms restricted between 20 and 70).

The above results indicate that these compounds were found to obey Lipinski’s rule and can easily bind to receptors and were taken further for the calculation of bioactivity scores by calculating the activity score of the G-protein-coupled receptor (GPCR) ligands, ion channel modulator, nuclear receptor ligand, kinase inhibitor, protease inhibitor and enzyme inhibitor. The probable biological activity profiles for complexes **1**–**5** and the determination of drug-likeness score of compounds through the molinspiration bioactivity score prediction (GPCR ligands), kinase inhibitors, ion channel modulators, nuclear receptors) are depicted in Table S6. The larger bioactivity score has a higher probability of a specific molecule being active [78]. If the bioactivity score of a molecule is greater than 0.00 and has considerable biological activities and a score between −0.50 to 0.00, it is considered to be moderately active, and if the value is less than −0.50, it is presumed to be inactive. Drug-likeness may be defined as a complex balance of various molecular properties and structure features that determine whether a particular molecule is similar to the known drugs. These properties, mainly hydrophobicity, electronic distribution, hydrogen bonding characteristics, molecule size and flexibility, and, of course, presence of various pharmacophoric features, influence the behavior of the molecule in a living organism, including bioavailability, transport properties, affinity to proteins, reactivity, toxicity, metabolic stability and many others. The obtained drug-likeness score values (Table S6) reveal that all complexes exhibit a bioactivity score higher than −0.50, indicating moderate activity for these compounds. A higher bioactivity score for all properties is displayed by complexes **4** and **5** and also for complex **1** for GPCR and protease inhibitor properties. All the compounds pare redicted to be highly active (≥0) towards GPCR ligands. As for the ion channel modulator, among the five compounds, complexes **1**, **4**, and **5** were found to be highly active (≥0). On the contrary, complexes **2** and **3** were found to be moderately active (≤0). Furthermore, complexes **4**, and **5** were found to be highly active (≥0) towards the kinase inhibitor, whereas complexes **1***–***3** were found to be moderately active (≤0). For the nuclear receptor ligand, complexes **2**, **4**, and **5** were found to be highly active (≥0), while **1** and **3** were found to be moderately active (≤0). Finally, all complexes were found to be highly active (≥0) towards the protease inhibitor and enzyme inhibitor properties.

#### 2.9.2. PASS Biological Activity Prediction Profile

Prediction of activity spectra is based on PASS technology which can predict over 4000 kinds of biological activity, including pharmacological effects, mechanisms of action, toxic and adverse effects, interaction with metabolic enzymes and transporters, influence on gene expression, etc.

##### Acute Rat Toxicity Prediction by GUSAR (on the Basis of PASS Prediction)

The rat acute toxicity assessment is an extremely important feature in the development of new drugs. However, given the relatively high cost of such experimental studies and ethical considerations, we used the prediction of rat acute toxicities for complexes **1***–***5** by the in silico tool GUSAR (General Unrestricted Structure-Activity Relationships) via four types of administration (intraperitoneal, intravenous, oral, and subcutaneous). Predictions of LD_50_ values of rat acute toxicity, based on the structural formula of the tested compounds, are reported along with the acute rodent toxicity classification of the compounds. Acute toxicity is an important adverse effect (or death) that occurs shortly after a single dose of the substance has started. The LD_50_ value is one of the important characteristics of acute toxicity corresponding to a dose that causes 50% mortality within 24 h after administration of the substance. Acute toxicity, determined by external, oral or inhalation administration of the substance, is an important parameter for assessing overall toxicological risk, whereas acute toxicity for intra-intravenous and intravenous (IV) substance administration is an important parameter for drug development. The results of the studies are presented in Table S7. The results of the acute toxicity prediction show that complexes **1***–***5** for most cases of administration can obviously be considered to possess moderate toxicity (4-class toxicity falling in the applicability domain, in AD). Out of the applicability domain (out of AD) is documented only for complexes **3** and **4** and only when considering intraperitoneal (IP) and oral route of administration (class 3, moderate toxicity). Complexes **1** (for IP), **2** (for IV), **3** (for IP and IV), **4** (for IP, IV, and oral), and **5** (for IV) were predicted to be very toxic. It should be understood that the predicted moderate toxic effects of the complexes are just an indication that the compound under study has some structural similarity with the compounds from the training set with those effects. However, in vivo experimental procedures should determine if adverse or toxic effects arise at the same dose/concentration as the desirable pharmacotherapeutic action or in much higher doses. Furthermore, it is necessary to keep in mind that adverse and toxic effect prediction is based on clinical practice, which is sometimes observed in a few or even in a single patient.

##### SMP: Prediction of Substrate/Metabolite Specificity (Pa > Pi)

The substrate and metabolite-based specificity predictions for complexes **1***–***5** are illustrated in Table S8. The interactions with metabolic enzymes and transporters are shown with their corresponding probability levels. The CYP enzymes, particularly isoforms 1A2, 2C9, 2C19, 2D6 and 3A4, were responsible for about 90% of oxidative metabolic reactions [79]. The more CYP isoforms a given molecule inhibits, the more likely it will be involved in drug–drug interactions (DDI) with many other drugs [80]. It should be noted that for both substrate-based and metabolite-based prediction results, for no complex a probability level higher than 0.5 was documented (marginally, only complex **2** showed to be an inhibitor for CYP enzymes isoforms 2A6 and 2B6 for substrate-based prediction). Drugs that inhibit CYP2C19 and CYP2C9 enzyme activity tend to increase plasma concentrations and, in some cases, adverse effects may occur [81]. None of the complexes were found to inhibit CYP2C19 and CYP2C9 enzyme activity. Furthermore, the results revealed that all complexes have no CYP2D6 and CYP3A4 inhibitory activity, which are responsible for the metabolism of many drugs and toxic chemicals. CYP2D6 is involved in the metabolism of drugs such as antiarrhythmics, adrenoceptor antagonists and tricyclic antidepressants [82], while CYP3A4 is an enzyme responsible for the oxidation of small organic molecules (xenobiotics), such as toxins or drugs, so that they can be removed from the body, found mainly in the liver and intestine [83].

##### ROSC-Pred: Rodent Organ-Specific Carcinogenicity Prediction

As is illustrated in Table S9, no carcinogenicity was predicted for complexes **1**–**5** and for probability levels higher than 0.7 (Pa > 0.7), except for the following cases: male rats specifically for the stomach and kidney, female rats for the stomach, male mice for the stomach and kidney, female mice for the lung and stomach (complex **2**), and male and female mice for the liver (complex **3**). Complexes **1**–**5** were predicted to be out of the applicability domain for both rats and mice with a percentage of new Multilevel Neighborhoods of Atoms (MNA) descriptors of 35.5%, 56%, 44%, 56%, and 29.6%, respectively. Since there are no tested molecules with up to 25% of new MNA descriptors, it is obvious that these complexes are out of the applicability domain. The percentage of new MNA descriptors for a tested molecule is used for the assessment of the applicability domain: the higher the percentage of new MNA descriptors, the less appropriate for the model the molecule structure is.

##### Quantitative Prediction of Anti-Target Interaction Profiles for Chemical Compounds by GUSAR Software

The quantitative prediction of anti-target (off-target) interaction profiles of chemical compounds is useful for researchers to increase the efficacy of finding drug-like leads with desirable pharmacological effects but without the side effects and toxicity caused by interactions with anti-targets. From Table S10, it is deduced that the total number of anti-targets for compounds falling in the applicability domain of the model for complexes **1**–**5** are 8, 6, 8, 7, and 8, respectively. The interactions of the complexes in focus with anti-targets might be the cause of adverse or toxic effects. Activities are specified as IC_50_ (50% of the inhibitory concentration), K_i_ (inhibition constant), or K_act_ (activation constant). When included in the model SAR base, it is possible to make predictions about what concentration of the compound is required to lead to an interaction (inhibition or activation) with one of the 18 anti-target proteins included (13 receptors, 3 transporters, 2 enzymes). Among these anti-targets, the GUSAR predictions correspond to multiple side-effects: hydroxytryptamine 1B receptor antagonist,5-hydroxytryptamine 2A receptor antagonist, 5-hydroxytryptamine 2C receptor antagonist, alpha1a adrenergic receptor antagonist, alpha-1b adrenergic receptor antagonist, alpha-2A adrenergic receptor antagonist, amine oxidase [flavin-containing] A inhibitor, androgen receptor antagonist, carbonic anhydrase I activator, carbonic anhydrase 2 activator, carbonic anhydrase II inhibitor, D(1A) dopamine receptor antagonist, D3 dopamine receptor antagonist, delta-type opioid receptor antagonist, estrogen receptor antagonist, kappa-type opioid receptor antagonist, mu-type opioid receptor antagonist, sodium- and chloride-dependent GABA transporter 1 antagonist, and sodium-dependent dopamine transporter antagonist.

##### Environmental Ecotoxicity Predicted by GUSAR

Quantitative prediction of ecotoxicity for chemical compounds by GUSAR software is reported in Table S11. The QSAR models showed that complexes **1**–**3** and **5** fall in the applicability domain of the model, for all predicted activities: bioaccumulation factor, Daphnia magna, Fathead Minnow, and Tetrahymena pyriformis, whilst complex **4** is out of the applicability domain of the model.

##### CLC-Pred: In Silico Prediction of Cytotoxicity for Tumor and Non-Tumor Cell Lines

The in silico prediction of cytotoxic activity of complexes **1**–**5** on a large number of cancer cell lines, by means of Pa and Pi values, is shown in Table S12. It is deduced that for probability levels higher than 0.5, no cancer cell line was found to be sensitive to complex **1**. For Pa > 0.3, the three cancer cell lines with the higher probability were revealed to be the HT-29 colon adenocarcinoma, HepG2 hepatoblastoma, and the brain-derived oligodendroglioma Hs 683, with Pa = 0.475, 0.474, and 0.474, respectively. On the other hand, the only non-tumor cell line that was found to be sensitive to complex **1** is the embryonic lung fibroblast WI-38 VA13 with Pa = 0.395. For Pa > 0.5. Only one cancer cell line was found to be sensitive to complex **2**, the oligodendroglioma Hs 683 with Pa = 0.603. Similarly, for complex **3**, the most sensitive cancer cell line was found to be again the oligodendroglioma Hs 683 with Pa = 0.645. For complex **4**, the two sensitive cancer cell lines were the ovarian carcinoma PA-1 and the oligodendroglioma Hs 683 with Pa values 0.603 and 0.585, respectively. No non-tumor cell line was found for Pa > 0.5 to be sensitive to complexes **2**–**4**. For complex **5**, the most sensitive cancer cell line was predicted to be the non-small cell lung cancer NCI-H838 (Pa = 0.601) non-tumor cell line and the embryonic lung fibroblast WI-38 VA13 (Pa = 0.748). Complexes **2** and **5** were predicted to be among the most active complexes against the cancer cell lines. Nevertheless, it should be understood that chemical–biological interaction is rather a complicated task because of the multifaceted structure–function relationships in biological systems. It is necessary to keep in mind that it is a qualitative estimation of the compound’s activities, calculating for this structural formula the probability of belonging to the classes of “actives” and “inactives”, respectively.

##### SOMP: Prediction of Sites of Metabolism

For the prediction of SOMPs for complex **1**, sets for the five isoforms of CYP P450 that metabolize the majority of xenobiotics have been prepared: 3A4, 2C9, 2C19, 2D6 and 1A2. The reaction of glucoronidation was also included, which is catalyzed by UGT (Table S13). The SoLAs are illustrated in Table S13, in which a labeled atom is a SOM with positive ΔP values. The labeled atoms of complex **1** in SOM (ΔP+) is revealed to be 20 and 21 atoms for UGT, 22, 24, 26, 28 (ranked 1), 7, 13 (ranked 2), 3, 17 (ranked 3), 4, 18 (ranked 4), 10 (ranked 5), 1, 15 (ranked 6), 23, 25, 27, and 29 (ranked 7) for CYP3A4, 22, 24, 26, 28 (ranked 1), 7, 13 (ranked 2), 10 (ranked 3), 3, and 17 (ranked 4) for CYP2D6, 22, 24, 26, 28 (ranked 1), 20, 21 (ranked 2), 7, 13 (ranked 3), 10 (ranked 4), 23, 25, 27, and 29 (ranked 5) for CYP2C9, 22, 24, 26, 28 (ranked 1), 7, 13 (ranked 2), 10 (ranked 3), 3, 17 (ranked 4), 23, 25, 27, and 29 (ranked 5) for CYP2C19, and 22, 24, 26, 28 (ranked 1), 7, 13 (ranked 2), 4, 18 (ranked 3), 3, 10, and 17 (ranked 4) for CYP1A2. Similar predictions of sites of metabolism were identified for the rest complexes **2**–**5** (Table S13).

##### Activity Spectra Prediction

The results of PASS prediction are given as a list of biological activities, for which the difference between probabilities to be active (Pa) and to be inactive (Pi) was calculated. The results for activity spectra prediction for complexes **1**–**5** with Pa > 0.7 are reported in Table S14. The output file represents a list of predictable biological activities. It is interesting to note that in the first places of predicted activity for complexes **1**–**5** revealed to be the phobic disorders treatment with Pa = 0.892 for **1** (first place), Pa = 0.793 for **2** (second place), Pa = 0.831 for **3** (fourth place), Pa = 0.799 for **4** (first place), and Pa = 0.847 for **5** (first place). Other targets among the first ones include the testosterone 17beta-dehydrogenase (NADP^+^) inhibitor (sixth for **1**, third for **2**, eighth for **3**, fourth for **4**, and third for **5**). The antineoplastic activity was also documented for complex **5** (second place), **4** (non-Hodgkin’s lymphoma antineoplastic, second place), and **2** (fifth place). Furthermore, vascular and cardiovascular activity was shown for **1** (third place) and **2** (first place). Furthermore, inhibitory activity against 27-hydroxycholesterol 7alpha-monooxygenase predicted for complexes **1** (fourth place) and **5** (twenty-second place) with Pa > 0.7. Complex **2** exhibited also a tyrosine-protein kinase receptor FLT3 inhibitory activity (therapeutic effects of FLT3 inhibitors have been reported in acute myeloid leukemia (AML)), and complexes **3** and **5** showed an anti-glaucomic and G-protein-coupled receptor kinase inhibitor activity, respectively. Complex **5** is predicted also to have a ubiquinol-cytochrome-c reductase inhibitor activity with a possible antibiotic activity against pathogenic fungi, and Janus kinase-2 (JAK2) expression inhibitor activity. The JAKs play critical roles in several important intracellular signaling pathways, including the eponymous JAK/STAT pathway, central to the mediation of cytokine signaling. JAK2 supports breast cancer growth playing a crucial role in the coordination of cell signaling pathways and thus JAK2 expression inhibitors may play a key role in the treatment of breast cancer [84].

As complex **2** was predicted to exhibit tyrosine-protein kinase receptor FLT3 inhibitory activity and **5** was found to exhibit inhibitory activity against JAK2, we adopted molecular docking studies on these two target proteins. The binding of each complex on FLT3 and JAK2 protein crystal structures is illustrated in Figure S15. The docking procedure reveals that both complexes are bound in a binding pocket of the proteins at exactly the same place as that occupied by the co-crystallized inhibitors. Complex **5** especially is shown to be stabilized in the ATP-binding pocket of JAK2 at the same place occupied by the GMP6 inhibitor, between the Hinge region and the Glycine loop, flanked by β-sheets β1, β2, and β3 of the upper part of 3/10A helix (below the five-stranded antiparallel β-sheets (β1 to β5)) and the 3/10B helix at the bottom of the ATP-binding cavity at the boundary of the antiparallel β-sheet pairs β7, β8. The common binding contacts between **5** and GMP6 were found to be Leu L855, Val V863, Ala A880, and Val V911. Similarly, complex **2** is positioned in the binding cavity of FLT3 at the same place as the Gilteritinib inhibitor, making it common with the inhibitor contacts such as Glu E692, Asp D829, Phe F830, and Cys C694. These results suggest that complexes **2** and **5** may have therapeutic potential in FLT3-mutated AML and in diseases where the JAKs play a pivotal role.

##### DIGEP-Pred: Prediction of Drug-Induced Changes of Gene Expression Profile

The DIGEP-Pred of drug-induced changes of gene expression profile for complexes **1**–**5** is shown in Table S15. With this in silico tool, it is possible to estimate the influence of complexes **1**–**5** on gene expression based on mRNA, protein and activity related to human cancer cell lines MCF-7 (breast) and VCaP (prostate) and the combination prediction results, respectively. The output file represents a list of activities with two probabilities, Pa (probability to be active) and Pi (probability to be inactive). The more probable changes in gene expression are at the top of the list. Only the down-regulated and up-regulated target proteins predicted with a Pa > 0.5 are shown.

It is interesting to note (Table S15) that the first entry with the highest Pa values in the mRNA-based training set prediction of down-regulation genes by complex **1** is *TAGLN* (transgelin) with Pa = 0.623. The *TAGLN* gene is associated with a number of diseases such as colonic neoplasms, esophageal squamous cell carcinoma, lung neoplasms, liver neoplasms, endometriosis, lipidoses, chemical and drug-induced liver injury, necrosis, inflammation, hyperplasia, hepatomegaly, kidney diseases, fibrosis, memory disorders, neurotoxicity syndromes, heart diseases, nerve degeneration, hypertension, fetal growth retardation, cardiovascular diseases, pulmonary edema, testicular diseases, insulin resistance, and chromosome breakage. It is also associated with Peyronie Disease and Werner Syndrome. Burn wound healing and PDGFR-beta signaling pathways are among the related pathways.

Complex **1** may regulate osteosarcoma cell proliferation and invasion by down-regulating its target gene, *TAGLN*, suggesting that complex **1** may be a potential therapeutic target for the treatment of osteosarcoma. Furthermore, elevated expression of *TAGLN* was associated with advanced colorectal cancer stage and poor predicted overall survival [85]. *TAGLN* is an actin-binding protein that affects the dynamics of the actin cytoskeleton indicating its robust potential as a metastasis initiator. Since *TAGLN* is highly expressed in metastatic bladder cancer, it may represent a novel target agent that can be utilized for the clinical management of invasive and metastatic bladder cancer [86].

At protein-based prediction, complex’s **1** best down-regulated gene (Pa = 0.737) is found to be *CHEK1* (checkpoint kinase 1) associated with breast neoplasms, polyploidy, necrosis, inflammation, kidney diseases, hyperplasia, hepatomegaly, neoplasms, micronuclei, chromosome-defective, liver neoplasms, and fibrosis. Checkpoint kinase 1 inhibition was found to enhance the sensitivity of triple-negative breast cancer cells to proton irradiation via down-regulation of DNA repair gene Rad51 [87]. The second down-regulated gene was found to be *CAT* (catalase).

For MCF-7 human breast cancer cells-based predictions of complex **1**, it was identified as down-regulated only in three genes, *C3orf52* (chromosome 3 open reading frame 52), *UBE2C* (ubiquitin conjugating enzyme E2 C), and *MRPL34* (mitochondrial ribosomal protein L34). For VCAP_6h and VCAP_24h based on the prediction results of complex **1**, the highest ranked down-regulated genes were found to be *WDR11* (WD repeat domain 11) (Pa = 0.961) and *CBX1* (chromobox 1) (Pa = 0.981), respectively.

For mRNA-based predictions of complex **2** at Pa > 0.7, the highest ranked down-regulated gene with Pa = 0.843 was identified to be the *TRAF5* (TNF receptor-associated factor 5) gene, associated with numerous diseases, among which are hepatocellular carcinoma, cardiovascular diseases, atherosclerosis, liver neoplasms, precancerous conditions, neurotoxicity syndromes, and nerve degeneration. Complex **2** at protein-based prediction was found to best down-regulate (Pa = 0.586) the *PPARA* (peroxisome proliferator-activated receptor alpha) gene, associated with hepatomegaly, kidney diseases, liver neoplasms, hypertriglyceridemia, diabetes mellitus type 2, acute kidney tubular necrosis, prostatic neoplasms, hyperlipoproteinemias, Crohn disease, hypertension, inflammation, insulin resistance, dyslipidemias, cardiomegaly, and others. For MCF-7 based predictions of **2** and Pa > 0.7, the highest ranked gene with Pa = 0.793, the down-regulated mode of action was identified as *FOXM1* (forkhead box M1).

For mRNA-based predictions of complex **3** at Pa > 0.7, the highest ranked down-regulated gene (Pa = 0.739) that was found to play a role is the *TRAF5* (TNF receptor-associated factor 5) gene, such as in complex **2**. For protein-based predictions, the highest ranked down-regulated gene was identified as *IFNG* (interferon gamma) (Pa = 0.769), while for MCF-7, VCAP_6h, and VCAP_24h-based prediction results, the following genes played a role: *BBS4* (Bardet–Biedl syndrome 4), *SIN3B* (SIN3 transcription regulator family member B), and *STX10* (syntaxin 10), with Pa = 0.621, 0.995, and 0.997, respectively.

For mRNA-based predictions of complex **4** at Pa > 0.7, the highest ranked down-regulated gene (Pa = 0.903) was predicted to be *CYP2S1* (cytochrome P450 family 2 subfamily S member 1). For protein-based predictions of complex **4**, only one down-regulated gene was found at a probability level of Pa > 0.7, with Pa = 0.859: PRKCA (protein kinase C alpha). For MCF-7 based predictions of **4**, the only three down-regulated genes were *BBS4* (Bardet–Biedl syndrome 4) (Pa = 0.562), *MRPL34* (mitochondrial ribosomal protein L34) (Pa = 0.505), and *SBF1* (SET binding factor 1) (Pa = 0.501). For VCAP_6 h, and VCAP_24 h-based prediction results, the following genes seemed to play a role: *PPAN* (peter pan homolog) and *SPAG7* (sperm associated antigen 7), with Pa values of 0.991 and 0.995, respectively.

For mRNA-based predictions of complex **5** at Pa > 0.7, the highest ranked down-regulated gene (Pa = 0.846) was predicted to be the same as the mRNA-based predictions for complexes **2** and **3**, the *TRAF5* (TNF receptor-associated factor 5) gene. In protein-based prediction, only three genes were found to be down-regulated. Complex’s **5** best down-regulated gene (Pa = 0.860) was found to be *CHEK1* (checkpoint kinase 1), the same for the protein-based prediction of complex **1**, while the second and third genes were predicted to be *MDM2* (MDM2 proto-oncogene) and *PRKCA* (protein kinase C alpha) with Pa values 0.695 and 0.550, respectively. For MCF-7, VCAP_6h, and VCAP_24h based prediction results of complex **5**, the highest ranked down-regulated genes were found to be *FOXM1* (forkhead box M1), *BAG2* (BAG cochaperone 2), and *IFT52* (intraflagellar transport 52) with Pa values 0.817, 0.908, and 0.941, respectively.

## 3. Materials and Methods

### 3.1. Materials, Instrumentation and Physical Measurements

All chemicals and solvents were reagent grade and were used as purchased from commercial sources: X-saloH, Pd(CH_3_COO)_2_, CH_3_ONa, trisodium citrate, NaCl, BSA, HSA, CT DNA, EB, ABTS, K_2_S_2_O_8_, NaH_2_PO_4_, NDGA and BHT were purchased from Sigma-Aldrich Co; Trolox from J&K; warfarin, ibuprofen and DPPH were bought from TCI; L-ascorbic acid and all solvents from Chemlab.

IR spectra (400–4000 cm^−1^) were recorded on a Nicolet FT-IR 6700 spectrometer with samples prepared as KBr pellets (abbreviations used: (s) for strong and (m) for medium). UV-vis spectra were recorded as nujol mulls and in DMSO solutions at concentrations in the range 10^−4^–5 × 10^−3^ M on a Hitachi U-2001 dual beam spectrophotometer. C, H and N elemental analyses were performed on a Perkin Elmer 240B elemental microanalyzer. Molecular conductivity measurements of 1 mM DMSO solution of the complexes were carried out with a Crison Basic 30 conductometer. Fluorescence spectra were recorded in solution on a Hitachi F-7000 fluorescence spectrophotometer. Viscosity experiments were carried out using an ALPHA L Fungilab rotational viscometer equipped with an 18 mL LCP spindle and the measurements were performed at 100 rpm. ^1^H NMR spectra were recorded on an Agilent 500/54 (500 MHz for ^1^H) spectrometer using DMSO-d_6_ as the solvent (abbreviations used: (s) for singlet, (d) for doublet and (dd) for double-doublet).

DNA stock solution was prepared by dilution of CT DNA to the buffer (containing 150 mM NaCl and 15 mM trisodium citrate at pH 7.0) followed by exhaustive stirring at 4 °C for 3 days, and was kept at 4 °C for no longer than a week. The stock solution of CT DNA gave a ratio of UV absorbance at 260 and 280 nm (A_260_/A_280_) of 1.88, indicating that the DNA was sufficiently free of protein contamination [88]. The DNA concentration per nucleotide was determined by the UV absorbance at 260 nm after 1:20 dilution using ε = 6600 M^−1^cm^−1^ [89].

### 3.2. Synthesis of the Complexes

Complexes **1**–**5** were prepared according to a published procedure [22]. An acetonitrile solution of the corresponding X-saloH (1 mmol), deprotonated by CH_3_ONa (1 mmol, 54 mg), was added to an acetonitrile solution of Pd(CH_3_COO)_2_ (0.5 mmol, 112 mg) at room temperature (RT). The reaction mixture was stirred for 1 h and the reaction solution was left to stand at RT for slow evaporation; after a few days, orange-yellow single crystals of complex **1** and microcrystalline products for complexes **2**–**5** were collected.

**[Pd(4-Et_2_N-salo)_2_]·CH_3_CN, (1)·CH_3_CN:** orange single crystals, suitable for X-ray crystallography determination (130 mg, yield 50%), analyzed as [Pd(4-Et_2_N-salo)_2_]·CH_3_CN (PdC_24_H_31_N_3_O_4_) (MW = 531.95) C: 54.19, H: 5.87, N: 7.90; Found: C: 54.25, H: 5.92, N: 7.95%; IR spectrum (KBr): selected peaks (cm^−1^): 1621(s) *v*(C=O), 1348(s) *v*(C-O→Pd), 586(m) *v*(Pd-O); UV-vis: λ/nm (ε, M^−1^cm^−1^) as nujol mull: 340, 350; in DMSO: 339 (7500), 350 (1500); ^1^H NMR spectrum in DMSO-d_6_, δ(ppm): 9.56 (2H, s, H^7^), 5.89 (2H, s, H^3^), 6.26 (2H, d, H^5^), 7.26 (2H, d, H^6^), 3.37 (2H, s, CH_2_), 1.10 (3H, d, CH_3_).

**[Pd(3,5-diBr-salo)_2_], (2)**: orange-yellow microcrystalline product, (160 mg, yield: 48%) analyzed as [Pd(3,5-diBr-salo)_2_] (PdC_14_H_6_Br_4_O_4_), (MW = 664.23): C: 25.31, H:0.91; Found: C: 25.31, H: 0.90%; IR spectrum (KBr): selected peaks (cm^−1^): 1600(s) *v*(C=O), 1315(m) *v*(C-O→Pd), 472(m) *v*(Pd-O); UV-vis: λ/nm (ε, M^−1^cm^−1^) as nujol mull: 315, 425; in DMSO: 314 (4000), 426 (8000); ^1^H NMR spectrum in DMSO-d_6_, δ(ppm): 9.94 (2H, s, H^7^), 7.98 (2H, d, H^6^), 7.26 (2H, dd, H^4^).

**[Pd(3,5-diCl-salo)_2_], (3):** orange-yellow microcrystalline product, (105 mg, yield 43%) analyzed as [Pd(3,5-diCl-salo)_2_] (PdC_14_H_6_Cl_4_O_4_) (MW = 486.43) C: 34.57, H: 1.24; Found: C: 34,55, H: 1.24%; IR spectrum (KBr): selected peaks (cm^−1^): 1608(s) *v*(C=O), 1340(m) *v*(C-O→Pd), 558(m) *v*(Pd-O); UV-vis: λ/nm (ε, M^−1^cm^−1^) as nujol mull: 316, 425 in DMSO: 316 (4000), 425 (10,000); ^1^H NMR spectrum in DMSO-d_6_, δ(ppm): 9.96 (2H, s, H^7^), 7.79 (2H, d, H^6^), 7.30 (2H, dd, H^4^).

**[Pd(5-F-salo)_2_], (4):** orange-yellow microcrystalline product (80 mg, yield 41%) analyzed as [Pd(5-F-salo)_2_], (PdC_14_H_8_F_2_O_4_) (MW = 384.63) C: 43.72, H: 2.10; Found: C: 43.72, H: 2.10%; IR spectrum (KBr): selected peaks (cm^−1^): 1608(s) *v*(C=O), 1319(m) *v*(C-O→Pd), 507(m) *v*(Pd-O); UV-vis: λ/nm (ε, M^−1^cm^−1^) as nujol mull: 324, 427; in DMSO: 325 (4000), 428 (4000). ^1^H NMR spectrum in DMSO-d_6_, δ(ppm): 9.93 (2H, s, H^7^), 7.20 (2H, s, H^6^), 7.00 (2H,d, H^4^), 6.35 (2H, s, H^3^).

**[Pd(4-OMe-salo)_2_], (5):** orange-yellow microcrystalline product (92 mg, yield 45%) analyzed as [Pd(4-OMe-salo)_2_], (PdC_16_H_14_O_6_) (MW = 408.7) C: 47.02, H: 3.45; Found: C: 47.03, H: 3.44%, IR spectrum (KBr): selected peaks (cm^−1^): 1617(s) *v*(C=O), 1308(m), *v*(C-O→Pd), 593(m) *v*(Pd-O); UV-vis: λ/nm (ε, M^−1^cm^−1^) as nujol mull: 315(sh), 386 in DMSO: 315 (6000), 386 (5000). ^1^H NMR spectrum in DMSO-d_6_, δ(ppm): 9.76 (2H, s, H^7^), 8.26 (2H, s, H^6^), 7.52(2H, d, H^3^), 6.39 (2H, d, H^5^), 3.60 (6H, s, OCH_3_).

### 3.3. X-ray Crystal Structure Determination

Single crystals of complex **1** suitable for crystal structure analysis were obtained by slow evaporation of the mother liquid at RT. The instrument used was a Bruker Kappa APEX2 diffractometer equipped with a triumph monochromator using Mo Kα radiation. Unit cell dimensions were determined and refined by using the angular settings of at least 225 high-intensity reflections (>10σ(I)) in the range 10° < θ < 20°. Intensity data were recorded at RT using φ and ω-scans. All crystals presented no decay during the data collection. The frames collected for each crystal were integrated with the Bruker SAINT Software package [90], using a narrow-frame algorithm. Data were corrected for absorption using the numerical method (SADABS) based on crystal dimensions [91]. The structure was solved using the SUPERFLIP package [92] incorporated in Crystals. Data refinement (full-matrix least-squares methods on *F*^2^) and all subsequent calculations were carried out using the Crystals version 14.61_build_6236 program [93]. All non-hydrogen atoms of the complex unit were refined anisotropically. The disordered atoms of the solvent molecules were isotropically refined. Hydrogen atoms were located by difference maps at their expected positions and refined using soft constraints. By the end of the refinement, they were positioned geometrically using riding constraints to bonded atoms. Illustrations with 50% ellipsoid probability were drawn by CAMERON [94]. Crystallographic data for complex **1** are presented in Table S1.

### 3.4. Study of the Biological Profile of the Compounds

All the specific protocols and relevant equations used in the in vitro study of the biological activity (antioxidant and antibacterial activity, interaction with CT DNA and albumins) of the compounds can be found in the Supporting Information file (Sections S1–S4). A series of in silico studies were employed in order to predict the biological activity of the complexes. Molecular docking calculations were adopted on the crystal structure of *E. coli* and *S. aureus* DNA-gyrase, 5-LOX, and FLAP. Details concerning the in silico computation procedures and the computational tools employed to predict the complete biological activity profile of complexes are given in the Supporting information file (Section S5).

## 4. Conclusions

Five novel palladium(II) complexes of X-saloH were synthesized and characterized by various techniques. X-salo^−^ ligands bind bidentately to a Pd(II) ion in all complexes through their carbonyl and phenolato oxygen atoms.

Palladium complexes **1**–**5** presented a low ability to scavenge the DPPH radical. Complexes **1** and **5** presented the highest ABTS-scavenging activity almost equal to the reference compound Trolox. All complexes **1**–**5** presented moderate ability to reduce H_2_O_2_. Considering the antimicrobial activity of the compounds against two Gram-negative and two Gram-positive bacterial strains, the complexes were more active than the corresponding X-saloH with complexes **2** and **3** with the dihalogeno-salo ligands being better antimicrobial agents than the other complexes. The highest antimicrobial activity is provided by complex **2** against all tested bacterial strains (MIC = 25 μg/mL, 38 μM).

The interaction of the compounds with CT DNA takes place via intercalation leading to tight binding. The complexes can bind tightly and reversibly to both serum albumins used in Sudlow’s sites 1 and 2.

The in silico molecular docking procedure suggests that complexes **1**–**5** may play a role in the therapeutic approaches in the search for pharmacological intervention and treatment of health problems related to the oxidative stress of inflammatory diseases, respiratory diseases, atherosclerosis, diabetes, and cancer. In general, the in silico studies were in accordance with the in vitro studies and provided useful complementary insight into the elucidation of the mechanism of action of the studied complexes at the molecular level and the interpretation of their biological activity in many diseases. Additionally, a variety of computational tools were employed to predict the complete biological activity profile of complexes **1**–**5** after virtual target screening, the pharmacokinetic properties, the drug-like nature, and the possible toxicity of the compounds, distinguishing those which posed risk.

## Figures and Tables

**Table 1:** % DPPH-scavenging ability (DPPH%), % ABTS-scavenging activity (ABTS%), and H_2_O_2_-reducing activity (H_2_O_2_ %) for X-saloH and complexes **1**–**5**.

Complex	DPPH% (30 min)	DPPH% (60 min)	ABTS%	H_2_O_2_%
4-Et_2_N-saloH	2.47 ± 0.09	3.20 ± 0.08	25.84 ± 0.34	76.57 ± 0.71
3,5-diBr-saloH [13]	7.97 ± 0.53	10.22 ± 0.30	16.55 ± 0.31	76.05 ± 1.51
3,5-diCl-saloH [14]	15.68 ± 0.15	18.80 ± 0.52	84.89 ± 0.16	79.92 ± 0.34
5-F-saloH	3.96 ± 1.16	5.56 ± 1.06	19.57 ± 0.58	71.84 ± 0.95
4-OMe-saloH	4.16 ± 0.10	6.27 ± 0.25	40.83 ± 0.44	94.24 ± 0.67
[Pd(4-Et_2_N-salo)_2_], **1**	2.45 ± 0.09	4.00 ± 0.53	97.05 ± 0.48	18.19 ± 0.29
[Pd(3,5-diBr-salo)_2_], **2**	7.99 ± 0.24	14.63 ± 0.53	50.46 ± 0.91	49.21 ± 0.32
[Pd(3,5-diCl-salo)_2_], **3**	4.60 ± 0.20	2.91 ± 0.11	63.45 ± 0.38	51.26 ± 0.70
[Pd(5-F-salo)_2_], **4**	14.82 ± 0.45	13.61 ± 0.48	63.30 ± 0.53	48.31 ± 0.67
[Pd(4-OMe-salo)_2_], **5**	2.29 ± 0.36	3.00 ± 0.25	96.66 ± 0.46	58.38 ± 0.54
NDGA	87.08 ± 0.12	87.47 ± 0.12	Not tested	Not tested
BHT	61.30 ± 1.16	79.78 ± 1.12	Not tested	Not tested
Trolox	Not tested	Not tested	98.10 ± 0.48	Not tested
L-ascorbic acid	Not tested	Not tested	Not tested	60.80 ± 0.20

**Table 2:** Antimicrobial activity of X-saloH and complexes **1**–**5** expressed as MIC (in μg/mL or μM (values in parentheses)).

Compound	*S. aureus*	*B. subtilis*	*E. coli*	*X. campestris*
4-Et_2_N-saloH	>200 (>1035)	>200 (>1035)	>200 (>1035)	>200 (>1035)
3,5-diBr-saloH	25 (89)	25 (89)	50 (179)	25 (89)
3,5-diCl-saloH	50 (262)	50 (262)	50 (262)	50 (262)
5-F-saloH	200 (1427)	200 (1427)	200 (1427)	200 (1427)
4-OMe-saloH	>200 (>1314)	>200 (>1314)	>200 (>1314)	>200 (>1314)
[Pd(4-Et_2_N-salo)_2_], **1**	100 (204)	100 (204)	100 (204)	200 (407)
[Pd(3,5-diBr-salo)_2_], **2**	25 (38)	25 (38)	25 (38)	25 (38)
[Pd(3,5-diCl-salo)_2_], **3**	25 (51)	50 (103)	25 (51)	25 (51)
[Pd(5-F-salo)_2_], **4**	100 (259)	100 (259)	100 (259)	100 (259)
[Pd(4-OMe-salo)_2_], **5**	100 (245)	100 (245)	100 (245)	100 (245)

**Table 3:** Spectral features of the UV-vis spectra of X-saloH and complexes **1**–**5** upon addition of DNA. UV-band (λ_max_, in nm) (percentage of hyper-/hypo-chromism (ΔA/A_0_, %), blue-/red-shift of the λ_max_ (Δλ, in nm) and the corresponding DNA-binding constants (K_b_, M^−1^).

Compound	Band (ΔA/A_0_ ^a^ (%), Δλ ^b^ (nm))	K_b_ (M^−1^)
4-Et_2_N-saloH	349 (−1 ^a^, 0 ^b^)	5.06 (±0.14) × 10^5^
3,5-dibromo-saloH [13]	337 (<−50, elim ^c^), 427 (>+50, 0)	3.71 (±0.14) × 10^5^
3,5-dichloro-saloH [14]	335 (*>*−50, +2); 426 (*>*+50, +9)	5.36 (±0.15) × 10^5^
5-fluoro-saloH	334 (−30, +1); 421 (*>*+50, 0)	8.37 (±0.47) × 10^4^
4-OMe-saloH [23]	315 (−44, 1)	9.25 (±0.12) × 10^5^
[Pd(4-Et_2_N-salo)_2_], **1**	339 (+3, 0); 350 (+2, 0); 388 (−10, 0)	3.66 (±0.22) × 10^5^
[Pd(3,5-diBr-salo)_2_], **2**	310 (−10, 0); 426 (+20, 0)	3.18 (±0.21) × 10^5^
[Pd(3,5-diCl-salo)_2_], **3**	314 (−14, +2); 425 (+24, 0)	1.90 (±0.12) × 10^6^
[Pd(5-F-salo)_2_], **4**	308 (−2, 0); 426 (+15, −10)	2.84 (±0.14) × 10^5^
[Pd(4-OMe-salo)_2_], **5**	310 (sh) (−12.5, +3); 386 (+16, −3)	1.27 (±0.10) × 10^6^

^a^ “+” denotes hyperchromism and “−” denotes hypochromism. ^b^ “+” denotes red-shift and “−” denotes blue-shift. ^c^ “elim” denotes elimination of the band.

**Table 4:** Percentage of EB-DNA fluorescence quenching (ΔI/I_0_, %), Stern–Volmer constant (K_SV_ in M^−1^) and EB-DNA quenching constant (k_q_, M^−1^s^−1^) for X-saloH and complexes **1**–**5**.

Compound	ΔΙ/Ι_0_ (%)	K_SV_ (M^−1^)	k_q_ (M^−1^s^−1^)
4-Et_2_N-saloH	43.4	2.89 (±0.07) × 10^4^	1.25 (±0.03) × 10^12^
3,5-diBr-saloH [13]	46.6	3.95 (±0.10) × 10^4^	1.72 (±0.04) × 10^12^
3,5-diCl-saloH [14]	43.2	3.56 (±0.09) × 10^4^	1.55 (±0.04) × 10^12^
5-F-saloH	51.3	3.79 (±0.11) × 10^4^	1.73 (±0.05) × 10^12^
4-OMe-saloH [23]	62.5	5.19 (±0.17) × 10^4^	2.26 (±0.07) × 10^12^
[Pd(4-Et_2_N-salo)_2_], **1**	51.2	1.03 (±0.02) × 10^5^	4.49 (±0.09) × 10^12^
[Pd(3,5-diBr-salo)_2_], **2**	64.8	1.00 (±0.02) × 10^5^	4.35 (±0.10) × 10^12^
[Pd(3,5-diCl-salo)_2_], **3**	60.6	7.53 (±0.18) × 10^4^	3.27 (±0.08) × 10^12^
[Pd(5-F-salo)_2_], **4**	51.4	5.71 (±0.10) × 10^4^	2.48 (±0.04) × 10^12^
[Pd(4-OMe-salo)_2_], **5**	44.2	4.26 (±0.08) × 10^4^	1.85 (±0.04) × 10^12^

**Table 5:** The quenching of the SA-fluorescence (ΔΙ/Ιο, %) and the BSA- and HSA-quenching (k_q_, M^−1^ s^−1^) and binding (K, M^−1^) constants for X-saloH and complexes **1**–**5**.

Compound	ΔΙ/Ιο (%)	k_q_ (M^−1^ s^−1^)	K (M^−1^)
**BSA**			
4-Et_2_N-saloH		1.41 (±0.06) × 10^13^	4.16 (±0.23) × 10^5^
3,5-diBr-saloH [13]		3.65 (±0.25) × 10^13^	2.97 (±0.16) × 10^6^
3,5-diCl-saloH [14]		1.47 (±0.09) × 10^13^	1.64 (±0.10) × 10^6^
5-F-saloH		1.98 (±0.08) × 10^12^	4.31 (±0.31) × 10^4^
4-OMe-saloH [23]		1.16 (±0.14) × 10^13^	4.11 (±0.23) × 10^5^
[Pd(4-Et_2_N-salo)_2_], **1**	92.9	5.72 (±0.44) × 10^13^	1.08 (±0.06) × 10^5^
[Pd(3,5-diBr-salo)_2_], **2**	97.5	2.11 (±0.08) × 10^14^	1.50 (±0.06) × 10^6^
[Pd(3,5-diCl-salo)_2_], **3**	97.2	1.11 (±0.04) × 10^14^	7.82 (±0.24) × 10^5^
[Pd(5-F-salo)_2_], **4**	85.7	2.24 (±0.14) × 10^13^	8.79 (±0.13) × 10^4^
[Pd(4-OMe-salo)_2_], **5**	97.2	2.17 (±0.09) × 10^14^	9.18 (±0.27) × 10^5^
**HSA**			
4-Et_2_N-saloH		2.10 (±0.22) × 10^13^	4.03 (±0.17) × 10^5^
3,5-diBr-saloH [13]		1.72 (±0.06) × 10^13^	4.04 (±0.30) × 10^5^
3,5-diCl-saloH [14]		7.11 (±0.32) × 10^12^	6.33 (±0.14) × 10^5^
5-F-saloH		1.96 (±0.07) × 10^12^	4.65 (±0.35) × 10^4^
4-OMe-saloH		2.28 (±0.13) × 10^12^	9.67 (±0.48) × 10^4^
[Pd(4-Et_2_N-salo)_2_], **1**	91.8	3.48 (±0.14) × 10^13^	1.25 (±0.06) × 10^5^
[Pd(3,5-diBr-salo)_2_], **2**	88.8	4.22 (±0.13) × 10^13^	4.12 (±0.15) × 10^5^
[Pd(3,5-diCl-salo)_2_], **3**	90.4	5.00 (±0.12) × 10^13^	4.10 (±0.17) × 10^5^
[Pd(5-F-salo)_2_], **4**	75.7	1.82 (±0.06) × 10^13^	1.38 (±0.05) × 10^5^
[Pd(4-OMe-salo)_2_], **5**	92.2	6.69 (±0.23) × 10^13^	3.56 (±0.12) × 10^5^

**Table 6:** BSA- and HSA-binding constants of the compounds (K, in M^−1^) in the presence of the site probes warfarin and ibuprofen.

Compound	No Marker	Marker: Warfarin	Marker: Ibuprofen
**BSA**			
4-Et_2_N-saloH	4.16 (±0.23) × 10^5^	1.33 (±0.07) × 10^5^	4.86 (±0.55) × 10^4^
3,5-diBr-saloH [13]	2.97 (±0.16) × 10^6^	2.84 (±0.10) × 10^5^	7.11 (±0.29) × 10^5^
3,5-diCl-saloH [14]	1.64 (±0.10) × 10^6^	5.09 (±0.09) × 10^5^	1.35 (±0.55) × 10^5^
5-F-saloH	4.31 (±0.31) × 10^4^	3.02 (±0.31) × 10^4^	4.60 (±0.44) × 10^4^
4-OMe-saloH	4.11 (±0.23) × 10^5^	1.46 (±0.33) × 10^4^	7.90 (±0.21) × 10^4^
[Pd(4-Et_2_N-salo)_2_], **1**	1.08 (±0.06) × 10^5^	9.34 (±0.60) × 10^4^	8.94 (±0.63) × 10^4^
[Pd(3,5-diBr-salo)_2_], **2**	1.50 (±0.06) × 10^6^	9.64 (±0.70) × 10^4^	1.05 (±0.09) × 10^5^
[Pd(3,5-diCl-salo)_2_], **3**	7.82 (±0.24) × 10^5^	3.26 (±0.34) × 10^5^	2.89 (±0.10) × 10^5^
[Pd(5-F-salo)_2_], **4**	8.79 (±0.13) × 10^4^	6.05 (±0.27) × 10^4^	9.27 (±0.48) × 10^4^
[Pd(4-OMe-salo)_2_], **5**	9.18 (±0.27) × 10^5^	1.46 (±0.14) × 10^5^	6.52 (±0.46) × 10^4^
**HSA**			
4-Et_2_N-saloH	4.03 (±0.17) × 10^5^	2.28 (±0.09) × 10^5^	4.21 (±0.40) × 10^4^
3,5-diBr-saloH [13]	4.04 (±0.30) × 10^5^	1.23 (±0.05) × 10^5^	4.56 (±0.37) × 10^4^
3,5-diCl-saloH [14]	6.33 (±0.14) × 10^5^	1.52 (±0.07) × 10^5^	1.87 (±0.08) × 10^5^
5-F-saloH	4.65 (±0.35) × 10^4^	3.02 (±0.31) × 10^4^	6.22 (±0.21) × 10^4^
4-OMe-saloH	9.67 (±0.48) × 10^4^	1.66 (±0.21) × 10^4^	4.69 (±0.40) × 10^4^
[Pd(4-Et_2_N-salo)_2_], **1**	1.25 (±0.06) × 10^5^	6.89 (±0.28) × 10^4^	1.86 (±0.12) × 10^5^
[Pd(3,5-diBr-salo)_2_], **2**	4.12 (±0.15) × 10^5^	1.43 (±0.09) × 10^5^	3.23 (±0.13) × 10^5^
[Pd(3,5-diCl-salo)_2_], **3**	4.10 (±0.17) × 10^5^	2.47 (±0.09) × 10^5^	3.78 (±0.14) × 10^5^
[Pd(5-F-salo)_2_], **4**	1.38 (±0.05) × 10^5^	8.98 (±0.82) × 10^4^	1.10 (±0.08) × 10^5^
[Pd(4-OMe-salo)_2_], **5**	3.56 (±0.12) × 10^5^	1.00 (±0.18) × 10^5^	1.45 (±0.17) × 10^4^

**Table 7:** ΔG_bind_ Glide Standard Precision (SP) binding energies (in kcal/mol) of Pd(II) complexes **1**–**5** docked on CT DNA (PDB: 1BNA), *E. coli* DNA-gyrase and *S. aureus* DNA-gyrase (PDB: 1KZN and 5CDM, respectively), 5-LOX (PDB: 6N2W), and FLAP (PDB: 2Q7M). Values in bold and italic type denote lowest (among complexes) and highest binding energy, respectively.

Compound	CT DNA	*E. coli* DNA-Gyrase	*S. aureus* DNA-Gyrase	5-LOX	FLAP
[Pd(4-Et_2_N-salo)_2_], **1**	**−44.86**	*−14.12*	−38.00	**−30.87**	−33.20
[Pd(3,5-diBr-salo)_2_], **2**	−34.91	−20.56	−28.71	−21.78	−32.58
[Pd(3,5-diCl-salo)_2_], **3**	−44.21	**−24.87**	**−38.26**	−23.25	**−38.29**
[Pd(5-F-salo)_2_], **4**	*−32.92*	−24.07	*−27.92*	*−17.21*	−28.82
[Pd(4-OMe-salo)_2_], **5**	−36.83	−14.23	−35.66	−22.74	*−26.18*
EB	−40.47	-	-	-	-
Chlorobiocin (CBN)	-	−18.26	-	-	-
Moxifloxacin (MFX)	-	-	−42.18	-	-
QPT-1	-	-	−45.66	-	-
Trolox	-	-	-	−22.40	-

**Table 8:** DFT and FMOs energies and values of chemical global reactivity indices for complexes **1**–**5**.

Complex	DFT Energy (Hartrees)		E_HOMO_ (eV)	E_LUMO_ (eV)	*η* (eV)	*s* (eV^−1^)	*χ* (eV)	*μ* (eV)	*ω* (eV)
	6-31G*(d,p)/LANL2DZ Double-ζ	**ωB97X-D/cc-pVTZ Triple-ζ**							
**1**									2.94
X-ray	−1392.40893	−1383.65149	−5.09	−1.45	1.82	0.274	3.27	−3.27	
**2**									6.05
*cis*	−11260.3055		−6.11	−2.77					
*trans*	−11260.3069	−11247.40728	−6.12	−2.82	1.65	0.303	4.47	−4.47	
**3**									5.97
*cis*	−2805.56390		−6.10	−2.72					
*trans*	−2805.56547	−2796.91806	−6.10	−2.79	1.655	0.302	4.445	−4.445	
**4**									4.86
*cis*	−1165.65626		−5.75	−2.33					
*trans*	−1165.65708	−1159.02699	−5.72	−2.36	1.68	0.297	4.04	−4.04	
**5**									3.86
*cis*	−1196.25448		−5.52	−1.90					
*trans*	−1195.47476	−1189.11065	−5.57	−1.93	1.82	0.274	3.75	−3.75	

*η* = (E_LUMO_ − E_HOMO_)/2; *s* = 1/(2*η*); *χ* = −(E_HOMO_ + E_LUMO_)/2; *μ* = (E_HOMO_ + E_LUMO_)/2; *ω* = *μ*^2^/(2*η*).

**Table 9:** ZPE (zero-point energy) and vibrational (v) corrections (as either Temp. Correction (Hv) or Entropy Correction (Hv-TSv)), as well as the entropy S°, enthalpy H°, Gibbs energy G°, and heat capacity at constant volume Cv for the *trans* conformation of complexes **1**–**5** (Standard Thermodynamic quantities at 298.15 K and 1.00 atm).

Comp.	ZPE (kJ/mol/kcal/mol) ^a^	Vibrational (v) Corrections (kJ/mol)		S° (J/mol·K)	H° (au)	G° (au)	Cv (J/mol·K)
		Hv	Hv-TSv				
**1**	1242.63/296.99	1308.7656	1110.2507	665.82	−1391.91074	−1391.98635	333.41
**2**	445.12/106.39	494.7563	321.2041	582.10	−11260.1184	−11260.1845	227.80
**3**	450.30/107.62	498.7245	331.2835	561.60	−2805.37550	−2805.43927	227.03
**4**	509.41/121.75	551.4305	399.8988	508.24	−1165.44706	−1165.50478	208.31
**5**	725.91/173.49	775.1167	609.3427	556.01	−1195.95943	−1196.02257	242.02

^a^ 1 kcal/mol = 1 kJ/mol/4.184.

**Table 10:** QSAR parameters from CPK model as: CPK Area (Å^2^), CPK Volume (Å^3^), PSA (Å^2^), CPK ovality, and polarizability from computed wavefunction.

Complex	CPK Area (Å^2^)	CPK Volume (Å^3^)	PSA (Å^2^)	CPK Ovality	Polarizability
**1**	458.89	426.90	45.789	1.67	75.15
**2**	357.82	326.54	43.555	1.56	67.08
**3**	339.68	308.59	43.792	1.54	65.62
**4**	289.42	263.24	44.710	1.46	61.93
**5**	337.02	307.98	58.305	1.53	65.50

## Data Availability

Data is contained within the article and Supplementary Material.

## Supplementary Materials

The following supporting information can be downloaded at: https://www.mdpi.com/article/10.3390/ph15070886/s1. Cif and checkcif file for compound **1**. Experimental protocols—Sections: S1. Antioxidant biological assay; S2. Antimicrobial activity; S3. Interaction with CT DNA; S4. Interaction with serum albumins; S5. In silico computational methods. Figures: Figure S1. UV-vis spectra of DMSO solution of the compounds in the presence of increasing amounts of CT DNA; Figure S2. Plots for the calculation of K_b_ for the compounds; Figure S3. Plots of EB-DNA relative fluorescence emission intensity at λ_emission_ = 592 nm (%) versus *r* (*r* = [complex]/[DNA]) in the presence of complexes; Figure S4. Stern–Volmer quenching plots of EB bound to CT DNA for the compounds; Figure S5. Plots of % relative BSA/HSA-fluorescence emission intensity at λ_em_= 351 nm (%) versus *r* (*r* = [complex]/[BSA/HSA]) for the compounds; Figures S6 and S7. Stern–Volmer quenching plots of BSA/HSA**.** Figures S8–S13. Scatchard plots of BSA/HSA in the absence/presence of warfarin/ibuprofen for the compounds. Figure S14. Depiction of computed frontier molecular orbitals HOMO and LUMO, MEP map, and LIP map for complexes **1**–**5.** Figure S15. Docking pose orientation of **2** on the crystal structure of the FLT3 kinase domain bound with the FLT3 inhibitor Gilteritinib and **5** on the crystal structure of JAK2 kinase domain bound with its inhibitor CMP6**.** Tables: Table S1. Crystallographic data, data collection and refinement details for complex **1**. Tables S2–S15: Tables concerning in silico calculations. References [95,96,97,98,99,100,101,102,103,104,105,106,107,108,109,110,111,112,113,114,115,116,117,118,119,120,121,122,123,124,125,126,127,128,129,130,131,132,133,134,135,136,137,138,139,140,141,142,143,144,145,146,147,148,149,150] are cited in the Supplementary Materials. CCDC 2178454 contains the supplementary crystallographic data for this paper. These data can be obtained free of charge via www.ccdc.cam.ac.uk/conts/retrieving.html, accessed on 20 June 2022, (or from the Cambridge Crystallographic Data Centre, 12 Union Road, Cambridge CB21EZ, UK; Fax: (+44) 1223-336-033; or deposit@ccde.cam.ac.uk).

## Abbreviations

3,5-diBr-saloH = 3,5-dibromo-salicylaldehyde; 3,5-diCl-saloH = 3,5-dichloro-sali cylaldehyde; 4-Et_2_N-saloH = 4-diethylamino-salicylaldehyde; 4-OMe-saloH = 4-methoxy-salicyl aldehyde; 5-F-saloH = 5-fluoro-salicylaldehyde; AA = arachidonic acid; ABTS = 2,2′-azinobis (3-ethylbenzothiazoline-6-sulfonic acid; ADMET = Absorption, distribution, metabolism, excretion, and toxicity; *B. subtilis = Bacillus subtilis* ATCC 6633; BHT = butylated hydroxytoluene; BSA = bovine serum albumin; CBN = Chlorobiocin; CLC-Pred = Cell Line Cytotoxicity Predictor; CT = calf-thymus; DDI = drug–drug interactions; DIGEP-Pred = Drug-Induced Gene Expression Profiles Prediction; DPPH = 1,1-diphenyl-picrylhydrazyl; *E. coli = Escherichia coli* NCTC 29,212; EB = ethidium bromide, 3,8-diamino-5-ethyl-6-phenyl-phenanthridinium bromide; FLAP = 5-lipoxygenase-activating protein; FMO = frontier molecular orbital; GPCR = G-protein-coupled receptor; GUSAR = General Unrestricted Structure-Activity Relationships; Hb = hydrogen bond; HOMO = highest occupied molecular orbital; Hph = hydrophobic; HSA = human serum albumin; IP = intraperitoneal; IV = intravenous; JAK = Janus kinase; K = SA-binding constant; K_act_ = activation constant; K_b_ = DNA-binding constant; K_i_ = inhibition constant; k_q_ = quenching constant; K_SV_ = Stern–Volmer constant; LD = lethal dose; LIP = local ionization potential; LOX = Lipoxygenase; LT = Leukotriene; LUMO = lowest unoccupied molecular orbital; MEP = electrostatic potential; MFX = Moxifloxacin; MIC *=* minimum inhibitory concentration; MNA = Multilevel Neighborhoods of Atoms; NDGA = nordihydroguaiaretic acid; P = polar; Pa = probability “to be active”; PASS = Prediction of activity spectra for substances; PDB = protein data bank; Pi = probability “to be inactive”; *r =* [compound]/[DNA or SA] ratio; PSA = polar surface area; Ro5 = Rule of Five; ROSC-Pred = Rodent Organ-Specific Carcinogenicity Prediction; RT = room temperature; *s =* softness; *S. aureus* = *Staphylococcus aureus* ATCC 6538; SA = serum albumin; saloH = salicylaldehyde; SMP = Substrate/Metabolite specificity Prediction; SoLAs = structures with one labeled atom; SOMP = sites of metabolism prediction; *TAGLN* = transgelin; TPSA = Topological Polar Surface Area; Trolox = 6-hydroxyl-2,5,7,8-tetramethylchromane-2-carboxylic acid; *X. campestris = Xanthomonas campestris* ATCC 1395; X-saloH = substituted salicylaldehyde; ZPE = zero-point energy; *η* = hardness; *μ =* electronic chemical potential; *χ =* electronegativity; *ω* *=* electrophilicity index.
