# Supplementary material for: Palladium(II) Complexes of Substituted Salicylaldehydes: Synthesis, Characterization and Investigation of Their Biological Profile
Source: Pharmaceuticals (Basel). 2022 Jul 18;15(7):886. doi: 10.3390/ph15070886 (PMC9323974; doi:10.3390/ph15070886)

checkCIF/PLATON report

Structure factors have been supplied for datablock(s) I

THIS REPORT IS FOR GUIDANCE ONLY. IF USED AS PART OF A REVIEW PROCEDURE FOR PUBLICATION, IT SHOULD NOT REPLACE THE EXPERTISE OF AN EXPERIENCED CRYSTALLOGRAPHIC REFEREE.

No syntax errors found. CIF dictionary Interpreting this report

Datablock: I

Bond precision: C-C = 0.0051 A Wavelength=0.71073

Cell:	a=6.4774(6)	b=8.7509(8)	c=21.197(2)
	alpha=90	beta=98.350(3)	gamma=90
Temperature:	295 K		

	Calculated	Reported
Volume	1188.78(19)	1188.76(19)
Space group	P 21/n	P 1 21/n 1
Hall group	-P 2yn	?
Moiety formula	2(C22 H28 N2 O4 Pd), C4 H6 N2	C24 H31 N3 O4 Pd1
Sum formula	C48 H62 N6 O8 Pd2	C24 H31 N3 O4 Pd1
Mr	1063.84	531.93
Dx,g cm-3	1.486	1.486
Z	1	2
Mu (mm-1)	0.815	0.815
F000	548.0	548.0
F000'	546.21	
h,k,lmax	7,10,25	7,10,25
Nref	2272	2246
Tmin,Tmax	0.864,0.960	0.880,0.960
Tmin'	0.864	

Correction method= # Reported T Limits: Tmin=0.880 Tmax=0.960
AbsCorr = NUMERICAL

Data completeness= 0.989 Theta(max)= 25.777

R(reflections)= 0.0345(1960) wR2(reflections)= 0.0726(1960)

S = 1.000 Npar= 145

The following ALERTS were generated. Each ALERT has the format
test-name_ALERT_alert-type_alert-level.
Click on the hyperlinks for more details of the test.

Alert level C

```
PLAT911_ALERT_3_C Missing FCF Refl Between Thmin & STh/L=    0.600         24 Report
```

Alert level G

```
PLAT002_ALERT_2_G Number of Distance or Angle Restraints on AtSite          3 Note
PLAT003_ALERT_2_G Number of Uiso or Uij Restrained non-H Atoms ...          3 Report
PLAT042_ALERT_1_G Calc. and Reported Moiety Formula Strings Differ     Please Check
PLAT045_ALERT_1_G Calculated and Reported Z Differ by a Factor ...       0.50 Check
PLAT300_ALERT_4_G Atom Site Occupancy of N2         Constrained at        0.5 Check
PLAT300_ALERT_4_G Atom Site Occupancy of C12        Constrained at        0.5 Check
PLAT300_ALERT_4_G Atom Site Occupancy of C13        Constrained at        0.5 Check
PLAT300_ALERT_4_G Atom Site Occupancy of H131       Constrained at        0.5 Check
PLAT300_ALERT_4_G Atom Site Occupancy of H132       Constrained at        0.5 Check
PLAT300_ALERT_4_G Atom Site Occupancy of H133       Constrained at        0.5 Check
PLAT302_ALERT_4_G Anion/Solvent/Minor-Residue Disorder (Resd  2  )       100% Note
PLAT413_ALERT_2_G Short Inter XH3 .. XHn     H11      ..H131     .       1.94 Ang.
                                                   1+x,y,z  =      1_655 Check
PLAT413_ALERT_2_G Short Inter XH3 .. XHn     H112     ..H133     .       2.08 Ang.
                                        1/2-x,-1/2+y,1/2-z  =      3_545 Check
PLAT769_ALERT_4_G CIF Embedded explicitly supplied scattering data     Please Note
PLAT794_ALERT_5_G Tentative Bond Valency for Pd1       (II)      .       2.40 Info
PLAT808_ALERT_5_G No Parseable SHELXL Style Weighting Scheme Found     Please Check
PLAT860_ALERT_3_G Number of Least-Squares Restraints ............          4 Note
PLAT882_ALERT_1_G No Datum for _diffrn_reflns_av_unetI/netI ......     Please Do !
PLAT912_ALERT_4_G Missing # of FCF Reflections Above STh/L=  0.600          8 Note
PLAT913_ALERT_3_G Missing # of Very Strong Reflections in FCF ....          3 Note
PLAT929_ALERT_5_G No Weight Pars,Obs and Calc R1,wR2,S not Checked          ! Info
PLAT960_ALERT_3_G Number of Intensities with I  <   - 2*sig(I) ...          2 Check
```

0 **ALERT level A** = Most likely a serious problem - resolve or explain
0 **ALERT level B** = A potentially serious problem, consider carefully
1 **ALERT level C** = Check. Ensure it is not caused by an omission or oversight
22 **ALERT level G** = General information/check it is not something unexpected

3 ALERT type 1 CIF construction/syntax error, inconsistent or missing data
4 ALERT type 2 Indicator that the structure model may be wrong or deficient
4 ALERT type 3 Indicator that the structure quality may be low
9 ALERT type 4 Improvement, methodology, query or suggestion
3 ALERT type 5 Informative message, check

It is advisable to attempt to resolve as many as possible of the alerts in all categories. Often the minor alerts point to easily fixed oversights, errors and omissions in your CIF or refinement strategy, so attention to these fine details can be worthwhile. In order to resolve some of the more serious problems it may be necessary to carry out additional measurements or structure refinements. However, the purpose of your study may justify the reported deviations and the more serious of these should normally be commented upon in the discussion or experimental section of a paper or in the "special_details" fields of the CIF. checkCIF was carefully designed to identify outliers and unusual parameters, but every test has its limitations and alerts that are not important in a particular case may appear. Conversely, the absence of alerts does not guarantee there are no aspects of the results needing attention. It is up to the individual to critically assess their own results and, if necessary, seek expert advice.

Publication of your CIF in IUCr journals

A basic structural check has been run on your CIF. These basic checks will be run on all CIFs submitted for publication in IUCr journals (*Acta Crystallographica*, *Journal of Applied Crystallography*, *Journal of Synchrotron Radiation*); however, if you intend to submit to *Acta Crystallographica Section C* or *E* or *IUCrData*, you should make sure that full publication checks are run on the final version of your CIF prior to submission.

Publication of your CIF in other journals

Please refer to the *Notes for Authors* of the relevant journal for any special instructions relating to CIF submission.

PLATON version of 13/07/2021; check.def file version of 13/07/2021

Datablock I - ellipsoid plot

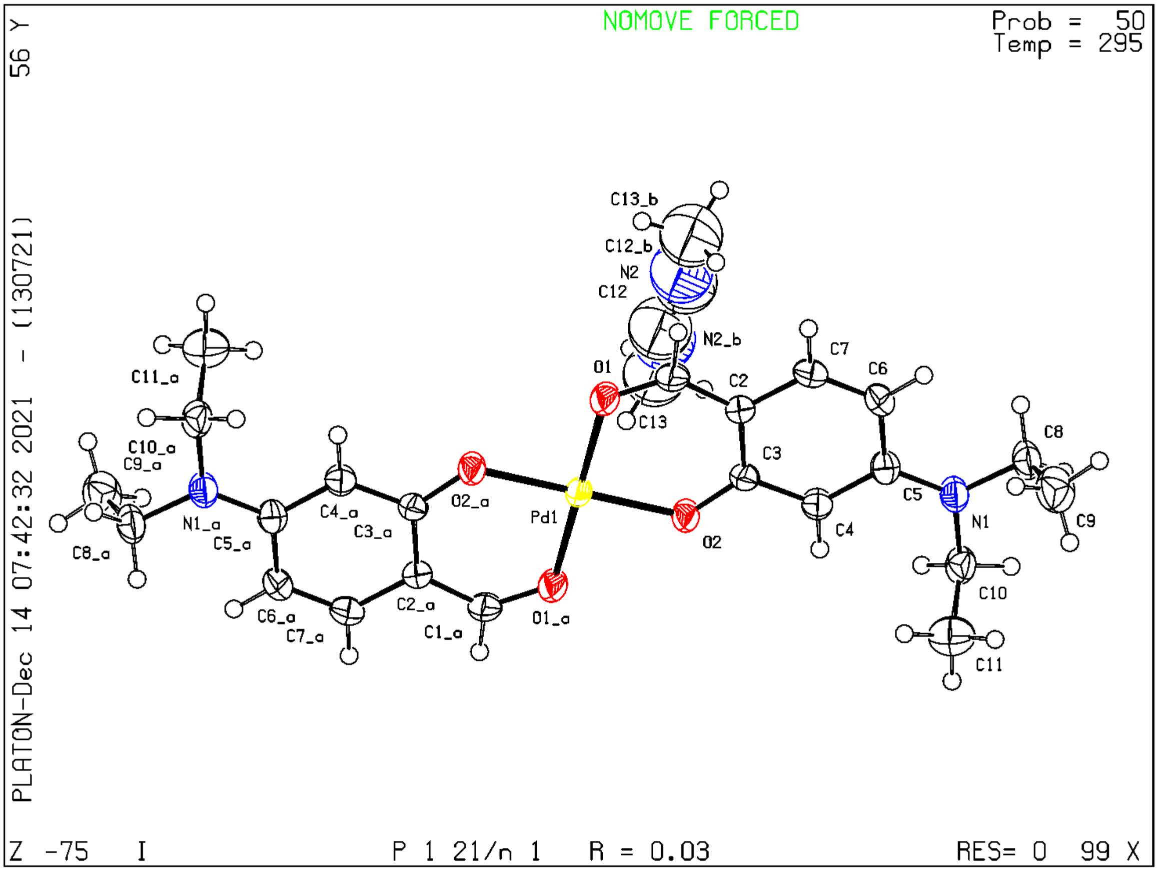

Supplement: Supplementary file 1 [file pharmaceuticals-15-00886-s001.zip › checkcif of 1.pdf]
