# Supplementary material for: Palladium(II) Complexes of Substituted Salicylaldehydes: Synthesis, Characterization and Investigation of Their Biological Profile
Source: Pharmaceuticals (Basel). 2022 Jul 18;15(7):886. doi: 10.3390/ph15070886 (PMC9323974; doi:10.3390/ph15070886)
Supplement: Supplementary file 1 [file pharmaceuticals-15-00886-s001.zip › pharmaceuticals-1806791 - REVISED ESI.pdf]

Palladium(II) complexes of substituted salicylaldehydes: Synthesis, characterization and investigation of their biological profile

Ariadni Zianna,[a,*] George Geromichalos,[a,*] Augusta–Maria Fiotaki,[b] Antonios G. Hatzidimitriou,[a] Stavros Kalogiannis,[b] George Psomas,[a,*]

[a] *Laboratory of Inorganic Chemistry, Department of Chemistry, Aristotle University of Thessaloniki, Thessaloniki GR–54124, GREECE.*

[b] *Department of Nutritional Sciences and Dietetics, International Hellenic University, Sindos, Thessaloniki, GREECE.*

Supplementary information

* Corresponding authors' e–mails:

ariadnezianna@gmail.com (A. Zianna); geromchem@yahoo.gr (G. Geromichalos); gepsomas@chem.auth.gr (G. Psomas)

**Content**	**Page**
S1 Antioxidant biological assay	5
S1.1 Determination of the reducing activity of the stable radical DPPH	5
S1.2 Assay of radical cation ABTS–scavenging activity	5
S1.3 Reduction of hydrogen peroxide	5
S2 Antibacterial activity	7
S3 Interaction with CT DNA	8
S3.1 Binding study with CT DNA by UV–vis spectroscopy	8
S3.2 CT DNA–binding studies by viscosity measurements	8
S3.3 EB–displacement studies	8
S4 Interaction with serum albumins	10
S4.1 Study of the affinity for serum albumins	10
S4.2 Competitive SA–fluorescence studies with warfarin and ibuprofen	11
S5. *In silico* computational methods	12
S5.1 Molecular modeling and docking calculations	12
S5.2 Molecular pharmacokinetic properties, drug-likeness, target proteins and toxicity prediction methods	14
S5.2.1 Calculation of molecular properties and prediction of drug-likeness	14
S5.2.1.1 Molecular properties prediction	14
S5.2.1.2 Drug–likeness	15
S5.2.1.3 Bioactivity score	17
S5.2.2 Prediction of toxicity	17
S5.2.2.1 Acute rat toxicity prediction by GUSAR (on the Basis of PASS Prediction)	18
S5.2.2.2 Substrate/Metabolite specificity Prediction (SMP)	19
S5.2.2.3 Sites of metabolism prediction (SOMP)	19
S5.2.2.4 ROSC–Pred: Rodent Organ–Specific Carcinogenicity Prediction	20
S5.2.2.5 Quantitative prediction of anti-target interaction profiles for chemical compounds by GUSAR software	20
S5.2.2.6 Environmental ecotoxicity predicted by GUSAR	22
S5.2.2.7 CLC–Pred: in silico prediction of cytotoxicity for tumor and non-tumor cell lines	22
S5.2.2.8 Prediction of activity spectra	22
S5.2.2.9 Drug–induced gene expression profiles prediction (DIGEP–Pred)	23
S6 References	25
Figure S1**.** UV-vis spectra of DMSO solution of (A) **1** (10^{-4} M), **(B) 3** (10^{-4} M), (C) **4** (10^{-4} M), (D) 4–Et_2N–saloH (10^{-5} M) and (E) 5–F–saloH (10^{-4} M) in the presence of increasing amounts of CT DNA. The arrows show the changes upon increasing amounts of CT DNA.	30
Figure S2. Plot of $\frac{[DNA]}{(\varepsilon_A - \varepsilon_f)}$ *versus* [DNA] for (A)-(G) 4–Et_2N–saloH, 5–F–saloH and complexes **1–5**, respectively**.**	31
Figure S3. Plot of EB–DNA relative fluorescence emission intensity at $\lambda_{emission}$ = 592 nm (%) *versus r* (*r* = [complex]/[DNA]) in the presence of complexes **1–5** (up to 48.8 % of the initial EB–DNA fluorescence emission intensity for **1**, 35.2 % for **2**, 39.4 % for **3**, 48.6 % for **4** and 55.8% for **5**).	32

Figure S4. Stern–Volmer quenching plot of EB bound to CT DNA for (A)-(G) 4–Et_2N–saloH, 5–F–saloH and complexes **1–5**, respectively**.**	33
Figure S5. (A) Plot of % relative BSA–fluorescence emission intensity at λ_{em}= 351 nm (%) vs *r* (*r* = [complex]/[BSA]) for complexes **1–5** (up to 7.2 % of the initial BSA fluorescence for **1**, 2.5 % for **2**, 2.8% for **3**, 14.3% for **4** and 2.8% for **5**) in buffer solution (150 mM NaCl and 15 mM trisodium citrate at pH 7.0). (B) Plot of% relative HSA–fluorescence emission intensity at λ_{em}= 342 nm (%) vs *r* (*r* = [complex]/[HSA]) for complexes **1–5** (up to 8.2% of the initial HSA fluorescence for **1**, 11.2% for **2**, 9.6% for **3**, 24.3% for **4** and 7.8 % for **5**) in buffer solution (150 mM NaCl and 15 mM trisodium citrate at pH 7.0).	34
Figure S6. Stern–Volmer quenching plot of BSA for (A)-(G) 4–Et_2N–saloH, 5–F–saloH and complexes **1–5**, respectively**.**	35
Figure S7. Stern–Volmer quenching plot of HSA for (A)-(H) 4–Et_2N–saloH, 5–F–saloH, 4–MeO–saloH and complexes **1–5**, respectively.	36
Figure S8. Scatchard plot of BSA for (A)-(G) 4–Et_2N–saloH, 5–F–saloH and complexes **1–5**, respectively**.**	37
Figure S9. Scatchard plots of HSA for (A)-(H) 4–Et_2N–saloH, 5–F–saloH, 4–MeO–saloH and complexes **1–5**, respectively.	38
Figure S10. Scatchard plots of BSA–ibuprofen for (A)-(H) 4–Et_2N–saloH, 5–F–saloH, 4–MeO–saloH and complexes **1–5**, respectively.	39
Figure S11. Scatchard plot of BSA–warfarin for (A)-(H) 4–Et_2N–saloH, 5–F–saloH, 4–MeO–saloH and complexes **1–5**, respectively.	40
Figure S12. Scatchard plots of HSA–ibuprofen for (A)-(H) 4–Et_2N–saloH, 5–F–saloH, 4–MeO–saloH and complexes **1–5**, respectively.	41
Figure S13. Scatchard plots of HSA–warfarin for (A)-(H) 4–Et_2N–saloH, 5–F–saloH, 4–MeO–saloH and complexes **1–5**, respectively.	42
Figure S14. Depiction of computed frontier molecular orbitals HOMO and LUMO (solid surface) (left panel), MEP map (center panel, transparent surface), and LIP map (right panel, transparent surface) for complexes **1**–**5**.	43
Figure S15. Docking pose orientation of **2** on the crystal structure of the FLT3 kinase domain (PDB: 6jqr) bound with the FLT3 inhibitor Gilteritinib and **5** on the crystal structure of JAK2 kinase domain (PDB: 2b7a) bound with its inhibitor CMP6.	44
Table S1. Crystallographic data, data collection and refinement details for complex **1.**	45
Table S2. Binding interactions of complex **1** with CT DNA (Atom numbering and bond lengths are derived from PyMol software).	46
Table S3. Binding interactions of lowest energy binding pose of complex **3** with *E. coli* and *S. aureus* DNA-gyrases (PDB accession numbers: 1KZN, and 5CDM, respectively). (Atom numbering and bond lengths are derived from PyMol software) (type of interaction: *π*–*π*: pi–pi sandwich, *π*–alkyl: pi–alkyl hydrophobic, *π*–cation and *π*–anion: pi–charged electrostatic interactions, *π*–polar: pi–polar, Hb: hydrogen bond, Hph: hydrophobic, P: polar, Halogen bond) (all binding contact residues are common with that of CBN).	47
Table S4. Binding interactions of lowest energy binding pose of complexes **1** with 5–LOX bound with NDGA (PDB accession number 6N2W), and **3** with FLAP protein bound with MK-591 (PDB ID: 2Q7M). (Atom numbering and bond lengths are derived from PyMol software) (type of interaction: Hb: hydrogen bond, Hph: hydrophobic, P: polar, *π*–alkyl: pi–alkyl hydrophobic, *π*–cation: pi–charged electrostatic interactions, *π*–polar: pi–polar)	48

(binding contact residues denoted in asterisk are common with that of trolox).	
Table S5. Calculated molecular physicochemical pharmacokinetics properties of complexes **1–5** related to Lipinski's Rule of Five with the aid of Molinspiration property engine v2018.10.	49
Table S6. Determination of drug–likeness score of complexes **1–5** through molinspiration bioactivity score v2018.03.	50
Table S7. Rat acute toxicity predicted by GUSAR (on the Basis of PASS Prediction) for complexes **1–5**.	51
Table S8. Substrate/Metabolite specificity Prediction (SMP) for complexes **1–5**.	52
Table S9. ROSC–Pred: Rodent Organ–Specific Carcinogenicity Prediction for complexes **1–5**.	56
Table S10. Quantitative prediction of anti-target interaction profiles for complexes **1–5**.	59
Table S11. Environmental ecotoxicity prediction for complexes 1–5 by GUSAR.	64
Table S12. CLC–Pred: in silico prediction of cytotoxicity for tumor and non-tumor cell lines (results for Pa > 0.3, Pa > 0.5, and Pa > Pi).	66
Table S13. Sites of metabolism prediction (SOMP) for complexes **1–5** by GUSAR.	75
Table S14. Prediction of activity spectra for complexes **1–5**, for Pa > 0.7 and Pa > 0.0.	105
Table S15. Drug–induced gene expression profiles prediction (DIGEP–Pred). mRNA based, protein based, MCF-7 based, VCaP 6h based, VCaP 24h based, and combination prediction results for complexes **1–5**. The name of each gene can be correlated with diseases, side-effects and biological pathways in Comparative Toxicogenomics Database (CTD), Mount Desert Island Biological Laboratory, Salisbury Cove, Maine (http://ctdbase.org/).	293

S1 Antioxidant biological assay

The antioxidant activity of the compounds was evaluated *via* their ability to scavenge *in vitro* free radicals such as DPPH and ABTS and to reduce H_2O_2. All the experiments were carried out at least in triplicate and the standard deviation of absorbance was less than 10% of the mean.

S1.1 Determination of the reducing activity of the stable radical DPPH

To an ethanolic solution of DPPH (0.1 mM) an equal volume solution of the compounds (0.1 mM) in ethanol was added. Absolute ethanol was also used as control solution. The absorbance at 517 nm was recorded at room temperature after 30 and 60 min, in order to examine the possible existence of a potential time–dependence of the DPPH radical scavenging activity [S1]. The DPPH–scavenging activity of the compounds was expressed as the percentage reduction of the absorbance values of the initial DPPH solution (DPPH%). NDGA and BHT were used as reference compounds.

S1.2 Assay of radical cation ABTS–scavenging activity

The ABTS assay was performed to determine the activity of the compounds to scavenge the radical cation ABTS. Initially, a water solution of ABTS was prepared (2 mM). ABTS radical cation ($ABTS^{+\cdot}$) was produced by the reaction of ABTS stock solution with potassium persulfate (0.17 mM) and the mixture was stored in the dark at room temperature for 12–16 h before its use. The ABTS was oxidized incompletely because the stoichiometric reaction ratio of ABTS and potassium persulfate is 1:0.5. The absorbance became maximal and stable only after more than 6 h of reaction although the oxidation of the ABTS started immediately. The radical was stable in this form for more than 2 days when allowed to stand in the dark at room temperature. Afterwards, the $ABTS^{+\cdot}$ solution was diluted in ethanol to an absorbance of 0.70 at 734 nm and 10 μL of diluted compounds or standards (0.1 mM) in DMSO were added. The absorbance was recorded out exactly 1 min after initial mixing [S1]. The ABTS radical scavenging activity was expressed as the percentage inhibition of the absorbance of the initial ABTS solution (ABTS%). Trolox was used as an appropriate standard.

S1.3 Reduction of hydrogen peroxide

The ability of the compounds to reduce hydrogen peroxide (H_2O_2) was estimated according to the method described in the literature [S2]. The reaction mixture contained 20 μL of each of the tested compounds (0.1 mM) and 5 μL H_2O_2 solution (40 mM) in phosphate buffer (50 mM, pH 7.4). The absorbance was measured at 230 nm after 10 min. The antioxidant activity (reduction of

hydrogen peroxide) of the compounds was expressed as the percentage decrease of the initial H_2O_2 solution (H_2O_2%). Ascorbic acid (or vitamin C) was used as a standard.

S2 Antibacterial activity

The antimicrobial activity of the compounds was evaluated by determining their respective MIC values towards two Gram–(–) (*Escherichia coli* NCTC 29212 (*E. coli*) and *Xanthomonas campestris* ATCC 1395 (*X. campestris*)) and two Gram–(+) (*Staphylococcus aureus* ATCC 6538 (*S. aureus*) and *Bacillus subtilis* ATCC 6633 (*B. subtilis*)) bacterial species. Cultures of these microbial strains were grown on a rich selective agar medium and stored at 4°C. The selective media used were Nutrient Agar or Broth for *B. subtilis* and *S. aureus*, Yeast Mold Agar or Broth for *X. campestris* and Luria Agar or Broth for *E. coli*. Cells picked from the surface of the stored cultures were used to initiate liquid pre–cultures of the same selective medium at an initial turbidity of roughly 1 McFarland unit. Pre–cultures were incubated for 24 h in a rotary shaking incubator and subsequently they were used to inoculate the test cultures used for the determination of MIC at an initial turbidity of 0.5 McFarland units. The test cultures consisted of Mueller–Hinton broth (Deben Diagnostics Ltd) containing different concentrations of the compounds.

Different concentrations were achieved as follows: the compounds were freshly dissolved in DMSO to a concentration of 1 mg mL^{-1} and they were diluted with DMSO, using the method of progressive double dilution. Therefore, working solutions with decreasing concentrations of the compounds under investigation were achieved. The working solutions were subsequently diluted to the final desired concentration by addition to the growth medium at a proportion of 2:98. MIC values were determined as the lowest concentrations of the tested compounds that inhibited visible growth of each respective organism after a 24 h incubation [S3]. Bacterial growth was determined by measuring the turbidity of appropriately diluted cultures at 600 nm with reference to equally diluted sterile growth medium and the inhibition achieved was calculated by comparing the turbidity of each culture to the average of the turbidity of three non–inhibited cultures. All test cultures were grown in triplicates and for the determination of MIC, growth had to be inhibited in at least two cultures of the triplicate. Incubation temperature at all stages was 37°C except for *X. campestris* that was cultivated at 28°C [S4].

S3 Interaction with CT DNA

The interaction of X–saloH and complexes **1–5** with CT–DNA was investigated by UV–vis spectroscopy, viscosity measurements and fluorescence emission spectroscopy studies.

S3.1 Binding study with CT DNA by UV–vis spectroscopy

UV–vis spectroscopy was used for the evaluation of the interaction of the compounds with CT DNA, and specifically the possible binding modes of the compounds to CT DNA. Control experiments with DMSO were performed and no changes in the spectra of CT DNA were observed.

In order to determine the binding mode, the UV–vis spectra of the compounds were recorded for a constant concentration (10^{-5}–10^{-4} M) at the corresponding λ_{max} with increasing concentrations of CT DNA for diverse *r* (*r* = [complex]/[DNA]) values. Effective use of the changes in the absorbance of the UV–vis spectra was made and the DNA–binding constants (K_b, M^{-1}) of the compounds were calculated by the Wolfe–Shimer equation (eq. S1) [S5] and the plots [DNA]/(ε_A–ε_f) *versus* [DNA]:

$$\frac{[DNA]}{(\varepsilon_A - \varepsilon_f)} = \frac{[DNA]}{(\varepsilon_b - \varepsilon_f)} + \frac{1}{K_b(\varepsilon_b - \varepsilon_f)} \qquad \text{(eq. S1)}$$

where [DNA] = the concentration of DNA in base pairs, ε_f= the extinction coefficient for the free compound at the corresponding λ_{max}, ε_A = A_{obsd}/[compound] and ε_b= the extinction coefficient for the compound in the fully bound form. K_b is given by the ratio of slope to the y intercept in plots [DNA]/(ε_A–ε_f) *versus* [DNA].

S3.2 CT DNA–binding studies by viscosity measurements

The viscosity of DNA (0.1 mM) in buffer solution was measured in the absence and presence of increasing amounts of the compounds. The experiments were executed at room temperature and the measurements are devised in a plot $(\eta/\eta_0)^{1/3}$ *versus* r, where η = the viscosity of DNA in the presence of the compound, and η_0 = the viscosity of neat DNA in buffer solution.

S3.3 EB–displacement studies

In order to determine and confirm the DNA–binding mode of the compounds, a competitive study with EB as an intercalating marker is performed by fluorescence emission spectroscopy. Therefore, the EB–displacing ability of the compounds from its EB–DNA conjugate was examined.

The DNA–EB adduct was prepared by addition of 20 μM EB and 26 μM CT DNA in buffer solution (150 mM NaCl and 15 mM trisodium citrate at pH 7.0). The potential intercalation of the compounds between the DNA–bases was studied by the addition of a certain amount of the

compound solution into the EB–DNA adduct solution. The influence of the compounds on the EB–DNA solution was monitored through the changes of the fluorescence emission spectra at excitation wavelength (λ_{ex}) at 540 nm [S6]. The tested compounds do not show any significant fluorescence at room temperature in solution or in the presence of DNA, under the same experimental conditions (λ_{ex} = 540 nm). Bearing that in mind, the observed quenching of the EB–DNA solution is evidently associated to the displacement of EB from its EB–DNA adduct.

The quenching efficiency (K_{SV}) for each compound was assessed according to the Stern–Volmer equation (eq. S2) [S6]:

$$\frac{Io}{I} = 1 + k_q \tau_0 [Q] = 1 + K_{SV}[Q] \qquad \text{(eq. S2)}$$

where I_0 and I = the fluorescence emission intensities of EB–DNA in the absence and presence of the quencher, respectively, [Q] = the concentration of the quencher (i.e. compounds). K_{SV} is obtained from the Stern–Volmer plots by the slope of the diagram I_0/I *versus* [Q]. Taking τ_0 = 23 ns as the fluorescence lifetime of the EB–DNA system [S7], the EB–DNA quenching constants (k_q, in $M^{-1}s^{-1}$) of the compounds can be determined according to equation S3:

$$K_{SV} = k_q \tau_o \qquad \text{(eq. S3)}$$

S4 Interaction with serum albumins

S4.1 Study of the affinity for serum albumins

The albumin–binding study for the compounds was carried out by fluorescence emission quenching experiments using BSA (3 μM) or HSA (3 μM) in buffer solution (15 mM trisodium citrate and 150 mM NaCl at pH 7.0). The tested compounds were used as quenchers with gradually increasing concentrations to monitor the quenching of the emission intensity of tryptophan residues of BSA at 345 nm or HSA at 340 nm [S6]. The fluorescence emission spectra were recorded between 300–500 nm with excitation wavelength of 295 nm. All the experiments were conducted at room temperature. The fluorescence spectra of the compounds were recorded under the same experimental conditions and presented a low–intensity emission band in the region 395–415 nm. Consequently, the SA fluorescence emission spectra were modified properly, by subtracting the spectra of the compounds, and quantitative studies followed.

The extent of the inner–filter effect can be roughly estimated with the following formula:

$$I_{corr} = I_{meas} \times 10^{\frac{\varepsilon(\lambda_{exc})cd}{2}} \times 10^{\frac{\varepsilon(\lambda_{em})cd}{2}} \qquad \text{(eq. S4)}$$

where I_{corr}= corrected intensity, I_{meas}= the measured intensity, c = the concentration of the quencher, d = the cuvette length (1 cm), $\varepsilon(\lambda_{exc})$ and $\varepsilon(\lambda_{em})$ = the ε of the quencher at the excitation and the emission wavelength, respectively, as calculated from the UV–vis spectra of the complexes [S8].

The interaction of the quencher (i.e. compounds) with serum albumins [S6] was studied through the Stern–Volmer and Scatchard equations [S6] and corresponding graphs. The values of the respective Stern–Volmer constant K_{SV} (M^{-1}), the quenching constant k_q ($M^{-1}s^{-1}$), the SA–binding constant K (M^{-1}) and the number of binding sites per albumin (n) were calculated.

According to Stern–Volmer quenching equation [S6] (eq. S2), where I_0 = the initial tryptophan fluorescence intensity of SA, I = the tryptophan fluorescence intensity of SA after the addition of the quencher, k_q = the quenching rate constants of SA, K_{SV} = the dynamic quenching constant, τ_0 = the average lifetime of SA without the quencher, [Q] = the concentration of the quencher), the Stern–Volmer constant (K_{SV}, M^{-1}) can be obtained by the slope of the diagram I_0/I *versus* [Q]. Taking $\tau_0 = 10^{-8}$ s as fluorescence lifetime of tryptophan in SA [S2], the quenching constant (k_q, $M^{-1}s^{-1}$) is calculated from equation S3.

From the Scatchard equation (eq. S5) [S6]:

$$\frac{\Delta I / I_0}{[Q]} = nK - K\frac{\Delta I}{I_0} \qquad \text{(eq. S5)}$$

where n = the number of binding sites per albumin and K = the SA–binding constant. The K constant (M^{-1}) is calculated from the slope in plots ($\Delta I/I_0$)/[Q] *versus* ($\Delta I/I_0$) and n is given by the ratio of y intercept to the slope [S9].

S4.2 Competitive SA–fluorescence studies with warfarin and ibuprofen

The competitive studies with warfarin or ibuprofen (site probes) [S10] were performed by tryptophan fluorescence quenching experiments using a fixed concentration of the BSA and site probes (3 μM) in buffer (containing 15 mM trisodium citrate and 150 mM NaCl at pH 7.0). The fluorescence emission spectra were recorded in the presence of increasing amounts of the compounds as quenchers with an excitation wavelength of 295 nm. The Scatchard equation (eq. 7) [S6] and plots were applied on the corrected SA–fluorescence emission spectra in order to determine the BSA–binding constant of the compounds in the presence of warfarin or ibuprofen.

S5. *In silico* computational methods

S5.1 Molecular modeling and docking calculations

The *in silico* predictive tools that have been employed to study the interaction of the compounds with the selected macromolecules, are Schrödinger, Mercury and PyMol molecular modeling software. The 3D structure of the synthesized complex **1** was generated from its X-ray crystal structure as CIF file. Mercury software (http://www.ccdc.cam.ac.uk/) was then used to convert the CIF file to PDB format file. Complexes **2-5** were built with the aid of ChemBioDraw Ultra v. 14.0.0.117 software and their structures were optimized with MM2 energy minimization force field method with the aid of ChemBio3D Ultra v. 14.0.0.117 software suite (Cambridge Soft Corporation). The best, most stable (lowest energy) conformation of the molecular model of the complexes were detected by geometrical optimization in the gas phase, as implemented in the Spartan '14 Molecular Modeling program suite (Spartan '14 v.1.1.4, Wavefunction Inc., Irvine, CA, USA; www.wavefun.com.). The structures were initially optimized (via energy minimization) by conformational search using the Monte Carlo method with the MMFF94 molecular mechanics model, included in the Spartan'14 program suite. Geometry optimization (leading to the most stable conformer with the lowest energy) was accomplished via quantum-chemical calculations by utilizing Density functional theory (DFT) computations at B3LYP level of theory with 6-31G*(d,p) basis set to describe the accurate structural and electronic properties of the compounds, implemented by Spartan' 14 program suite.

Molecular docking calculations were carried out on the crystal structure of the following target macromolecules: the crystal structure of CT DNA (PDB entry code 1BNA), *E. coli* and *S. aureus* DNA–gyrase (PDB entry codes 1KZN and 5CDM, respectively), 5–LOX (PDB entry code 6N2W), FLAP (PDB entry code 2Q7M), FLT3 (PDB entry code 6JQR), and JAK2 (PDB entry code 2B7A), to investigate the effect of the complexes on these targets. X-ray crystal structures of CT DNA dodecamer d(CpGpCpGpApApTpTpCpGpCpG) [S11] *E. coli* DNA-gyrase in complex with bound co–crystallized drug chlorobiocin (CBN) [S12], *S. aureus* DNA-gyrase in complex with bound co–crystallized drugs moxifloxacin (MFX) and QPT–1 [S13], 5–LOX bound to redox-type inhibitor nordihydroguaiaretic acid (NDGA) [S14], the integral membrane protein FLAP in complex with leukotriene biosynthesis inhibitors MK–591 and the iodinated analog of MK–591 [S15], FLT3 bound to co–crystallized inhibitor Gilteritinib, and JAK2 bound to co–crystallized inhibitor CMP6, were obtained from the Brookhaven Protein Data Bank (operated by the Research Collaboratory for Structural Bioinformatics, RCSB) [S16-S18]. The crystal structures of *E. coli* and *S. aureus* DNA-gyrase enzymes have been refined at 2.3 Å and 2.5 Å resolution, respectively, while the crystal

structures of 5–LOX bound with NDGA at 2.71 Å resolution, FLAP bound with MK–591 at 4.25 Å resolution, FLT3 at 2.20Å resolution, and JAK2 at 2.00 Å resolution.

In our studies, molecular docking calculations were performed with Schrödinger modeling suite having the ability for accurate calculations. The Schrödinger software suite contains a broad array of computational chemistry tools. In the procedure for molecular docking with the employment of Schrödinger suite all compounds were sketched and converted into three-dimensional MOL2 files using Schrödinger Release 2020-3 Maestro Version 11.1 and minimized using LigPrep 3.5 [S19] (which can generate a number of structures from each input structure with various ionization states, tautomers, stereochemical characteristics, and ring conformations to eliminate molecules on the basis of various criteria such as molecular weight or specified numbers and types of functional groups with correct chiralities for each successfully processed input structure), and the OPLS3 (**O**ptimized **P**otential for **L**iquid **S**imulations) [S20] force field for the optimization, producing the low–energy isomers of the ligands (Schrödinger, http://www.schrodinger.com). Energy minimized 3D molecular structures were generated with the employment of LigPrep run from Maestro utility of the Schrödinger suite. The ligand preparation included 2D–3D conversions, generating variations, correction, verification and optimization of the structures. A preparation of receptor and ligand structures was integrated before the actual docking procedure [S21]. The crystal structures of the proteins were prepared using the Protein Preparation Wizard [S22], in Schrödinger Suite 2020-3 (Schrödinger, LLC, New York, NY) [S22,S23]. Protein was prepared by adding the hydrogen atoms, optimizing hydrogen bonds, removing atomic clashes, adding formal charges to the hetero groups and then optimizing at neutral pH. Missing loops and side chains were prepared using Prime version 3.2 [S24,S25]. Finally, the structure was minimized using OPLS3 force field. Active site of studied proteins was obtained using SiteMap tool (version 3.6, Schrödinger) [S26,S27], which provides a fast and effective means of identifying potential binding pockets of proteins. SiteMap identifies the character of binding sites using novel search and assesses each site by calculating various properties like size, volume, amino acid exposure, enclosure, contact, hydrophobicity, hydrophilicity and donor/acceptor ratio. Receptor grid was generated around the active site for effective binding using Receptor grid generation in the Glide (version 5.9) application of Maestro. Once the receptor grid is generated, the ligands are docked to the proteins using Glide docking tool of Schrödinger (**G**rid based **LI**gand **D**ocking with **E**nergetics) (version 6.8) [S23,S28]. Compounds were docked in the binding site of the proteins using Induced-Fit Docking (IFD) protocol 2020-3 [S28-S30]. The ligand interactions are shown in Ligand interaction tool of Maestro (Schrödinger). Waters were deleted with Maestro, the graphical user interface (GUI) of Schrödinger software, prior to docking. Molecular docking studies were carried out for the best fitted compounds to the model, while the final selection criteria were compounds docking scores and the presence of crucial

interactions for binding to the studied proteins [S31]. The resulting poses were examined manually and the most promising ones were redocked with IFD calculations. Poses that pass the initial screens enter the final stage of the algorithm, which involves evaluation and minimization of a grid approximation to the OPLS-AA non-bonded ligand-receptor interaction energy. Final scoring is then carried out on the energy-minimized poses. By default, Schrödinger's proprietary GlideScore [S28] multi-ligand scoring function is used to score the poses. The rescoring was performed to calculate and improved binding energy calculations with Prime's Molecular Mechanics–Generalized Born Surface Area (MM–GBSA) protocol using VSGB solvation model [S32,S33]. All complexes showed good docking scores reflecting drug-binding affinities with the studied proteins. PyMol Molecular Graphics System (Schrödinger, LLC. version 2.3.5, www.pymol.org) [S34], was used to visualize the molecules and analyze the results of the docking and to construct the molecular models.

The binding interactions of the studied complexes on the crystal structure of the following target macromolecules: CT DNA (PDB: 1BNA), *E. coli* and *S. aureus* DNA–gyrase (PDB: 1KZN and 5CDM, respectively), 5–LOX (PDB: 6N2W), and FLAP (PDB: 2Q7M), are presented in Tables S2–S4.

S5.2 *Molecular pharmacokinetic properties, drug-likeness, target proteins and toxicity prediction methods*

General pharmacological potential, pharmacokinetic properties and toxicity prediction studies were adopted with the employment of various computational tools.

S5.2.1 *Calculation of molecular properties and prediction of drug-likeness*

S5.2.1.1 *Molecular properties prediction*

Physicochemical parameters play a vital role in generation and determination of bioactivity of any compound. Molinspiration, web-based cheminformatics software, was used to explore the various parameters such as miLogP, Topological Polar Surface Area (TPSA), molecular weight **(**MW), number of atoms, number of O or N, number of OH or NH, number of rotatable bonds, volume, and drug likeness including G-protein-coupled receptors ligand (GPCR), ion channel modulator, kinase inhibitor, and nuclear receptor ligand, and the number of violations to Lipinski's rule. MiLog*P* (octanol/water partition coefficient) was calculated by the method developed by Molinspiration software as a sum of fragment-based contributions and correction factors and used to predict the permeability of molecule across the cell membrane (Molinspiration Cheminformatics, Nova Ulica, Slovensky Grob, Slovakia, 2012, http://www.molinspiration.com/.). Method is very robust and is able to process practically all organic, and most organometallic molecules. The fragment

contributions were obtained by fitting a training set of >12,000 mostly drug-like molecules with experimental Log*P* data. In general, theoretical partition coefficient miLogP show high agreement with experimental partition coefficients ($logP_{exp}$) [S35] validating the very good prediction quality of the Molinspiration miLogP model and justifying its use in the popular ZINC database for virtual screening of commercially-available compounds. Lipophilicity of a compound is a measure of its lipophilic (or hydrophobic) character. It is expressed by octanol-water partition coefficient (log*P*), which can be determined either experimentally or it can be calculated theoretically (miLogP denotes theoretical octanol/water partition coefficient calculated by Molinspiration software). A high log*P* value of a compound, i.e., a compound with high lipophilic character that is predominantly dissolved in lipids, is expected to exhibit increased cell permeability as it would be expected to enter cell membrane through simple diffusion without the need of a transport protein [S36]. Nevertheless, the high lipophilicity of a compound can be associated with increased toxicity. Although there is no strong evidence for this correlation [S37], a highly lipophilic compound may be sequestered by fatty tissue and therefore it will be difficult to excrete leading to its accumulation that will impact systemic toxicity [S38]. Therefore, in order to ensure high cell permeability for a potential drug compound, a moderate (not too high, not too low) log*P* value is needed. Indeed, it has been shown that drugs with good cell permeability exhibit moderate log*P* values [S39].

TPSA is calculated based on the methodology published by Ertl et al. [S40] as the sum of fragment-based contributions in which O- and N-centered polar fragments are to be considered and calculated by surface areas that are occupied by oxygen and nitrogen atoms and by hydrogen atoms attached to them. TPSA has been used for characterizing drug absorption, including intestinal absorption, bioavailability, Caco-2 permeability, and blood brain barrier permeability. Method for calculation of molecular volume developed by molinspiration is based on group contributions. Number of rotatable bonds (nrotb) is a simple topological parameter that measures molecular flexibility. It has been shown to be a very good descriptor of absorption and oral bioavailability of drugs [S41]. Rotatable bond is defined as any single non-ring bond, bounded to non-terminal heavy (i.e., non-hydrogen) atom. Amide C–N bonds are not considered because they are having high rotational energy barrier. Computation of volume developed at Molinspiration is based on group contributors.

It should be mentioning that although tight binding of a drug molecule to its intended target is very important for high potency, poor cell membrane permeability often translates into poor *in vivo* efficacy. Two critical properties that are related to the cell permeability of a potential drug compound are its hydrogen bonding capacity and its lipophilicity [S42–S44].

S5.2.1.2 *Drug–likeness*

Drug likeliness is a qualitative means of analysis to check whether the given molecule has drug-like properties and it is defined as a complex balance of various molecular properties and structural features which determine whether particular molecule is similar to known drugs that would affect its bioavailability, affinity to proteins, toxicity, and metabolic stability. These properties, mainly hydrophobicity/lipophilicity, electronic distribution, hydrogen bonding characteristics, molecule size, and flexibility and presence of various pharmacophoric features, influence the behavior of a molecule in a living organism, including bioavailability, transport properties, affinity to proteins, reactivity, toxicity, and metabolic stability (Molinspiration Cheminformatics, Nova Ulica, Slovensky Grob, Slovakia, 2012, http://www.molinspiration.com/.). Through the drug-likeness data, the molecular properties and structure features can be checked with regard to known drugs. There is a number of empirical methods for drug-likeness evaluation of a compound. Lipinski's rule of five (Ro5) [S45], is a rule of thumb to evaluate drug-likeness or determine if a chemical compound with a certain pharmacological or biological activity has chemical properties and physical properties (defined as simple molecular descriptors) that would make it a likely orally active drug in humans (good membrane permeability), based on the observation that most orally administered drugs are relatively small and moderately lipophilic molecules. The rule expresses molecular properties vital for a drug's pharmacokinetics in the human body, including their absorption, distribution, metabolism and elimination (ADME) components of the Lipinski's rule. However, Lipinski specifically states that the Ro5 only holds for compounds that are not substrates for active transporters [S46]. According to Ro5, most "drug-like" molecules have an octanol-water partition coefficient (log$P$) $\leq$ 5 [S47], number of hydrogen bond acceptors (HBA) $\leq$ 10 (nON, nitrogen or oxygen atoms), number of hydrogen bond donors (HBD) $\leq$ 5 (nOHNH, the total number of nitrogen–hydrogen and oxygen–hydrogen bonds), and molecular weight (MW) $\leq$ 500 Da. The violation of 2 or more of these conditions predicts a molecule as a non-orally available drug. In general, poor permeation of a compound is more likely to be observed when HBA and HBD of its molecular structure, according to Ro5, exceed the values of 10 and 5, respectively [S48].

Nevertheless, there are many exceptions to this rule since only about 50 % of orally administered new chemical entities actually obey it [S49]. There are many compounds with two or more Ro5 violations being pursued in drug development. The partition coefficient (P) describes the propensity of a neutral (uncharged) compound to dissolve in an immiscible biphasic system of lipid (fats, oils, organic solvents) and water. In simple terms, it measures how much of a solute dissolve in the water portion versus an organic portion. Solutes that are predominantly dissolved in the water layer are called hydrophilic (water liking) and those predominantly dissolved in lipids are lipophilic (lipid liking). The partition coefficient is an important measurement of the physical nature of a substance and thereby a predictor of its behavior in different environments.

Another empirical method to distinguish compounds that are orally active, from those that are not, is given by the Veber's Rule [S41], which uses the number of rotatable bonds and the TPSA of a potential drug molecule, further questioning a 500 molecular weight cutoff. The TPSA and the nrotb have been found to better discriminate between compounds that are orally active and those that are not for a large data set of compounds in the rat. According to Veber's Rule compounds which meet only the two criteria of 10 or fewer rotatable bonds and polar surface area no greater than 140 $Å^2$ are predicted to have good oral bioavailability.

Furthermore, according to Ghose filter [S50] the number of all atoms (natoms) should be restricted from 20 to 70.

S5.2.1.3 Bioactivity score

The drug-likeness score of lead molecules and their bioactivity scores are determined with combination of GPCR, ion channel modulator, kinase inhibitor, nuclear receptor ligands, protease inhibitor, and enzyme inhibitor, which has been applied to investigate the efficiency of molecules to qualify for drug development. All the parameters were determined with the aid of Molinspiration drug-likeness score online (www.molinspiration.com). The calculated drug-likeness score of each compound was compared with the specific bodily process of each compound, and the results were compared with Naringenin. For organic molecules the probability is if the bioactivity score is (> 0), then it is active; if (–5.0–0.0), then moderately active; if (< –5.0), then inactive. The larger the value of the bioactivity score is, the higher is the probability of the specific molecule to be active [S51]. If bioactivity score of a molecule is greater than 0.00, has considerable biological activities and score between –0.50 to 0.00 is considered to be moderately active and if value is less than –0.50 it is presumed to be inactive. Drug-likeness may be defined as a complex balance of various molecular properties and structure features which determine whether particular molecule is similar to the known drugs. These properties, mainly hydrophobicity, electronic distribution, hydrogen bonding characteristics, molecule size and flexibility and of course presence of various pharmacophoric features influence the behavior of molecule in a living organism, including bioavailability, transport properties, affinity to proteins, reactivity, toxicity, metabolic stability and many others.

S5.2.2 *Prediction of toxicity*

Toxicity risk alerts are an indication that the drawn structure may be harmful concerning the risk category specified. However, risk alerts are by no means meant to be a fully reliable toxicity prediction, nor should be concluded from the absence of risk alerts that a particular substance is completely free of any toxic effect. The prediction process relies on a precomputed set of structural fragments that give rise to toxicity alerts in case they are encountered in the structure currently

drawn. These fragment lists were created by rigorously shredding all compounds of the Registry of Toxic Effects of Chemical Substances (RTECS) database known to be active in a certain toxicity class (e.g., mutagenicity). Six types of toxicity data are included: (1) primary irritation; (2) mutagenic effects; (3) reproductive effects; (4) tumorigenic effects; (5) acute toxicity; and (6) other multiple dose toxicity. Specific numeric toxicity values such as LD_{50}, LC_{50}, TDLo, and TCLo are noted as well as species studied and route of administration used.

S5.2.2.1 ***Acute rat toxicity prediction by GUSAR (on the Basis of PASS Prediction)***

In silico prediction of LD_{50} values for rats with four types of administration (oral, intravenous, intraperitoneal, subcutaneous, inhalation) were calculated with the aid of GUSAR software from the Institute of Biomedical Chemistry (IBMC), Russia [S52-S54]. GUSAR (General Unrestricted Structure-Activity Relationships) software is a tool developed to create models on quantitative structure-activity relationships/quantitative structure-property relationship (QSAR/QSPR) on the basis of the appropriate training sets represented as SD file contained data about chemical structures and endpoint quantitative data on biological activities. The output is a reliable quantitative SAR/SPR (Structure Activity and Property Relationship) model. GUSAR predicts biological activities quantitatively, based on (Q)SAR models, which can also be created with the software. GUSAR has been developed according to OECD principals and includes last achievements in the field of QSAR modeling: consensus prediction, applicability domain assessment, internal and external models validation and clearly interpretations of obtaining results. The GUSAR is a prediction module of PASS (Prediction of Activity Spectra for Substances), predicting whole bioactivity spectra for compounds qualitatively, based only on 2D structural formulae (http://www.pharmaexpert.ru/GUSAR/AcuToxPredict/). The training sets were created on the basis of data from SYMYX MDL Toxicity Database. They include the information about ~10000 chemical structures with data on acute rat's toxicity represented on the LD_{50} values (log10 (mmol/kg)) [S55]. The core of GUSAR consists of a unique algorithm of self-consistent regression allows to select the best set of MNA (Multilevel Neighborhoods of Atoms), biologically-based descriptors calculated on the basis of PASS predictions and QNA (Quantitative Neighborhoods of Atoms) descriptors for a robust and reliable QSAR model. Curated chemical gene interactions data in the training sets were retrieved from the Comparative Toxicogenomics Database (CTD), Mount Desert Island Biological Laboratory, Salisbury Cove, Maine. http://ctdbase.org/ [July, 2012].

Prediction of toxicity of the studied compounds is facilitated with the calculation of the LD_{50} values. LD_{50} value is one of important characteristics of acute toxicity that corresponds to the dose causing 50% mortality (median lethal dose) within 24 hours of administration. Acute oral, dermal and inhalation rodent toxicity are important parameters for general toxicological risk assessments,

whereas oral, intraperitoneal and intravenous acute rodent toxicity are important in drug design. It should be stressed out that computational toxicity estimations are not only faster than the determination of toxic doses in animals, but can also help to reduce the amount of animal experiments. The model of choice to predict possible toxicity of the studied compounds is the rat acute toxicity.

Rat acute toxicity predicted by GUSAR is based on the combination of QNA (Quantitative Neighborhoods of Atoms) descriptors [S52], PASS (Prediction of Activity Spectra for Substances) predicted biological activity profiles [S53-S57], and self-consistent regression [S54]. QNA descriptors are calculated based on the connectivity matrix (C), standard values of ionization potential (IP) and electron affinity (EA) of atoms in a molecule.

Toxic doses are often given as LD_{50} values in mg/kg body weight. The LD_{50} is the median lethal dose meaning the dose at which 50% of test subjects die upon exposure to a compound [S58]. Using acceptable toxicity scales, the chemicals are assigned to various groups. One of the most common scales used is the Gosselin, Smith and Hodge scale using the following thresholds: class 1 ($LD_{50} \leq 5$ mg/kg) is the highest toxicity category (extremely or super toxic), class 2 (extremely toxic) includes chemicals with $5 < LD_{50} \leq 50$ mg/kg, class 3 (very toxic) includes chemicals with $50 < LD_{50} \leq 500$ mg/kg, class 4 (moderately toxic) includes chemicals with $500 < LD_{50} \leq 5.000$ mg/kg), class 5 (slightly toxic) includes chemicals with $5.000 < LD_{50} \leq 15.000$ mg/kg, and practically non-toxic with $LD_{50} > 15.000$ mg/kg (Canadian Center for Occupational Health and Safety. What is an LD50 and LC50. http://www.ccohs.ca/oshanswers/chemicals/LD50.html#_1_6.).

S5.2.2.2 ***Substrate/Metabolite specificity Prediction (SMP)***

SMP is a web–service for *in silico* prediction substrate/metabolite specificity. Prediction of interaction with 18 cytochrome P450 and UGT isoforms: CYP1A2, CYP2C9, CYP2C19, CYP2D6, CYP3A4, UGT1A10, UGT1A1, UGT2B7, UGT1A7, UGT2B15, UGT1A8, UGT1A4, UGT2B17, UGT2B10, UGT1A3, UGT1A9, UGT1A6, UGT2B4. Prediction is based on PASS (Prediction of Activity Spectra for Substances) technology (http://www.way2drug.com/PASSonline) and MNA descriptors.

S5.2.2.3 ***Sites of metabolism prediction (SOMP)***

The sites of metabolism prediction (SOMP) [S59] is an *in silico* tool for the prediction of SOMs for drug-like compounds for (five major human) cytochrome P450s: CYP1A2, CYP2C9, CYP2C19, CYP2D6 and CYP3A4. Also in the training set, the sites of glucoronidation catalyzed by UGT were included. It is based on PASS technology and Labelled Multilevel Neighborhoods of Atoms (LMNA) descriptors. A set of all possible structures with one labeled atom (SoLAs) with the

appropriate LMNA descriptors is generated for a new compound under the prediction of sites of metabolism (SOMs). The results of prediction of SOMs for new compounds are created on the basis of the prediction results of all SoLAs generated for a compound. Each SoLA relates to one appropriate SOM. For every SoLA the following values calculate the probability values Pt and Pf (Pt is the probability that labeled atom in the SoLA is the SOM of the appropriate enzyme and Pf is the probability that the labeled atom in SoLA is not the SOM of the appropriate enzyme). The atoms in compounds are arranged according to ΔP (Pt–Pf) values. The Invariant Accuracy of Prediction (IAP) criterion, similar to AUC (the area under the receiver operating characteristic (ROC) curve), was used to estimate the accuracy of the created method. Mathematically, IAP values equal the probability that the ΔP estimation has a higher value for a randomly selected positive example (SoLAs in which labeled atom is a SOM, ΔP+) than for a randomly selected negative example (SoLAs in which labeled atom is not a SOM, ΔP–):

IAP = Probability (ΔP+>ΔP–).

S5.2.2.4 *ROSC–Pred: Rodent Organ–Specific Carcinogenicity Prediction*

ROSC–Pred is an *in silico* predictive tool for rodent organ–specific carcinogenicity prediction on the basis of structural formula of compounds [S60]. Prediction is based on PASS (Prediction of Activity Spectra for Substances) technology and training sets created on the basis of data from Carcinogenic Potency Database (CPDB). The CPDB data are available on EPA Distributed Structure-Searchable Toxicity (DSSTox) Public Database Network (ftp://ftp.epa.gov/dsstoxftp/DSSTox_Archive_20150930/CPDBAS_DownloadFiles/). For input data on the structure of test compound it may be used SMILES and MOL files or even the integrated Marvin Sketch Javascript applet.

S5.2.2.5 *Quantitative prediction of* anti-target *interaction profiles for chemical compounds by GUSAR software.*

GUSAR software was developed to create QSAR/QSPR models on the basis of the appropriate training sets represented as SD file contained data about chemical structures and endpoint in quantitative terms. The QSAR models for the sets of thirty–two end–points (IC_{50}, K_i and K_{act}) include the data about 4000 chemical compounds interacting with 18 anti-target proteins (13 receptors, 2 enzymes and 3 transporters) [S61].

CHARACTERISTICS OF QSAR MODELS FOR ANTITARGETS SETS

Activity Name	End-point	Number of compounds Training set /	Number of models	R2 training set	Q2 training set	R2 test set	Coverage,%

		Test set					
5-hydroxytryptamine 1B receptor antagonist	IC_{50}	297 / 74	8	0.83	0.79	0.67	100.0
5-hydroxytryptamine 1B receptor antagonist	Ki	266 / 66	7	0.73	0.66	0.72	100.0
5-hydroxytryptamine 2A receptor antagonist	IC_{50}	555 / 143	13	0.83	0.78	0.71	98.6
5-hydroxytryptamine 2A receptor antagonist	Ki	1010 / 252	13	0.72	0.65	0.59	99.6
5-hydroxytryptamine 2C receptor antagonist	IC_{50}	128 / 32	18	0.77	0.73	0.58	100.0
5-hydroxytryptamine 2C receptor antagonist	Ki	487 / 121	14	0.74	0.66	0.62	99.2
alpha1a adrenergic receptor antagonist	IC_{50}	438 / 111	16	0.79	0.73	0.72	98.2
alpha1a adrenergic receptor antagonist	Ki	1366 / 344	5	0.83	0.79	0.80	97.0
alpha1b adrenergic receptor antagonist	Ki	410 / 102	17	0.73	0.66	0.63	100.0
alpha-2A adrenergic receptor antagonist	IC_{50}	109 / 27	16	0.88	0.84	0.75	100.0
alpha-2A adrenergic receptor antagonist	Ki	525 / 131	17	0.84	0.79	0.77	99.2
amine oxidase [flavin-containing] A inhibitor	IC_{50}	286 / 71	9	0.80	0.75	0.72	100.0
amine oxidase [flavin-containing] A inhibitor	Ki	60 / 15	5	0.73	0.62	0.64	100.0
androgen receptor antagonist	IC_{50}	116 / 29	8	0.79	0.73	0.67	100.0
carbonic anhydrase II activator	K_{act}	104 / 26	20	0.92	0.90	0.91	100.0
carbonic anhydrase I activator	K_{act}	108 / 27	12	0.98	0.97	0.93	100.0
carbonic anhydrase I inhibitor	Ki	935 / 234	11	0.91	0.86	0.86	98.3
carbonic anhydrase II inhibitor	IC_{50}	866 / 217	7	0.87	0.79	0.76	98.6
d(1A) dopamine receptor antagonist	IC_{50}	126 / 31	11	0.76	0.72	0.80	100.0
d(1A) dopamine receptor antagonist	Ki	291 / 73	10	0.72	0.66	0.57	100.0
d3 dopamine receptor antagonist	Ki	822 / 206	9	0.73	0.66	0.62	98.0
delta-type opioid receptor antagonist	Ki	1044 / 261	16	0.75	0.70	0.65	98.5
estrogen receptor antagonist	IC_{50}	402 / 100	4	0.66	0.61	0.70	97.0
estrogen receptor antagonist	Ki	255 / 68	13	0.76	0.71	0.70	100.0
kappa-type opioid receptor antagonist	Ki	884 / 221	7	0.74	0.67	0.65	100.0
mu-type opioid receptor antagonist	IC_{50}	545 / 136	7	0.67	0.61	0.70	97.8
mu-type opioid receptor antagonist	Ki	1354 / 338	4	0.69	0.62	0.60	96.7
sodium- and chloride-dependent GABA transporter 1 antagonist	IC_{50}	75 / 19	10	0.9	0.86	0.89	100.0
sodium-dependent dopamine transporter antagonist	IC_{50}	920 / 230	5	0.7	0.65	0.67	98.3
sodium-dependent dopamine transporter antagonist	Ki	655 / 164	7	0.77	0.69	0.64	100.0
sodium-dependent serotonin transporter antagonist	IC_{50}	796 / 199	7	0.8	0.75	0.69	97.5

sodium-dependent serotonin transporter antagonist	Ki	823 / 206	2	0.72	0.65	0.61	95.6

S5.2.2.6 ***Environmental ecotoxicity predicted by GUSAR***

Quantitative prediction of ecotoxicity for chemical compounds by GUSAR software. The QSAR models were developed for the following endpoints: 96–h fathead minnow 50% lethal concentration, 48–h daphnia magna 50% lethal concentration, Tetrahymena pyriformis 50% growth inhibition concentration and Bioconcentration Factor.

CHARACTERISTICS OF QSAR MODELS FOR ECOTOXICITY PREDICTIONS

**Activity Name**	**N train**	**N test**	**N models**	**R2**	**Q2**	**R2 test**	**RMSE test**	**Coverage, %**
Fathead Minnow LC50 Log10(mmol/L)	462	115	173	0.83	0.79	0.83	0.68	100
Bioaccumulation factor Log10(BCF)	478	120	196	0.83	0.80	0.85	0.56	100
Tetrahymena pyriformis IGC50 Log10(mol/L)	874	219	196	0.91	0.88	0.87	0.39	100
Daphnia magna LC50 -Log10(mol/L)	269	68	117	0.82	0.78	0.65	0.99	100

N train - number of compounds in the training set;
N test - number of compounds in the test set;
R2 - average R2 of the models calculated for the appropriate training set;
Q2 - average Q2 of the models calculated for the appropriate training set;
Coverage - % compounds from the test set in Applicability Domain.

S5.2.2.7 ***CLC–Pred: in silico prediction of cytotoxicity for tumor and non-tumor cell lines***

CLC–Pred (Cell Line Cytotoxicity Predictor) is an *in silico* predictive tool for the prediction of cytotoxic effect of chemical compounds in non–transformed and cancer cell lines, based on structural formula. CLC–Pred provides a prediction of the cytotoxicity of a chemical compound to assess the relevance of the substance's inclusion in experimental screening. Prediction is based on PASS (Prediction of Activity Spectra for Substances) technology (http://www.way2drug.com/PASSonline) and the training set created on the basis of data on cytotoxicity retrieved from ChEMBLdb (version 23) (https://www.ebi.ac.uk/chembldb/). For input data on the structure of test compound it may be used SMILES, MOL file or even the integrated Marvin Javascript applet.

S5.2.2.8 ***Prediction of activity spectra***

Virtual target screening was adopted in order to computationally screen one selected compound against a collection of virtual protein structures, to determine if a particular protein would be a potential target of a compound of interest. Understanding chemical-biological interactions is rather a complicated task because of the multifaceted structure-function relationships in biological

systems. Prediction of activity spectra for substances (PASS) project encompasses a computational tool being able to predict the complete biological activity profile of drug-like compounds [S62]. PASS may be used to estimate general pharmacological potential of compounds under study. Since computing is carried out based on a structural formula, the prediction may be obtained for compounds that are just designed on the computer, neither synthesized nor tested yet [S63]. Based on prediction, one may select which of the proposed structures are the most promising for a particular purpose.

The PASS estimates the probable biological activity profiles for compounds under study based on their structural formulae presented in MOL file or SDF file format. PASS prediction evaluates the general biological potential of a drug-like molecule, based on the comparison of the user's compound to a database about structure-activity relationships of more than 313,000 of drug-like biologically active compounds, using the Multilevel Neighborhoods of Atoms (MNA) structure descriptors. General list of predictable biological activities consists of over 6,400 terms including pharmacotherapeutic effects (e.g. antiarrhythmic, antihypertensive, hepatoprotectant, nootropic, etc.), biochemical mechanisms of action (e.g. cyclooxygenase inhibitor, 5 hydroxytryptamine antagonist, acetylcholine M1 receptor agonist, etc.), adverse & toxic effects (e.g. carcinogenic, mutagenic, hematotoxic, etc.), metabolism (e.g. CYP3A4 and CYP1A1 inhibition, CYP1A inducer, etc.), gene expression regulation (e.g. TH expression enhancer, TNF expression inhibitor, VEGF expression inhibition), transporter-related activities (e.g. P–glycoprotein 3 inhibitor, nucleoside transporters inhibitors) [S63].

S5.2.2.9 *Drug–induced gene expression profiles prediction (DIGEP–Pred)*

Drug-Induced Gene Expression Profiles Prediction (DIGEP–Pred) is a web-service for *in silico* prediction of drug–induced changes of gene expression profiles based on structural formula. Prediction of drug–induced changes of gene expression with DIGEP–Pred *in silico* tool is based on PASS technology and two training sets created on the basis of data on drug–induced changes of gene expression profiles retrieved from Comparative Toxicogenomics Database (CTD), Connectivity Map Database and L1000 Project [S64]. *In silico* prediction is based on the structural formula of the tested compound. The gene expression changes can be considered as a particular type of the biological activity of a drug. The probability "to be active" (Pa) estimates the chance that the studied compound belongs to the sub-class of active compounds (resembles the structures of molecules, which are the most typical in a sub–set of "actives" in PASS training set). The probability "to be inactive" (Pi) estimates the chance that the studied compound belongs to the sub-class of inactive compounds (resembles the structures of molecules, which are the most typical in a sub-set of "inactives" in PASS training set).Curated chemical gene interactions data in the training

sets were retrieved from the Comparative Toxicogenomics Database (CTD), Mount Desert Island Biological Laboratory, Salisbury Cove, Maine. http://ctdbase.org/ [October, 2015].

S6 References

S1 Kontogiorgis, C.; Hadjipavlou–Litina, D. Biological evaluation of several coumarin derivatives designed as possible anti-inflammatory/antioxidant agents, *J. Enz.Inhib. Med. Chem.* **2003**, 18, 63–69.

S2 Ruch, R.J.; Cheng,C.; Klaunig, J.E. Prevention of cytotoxicity and inhibition of intercellular communication by antioxidant catechins isolated from Chinese green tea, Carcinogenesis **1989**, 10, 1003–1008.

S3 Andrews, J.M. Determination of minimum inhibitory concentrations, J. *Antimicrob. Chemotherapy*, **2001**, 48(S1) 5–16.

S4 Irgi, E.P.; Geromichalos, G.D.; Balala, S.; Kljun, J.; Kalogiannis, S.; Papadopoulos, A.; Turel, I.; Psomas, G. Cobalt(II) complexes with the quinolone antimicrobial drug oxolinic acid: structure and biological perspectives, *RSC Adv*., **2015**, 5, 36353–36367.

S5 Wolfe, A.; Shimer, G.; Meehan, T. Polycyclic Aromatic Hydrocarbons Physically Intercalate into Duplex Regions of Denatured DNA. *Biochemistry***1987**, *26*, 6392–6396.

S6 Lakowicz, J.R. Principles of Fluorescence Spectroscopy, 3rd ed. Plenum Press, New York, **2006**.

S7 Heller, D.P.; Greenstock, C.L. Fluorescence lifetime analysis of DNA intercalated ethidium bromide and quenching by free dye. *Biophys. Chem.* **1994**, *50*, 305–312.

S8 Stella, L.; Capodilupo, A.L.; Bietti, M. A reassessment of the association between azulene and [60]fullerene. Possible pitfalls in the determination of binding constants through fluorescence spectroscopy,*Chem. Commun*., **2008**, 4744–4746.

S9 Wang, Y.; Zhang, H.; Zhang, G.; Tao, W.; Tang, S. Interaction of the flavonoid hesperidin with bovine serum albumin: A fluorescence quenching study, *J. Lumin.* **2007**, 126, 211–218.

S10 Lazou, M. Tarushi, A. Gritzapis, P. Psomas, G. Transition metal complexes with a novel guanine-based (E)-2-(2-(pyridin-2-ylmethylene)hydrazinyl)quinazolin-4(3H)-one: Synthesis, characterization, interaction with DNA and albumins and antioxidant activity, *J. Inorg. Biochem*. **2020,**206, 111019.

S11 Drew, H.R.; Wing, R.M.; Takano, T.; Broka, C.; Tanaka, S.; Itakura, K.; Dickerson, R. E. Structure of a B-DNA dodecamer: conformation and dynamics, *Proc. Natl. Acad. Sci*. **1981**,78, 2179–2183.

S12 Lafitte, D.; Lamour, V.; Tsvetkov, P.O.; Makarov, A.A; Klich, M.; Deprez, P.; Moras, D.; Briand, C.; Gilli, R. DNA gyrase interaction with coumarin-based inhibitors: the role of the hydroxybenzoate isopentenyl moiety and the 5'-methyl group of the noviose, *Biochemistry* **2002**, 41, 7217–7223.

S13 Chan P.F.; Srikannathasan, V. Huang, J.; Cui, H.; Fosberry, A.P.; Gu, M.; Hann, M.M.; Hibbs, M.; Homes, P.; Ingraham, K.; Pizzollo, J.; Shen, C.; Shillings, A.J.; Spitzfaden, C.E.; Tanner, R.; Theobald, A,J.; Stavenger, R.A.; Bax, B.D.; Gwynn, M.N. Structural basis of DNA gyrase inhibition by antibacterial QPT-1, anticancer drug etoposide and moxifloxacin. *Nat. Commun.* **2015,** 6, 10048.

S14 Gilbert, N.C.; Gerstmeier, J.; Schexnaydre, E.E.; Borner, F.; Garscha, U.; Neau, D.B; Werz, O.; Newcomer, M.E. Structural and mechanistic insights into 5-lipoxygenase inhibition by natural products. *Nat. Chem. Biol.* **2020**, 16, 783–790.

S15 Ferguson, A.D.; McKeever, B.M.; Xu, S.; Wisniewski, D.; Miller, D.K.; Yamin, T.T.; Spencer, R.H.; Chu, L.; Ujjainwalla, F.; Cunningham, B.R.; Evans, J.F.; Becker, J.W. Crystal structure of inhibitor-bound human 5-lipoxygenase-activating protein. *Science* **2007**,317, 510–512.

S16 Berman, H.M.; Westbrook, J.; Feng, Z.; Gilliland, G.; Bhat, T.N.; Weissig, H.; Shindyalov, I.N.; Bourne, P.E. The Protein Data Bank, *Nucleic Acids Res.***2000**, 28 235–242.

S17 Berman, H.M.; Henrick, K.; Nakamura, H. Announcing the worldwide Protein Data Bank, *Nature Struct. Mol. Biol*. **2003**, 10, 980.

S18 Bernstein, F.C.; Koetzle, T.F.; Williams, G.J.; Meyer, E.E.; Brice, M.D.; Rodgers, J.R.; Kennard, O.; Shimanouchi, T.; Tasumi, M. The Protein Data Bank: a computer-based archival file for macromolecular structures, *J. Mol. Biol.* **1977**, 112, 535–542.

S19 Fosgerau, K.; Hoffmann, T. Peptide therapeutics: current status and future directions, *Drug Discov. Today* **2015**,20, 122–128.

S20 Shivakumar, D.; Williams, J.; Wu, Y.; Damm, W.; Shelley, J.; Sherman, W. Prediction of Absolute Solvation Free Energies using Molecular Dynamics Free Energy Perturbation and the OPLS Force Field, *J. Chem. Theory Comput.* **2010**, 6, 1509–1519.

S21 Sastry, G.M.; Adzhigirey, M.; Day, T.; Annabhimoju, R.; Sherman, W.J. Protein and ligand preparation: parameters, protocols, and influence on virtual screening enrichments, *Comput. Aided Mol. Des*. **2013**, 27, 221–234.

S22 Friesner, R.A.; Murphy, R.B.; Repasky, M.P.; Frye, L.L; Greenwood, J.R.; Halgren, T.A; Sanschagrin, P.C.; Mainz, D.T. Extra Precision Glide: Docking and Scoring Incorporating a Model of Hydrophobic Enclosure for Protein−Ligand Complexes, *J. Med. Chem.***2006**, 49, 6177–6196.

S23 Halgren, T.A.; Murphy, R.B.; Friesner, R.A.; Beard, H.S.; Frye, L.L.; Pollard, W.T.; Banks, J.L. Glide: A New Approach for Rapid, Accurate Docking and Scoring. 2. Enrichment Factors in Database Screening, *J. Med. Chem.***2004**, 47, 1750–1759.

S24 Jacobson, M.P.; Pincus, D.L.; Rapp, C.S.; Day, T.J.F.; Honig, B.; Shaw, D.E.; Friesner, R.A. A hierarchical approach to all-atom protein loop prediction, *Proteins: Structure, Function and Bioinformatics*, **2004**, 55, 351–367.

S25 Jacobson, M.P.; Friesner, R.A; Xiang, Z.; Honig, B. On the Role of the Crystal Environment in Determining Protein Side-chain Conformations, *J. Mol. Biol.* **2002**, 320, 597–608.

S26 Halgren, T. New Method for Fast and Accurate Binding-site Identification and Analysis, *Chem. Biol. Drug Des*. **2007**, 69, 146–148.

S27 Halgren, T. Identifying and Characterizing Binding Sites and Assessing Druggability, *J. Chem. Inf. Model.* **2009**, 49, 377–389.

S28 Friesner, R.A.; Banks, J.L.; Murphy, R.B.; Halgren, T.A.; Klicic, J.J.; Mainz, D.T.; Repasky, M.P.; Knoll, E.H; Shaw, D.E.; Shelley, M.; Perry, J.K; Francis, P.; Shenkin, P.S. Glide: A New Approach for Rapid, Accurate Docking and Scoring. 1. Method and Assessment of Docking Accuracy, *J. Med. Chem.***2004**, 47, 1739–1749.

S29 Farid, R.; Day, T.; Friesner, R.A; Pearlstein, R.A. New insights about HERG blockade obtained from protein modeling, potential energy mapping, and docking studies, *Bioorg. Med. Chem.* **2006**, 14, 3160–3173.

S30 Sherman, W.; Day, T.; Jacobson, M.P.; Friesner, R.A.; Farid, R. Novel Procedure for Modeling Ligand/Receptor Induced Fit Effects, *J. Med. Chem.***2006**, 49, 534–553.

S31 Geromichalos, G.D.; Alifieris, C.E.; Geromichalou, E.G.; Trafalis, D.T. Overview on the current status on virtual high-through put screening and combinatorial chemistry approaches inmulti-target anticancer drug discovery; Part II,*J. BUON* **2016,** 21, 764–779.

S32 Bashford, D.; Case, D.A. Generalized born models ofmacromolecular solvation effects, *Annu. Rev. Phys. Chem.* **2000**, 51, 129–152.

S33 Li, J.; Abel, R.; Zhu, K.; Cao,Y.; Zhao, S.; Friesner, R. A. The VSGB 2.0 model: A next generation energy model for high resolution protein structure modeling, *Proteins* **2011**,79, 2794–2812.

S34 DeLano, W.L. The PyMOL Molecular Graphics System 0.99, DeLano Scientific, San Carlos, CA, USA, **2006**.

S35 Pyka, A.; Babuska, M.; Zachariasz, M. A comparison of theoretical methods of calculation of partition coefficients for selected drugs. *Acta Pol. Pharm*. **2006**, 63, 159–167.

S36 Leung, S.S.F.; Mijalkovic, J.; Borrelli K.; Jacobson; M.P. Testing physical models of passive membrane permeation, *J. Chem. Inf. Model*. **2012**, 52, 1621–1636.

S37 Sharma, R.; Majer, F.; Peta, V.K.; Wang, J.; Keaveney, R.; Kelleher, D.; Long, A.; Gilmer, J.F. Bile acid toxicity structure–activity relationships: Correlations between cell viability and lipophilicity in a panel of new and known bile acids using an oesophageal cell line (HET-1A), *Bioorg. Med. Chem.* **2010**, 18, 6886–6895.

S38 Pliska, V.; Testa, B.; van De Waterbed H. Lipophilicity in Drug Action and Toxicology. New York: John Wiley & Sons Ltd. **1996**. 439 ISBN 978-3-527-29383-4.

S39 Darmostuk, M.; Jurášek, M.; Lengyel, K.; Zelenka, J.; Rumlová, M.; Drašar, P.; Ruml, T. Conjugation of chlorins with spermine enhances phototoxicity to cancer cells in vitro, *J.Photochem. Photobiol.* **2017**, 168, 175–184.

S40 Ertl, P.; Rohde, B.; Selzer, P. Fast calculation of molecular polar surface area as a sum of fragment-based contributions and its application to the prediction of drug transport properties, *J. Med. Chem.* **2000**, 43, 3714–3717.

S41 Veber, D.F.; Johnson, S.R.; Cheng, H.; Smith, B.R; Ward, K.W.; Kopple, K.D. Molecular properties that influence the oral bioavailability of drug candidates, *J. Med. Chem.* **2002**, 45, 2615–2623.

S42 Liu, X.; Testa, B.; Fahr, A. Lipophilicity and its relationship with passive drug permeation, *Pharm. Res*. **2011**, 28, 962–977.

S43 Refsgaard, H.H.F.; Jensen, B.F.; Brockhoff, P.B.; Padkjaer, S.B.; Guldbrandt, M.; Christensen, M.S. In silico prediction of membrane permeability from calculated molecular parameters, *J. Med. Chem*. **2005**, 48, 805–811.

S44 Li, D.; Kerns,E.H. Lipophilicity Methods in Drug-Like Properties (Second Edition), **2016**.

S45 Lipinski, C.A.; Lombardo, F.; Dominy, B.W.; Feeney, P.J. Experimental and computational approaches to estimate solubility and permeability in drug discovery and development settings, *Adv. Drug Del. Rev*. **1997**, 23, 3–25.

S46 Lipinski, C.A.; Lombardo, F.; Dominy, B.W.; Feeney, P.J. Experimental and computational approaches to estimate solubility and permeability in drug discovery and development settings, *Adv. Drug Deliv. Rev*. **2001**, 46, 3–26.

S47 Leo, A.; Hansch, C.; Elkins, D. Partition coefficients and their uses, *Chem. Rev.* **1971**, 71, 525–616.

S48 Bakht, M.A.; Yar, M.S.; Abdel-Hamid, S.G.; Qasoumi, S.I.; Samad, A. Molecular properties prediction, synthesis and antimicrobial activity of some newer oxadiazole derivatives, *Eur. J. Med. Chem.* **2010**, 45, 5862–5869.

S49 Hagan, O.S.; Swainston, N.; Handl, J.; Kell, D.B. A 'rule of 0.5' for the metabolite-likeness of approved pharmaceutical drugs, *Metabolomics* **2015**, 11, 323–339.

S50 Ghose, A.K.; Viswanadhan, V.N.; Wendoloski, J.J. A knowledge-based approach in designing combinatorial or medicinal chemistry libraries for drug discovery. 1. A qualitative and quantitative characterization of known drug databases, *J. Comb. Chem.* **1999**, 1, 55–68.

S51 Srivastava, A.K.; Tewari, M.; Shukla, H.S.; Roy, B.K. In silico profiling of the potentiality of curcumin and conventional drugs for CagA oncoprotein inactivation, *Arch. Pharm.,* **2015**, 348, 548–555.

S52 Filimonov, D.A.; Zakharov, A.V.; Lagunin, A.A.; Poroikov, V.V.; QNA-based 'Star Track' QSAR approach, *SAR QSAR,Environ. Res.* **2009**, 20, 679–709.

S53 Poroikov, V.V.; Filimonov, D.A.; Borodina, Y.V.; Lagunin, A.A.; Kos A.J. Robustness of biological activity spectra predicting by computer program PASS for noncongeneric sets of chemical compounds, *Chem. Inf. Comput. Sci*. **2000**, 40, 1349–1355.

S54 Lagunin, A.A.; Zakharov, A.V.; Filimonov, D.A.; Poroikov, V.V. A new approach to QSAR modelling of acute toxicity, *SAR QSAR, Environ. Res.* **2007**, 18, 285–298.

S55 Lagunin, A.; Zakharov, A.; Filimonov, D.; Poroikov, V. QSAR Modelling of rat acute toxicity on the basis of PASS prediction, *Mol. Informatics* **2011**, 30, 241–250.

S56 Sadym, A.; Lagunin, A.; Filimonov, D.; Poroikov, V. Prediction of Biological Activity Spectra via The Internet, *SAR QSAR Env. Res.* **2003**, 14, 339–347.

S57 Poroikov, V.V.; Filimonov, D.A.; Ihlenfeld, W.; Gloriozova, T.A.; Lagunin, A.A.; Borodina, Y.V.; Stepanchikova, A.V.; Nicklaus, M. CPASS Biological Activity Spectrum Predictions in the Enhanced Open NCI Database Browser, *J. Chem. Inf. Comput. Sci.* **2003**, 43, 228–236.

S58 Drwal, M.N.; Banerjee, P.; Dunkel, M.; Wettig, M.R.; Preissner, R. ProTox: a web server for the *in silico* prediction of rodent oral toxicity, *Nucleic Acids Res.* **201 4**(Web server issue).

S59 Rudik, A.; Dmitriev, A.; Lagunin, A.; Filimonov, D.; Poroikov, V. SOMP: web server for in silico prediction of sites of metabolism for drug-like compounds, *Bioinformatics* **2015,** 31, 2046–2048.

S60 Lagunin, A.; Rudik, A.; Filimonov, D.; Druzhilovsky, D.; Poroikov, V. ROSC-Pred: web-service for rodent organ-specific carcinogenicity prediction,*Bioinformatics,* **2017**, 34, 710–712.

S61 Zakharov, A.V.; Lagunin, A.A.; Filimonov, D.A; Poroikov, V.V. Quantitative prediction of antitarget interaction profiles for chemical compounds, *Chem. Res. Toxicol.* **2012**, 25, 2378–2385.

S62. Poroikov, V.; Akimov, D.; Shabelnikova, E.; Filimonov, D. Top 200 Medicines: Can New Actions be Discovered Through Computer-aided Prediction? *SAR and QSAR, Envir. Res.* **2001**, 12327–12344.

S63 Filimonov, D.A.; Lagunin, A.A.; Gloriozova, T.A.; Rudik, A.V.; Druzhilovskii, D.S.; Pogodin, P.V.; Poroikov, V.V. Prediction of the Biological Activity Spectra of Organic Compounds Using the Pass Online Web Resource, *Chem. Heterocycl. Comp* .**2014**, 50, 444–457.

S64 Lagunin, A.; Ivanov, S.; Rudik, A.; Filimonov, D.; Poroikov, V. DIGEP-Pred: web service for in silico prediction of drug-induced gene expression profiles based on structural formula, *Bioinformatics* **2013**, 29,2062–2063.

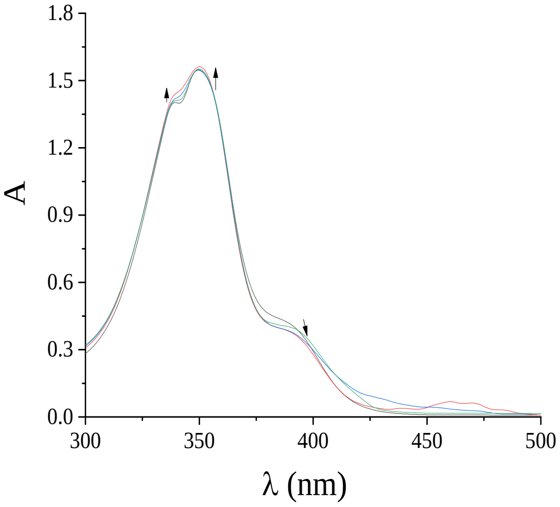

(A)

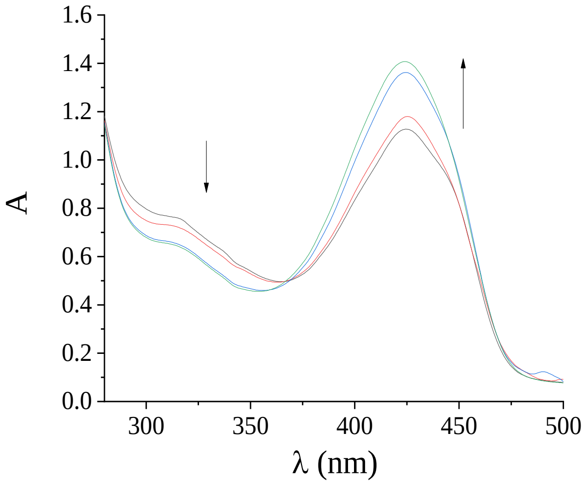

(B)

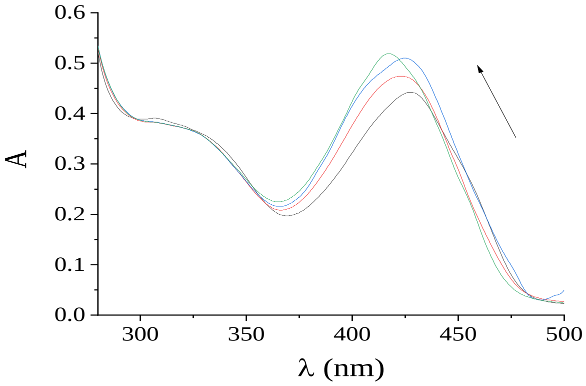

(C)

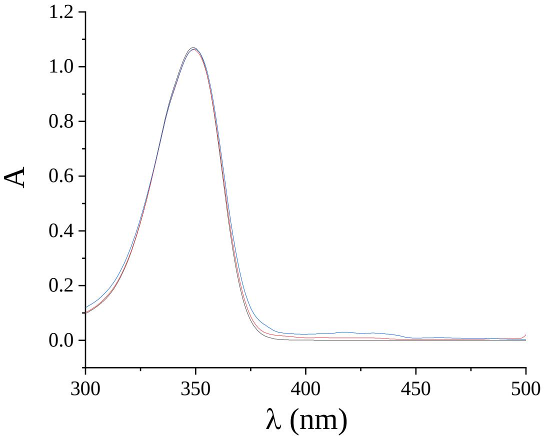

(D)

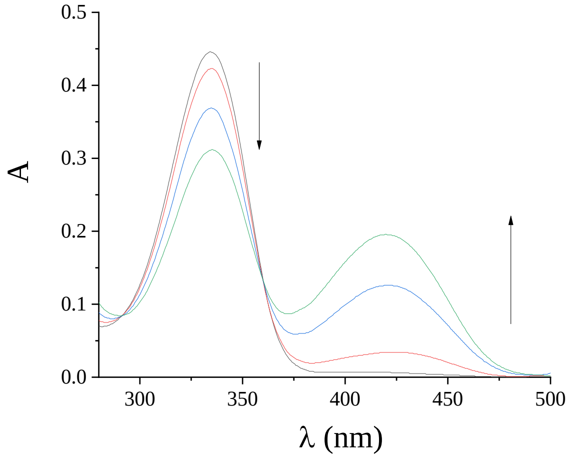

(E)

Figure S1. UV spectra of DMSO solution of (A) **1** (10^{-4} M), **(B) 3** (10^{-4} M), (C) **4** (10^{-4} M), (D) 4–Et_2N–saloH (10^{-5} M) and (E) 5–F–saloH (10^{-4} M) in the presence of increasing amounts of CT DNA. The arrows show the changes upon increasing amounts of CT DNA.

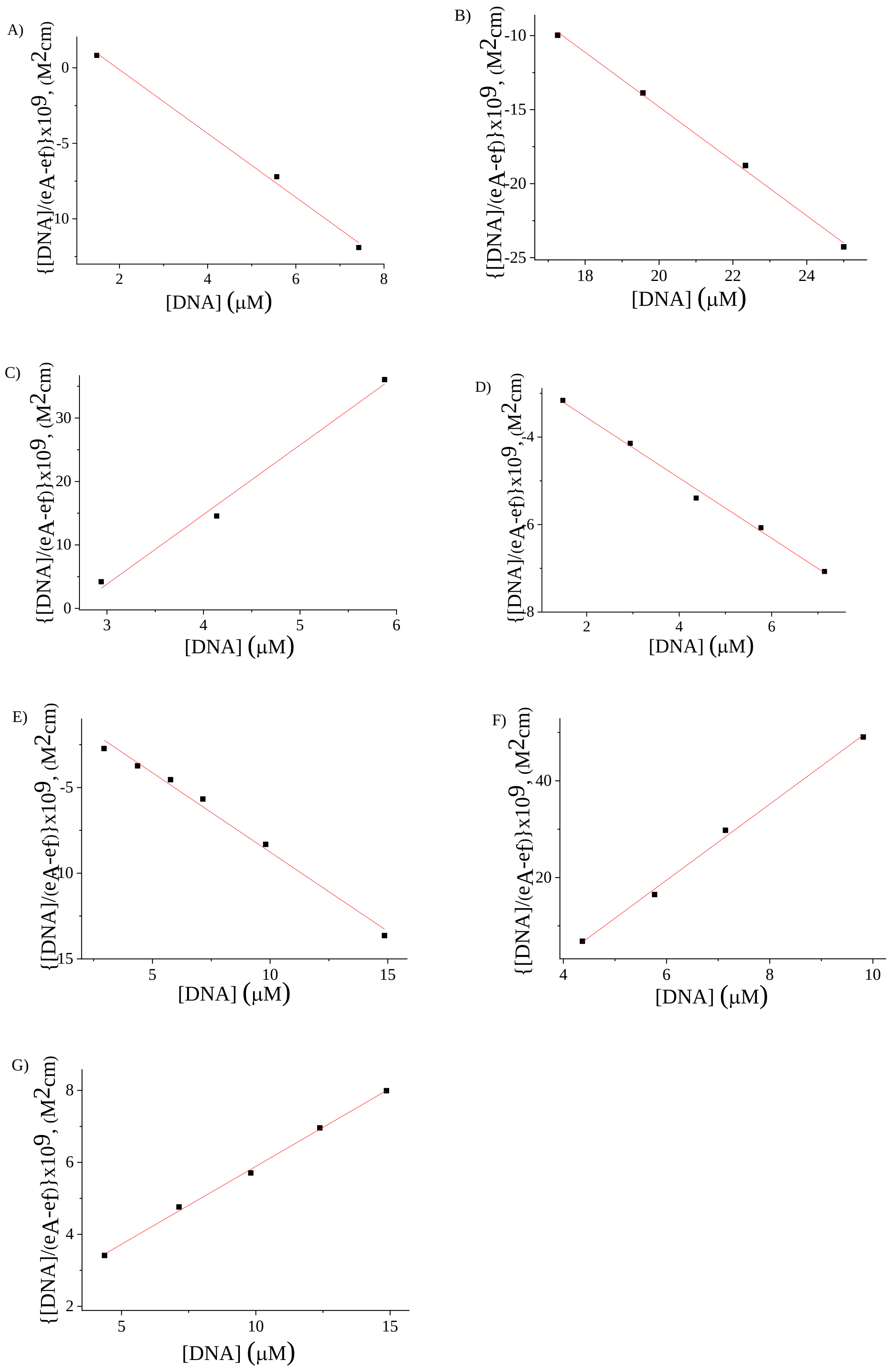

Figure S2. Plot of $\frac{[DNA]}{(\varepsilon_A - \varepsilon_f)}$ *versus* [DNA] for (A)-(G) 4–Et_2N–saloH, 5–F–saloH and complexes **1–5**, respectively**.**

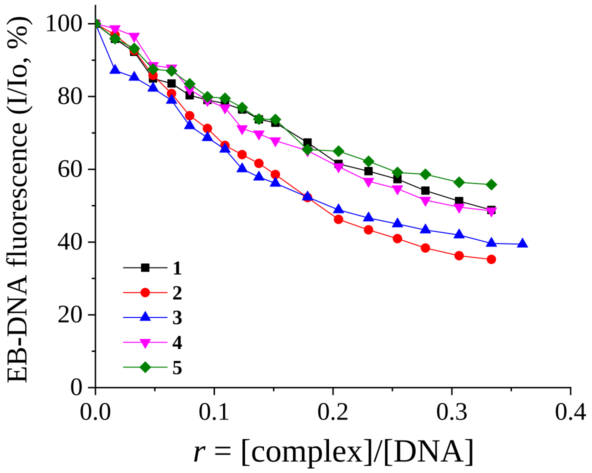

Figure S3. Plot of EB–DNA relative fluorescence emission intensity at $\lambda_{emission}$ = 592 nm (%) *versus r* (*r* = [complex]/[DNA]) in the presence of complexes **1–5** (up to 48.8 % of the initial EB–DNA fluorescence emission intensity for **1**, 35.2 % for **2**, 39.4 % for **3**, 48.6 % for **4** and 55.8% for **5**).

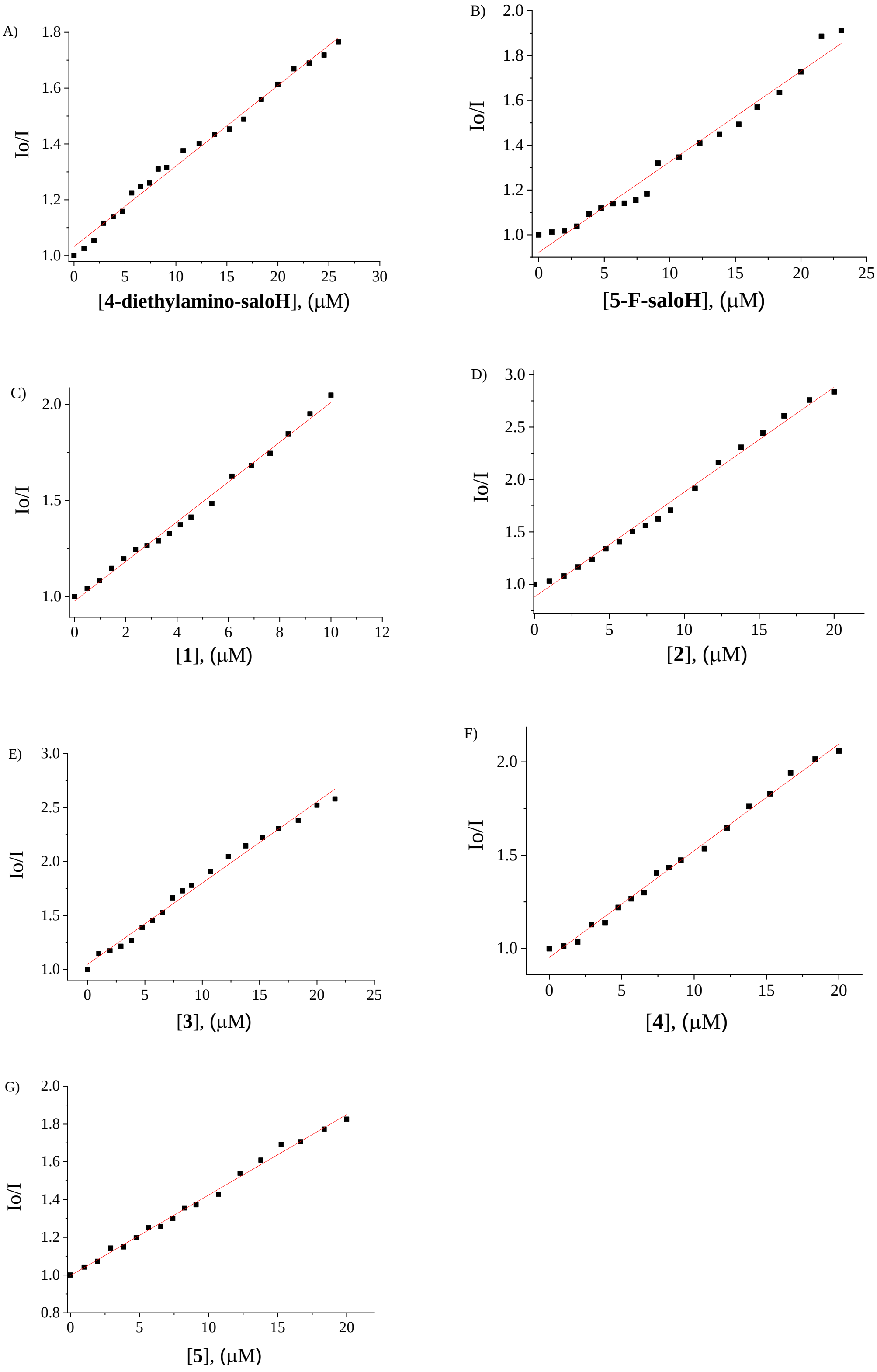

Figure S4. Stern–Volmer quenching plot of EB bound to CT DNA for (A)-(G) 4–Et_2N–saloH, 5–F–saloH and complexes **1**–**5**, respectively**.**

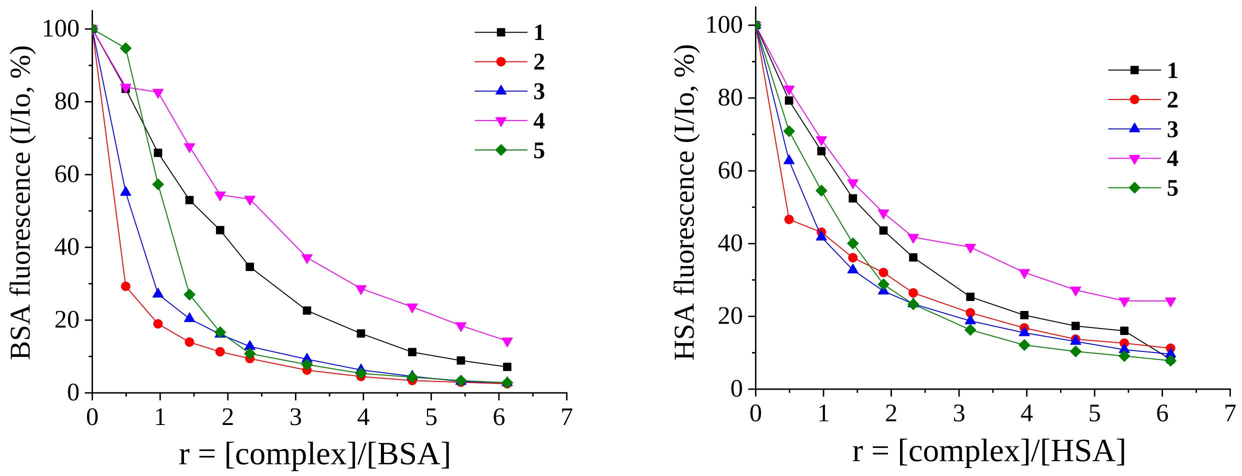

(A) (B)

Figure S5. (A) Plot of % relative BSA–fluorescence emission intensity at λ_{em}= 351 nm (%) *versus r* (*r* = [complex]/[BSA]) for complexes **1–5** (up to 7.2 % of the initial BSA fluorescence for **1**, 2.5 % for **2**, 2.8% for **3**, 14.3% for **4** and 2.8% for **5**) in buffer solution (150 mM NaCl and 15 mM trisodium citrate at pH 7.0). (B) Plot of% relative HSA–fluorescence emission intensity at λ_{em}= 342 nm (%) vs *r* (*r* = [complex]/[HSA]) for complexes **1–5** (up to 8.2% of the initial HSA fluorescence for **1**, 11.2% for **2**, 9.6% for **3**, 24.3% for **4** and 7.8 % for **5**) in buffer solution (150 mM NaCl and 15 mM trisodium citrate at pH 7.0).

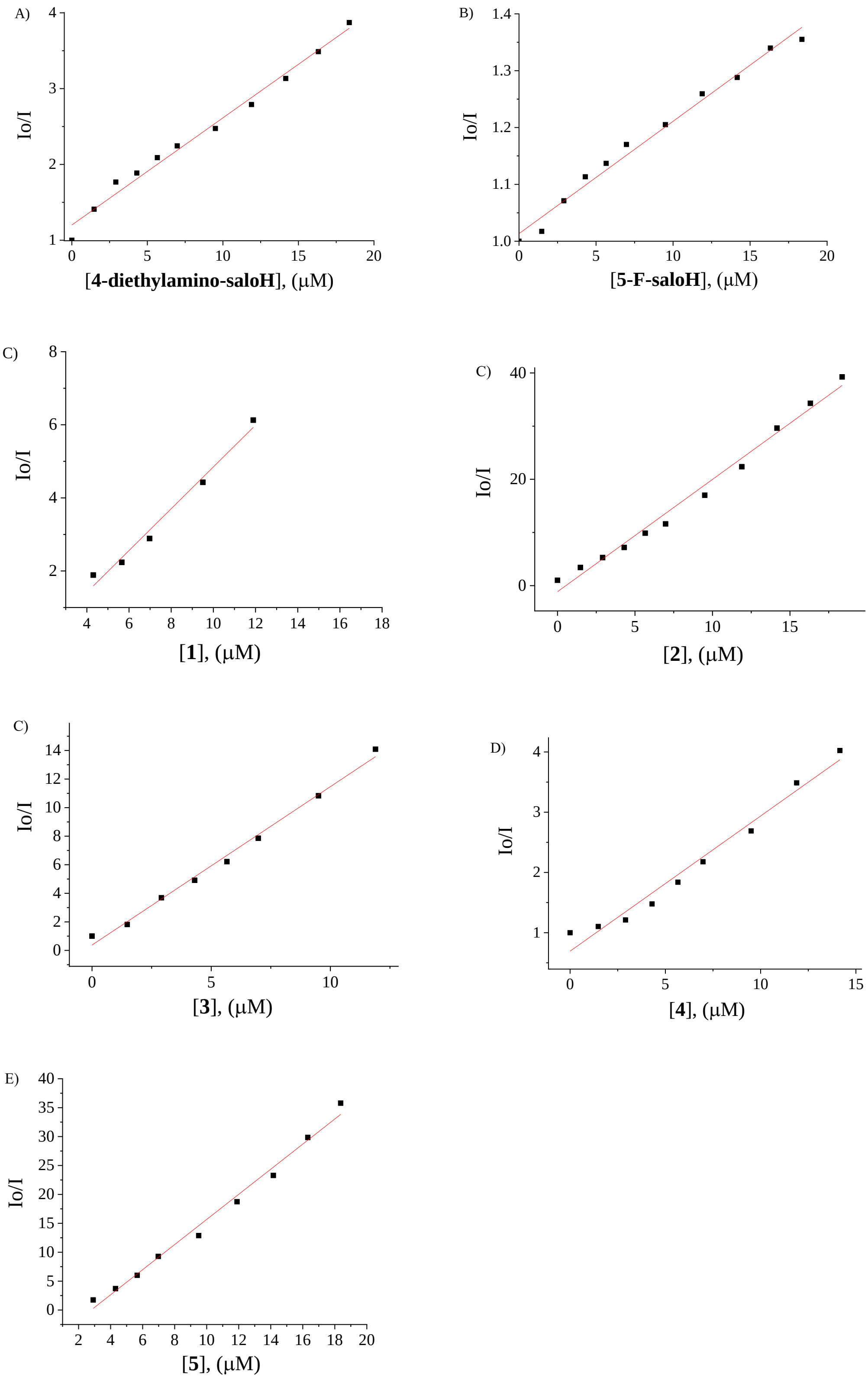

Figure S6. Stern–Volmer quenching plot of BSA for (A)-(G) 4–Et_2N–saloH, 5–F–saloH and complexes **1–5**, respectively**.**

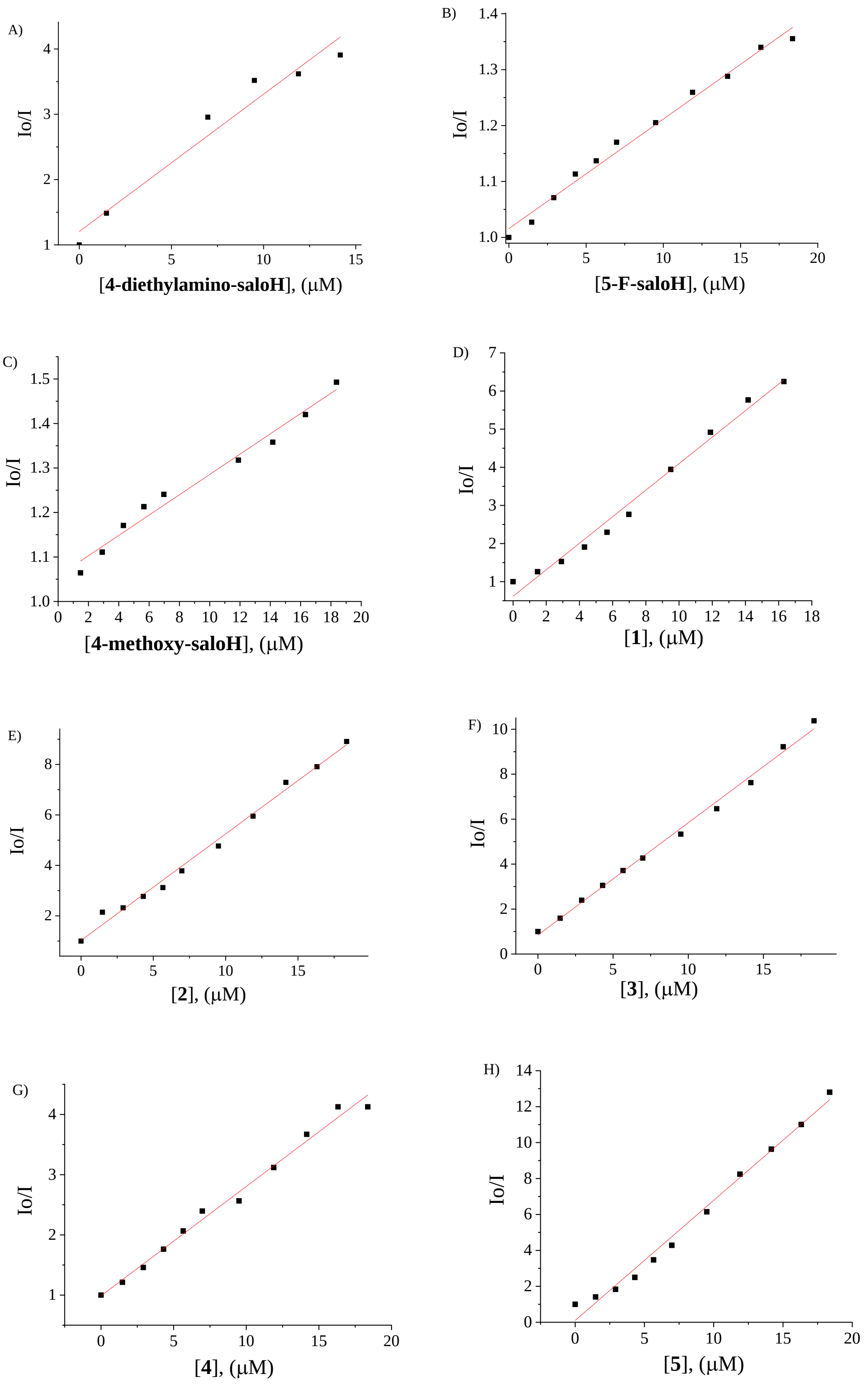

Figure S7. Stern–Volmer quenching plot of HSA for (A)-(H) 4–Et_2N–saloH, 5–F–saloH, 4–MeO–saloH and complexes **1–5**, respectively.

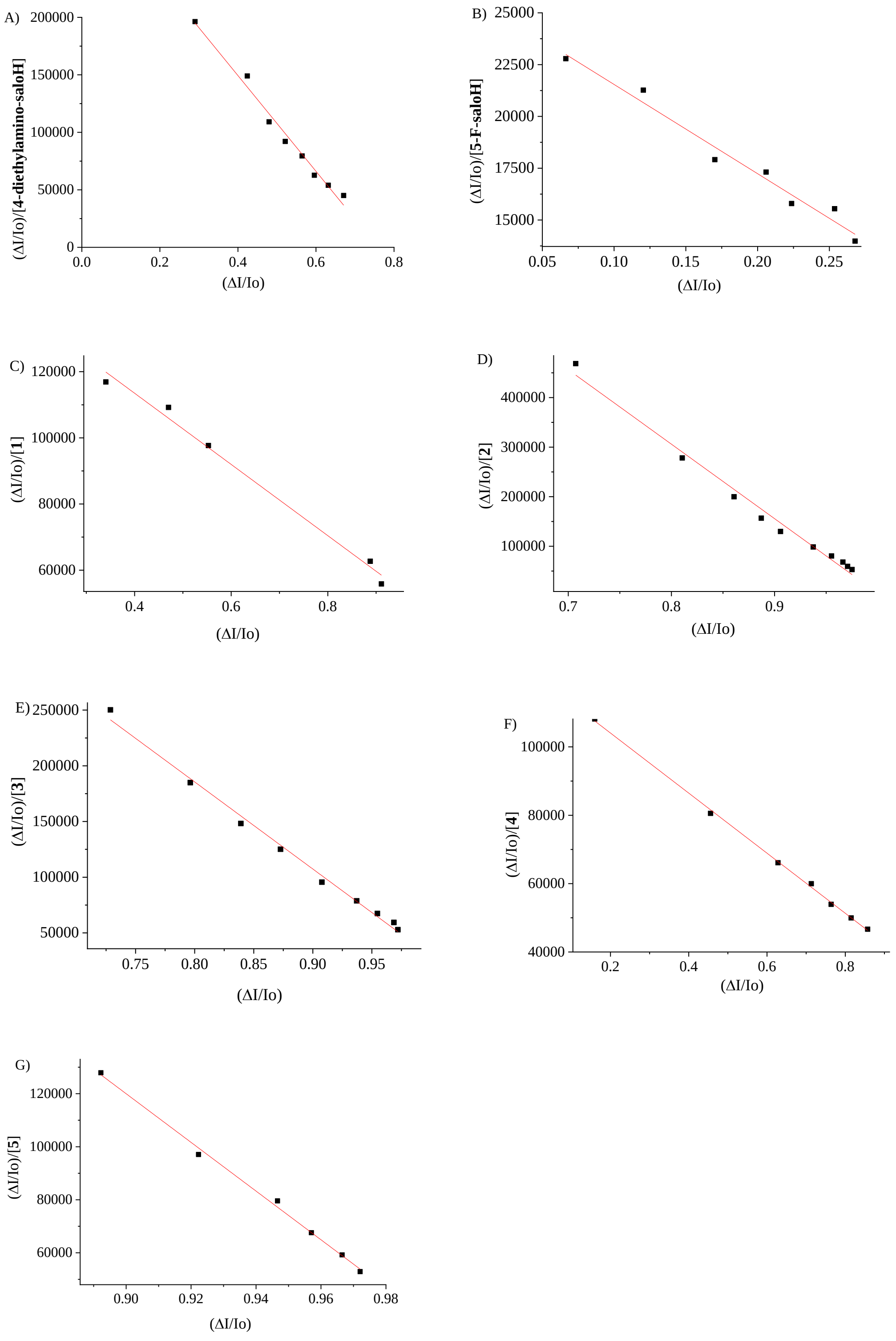

Figure S8. Scatchard plot of BSA for (A)-(G) 4–Et_2N–saloH, 5–F–saloH and complexes **1–5**, respectively.

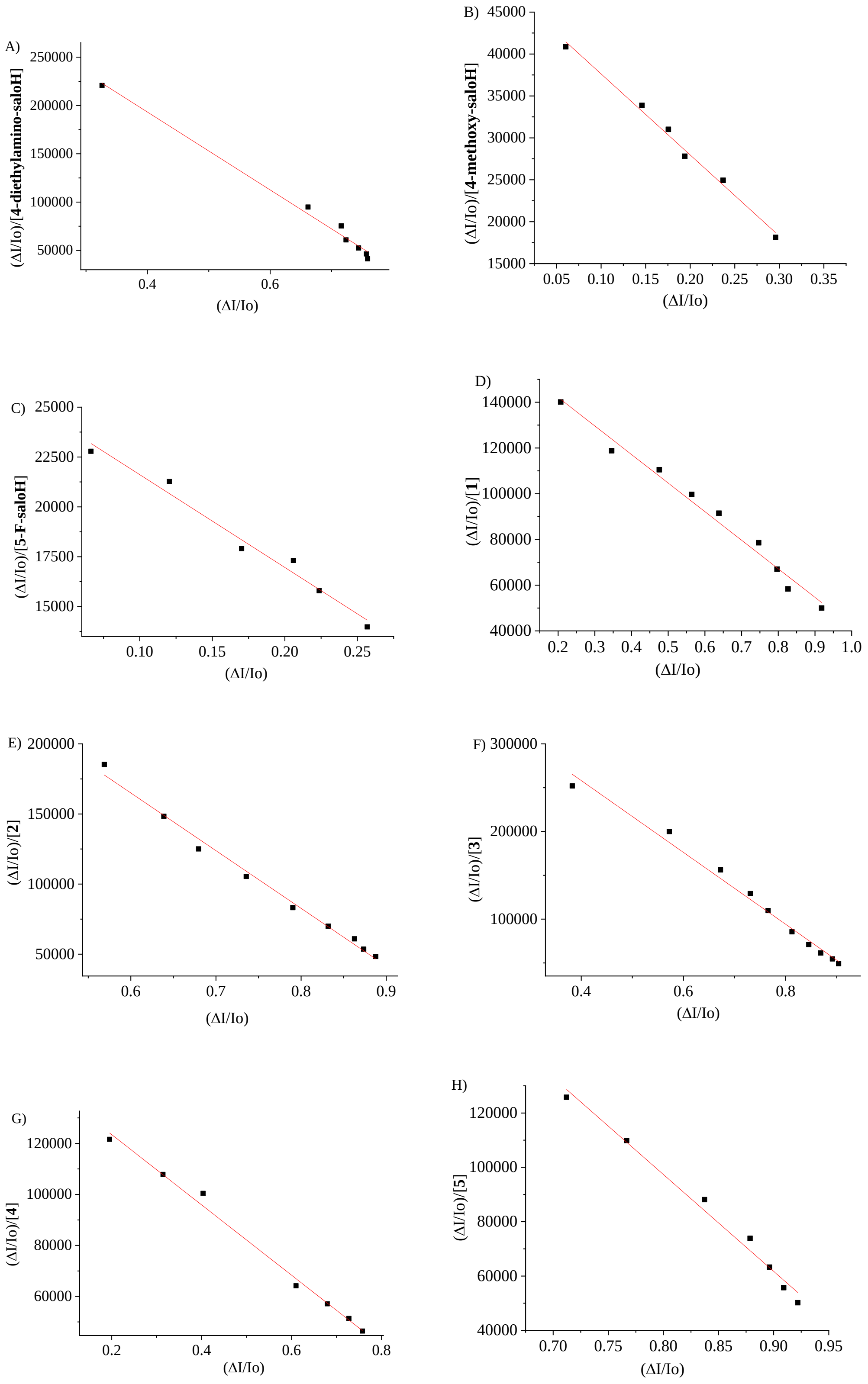

Figure S9. Scatchard plots of HSA for (A)-(H) 4–Et_2N–saloH, 5–F–saloH, 4–MeO–saloH and complexes **1–5**, respectively.

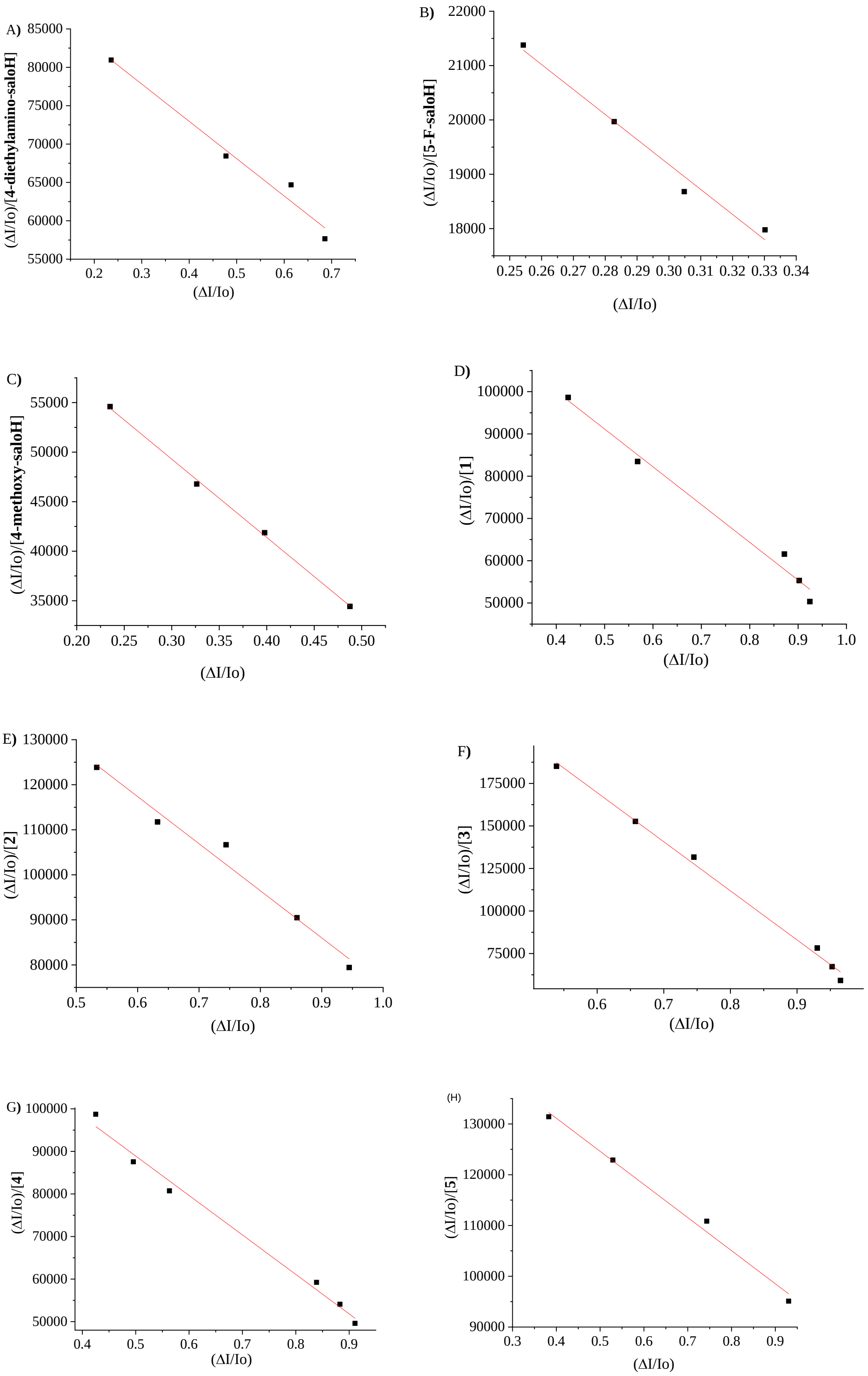

Figure S10. Scatchard plots of BSA–ibuprofen for (A)-(H) 4–Et_2N–saloH, 5–F–saloH, 4–MeO–saloH and complexes **1–5**, respectively.

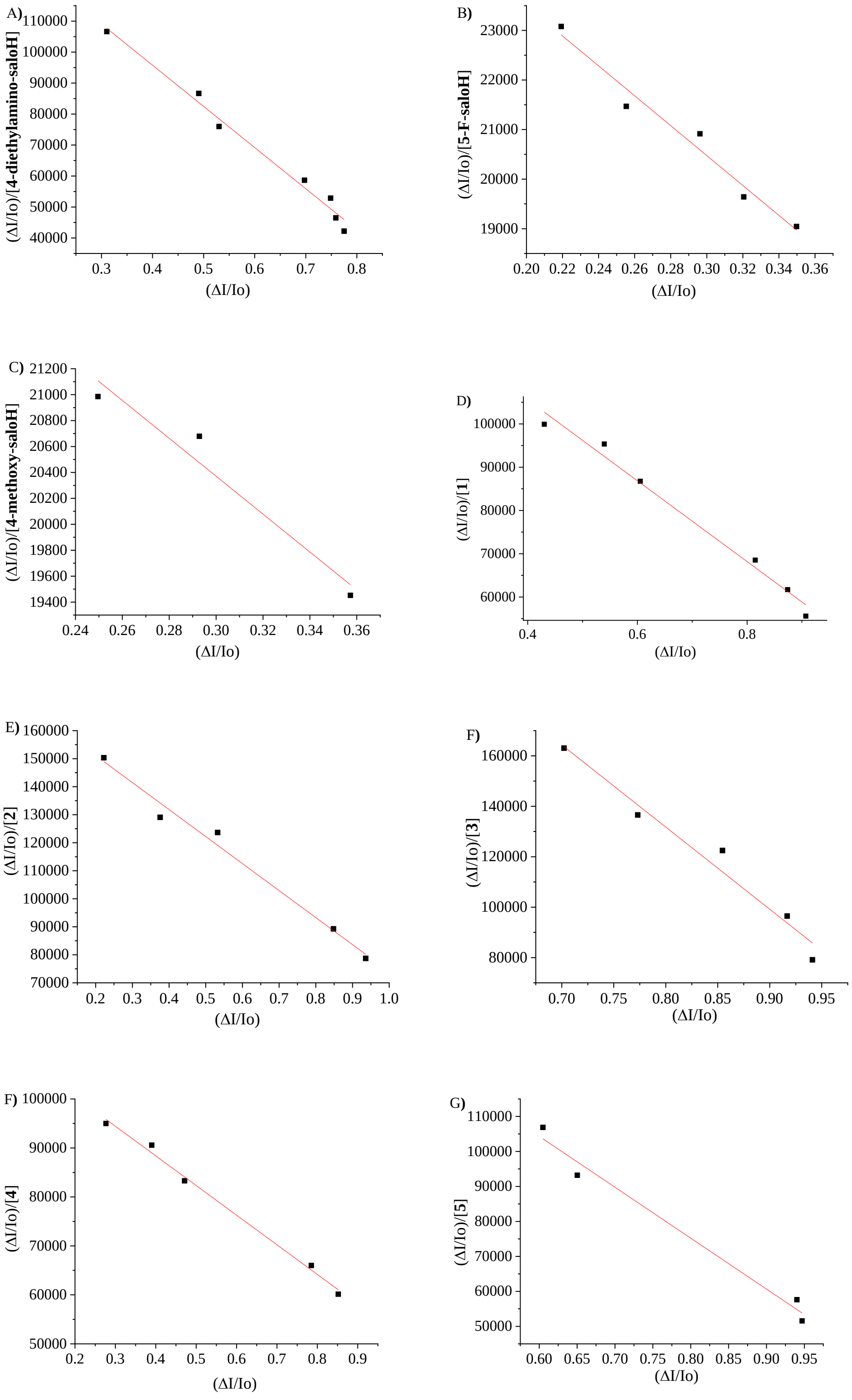

Figure S11. Scatchard plot of BSA–warfarin for (A)-(H) 4–Et_2N–saloH, 5–F–saloH, 4–MeO–saloH and complexes **1–5**, respectively.

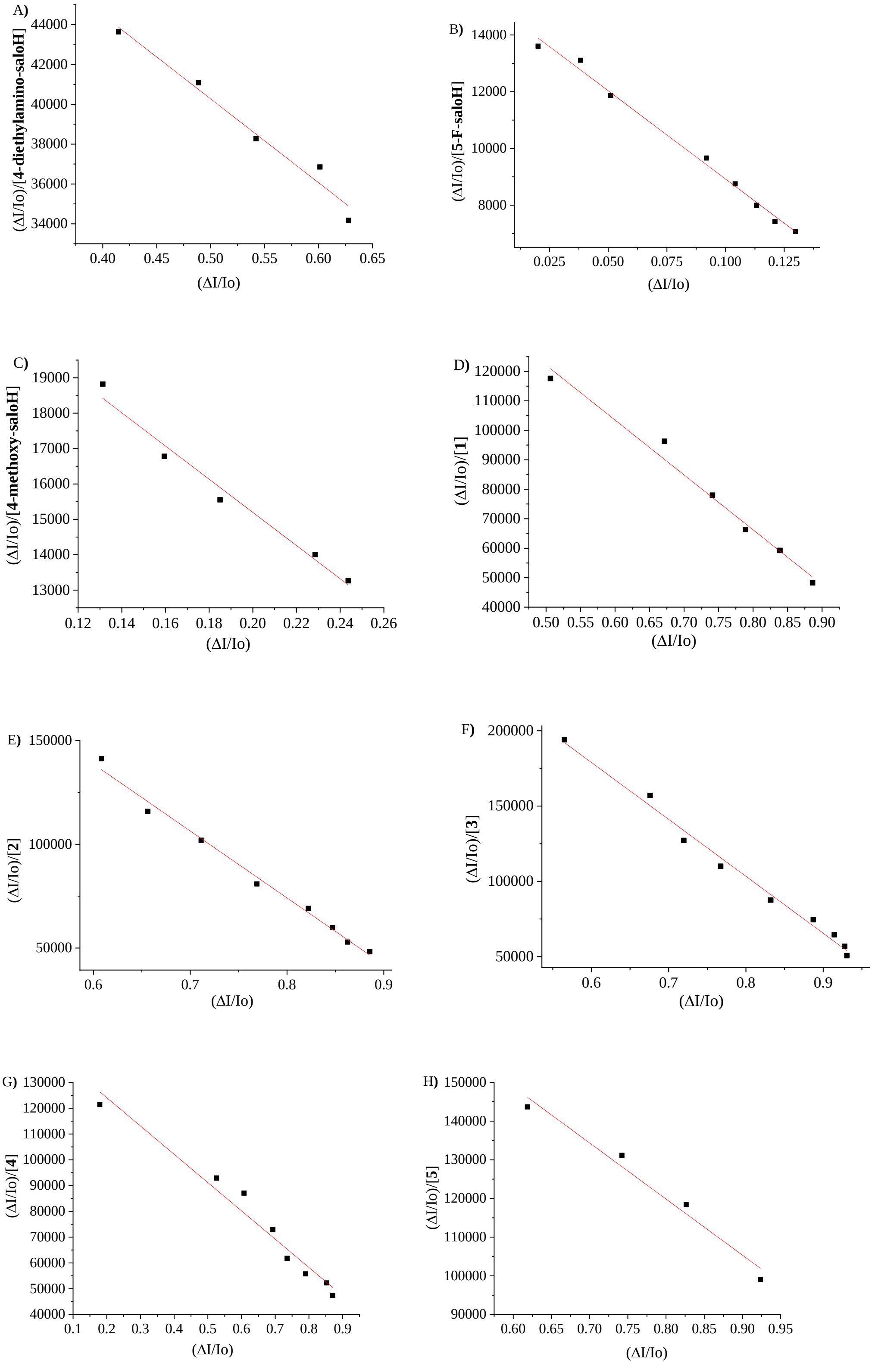

Figure S12. Scatchard plots of HSA–ibuprofen for (A)-(H) 4–Et_2N–saloH, 5–F–saloH, 4–MeO–saloH and complexes **1–5**, respectively.

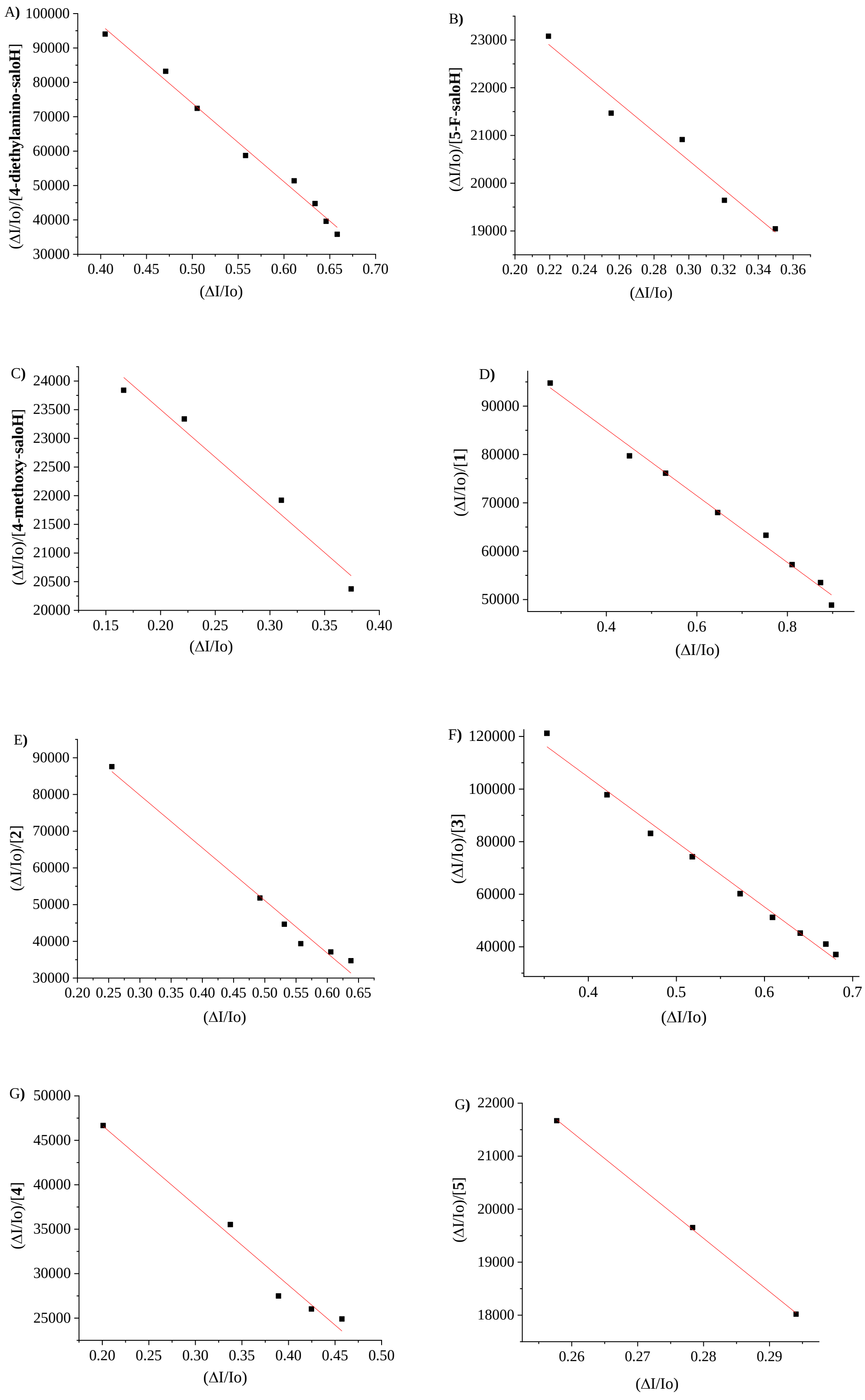

Figure S13. Scatchard plots of HSA–warfarin for (A)-(H) 4–Et_2N–saloH, 5–F–saloH, 4–MeO–saloH and complexes **1–5**, respectively.

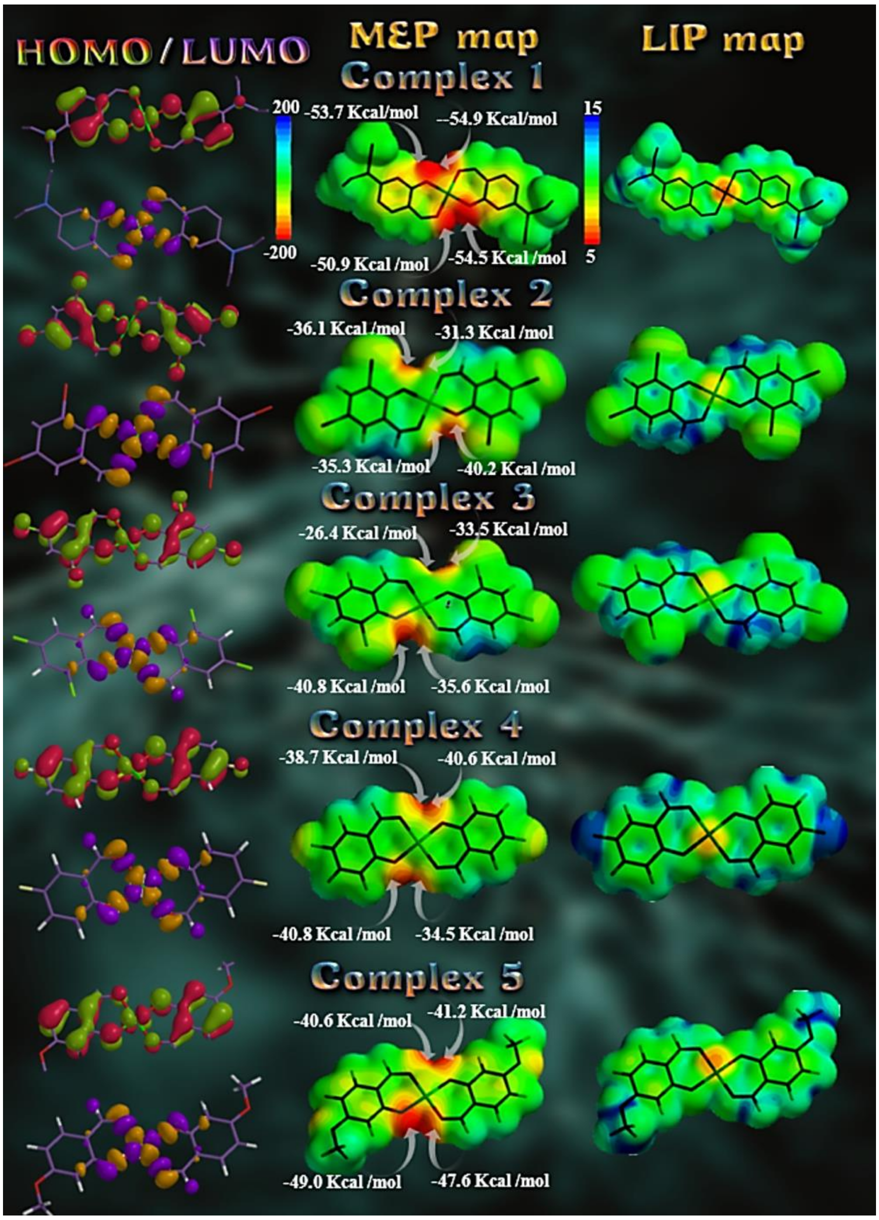

Figure S14. Depiction of computed frontier molecular orbitals HOMO and LUMO (solid surface) (left panel), MEP map (center panel, transparent surface), and LIP map (right panel, transparent surface) for complexes **1**–**5**. In MEP maps, a molecular electron density surface (isovalue = 0.002 e/bohr^3) generally encompassing at least 95% of the electronic charge, mapped with electrostatic potential (superimposed on the isodensity surface). The potential represents electron–rich (red, negative values) and electron–poor (blues, positive values) regions (intermediate values are colored orange, yellow and green) (potential ranges from –200 to 200 kJ/mol for MEP and 5–15 kJ/mol for LIP, shown in color scale). The exact MEP on phenolato and carboxylato oxygens on each molecule is also illustrated. The order of electron rich to poor regions is represented in rainbow colors as red < orange < yellow < green < blue. Colors toward red correspond to negative potential (stabilizing interaction between the molecule and a positive charge), while colors toward blue correspond to positive potential. LIP maps, representing an indicator of electrophilic addition, are an overlay of the energy of electron removal (ionization) on the electron density. By convention, red regions on a local ionization potential map indicate areas from which electron removal (ionization) is relatively easy, meaning that they are subject to electrophilic attack. These are easily distinguished from regions where ionization is relatively difficult (by convention, colored blue). The red and green color distributions represent positive and negative phase in molecular orbital wave function, respectively (isovalue = 0.032 au). HOMO (electron donor regions) determines the ionization potentials (assess the ionization energy), while LUMO (electron acceptor regions) determines the electron affinity.

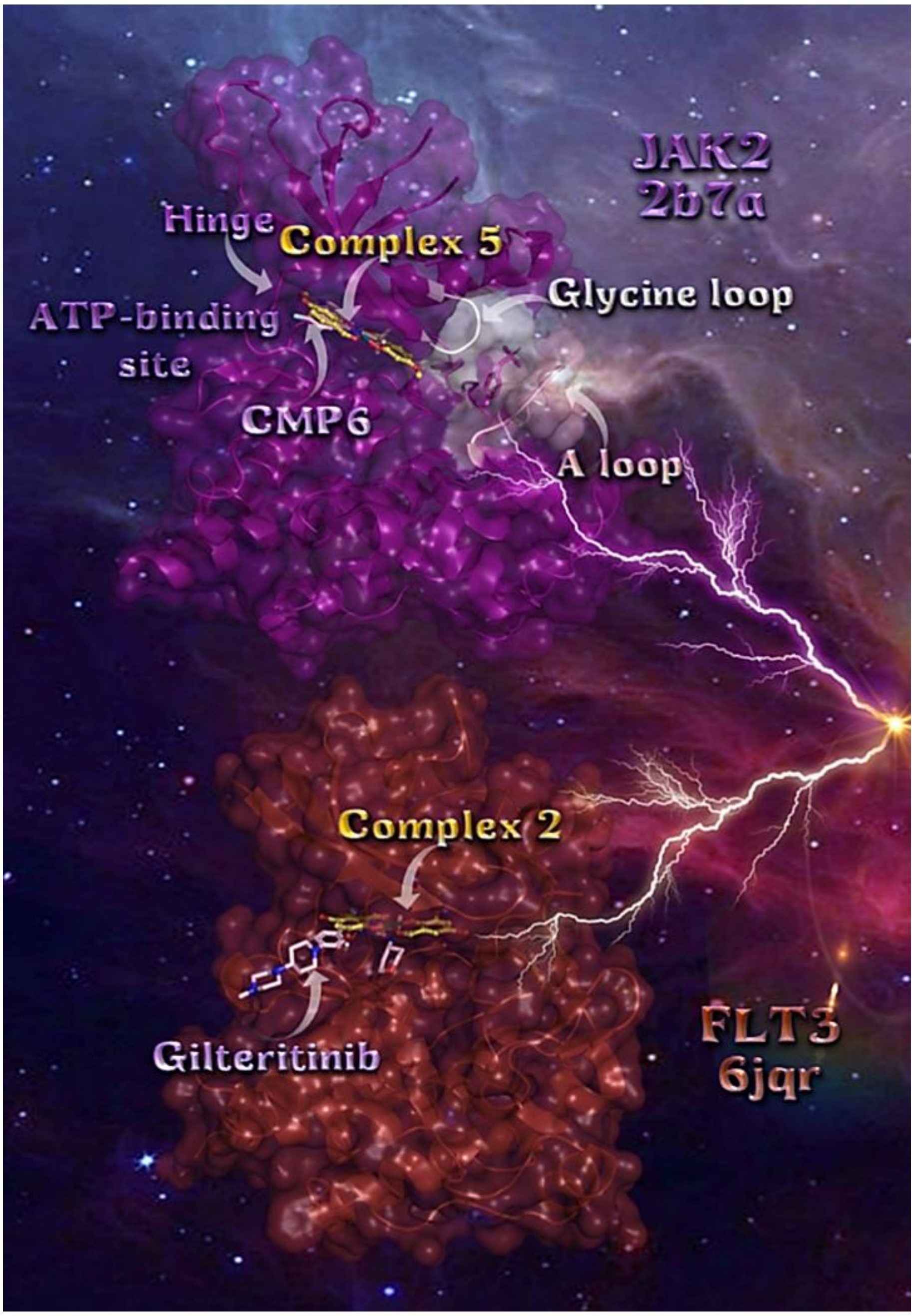

Figure S15. (Lower panel) Docking pose orientation of **2** on the crystal structure of the FLT3 kinase domain (PDB: 6jqr) bound with the FLT3 inhibitor Gilteritinib. Target protein is illustrated as cartoon–colored in ruby with depth cue in the ray–tracing rendering of the whole structure with additional depiction of semi–transparent surface colored according to cartoon. At the binding pocket of 2 in the crystal structure of FLT3 is also accommodated the Gilteritinib inhibitor. (Upper panel) Docking pose orientation of **5** on the crystal structure of JAK2 kinase domain (PDB: 2b7a) bound with its inhibitor CMP6. Target protein is illustrated as cartoon–colored in deep purple with depth cue in the ray–tracing rendering of the whole structure with additional depiction of semi–transparent surface colored according to cartoon. At the ATP–binding pocket of **5** in the crystal structure of JAK2 is also accommodated the CMP6 inhibitor. In both structures, complexes **2** and **5** are rendered in ball–and–stick mode colored according to atom type in yellow orange C atoms, while Gilteritinib and CMP6 inhibitors are rendered in stick model colored according to atom type in light pink. Heteroatom color–code: O: red, N: blue, Br: firebrick red, and Pd: deep teal. Hydrogen atoms are omitted from all molecules for shake of clarity. The final structure was ray–traced and illustrated with the aid of PyMol Molecular Graphics Systems.

Table S1. Crystallographic data, data collection and refinement details for complex **1.**

Crystal data	
Chemical formula	$C_{24}H_{31}N_3O_4Pd$
M_r	531.93
Crystal system	Monoclinic
Space group	$P2_1/n$
Temperature (K)	295
a (Å)	6.4774 (6)
b (Å)	8.7509 (8)
c (Å)	21.197 (2)
β (°)	98.350 (3)
V (Å^3)	1188.76 (19)
Z	2
Radiation type	Mo $K\alpha$
μ (mm^{-1})	0.82
Crystal size (mm)	0.18 × 0.16 × 0.05
Data collection	
Diffractometer	Bruker Kappa Apex2
Absorption correction	Numerical, Analytical Absorption (De Meulenaer & Tompa, 1965)
T_{min}, T_{max}	0.88, 0.96
No. of measured reflections	5412
No. of independent reflections	2246
No. of observed [I> 2.0σ(I)] reflections	1960
R_{int}	0.023
(sin θ/λ)$_{max}$ (Å^{-1})	0.612
Refinement	
$R[F^2> 2\sigma(F^2)]$	0.035
$wR(F^2)$	0.067
S	1.00
No. of reflections	1960
No. of parameters	145
No. of restraints	4
H–atom treatment	H–atom parameters constrained
$\Delta\rho_{max}$, $\Delta\rho_{min}$ (e Å^{-3})	0.56, −0.44

Table S2. Binding interactions of complex **1** with CT DNA (Atom numbering and bond lengths are derived from PyMol software).

Complex 1 moiety	CT DNA		
	**Pyrimidine/Purine**	**Bond length (Å)**	**Type[a]**
4–Et_2N–salo	dC11/C4	2.8	Hph
	**dC15/O2	3.8	π–polar
	dC11/C5	2.7	π–alkyl
	dG10/C4	2.7	π–alkyl
	dA18/OP1	3.0	P
	dG10/O3	3.3	π–polar
	*dC11/O2	3.5	P
	**dG10/N2	3.8	Hb
	dG10/N3	3.7	P
	***dG16/N2	3.8	Hb
	dA17/C5	3.5	π–alkyl
	*dG14/N2	3.9	π–polar
	dG14/N3	3.2	π–alkyl
	dC15/C2	3.9	Hph
	dG16/C4	3.0	π–alkyl
	dG16/C5	2.7	Hph
	dC11/OP1	3.4	π–polar
	dC11/O4	2.4	P

[a]type of interaction: Hb = hydrogen bond; Hph = hydrophobic; P = polar; π–polar =pi–polar; π–alkyl =pi–alkyl hydrophobic

Complex **1** interrupts interstrand Hb base paring dG14≡dC11 (*), dG10≡dC15 (**), and dG16≡dC9 (***)

Table S3. Binding interactions of lowest energy binding pose of complex **3** with *E. coli* and *S. aureus* DNA-gyrases (PDB accession numbers: 1KZN, and 5CDM, respectively). (Atom numbering and bond lengths are derived from PyMol software) (type of interaction: π–π: pi–pi sandwich, π–alkyl: pi–alkyl hydrophobic, π–cation and π–anion: pi–charged electrostatic interactions, π–polar: pi–polar, Hb: hydrogen bond, Hph: hydrophobic, P: polar, Halogen bond) (all binding contact residues are common with that of CBN).

Complex 3 moiety	*E. coli* DNA-gyrase amino acid residue	Bond length (Å)	Type
3,5–diCl–salo	Val43 (V43)/O	4.0	π–polar
	Val43 (V43)/O	3.5	Halogen bond
	Glu (E50)/Oε2	3.5	Hb
	Glu (E50)/Oε2	3.3	π–anion
	Asp (D73)/Oδ1	2.6	Halogen bond
	Asp (D73)/Oδ1	3.9	π–anion
	Arg (R76)/Nη1	3.7	π–cation
	Arg (R76)/Cζ	3.3	Cation–dipole halogen bond
	Gly (G77)/NH	3.3	Hb
	Gly (G77)/O	3.0	π–polar
	Ile (I78)/NH	3.5	Hb
	Ile (I78)/Cγ1	3.7	Hph
	Pro (P79)/Cδ	3.2	π–alkyl
	Pro (P79)/Cδ	3.5	Halogen bond
	Val(V120)/Cγ2	3.5	π–alkyl
	Arg (R76)/Nη1	2.8	π–cation
	Thr (T165)/Oγ1	3.4	Hb
	Thr (T165)/Cβ	3.6	π–alkyl
**Complex 3 moiety**	***S. aureus* DNA gyrase amino acid residue/ DNA Pyrimidine-Purine**	**Bond length (Å)**	**Type**
3,5–diCl–salo	Arg (R458)/Cβ	2.6	π–alkyl
	Arg (R458)/Nη1	3.7	Hb
	Glu (E477)/Oε1, ε2	3.8, 2.9	π–anion
	Glu (E477)/Oε2	2.7	Halogen bond
	PTR–123/O3P	3.7	π–polar
3,5–diCl–salo	dG'2009	3.3-3.5	π–π sandwich
	dG'2009/O	3.5	Halogen bond
	dG'2013/O4	3.8	Halogen bond
	dG'2009/O4	3.4	Halogen bond
	dG'2013/O4	2.9	π–polar
	dG'2013/N9	3.2	π–polar
	dG'2013/N3	3.2	P

Table S4. Binding interactions of lowest energy binding pose of complexes **1** with 5–LOX bound with NDGA (PDB accession number 6N2W), and **3** with FLAP protein bound with MK-591 (PDB ID: 2Q7M). (Atom numbering and bond lengths are derived from PyMol software) (type of interaction: Hb: hydrogen bond, Hph: hydrophobic, P: polar, π–alkyl: pi–alkyl hydrophobic, π–cation: pi–charged electrostatic interactions, π–polar: pi–polar) (binding contact residues denoted in asterisk are common with that of trolox).

**Complex 1 moiety**	**5-LOX bound with NDGA amino acid residue - 6N2W**	**Bond length (Å)**	**Type**
4–Et_2N–salo	Arg (R370)/Nη2	2.6	Hb
	Arg (R370)/Nη2	3.0	π–cation
	Arg (R370)/Nη1	2.3	Hb
	*Arg (R457)/Nη1	3.5	π–cation
	*Arg (R457)/Cδ	3.6	π–alkyl
	Phe (F450)	2.8	π–alkyl
	*Ser (S447)/O	3.3	P
	Leu (L448)/Cδ	2.7	Hph
	Asp (D442)/Oδ1	3.0	π–anion
	Asp (D442)/Cβ	3.5	π–alkyl
	Asp (D285)/O	3.0	P
	Leu (L288)/N	3.3	P
	Leu (L288)/Cβ	2.9	Hph
	Leu (L288)/Cδ1	3.0	Hph
**Complex 3 moiety**	**FLAP bound with MK591 - 2Q7M**	**Bond length (Å)**	**Type**
3,5–diCl–salo with α1 helix of chain E	Ala (A27)/Cβ	3.4	πvalkyl
	Gly (G24)/Cα	3.6	P
	Val (V20)/O	3.1	π–polar
	Val (V20)/Cγ1	3.2	π–alkyl
	Val (V21)/Cα	3.9	π–alkyl
3,5–diCl–salo with α2 helix of chain D	Asn (N59)/O	3.5	Hydrogen bond σ–hole
	Asp (D62)/Cβ	3.9	π–alkyl
	Asp (D62)/O	2.4	Halogen bond
	Thr (T66)/Cγ2	3.7	Hph
	Thr (T66)/Oγ1	2.7	Halogen bond
3,5–diCl–salo with α4 helix of chain D	Lys (K116)/Cε	3.5	P
	Lys (K116)/NH	3.6	Hb
	Phe (F123)	3.1	Halogen–π
3,5–diCl–salo with C2 loop connecting α3 to α4 helices of chain D	Tyr (Y112)	3.2	Halogen–π
	Tyr (Y112)/O	3.2	π–polar

Table S5. Calculated molecular physicochemical pharmacokinetics properties of complexes **1**–**5** related to Lipinski's Rule of Five with the aid of Molinspiration property engine v2018.10.

**Property**	**1**	**2**	**3**	**4**	**5**
**nON***	6	4	4	4	6
**nOHNH****	0	0	0	0	0
**miLog*P***	2.77	3.59	3.06	1.80	1.18
**MW**	505.01	> 500	505.54	398.74	422.81
**TPSA**	43.41	36.94	36.94	36.94	55.40
**natoms**	29	23	23	21	23
**nrotb**	6	0	0	0	0
**volume**	438.76	351.33	333.94	289.60	330.83
**Nviolations*****	1	1	1	0	0

*Hydrogen Bond Acceptors (HBA) (the sum of Os and Ns)

**Hydrogen Bond Donors (HBD)(the sum of OHs andNHs)

***Number of violations according to Lipinski's rule validation

Table S6. Determination of drug–likeness score of complexes **1**–**5** through molinspiration bioactivity score v2018.03.

**Properties**	**1**	**2**	**3**	**4**	**5**
GPCR	0.20	0.03	0.07	0.34	0.17
Ion channel modulator	0.09	–0.21	–0.05	0.25	0.17
Kinase inhibitor	–0.02	–0.25	–0.12	0.21	0.01
Nuclear receptor ligand	–0.03	0.11	–0.02	0.35	0.22
Protease inhibitor	0.23	0.04	0.07	0.53	0.30
Enzyme inhibitor	0.10	0.04	0.08	0.38	0.31

Table S7. Rat acute toxicity predicted by GUSAR (on the Basis of PASS Prediction) for complexes **1**–**5**.

**Acute rat toxicity prediction parameters**	**1**	**2**	**3**	**4**	**5**
	**Rat LD_{50} values**				
**Rat IP[a] LD_{50} (mg/kg)**	277,900 in AD	877,700 in AD	11,940 out of AD	289,400 in AD	946,500 in AD
**Rat IV[b] LD_{50} (mg/kg)**	77,030 in AD	85,770 in AD	64,290 in AD	77,660 in AD	94,720 in AD
**Rat Oral[c] LD_{50} (mg/kg)**	1509,000 in AD	2105,000 in AD	3613,000 out of AD	221,200 out of AD	2245,000 in AD
**Rat SC[d] LD_{50} (mg/kg)**	598,300 in AD	1096,000 in AD	1370,000 in AD	1510,000 in AD	2363,000 in AD
**Acute rodent toxicity classification of compounds by OECD project**					
**Rat IP LD_{50} Classification**	Class 4 in AD	Class 5 in AD	Class 3 out of AD	Class 4 in AD	Class 5in AD
**Rat IV LD_{50} Classification**	Class 4 in AD	Class 4 in AD	Class 4 in AD	Class 4 in AD	Class 4in AD
**Rat Oral LD_{50} Classification**	Class 4 in AD	Class 5 in AD	Class 5 out of AD	Class 3 out of AD	Class 5 in AD
**Rat SC LD_{50} Classification**	Class 4 in AD	Class 5 in AD	Class 5in AD	Class 5in AD	Class 5 in AD

[a] IP - Intraperitoneal route of administration
[b] IV –Intravenous route of administration
[c] Oral –Oral route of administration
[d] SC –Subcutaneous route of administration
in AD - compound falls in the applicability domain of the model
out of AD - compound is out of the applicability domain of the model

Table S8. Substrate/Metabolite specificity Prediction (SMP) for complexes **1**–**5**.

Complex 1

Pa > Pi

Substrate based prediction result

Pa	Pi	Enzyme
0.461	0.137	2A6
0.464	0.164	2C8
0.412	0.141	2B6
0.384	0.166	2E1
0.343	0.158	3A4
0.329	0.230	1A1
0.206	0.112	Aldoketoreductase
0.270	0.202	2C19
0.252	0.219	2D6

Metabolite based prediction result

Pa	Pi	Enzyme
0.497	0.113	2C19
0.452	0.107	UGT2B7
0.406	0.116	UGT2B4
0.368	0.111	UGT2B10
0.386	0.173	3A4
0.355	0.167	2D6
0.285	0.166	UGT2B17
0.209	0.128	Aldoketoreductase
0.249	0.225	2B6
0.246	0.231	2A6

Complex 2

Pa > Pi

Substrate based prediction result

Pa	Pi	Enzyme
0.529	0.102	2A6
0.515	0.099	2B6
0.261	0.064	Aldoketoreductase

0.381	0.245	2C8
0.334	0.223	2E1

Metabolite based prediction result

Pa	Pi	Enzyme
0.377	0.093	UGT2B10
0.276	0.071	Aldoketoreductase
0.318	0.172	2B6
0.325	0.200	UGT1A4
0.314	0.223	3A4
0.303	0.215	2D6
0.292	0.210	UGT2B4
0.270	0.191	UGT1A6
0.284	0.225	UGT2B7
0.227	0.193	2E1
0.234	0.232	UGT2B17
0.241	0.239	2A6

Complex 3

Pa > Pi

Substrate based prediction result

Pa	Pi	Enzyme
0.505	0.111	2E1
0.406	0.175	2A6
0.399	0.219	2C8
0.356	0.176	2B6
0.232	0.089	Aldoketoreductase

Metabolite based prediction result

Pa	Pi	Enzyme
0.390	0.073	UGT2B10
0.387	0.132	UGT2B4
0.248	0.091	Aldoketoreductase
0.324	0.202	UGT1A4
0.309	0.199	UGT2B7
0.295	0.186	2B6

0.280	0.190	2A6
0.268	0.187	UGT2B17
0.268	0.193	UGT1A6
0.239	0.182	2E1

Complex 4

Pa > Pi

Substrate based prediction result

Pa	Pi	Enzyme
0.425	0.119	3A4
0.267	0.205	2C19

Metabolite based prediction result

Pa	Pi	Enzyme
0.457	0.134	2C19
0.450	0.141	3A4
0.433	0.201	2C9
0.315	0.196	UGT1A9
0.315	0.217	UGT2B10

Complex 5

Pa > Pi

Substrate based prediction result

Pa	Pi	Enzyme
0.431	0.188	2C8
0.373	0.138	3A4
0.230	0.091	Aldoketoreductase
0.310	0.222	2B6
0.289	0.266	2E1
0.304	0.282	2A6

Metabolite based prediction result

Pa	Pi	Enzyme
0.400	0.062	UGT2B10

0.431	0.098	UGT2B4
0.343	0.118	UGT2B17
0.376	0.179	3A4
0.254	0.087	Aldoketoreductase
0.295	0.168	2C8
0.314	0.194	UGT2B7
0.246	0.164	UGT2B15
0.287	0.252	UGT1A4
0.238	0.222	UGT1A6

Table S9. ROSC–Pred: Rodent Organ–Specific Carcinogenicity Prediction for complexes **1**–**5**.

Complex 1

Pa > Pi

The structure is out of the applicability domain.Percentage of new MNA descriptors: **35.5 %**

Rats

Male			Female		
Pa	Pi	Organs	Pa	Pi	Organs
0.528	0.059	vascular system	0.520	0.237	uterus
0.526	0.155	liver	0.389	0.112	vascular system
0.413	0.186	all tumor bearing animals	0.453	0.353	hematopoietic system
0.459	0.276	hematopoietic system	0.272	0.258	nasal cavity
0.352	0.254	nasal cavity			
0.383	0.339	thyroid gland			
0.168	0.160	esophagus			

Mice

Male			Female		
Pa	Pi	Organs	Pa	Pi	Organs
0.396	0.301	lung	0.460	0.090	pituitary gland

Complex 2

The structure is out of the applicability domain.Percentage of new MNA descriptors: **56 %**

Rats

Male			Female		
Pa	Pi	Organs	Pa	Pi	Organs
0.765	0.043	stomach	0.807	0.051	stomach
0.777	0.127	kidney	0.621	0.063	kidney
0.659	0.073	hematopoietic system	0.471	0.092	vascular system
0.549	0.106	oral cavity	0.450	0.078	nasal cavity
0.482	0.059	large intestine	0.560	0.228	hematopoietic system
0.508	0.099	nasal cavity	0.527	0.223	uterus
0.460	0.083	vascular system	0.433	0.190	liver
0.529	0.164	thyroid gland	0.291	0.086	oral cavity
0.520	0.160	liver	0.362	0.246	thyroid gland
0.273	0.160	lung	0.292	0.186	mammary gland
			0.309	0.251	lung

Mice

Male			Female		
Pa	Pi	Organs	Pa	Pi	Organs
0.904	0.006	stomach	0.730	0.013	lung

0.717	0.028	kidney	0.753	0.041	stomach
0.627	0.059	lung	0.566	0.027	pituitary gland
0.383	0.238	vascular system	0.576	0.145	hematopoietic system
0.117	0.018	small intestine	0.216	0.079	peritoneal cavity
			0.361	0.232	ovary
			0.381	0.262	liver
			0.117	0.018	small intestine
			0.399	0.382	urinary bladder

Complex 3

The structure is out of the applicability domain.Percentage of new MNA descriptors: **44 %**

Rats					
Male			Female		
Pa	Pi	Organs	Pa	Pi	Organs
0.629	0.072	liver	0.272	0.150	vascular system
0.501	0.230	hematopoietic system	0.451	0.364	uterus
0.365	0.153	vascular system	0.437	0.373	hematopoietic system
0.454	0.370	kidney	0.332	0.305	stomach
0.313	0.272	oral cavity			
0.368	0.358	thyroid gland			

Mice					
Male			Female		
Pa	Pi	Organs	Pa	Pi	Organs
0.901	0.015	liver	0.904	0.020	liver
0.525	0.195	stomach	0.643	0.038	lung
0.099	0.037	small intestine	0.478	0.069	pituitary gland
			0.473	0.176	stomach
			0.183	0.111	peritoneal cavity
			0.099	0.037	small intestine

Complex 4

The structure is out of the applicability domain.Percentage of new MNA descriptors: **56 %**

Rats					
Male			Female		
Pa	Pi	Organs	Pa	Pi	Organs
0.598	0.124	hematopoietic system	0.471	0.092	vascular system
0.460	0.083	vascular system	0.560	0.228	hematopoietic system
0.499	0.196	thyroid gland	0.527	0.223	uterus
0.574	0.299	kidney	0.450	0.251	kidney
0.475	0.212	liver	0.371	0.176	lung
0.420	0.175	nasal cavity	0.390	0.234	liver
0.393	0.237	stomach	0.323	0.171	nasal cavity
0.269	0.168	lung	0.376	0.260	stomach

0.301	0.287	oral cavity

Mice

Male			Female		
Pa	Pi	Organs	Pa	Pi	Organs
0.628	0.138	stomach	0.660	0.100	stomach
0.475	0.205	lung	0.566	0.027	pituitary gland
0.383	0.238	vascular system	0.542	0.171	hematopoietic system
0.117	0.018	small intestine	0.216	0.079	peritoneal cavity
			0.361	0.232	ovary
			0.402	0.279	lung
			0.117	0.018	small intestine
			0.399	0.382	urinary bladder

Complex 5

The structure is out of the applicability domain.Percentage of new MNA descriptors: **29.6 %**

Rats

Male			Female		
Pa	Pi	Organs	Pa	Pi	Organs
0.382	0.135	vascular system	0.243	0.163	vascular system
0.466	0.269	hematopoietic system	0.439	0.370	hematopoietic system
0.429	0.267	liver	0.269	0.263	nasal cavity

Mice

Male			Female		
Pa	Pi	Organs	Pa	Pi	Organs
0.508	0.207	stomach	0.491	0.221	hematopoietic system
0.365	0.348	lung	0.408	0.149	pituitary gland

Table S10. Quantitative prediction of anti–target interaction profiles for complexes**1**–**5**.

Quantitative prediction of anti–target interaction profiles for complex **1**

**Activity**	**Prediction Value, -Log10(Value), Mole**	**Applicability Domain**
5-hydroxytryptamine 1B receptor antagonist IC50	5,885	Out of AD
5-hydroxytryptamine 1B receptor antagonist Ki	6,831	In AD
5-hydroxytryptamine 2A receptor antagonist IC50	7,379	Out of AD
5-hydroxytryptamine 2A receptor antagonist Ki	6,364	Out of AD
5-hydroxytryptamine 2C receptor antagonist IC50	6,469	In AD
5-hydroxytryptamine 2C receptor antagonist Ki	6,890	Out of AD
alpha1a adrenergic receptor antagonist IC50	6,031	Out of AD
alpha1a adrenergic receptor antagonist Ki	6,755	Out of AD
alpha1b adrenergic receptor antagonist Ki	6,698	Out of AD
Alpha-2A adrenergic receptor antagonist IC50	6,033	In AD
Alpha-2A adrenergic receptor antagonist Ki	6,180	In AD
amine oxidase [flavin-containing] A inhibitor IC50	5,456	In AD
amine oxidase [flavin-containing] A inhibitor Ki	5,519	In AD
androgen receptor antagonist IC50	5,515	In AD
carbonic anhydrase I activator Kact	8,372	In AD
carbonic anhydrase I inhibitor Ki	7,359	In AD
Carbonic anhydrase 2 activator Kact	8,713	In AD
carbonic anhydrase II inhibitor Ki	7,707	Out of AD
D(1A) dopamine receptor antagonist IC50	4,772	In AD
D(1A) dopamine receptor antagonist Ki	5,692	In AD
D3 dopamine receptor antagonist Ki	6,387	Out of AD
delta-type opioid receptor antagonist Ki	5,951	Out of AD
estrogen receptor antagonist IC50	6,171	In AD
estrogen receptor antagonist Ki	6,669	In AD
kappa-type opioid receptor antagonist Ki	5,483	Out of AD
mu-type opioid receptor antagonist IC50	5,354	In AD
mu-type opioid receptor antagonist Ki	5,456	In AD
sodium- and chloride-dependent GABA transporter 1 antagonist IC50	4,454	In AD
sodium-dependent dopamine transporter antagonist IC50	5,470	Out of AD
sodium-dependent dopamine transporter antagonist Ki	5,927	In AD
sodium-dependent serotonin transporter antagonist IC50	5,279	Out of AD
sodium-dependent serotonin transporter antagonist Ki	6,223	Out of AD

The total number of anti–target(s): 8
in AD – compound falls in the applicability domain of the model
out of AD –compound is out of the applicability domain of the model

Quantitative prediction of anti-target interaction profiles for chemical complex **2**

**Activity**	**Prediction Value, -Log10(Value), Mole**	**Applicability Domain**
5-hydroxytryptamine 1B receptor antagonist IC50	4,752	Out of AD
5-hydroxytryptamine 1B receptor antagonist Ki	6,073	Out of AD
5-hydroxytryptamine 2A receptor antagonist IC50	7,070	Out of AD
5-hydroxytryptamine 2A receptor antagonist Ki	8,043	Out of AD
5-hydroxytryptamine 2C receptor antagonist IC50	7,272	In AD
5-hydroxytryptamine 2C receptor antagonist Ki	7,388	Out of AD
alpha1a adrenergic receptor antagonist IC50	5,423	Out of AD
alpha1a adrenergic receptor antagonist Ki	5,766	Out of AD
alpha1b adrenergic receptor antagonist Ki	6,597	Out of AD
Alpha-2A adrenergic receptor antagonist IC50	5,319	In AD
Alpha-2A adrenergic receptor antagonist Ki	6,141	Out of AD
amine oxidase [flavin-containing] A inhibitor IC50	5,706	In AD
amine oxidase [flavin-containing] A inhibitor Ki	4,629	In AD
androgen receptor antagonist IC50	5,792	In AD
carbonic anhydrase I activator Kact	8,600	In AD
carbonic anhydrase I inhibitor Ki	7,570	Out of AD
Carbonic anhydrase 2 activator Kact	8,985	In AD
carbonic anhydrase II inhibitor Ki	8,950	Out of AD
D(1A) dopamine receptor antagonist IC50	5,593	In AD
D(1A) dopamine receptor antagonist Ki	6,319	In AD
D3 dopamine receptor antagonist Ki	5,337	Out of AD
delta-type opioid receptor antagonist Ki	5,921	Out of AD
estrogen receptor antagonist IC50	6,077	In AD
estrogen receptor antagonist Ki	6,495	In AD
kappa-type opioid receptor antagonist Ki	5,192	Out of AD
mu-type opioid receptor antagonist IC50	6,073	Out of AD
mu-type opioid receptor antagonist Ki	6,623	Out of AD
sodium- and chloride-dependent GABA transporter 1 antagonist IC50	4,084	Out of AD
sodium-dependent dopamine transporter antagonist IC50	6,221	Out of AD
sodium-dependent dopamine transporter antagonist Ki	5,592	Out of AD
sodium-dependent serotonin transporter antagonist IC50	6,470	Out of AD
sodium-dependent serotonin transporter antagonist Ki	6,129	Out of AD

The total number of anti–target(s): 6
in AD –compound falls in the applicability domain of the model
out of AD – compound is out of the applicability domain of the model

Quantitative prediction of anti–target interaction profiles for chemical complex **3**

Activity	Prediction Value, -Log10(Value), Mole	Applicability Domain
5-hydroxytryptamine 1B receptor antagonist IC50	5,498	Out of AD
5-hydroxytryptamine 1B receptor antagonist Ki	6,635	In AD
5-hydroxytryptamine 2A receptor antagonist IC50	7,011	Out of AD
5-hydroxytryptamine 2A receptor antagonist Ki	6,637	Out of AD
5-hydroxytryptamine 2C receptor antagonist IC50	7,394	In AD
5-hydroxytryptamine 2C receptor antagonist Ki	6,946	Out of AD
alpha1a adrenergic receptor antagonist IC50	5,659	Out of AD
alpha1a adrenergic receptor antagonist Ki	5,886	Out of AD
alpha1b adrenergic receptor antagonist Ki	6,998	Out of AD
Alpha-2A adrenergic receptor antagonist IC50	5,608	Out of AD
Alpha-2A adrenergic receptor antagonist Ki	6,058	In AD
amine oxidase [flavin-containing] A inhibitor IC50	5,719	In AD
amine oxidase [flavin-containing] A inhibitor Ki	5,515	In AD
androgen receptor antagonist IC50	5,686	In AD
carbonic anhydrase I activator Kact	6,717	In AD
carbonic anhydrase I inhibitor Ki	7,197	Out of AD
Carbonic anhydrase 2 activator Kact	8,809	In AD
carbonic anhydrase II inhibitor Ki	8,728	Out of AD
D(1A) dopamine receptor antagonist IC50	5,122	In AD
D(1A) dopamine receptor antagonist Ki	6,618	In AD
D3 dopamine receptor antagonist Ki	6,016	Out of AD
delta-type opioid receptor antagonist Ki	5,524	Out of AD
estrogen receptor antagonist IC50	5,862	In AD
estrogen receptor antagonist Ki	5,697	In AD
kappa-type opioid receptor antagonist Ki	5,976	Out of AD
mu-type opioid receptor antagonist IC50	4,855	Out of AD
mu-type opioid receptor antagonist Ki	5,934	Out of AD
sodium- and chloride-dependent GABA transporter 1 antagonist IC50	4,196	In AD
sodium-dependent dopamine transporter antagonist IC50	6,318	Out of AD
sodium-dependent dopamine transporter antagonist Ki	6,185	Out of AD
sodium-dependent serotonin transporter antagonist IC50	5,480	Out of AD
sodium-dependent serotonin transporter antagonist Ki	6,222	Out of AD

The total number of anti–target(s): 8

in AD – compound falls in the applicability domain of the model

out of AD – compound is out of the applicability domain of the model

Quantitative prediction of anti–target interaction profiles for chemical complex **4**

Activity	Prediction Value, -Log10(Value), Mole	Applicability Domain
5-hydroxytryptamine 1B receptor antagonist IC50	5,711	Out of AD
5-hydroxytryptamine 1B receptor antagonist Ki	5,911	In AD
5-hydroxytryptamine 2A receptor antagonist IC50	7,301	Out of AD
5-hydroxytryptamine 2A receptor antagonist Ki	6,965	In AD
5-hydroxytryptamine 2C receptor antagonist IC50	7,214	In AD
5-hydroxytryptamine 2C receptor antagonist Ki	6,703	Out of AD
alpha1a adrenergic receptor antagonist IC50	5,885	Out of AD
alpha1a adrenergic receptor antagonist Ki	5,629	Out of AD
alpha1b adrenergic receptor antagonist Ki	7,085	Out of AD
Alpha-2A adrenergic receptor antagonist IC50	5,451	In AD
Alpha-2A adrenergic receptor antagonist Ki	5,995	In AD
amine oxidase [flavin-containing] A inhibitor IC50	5,310	In AD
amine oxidase [flavin-containing] A inhibitor Ki	5,727	In AD
androgen receptor antagonist IC50	6,050	Out of AD
carbonic anhydrase I activator Kact	8,431	In AD
carbonic anhydrase I inhibitor Ki	7,962	Out of AD
Carbonic anhydrase 2 activator Kact	8,896	In AD
carbonic anhydrase II inhibitor Ki	8,220	Out of AD
D(1A) dopamine receptor antagonist IC50	5,293	In AD
D(1A) dopamine receptor antagonist Ki	6,704	In AD
D3 dopamine receptor antagonist Ki	5,900	Out of AD
delta-type opioid receptor antagonist Ki	5,441	Out of AD
estrogen receptor antagonist IC50	5,555	In AD
estrogen receptor antagonist Ki	6,123	In AD
kappa-type opioid receptor antagonist Ki	5,600	Out of AD
mu-type opioid receptor antagonist IC50	5,044	In AD
mu-type opioid receptor antagonist Ki	6,038	Out of AD
sodium- and chloride-dependent GABA transporter 1 antagonist IC50	4,637	In AD
sodium-dependent dopamine transporter antagonist IC50	5,629	Out of AD
sodium-dependent dopamine transporter antagonist Ki	6,919	Out of AD
sodium-dependent serotonin transporter antagonist IC50	5,586	Out of AD
sodium-dependent serotonin transporter antagonist Ki	7,099	Out of AD

The total number of anti–target(s): 7

in AD – compound falls in the applicability domain of the model

out of AD – compound is out of the applicability domain of the model

Quantitative prediction of anti–target interaction profiles for chemical complex **5**

Activity	Prediction Value, -Log10(Value), Mole	Applicability Domain
5-hydroxytryptamine 1B receptor antagonist IC50	4,531	Out of AD
5-hydroxytryptamine 1B receptor antagonist Ki	6,274	In AD
5-hydroxytryptamine 2A receptor antagonist IC50	6,801	Out of AD
5-hydroxytryptamine 2A receptor antagonist Ki	5,995	In AD
5-hydroxytryptamine 2C receptor antagonist IC50	7,191	In AD
5-hydroxytryptamine 2C receptor antagonist Ki	6,325	Out of AD
alpha1a adrenergic receptor antagonist IC50	6,131	Out of AD
alpha1a adrenergic receptor antagonist Ki	6,698	Out of AD
alpha1b adrenergic receptor antagonist Ki	7,103	Out of AD
Alpha-2A adrenergic receptor antagonist IC50	5,845	In AD
Alpha-2A adrenergic receptor antagonist Ki	5,949	In AD
amine oxidase [flavin-containing] A inhibitor IC50	5,212	In AD
amine oxidase [flavin-containing] A inhibitor Ki	6,591	In AD
androgen receptor antagonist IC50	6,040	Out of AD
carbonic anhydrase I activator Kact	5,491	Out of AD
carbonic anhydrase I inhibitor Ki	8,029	Out of AD
Carbonic anhydrase 2 activator Kact	7,539	In AD
carbonic anhydrase II inhibitor Ki	7,769	Out of AD
D(1A) dopamine receptor antagonist IC50	4,978	In AD
D(1A) dopamine receptor antagonist Ki	6,257	In AD
D3 dopamine receptor antagonist Ki	5,998	In AD
delta-type opioid receptor antagonist Ki	5,432	Out of AD
estrogen receptor antagonist IC50	5,703	In AD
estrogen receptor antagonist Ki	8,188	In AD
kappa-type opioid receptor antagonist Ki	5,929	Out of AD
mu-type opioid receptor antagonist IC50	4,777	In AD
mu-type opioid receptor antagonist Ki	6,191	Out of AD
sodium- and chloride-dependent GABA transporter 1 antagonist IC50	4,603	In AD
sodium-dependent dopamine transporter antagonist IC50	6,015	Out of AD
sodium-dependent dopamine transporter antagonist Ki	5,647	Out of AD
sodium-dependent serotonin transporter antagonist IC50	5,615	Out of AD
sodium-dependent serotonin transporter antagonist Ki	5,789	Out of AD

The total number of anti–target(s): 8

in AD – compound falls in the applicability domain of the model

out of AD – compound is out of the applicability domain of the model

Table S11. Environmental ecotoxicity prediction for complexes **1**–**5** by GUSAR.

**Complex 1**		
**Activity**	**Prediction Value**	**Applicability Domain**
Bioaccumulation factor Log10(BCF)	1,172	In AD
Daphnia magna LC50 -Log10(mol/L)	5,091	In AD
Fathead Minnow LC50 Log10(mmol/L)	-3,487	In AD
Tetrahymena pyriformis IGC50 -Log10(mol/L)	1,265	In AD
**Complex 2**		
**Activity**	**Prediction Value**	**Applicability Domain**
Bioaccumulation factor Log10(BCF)	1,568	In AD
Daphnia magna LC50 -Log10(mol/L)	5,274	In AD
Fathead Minnow LC50 Log10(mmol/L)	-3,857	In AD
Tetrahymena pyriformis IGC50 -Log10(mol/L)	1,790	In AD
**Complex 3**		
**Activity**	**Prediction Value**	**Applicability Domain**
Bioaccumulation factor Log10(BCF)	2,231	In AD
Daphnia magna LC50 -Log10(mol/L)	5,084	In AD
Fathead Minnow LC50 Log10(mmol/L)	-3,606	In AD
Tetrahymena pyriformis IGC50 -Log10(mol/L)	1,985	In AD
**Complex 4**		
**Activity**	**Prediction Value**	**Applicability Domain**
Bioaccumulation factor Log10(BCF)	0,000	0,853
Daphnia magna LC50 -Log10(mol/L)	0,000	0,853
Fathead Minnow LC50 Log10(mmol/L)	0,000	0,853
Tetrahymena pyriformis IGC50 -Log10(mol/L)	0,000	0,853
**Complex 5**		
**Activity**	**Prediction Value**	**Applicability Domain**
Bioaccumulation factor Log10(BCF)	1,095	In AD
Daphnia magna LC50 -Log10(mol/L)	4,243	In AD
Fathead Minnow LC50 Log10(mmol/L)	-2,169	In AD
Tetrahymena pyriformis IGC50 -Log10(mol/L)	0,869	In AD

in AD – compound falls in the applicability domain of the model
out of AD – compound is out of the applicability domain of the model

Table S12. CLC–Pred: *in silico* prediction of cytotoxicity for tumor and non-tumor cell lines (results for Pa > 0.3, Pa > 0.5, and Pa > Pi).Pa: probability "to be active", Pi: probability "to be inactive".

Complex 1

Cancer cell line prediction result

Pa > 0.3

Pa	Pi	Cell-line	Cell-line full name	Tissue	Tumor type
0.474	0.027	HepG2	Hepatoblastoma	Liver	Hepatoblastoma
0.475	0.037	HT-29	Colon adenocarcinoma	Colon	Adenocarcinoma
0.474	0.070	Hs 683	Oligodendroglioma	Brain	Glioma
0.464	0.073	NCI-H838	Non-small cell lung cancer. 3 stage	Lung	Carcinoma
0.360	0.043	HOP-18	Non-small cell lung carcinoma	Lung	Carcinoma
0.321	0.034	MKN-7	Gastric carcinoma	Stomach	Carcinoma
0.402	0.132	DMS-114	Lung carcinoma	Lung	Carcinoma
0.387	0.122	MDA-MB-453	Breast adenocarcinoma	Breast	Adenocarcinoma
0.319	0.075	T98G	Glioblastoma	Brain	Carcinoma
0.307	0.076	8505C	Thyroid gland undifferentiated (anaplastic) carcinoma	Thyroid	Carcinoma
0.306	0.275	SK-MEL-1	Metastatic melanoma	Skin	Melanoma

Pa > Pi

Pa	Pi	Cell-line	Cell-line full name	Tissue	Tumor type
0.474	0.027	HepG2	Hepatoblastoma	Liver	Hepatoblastoma
0.475	0.037	HT-29	Colon adenocarcinoma	Colon	Adenocarcinoma
0.474	0.070	Hs 683	Oligodendroglioma	Brain	Glioma
0.464	0.073	NCI-H838	Non-small cell lung cancer. 3 stage	Lung	Carcinoma
0.360	0.043	HOP-18	Non-small cell lung carcinoma	Lung	Carcinoma
0.321	0.034	MKN-7	Gastric carcinoma	Stomach	Carcinoma
0.402	0.132	DMS-114	Lung carcinoma	Lung	Carcinoma
0.387	0.122	MDA-MB-453	Breast adenocarcinoma	Breast	Adenocarcinoma
0.319	0.075	T98G	Glioblastoma	Brain	Carcinoma
0.307	0.076	8505C	Thyroid gland undifferentiated (anaplastic) carcinoma	Thyroid	Carcinoma
0.222	0.011	U-937/GTB	Histiocytic lymphoma	Blood	Lymphoma
0.204	0.004	SK-HEP1	Hepatocellular carcinoma	Liver	Carcinoma
0.277	0.094	H9	T-lymphoid	Haematopoietic and lymphoid tissue	Leukemia
0.278	0.107	HOS	Osteosarcoma	Bone	Sarcoma
0.173	0.003	MEXF276L	Xenograft melanoma	Skin	Melanoma
0.173	0.003	MEXF989	Xenograft melanoma	Skin	Melanoma
0.173	0.003	PAXF546	Pancreatic carcinoma	Pancreas	Carcinoma
0.190	0.045	ADR5000	Childhood T acute lymphoblastic leukemia	Blood	Leukemia

0.225	0.111	MDA-MB-361	Breast adenocarcinoma	Breast	Adenocarcinoma
0.247	0.141	PC-6	Small cell lung carcinoma	Lung	Carcinoma
0.096	0.003	H322	Lung carcinoma	Lung	Carcinoma
0.150	0.069	DLD-1	Colon adenocarcinoma	Colon	Adenocarcinoma
0.184	0.108	St-4	Stomach carcinoma	Stomach	Carcinoma
0.207	0.133	Raji	B-lymphoblastic cells	Haematopoietic and lymphoid tissue	Leukemia
0.204	0.132	M19-MEL	Melanoma	Skin	Melanoma
0.159	0.089	SHP77	Small cell lung carcinoma	Lung	Carcinoma
0.271	0.203	A2058	Melanoma	Skin	Melanoma
0.168	0.101	Caco-2	Colon adenocarcinoma	Colon	Adenocarcinoma
0.084	0.019	UMSCC22B	Hypopharyngeal squamous cell carcinoma	Upper aerodigestive tract	Carcinoma
0.104	0.044	SQ20B	Head and neck Squamous carcinoma	Head and neck	Carcinoma
0.065	0.006	UCLA P-3	Lung carcinoma cell line	Lung	Carcinoma
0.082	0.028	FaDu	Hypopharyngeal squamous carcinoma	Upper aerodigestive tract	Carcinoma
0.065	0.019	UMUC3	Bladder Carcinoma	Urinary tract	Carcinoma
0.132	0.089	Caov-3	High grade ovarian serous adenocarcinoma	Ovarium	Adenocarcinoma
0.277	0.245	CFPAC-1	Pancreatic carcinoma	Pancreas	Carcinoma
0.306	0.275	SK-MEL-1	Metastatic melanoma	Skin	Melanoma
0.096	0.064	COLO 320	Colon adenocarcinoma	Colon	Adenocarcinoma
0.185	0.154	HeLa	Cervical adenocarcinoma	Cervix	Adenocarcinoma
0.027	0.003	OVXF1023	Ovarian adenocarcinoma	Ovarium	Adenocarcinoma
0.027	0.003	OVXF1353	Ovarian adenocarcinoma	Ovarium	Adenocarcinoma
0.027	0.003	RXF 423	Renal carcinoma	Kidney	Carcinoma
0.055	0.032	Leukemia cells	Leukemia	Blood	Leukemia
0.224	0.213	NCI-H1299	Non-small cell lung carcinoma	Lung	Carcinoma
0.015	0.004	SW-60	Colorectal carcinoma	Colon	Carcinoma
0.286	0.285	NALM-6	Adult B acute lymphoblastic leukemia	Haematopoietic and lymphoid tissue	Leukemia
0.020	0.019	NT2	Embryonal carcinoma	Germ cell. fibroblast	Carcinoma
0.017	0.016	NCH82	Glioblastoma	Brain	Glioblastoma

Non-tumor cell line prediction result

Pa > 0.3

Pa	Pi	Cell-line	Cell-line full name	Tissue
0.395	0.036	WI-38 VA13	Embryonic lung fibroblast	Lung

Pa > Pi

Pa	Pi	Cell-line	Cell-line full name	Tissue
0.395	0.036	WI-38 VA13	Embryonic lung fibroblast	Lung

0.277	0.008	NHDF	Fibroblast	Skin
0.223	0.004	IMR-90	Embryonic lung fibroblast	Lung
0.271	0.069	MRC5	Embryonic lung fibroblast	Lung
0.188	0.024	PrEC	Prostate epithelial cell	Prostate
0.110	0.058	WI-38	Embryonic lung fibroblast	Lung
0.029	0.009	HASMC	Aortic smooth muscle	Muscle
0.090	0.089	HUVEC	Umbilical vein endothelial cell	Endothelium

Complex 2

Cancer cell line prediction result

Pa > 0.5

Pa	Pi	Cell-line	Cell-line full name	Tissue	Tumor type
0.603	0.025	Hs 683	Oligodendroglioma	Brain	Glioma

Pa > Pi

Pa	Pi	Cell-line	Cell-line full name	Tissue	Tumor type
0.603	0.025	Hs 683	Oligodendroglioma	Brain	Glioma
0.404	0.029	HOP-18	Non-small cell lung carcinoma	Lung	Carcinoma
0.338	0.026	MKN-7	Gastric carcinoma	Stomach	Carcinoma
0.346	0.037	SF-295	Glioblastoma	Brain	Glioblastoma
0.268	0.028	Jurkat	Acute leukemic T-cells	Blood	Leukemia
0.381	0.152	NCI-H838	Non-small cell lung cancer. 3 stage	Lung	Carcinoma
0.178	0.003	MEXF276L	Xenograft melanoma	Skin	Melanoma
0.178	0.003	MEXF989	Xenograft melanoma	Skin	Melanoma
0.178	0.003	PAXF546	Pancreatic carcinoma	Pancreas	Carcinoma
0.173	0.005	MOLT-3	T-lymphoblastic leukemia	Blood	Leukemia
0.167	0.016	COLO 320	Colon adenocarcinoma	Colon	Adenocarcinoma
0.194	0.043	ADR5000	Childhood T acute lymphoblastic leukemia	Blood	Leukemia
0.320	0.176	NCI-H187	Small cell lung carcinoma	Lung	Carcinoma
0.206	0.072	St-4	Stomach carcinoma	Stomach	Carcinoma
0.139	0.007	Bcap37	Breast adenocarcinoma	Breast	Adenocarcinoma
0.255	0.132	PC-6	Small cell lung carcinoma	Lung	Carcinoma
0.146	0.030	HCT-8	Ileocecal adenocarcinoma	Large intestine	Adenocarcinoma
0.114	0.009	UMSCC22B	Hypopharyngeal squamous cell carcinoma	Upper aerodigestive tract	Carcinoma
0.209	0.132	HL-60	Promyeloblast leukemia	Haematopoietic and lymphoid tissue	Leukemia
0.111	0.050	MES-SA	Uterine corpus sarcoma	Uterus	Sarcoma
0.063	0.004	H322	Lung carcinoma	Lung	Carcinoma
0.063	0.007	UCLA P-3	Lung carcinoma cell line	Lung	Carcinoma
0.218	0.177	LS174T	Colon adencocarcinoma	Colon	Adenocarcinoma

0.062	0.023	Leukemia cells	Leukemia	Blood	Leukemia
0.062	0.024	UMUC3	Bladder Carcinoma	Urinary tract	Carcinoma
0.209	0.180	KM12	Colon adenocarcinoma	Colon	Adenocarcinoma
0.036	0.009	KKLS	Gastric adenocarcinoma	Stomach	Adenocarcinoma
0.238	0.212	8505C	Thyroid gland undifferentiated (anaplastic) carcinoma	Thyroid	Carcinoma
0.071	0.046	FaDu	Hypopharyngeal squamous carcinoma	Upper aerodigestive tract	Carcinoma
0.027	0.003	OVXF1023	Ovarian adenocarcinoma	Ovarium	Adenocarcinoma
0.027	0.003	OVXF1353	Ovarian adenocarcinoma	Ovarium	Adenocarcinoma
0.027	0.003	RXF 423	Renal carcinoma	Kidney	Carcinoma
0.069	0.047	SISO	Uterine cervical adenocarcinoma	Cervix	Adenocarcinoma
0.025	0.005	NCI-H46	Lung carcinoma	Lung	Carcinoma
0.022	0.003	SW-60	Colorectal carcinoma	Colon	Carcinoma
0.031	0.014	GLC4			
0.030	0.017	SNB-7	Glioblastoma	Brain	Glioblastoma
0.017	0.007	SPC-A4	Lung Adenocarcinoma	Lung	Adenocarcinoma
0.062	0.053	SK-ES1	Ewing sarcoma	Bone	Sarcoma
0.016	0.011	ST-KM-1	Gastric carcinoma	Stomach	Carcinoma

Non-tumor cell line prediction result

Pa > Pi

Pa	Pi	Cell-line	Cell-line full name	Tissue
0.499	0.022	WI-38 VA13	Embryonic lung fibroblast	Lung
0.336	0.005	PBMC	Peripheral blood mononuclear cell	Blood
0.205	0.005	IMR-90	Embryonic lung fibroblast	Lung
0.189	0.023	PrEC	Prostate epithelial cell	Prostate
0.191	0.048	NHDF	Fibroblast	Skin
0.205	0.118	MRC5	Embryonic lung fibroblast	Lung
0.082	0.026	WIL2-NS	Lymphoblastoid cell	Haematopoietic, lymphoid tissue
0.047	0.005	TERT-RPE1	Retinal pigmented epithelial cell	Retina
0.099	0.085	WI-38	Embryonic lung fibroblast	Lung
0.023	0.019	HASMC	Aortic smooth muscle	Muscle

Complex 3

Cancer cell line prediction result

Pa > 0.5

Pa	Pi	Cell-line	Cell-line full name	Tissue	Tumor type
0.645	0.017	Hs 683	Oligodendroglioma	Brain	Glioma

0.516	0.012	NCI-H187	Small cell lung carcinoma	Lung	Carcinoma

Pa > Pi

Pa	Pi	Cell-line	Cell-line full name	Tissue	Tumor type
0.645	0.017	Hs 683	Oligodendroglioma	Brain	Glioma
0.516	0.012	NCI-H187	Small cell lung carcinoma	Lung	Carcinoma
0.438	0.021	HOP-18	Non-small cell lung carcinoma	Lung	Carcinoma
0.330	0.030	MKN-7	Gastric carcinoma	Stomach	Carcinoma
0.295	0.063	HepG2	Hepatoblastoma	Liver	Hepatoblastoma
0.381	0.152	NCI-H838	Non-small cell lung cancer. 3 stage	Lung	Carcinoma
0.189	0.011	COLO 320	Colon adenocarcinoma	Colon	Adenocarcinoma
0.178	0.003	MEXF276L	Xenograft melanoma	Skin	Melanoma
0.178	0.003	MEXF989	Xenograft melanoma	Skin	Melanoma
0.178	0.003	PAXF546	Pancreatic carcinoma	Pancreas	Carcinoma
0.194	0.043	ADR5000	Childhood T acute lymphoblastic leukemia	Blood	Leukemia
0.255	0.132	PC-6	Small cell lung carcinoma	Lung	Carcinoma
0.190	0.097	St-4	Stomach carcinoma	Stomach	Carcinoma
0.253	0.169	8505C	Thyroid gland undifferentiated (anaplastic) carcinoma	Thyroid	Carcinoma
0.092	0.015	UMSCC22B	Hypopharyngeal squamous cell carcinoma	Upper aerodigestive tract	Carcinoma
0.227	0.154	LS174T	Colon adencocarcinoma	Colon	Adenocarcinoma
0.063	0.004	H322	Lung carcinoma	Lung	Carcinoma
0.063	0.007	UCLA P-3	Lung carcinoma cell line	Lung	Carcinoma
0.156	0.107	NUGC-3	Gastric carcinoma	Stomach	Carcinoma
0.064	0.020	Leukemia cells	Leukemia	Blood	Leukemia
0.074	0.041	FaDu	Hypopharyngeal squamous carcinoma	Upper aerodigestive tract	Carcinoma
0.060	0.030	UMUC3	Bladder Carcinoma	Urinary tract	Carcinoma
0.071	0.041	SISO	Uterine cervical adenocarcinoma	Cervix	Adenocarcinoma
0.036	0.009	KKLS	Gastric adenocarcinoma	Stomach	Adenocarcinoma
0.092	0.066	Bcap37	Breast adenocarcinoma	Breast	Adenocarcinoma
0.029	0.004	NCI-H46	Lung carcinoma	Lung	Carcinoma
0.027	0.003	OVXF1023	Ovarian adenocarcinoma	Ovarium	Adenocarcinoma
0.027	0.003	OVXF1353	Ovarian adenocarcinoma	Ovarium	Adenocarcinoma
0.027	0.003	RXF 423	Renal carcinoma	Kidney	Carcinoma
0.022	0.003	SW-60	Colorectal carcinoma	Colon	Carcinoma
0.031	0.014	GLC4			
0.063	0.049	SW1353	Bone chondrosarcoma	Bone	Sarcoma
0.023	0.010	NUGC	Gastric carcinoma	Stomach	Carcinoma
0.030	0.017	SNB-7	Glioblastoma	Brain	Glioblastoma
0.062	0.053	SK-ES1	Ewing sarcoma	Bone	Sarcoma
0.059	0.052	HPAC	Pancreatic adenocarcinoma	Pancreas	Adenocarcinoma
0.016	0.011	ST-KM-1	Gastric carcinoma	Stomach	Carcinoma

Non-tumor cell line prediction result

Pa > Pi

Pa	Pi	Cell-line	Cell-line full name	Tissue
0.445	0.029	WI-38 VA13	Embryonic lung fibroblast	Lung
0.196	0.008	IMR-90	Embryonic lung fibroblast	Lung
0.175	0.033	PrEC	Prostate epithelial cell	Prostate
0.152	0.087	NHDF	Fibroblast	Skin
0.078	0.032	WIL2-NS	Lymphoblastoid cell	Haematopoietic, lymphoid tissue
0.044	0.007	TERT-RPE1	Retinal pigmented epithelial cell	Retina
0.025	0.014	HASMC	Aortic smooth muscle	Muscle

Complex 4

Cancer cell line prediction result

Pa > 0.5

Pa	Pi	Cell-line	Cell-line full name	Tissue	Tumor type
0.603	0.004	PA-1	Ovarian carcinoma	Ovarium	Carcinoma
0.585	0.029	Hs 683	Oligodendroglioma	Brain	Glioma

Pa > Pi

Pa	Pi	Cell-line	Cell-line full name	Tissue	Tumor type
0.603	0.004	PA-1	Ovarian carcinoma	Ovarium	Carcinoma
0.585	0.029	Hs 683	Oligodendroglioma	Brain	Glioma
0.427	0.009	DLD-1	Colon adenocarcinoma	Colon	Adenocarcinoma
0.420	0.042	HL-60	Promyeloblast leukemia	Haematopoietic and lymphoid tissue	Leukemia
0.363	0.018	MKN-7	Gastric carcinoma	Stomach	Carcinoma
0.343	0.014	C8166	Leukemic T-cells	Blood	Leukemia
0.352	0.047	HOP-18	Non-small cell lung carcinoma	Lung	Carcinoma
0.302	0.072	UO-31	Renal carcinoma	Kidney	Carcinoma
0.241	0.031	St-4	Stomach carcinoma	Stomach	Carcinoma
0.360	0.178	NCI-H838	Non-small cell lung cancer. 3 stage	Lung	Carcinoma
0.156	0.004	MEXF276L	Xenograft melanoma	Skin	Melanoma
0.156	0.004	MEXF989	Xenograft melanoma	Skin	Melanoma
0.156	0.004	PAXF546	Pancreatic carcinoma	Pancreas	Carcinoma
0.360	0.210	DMS-114	Lung carcinoma	Lung	Carcinoma
0.249	0.130	Hs-578T	Invasive ductal breast carcinoma	Breast	Carcinoma
0.142	0.025	COLO 320	Colon adenocarcinoma	Colon	Adenocarcinoma
0.169	0.063	ADR5000	Childhood T acute lymphoblastic leukemia	Blood	Leukemia
0.118	0.024	SiHa	Cervical squamous cell	Cervix	Carcinoma

			carcinoma		
0.166	0.104	Caco-2	Colon adenocarcinoma	Colon	Adenocarcinoma
0.246	0.190	8505C	Thyroid gland undifferentiated (anaplastic) carcinoma	Thyroid	Carcinoma
0.072	0.028	UMSCC22B	Hypopharyngeal squamous cell carcinoma	Upper aerodigestive tract	Carcinoma
0.049	0.006	H322	Lung carcinoma	Lung	Carcinoma
0.092	0.050	ZR-75-1	Breast carcinoma	Breast	Carcinoma
0.062	0.023	Leukemia cells	Leukemia	Blood	Leukemia
0.051	0.014	UCLA P-3	Lung carcinoma cell line	Lung	Carcinoma
0.035	0.010	KKLS	Gastric adenocarcinoma	Stomach	Adenocarcinoma
0.070	0.049	FaDu	Hypopharyngeal squamous carcinoma	Upper aerodigestive tract	Carcinoma
0.021	0.004	OVXF1023	Ovarian adenocarcinoma	Ovarium	Adenocarcinoma
0.021	0.004	OVXF1353	Ovarian adenocarcinoma	Ovarium	Adenocarcinoma
0.021	0.004	RXF 423	Renal carcinoma	Kidney	Carcinoma
0.017	0.004	SW-60	Colorectal carcinoma	Colon	Carcinoma
0.019	0.011	NCI-H46	Lung carcinoma	Lung	Carcinoma
0.177	0.170	SF-295	Glioblastoma	Brain	Glioblastoma
0.024	0.022	GLC4			

Non-tumor cell line prediction result

Pa > Pi

Pa	Pi	Cell-line	Cell-line full name	Tissue
0.309	0.053	WI-38 VA13	Embryonic lung fibroblast	Lung
0.212	0.010	PBMC	Peripheral blood mononuclear cell	Blood
0.173	0.027	IMR-90	Embryonic lung fibroblast	Lung
0.143	0.078	PrEC	Prostate epithelial cell	Prostate
0.036	0.019	TERT-RPE1	Retinal pigmented epithelial cell	Retina

Complex 5

Cancer cell line prediction result

Pa > 0.5

Pa	Pi	Cell-line	Cell-line full name	Tissue	Tumor type
0.601	0.020	NCI-H838	Non-small cell lung cancer. 3 stage	Lung	Carcinoma
0.572	0.033	Hs 683	Oligodendroglioma	Brain	Glioma
0.536	0.044	MCF7	Breast carcinoma	Breast	Carcinoma
0.507	0.060	A549	Lung carcinoma	Lung	Carcinoma

Pa > Pi

Pa	Pi	Cell-line	Cell-line full name	Tissue	Tumor type
0.601	0.020	NCI-H838	Non-small cell lung cancer. 3 stage	Lung	Carcinoma
0.572	0.033	Hs 683	Oligodendroglioma	Brain	Glioma
0.536	0.044	MCF7	Breast carcinoma	Breast	Carcinoma
0.474	0.014	HOP-18	Non-small cell lung carcinoma	Lung	Carcinoma
0.507	0.060	A549	Lung carcinoma	Lung	Carcinoma
0.469	0.023	NCI-H187	Small cell lung carcinoma	Lung	Carcinoma
0.483	0.038	SK-MEL-1	Metastatic melanoma	Skin	Melanoma
0.466	0.057	DMS-114	Lung carcinoma	Lung	Carcinoma
0.414	0.020	NALM-6	Adult B acute lymphoblastic leukemia	Haematopoietic and lymphoid tissue	Leukemia
0.430	0.038	A2058	Melanoma	Skin	Melanoma
0.395	0.013	DLD-1	Colon adenocarcinoma	Colon	Adenocarcinoma
0.416	0.035	CFPAC-1	Pancreatic carcinoma	Pancreas	Carcinoma
0.379	0.014	MKN-7	Gastric carcinoma	Stomach	Carcinoma
0.363	0.023	8505C	Thyroid gland undifferentiated (anaplastic) carcinoma	Thyroid	Carcinoma
0.359	0.029	H9	T-lymphoid	Haematopoietic and lymphoid tissue	Leukemia
0.407	0.083	MDA-MB-453	Breast adenocarcinoma	Breast	Adenocarcinoma
0.365	0.043	M19-MEL	Melanoma	Skin	Melanoma
0.356	0.056	PC-6	Small cell lung carcinoma	Lung	Carcinoma
0.388	0.117	YAPC	Pancreatic carcinoma	Pancreas	Carcinoma
0.378	0.113	Kasumi 1	Childhood acute myeloid leukemia with maturation	Haematopoietic and lymphoid tissue	Leukemia
0.326	0.063	T98G	Glioblastoma	Brain	Carcinoma
0.289	0.028	Raji	B-lymphoblastic cells	Haematopoietic and lymphoid tissue	Leukemia
0.273	0.037	A2780	Ovarian carcinoma	Ovarium	Carcinoma
0.278	0.043	PANC-1	Pancreatic carcinoma	Pancreas	Carcinoma
0.305	0.073	SN12C	Renal carcinoma	Kidney	Carcinoma
0.257	0.025	BGC-823	Stomach adenocarcinoma	Stomach	Adenocarcinoma
0.248	0.020	ADR5000	Childhood T acute lymphoblastic leukemia	Blood	Leukemia
0.286	0.064	CWR22R	Prostate carcinoma epithelial cell line	Prostate	Carcinoma
0.316	0.097	SJSA-1	Osteosarcoma	Bone	Sarcoma
0.202	0.009	COLO 320	Colon adenocarcinoma	Colon	Adenocarcinoma
0.208	0.023	SMMC-7721	Hepatocellular carcinoma	Liver	Carcinoma
0.262	0.078	LS174T	Colon adencocarcinoma	Colon	Adenocarcinoma
0.307	0.129	U-266	Plasma cell myeloma	Blood	Myeloma
0.176	0.003	MEXF276L	Xenograft melanoma	Skin	Melanoma
0.176	0.003	MEXF989	Xenograft melanoma	Skin	Melanoma
0.176	0.003	PAXF546	Pancreatic carcinoma	Pancreas	Carcinoma
0.197	0.025	SHP77	Small cell lung carcinoma	Lung	Carcinoma
0.218	0.056	St-4	Stomach carcinoma	Stomach	Carcinoma
0.271	0.110	HuP-T3	Pancreatic adenocarcinoma	Pancreas	Adenocarcinoma
0.183	0.028	LNCaP	Prostate carcinoma	Prostate	Carcinoma
0.231	0.087	UACC-62	Melanoma	Skin	Melanoma
0.275	0.133	OVCAR-5	Ovarian adenocarcinoma	Ovarium	Adenocarcinoma

0.208	0.080	Bel-7402	Hepatoma	Liver	Hepatoma
0.292	0.169	RKO	Colon carcinoma	Colon	Carcinoma
0.147	0.031	RXF 944	Renal carcinoma	Kidney	Carcinoma
0.271	0.162	OVCAR-4	Ovarian adenocarcinoma	Ovarium	Adenocarcinoma
0.154	0.048	U-937	Histiocytic lymphoma	Haematopoietic and lymphoid tissue	Lymphoma
0.170	0.065	MAXF401	Breast carcinoma	Breast	Carcinoma
0.215	0.114	HOP-62	Non-small cell lung carcinoma	Lung	Carcinoma
0.144	0.046	SK-BR-3	Breast adenocarcinoma	Breast	Adenocarcinoma
0.175	0.078	SW1573	Lung carcinoma	Lung	Carcinoma
0.116	0.021	1A9	Ovarian adenocarcinoma	Ovarium	Adenocarcinoma
0.107	0.014	FaDu	Hypopharyngeal squamous carcinoma	Upper aerodigestive tract	Carcinoma
0.252	0.160	HOS	Osteosarcoma	Bone	Sarcoma
0.235	0.144	KARPAS-299	Anaplastic large cell lymphoma	Haematopoietic and lymphoid tissue	Leukemia
0.238	0.154	Hs-578T	Invasive ductal breast carcinoma	Breast	Carcinoma
0.085	0.004	UCLA P-3	Lung carcinoma cell line	Lung	Carcinoma
0.107	0.029	Bcap37	Breast adenocarcinoma	Breast	Adenocarcinoma
0.232	0.157	UO-31	Renal carcinoma	Kidney	Carcinoma
0.186	0.112	SF-539	Glioblastoma	Brain	Glioblastoma
0.141	0.068	U-937/GTB	Histiocytic lymphoma	Blood	Lymphoma
0.098	0.026	SK-MEL	Melanoma	Skin	Melanoma
0.221	0.154	PC-3	Prostate carcinoma	Prostate	Carcinoma
0.098	0.032	Lu1	Lung carcinoma	Lung	Carcinoma
0.201	0.135	HeLa	Cervical adenocarcinoma	Cervix	Adenocarcinoma
0.216	0.157	SK-MEL-28	Melanoma	Skin	Melanoma
0.102	0.046	ASPC1	Pancreatic ductal adenocarcinoma	Pancreas	Adenocarcinoma
0.097	0.044	ZR-75-1	Breast carcinoma	Breast	Carcinoma
0.069	0.016	Leukemia cells	Leukemia	Blood	Leukemia
0.265	0.212	PA-1	Ovarian carcinoma	Ovarium	Carcinoma
0.113	0.061	SW480	Colon adenocarcinoma	Colon	Adenocarcinoma
0.219	0.167	SF-268	Glioblastoma	Brain	Glioblastoma
0.206	0.155	MDA-MB-361	Breast adenocarcinoma	Breast	Adenocarcinoma
0.072	0.022	SK-VLB	Ovarian carcinoma	Ovarium	Carcinoma
0.071	0.022	L2987	Lung adenocarcinoma	Lung	Adenocarcinoma
0.052	0.005	H322	Lung carcinoma	Lung	Carcinoma
0.255	0.210	SK-MES-1	Squamous cell lung carcinoma	Lung	Carcinoma
0.048	0.007	GLC4			
0.131	0.092	Caov-3	High grade ovarian serous adenocarcinoma	Ovarium	Adenocarcinoma
0.061	0.026	UMUC3	Bladder Carcinoma	Urinary tract	Carcinoma
0.177	0.144	HT-1080	Fibrosarcoma	Soft tissue	Sarcoma
0.073	0.041	MCF7S	Breast carcinoma	Breast	Carcinoma
0.026	0.003	SW-60	Colorectal carcinoma	Colon	Carcinoma
0.211	0.187	NCI-H226	Non-small cell lung carcinoma	Lung	Carcinoma
0.026	0.005	NCI-H46	Lung carcinoma	Lung	Carcinoma
0.032	0.011	KKLS	Gastric adenocarcinoma	Stomach	Adenocarcinoma

0.204	0.186	OVCAR-8	Ovarian adenocarcinoma	Ovarium	Adenocarcinoma
0.021	0.004	OVXF1023	Ovarian adenocarcinoma	Ovarium	Adenocarcinoma
0.021	0.004	OVXF1353	Ovarian adenocarcinoma	Ovarium	Adenocarcinoma
0.021	0.004	RXF 423	Renal carcinoma	Kidney	Carcinoma
0.091	0.075	SiHa	Cervical squamous cell carcinoma	Cervix	Carcinoma
0.078	0.062	Col2	Colon carcinoma	Colon	Carcinoma
0.237	0.224	UACC-257	Melanoma	Skin	Melanoma
0.037	0.024	1A9/ptx-10	Ovarian adenocarcinoma	Ovarium	Adenocarcinoma
0.122	0.111	LXFL 529	Non-small cell lung carcinoma	Lung	Carcinoma
0.150	0.140	Caco-2	Colon adenocarcinoma	Colon	Adenocarcinoma
0.091	0.082	HCT-8	Ileocecal adenocarcinoma	Large intestine	Adenocarcinoma
0.057	0.050	UMSCC22B	Hypopharyngeal squamous cell carcinoma	Upper aerodigestive tract	Carcinoma
0.025	0.021	SNB-7	Glioblastoma	Brain	Glioblastoma
0.014	0.013	ST-KM-1	Gastric carcinoma	Stomach	Carcinoma
0.176	0.175	HCC 2998	Colon adenocarcinoma	Colon	Adenocarcinoma
0.016	0.015	SW-1736	Thyroid gland undifferentiated (anaplastic) carcinoma	Thyroid	Carcinoma
0.005	0.004	MOVP-3	Adult T acute lymphoblastic leukemia	Blood	Leukemia

Non-tumor cell line prediction result

Pa > Pi

Pa	Pi	Cell-line	Cell-line full name	Tissue
0.748	0.009	WI-38 VA13	Embryonic lung fibroblast	Lung
0.262	0.047	BJ	Foreskin fibroblast	Foreskin
0.223	0.025	NHDF	Fibroblast	Skin
0.180	0.018	IMR-90	Embryonic lung fibroblast	Lung
0.232	0.100	MRC5	Embryonic lung fibroblast	Lung
0.164	0.041	HUVEC	Umbilical vein endothelial cell	Endothelium
0.177	0.088	HEL 299	Fibroblast	Lung
0.140	0.085	PrEC	Prostate epithelial cell	Prostate
0.050	0.009	RPTEC	Renal proximal tubule epithelial cells	Kidney
0.086	0.046	HaCaT	Keratinocyte	Skin
0.073	0.044	WIL2-NS	Lymphoblastoid cell	Haematopoietic, lymphoid tissue
0.039	0.014	TERT-RPE1	Retinal pigmented epithelial cell	Retina
0.029	0.009	HASMC	Aortic smooth muscle	Muscle

Table S13. Sites of metabolism prediction (SOMP) for complexes **1–5** by GUSAR.

Complex 1

Meta-Pred Web Server prediction results of site of metabolism for enzyme **UGT**

Atom Number	Rank	DeltaP
20	1	0,126
21	1	0,126
3	2	-0,818
17	2	-0,818
4	3	-0,761
5	3	-0,761
18	3	-0,761
19	3	-0,761
1	4	-0,680
2	4	-0,680
15	4	-0,680
16	4	-0,680
22	4	-0,680
24	4	-0,680
26	4	-0,680
28	4	-0,680
6	5	-0,574
14	5	-0,574
23	6	-0,461
25	6	-0,461
27	6	-0,461
29	6	-0,461
7	7	-0,341
13	7	-0,341
10	8	-0,071
9	9	-0,067
11	9	-0,067
8	10	-0,049
12	10	-0,049

Meta-Pred Web Server prediction results of site of metabolism for enzyme **CYP3A4**

Atom Number	Rank	DeltaP
22	1	0,917
24	1	0,917
26	1	0,917
28	1	0,917
7	2	0,287
13	2	0,287
3	3	0,172
17	3	0,172
4	4	0,155
18	4	0,155
10	5	0,083
1	6	0,027
15	6	0,027
23	7	0,013
25	7	0,013
27	7	0,013
29	7	0,013
20	8	-0,536
21	8	-0,536
2	9	-0,447
16	9	-0,447
9	10	-0,336
11	10	-0,336
6	11	-0,239
14	11	-0,239
5	12	-0,161
19	12	-0,161
8	13	-0,128
12	13	-0,128

Meta-Pred Web Server prediction results of site of metabolism for enzyme **CYP2D6**

Atom Number	Rank	DeltaP
22	1	0,732
24	1	0,732
26	1	0,732
28	1	0,732
7	2	0,154
13	2	0,154
10	3	0,019
3	4	0,013
17	4	0,013
20	5	-0,609
21	5	-0,609
6	6	-0,443
14	6	-0,443
5	7	-0,417
9	7	-0,417
11	7	-0,417
19	7	-0,417
2	8	-0,407
16	8	-0,407
23	9	-0,252
25	9	-0,252
27	9	-0,252
29	9	-0,252
8	10	-0,218
12	10	-0,218
1	11	-0,190
15	11	-0,190
4	12	-0,141
18	12	-0,141

Meta-Pred Web Server prediction results of site of metabolism for enzyme **CYP2C9**

Atom Number	Rank	DeltaP
22	1	0,748
24	1	0,748
26	1	0,748
28	1	0,748
20	2	0,604
21	2	0,604
7	3	0,233
13	3	0,233
10	4	0,184
23	5	0,050
25	5	0,050
27	5	0,050
29	5	0,050
6	6	-0,431
14	6	-0,431
2	7	-0,361
16	7	-0,361
5	8	-0,294
19	8	-0,294
9	9	-0,252
11	9	-0,252
1	10	-0,119
15	10	-0,119
4	11	-0,107
18	11	-0,107
3	12	-0,032
17	12	-0,032
8	13	-0,028
12	13	-0,028

Meta-Pred Web Server prediction results of site of metabolism for enzyme **CYP2C19**

Atom Number	Rank	DeltaP
22	1	0,737
24	1	0,737
26	1	0,737
28	1	0,737
7	2	0,325
13	2	0,325
10	3	0,186
3	4	0,091
17	4	0,091
23	5	0,040
25	5	0,040
27	5	0,040
29	5	0,040
20	6	-0,425
21	6	-0,425
6	7	-0,328
14	7	-0,328
2	8	-0,284
16	8	-0,284
9	9	-0,235
11	9	-0,235
5	10	-0,123
19	10	-0,123
4	11	-0,036
18	11	-0,036
8	12	-0,029
12	12	-0,029
1	13	-0,008
15	13	-0,008

Meta-Pred Web Server prediction results of site of metabolism for enzyme **CYP1A2**

Atom Number	Rank	DeltaP
22	1	0,715
24	1	0,715
26	1	0,715
28	1	0,715
7	2	0,210
13	2	0,210
4	3	0,135
18	3	0,135
3	4	0,059
10	4	0,059
17	4	0,059
20	5	-0,453
21	5	-0,453
2	6	-0,332
16	6	-0,332
9	7	-0,252
11	7	-0,252
6	8	-0,197
14	8	-0,197
23	9	-0,153
25	9	-0,153
27	9	-0,153
29	9	-0,153
5	10	-0,137
19	10	-0,137
8	11	-0,102
12	11	-0,102
1	12	-0,089
15	12	-0,089

Complex 2

Meta-Pred Web Server prediction results of site of metabolism for enzyme **UGT**

Atom Number	Rank	DeltaP
7	1	0,059
14	1	0,059
4	2	-0,757
5	2	-0,757
19	2	-0,757
20	2	-0,757
1	3	-0,640
2	3	-0,640
3	3	-0,640
16	3	-0,640
17	3	-0,640
18	3	-0,640
6	4	-0,330
8	4	-0,330
10	4	-0,330
15	4	-0,330
21	4	-0,330
22	4	-0,330
9	5	-0,138
23	5	-0,138
11	6	-0,022
13	6	-0,022
12	7	-0,021

Meta-Pred Web Server prediction results of site of metabolism for enzyme **CYP3A4**

Atom Number	Rank	DeltaP
8	1	0,069
21	1	0,069
10	2	-0,986
22	2	-0,986
3	3	-0,972
18	3	-0,972
6	4	-0,937
15	4	-0,937
1	5	-0,930
16	5	-0,930
9	6	-0,919
23	6	-0,919
2	7	-0,809
17	7	-0,809
4	8	-0,774
19	8	-0,774
11	9	-0,615
13	9	-0,615
5	10	-0,605
20	10	-0,605
7	11	-0,358
14	11	-0,358
12	12	-0,056

Meta-Pred Web Server prediction results of site of metabolism for enzyme **CYP2D6**

Atom Number	Rank	DeltaP
8	1	0,372
21	1	0,372
12	2	0,215
1	3	-0,928
16	3	-0,928
3	4	-0,927
18	4	-0,927
9	5	-0,881
10	5	-0,881
22	5	-0,881
23	5	-0,881
6	6	-0,853
15	6	-0,853
2	7	-0,638
17	7	-0,638
5	8	-0,528
20	8	-0,528
4	9	-0,491
19	9	-0,491
7	10	-0,326
11	10	-0,326
13	10	-0,326
14	10	-0,326

Meta-Pred Web Server prediction results of site of metabolism for enzyme **CYP2C9**

Atom Number	Rank	DeltaP
8	1	0,319
21	1	0,319
12	2	0,141
1	3	-0,950
16	3	-0,950
3	4	-0,948
18	4	-0,948
6	5	-0,888
15	5	-0,888
9	6	-0,737
10	6	-0,737
22	6	-0,737
23	6	-0,737
4	7	-0,722
19	7	-0,722
2	8	-0,705
17	8	-0,705
5	9	-0,544
20	9	-0,544
11	10	-0,494
13	10	-0,494
7	11	-0,172
14	11	-0,172

Meta-Pred Web Server prediction results of site of metabolism for enzyme **CYP2C19**

Atom Number	Rank	DeltaP
8	1	0,458
21	1	0,458
12	2	0,227
10	3	-0,899
22	3	-0,899
3	4	-0,874
18	4	-0,874
1	5	-0,843
16	5	-0,843
9	6	-0,788
23	6	-0,788
6	7	-0,777
15	7	-0,777
2	8	-0,565
17	8	-0,565
4	9	-0,564
19	9	-0,564
11	10	-0,339
13	10	-0,339
5	11	-0,210
20	11	-0,210
7	12	-0,060
14	12	-0,060

Meta-Pred Web Server prediction results of site of metabolism for enzyme **CYP1A2**

Atom Number	Rank	DeltaP
8	1	0,299
21	1	0,299
12	2	0,042
10	3	-0,908
22	3	-0,908
3	4	-0,820
18	4	-0,820
1	5	-0,809
16	5	-0,809
9	6	-0,796
23	6	-0,796
6	7	-0,746
15	7	-0,746
2	8	-0,410
17	8	-0,410
11	9	-0,399
13	9	-0,399
4	10	-0,382
19	10	-0,382
5	11	-0,336
20	11	-0,336
7	12	-0,193
14	12	-0,193

Complex 3

Meta-Pred Web Server prediction results of site of metabolism for enzyme **UGT**

Atom Number	Rank	DeltaP
8	1	0,073
12	1	0,073
4	2	-0,736
18	2	-0,736
1	3	-0,626
2	3	-0,626
3	3	-0,626
5	3	-0,626
15	3	-0,626
16	3	-0,626
17	3	-0,626
19	3	-0,626
6	4	-0,345
14	4	-0,345
20	5	-0,261
21	5	-0,261
7	6	-0,227
13	6	-0,227
22	7	-0,127
23	7	-0,127
9	8	-0,010
11	8	-0,010
10	9	-0,006

Meta-Pred Web Server prediction results of site of metabolism for enzyme **CYP3A4**

Atom Number	Rank	DeltaP
2	1	0,588
16	1	0,588
4	2	0,334
18	2	0,334
7	3	0,267
13	3	0,267
10	4	0,233
6	5	0,012
14	5	0,012
20	6	-0,953
21	6	-0,953
3	7	-0,920
17	7	-0,920
22	8	-0,776
23	8	-0,776
1	9	-0,684
15	9	-0,684
9	10	-0,327
11	10	-0,327
5	11	-0,214
19	11	-0,214
8	12	-0,047
12	12	-0,047

Meta-Pred Web Server prediction results of site of metabolism for enzyme **CYP2D6**

Atom Number	Rank	DeltaP
2	1	0,544
16	1	0,544
4	2	0,326
18	2	0,326
7	3	0,146
13	3	0,146
10	4	0,139
6	5	0,025
14	5	0,025
20	6	-0,965
21	6	-0,965
3	7	-0,948
17	7	-0,948
22	8	-0,808
23	8	-0,808
1	9	-0,782
15	9	-0,782
5	10	-0,445
19	10	-0,445
9	11	-0,412
11	11	-0,412
8	12	-0,157
12	12	-0,157

Meta-Pred Web Server prediction results of site of metabolism for enzyme **CYP2C9**

Atom Number	Rank	DeltaP
10	1	0,136
7	2	0,123
13	2	0,123
20	3	-0,940
21	3	-0,940
3	4	-0,888
17	4	-0,888
1	5	-0,736
15	5	-0,736
22	6	-0,689
23	6	-0,689
6	7	-0,679
14	7	-0,679
5	8	-0,609
19	8	-0,609
9	9	-0,459
11	9	-0,459
4	10	-0,281
18	10	-0,281
8	11	-0,155
12	11	-0,155
2	12	-0,021
16	12	-0,021

Meta-Pred Web Server prediction results of site of metabolism for enzyme **CYP2C19**

Atom Number	Rank	DeltaP
7	1	0,335
13	1	0,335
10	2	0,271
8	3	0,043
12	3	0,043
20	4	-0,915
21	4	-0,915
3	5	-0,896
17	5	-0,896
22	6	-0,686
23	6	-0,686
1	7	-0,680
15	7	-0,680
6	8	-0,550
14	8	-0,550
9	9	-0,225
11	9	-0,225
4	10	-0,215
18	10	-0,215
2	11	-0,201
16	11	-0,201
5	12	-0,198
19	12	-0,198

Meta-Pred Web Server prediction results of site of metabolism for enzyme **CYP1A2**

Atom Number	Rank	DeltaP
2	1	0,710
16	1	0,710
4	2	0,388
18	2	0,388
7	3	0,126
13	3	0,126
10	4	0,082
20	5	-0,930
21	5	-0,930
3	6	-0,861
17	6	-0,861
22	7	-0,713
23	7	-0,713
1	8	-0,575
15	8	-0,575
9	9	-0,332
11	9	-0,332
5	10	-0,287
19	10	-0,287
8	11	-0,140
12	11	-0,140
6	12	-0,002
14	12	-0,002

Complex 4

Meta-Pred Web Server prediction results of site of metabolism for enzyme **UGT**

Atom Number	Rank	DeltaP
2	1	-0,914
16	1	-0,914
1	2	-0,818
3	2	-0,818
4	2	-0,818
5	2	-0,818
15	2	-0,818
17	2	-0,818
18	2	-0,818
19	2	-0,818
6	3	-0,637
14	3	-0,637
20	4	-0,503
21	4	-0,503
7	5	-0,355
13	5	-0,355
10	6	-0,045
9	7	-0,043
11	7	-0,043
8	8	-0,024
12	8	-0,024

Meta-Pred Web Server prediction results of site of metabolism for enzyme **CYP3A4**

Atom Number	Rank	DeltaP
7	1	0,182
13	1	0,182
10	2	0,107
1	3	0,102
15	3	0,102
2	4	0,054
16	4	0,054
20	5	-0,961
21	5	-0,961
3	6	-0,918
17	6	-0,918
6	7	-0,573
14	7	-0,573
8	8	-0,422
9	8	-0,422
11	8	-0,422
12	8	-0,422
5	9	-0,374
19	9	-0,374
4	10	-0,033
18	10	-0,033

Meta-Pred Web Server prediction results of site of metabolism for enzyme **CYP2D6**

Atom Number	Rank	DeltaP
2	1	0,425
16	1	0,425
7	2	0,145
13	2	0,145
10	3	0,139
20	4	-0,952
21	4	-0,952
3	5	-0,844
17	5	-0,844
6	6	-0,697
14	6	-0,697
5	7	-0,595
19	7	-0,595
8	8	-0,377
9	8	-0,377
11	8	-0,377
12	8	-0,377
4	9	-0,280
18	9	-0,280
1	10	-0,003
15	10	-0,003

Meta-Pred Web Server prediction results of site of metabolism for enzyme **CYP2C9**

Atom Number	Rank	DeltaP
1	1	0,160
15	1	0,160
10	2	0,132
7	3	0,120
13	3	0,120
20	4	-0,918
21	4	-0,918
3	5	-0,909
17	5	-0,909
6	6	-0,819
14	6	-0,819
5	7	-0,691
19	7	-0,691
4	8	-0,530
18	8	-0,530
8	9	-0,426
9	9	-0,426
11	9	-0,426
12	9	-0,426
2	10	-0,017
16	10	-0,017

Meta-Pred Web Server prediction results of site of metabolism for enzyme **CYP2C19**

Atom Number	Rank	DeltaP
7	1	0,277
13	1	0,277
1	2	0,230
15	2	0,230
10	3	0,194
2	4	0,183
16	4	0,183
20	5	-0,925
21	5	-0,925
3	6	-0,835
17	6	-0,835
6	7	-0,710
14	7	-0,710
5	8	-0,387
19	8	-0,387
8	9	-0,310
9	9	-0,310
11	9	-0,310
12	9	-0,310
4	10	-0,056
18	10	-0,056

Meta-Pred Web Server prediction results of site of metabolism for enzyme **CYP1A2**

Atom Number	Rank	DeltaP
2	1	0,055
16	1	0,055
20	2	-0,959
21	2	-0,959
3	3	-0,898
17	3	-0,898
6	4	-0,676
14	4	-0,676
5	5	-0,496
19	5	-0,496
8	6	-0,479
9	6	-0,479
11	6	-0,479
12	6	-0,479
4	7	-0,200
18	7	-0,200
10	8	-0,095
1	9	-0,010
15	9	-0,010
7	10	-0,003
13	10	-0,003

Complex 5

Meta-Pred Web Server prediction results of site of metabolism for enzyme **UGT**

Atom Number	Rank	DeltaP
3	1	-0,838
17	1	-0,838
2	2	-0,783
16	2	-0,783
4	3	-0,708
5	3	-0,708
18	3	-0,708
19	3	-0,708
1	4	-0,603
15	4	-0,603
6	5	-0,487
14	5	-0,487
22	5	-0,487
23	5	-0,487
7	6	-0,250
13	6	-0,250
10	7	-0,040
9	8	-0,039
11	8	-0,039
20	9	-0,022
21	9	-0,022
8	10	-0,021
12	10	-0,021

Meta-Pred Web Server prediction results of site of metabolism for enzyme **CYP3A4**

Atom Number	Rank	DeltaP
22	1	0,802
23	1	0,802
7	2	0,252
13	2	0,252
4	3	0,165
18	3	0,165
3	4	0,138
17	4	0,138
1	5	0,012
15	5	0,012
20	6	-0,991
21	6	-0,991
2	7	-0,807
16	7	-0,807
5	8	-0,517
19	8	-0,517
8	9	-0,471
9	9	-0,471
11	9	-0,471
12	9	-0,471
6	10	-0,392
14	10	-0,392
10	11	-0,006

Meta-Pred Web Server prediction results of site of metabolism for enzyme **CYP2D6**

Atom Number	Rank	DeltaP
22	1	0,916
23	1	0,916
7	2	0,035
13	2	0,035
20	3	-0,991
21	3	-0,991
2	4	-0,845
16	4	-0,845
5	5	-0,691
19	5	-0,691
6	6	-0,677
14	6	-0,677
8	7	-0,605
9	7	-0,605
11	7	-0,605
12	7	-0,605
4	8	-0,194
18	8	-0,194
1	9	-0,192
15	9	-0,192
10	10	-0,187
3	11	-0,072
17	11	-0,072

Meta-Pred Web Server prediction results of site of metabolism for enzyme **CYP2C9**

Atom Number	Rank	DeltaP
22	1	0,844
23	1	0,844
7	2	0,038
13	2	0,038
20	3	-0,991
21	3	-0,991
2	4	-0,927
16	4	-0,927
5	5	-0,779
19	5	-0,779
6	6	-0,759
14	6	-0,759
9	7	-0,588
11	7	-0,588
8	8	-0,355
12	8	-0,355
4	9	-0,292
18	9	-0,292
1	10	-0,155
15	10	-0,155
3	11	-0,113
17	11	-0,113
10	12	-0,094

Meta-Pred Web Server prediction results of site of metabolism for enzyme **CYP2C19**

Atom Number	Rank	DeltaP
22	1	0,877
23	1	0,877
7	2	0,262
13	2	0,262
3	3	0,223
17	3	0,223
1	4	0,211
15	4	0,211
10	5	0,035
20	6	-0,989
21	6	-0,989
2	7	-0,822
16	7	-0,822
6	8	-0,546
14	8	-0,546
5	9	-0,479
19	9	-0,479
8	10	-0,419
9	10	-0,419
11	10	-0,419
12	10	-0,419
4	11	-0,135
18	11	-0,135

Meta-Pred Web Server prediction results of site of metabolism for enzyme **CYP1A2**

Atom Number	Rank	DeltaP
22	1	0,810
23	1	0,810
3	2	0,062
17	2	0,062
7	3	0,057
13	3	0,057
4	4	0,025
18	4	0,025
20	5	-0,991
21	5	-0,991
2	6	-0,814
16	6	-0,814
5	7	-0,604
19	7	-0,604
8	8	-0,525
9	8	-0,525
11	8	-0,525
12	8	-0,525
6	9	-0,461
14	9	-0,461
10	10	-0,184
1	11	-0,021
15	11	-0,021

Table S14. Prediction of activity spectra for complexes **1–5**, for Pa > 0.7 and Pa > 0.0.

COMPLEX 1

All Pa>Pi Pa>0,3 Pa>0,7

Pa	Pi	Activity
0,892	0,007	Phobic disorders treatment
0,730	0,022	Acylcarnitine hydrolase inhibitor
0,707	0,007	Cardiovascular analeptic
0,703	0,013	27-Hydroxycholesterol 7alpha-monooxygenase inhibitor
0,722	0,053	CYP2C12 substrate
0,702	0,054	Testosterone 17beta-dehydrogenase (NADP+) inhibitor

COMPLEX 2

All Pa>Pi Pa>0,3 Pa>0,7

Pa	Pi	Activity
0,787	0,003	Vascular endothelial growth factor 3 antagonist
0,793	0,036	Phobic disorders treatment
0,778	0,031	Testosterone 17beta-dehydrogenase (NADP+) inhibitor
0,762	0,018	Nicotinic alpha6beta3beta4alpha5 receptor antagonist
0,758	0,017	Antineoplastic
0,760	0,029	Acrocylindropepsin inhibitor
0,760	0,029	Saccharopepsin inhibitor
0,760	0,029	Chymosin inhibitor
0,720	0,008	Glucan 1,4-alpha-maltotriohydrolase inhibitor
0,706	0,003	Tyrosine-protein kinase receptor FLT3 inhibitor
0,721	0,019	5-O-(4-coumaroyl)-D-quinate 3'-monooxygenase inhibitor
0,746	0,049	Aspulvinone dimethylallyltransferase inhibitor
0,711	0,024	Nicotinic alpha2beta2 receptor antagonist

COMPLEX 3

All Pa>Pi Pa>0,3 Pa>0,7

Pa	Pi	Activity
0,850	0,001	Prostaglandin E1 antagonist

0,836	0,004	Ophthalmic drug
0,824	0,003	Antiglaucomic
0,831	0,023	Phobic disorders treatment
0,799	0,011	Nicotinic alpha6beta3beta4alpha5 receptor antagonist
0,767	0,002	Gestagen antagonist
0,760	0,013	5-O-(4-coumaroyl)-D-quinate 3'-monooxygenase inhibitor
0,778	0,031	Testosterone 17beta-dehydrogenase (NADP+) inhibitor
0,760	0,016	Nicotinic alpha2beta2 receptor antagonist
0,760	0,029	Acrocylindropepsin inhibitor
0,760	0,029	Saccharopepsin inhibitor
0,760	0,029	Chymosin inhibitor
0,720	0,008	Glucan 1,4-alpha-maltotriohydrolase inhibitor
0,702	0,050	CYP2J substrate

COMPLEX 4

All Pa>Pi Pa>0,3 Pa>0,7

Pa	Pi	Activity
0,799	0,034	Phobic disorders treatment
0,731	0,003	Antineoplastic (non-Hodgkin's lymphoma)
0,733	0,008	Phosphatase inhibitor
0,752	0,038	Testosterone 17beta-dehydrogenase (NADP+) inhibitor
0,733	0,025	Nicotinic alpha6beta3beta4alpha5 receptor antagonist
0,731	0,036	Chymosin inhibitor
0,731	0,036	Acrocylindropepsin inhibitor
0,731	0,036	Saccharopepsin inhibitor

COMPLEX 5

○ All ○ Pa>Pi ○ Pa>0,3 ● Pa>0,7

Pa	Pi	Activity
0,847	0,018	Phobic disorders treatment
0,829	0,009	Antineoplastic
0,832	0,018	Testosterone 17beta-dehydrogenase (NADP+) inhibitor
0,816	0,032	CYP2C12 substrate
0,786	0,015	Acylcarnitine hydrolase inhibitor
0,790	0,023	Saccharopepsin inhibitor
0,790	0,023	Chymosin inhibitor
0,790	0,023	Acrocylindropepsin inhibitor
0,774	0,024	Antiseborrheic
0,754	0,014	Alkylacetylglycerophosphatase inhibitor
0,757	0,024	Alkenylglycerophosphocholine hydrolase inhibitor
0,745	0,013	JAK2 expression inhibitor
0,733	0,003	Antineoplastic (non-Hodgkin's lymphoma)
0,746	0,018	Beta-adrenergic receptor kinase inhibitor
0,746	0,018	G-protein-coupled receptor kinase inhibitor
0,753	0,030	Antieczematic
0,727	0,004	Imidazoline receptor agonist
0,764	0,044	Aspulvinone dimethylallyltransferase inhibitor
0,730	0,018	Lysase inhibitor
0,717	0,008	Glucan 1,4-alpha-maltotriohydrolase inhibitor
0,744	0,036	CYP2J substrate
0,702	0,013	27-Hydroxycholesterol 7alpha-monooxygenase inhibitor
0,729	0,058	Ubiquinol-cytochrome-c reductase inhibitor

0,703	0,032	CYP2J2 substrate
0,701	0,034	Nicotinic alpha6beta3beta4alpha5 receptor antagonist

Complex 1 All

All Pa>Pi Pa>0,3 Pa>0,7

Pa	Pi	Activity
0,892	0,007	Phobic disorders treatment
0,730	0,022	Acylcarnitine hydrolase inhibitor
0,707	0,007	Cardiovascular analeptic
0,703	0,013	27-Hydroxycholesterol 7alpha-monooxygenase inhibitor
0,692	0,004	Peptidoglycan glycosyltransferase inhibitor
0,722	0,053	CYP2C12 substrate
0,671	0,006	Sigma receptor agonist
0,686	0,022	Alkylacetylglycerophosphatase inhibitor
0,664	0,006	Antineoplastic (non-Hodgkin's lymphoma)
0,702	0,054	Testosterone 17beta-dehydrogenase (NADP+) inhibitor
0,666	0,050	Antineurotic
0,623	0,008	Dolichyl-diphosphooligosaccharide-protein glycotransferase inhibitor
0,687	0,073	Ubiquinol-cytochrome-c reductase inhibitor
0,667	0,054	Chymosin inhibitor
0,667	0,054	Acrocylindropepsin inhibitor
0,667	0,054	Saccharopepsin inhibitor
0,607	0,004	Sigma 1 receptor agonist
0,651	0,050	Nicotinic alpha6beta3beta4alpha5 receptor antagonist
0,602	0,006	Imidazoline receptor agonist
0,633	0,038	5-O-(4-coumaroyl)-D-quinate 3'-monooxygenase inhibitor
0,618	0,034	Antidyskinetic
0,607	0,033	Glucan endo-1,6-beta-glucosidase inhibitor
0,638	0,073	CYP2J substrate
0,574	0,021	Glucan 1,4-alpha-maltotriohydrolase inhibitor
0,588	0,044	Ribulose-phosphate 3-epimerase inhibitor
0,581	0,037	UDP-N-acetylglucosamine 4-epimerase inhibitor
0,612	0,081	Membrane permeability inhibitor
0,544	0,016	Apyrase inhibitor

0,536	0,019	Cyclic AMP agonist
0,544	0,028	Trimethylamine-oxide aldolase inhibitor
0,584	0,068	CYP2J2 substrate
0,556	0,042	Acetylcholine neuromuscular blocking agent
0,552	0,039	Glucan endo-1,3-beta-D-glucosidase inhibitor
0,552	0,043	Platelet aggregation stimulant
0,544	0,036	Ovulation inhibitor
0,571	0,063	Phosphatase inhibitor
0,548	0,050	CYP3A2 substrate
0,556	0,059	Nicotinic alpha2beta2 receptor antagonist
0,597	0,101	Mucomembranous protector
0,532	0,042	Platelet adhesion inhibitor
0,493	0,020	H+-exporting ATPase inhibitor
0,525	0,060	Protein-disulfide reductase (glutathione) inhibitor
0,550	0,088	Polyporopepsin inhibitor
0,481	0,021	2-Haloacid dehalogenase inhibitor
0,531	0,074	Sugar-phosphatase inhibitor
0,505	0,051	5 Hydroxytryptamine release stimulant
0,482	0,029	Na+-transporting two-sector ATPase inhibitor
0,528	0,077	Fusarinine-C ornithinesterase inhibitor
0,467	0,017	Antiparkinsonian
0,518	0,069	Antiseborrheic
0,494	0,053	Antiviral (Picornavirus)
0,472	0,036	CYP2A4 substrate
0,462	0,026	Nitrite reductase (NO-forming) inhibitor
0,465	0,031	CYP2A2 substrate
0,518	0,084	Glutamyl endopeptidase II inhibitor
0,493	0,061	Carboxypeptidase Taq inhibitor
0,484	0,054	CYP3A1 substrate
0,455	0,028	CYP2D2 inhibitor
0,468	0,042	Phenol O-methyltransferase inhibitor
0,475	0,052	Antinociceptive
0,455	0,040	Antisecretoric
0,470	0,057	1,4-Lactonase inhibitor
0,484	0,071	Macrophage colony stimulating factor agonist
0,476	0,063	Venombin AB inhibitor
0,464	0,053	Membrane integrity antagonist

0,415	0,006	Cycloartenol synthase inhibitor
0,439	0,032	Antimyopathies
0,426	0,024	Hematopoietic inhibitor
0,494	0,094	Pseudolysin inhibitor
0,451	0,051	Respiratory analeptic
0,513	0,114	CYP2H substrate
0,460	0,063	Lysine 2,3-aminomutase inhibitor
0,431	0,036	Dementia treatment
0,464	0,069	Limulus clotting factor B inhibitor
0,439	0,044	CYP2D15 substrate
0,448	0,054	Exoribonuclease II inhibitor
0,436	0,043	Acetylgalactosaminyl-O-glycosyl-glycoprotein beta-1,3-N-acetylglucosaminyltransferase inhibitor
0,480	0,087	Omptin inhibitor
0,413	0,020	Chaperonin ATPase inhibitor
0,448	0,056	Ecdysone 20-monooxygenase inhibitor
0,396	0,007	Histamine H1 receptor agonist
0,430	0,043	Membrane permeability enhancer
0,462	0,076	Lysase inhibitor
0,411	0,027	DELTA14-sterol reductase inhibitor
0,385	0,005	Antineoplastic, alkylator
0,436	0,057	CYP2D16 substrate
0,439	0,061	Fragilysin inhibitor
0,416	0,040	Peptide-N4-(N-acetyl-beta-glucosaminyl)asparagine amidase inhibitor
0,393	0,019	Imidazoline I1 receptor agonist
0,427	0,054	Formaldehyde transketolase inhibitor
0,440	0,068	Neurotransmitter antagonist
0,438	0,067	Fibrinogen receptor antagonist
0,441	0,074	Alkenylglycerophosphocholine hydrolase inhibitor
0,452	0,087	Electron-transferring-flavoprotein dehydrogenase inhibitor
0,401	0,036	Albendazole monooxygenase inhibitor
0,427	0,064	Beta-adrenergic receptor kinase inhibitor
0,427	0,064	G-protein-coupled receptor kinase inhibitor
0,425	0,064	4-Nitrophenol 2-monooxygenase inhibitor
0,395	0,035	Long-chain-aldehyde dehydrogenase inhibitor
0,412	0,053	Analeptic
0,407	0,048	All-trans-retinyl-palmitate hydrolase inhibitor
0,385	0,027	Opioid kappa 3 receptor antagonist

0,438	0,081	Alopecia treatment
0,405	0,050	Acetylesterase inhibitor
0,421	0,069	Dimethylargininase inhibitor
0,375	0,022	Galactolipase inhibitor
0,373	0,022	Flavin-containing monooxygenase inhibitor
0,423	0,073	Spasmolytic, urinary
0,357	0,008	Antineoplastic (liver cancer)
0,407	0,060	Gluconate 5-dehydrogenase inhibitor
0,399	0,053	Mucinaminylserine mucinaminidase inhibitor
0,379	0,033	CYP2G1 substrate
0,372	0,027	Oxidizing agent
0,371	0,028	Sorbitol-6-phosphate 2-dehydrogenase inhibitor
0,396	0,057	CYP2B5 substrate
0,386	0,050	Phosphoinositide 5-phosphatase inhibitor
0,403	0,067	Polyamine-transporting ATPase inhibitor
0,370	0,035	Hydroxylamine reductase (NADH) inhibitor
0,519	0,185	Gluconate 2-dehydrogenase (acceptor) inhibitor
0,355	0,021	Adrenaline release stimulant
0,480	0,147	Aspulvinone dimethylallyltransferase inhibitor
0,371	0,039	Poly(beta-D-mannuronate) lyase inhibitor
0,358	0,029	Benzaldehyde dehydrogenase (NADP+) inhibitor
0,371	0,043	Phosphatidylinositol diacylglycerol-lyase inhibitor
0,431	0,105	Complement factor D inhibitor
0,378	0,052	Limulus clotting factor C inhibitor
0,384	0,060	Simian immunodeficiency virus proteinase inhibitor
0,383	0,059	Cyclohexanone monooxygenase inhibitor
0,356	0,033	Xylan endo-1,3-beta-xylosidase inhibitor
0,452	0,130	Kidney function stimulant
0,417	0,095	5 Hydroxytryptamine uptake stimulant
0,369	0,051	Myc inhibitor
0,365	0,048	CYP3A7 substrate
0,345	0,030	N-hydroxy-2-acetamidofluorene reductase inhibitor
0,371	0,058	CYP2C9 inducer
0,364	0,052	Pullulanase inhibitor
0,350	0,039	CYP2B11 substrate
0,401	0,093	Phosphatidylcholine-retinol O-acyltransferase inhibitor
0,347	0,039	Clavaminate synthase inhibitor

0,371	0,063	Tpr proteinase (Porphyromonas gingivalis) inhibitor
0,412	0,105	NADPH-cytochrome-c2 reductase inhibitor
0,381	0,074	Sphinganine kinase inhibitor
0,389	0,083	CYP2A1 substrate
0,347	0,042	Antiprotozoal (Amoeba)
0,334	0,029	Steroid synthesis inhibitor
0,363	0,058	Leukopoiesis inhibitor
0,401	0,097	Cytoprotectant
0,387	0,084	Fibrolase inhibitor
0,324	0,025	Vascular dementia treatment
0,342	0,045	Glycolate dehydrogenase inhibitor
0,345	0,048	Dextranase inhibitor
0,316	0,020	Raynaud's phenomenon treatment
0,342	0,049	Undecaprenyldiphospho-muramoylpentapeptide beta-N-acetylglucosaminyltransferase inhibitor
0,365	0,072	Hydroxylamine oxidase inhibitor
0,366	0,074	Mannan endo-1,4-beta-mannosidase inhibitor
0,360	0,068	GABA aminotransferase inhibitor
0,322	0,032	Alpha-N-acetylglucosaminidase inhibitor
0,313	0,023	Antineoplastic (small cell lung cancer)
0,374	0,085	Neurodegenerative diseases treatment
0,321	0,032	Thiamine-triphosphatase inhibitor
0,350	0,063	Aspergillopepsin I inhibitor
0,408	0,123	Gastrin inhibitor
0,342	0,057	CYP7 inhibitor
0,344	0,060	Lysostaphin inhibitor
0,327	0,045	Taurine-2-oxoglutarate transaminase inhibitor
0,293	0,012	Gastrointestinal motility stimulant
0,373	0,092	Vasoprotector
0,320	0,040	Shikimate O-hydroxycinnamoyltransferase inhibitor
0,300	0,020	Calcium-sensing receptor agonist
0,296	0,016	Farnesoid X receptor antagonist
0,361	0,084	Gonadotropin antagonist
0,412	0,135	Membrane integrity agonist
0,314	0,037	Signal peptidase I inhibitor
0,341	0,065	Chitinase inhibitor
0,337	0,062	Enteropeptidase inhibitor
0,366	0,093	Hydrogen dehydrogenase inhibitor

0,316	0,044	Antitoxic
0,287	0,015	Alpha-pinene-oxide decyclase inhibitor
0,348	0,078	N-acetylneuraminate 7-O(or 9-O)-acetyltransferase inhibitor
0,295	0,026	2-Haloacid dehalogenase (configuration-inverting) inhibitor
0,291	0,023	ATP-binding cassette A1 stimulant
0,288	0,020	Antineoplastic (ovarian cancer)
0,350	0,083	Glycerol-3-phosphate oxidase inhibitor
0,336	0,069	ADP-thymidine kinase inhibitor
0,294	0,028	Oxytocic
0,309	0,046	Dipeptidase E inhibitor
0,346	0,085	Menopausal disorders treatment
0,309	0,048	Opioid dependency treatment
0,267	0,007	Prolactin inhibitor
0,283	0,023	Cyclamate sulfohydrolase inhibitor
0,323	0,063	D-lactaldehyde dehydrogenase inhibitor
0,283	0,024	Alkylator
0,293	0,034	N-acetyllactosaminide beta-1,3-N-acetylglucosaminyltransferase inhibitor
0,310	0,052	Antineoplastic (pancreatic cancer)
0,311	0,054	Thiosulfate dehydrogenase inhibitor
0,293	0,036	Alkylglycerophosphoethanolamine phosphodiesterase inhibitor
0,351	0,094	Fatty-acyl-CoA synthase inhibitor
0,336	0,080	Peptidyl-dipeptidase Dcp inhibitor
0,302	0,046	Hyaluronic acid agonist
0,307	0,052	Arylacetonitrilase inhibitor
0,336	0,080	Alkenylglycerophosphoethanolamine hydrolase inhibitor
0,294	0,038	Glycine dehydrogenase (decarboxylating) inhibitor
0,346	0,091	Caspase 8 stimulant
0,307	0,052	Antiparkinsonian, rigidity relieving
0,367	0,113	Erythropoiesis stimulant
0,349	0,094	Insulin promoter
0,338	0,084	Antipruritic
0,349	0,095	Rubredoxin-NAD+ reductase inhibitor
0,359	0,106	Antihypoxic
0,324	0,071	Antiviral (Adenovirus)
0,400	0,146	Glycosylphosphatidylinositol phospholipase D inhibitor
0,380	0,127	Phospholipid-translocating ATPase inhibitor
0,312	0,059	Cyclomaltodextrinase inhibitor

0,351	0,098	Arginine 2-monooxygenase inhibitor
0,369	0,117	NADPH peroxidase inhibitor
0,297	0,046	MAP kinase kinase 4 inhibitor
0,329	0,079	Glyoxylate reductase inhibitor
0,330	0,080	(R)-Pantolactone dehydrogenase (flavin) inhibitor
0,314	0,065	N-acylmannosamine kinase inhibitor
0,297	0,048	2,3,4,5-Tetrahydropyridine-2,6-dicarboxylate N-succinyltransferase inhibitor
0,337	0,089	Thymidylate 5'-phosphatase inhibitor
0,315	0,068	Loop diuretic
0,369	0,121	Octopamine antagonist
0,305	0,057	Glucan 1,4-alpha-maltotetraohydrolase inhibitor
0,295	0,049	Yeast ribonuclease inhibitor
0,364	0,118	Leukopoiesis stimulant
0,305	0,062	CYP2C10 substrate
0,270	0,028	Antidepressant, Imipramin-like
0,355	0,115	Histamine release stimulant
0,363	0,122	MAP kinase stimulant
0,283	0,045	2-Hydroxy-3-oxoadipate synthase inhibitor
0,333	0,095	Feruloyl esterase inhibitor
0,305	0,067	Pyroglutamyl-peptidase II inhibitor
0,323	0,086	Sulfite dehydrogenase inhibitor
0,314	0,079	Antineoplastic (solid tumors)
0,324	0,090	Gamma-guanidinobutyraldehyde dehydrogenase inhibitor
0,362	0,128	Fructose 5-dehydrogenase inhibitor
0,317	0,085	Methylamine-glutamate N-methyltransferase inhibitor
0,269	0,041	RNA directed DNA polymerase inhibitor
0,312	0,085	Fucosterol-epoxide lyase inhibitor
0,269	0,042	Aldosterone antagonist
0,274	0,048	Sclerosant
0,258	0,033	CYP2D1 substrate
0,375	0,150	Phthalate 4,5-dioxygenase inhibitor
0,260	0,035	Maltose-transporting ATPase inhibitor
0,301	0,077	4-Hydroxymandelate oxidase inhibitor
0,263	0,039	Licheninase inhibitor
0,310	0,087	Adenomatous polyposis treatment
0,267	0,044	Mannitol-1-phosphatase inhibitor
0,300	0,078	Steroid N-acetylglucosaminyltransferase inhibitor

0,303	0,080	Adenylyl-sulfate reductase inhibitor
0,350	0,127	2-Hydroxyquinoline 8-monooxygenase inhibitor
0,270	0,049	Antitussive
0,264	0,044	Phosphoenolpyruvate mutase inhibitor
0,253	0,033	CNS active muscle relaxant
0,239	0,021	GST T substrate
0,239	0,021	GST T1-1 substrate
0,354	0,135	Chloride peroxidase inhibitor
0,301	0,082	3-Cyanoalanine hydratase inhibitor
0,228	0,010	3-Isopropylmalate dehydrogenase inhibitor
0,294	0,076	Mannose isomerase inhibitor
0,320	0,103	Vasodilator, coronary
0,253	0,036	Acaricide
0,278	0,061	Gluconolactonase inhibitor
0,267	0,051	Glutarate-semialdehyde dehydrogenase inhibitor
0,246	0,030	Bile-salt sulfotransferase inhibitor
0,331	0,115	GST A substrate
0,295	0,079	N-formylmethionyl-peptidase inhibitor
0,291	0,075	IgA-specific serine endopeptidase inhibitor
0,281	0,066	Glycerol-3-phosphate dehydrogenase inhibitor
0,268	0,054	Pediculicide
0,345	0,132	Aminobutyraldehyde dehydrogenase inhibitor
0,276	0,063	HIV attachment inhibitor
0,277	0,064	Fructan beta-fructosidase inhibitor
0,308	0,096	MMP9 expression inhibitor
0,242	0,031	Mucolytic
0,256	0,044	Cellulose 1,4-beta-cellobiosidase inhibitor
0,279	0,067	Ophthalmic drug
0,288	0,078	CYP2C11 substrate
0,295	0,085	Creatininase inhibitor
0,339	0,129	Sulfur reductase inhibitor
0,249	0,039	Protein synthesis inhibitor
0,264	0,054	Envelysin inhibitor
0,263	0,054	Antiprotozoal (Trichomonas)
0,261	0,052	Dopamine release stimulant
0,248	0,040	Aureolysin inhibitor
0,315	0,108	Intermittent claudication treatment

0,326	0,121	JAK2 expression inhibitor
0,255	0,049	Antiparkinsonian, tremor relieving
0,286	0,082	Isopenicillin-N epimerase inhibitor
0,341	0,137	Oxygen scavenger
0,283	0,079	Inotropic
0,289	0,086	Nitrite reductase [NAD(P)H] inhibitor
0,302	0,100	Bilirubin oxidase inhibitor
0,259	0,057	Cutinase inhibitor
0,257	0,055	Antipruritic, non-allergic
0,364	0,163	Proteasome ATPase inhibitor
0,222	0,021	Estradiol 17alpha-dehydrogenase inhibitor
0,257	0,058	CDP-diacylglycerol-glycerol-3-phosphate 3-phosphatidyltransferase inhibitor
0,252	0,053	Alpha-1,6-mannosyl-glycoprotein 4-beta-N-acetylglucosaminyltransferase inhibitor
0,282	0,084	Methylumbelliferyl-acetate deacetylase inhibitor
0,310	0,113	Malate dehydrogenase (acceptor) inhibitor
0,293	0,096	Prostaglandin-A1 DELTA-isomerase inhibitor
0,296	0,099	Alkane 1-monooxygenase inhibitor
0,216	0,019	Beta-glucosidase inhibitor
0,236	0,039	Undecaprenyl-diphosphatase inhibitor
0,324	0,127	Vasodilator, peripheral
0,241	0,045	Hydroxysteroid dehydrogenase inhibitor
0,241	0,045	Bisphosphoglycerate mutase inhibitor
0,217	0,022	Creatinase inhibitor
0,312	0,119	3-Hydroxybenzoate 6-monooxygenase inhibitor
0,309	0,117	Aspartate-phenylpyruvate transaminase inhibitor
0,257	0,065	DNA ligase (ATP) inhibitor
0,214	0,023	Gaucher disease treatment
0,308	0,118	Cl--transporting ATPase inhibitor
0,272	0,081	Aspergillus nuclease S1 inhibitor
0,234	0,044	Aryldialkylphosphatase inhibitor
0,246	0,056	N-(long-chain-acyl)ethanolamine deacylase inhibitor
0,222	0,032	Allantoate deiminase inhibitor
0,222	0,033	Ganglioside galactosyltransferase inhibitor
0,257	0,069	Ethanolamine-phosphate cytidylyltransferase inhibitor
0,306	0,119	CYP3A5 substrate
0,246	0,059	Pappalysin-1 inhibitor
0,296	0,110	CYP2E1 inducer

0,299	0,113	Malate oxidase inhibitor
0,293	0,108	TNF expression inhibitor
0,230	0,046	Expectorant
0,218	0,034	Saccharolysin inhibitor
0,296	0,113	Histidine N-acetyltransferase inhibitor
0,268	0,085	Phosphatidylserine decarboxylase inhibitor
0,217	0,035	Antiglaucomic
0,254	0,072	Trimethylamine dehydrogenase inhibitor
0,246	0,063	N-acetyllactosamine synthase inhibitor
0,195	0,013	5 Hydroxytryptamine 1A agonist
0,247	0,065	Levanase inhibitor
0,348	0,166	Taurine dehydrogenase inhibitor
0,226	0,044	Antianorexic
0,227	0,047	Alpha-amylase inhibitor
0,231	0,052	Poly(alpha-L-guluronate) lyase inhibitor
0,208	0,029	Psychosexual dysfunction treatment
0,245	0,066	Dimethylmaleate hydratase inhibitor
0,212	0,033	Protein-S-isoprenylcysteine O-methyltransferase inhibitor
0,238	0,059	Protein-Npi-phosphohistidine-sugar phosphotransferase inhibitor
0,217	0,039	Deoxyribonuclease I inhibitor
0,217	0,038	Antihypotensive
0,221	0,043	Galactose oxidase inhibitor
0,205	0,028	Adenylate cyclase inhibitor
0,284	0,107	Pro-opiomelanocortin converting enzyme inhibitor
0,305	0,127	EIF4E expression inhibitor
0,238	0,061	Ornithine cyclodeaminase inhibitor
0,240	0,063	UGT2B28 substrate
0,298	0,122	Superoxide dismutase inhibitor
0,262	0,086	Anesthetic general
0,234	0,058	N-Acyl-D-aspartate deacylase inhibitor
0,309	0,135	Antipruritic, allergic
0,241	0,066	Sulfite reductase inhibitor
0,227	0,053	Lipocortins synthesis antagonist
0,260	0,086	CYP2C29 substrate
0,254	0,080	NF-E2-related factor 2 stimulant
0,207	0,033	Glycerone-phosphate O-acyltransferase inhibitor
0,278	0,105	Antiviral (Influenza)

0,257	0,084	2-Hydroxymuconate-semialdehyde hydrolase inhibitor
0,233	0,060	Phospholipase A1 inhibitor
0,229	0,057	D-alanine 2-hydroxymethyltransferase inhibitor
0,299	0,127	Spermidine dehydrogenase inhibitor
0,221	0,050	Snapalysin inhibitor
0,220	0,050	Tropinesterase inhibitor
0,233	0,063	Fumarate reductase (NADH) inhibitor
0,235	0,066	Oryzin inhibitor
0,257	0,088	Flavin-containing monooxygenase substrate
0,259	0,090	RNA synthesis inhibitor
0,237	0,068	Di-trans,poly-cis-decaprenylcistransferase inhibitor
0,258	0,089	CYP2B10 substrate
0,330	0,162	Glyceryl-ether monooxygenase inhibitor
0,223	0,054	Dynein ATPase inhibitor
0,207	0,039	Mannan endo-1,6-alpha-mannosidase inhibitor
0,300	0,133	CYP4A11 substrate
0,213	0,045	CYP2B1 substrate
0,296	0,129	Amine dehydrogenase inhibitor
0,236	0,070	Carboxypeptidase D inhibitor
0,248	0,082	Transcription factor NF kappa A inhibitor
0,218	0,053	Rhizopuspepsin inhibitor
0,268	0,103	3-Phytase inhibitor
0,372	0,207	Calcium channel (voltage-sensitive) activator
0,180	0,016	Urolithiasis treatment
0,273	0,109	FMO1 substrate
0,279	0,115	RELA expression inhibitor
0,243	0,079	Aspartate-ammonia ligase inhibitor
0,178	0,014	4-Carboxymethyl-4-methylbutenolide mutase inhibitor
0,246	0,082	Mood disorders treatment
0,239	0,075	Quinoprotein glucose dehydrogenase inhibitor
0,237	0,074	Antiperistaltic
0,288	0,126	Endopeptidase So inhibitor
0,207	0,045	3-Chloro-D-alanine dehydrochlorinase inhibitor
0,219	0,057	Guanosine-3',5'-bis(diphosphate) 3'-diphosphatase inhibitor
0,211	0,049	GABA B receptor agonist
0,283	0,122	Manganese peroxidase inhibitor
0,225	0,064	Interleukin 10 agonist

0,269	0,109	Alcohol dehydrogenase (acceptor) inhibitor
0,210	0,051	Tentoxilysin inhibitor
0,241	0,082	Antidepressant
0,213	0,054	Mitochondrial intermediate peptidase inhibitor
0,292	0,134	P-glycoprotein substrate
0,218	0,061	Polygalacturonase inhibitor
0,290	0,133	S-formylglutathione hydrolase inhibitor
0,180	0,023	Glycosylphosphatidylinositol diacylglycerol-lyase inhibitor
0,217	0,060	Peptidyl-dipeptidase B inhibitor
0,252	0,095	Monoamine uptake inhibitor
0,264	0,107	Chenodeoxycholoyltaurine hydrolase inhibitor
0,224	0,069	3-Methylbutanal reductase inhibitor
0,244	0,089	DNA synthesis inhibitor
0,198	0,042	Endo-1,3(4)-beta-glucanase inhibitor
0,194	0,039	Diisopropyl-fluorophosphatase inhibitor
0,203	0,048	Gametolysin inhibitor
0,257	0,103	CYP4A substrate
0,253	0,099	Peptide alpha-N-acetyltransferase inhibitor
0,294	0,141	Caspase 3 stimulant
0,200	0,048	Alpha-Methylacyl-CoA racemase inhibitor
0,221	0,068	2-Oxoaldehyde dehydrogenase (NADP+) inhibitor
0,204	0,051	1,4-Alpha-glucan branching enzyme inhibitor
0,191	0,039	Phosphoinositide phospholipase C inhibitor
0,220	0,068	Cyclooxygenase substrate
0,217	0,067	Retinal dehydrogenase inhibitor
0,188	0,037	3-Ketovalidoxylamine C-N-lyase inhibitor
0,234	0,084	Cyclic AMP modulator
0,160	0,009	Alpha-L-fucosidase inhibitor
0,177	0,027	Trehalose-phosphatase inhibitor
0,201	0,051	D-xylulose reductase inhibitor
0,214	0,064	D-threo-aldose 1-dehydrogenase inhibitor
0,255	0,105	Antimetastatic
0,259	0,109	Antiinflammatory, intestinal
0,240	0,090	Antialcoholic
0,186	0,037	Galacturan 1,4-alpha-galacturonidase inhibitor
0,177	0,028	Adenylate cyclase V inhibitor
0,229	0,080	Antineoplastic (brain cancer)

0,156	0,008	Squalene-hopene cyclase inhibitor
0,203	0,054	Isopenicillin-N synthase inhibitor
0,233	0,085	Transactivator transcription protein inhibitor
0,300	0,153	Antineoplastic
0,334	0,187	Antiviral (Rhinovirus)
0,185	0,038	2,2-Dialkylglycine decarboxylase (pyruvate) inhibitor
0,253	0,107	Phosphopantothenoylcysteine decarboxylase inhibitor
0,224	0,078	Nardilysin inhibitor
0,241	0,096	Sulfite oxidase inhibitor
0,229	0,084	Glutaminyl-peptide cyclotransferase inhibitor
0,252	0,107	Tyrosine 3 hydroxylase inhibitor
0,275	0,130	CYP3A4 inducer
0,162	0,017	DELTA24-sterol reductase inhibitor
0,298	0,153	Pancreatic elastase inhibitor
0,265	0,120	CYP17 inhibitor
0,229	0,085	Linoleoyl-CoA desaturase inhibitor
0,239	0,095	Transketolase inhibitor
0,267	0,123	CYP3A inducer
0,209	0,066	Sodium channel blocker class Ib
0,155	0,012	Gentamicin 2''-nucleotidyltransferase inhibitor
0,247	0,104	Chitosanase inhibitor
0,193	0,051	5 Hydroxytryptamine 7 agonist
0,194	0,052	Aldehyde dehydrogenase (NADP+) inhibitor
0,174	0,034	Pectin lyase inhibitor
0,280	0,139	Apoptosis agonist
0,184	0,045	Retinyl-palmitate esterase inhibitor
0,170	0,031	Alpha-N-acetylgalactosaminidase inhibitor
0,146	0,007	Protein 30S ribosomal subunit inhibitor
0,327	0,188	TP53 expression enhancer
0,187	0,049	Ligase inhibitor
0,192	0,054	2-Acylglycerol O-acyltransferase inhibitor
0,227	0,090	NADH kinase inhibitor
0,173	0,037	Histamine agonist
0,245	0,109	CYP2A5 substrate
0,221	0,085	GST M substrate
0,222	0,086	Glycine amidinotransferase inhibitor
0,270	0,135	Cyanoalanine nitrilase inhibitor

0,163	0,028	CYP3A5 inducer
0,173	0,039	N4-(beta-N-acetylglucosaminyl)-L-asparaginase inhibitor
0,219	0,085	Aspergillopepsin II inhibitor
0,184	0,051	Antitussive, narcotic
0,268	0,134	Glucose oxidase inhibitor
0,257	0,124	Antiviral (Herpes)
0,188	0,055	Inulinase inhibitor
0,193	0,060	Antirickettsial
0,297	0,165	Oxidoreductase inhibitor
0,206	0,074	UGT2B17 substrate
0,271	0,139	Antihelmintic (Nematodes)
0,159	0,026	Demethylsterigmatocystin 6-O-methyltransferase inhibitor
0,188	0,058	Lactaldehyde reductase inhibitor
0,236	0,106	Skeletal muscle relaxant
0,168	0,038	Riboflavin phosphotransferase inhibitor
0,179	0,049	Polar-amino-acid-transporting ATPase inhibitor
0,203	0,073	Prenyl-diphosphatase inhibitor
0,215	0,085	Methanol dehydrogenase inhibitor
0,188	0,058	tRNA nucleotidyltransferase inhibitor
0,200	0,071	Vitamin-K-epoxide reductase (warfarin-insensitive) inhibitor
0,225	0,096	Muscle relaxant
0,317	0,188	Thromboxane B2 antagonist
0,156	0,027	Narcotic antagonist
0,162	0,033	1-Aminocyclopropane-1-carboxylate deaminase inhibitor
0,206	0,077	Horrilysin inhibitor
0,278	0,149	Biotinidase inhibitor
0,173	0,044	Valine decarboxylase inhibitor
0,176	0,047	Protein synthesis stimulant
0,279	0,151	Pterin deaminase inhibitor
0,179	0,051	Methyltransferase substrate
0,187	0,059	ADP-ribosylarginine hydrolase inhibitor
0,157	0,030	Choline dehydrogenase inhibitor
0,217	0,091	[acyl-carrier-protein] S-acetyltransferase inhibitor
0,168	0,042	Glycopeptide alpha-N-acetylgalactosaminidase inhibitor
0,171	0,045	Pancreatic endopeptidase E inhibitor
0,183	0,057	Choline-sulfatase inhibitor
0,175	0,050	Sulfur dioxygenase inhibitor

0,206	0,082	Carminative
0,263	0,138	CYP3A3 substrate
0,148	0,024	Shab potassium channel blocker
0,171	0,047	2-Dehydropantoate aldolase inhibitor
0,199	0,075	Procollagen N-endopeptidase inhibitor
0,240	0,117	4-Methoxybenzoate monooxygenase (O-demethylating) inhibitor
0,167	0,044	Lysyl endopeptidase inhibitor
0,233	0,110	Aldehyde dehydrogenase (pyrroloquinoline-quinone) inhibitor
0,158	0,037	Pregnane X receptor agonist
0,167	0,046	Furin inhibitor
0,297	0,178	Thioredoxin inhibitor
0,270	0,151	Linoleate diol synthase inhibitor
0,179	0,060	Antineoplastic (renal cancer)
0,247	0,128	4-Hydroxyproline epimerase inhibitor
0,168	0,049	Ferredoxin-NADP+ reductase inhibitor
0,313	0,195	Fibrinolytic
0,182	0,064	Plasmanylethanolamine desaturase inhibitor
0,266	0,148	Leukotriene-C4 synthase inhibitor
0,226	0,109	Arylmalonate decarboxylase inhibitor
0,184	0,066	Sterol 3-beta-glucosyltransferase inhibitor
0,218	0,101	Chloride channel activator
0,191	0,074	(S)-3-amino-2-methylpropionate transaminase inhibitor
0,160	0,043	Antibacterial, ophthalmic
0,255	0,138	Antiprotozoal (Leishmania)
0,194	0,077	Phosphatidylglycerophosphatase inhibitor
0,216	0,101	Phosphatidylcholine-sterol O-acyltransferase inhibitor
0,121	0,006	Dopamine D2A antagonist
0,179	0,064	NAD+ synthase (glutamine-hydrolysing) inhibitor
0,265	0,150	Transcription factor stimulant
0,265	0,150	Transcription factor NF kappa B stimulant
0,244	0,129	Aldehyde oxidase inhibitor
0,176	0,062	Antineoplastic (thyroid cancer)
0,152	0,038	Lysozyme inhibitor
0,193	0,079	Crotonoyl-[acyl-carrier-protein] hydratase inhibitor
0,229	0,116	Pyruvate decarboxylase inhibitor
0,196	0,083	Cyclopropane-fatty-acyl-phospholipid synthase inhibitor
0,169	0,056	Glycerol-1-phosphatase inhibitor

0,249	0,136	Dehydro-L-gulonate decarboxylase inhibitor
0,214	0,102	Coccolysin inhibitor
0,196	0,084	Ubiquitin thiolesterase inhibitor
0,180	0,068	Gamma-butyrobetaine dioxygenase inhibitor
0,223	0,112	Lipid peroxidase inhibitor
0,336	0,225	Nicotinic alpha4beta4 receptor agonist
0,190	0,080	Indoleacetaldoxime dehydratase inhibitor
0,165	0,054	Alcohol dehydrogenase inhibitor
0,183	0,072	Glutamate 5-kinase inhibitor
0,197	0,087	CYP2A3 substrate
0,204	0,094	Cyclohexyl-isocyanide hydratase inhibitor
0,217	0,107	DNA-3-methyladenine glycosylase I inhibitor
0,186	0,077	Antinaupathic
0,126	0,017	Sodium/hydrogen exchanger 5 inhibitor
0,225	0,116	Gingipain K inhibitor
0,255	0,146	Lipoprotein lipase inhibitor
0,215	0,106	UGT2B4 substrate
0,189	0,081	Tauropine dehydrogenase inhibitor
0,164	0,056	Phosphoenolpyruvate-protein phosphotransferase inhibitor
0,141	0,033	2-Aminoethylphosphonate-pyruvate transaminase inhibitor
0,213	0,106	Arylesterase inhibitor
0,116	0,009	Alpha-mannosidase inhibitor
0,237	0,130	Antiamyloidogenic
0,202	0,094	Bontoxilysin inhibitor
0,256	0,149	HCV IRES inhibitor
0,159	0,052	Endoglycosylceramidase inhibitor
0,125	0,018	CC chemokine 4 receptor antagonist
0,182	0,075	Sweetener
0,175	0,068	Stromelysin 2 inhibitor
0,147	0,040	(S)-carnitine 3-dehydrogenase inhibitor
0,158	0,052	Pectate lyase inhibitor
0,217	0,111	Methane monooxygenase inhibitor
0,138	0,033	Beta-D-fucosidase inhibitor
0,121	0,016	Imidazoleglycerol-phosphate dehydratase inhibitor
0,235	0,131	Arylsulfate sulfotransferase inhibitor
0,234	0,130	Antiviral (Influenza A)
0,166	0,062	3-Carboxyethylcatechol 2,3-dioxygenase inhibitor

0,162	0,058	UGT2B18 substrate
0,211	0,108	NOS2 expression inhibitor
0,211	0,108	CYP2B substrate
0,184	0,081	UGT2B10 substrate
0,165	0,063	Lipotropic
0,186	0,083	Rhodotorulapepsin inhibitor
0,163	0,060	Adenylate cyclase stimulant
0,204	0,101	Diamine N-acetyltransferase inhibitor
0,155	0,053	Restless leg syndrome treatment
0,175	0,072	Alpha-glucuronidase inhibitor
0,222	0,120	CYP2C6 substrate
0,175	0,073	Nitric oxide scavenger
0,182	0,080	Anesthetic
0,172	0,070	Aspartoacylase inhibitor
0,197	0,096	Gamma-D-Glutamyl-meso-diaminopimelate peptidase inhibitor
0,163	0,063	Glutamate (mGluR5) agonist
0,157	0,057	Cellulase inhibitor
0,141	0,042	Heparan-alpha-glucosaminide N-acetyltransferase inhibitor
0,187	0,087	Serine-pyruvate transaminase inhibitor
0,238	0,139	(S)-6-hydroxynicotine oxidase inhibitor
0,197	0,098	Nucleoside oxidase (H2O2-forming) inhibitor
0,201	0,102	1,2-alpha-L-fucosidase inhibitor
0,248	0,150	Immunosuppressant
0,131	0,033	Phosphatidylinositol 3-kinase stimulant
0,162	0,064	Glucan 1,4-beta-glucosidase inhibitor
0,226	0,128	Glutamate-tRNA ligase inhibitor
0,143	0,045	Cholate-CoA ligase inhibitor
0,271	0,173	Muramoyltetrapeptide carboxypeptidase inhibitor
0,126	0,028	Arabinose isomerase inhibitor
0,153	0,056	BRAF expression inhibitor
0,206	0,108	CYP2B2 substrate
0,114	0,017	Acetylcholine M3 receptor agonist
0,146	0,050	Agmatinase inhibitor
0,150	0,054	N-(5-amino-5-carboxypentanoyl)-L-cysteinyl-D-valine synthase inhibitor
0,272	0,176	(R)-6-hydroxynicotine oxidase inhibitor
0,182	0,086	Nicotinic alpha4beta2 receptor antagonist
0,141	0,045	Aldehyde dehydrogenase [NAD(P)+] inhibitor

0,141	0,045	Choloylglycine hydrolase inhibitor
0,221	0,126	Choline-phosphate cytidylyltransferase inhibitor
0,234	0,139	L-glutamate oxidase inhibitor
0,190	0,096	Bisphosphoglycerate phosphatase inhibitor
0,141	0,047	CYP19 inhibitor
0,179	0,085	Arachidonic acid antagonist
0,247	0,153	Lysyl oxidase inhibitor
0,144	0,050	Obsessive-compulsive disorder treatment
0,169	0,075	Serratia marcescens nuclease inhibitor
0,195	0,102	Meprin B inhibitor
0,248	0,155	Mitochondrial processing peptidase inhibitor
0,156	0,064	Aspartate-tRNA ligase inhibitor
0,162	0,070	Farnesyltranstransferase inhibitor
0,098	0,006	Antibiotic Aminoglycoside-like
0,222	0,131	N-benzyloxycarbonylglycine hydrolase inhibitor
0,174	0,084	Polyneuridine-aldehyde esterase inhibitor
0,216	0,126	4-Nitrophenylphosphatase inhibitor
0,186	0,097	Polarisation inhibitor
0,133	0,043	Glutamate release inhibitor
0,164	0,075	Sphinganine-1-phosphate aldolase inhibitor
0,160	0,070	Trans-pentaprenyltranstransferase inhibitor
0,189	0,100	Spasmolytic, Papaverin-like
0,136	0,047	Trimethyllysine dioxygenase inhibitor
0,239	0,150	CYP2A8 substrate
0,213	0,124	Phospholipase C inhibitor
0,187	0,099	Morphine 6-dehydrogenase inhibitor
0,132	0,044	Ribonuclease inhibitor
0,168	0,080	UGT1A5 substrate
0,151	0,063	Peristaltic stimulant
0,205	0,117	Antifibrinolytic
0,270	0,182	CYP2C8 substrate
0,117	0,031	Galactosylgalactosylglucosylceramidase inhibitor
0,157	0,071	N-Acyl-D-amino-acid deacylase inhibitor
0,130	0,043	Cytochrome-c3 hydrogenase inhibitor
0,124	0,037	Squalene epoxidase inhibitor
0,221	0,135	Naphthalene 1,2-dioxygenase inhibitor
0,221	0,135	Ferredoxin-NAD+ reductase inhibitor

0,253	0,167	Polarisation stimulant
0,117	0,032	Acetylcholine M2 receptor agonist
0,194	0,109	Opheline kinase inhibitor
0,194	0,109	Taurocyamine kinase inhibitor
0,202	0,117	Endopeptidase La inhibitor
0,132	0,048	Secretase stimulant
0,132	0,048	Secretase alpha stimulant
0,150	0,065	Insecticide
0,145	0,061	Aminocarboxymuconate-semialdehyde decarboxylase inhibitor
0,190	0,106	Ceramide glucosyltransferase inhibitor
0,137	0,053	Serine 3-dehydrogenase inhibitor
0,097	0,013	Mannosidase inhibitor
0,107	0,023	Fatty-acyl-ethyl-ester synthase inhibitor
0,109	0,026	5 Hydroxytryptamine 1 agonist
0,192	0,109	Thiol oxidase inhibitor
0,142	0,059	Selenocysteine lyase inhibitor
0,220	0,138	DNA-(apurinic or apyrimidinic site) lyase inhibitor
0,087	0,004	FtsZ polymerization inhibitor
0,163	0,081	Glyoxylate reductase (NADP+) inhibitor
0,109	0,027	High-mannose-oligosaccharide beta-1,4-N-acetylglucosaminyltransferase inhibitor
0,163	0,082	6-Pyruvoyltetrahydropterin synthase inhibitor
0,143	0,062	Alpha-N-arabinofuranosidase inhibitor
0,182	0,101	Alanine-tRNA ligase inhibitor
0,111	0,030	Homoserine O-succinyltransferase inhibitor
0,144	0,064	Dolichyl-phosphatase inhibitor
0,120	0,040	Lysine decarboxylase inhibitor
0,098	0,018	Anesthetic inhalation
0,106	0,027	Amylo-alpha-1,6-glucosidase inhibitor
0,136	0,056	Carnitine dehydratase inhibitor
0,163	0,083	Styrene-oxide isomerase inhibitor
0,114	0,034	Ca2+-transporting ATPase inhibitor
0,104	0,025	Polyamine biosynthesis inhibitor
0,211	0,132	4-Coumarate-CoA ligase inhibitor
0,168	0,089	Myeloblastin inhibitor
0,157	0,079	CYP2B18 substrate
0,158	0,081	UGT2B9 substrate
0,251	0,174	Antiinflammatory, ophthalmic

0,208	0,131	tRNA-pseudouridine synthase I inhibitor
0,167	0,090	Leucine dehydrogenase inhibitor
0,270	0,193	Nicotine dehydrogenase inhibitor
0,144	0,067	Protein-tyrosine sulfotransferase inhibitor
0,166	0,089	Uterine stimulant
0,130	0,054	Cholesterol oxidase inhibitor
0,187	0,112	Diabetic nephropathy treatment
0,216	0,141	Antiviral (CMV)
0,155	0,081	Acetylspermidine deacetylase inhibitor
0,168	0,094	Cyclopentanone monooxygenase inhibitor
0,138	0,064	Allantoinase inhibitor
0,121	0,047	4-Alpha-glucanotransferase inhibitor
0,141	0,067	Valine-tRNA ligase inhibitor
0,253	0,179	Nitrate reductase (cytochrome) inhibitor
0,189	0,115	Peroxidase substrate
0,177	0,104	Interleukin 6 antagonist
0,121	0,048	Rhodopsin kinase inhibitor
0,271	0,198	Pin1 inhibitor
0,168	0,095	Vascular adhesion protein 1 inhibitor
0,249	0,177	Centromere associated protein inhibitor
0,105	0,032	Homospermidine synthase inhibitor
0,221	0,149	HMOX1 expression enhancer
0,106	0,034	L-fuconate dehydratase inhibitor
0,134	0,062	Glucan 1,6-alpha-glucosidase inhibitor
0,132	0,060	CYP19 substrate
0,189	0,117	Glutathione dehydrogenase (ascorbate) inhibitor
0,129	0,057	Diacylglycerol cholinephosphotransferase inhibitor
0,153	0,082	Allyl-alcohol dehydrogenase inhibitor
0,115	0,043	Diaminopropionate ammonia-lyase inhibitor
0,145	0,074	Testosterone 17beta-dehydrogenase inhibitor
0,134	0,063	Glutamate-1-semialdehyde 2,1-aminomutase inhibitor
0,200	0,130	Cathepsin T inhibitor
0,094	0,023	Alpha,alpha-trehalose phosphorylase inhibitor
0,119	0,049	2-Aminohexano-6-lactam racemase inhibitor
0,133	0,063	Protein-glucosylgalactosylhydroxylysine glucosidase inhibitor
0,161	0,091	Microtubule formation inhibitor
0,113	0,043	Globoside alpha-N-acetylgalactosaminyltransferase inhibitor

0,108	0,038	Ribonuclease U2 inhibitor
0,109	0,039	Beta-amylase inhibitor
0,156	0,086	Carnosine synthase inhibitor
0,083	0,013	Glutamate (mGluR3) agonist
0,139	0,071	Corticosteroid antagonist
0,132	0,064	(S)-2-hydroxy-acid oxidase inhibitor
0,119	0,051	Site-specific DNA-methyltransferase (adenine-specific) inhibitor
0,184	0,116	Alcohol dehydrogenase [NAD(P)+] inhibitor
0,120	0,053	Hyperprolactinemia treatment
0,116	0,049	Ribitol 2-dehydrogenase inhibitor
0,135	0,068	Thiamine-phosphate kinase inhibitor
0,210	0,143	Antiviral (Poxvirus)
0,072	0,005	GHS receptor antagonist
0,103	0,036	Neolactotetraosylceramide alpha-2,3-sialyltransferase inhibitor
0,122	0,056	Glycerol-3-phosphate O-acyltransferase inhibitor
0,106	0,040	Ribose-5-phosphate-ammonia ligase inhibitor
0,112	0,046	Glutamine-pyruvate transaminase inhibitor
0,144	0,079	Acylaminoacyl-peptidase inhibitor
0,154	0,089	Anthranilate-CoA ligase inhibitor
0,108	0,043	UDP-N-acetylglucosamine diphosphorylase inhibitor
0,180	0,115	Acyl-CoA oxidase inhibitor
0,153	0,088	2,4-Dichlorophenol 6-monooxygenase inhibitor
0,082	0,018	Acetylcholine muscarinic agonist
0,088	0,023	2,3-Oxidosqualene-lanosterol cyclase inhibitor
0,222	0,158	Cardioprotectant
0,115	0,051	NMDA receptor polyamine site antagonist
0,098	0,034	11-Cis-retinyl-palmitate hydrolase inhibitor
0,135	0,071	Ethanolaminephosphotransferase inhibitor
0,141	0,077	Acetylenecarboxylate hydratase inhibitor
0,116	0,052	Succinate dehydrogenase inhibitor
0,297	0,233	CYP2C8 inhibitor
0,112	0,049	Inositol-polyphosphate 5-phosphatase inhibitor
0,112	0,049	Pyridoxal kinase inhibitor
0,129	0,066	Polyamine oxidase inhibitor
0,200	0,138	CYP2C3 substrate
0,096	0,034	5 Hydroxytryptamine 1A antagonist
0,117	0,055	D-Ornithine 4,5-aminomutase inhibitor

0,205	0,143	CYP2F1 substrate
0,098	0,036	Amino-acid racemase inhibitor
0,136	0,074	GABA inverse agonist
0,136	0,074	GABA A inverse agonist
0,110	0,048	Cytokine modulator
0,109	0,048	Glycerophosphocholine cholinephosphodiesterase inhibitor
0,106	0,045	Antineoplastic antibiotic
0,086	0,025	Beta galactosidase inhibitor
0,293	0,232	Diabetic neuropathy treatment
0,124	0,064	Methionine decarboxylase inhibitor
0,120	0,060	Endo-1,4-beta-xylanase inhibitor
0,145	0,085	Homoserine dehydrogenase inhibitor
0,157	0,097	Sedoheptulose-bisphosphatase inhibitor
0,124	0,064	Mevalonate kinase inhibitor
0,231	0,172	Glutathione thiolesterase inhibitor
0,134	0,075	Glucuronolactone reductase inhibitor
0,126	0,067	Acyl-lysine deacylase inhibitor
0,125	0,066	2-Oxoisovalerate dehydrogenase (acylating) inhibitor
0,108	0,049	Tyrosine-protein kinase TYRO 10 inhibitor
0,113	0,054	Putrescine oxidase inhibitor
0,183	0,125	Aromatic-hydroxylamine O-acetyltransferase inhibitor
0,233	0,174	Antiinfective
0,141	0,083	Procollagen C-endopeptidase inhibitor
0,084	0,026	Arabinogalactan endo-1,4-beta-galactosidase inhibitor
0,131	0,073	Gingipain R inhibitor
0,112	0,054	Glyceraldehyde-3-phosphate dehydrogenase (phosphorylating) inhibitor
0,125	0,068	Thiol S-methyltransferase inhibitor
0,154	0,097	Antineoplastic (lung cancer)
0,137	0,080	CYP2E1 inhibitor
0,177	0,121	Antihematotoxic
0,188	0,132	Acetylserotonin O-methyltransferase inhibitor
0,142	0,085	Aminopeptidase Y inhibitor
0,124	0,068	Glucan endo-1,3-alpha-glucosidase inhibitor
0,122	0,067	Deoxyribose-phosphate aldolase inhibitor
0,070	0,015	CYP24 substrate
0,090	0,035	Alpha-1,6-mannosyl-glycoprotein 6-beta-N-acetylglucosaminyltransferase inhibitor
0,109	0,054	Gluconate dehydratase inhibitor

0,159	0,104	Metallocarboxypeptidase D inhibitor
0,117	0,062	Xylose isomerase inhibitor
0,110	0,056	Aerobactin synthase inhibitor
0,184	0,130	Dactylysin inhibitor
0,112	0,057	Omega-amidase inhibitor
0,181	0,127	Prolyl aminopeptidase inhibitor
0,132	0,078	Cerebroside-sulfatase inhibitor
0,119	0,067	Neuropsin inhibitor
0,117	0,064	Glucarate dehydratase inhibitor
0,090	0,038	Alpha 2a adrenoreceptor antagonist
0,096	0,043	5 Hydroxytryptamine agonist
0,106	0,054	Leucine-tRNA ligase inhibitor
0,111	0,059	Pantothenase inhibitor
0,119	0,067	Carboxymethylenebutenolidase inhibitor
0,094	0,042	Shock treatment
0,112	0,060	D-arabinonolactone oxidase inhibitor
0,155	0,104	Maleate isomerase inhibitor
0,152	0,100	UGT2B7 substrate
0,083	0,032	Peptidoglycan beta-N-acetylmuramidase inhibitor
0,093	0,042	Argininosuccinate lyase inhibitor
0,124	0,073	CYP26A substrate
0,131	0,080	CYP4B substrate
0,101	0,050	Heme oxygenase inhibitor
0,076	0,025	NMDA receptor polyamine site agonist
0,110	0,059	Allantoicase inhibitor
0,246	0,195	Muscular dystrophy treatment
0,091	0,041	N-acetylglucosamine-6-phosphate deacetylase inhibitor
0,116	0,066	Dethiobiotin synthase inhibitor
0,149	0,099	Antimycoplasmal
0,211	0,161	CYP2B6 substrate
0,115	0,066	Aspartate kinase inhibitor
0,095	0,046	Oligo-1,6-glucosidase inhibitor
0,114	0,065	L-lysine 6-transaminase inhibitor
0,205	0,156	CYP2C18 substrate
0,062	0,013	Neuraminidase inhibitor
0,134	0,085	Peptidylamidoglycolate lyase inhibitor
0,107	0,058	Sorbose dehydrogenase inhibitor

0,120	0,071	Cocain dependency treatment
0,138	0,089	IgA-specific metalloendopeptidase inhibitor
0,222	0,173	Nicotinate dehydrogenase inhibitor
0,117	0,069	ATP adenylyltransferase inhibitor
0,113	0,065	Arginine deiminase inhibitor
0,119	0,071	UDP-N-acetylglucosamine-dolichyl-phosphate N-acetylglucosaminephosphotransferase inhibitor
0,097	0,050	Proline dehydrogenase inhibitor
0,119	0,072	Carnitine 3-dehydrogenase inhibitor
0,148	0,101	Rhamnulose-1-phosphate aldolase inhibitor
0,202	0,155	Cell wall biosynthesis inhibitor
0,107	0,061	Aminoacylase inhibitor
0,125	0,078	Tardive dyskinesia treatment
0,151	0,105	Male reproductive disfunction treatment
0,181	0,134	TRPA1 agonist
0,106	0,060	Phosphatidate cytidylyltransferase inhibitor
0,140	0,094	Trans-2-enoyl-CoA reductase (NAD+) inhibitor
0,140	0,094	Pyruvate dehydrogenase (cytochrome) inhibitor
0,104	0,058	Poly(3-hydroxybutyrate) depolymerase inhibitor
0,115	0,069	5 Hydroxytryptamine 4A antagonist
0,147	0,102	N-acetyl-gamma-glutamyl-phosphate reductase inhibitor
0,119	0,074	D-Octopine dehydrogenase inhibitor
0,140	0,094	Oxalate oxidase inhibitor
0,166	0,120	Opine dehydrogenase inhibitor
0,205	0,160	Cytochrome-b5 reductase inhibitor
0,114	0,068	NADH dehydrogenase inhibitor
0,151	0,106	Aldehyde dehydrogenase 2 substrate
0,130	0,085	CDP-diacylglycerol-inositol 3-phosphatidyltransferase inhibitor
0,080	0,035	Formimidoylglutamase inhibitor
0,125	0,081	Galactokinase inhibitor
0,175	0,131	Protein-synthesizing GTPase inhibitor
0,116	0,072	Diacylglycerol kinase inhibitor
0,072	0,028	Alpha-1,6-mannosyl-glycoprotein 2-beta-N-acetylglucosaminyltransferase inhibitor
0,100	0,057	Acetylcholine agonist
0,190	0,147	Succinate-semialdehyde dehydrogenase [NAD(P)+] inhibitor
0,111	0,068	UGT2B11 substrate
0,103	0,060	Ornithine-oxo-acid transaminase inhibitor
0,159	0,117	Urate-ribonucleotide phosphorylase inhibitor

0,081	0,039	CYP4F12 substrate
0,125	0,083	Anesthetic local
0,130	0,087	Antineoplastic (gastric cancer)
0,076	0,034	3-Alpha-hydroxysteroid dehydrogenase (B-specific) inhibitor
0,145	0,102	3-Hydroxybutyryl-CoA dehydrogenase inhibitor
0,145	0,102	2,4-Diaminopentanoate dehydrogenase inhibitor
0,145	0,102	Lysine 6-dehydrogenase inhibitor
0,091	0,049	Phosphatidylglycerol-membrane-oligosaccharide glycerophosphotransferase inhibitor
0,113	0,071	D-2-hydroxy-acid dehydrogenase inhibitor
0,153	0,111	GABA C receptor agonist
0,154	0,112	Prunasin beta-glucosidase inhibitor
0,130	0,089	Gly-X carboxypeptidase inhibitor
0,143	0,102	CYP2C8 inducer
0,073	0,031	Chitin deacetylase inhibitor
0,124	0,083	N-acetylneuraminate synthase inhibitor
0,101	0,059	Thermomycolin inhibitor
0,081	0,040	4a-Hydroxytetrahydrobiopterin dehydratase inhibitor
0,085	0,043	Alpha glucosidase inhibitor
0,096	0,055	Bis(5'-adenosyl)-triphosphatase inhibitor
0,066	0,025	Glucan 1,3-alpha-glucosidase inhibitor
0,082	0,041	mRNA (guanine-N7-)-methyltransferase inhibitor
0,200	0,159	Formate-dihydrofolate ligase inhibitor
0,088	0,048	(R)-aminopropanol dehydrogenase inhibitor
0,189	0,149	Anti-Helicobacter pylori
0,131	0,091	Alkene monooxygenase inhibitor
0,093	0,053	Alpha 2c adrenoreceptor antagonist
0,060	0,020	CC chemokine 2 receptor antagonist
0,075	0,035	Glutathione reductase stimulant
0,075	0,035	Glutathione reductase (NADPH) stimulant
0,226	0,186	Retinoic acid metabolism inhibitor
0,095	0,055	1,5-Anhydro-D-fructose reductase inhibitor
0,123	0,083	Aldehyde dehydrogenase (NAD+) inhibitor
0,094	0,055	Beta-N-acetylgalactosaminidase inhibitor
0,075	0,036	Inositol 1,4,5-triphosphate receptor antagonist
0,134	0,095	Nuclease inhibitor
0,114	0,075	Glucosamine-6-phosphate deaminase inhibitor
0,169	0,130	Renal tissue kallikrein inhibitor

0,125	0,086	Cysteine synthase inhibitor
0,137	0,098	Geranylgeranyl-diphosphate geranylgeranyltransferase inhibitor
0,061	0,023	Na+ K+ transporting ATPase inhibitor
0,096	0,058	Valine dehydrogenase (NADP+) inhibitor
0,154	0,115	Antiprotozoal (Plasmodium)
0,255	0,217	2-Dehydropantoate 2-reductase inhibitor
0,118	0,080	Prepilin peptidase inhibitor
0,118	0,080	Haloacetate dehalogenase inhibitor
0,065	0,027	Glycine N-choloyltransferase inhibitor
0,193	0,156	Vasodilator
0,142	0,104	4-Chlorophenylacetate 3,4-dioxygenase inhibitor
0,112	0,075	Anabolic
0,218	0,181	H+-transporting two-sector ATPase inhibitor
0,060	0,024	Biotin-[methylmalonyl-CoA-carboxytransferase] ligase inhibitor
0,132	0,096	Glutamate synthase (ferredoxin) inhibitor
0,131	0,094	Oligopeptidase B inhibitor
0,135	0,098	Thioredoxin reductase inhibitor
0,121	0,085	Cancer procoagulant inhibitor
0,117	0,081	8-Amino-7-oxononanoate synthase inhibitor
0,110	0,074	(R)-limonene 6-monooxygenase inhibitor
0,049	0,013	CYP4F8 substrate
0,125	0,089	Guanylate cyclase stimulant
0,159	0,124	Lymphocytopoiesis inhibitor
0,129	0,094	Hepatocyte nuclear factor antagonist
0,129	0,094	Hepatocyte nuclear factor 4 alpha antagonist
0,136	0,101	4-Chlorobenzoyl-CoA dehalogenase inhibitor
0,137	0,102	Alkylglycerone-phosphate synthase inhibitor
0,078	0,043	Alpha adrenoreceptor agonist
0,102	0,068	Galactoside 2-alpha-L-fucosyltransferase inhibitor
0,155	0,120	Thermitase inhibitor
0,073	0,039	Deoxyhypusine synthase inhibitor
0,111	0,076	Renal failure treatment
0,122	0,088	Interferon antagonist
0,056	0,022	Anhydrotetracycline monooxygenase inhibitor
0,179	0,145	Cholestanetriol 26-monooxygenase inhibitor
0,128	0,094	Glutamin-(asparagin-)ase inhibitor
0,125	0,091	Candidapepsin inhibitor

0,087	0,053	Galactosylceramidase inhibitor
0,097	0,064	Aspartate transaminase inhibitor
0,077	0,043	L-aminoadipate-semialdehyde dehydrogenase inhibitor
0,129	0,095	Penicillopepsin inhibitor
0,105	0,072	Constipation treatment
0,117	0,084	Cathepsin H inhibitor
0,320	0,287	CDP-glycerol glycerophosphotransferase inhibitor
0,087	0,054	Guanidinopropionase inhibitor
0,107	0,074	NAD(P)+ transhydrogenase (AB-specific) inhibitor
0,076	0,044	Alternansucrase inhibitor
0,115	0,082	L-iduronidase inhibitor
0,173	0,140	O-aminophenol oxidase inhibitor
0,180	0,148	L-threonine 3-dehydrogenase inhibitor
0,073	0,041	UGT2B7H substrate
0,073	0,041	UGT2B7Y substrate
0,093	0,061	Glycerol dehydratase inhibitor
0,079	0,047	Glycerophosphocholine phosphodiesterase inhibitor
0,169	0,137	Antihypercholesterolemic
0,247	0,216	RNA-directed RNA polymerase inhibitor
0,100	0,069	Glutamate N-acetyltransferase inhibitor
0,063	0,031	D-Serine ammonia-lyase inhibitor
0,086	0,054	Cellobiose phosphorylase inhibitor
0,140	0,109	Inorganic diphosphatase inhibitor
0,058	0,026	Potassium channel Kv1.3 blocker
0,183	0,152	Hepatoprotectant
0,074	0,043	Alpha-L-rhamnosidase inhibitor
0,076	0,044	5 Hydroxytryptamine 1 antagonist
0,072	0,041	Sleep apnea treatment
0,158	0,127	Mandelate 4-monooxygenase inhibitor
0,136	0,104	Phosphatidate phosphatase inhibitor
0,192	0,161	AR expression inhibitor
0,152	0,121	Endopeptidase inhibitor
0,118	0,087	Acylglycerone-phosphate reductase inhibitor
0,117	0,086	Allophanate hydrolase inhibitor
0,079	0,049	Beta 1 adrenoreceptor agonist
0,138	0,107	Anthranilate 3-monooxygenase (deaminating) inhibitor
0,090	0,060	CYP4F substrate

0,079	0,049	Aspartate-semialdehyde dehydrogenase inhibitor
0,098	0,068	Aspartate-ammonia ligase (ADP-forming) inhibitor
0,118	0,088	Interferon gamma antagonist
0,036	0,006	Sigma 3 receptor antagonist
0,136	0,106	3-Hydroxy-4-oxoquinoline 2,4-dioxygenase inhibitor
0,200	0,171	Venom exonuclease inhibitor
0,127	0,098	3-Aminobutyryl-CoA ammonia-lyase inhibitor
0,096	0,066	Homocysteine desulfhydrase inhibitor
0,109	0,080	Isovaleryl-CoA dehydrogenase inhibitor
0,036	0,007	Cholesterol ester transfer protein antagonist
0,136	0,107	Tryptophan dimethylallyltransferase inhibitor
0,083	0,054	Sterol 24-C-methyltransferase inhibitor
0,059	0,031	Glucan 1,4-alpha-glucosidase inhibitor
0,172	0,144	Antibacterial
0,087	0,059	Necroptosis inhibitor
0,097	0,070	DNA nucleotidylexotransferase inhibitor
0,080	0,053	Anticholelithogenic
0,060	0,033	CMP-KDO synthase inhibitor
0,135	0,108	SMN2 expression enhancer
0,180	0,154	Lactose synthase inhibitor
0,082	0,055	Ribulose-bisphosphate carboxylase inhibitor
0,077	0,051	Steroid sulfotransferase inhibitor
0,045	0,019	Glucosylceramidase inhibitor
0,179	0,152	Reductant
0,142	0,116	Glycine C-acetyltransferase inhibitor
0,058	0,032	D-Alanine-poly(phosphoribitol) ligase inhibitor
0,148	0,122	Skin irritation, inactive
0,103	0,078	Phosphonoacetate hydrolase inhibitor
0,062	0,037	Hyperparathyroidism treatment
0,170	0,145	Uroporphyrinogen-III synthase inhibitor
0,074	0,049	Inositol-3-phosphate synthase inhibitor
0,105	0,080	Acetolactate decarboxylase inhibitor
0,047	0,022	Glutamate (mGluR2) agonist
0,094	0,069	CMP-N-acetylneuraminate monooxygenase inhibitor
0,074	0,049	CYP2C2 substrate
0,105	0,081	Poly(ADP-ribose) glycohydrolase inhibitor
0,128	0,104	Glucosyl transferase inhibitor

0,052	0,028	Premature ejaculation treatment
0,158	0,134	Glyoxylate oxidase inhibitor
0,053	0,029	Sucrose-phosphate phosphatase inhibitor
0,104	0,080	2-Aminoadipate transaminase inhibitor
0,058	0,035	Sucrose alpha-glucosidase inhibitor
0,136	0,113	Acetylornithine deacetylase inhibitor
0,076	0,053	Isoamylase inhibitor
0,099	0,076	Dopamine D2S antagonist
0,076	0,053	Opioid mu 2 receptor antagonist
0,081	0,059	Acetylcholine muscarinic antagonist
0,042	0,020	Endo-beta-N-acetylglucosaminidase inhibitor
0,112	0,090	Biotin carboxylase inhibitor
0,159	0,137	Pitrilysin inhibitor
0,102	0,080	Cysteine desulfurase inhibitor
0,078	0,056	Paget's disease treatment
0,047	0,026	Glutamate (mGluR group II) agonist
0,099	0,078	MAP kinase kinase 7 inhibitor
0,101	0,080	D-lactate dehydrogenase (cytochrome) inhibitor
0,150	0,129	Urticaria treatment
0,096	0,075	Cyclooxygenase 1 substrate
0,101	0,080	NAD+ kinase inhibitor
0,053	0,033	CYP17 substrate
0,086	0,065	Dichloromuconate cycloisomerase inhibitor
0,088	0,068	Alpha,alpha-trehalase inhibitor
0,103	0,083	Fructose-2,6-bisphosphate 2-phosphatase inhibitor
0,174	0,154	Tankyrase inhibitor
0,144	0,124	CYP4A2 substrate
0,105	0,085	Glycogen (starch) synthase inhibitor
0,101	0,082	Decylcitrate synthase inhibitor
0,081	0,062	Arginyltransferase inhibitor
0,125	0,106	Brachyurin inhibitor
0,153	0,134	Fibromyalgia syndrome treatment
0,091	0,072	Cytosole dipeptidase inhibitor
0,031	0,012	Alpha 2b adrenoreceptor agonist
0,105	0,087	Glycoprotein 3-alpha-L-fucosyltransferase inhibitor
0,087	0,068	2-Dehydropantolactone reductase (A-specific) inhibitor
0,083	0,065	Chelator

0,091	0,073	Ketohexokinase inhibitor
0,052	0,034	Spermidine synthase inhibitor
0,108	0,090	Pyruvate dehydrogenase inhibitor
0,071	0,054	Dihydrolipoamide S-acetyltransferase inhibitor
0,082	0,064	Nitrile hydratase inhibitor
0,109	0,092	3-Isopropylmalate dehydratase inhibitor
0,055	0,038	Osmotic diuretic
0,139	0,122	Camphor 1,2-monooxygenase inhibitor
0,152	0,134	Antineoplastic (endocrine cancer)
0,187	0,170	CYP2A substrate
0,122	0,105	Chitin synthase inhibitor
0,138	0,122	Hydroxymethylglutaryl-CoA lyase inhibitor
0,132	0,116	N-acetylneuraminate 4-O-acetyltransferase inhibitor
0,124	0,107	VCAM1 expression inhibitor
0,175	0,159	Cis-1,2-dihydro-1,2-dihydroxynaphthalene dehydrogenase inhibitor
0,064	0,048	Steroid DELTA-isomerase inhibitor
0,095	0,079	Chondroitin 6-sulfotransferase inhibitor
0,077	0,061	Retinol O-fatty-acyltransferase inhibitor
0,093	0,078	Beta-mannosidase inhibitor
0,094	0,078	Glycoprotein-fucosylgalactoside alpha-N-acetylgalactosaminyltransferase inhibitor
0,105	0,090	O-acetylhomoserine aminocarboxypropyltransferase inhibitor
0,110	0,095	2-Oxoglutarate decarboxylase inhibitor
0,036	0,021	Gentamicin 3'-N-acetyltransferase inhibitor
0,064	0,049	CYP24 inhibitor
0,030	0,015	Alpha glucosidase I inhibitor
0,148	0,133	Dihydroxy-acid dehydratase inhibitor
0,136	0,122	Glutathione peroxidase inhibitor
0,081	0,067	UDP-N-acetylglucosamine 2-epimerase inhibitor
0,065	0,051	Corticotropin releasing factor antagonist
0,141	0,128	Laxative
0,095	0,082	Malyl-CoA lyase inhibitor
0,067	0,054	3(or 17)alpha-hydroxysteroid dehydrogenase inhibitor
0,065	0,052	Xylan 1,4-beta-xylosidase inhibitor
0,146	0,133	Choleretic
0,052	0,039	D-lactate-2-sulfatase inhibitor
0,080	0,067	Phosphorylase kinase inhibitor
0,097	0,085	Antibiotic

0,165	0,152	Carbon-monoxide dehydrogenase inhibitor
0,132	0,120	Polynucleotide 5'-hydroxy-kinase inhibitor
0,039	0,027	Isopentenyl-diphosphate DELTA-isomerase inhibitor
0,055	0,043	Alpha,alpha-trehalose phosphorylase (configuration-retaining) inhibitor
0,073	0,061	Cyanate hydratase inhibitor
0,061	0,049	Beta-1,4-mannosyl-glycoprotein 4-beta-N-acetylglucosaminyltransferase inhibitor
0,066	0,054	Alpha 3 adrenoreceptor agonist
0,150	0,138	Bothrolysin inhibitor
0,184	0,172	Myosin ATPase inhibitor
0,300	0,288	Antieczematic
0,053	0,042	Assemblin inhibitor
0,064	0,052	Lactaldehyde dehydrogenase inhibitor
0,040	0,028	Antimitotic, Podophyllotoxin-like
0,073	0,061	Mycodextranase inhibitor
0,058	0,047	Opioid mu 1 receptor antagonist
0,074	0,063	3(or 17)beta-hydroxysteroid dehydrogenase inhibitor
0,138	0,127	Guanidinoacetate kinase inhibitor
0,016	0,004	Alcohol dehydrogenase gamma inhibitor
0,016	0,004	Alcohol dehydrogenase gamma 2 inhibitor
0,081	0,070	CYP26 substrate
0,128	0,117	Heat shock protein agonist
0,081	0,070	D-proline reductase (dithiol) inhibitor
0,134	0,123	Choline kinase inhibitor
0,185	0,174	Plastoquinol-plastocyanin reductase inhibitor
0,041	0,030	Strombine dehydrogenase inhibitor
0,109	0,099	CYP4B1 substrate
0,062	0,051	Alpha,alpha-phosphotrehalase inhibitor
0,081	0,070	Alpha 2b adrenoreceptor antagonist
0,075	0,064	N-acetylneuraminate lyase inhibitor
0,133	0,122	Antineoplastic (melanoma)
0,081	0,071	Trimerelysin II inhibitor
0,096	0,085	Lactaldehyde reductase (NADPH) inhibitor
0,177	0,167	Gamma-glutamyltransferase inhibitor
0,164	0,154	Antineoplastic alkaloid
0,119	0,109	Narcolepsy treatment
0,090	0,080	Monocarboxylic acid transporter inhibitor
0,090	0,080	Monocarboxylic acid transporter 1 inhibitor

0,058	0,048	Malate synthase inhibitor
0,077	0,067	2-Methyleneglutarate mutase inhibitor
0,183	0,173	Antihypertensive
0,078	0,068	5-Oxoprolinase (ATP-hydrolysing) inhibitor
0,074	0,064	Cystathionine beta-lyase inhibitor
0,023	0,014	Alpha galactosidase inhibitor
0,091	0,082	Cholestenone 5alpha-reductase inhibitor
0,125	0,116	Lipoxygenase substrate
0,077	0,068	Threonine ammonia-lyase inhibitor
0,139	0,130	CYP4F2 substrate
0,052	0,043	Hydroxylysine kinase inhibitor
0,056	0,047	Alpha 2 adrenoreceptor agonist
0,010	0,002	Alcohol dehydrogenase alpha inhibitor
0,047	0,039	Delayed rectifier potassium channel blocker
0,079	0,071	2-Dehydro-3-deoxy-L-arabinonate dehydratase inhibitor
0,053	0,045	UGT2B4D substrate
0,053	0,045	UGT2B19 substrate
0,053	0,045	UGT2B4E substrate
0,053	0,045	UGT2B30 substrate
0,053	0,045	UGT2B23 substrate
0,068	0,060	1-Pyrroline-5-carboxylate dehydrogenase inhibitor
0,037	0,029	Tartrate decarboxylase inhibitor
0,027	0,020	I(Ks) voltage-gated potassium channel blocker
0,074	0,066	Nav1.7 sodium channel blocker
0,035	0,028	RNA agonist
0,091	0,084	DNA-3-methyladenine glycosylase II inhibitor
0,128	0,121	Lactate 2-monooxygenase inhibitor
0,042	0,035	D(-)-tartrate dehydratase inhibitor
0,017	0,010	Antiprotozoal activity enhancer
0,053	0,046	1-Alkyl-2-acetylglycerol O-acyltransferase inhibitor
0,091	0,085	Sulfate adenylyltransferase inhibitor
0,134	0,129	Penicillin amidase inhibitor
0,090	0,084	Glycine receptor antagonist
0,151	0,145	Carnitinamidase inhibitor
0,025	0,020	Histone-lysine N-methyltransferase inhibitor
0,122	0,117	3-Hydroxybenzoate 4-monooxygenase inhibitor
0,124	0,118	Melanin inhibitor

0,222	0,216	Cancer associated disorders treatment
0,033	0,028	Diphosphate-serine phosphotransferase inhibitor
0,060	0,055	3-Dehydroquinate synthase inhibitor
0,163	0,158	Hydroxylamine reductase inhibitor
0,069	0,064	Malate dehydrogenase (NADP+) inhibitor
0,087	0,082	tRNA (cytosine-5-)-methyltransferase inhibitor
0,049	0,044	Androgen agonist
0,069	0,065	2-Oxoaldehyde dehydrogenase (NAD+) inhibitor
0,197	0,193	FMO3 substrate
0,108	0,104	Iron-cytochrome-c reductase inhibitor
0,067	0,063	Aminolevulinate transaminase inhibitor
0,114	0,109	Laminaribiose phosphorylase inhibitor
0,134	0,130	Atrolysin A inhibitor
0,007	0,003	Alcohol dehydrogenase I inhibitor
0,048	0,044	Testosterone agonist
0,039	0,035	Androst-4-ene-3,17-dione monooxygenase inhibitor
0,168	0,164	Acidifying agent non gastric
0,087	0,083	Cholinergic antagonist
0,115	0,111	Granzyme A inhibitor
0,191	0,188	Urethanase inhibitor
0,067	0,063	D-alanine transaminase inhibitor
0,021	0,018	Dopamine D2B antagonist
0,108	0,105	VEGF expression inhibitor
0,068	0,065	Serine-glyoxylate transaminase inhibitor
0,086	0,084	Adenosylmethionine-8-amino-7-oxononanoate transaminase inhibitor
0,012	0,009	Smo receptor agonist
0,129	0,126	3-Demethylubiquinone-9 3-O-methyltransferase inhibitor
0,052	0,049	Prostaglandin-I synthase inhibitor
0,128	0,126	Glucuronate isomerase inhibitor
0,210	0,208	NAD(P)+-arginine ADP-ribosyltransferase inhibitor
0,054	0,052	Asparagine-oxo-acid transaminase inhibitor
0,074	0,071	Scyllo-inosamine-4-phosphate amidinotransferase inhibitor
0,063	0,061	Propanediol dehydratase inhibitor
0,196	0,194	ICAM1 expression inhibitor
0,056	0,054	Thioether S-methyltransferase inhibitor
0,060	0,058	Acetylcholine M3 receptor antagonist
0,071	0,069	Cysteine transaminase inhibitor

0,104	0,102	Membrane dipeptidase inhibitor
0,019	0,018	NMDA receptor phencyclidine site antagonist
0,127	0,126	Antileprosy
0,195	0,194	Antiprotozoal (Coccidial)
0,055	0,054	Acyl-CoA dehydrogenase inhibitor
0,112	0,111	Guanidinoacetate N-methyltransferase inhibitor
0,082	0,081	Acetylcholine antagonist
0,088	0,088	Glucose 1-dehydrogenase inhibitor
0,096	0,096	CYP3C substrate
0,012	0,011	CYP19 inducer
0,052	0,051	ATP diphosphatase inhibitor
0,089	0,088	Cell wall synthesis inhibitor
0,025	0,025	Phosphopyruvate hydratase inhibitor
0,074	0,073	Anthranilate synthase inhibitor
0,104	0,104	Clostripain inhibitor
0,043	0,043	CYP3A11 substrate
0,109	0,109	Catalase inhibitor
0,094	0,094	Coagulant

Complex 2 All

All Pa>Pi Pa>0,3 Pa>0,7

Pa	Pi	Activity
0,787	0,003	Vascular endothelial growth factor 3 antagonist
0,793	0,036	Phobic disorders treatment
0,778	0,031	Testosterone 17beta-dehydrogenase (NADP+) inhibitor
0,762	0,018	Nicotinic alpha6beta3beta4alpha5 receptor antagonist
0,758	0,017	Antineoplastic
0,760	0,029	Acrocylindropepsin inhibitor
0,760	0,029	Saccharopepsin inhibitor
0,760	0,029	Chymosin inhibitor
0,720	0,008	Glucan 1,4-alpha-maltotriohydrolase inhibitor
0,706	0,003	Tyrosine-protein kinase receptor FLT3 inhibitor
0,721	0,019	5-O-(4-coumaroyl)-D-quinate 3'-monooxygenase inhibitor

0,746	0,049	Aspulvinone dimethylallyltransferase inhibitor
0,711	0,024	Nicotinic alpha2beta2 receptor antagonist
0,680	0,001	Steroid DELTA-isomerase inhibitor
0,696	0,034	Sugar-phosphatase inhibitor
0,683	0,022	Alkylacetylglycerophosphatase inhibitor
0,689	0,028	Acylcarnitine hydrolase inhibitor
0,662	0,006	Antineoplastic (non-Hodgkin's lymphoma)
0,646	0,030	Ribulose-phosphate 3-epimerase inhibitor
0,663	0,064	CYP2J substrate
0,597	0,006	Imidazoline receptor agonist
0,617	0,031	Glucan endo-1,6-beta-glucosidase inhibitor
0,639	0,056	Polyporopepsin inhibitor
0,592	0,011	Dolichyl-diphosphooligosaccharide-protein glycotransferase inhibitor
0,583	0,005	Platelet aggregation inhibitor
0,653	0,086	Ubiquinol-cytochrome-c reductase inhibitor
0,613	0,050	Glutamyl endopeptidase II inhibitor
0,589	0,033	Feruloyl esterase inhibitor
0,583	0,029	27-Hydroxycholesterol 7alpha-monooxygenase inhibitor
0,595	0,046	Alkenylglycerophosphocholine hydrolase inhibitor
0,604	0,061	CYP2J2 substrate
0,560	0,019	Mannan endo-1,4-beta-mannosidase inhibitor
0,556	0,022	All-trans-retinyl-palmitate hydrolase inhibitor
0,568	0,035	Glucan endo-1,3-beta-D-glucosidase inhibitor
0,550	0,020	CYP2A4 substrate
0,575	0,050	NADPH peroxidase inhibitor
0,570	0,046	Complement factor D inhibitor
0,526	0,005	Antineoplastic (lymphocytic leukemia)
0,572	0,054	Kidney function stimulant
0,546	0,046	Platelet aggregation stimulant
0,509	0,013	Dementia treatment
0,537	0,041	Chloride peroxidase inhibitor
0,486	0,002	4-Carboxymethyl-4-methylbutenolide mutase inhibitor
0,530	0,048	UDP-N-acetylglucosamine 4-epimerase inhibitor
0,522	0,043	Pterin deaminase inhibitor
0,531	0,052	Macrophage colony stimulating factor agonist
0,502	0,027	Glyoxylate reductase inhibitor
0,493	0,019	Albendazole monooxygenase inhibitor

0,498	0,031	Pullulanase inhibitor
0,510	0,044	Fatty-acyl-CoA synthase inhibitor
0,498	0,033	CYP2B5 substrate
0,498	0,038	L-glutamate oxidase inhibitor
0,510	0,052	Limulus clotting factor B inhibitor
0,464	0,011	Imidazoline I1 receptor agonist
0,533	0,086	Phosphatase inhibitor
0,505	0,058	Carboxypeptidase Taq inhibitor
0,475	0,028	Peptidoglycan glycosyltransferase inhibitor
0,533	0,086	Glycosylphosphatidylinositol phospholipase D inhibitor
0,508	0,061	Electron-transferring-flavoprotein dehydrogenase inhibitor
0,488	0,046	Exoribonuclease II inhibitor
0,445	0,010	Endo-1,3(4)-beta-glucanase inhibitor
0,485	0,051	1,4-Lactonase inhibitor
0,508	0,075	Omptin inhibitor
0,433	0,005	Platelet activating factor antagonist
0,496	0,069	Platelet adhesion inhibitor
0,481	0,061	Venombin AB inhibitor
0,423	0,004	Platelet activating factor alpha antagonist
0,450	0,032	Chemosensitizer
0,469	0,051	Hydrogen dehydrogenase inhibitor
0,477	0,060	JAK2 expression inhibitor
0,462	0,046	Gluconate 5-dehydrogenase inhibitor
0,466	0,050	Trimethylamine-oxide aldolase inhibitor
0,442	0,031	Cardiovascular analeptic
0,491	0,080	Acetylcholine neuromuscular blocking agent
0,475	0,064	GST A substrate
0,459	0,051	Cyclic AMP agonist
0,448	0,041	Acetylesterase inhibitor
0,450	0,048	3-Hydroxybenzoate 6-monooxygenase inhibitor
0,438	0,037	Simian immunodeficiency virus proteinase inhibitor
0,463	0,063	Phosphatidylcholine-retinol O-acyltransferase inhibitor
0,471	0,073	Lysase inhibitor
0,490	0,096	Fusarinine-C ornithinesterase inhibitor
0,418	0,025	Xylan endo-1,3-beta-xylosidase inhibitor
0,421	0,028	Cutinase inhibitor
0,392	0,001	Elastase 1 inhibitor

0,394	0,005	Proto-oncogene tyrosine-protein kinase Kit inhibitor
0,412	0,024	Taurine-2-oxoglutarate transaminase inhibitor
0,392	0,005	GST T substrate
0,392	0,005	GST T1-1 substrate
0,435	0,048	Alkenylglycerophosphoethanolamine hydrolase inhibitor
0,409	0,022	Sorbitol-6-phosphate 2-dehydrogenase inhibitor
0,395	0,009	Vascular dementia treatment
0,485	0,100	Pseudolysin inhibitor
0,458	0,074	Thioredoxin inhibitor
0,417	0,036	Arylmalonate decarboxylase inhibitor
0,420	0,039	Aspergillopepsin I inhibitor
0,453	0,072	Alopecia treatment
0,410	0,032	Glucan 1,4-alpha-maltotetraohydrolase inhibitor
0,423	0,048	Acetylgalactosaminyl-O-glycosyl-glycoprotein beta-1,3-N-acetylglucosaminyltransferase inhibitor
0,404	0,032	Poly(beta-D-mannuronate) lyase inhibitor
0,420	0,049	Na+-transporting two-sector ATPase inhibitor
0,400	0,030	Glycolate dehydrogenase inhibitor
0,393	0,023	CDP-diacylglycerol-glycerol-3-phosphate 3-phosphatidyltransferase inhibitor
0,396	0,027	Shikimate O-hydroxycinnamoyltransferase inhibitor
0,389	0,021	Pediculicide
0,423	0,055	Formaldehyde transketolase inhibitor
0,430	0,064	Fragilysin inhibitor
0,416	0,051	N-acetylneuraminate 7-O(or 9-O)-acetyltransferase inhibitor
0,445	0,081	Antidyskinetic
0,457	0,094	Phthalate 4,5-dioxygenase inhibitor
0,434	0,072	Lysine 2,3-aminomutase inhibitor
0,393	0,033	Antineoplastic (breast cancer)
0,381	0,024	Myeloblastin inhibitor
0,425	0,068	Dehydro-L-gulonate decarboxylase inhibitor
0,445	0,091	Ovulation inhibitor
0,383	0,030	Clavaminate synthase inhibitor
0,360	0,007	Alpha-pinene-oxide decyclase inhibitor
0,437	0,090	Phospholipid-translocating ATPase inhibitor
0,408	0,065	CYP2D15 substrate
0,358	0,015	Maltose-transporting ATPase inhibitor
0,410	0,068	CYP2D16 substrate
0,440	0,099	Protein-disulfide reductase (glutathione) inhibitor

0,379	0,039	2-Haloacid dehalogenase inhibitor
0,377	0,038	Undecaprenyldiphospho-muramoylpentapeptide beta-N-acetylglucosaminyltransferase inhibitor
0,388	0,050	Malate oxidase inhibitor
0,437	0,101	CYP2C12 substrate
0,383	0,048	IgA-specific serine endopeptidase inhibitor
0,390	0,056	Cyclohexanone monooxygenase inhibitor
0,404	0,070	Apyrase inhibitor
0,430	0,096	NADPH-cytochrome-c2 reductase inhibitor
0,364	0,031	Yeast ribonuclease inhibitor
0,357	0,024	Sclerosant
0,383	0,051	Limulus clotting factor C inhibitor
0,368	0,036	H+-exporting ATPase inhibitor
0,374	0,042	Mannose isomerase inhibitor
0,372	0,041	CYP7 inhibitor
0,367	0,037	Fructan beta-fructosidase inhibitor
0,414	0,084	Leukopoiesis stimulant
0,363	0,035	CYP2B11 substrate
0,367	0,038	Cyclomaltodextrinase inhibitor
0,410	0,082	Erythropoiesis stimulant
0,369	0,043	Sulfite oxidase inhibitor
0,350	0,024	Styrene-oxide isomerase inhibitor
0,382	0,058	Mucinaminylserine mucinaminidase inhibitor
0,348	0,025	Phosphoenolpyruvate mutase inhibitor
0,340	0,018	Undecaprenyl-diphosphatase inhibitor
0,393	0,070	Polyamine-transporting ATPase inhibitor
0,353	0,031	Oxidizing agent
0,370	0,048	Nitrite reductase (NO-forming) inhibitor
0,380	0,059	EIF4E expression inhibitor
0,371	0,054	CYP2D2 inhibitor
0,380	0,063	Thymidylate 5'-phosphatase inhibitor
0,405	0,089	Neurotransmitter antagonist
0,345	0,030	D-alanine 2-hydroxymethyltransferase inhibitor
0,374	0,060	GABA aminotransferase inhibitor
0,368	0,054	Steroid N-acetylglucosaminyltransferase inhibitor
0,392	0,079	Arginine 2-monooxygenase inhibitor
0,381	0,068	Phenol O-methyltransferase inhibitor
0,398	0,089	(R)-6-hydroxynicotine oxidase inhibitor

0,320	0,012	Ligase inhibitor
0,361	0,053	Phosphopantothenoylcysteine decarboxylase inhibitor
0,472	0,165	Membrane permeability inhibitor
0,387	0,080	Ecdysone 20-monooxygenase inhibitor
0,389	0,083	CYP2A1 substrate
0,372	0,068	(S)-6-hydroxynicotine oxidase inhibitor
0,373	0,069	Caspase 8 stimulant
0,356	0,052	Chitosanase inhibitor
0,374	0,071	Adenomatous polyposis treatment
0,430	0,127	Membrane integrity agonist
0,344	0,042	Glyoxylate oxidase inhibitor
0,338	0,037	Carminative
0,357	0,056	N-formylmethionyl-peptidase inhibitor
0,385	0,085	Vasoprotector
0,405	0,106	Antiviral (Picornavirus)
0,375	0,077	Cl--transporting ATPase inhibitor
0,383	0,086	4-Nitrophenol 2-monooxygenase inhibitor
0,325	0,028	Licheninase inhibitor
0,344	0,048	Dextranase inhibitor
0,382	0,086	Fibrolase inhibitor
0,401	0,108	Antinociceptive
0,352	0,059	Peptide-N4-(N-acetyl-beta-glucosaminyl)asparagine amidase inhibitor
0,371	0,078	Aspartate-phenylpyruvate transaminase inhibitor
0,379	0,086	Dimethylargininase inhibitor
0,320	0,028	Antiparkinsonian, tremor relieving
0,389	0,097	Aminobutyraldehyde dehydrogenase inhibitor
0,318	0,026	Fumarate reductase (NADH) inhibitor
0,329	0,038	CYP2B10 substrate
0,329	0,040	2,3,4,5-Tetrahydropyridine-2,6-dicarboxylate N-succinyltransferase inhibitor
0,378	0,090	Radiosensitizer
0,333	0,045	DELTA14-sterol reductase inhibitor
0,365	0,078	Manganese peroxidase inhibitor
0,337	0,052	Opioid kappa 3 receptor antagonist
0,365	0,081	Pro-opiomelanocortin converting enzyme inhibitor
0,347	0,064	Phosphoinositide 5-phosphatase inhibitor
0,331	0,048	CYP2A2 substrate
0,324	0,042	Quinoprotein glucose dehydrogenase inhibitor

0,365	0,085	Beta-adrenergic receptor kinase inhibitor
0,365	0,085	G-protein-coupled receptor kinase inhibitor
0,340	0,062	Creatininase inhibitor
0,346	0,067	Prostaglandin-A1 DELTA-isomerase inhibitor
0,357	0,079	Centromere associated protein inhibitor
0,309	0,031	Galactolipase inhibitor
0,308	0,031	Alkylglycerone-phosphate synthase inhibitor
0,384	0,107	Cytoprotectant
0,306	0,030	Bisphosphoglycerate mutase inhibitor
0,337	0,062	Lysostaphin inhibitor
0,409	0,134	CYP3A2 substrate
0,351	0,076	Histidine N-acetyltransferase inhibitor
0,423	0,148	Calcium channel (voltage-sensitive) activator
0,320	0,046	Antineoplastic (pancreatic cancer)
0,353	0,080	Glycerol-3-phosphate oxidase inhibitor
0,336	0,062	tRNA-pseudouridine synthase I inhibitor
0,356	0,083	Antimyopathies
0,350	0,079	CYP2A8 substrate
0,309	0,042	GST M substrate
0,303	0,037	Alpha-N-acetylglucosaminidase inhibitor
0,320	0,054	Hematopoietic inhibitor
0,307	0,042	Steroid synthesis inhibitor
0,349	0,084	Membrane permeability enhancer
0,350	0,086	Sphinganine kinase inhibitor
0,308	0,044	Hyaluronic acid agonist
0,332	0,069	RELA expression inhibitor
0,295	0,033	Antineoplastic (small cell lung cancer)
0,302	0,040	Oryzin inhibitor
0,296	0,035	Alkylglycerophosphoethanolamine phosphodiesterase inhibitor
0,355	0,094	Pancreatic elastase inhibitor
0,282	0,022	2-Dehydropantoate aldolase inhibitor
0,327	0,068	Alcohol dehydrogenase (acceptor) inhibitor
0,334	0,076	Leukopoiesis inhibitor
0,293	0,036	Polyneuridine-aldehyde esterase inhibitor
0,338	0,081	Tpr proteinase (Porphyromonas gingivalis) inhibitor
0,283	0,026	Hydroxysteroid dehydrogenase inhibitor
0,326	0,071	Chitinase inhibitor

0,321	0,067	3-Cyanoalanine hydratase inhibitor
0,308	0,057	Hydroxylamine reductase (NADH) inhibitor
0,323	0,072	Antifungal
0,321	0,070	Glutamine-phenylpyruvate transaminase inhibitor
0,346	0,096	Linoleate diol synthase inhibitor
0,291	0,041	N-Acyl-D-aspartate deacylase inhibitor
0,299	0,050	Linoleoyl-CoA desaturase inhibitor
0,330	0,080	Methylamine-glutamate N-methyltransferase inhibitor
0,291	0,043	Protein-Npi-phosphohistidine-sugar phosphotransferase inhibitor
0,360	0,112	Histamine release stimulant
0,382	0,134	5 Hydroxytryptamine uptake stimulant
0,358	0,111	2-Dehydropantoate 2-reductase inhibitor
0,326	0,079	CYP2F1 substrate
0,286	0,040	Poly(alpha-L-guluronate) lyase inhibitor
0,265	0,021	Galacturan 1,4-alpha-galacturonidase inhibitor
0,319	0,075	ADP-thymidine kinase inhibitor
0,335	0,092	Glucose oxidase inhibitor
0,286	0,042	Pseudouridylate synthase inhibitor
0,261	0,019	Deoxyribonuclease I inhibitor
0,319	0,077	Chenodeoxycholoyltaurine hydrolase inhibitor
0,299	0,058	Glycerol-3-phosphate dehydrogenase inhibitor
0,302	0,061	2-Hydroxymuconate-semialdehyde hydrolase inhibitor
0,268	0,028	Alpha-glucuronidase inhibitor
0,265	0,025	Gamma-butyrobetaine dioxygenase inhibitor
0,342	0,102	Rubredoxin-NAD+ reductase inhibitor
0,342	0,103	Apoptosis agonist
0,319	0,082	Fucosterol-epoxide lyase inhibitor
0,310	0,073	3-Phytase inhibitor
0,275	0,038	RNA directed DNA polymerase inhibitor
0,282	0,046	Horrilysin inhibitor
0,263	0,028	Tropinesterase inhibitor
0,309	0,076	Adenylyl-sulfate reductase inhibitor
0,305	0,072	Phosphatidylserine decarboxylase inhibitor
0,276	0,044	Dopamine release stimulant
0,277	0,045	Aspergillopepsin II inhibitor
0,304	0,071	N-acylmannosamine kinase inhibitor
0,260	0,029	Cycloartenol synthase inhibitor

0,273	0,042	Rhodotorulapepsin inhibitor
0,334	0,103	Biotinidase inhibitor
0,289	0,059	NF-E2-related factor 2 stimulant
0,278	0,048	Chaperonin ATPase inhibitor
0,257	0,028	Phosphoenolpyruvate-protein phosphotransferase inhibitor
0,297	0,069	Cytostatic
0,258	0,030	Rhizopuspepsin inhibitor
0,282	0,054	Adrenaline release stimulant
0,291	0,064	Long-chain-aldehyde dehydrogenase inhibitor
0,328	0,101	S-formylglutathione hydrolase inhibitor
0,282	0,055	Levanase inhibitor
0,241	0,015	Isoamylase inhibitor
0,299	0,075	D-lactaldehyde dehydrogenase inhibitor
0,316	0,092	Sulfite dehydrogenase inhibitor
0,392	0,168	Antineurotic
0,272	0,049	2-Oxoaldehyde dehydrogenase (NADP+) inhibitor
0,284	0,061	Endothelial growth factor antagonist
0,271	0,048	UGT2B28 substrate
0,267	0,044	Aldosterone antagonist
0,298	0,075	Isopenicillin-N epimerase inhibitor
0,265	0,043	Anthranilate-CoA ligase inhibitor
0,295	0,073	Peptide alpha-N-acetyltransferase inhibitor
0,361	0,140	Thromboxane B2 antagonist
0,280	0,059	Bisphosphoglycerate phosphatase inhibitor
0,280	0,060	Dipeptidase E inhibitor
0,289	0,069	Glutamate-tRNA ligase inhibitor
0,235	0,015	Trehalose-phosphatase inhibitor
0,261	0,042	Vitamin-K-epoxide reductase (warfarin-insensitive) inhibitor
0,282	0,063	CYP2C29 substrate
0,256	0,037	D-xylulose reductase inhibitor
0,348	0,130	Chlordecone reductase inhibitor
0,268	0,050	2-Hydroxy-3-oxoadipate synthase inhibitor
0,250	0,033	1,4-Alpha-glucan branching enzyme inhibitor
0,262	0,045	Mannitol-1-phosphatase inhibitor
0,325	0,108	CYP4A11 substrate
0,307	0,090	Plastoquinol-plastocyanin reductase inhibitor
0,278	0,062	Salicylate 1-monooxygenase inhibitor

0,245	0,029	BRAF expression inhibitor
0,317	0,101	Hydroxylamine oxidase inhibitor
0,298	0,083	Phosphatidylinositol diacylglycerol-lyase inhibitor
0,270	0,056	Benzaldehyde dehydrogenase (NADP+) inhibitor
0,305	0,092	Peptidyl-dipeptidase Dcp inhibitor
0,266	0,053	Flavin-containing monooxygenase inhibitor
0,278	0,066	Transketolase inhibitor
0,230	0,017	Alpha-N-acetylgalactosaminidase inhibitor
0,332	0,120	CYP3A1 substrate
0,275	0,062	Gluconolactonase inhibitor
0,258	0,046	N-acetyllactosaminide beta-1,3-N-acetylglucosaminyltransferase inhibitor
0,278	0,066	NADH kinase inhibitor
0,245	0,034	3-Chloro-D-alanine dehydrochlorinase inhibitor
0,248	0,037	NAD+ synthase (glutamine-hydrolysing) inhibitor
0,262	0,052	Glutarate-semialdehyde dehydrogenase inhibitor
0,338	0,130	Pin1 inhibitor
0,305	0,098	(R)-Pantolactone dehydrogenase (flavin) inhibitor
0,251	0,044	Dynein ATPase inhibitor
0,285	0,081	CYP2A5 substrate
0,251	0,046	Cellulose 1,4-beta-cellobiosidase inhibitor
0,320	0,116	Antiseborrheic
0,245	0,041	Aureolysin inhibitor
0,303	0,099	Cyanoalanine nitrilase inhibitor
0,261	0,057	Lactose synthase inhibitor
0,305	0,102	Leukotriene-C4 synthase inhibitor
0,270	0,068	Cystic fibrosis treatment
0,233	0,030	Alkene monooxygenase inhibitor
0,252	0,051	Prunasin beta-glucosidase inhibitor
0,257	0,057	N-hydroxy-2-acetamidofluorene reductase inhibitor
0,222	0,022	Riboflavin phosphotransferase inhibitor
0,227	0,028	Ferredoxin-NADP+ reductase inhibitor
0,311	0,112	Malate dehydrogenase (acceptor) inhibitor
0,241	0,042	Aryldialkylphosphatase inhibitor
0,264	0,066	Ethanolamine-phosphate cytidylyltransferase inhibitor
0,237	0,039	Inulinase inhibitor
0,284	0,086	P-benzoquinone reductase (NADPH) inhibitor
0,338	0,140	Oxygen scavenger

0,251	0,053	Alpha-1,6-mannosyl-glycoprotein 4-beta-N-acetylglucosaminyltransferase inhibitor
0,339	0,142	MAP kinase stimulant
0,244	0,049	Polygalacturonase inhibitor
0,251	0,056	Pappalysin-1 inhibitor
0,298	0,103	MMP9 expression inhibitor
0,225	0,030	Glucan 1,6-alpha-glucosidase inhibitor
0,272	0,077	Opioid dependency treatment
0,310	0,116	CYP2C9 inducer
0,245	0,051	3-Methylbutanal reductase inhibitor
0,291	0,097	Antiviral (Adenovirus)
0,235	0,042	Glucan 1,4-beta-glucosidase inhibitor
0,227	0,034	Glycerol-1-phosphatase inhibitor
0,235	0,042	Lactaldehyde reductase inhibitor
0,221	0,028	2,2-Dialkylglycine decarboxylase (pyruvate) inhibitor
0,262	0,070	S-alkylcysteine lyase inhibitor
0,273	0,081	Aspergillus nuclease S1 inhibitor
0,268	0,077	MAP kinase kinase 4 inhibitor
0,236	0,045	Snapalysin inhibitor
0,244	0,053	Glycine dehydrogenase (decarboxylating) inhibitor
0,239	0,049	Cyclooxygenase substrate
0,213	0,024	Estradiol 17alpha-dehydrogenase inhibitor
0,227	0,039	2-Haloacid dehalogenase (configuration-inverting) inhibitor
0,253	0,065	CYP2G1 substrate
0,225	0,037	Aspartate-tRNA ligase inhibitor
0,243	0,056	GABA C receptor agonist
0,276	0,089	DNA-(apurinic or apyrimidinic site) lyase inhibitor
0,250	0,064	Antiperistaltic
0,273	0,088	Antituberculosic
0,218	0,033	Allantoate deiminase inhibitor
0,222	0,037	Bile-salt sulfotransferase inhibitor
0,214	0,030	CYP2B18 substrate
0,251	0,068	Cyclohexyl-isocyanide hydratase inhibitor
0,300	0,117	Endopeptidase So inhibitor
0,276	0,093	Lysyl oxidase inhibitor
0,209	0,027	Carnitine dehydratase inhibitor
0,239	0,057	Crotonoyl-[acyl-carrier-protein] hydratase inhibitor
0,242	0,059	Ornithine cyclodeaminase inhibitor

0,268	0,087	4-Coumarate-CoA ligase inhibitor
0,221	0,041	GABA B receptor agonist
0,287	0,107	Myc inhibitor
0,195	0,015	CYP3A5 inducer
0,244	0,064	N-acetyllactosamine synthase inhibitor
0,212	0,032	L-iduronidase inhibitor
0,238	0,059	N-(long-chain-acyl)ethanolamine deacylase inhibitor
0,216	0,038	Cyclamate sulfohydrolase inhibitor
0,251	0,072	Glutathione dehydrogenase (ascorbate) inhibitor
0,247	0,069	Alanine-tRNA ligase inhibitor
0,269	0,091	Methylumbelliferyl-acetate deacetylase inhibitor
0,209	0,033	Glycopeptide alpha-N-acetylgalactosaminidase inhibitor
0,209	0,033	Glycerone-phosphate O-acyltransferase inhibitor
0,227	0,050	Sedoheptulose-bisphosphatase inhibitor
0,251	0,075	Aspartate-ammonia ligase inhibitor
0,210	0,034	Pectate lyase inhibitor
0,264	0,088	Pyruvate decarboxylase inhibitor
0,276	0,100	CYP3A7 substrate
0,298	0,123	Cytochrome P450 stimulant
0,235	0,060	Procollagen N-endopeptidase inhibitor
0,251	0,077	Transcription factor NF kappa A inhibitor
0,234	0,059	Acyl-CoA oxidase inhibitor
0,265	0,091	N-benzyloxycarbonylglycine hydrolase inhibitor
0,260	0,086	Antiviral (Poxvirus)
0,208	0,035	Antineoplastic (ovarian cancer)
0,209	0,036	Retinyl-palmitate esterase inhibitor
0,210	0,037	Acetylenecarboxylate hydratase inhibitor
0,336	0,163	Octopamine antagonist
0,177	0,005	Cathepsin B inhibitor
0,290	0,119	Antihelmintic (Nematodes)
0,270	0,099	Naphthalene 1,2-dioxygenase inhibitor
0,270	0,099	Ferredoxin-NAD+ reductase inhibitor
0,223	0,052	2,3-Dihydroxyindole 2,3-dioxygenase inhibitor
0,252	0,081	Carbon-monoxide dehydrogenase inhibitor
0,249	0,079	Antiviral (CMV)
0,267	0,097	Para amino benzoic acid antagonist
0,248	0,078	Cholestanetriol 26-monooxygenase inhibitor

0,270	0,101	Antimycobacterial
0,242	0,074	Morphine 6-dehydrogenase inhibitor
0,271	0,104	Enteropeptidase inhibitor
0,183	0,017	Demethylsterigmatocystin 6-O-methyltransferase inhibitor
0,216	0,050	Sterol 3-beta-glucosyltransferase inhibitor
0,284	0,118	NAD(P)+-arginine ADP-ribosyltransferase inhibitor
0,234	0,068	Antipruritic, non-allergic
0,197	0,031	Protein-glucosylgalactosylhydroxylysine glucosidase inhibitor
0,193	0,028	Pectin lyase inhibitor
0,297	0,132	Gonadotropin antagonist
0,223	0,058	D-threo-aldose 1-dehydrogenase inhibitor
0,311	0,147	Antihypoxic
0,327	0,163	Fructose 5-dehydrogenase inhibitor
0,207	0,044	Nitric oxide scavenger
0,195	0,032	Glucuronolactone reductase inhibitor
0,234	0,071	Carboxypeptidase D inhibitor
0,232	0,070	Di-trans,poly-cis-decaprenylcistransferase inhibitor
0,204	0,042	Glutamin-(asparagin-)ase inhibitor
0,187	0,025	(R)-limonene 6-monooxygenase inhibitor
0,241	0,079	Cell wall biosynthesis inhibitor
0,257	0,096	Thiosulfate dehydrogenase inhibitor
0,282	0,122	CYP3A4 inducer
0,196	0,037	Thioredoxin reductase inhibitor
0,256	0,097	Immunostimulant
0,238	0,080	Opheline kinase inhibitor
0,238	0,080	Taurocyamine kinase inhibitor
0,231	0,073	Dimethylmaleate hydratase inhibitor
0,244	0,086	Succinate-semialdehyde dehydrogenase [NAD(P)+] inhibitor
0,201	0,043	Vascular adhesion protein 1 inhibitor
0,282	0,125	Lipoprotein lipase inhibitor
0,218	0,061	Tauropine dehydrogenase inhibitor
0,210	0,054	Leucine dehydrogenase inhibitor
0,285	0,130	Glutathione thiolesterase inhibitor
0,240	0,085	NOS2 expression inhibitor
0,188	0,033	Gaucher disease treatment
0,278	0,124	5 Hydroxytryptamine release stimulant
0,234	0,080	[acyl-carrier-protein] S-acetyltransferase inhibitor

0,272	0,118	CYP3A inducer
0,197	0,043	Dolichyl-phosphatase inhibitor
0,204	0,051	Farnesyltranstransferase inhibitor
0,181	0,028	Dihydrouracil dehydrogenase (NAD+) inhibitor
0,194	0,042	Ganglioside galactosyltransferase inhibitor
0,233	0,080	Prolyl aminopeptidase inhibitor
0,185	0,032	Endo-1,4-beta-xylanase inhibitor
0,261	0,110	Antiprotozoal (Amoeba)
0,222	0,072	Glycerol 2-dehydrogenase (NADP+) inhibitor
0,226	0,076	Gamma-D-Glutamyl-meso-diaminopimelate peptidase inhibitor
0,293	0,143	Caspase 3 stimulant
0,217	0,068	UGT2B17 substrate
0,238	0,089	Thiamine-triphosphatase inhibitor
0,237	0,088	Arylesterase inhibitor
0,205	0,056	Serratia marcescens nuclease inhibitor
0,289	0,140	Nitrate reductase (cytochrome) inhibitor
0,277	0,128	CYP2E1 inducer
0,202	0,053	Arachidonic acid antagonist
0,197	0,049	Trans-pentaprenyltranstransferase inhibitor
0,182	0,034	Deoxyribose-phosphate aldolase inhibitor
0,212	0,064	Peptidyl-dipeptidase B inhibitor
0,265	0,117	Antiviral (Herpes)
0,190	0,042	Saccharolysin inhibitor
0,212	0,064	Guanosine-3',5'-bis(diphosphate) 3'-diphosphatase inhibitor
0,242	0,095	Thiamine pyridinylase inhibitor
0,187	0,039	Protein synthesis stimulant
0,185	0,039	3-Ketovalidoxylamine C-N-lyase inhibitor
0,175	0,028	Choloylglycine hydrolase inhibitor
0,191	0,044	Mannan endo-1,6-alpha-mannosidase inhibitor
0,271	0,124	Bilirubin oxidase inhibitor
0,226	0,081	Meprin B inhibitor
0,170	0,024	Aconitate decarboxylase inhibitor
0,252	0,107	Formate-dihydrofolate ligase inhibitor
0,244	0,099	Aldehyde dehydrogenase (pyrroloquinoline-quinone) inhibitor
0,206	0,061	(S)-3-amino-2-methylpropionate transaminase inhibitor
0,274	0,130	Gamma-guanidinobutyraldehyde dehydrogenase inhibitor
0,189	0,044	Testosterone 17beta-dehydrogenase inhibitor

0,226	0,084	DNA ligase (ATP) inhibitor
0,186	0,044	Polar-amino-acid-transporting ATPase inhibitor
0,173	0,032	Poly(3-hydroxybutyrate) depolymerase inhibitor
0,182	0,041	Allantoinase inhibitor
0,204	0,064	Sweetener
0,230	0,090	Phosphatidylcholine-sterol O-acyltransferase inhibitor
0,168	0,028	Glyceraldehyde-3-phosphate dehydrogenase (phosphorylating) inhibitor
0,177	0,037	Xylose isomerase inhibitor
0,146	0,006	Anesthetic inhalation
0,224	0,085	Glycine amidinotransferase inhibitor
0,254	0,115	Antiinflammatory, intestinal
0,214	0,075	CYP2A3 substrate
0,242	0,103	Leukotriene-B4 20-monooxygenase inhibitor
0,197	0,059	Alpha-amylase inhibitor
0,192	0,054	ADP-ribosylarginine hydrolase inhibitor
0,150	0,012	Fatty-acyl-ethyl-ester synthase inhibitor
0,179	0,041	Pancreatic endopeptidase E inhibitor
0,184	0,046	N-acetylneuraminate synthase inhibitor
0,246	0,108	Methylenetetrahydrofolate reductase (NADPH) inhibitor
0,282	0,144	Amine dehydrogenase inhibitor
0,343	0,206	Mucomembranous protector
0,189	0,053	Laminaribiose phosphorylase inhibitor
0,172	0,036	Glucarate dehydratase inhibitor
0,231	0,095	DNA-3-methyladenine glycosylase I inhibitor
0,250	0,114	4-Hydroxymandelate oxidase inhibitor
0,253	0,117	Nitrite reductase [NAD(P)H] inhibitor
0,175	0,040	Excitatory amino acid transporter EAAC1 inhibitor
0,146	0,011	Imidazoleglycerol-phosphate dehydratase inhibitor
0,175	0,040	Cholesterol oxidase inhibitor
0,197	0,063	4-Chlorobenzoyl-CoA dehalogenase inhibitor
0,165	0,031	Trimethyllysine dioxygenase inhibitor
0,173	0,039	Hepatocyte nuclear factor antagonist
0,173	0,039	Hepatocyte nuclear factor 4 alpha antagonist
0,154	0,020	Deoxycytidine deaminase inhibitor
0,276	0,142	Menopausal disorders treatment
0,179	0,046	Alpha-N-arabinofuranosidase inhibitor
0,237	0,104	Cis-1,2-dihydro-1,2-dihydroxynaphthalene dehydrogenase inhibitor

0,172	0,040	Asparagine-tRNA ligase inhibitor
0,183	0,051	Galactokinase inhibitor
0,250	0,118	Arylsulfate sulfotransferase inhibitor
0,207	0,075	Ubiquitin thiolesterase inhibitor
0,293	0,161	Nicotine dehydrogenase inhibitor
0,174	0,043	N-(5-amino-5-carboxypentanoyl)-L-cysteinyl-D-valine synthase inhibitor
0,146	0,016	Mycodextranase inhibitor
0,265	0,134	Mitochondrial processing peptidase inhibitor
0,193	0,063	Tentoxilysin inhibitor
0,205	0,075	Serine-pyruvate transaminase inhibitor
0,223	0,093	Antiprotozoal (Trichomonas)
0,168	0,039	Antineoplastic (gastric cancer)
0,161	0,031	4-Alpha-glucanotransferase inhibitor
0,191	0,061	Isopenicillin-N synthase inhibitor
0,240	0,111	ICAM1 expression inhibitor
0,162	0,033	(S)-carnitine 3-dehydrogenase inhibitor
0,205	0,076	Antileukemic
0,170	0,041	Acyl-lysine deacylase inhibitor
0,191	0,063	Antirickettsial
0,203	0,077	Cyclopropane-fatty-acyl-phospholipid synthase inhibitor
0,200	0,073	Signal peptidase I inhibitor
0,181	0,055	Ferrochelatase inhibitor
0,167	0,041	Serine 3-dehydrogenase inhibitor
0,141	0,016	Gentamicin 2''-nucleotidyltransferase inhibitor
0,182	0,057	Penicillopepsin inhibitor
0,158	0,033	Creatinase inhibitor
0,188	0,063	Glyoxylate reductase (NADP+) inhibitor
0,188	0,064	Sphinganine-1-phosphate aldolase inhibitor
0,193	0,068	Picornain 3C inhibitor
0,148	0,024	D-proline reductase (dithiol) inhibitor
0,159	0,036	Pregnane X receptor agonist
0,148	0,024	Shab potassium channel blocker
0,151	0,028	Thermomycolin inhibitor
0,150	0,027	Succinate dehydrogenase inhibitor
0,181	0,058	Choline-sulfatase inhibitor
0,190	0,067	3-Hydroxy-4-oxoquinoline 2,4-dioxygenase inhibitor
0,216	0,093	Uroporphyrinogen-III synthase inhibitor

0,168	0,047	Valine decarboxylase inhibitor
0,131	0,009	Osmotic diuretic
0,189	0,067	Glutamate 5-kinase inhibitor
0,187	0,066	Acaricide
0,159	0,039	Fructose-2,6-bisphosphate 2-phosphatase inhibitor
0,154	0,034	Antineoplastic, alkylator
0,195	0,075	Threonine aldolase inhibitor
0,183	0,063	N-acetylneuraminate 4-O-acetyltransferase inhibitor
0,141	0,021	Phosphatidylinositol 3-kinase stimulant
0,175	0,055	Galactose oxidase inhibitor
0,218	0,098	Coccolysin inhibitor
0,246	0,126	Alkane 1-monooxygenase inhibitor
0,215	0,096	Oxytocic
0,136	0,018	High-mannose-oligosaccharide beta-1,4-N-acetylglucosaminyltransferase inhibitor
0,151	0,033	Choline dehydrogenase inhibitor
0,206	0,088	Bothrolysin inhibitor
0,162	0,043	Methionine decarboxylase inhibitor
0,175	0,057	3-Carboxyethylcatechol 2,3-dioxygenase inhibitor
0,164	0,046	CDP-diacylglycerol-inositol 3-phosphatidyltransferase inhibitor
0,161	0,043	Glutamate-1-semialdehyde 2,1-aminomutase inhibitor
0,131	0,013	Acetylcholine M3 receptor agonist
0,201	0,083	Diabetic nephropathy treatment
0,182	0,063	IgA-specific metalloendopeptidase inhibitor
0,144	0,026	Beta-amylase inhibitor
0,159	0,041	Carboxymethylenebutenolidase inhibitor
0,209	0,092	Nardilysin inhibitor
0,245	0,128	HMOX1 expression enhancer
0,172	0,056	Methyltransferase substrate
0,146	0,030	2-Dehydropantolactone reductase (A-specific) inhibitor
0,127	0,012	Alpha,alpha-trehalose phosphorylase inhibitor
0,131	0,016	11-Cis-retinyl-palmitate hydrolase inhibitor
0,190	0,075	CYP4A2 substrate
0,283	0,169	RNA-directed RNA polymerase inhibitor
0,170	0,056	Glutamate (mGluR5) agonist
0,194	0,080	2,6-Dihydroxypyridine 3-monooxygenase inhibitor
0,146	0,033	Nicotinamide-nucleotide adenylyltransferase inhibitor
0,176	0,063	Vomilenine glucosyltransferase inhibitor

0,148	0,036	D-arabinonolactone oxidase inhibitor
0,133	0,020	Alternansucrase inhibitor
0,233	0,121	RNA synthesis inhibitor
0,191	0,079	Phosphatidylglycerophosphatase inhibitor
0,180	0,068	Mitochondrial intermediate peptidase inhibitor
0,224	0,112	ATP phosphoribosyltransferase inhibitor
0,194	0,082	Antihematotoxic
0,159	0,047	Acetyl-CoA C-acyltransferase inhibitor
0,176	0,065	tRNA nucleotidyltransferase inhibitor
0,142	0,031	Glutamine-pyruvate transaminase inhibitor
0,294	0,184	2-Hydroxyquinoline 8-monooxygenase inhibitor
0,137	0,027	Rhodopsin kinase inhibitor
0,143	0,032	Valine dehydrogenase (NADP+) inhibitor
0,167	0,057	Geranylgeranyl-diphosphate geranylgeranyltransferase inhibitor
0,234	0,124	4-Methoxybenzoate monooxygenase (O-demethylating) inhibitor
0,217	0,108	CYP2C3 substrate
0,187	0,078	UGT2B10 substrate
0,180	0,071	UGT1A5 substrate
0,131	0,023	Homoserine O-succinyltransferase inhibitor
0,134	0,025	Fructuronate reductase inhibitor
0,134	0,025	Tagaturonate reductase inhibitor
0,142	0,034	DELTA24-sterol reductase inhibitor
0,187	0,080	2-Enoate reductase inhibitor
0,149	0,043	Phosphonoacetate hydrolase inhibitor
0,201	0,094	Methanol dehydrogenase inhibitor
0,140	0,033	Glucan 1,3-beta-glucosidase inhibitor
0,177	0,071	Microtubule formation inhibitor
0,200	0,094	Carnitinamidase inhibitor
0,140	0,035	Secretase alpha stimulant
0,140	0,035	Secretase stimulant
0,180	0,074	N-acetyl-gamma-glutamyl-phosphate reductase inhibitor
0,160	0,055	3-Oxoadipate enol-lactonase inhibitor
0,140	0,036	Malonate-semialdehyde dehydrogenase inhibitor
0,198	0,094	Hyponitrite reductase inhibitor
0,148	0,044	Glycosylphosphatidylinositol diacylglycerol-lyase inhibitor
0,121	0,017	2-Dehydro-3-deoxy-phosphogluconate aldolase inhibitor
0,123	0,019	Ca2+-transporting ATPase inhibitor

0,141	0,037	Deoxyribonuclease X inhibitor
0,180	0,076	Rhamnulose-1-phosphate aldolase inhibitor
0,186	0,083	Phenylalanine(histidine) transaminase inhibitor
0,133	0,030	Isomaltulose synthase inhibitor
0,224	0,121	AR expression inhibitor
0,230	0,127	Urethanase inhibitor
0,130	0,027	Ribose-5-phosphate-ammonia ligase inhibitor
0,170	0,067	3'-Nucleotidase inhibitor
0,200	0,097	Bontoxilysin inhibitor
0,268	0,165	Spermidine dehydrogenase inhibitor
0,181	0,079	CYP4F2 substrate
0,177	0,075	2,4-Diaminopentanoate dehydrogenase inhibitor
0,177	0,075	3-Hydroxybutyryl-CoA dehydrogenase inhibitor
0,177	0,075	Lysine 6-dehydrogenase inhibitor
0,168	0,066	Allantoin racemase inhibitor
0,148	0,046	Beta-mannosidase inhibitor
0,129	0,027	Arabinose isomerase inhibitor
0,111	0,010	Phosphatidylinositol 3-kinase beta inhibitor
0,115	0,014	Arabinogalactan endo-1,4-beta-galactosidase inhibitor
0,150	0,049	2-Oxoisovalerate dehydrogenase (acylating) inhibitor
0,229	0,128	Venom exonuclease inhibitor
0,185	0,085	Gallate decarboxylase inhibitor
0,111	0,012	Inositol 1,4,5-triphosphate receptor antagonist
0,139	0,040	Fructose-2,6-bisphosphate 6-phosphatase inhibitor
0,166	0,067	Calcium-sensing receptor agonist
0,167	0,068	Granzyme A inhibitor
0,205	0,107	Eye irritation, inactive
0,208	0,111	Antifibrinolytic
0,283	0,186	Preneoplastic conditions treatment
0,123	0,026	2-Methyleneglutarate mutase inhibitor
0,219	0,123	4-Nitrophenylphosphatase inhibitor
0,160	0,065	Homoserine dehydrogenase inhibitor
0,111	0,015	Alpha,alpha-phosphotrehalase inhibitor
0,215	0,120	Myosin ATPase inhibitor
0,126	0,031	3-Deoxy-8-phosphooctulonate synthase inhibitor
0,147	0,052	D-lactate dehydrogenase inhibitor
0,136	0,040	Cytochrome-c3 hydrogenase inhibitor

0,169	0,073	Protein synthesis inhibitor
0,142	0,047	Lactaldehyde reductase (NADPH) inhibitor
0,152	0,057	Diisopropyl-fluorophosphatase inhibitor
0,138	0,044	Omega-amidase inhibitor
0,142	0,048	CYP19 substrate
0,158	0,064	Cerebroside-sulfatase inhibitor
0,132	0,039	Phosphofructokinase-1 inhibitor
0,132	0,039	Sorbose dehydrogenase inhibitor
0,167	0,074	Lactate 2-monooxygenase inhibitor
0,101	0,008	Glutamate (mGluR3) agonist
0,150	0,057	Glutathione S-transferase substrate
0,193	0,100	Nucleoside oxidase (H2O2-forming) inhibitor
0,166	0,073	Tetrahydroxynaphthalene reductase inhibitor
0,175	0,082	Biotin-CoA ligase inhibitor
0,175	0,082	Triacetate-lactonase inhibitor
0,175	0,082	6-Carboxyhexanoate-CoA ligase inhibitor
0,175	0,082	Homoaconitate hydratase inhibitor
0,158	0,066	Lipotropic
0,177	0,085	Camphor 1,2-monooxygenase inhibitor
0,132	0,040	Gluconate dehydratase inhibitor
0,165	0,073	Pyruvate dehydrogenase (cytochrome) inhibitor
0,165	0,073	Trans-2-enoyl-CoA reductase (NAD+) inhibitor
0,176	0,084	Metallocarboxypeptidase D inhibitor
0,145	0,054	GABA inverse agonist
0,145	0,054	GABA A inverse agonist
0,182	0,090	Antihypotensive
0,158	0,067	Gly-X carboxypeptidase inhibitor
0,282	0,191	Sulfur reductase inhibitor
0,127	0,036	1,5-Anhydro-D-fructose reductase inhibitor
0,164	0,073	N6-methyl-lysine oxidase inhibitor
0,117	0,027	2-Methylcitrate dehydratase inhibitor
0,194	0,103	Alcohol dehydrogenase [NAD(P)+] inhibitor
0,129	0,039	Glycerophosphocholine cholinephosphodiesterase inhibitor
0,114	0,025	Heme oxygenase inhibitor
0,156	0,067	Melanin inhibitor
0,115	0,026	Inositol-3-phosphate synthase inhibitor
0,093	0,004	Protein phosphatase 2A inhibitor

0,150	0,061	Iron-cytochrome-c reductase inhibitor
0,151	0,062	Cellulase inhibitor
0,119	0,030	Phosphatidylglycerol-membrane-oligosaccharide glycerophosphotransferase inhibitor
0,129	0,040	Aerobactin synthase inhibitor
0,167	0,078	Antineoplastic (renal cancer)
0,137	0,049	2-Aminoadipate transaminase inhibitor
0,127	0,039	2-Aminoethylphosphonate-pyruvate transaminase inhibitor
0,139	0,051	UDP-N-acetylglucosamine-dolichyl-phosphate N-acetylglucosaminephosphotransferase inhibitor
0,119	0,031	Platelet activating factor beta antagonist
0,151	0,063	Phosphoinositide phospholipase C inhibitor
0,131	0,043	DNA nucleotidylexotransferase inhibitor
0,187	0,100	Retinal dehydrogenase inhibitor
0,121	0,034	Ketohexokinase inhibitor
0,125	0,038	Beta-D-fucosidase inhibitor
0,121	0,034	Glyceraldehyde-3-phosphate dehydrogenase (NADP+) inhibitor
0,165	0,079	Carnosine synthase inhibitor
0,190	0,104	Glutaminase inhibitor
0,117	0,031	Beta-N-acetylgalactosaminidase inhibitor
0,117	0,031	CYP4F substrate
0,120	0,035	Proline dehydrogenase inhibitor
0,115	0,029	L-fuconate dehydratase inhibitor
0,133	0,047	Diacylglycerol kinase inhibitor
0,144	0,059	Allophanate hydrolase inhibitor
0,147	0,062	Valine-tRNA ligase inhibitor
0,132	0,047	Lysozyme inhibitor
0,143	0,058	Prepilin peptidase inhibitor
0,143	0,058	Haloacetate dehalogenase inhibitor
0,152	0,067	UGT2B18 substrate
0,151	0,066	Alpha-Methylacyl-CoA racemase inhibitor
0,143	0,058	Selenocysteine lyase inhibitor
0,107	0,022	Alpha,alpha-trehalose phosphorylase (configuration-retaining) inhibitor
0,201	0,116	CYP2B2 substrate
0,154	0,070	3-Aminobutyryl-CoA ammonia-lyase inhibitor
0,220	0,136	Cytochrome-b5 reductase inhibitor
0,297	0,213	TP53 expression enhancer
0,116	0,032	Ribonuclease U2 inhibitor

0,125	0,041	Homocysteine desulfhydrase inhibitor
0,210	0,126	Cyclic AMP modulator
0,115	0,031	Beta-glucosidase inhibitor
0,243	0,159	CYP3A3 substrate
0,113	0,029	Amino-acid racemase inhibitor
0,228	0,144	Tyrosine 3 hydroxylase inhibitor
0,121	0,037	Bis(5'-adenosyl)-triphosphatase inhibitor
0,120	0,037	2-Hydroxy-3-oxopropionate reductase inhibitor
0,201	0,119	UGT2B4 substrate
0,101	0,018	Chitin deacetylase inhibitor
0,135	0,053	NAD+ kinase inhibitor
0,164	0,082	GST M1-1 substrate
0,129	0,047	1-Aminocyclopropane-1-carboxylate deaminase inhibitor
0,135	0,054	Poly(ADP-ribose) glycohydrolase inhibitor
0,163	0,082	6-Pyruvoyltetrahydropterin synthase inhibitor
0,120	0,038	Dichloromuconate cycloisomerase inhibitor
0,108	0,026	Alpha-L-fucosidase inhibitor
0,137	0,055	Mevalonate kinase inhibitor
0,156	0,076	Phenylacetate-CoA ligase inhibitor
0,116	0,035	3-Oxosteroid 1-dehydrogenase inhibitor
0,154	0,073	SMN2 expression enhancer
0,149	0,068	Aldehyde ferredoxin oxidoreductase inhibitor
0,109	0,029	Alpha-L-rhamnosidase inhibitor
0,181	0,101	Sulfite reductase inhibitor
0,166	0,086	Penicillin amidase inhibitor
0,138	0,058	Urolithiasis treatment
0,163	0,083	4-Hydroxyglutamate transaminase inhibitor
0,173	0,093	Urate-ribonucleotide phosphorylase inhibitor
0,142	0,062	Galactose 1-dehydrogenase (NADP+) inhibitor
0,142	0,062	Beta-alanine-pyruvate transaminase inhibitor
0,116	0,036	N-acetylneuraminate lyase inhibitor
0,154	0,074	2-Acylglycerol O-acyltransferase inhibitor
0,095	0,016	NMDA receptor polyamine site agonist
0,136	0,057	ATP adenylyltransferase inhibitor
0,141	0,062	8-Amino-7-oxononanoate synthase inhibitor
0,136	0,057	CYP26A substrate
0,114	0,035	Gluconokinase inhibitor

0,206	0,128	Indanol dehydrogenase inhibitor
0,137	0,059	Agmatinase inhibitor
0,142	0,064	Thiamine-phosphate kinase inhibitor
0,125	0,047	Glutamate N-acetyltransferase inhibitor
0,103	0,025	(S)-2-Methylmalate dehydratase inhibitor
0,122	0,044	NMDA receptor polyamine site antagonist
0,161	0,083	Creatinine deaminase inhibitor
0,157	0,079	Allyl-alcohol dehydrogenase inhibitor
0,208	0,131	Gingipain K inhibitor
0,263	0,186	Antipruritic, allergic
0,126	0,048	Heparan-alpha-glucosaminide N-acetyltransferase inhibitor
0,127	0,050	Guanidinodeoxy-scyllo-inositol-4-phosphatase inhibitor
0,138	0,061	Biotin carboxylase inhibitor
0,190	0,112	Aromatic-hydroxylamine O-acetyltransferase inhibitor
0,141	0,063	Aminocarboxymuconate-semialdehyde decarboxylase inhibitor
0,158	0,081	Lipoxygenase substrate
0,178	0,101	Dihydroxy-acid dehydratase inhibitor
0,169	0,092	Antiprotozoal (Plasmodium)
0,164	0,087	Acetylornithine deacetylase inhibitor
0,174	0,097	Coenzyme-B sulfoethylthiotransferase inhibitor
0,125	0,049	Galactoside 2-alpha-L-fucosyltransferase inhibitor
0,163	0,088	Aldehyde dehydrogenase 2 substrate
0,241	0,166	Retinoic acid metabolism inhibitor
0,159	0,083	2,4-Dichlorophenol 6-monooxygenase inhibitor
0,112	0,037	2-Dehydro-3-deoxy-L-arabinonate dehydratase inhibitor
0,181	0,106	Opine dehydrogenase inhibitor
0,157	0,082	UGT2B9 substrate
0,164	0,090	Glutathione peroxidase inhibitor
0,092	0,018	Alpha-mannosidase inhibitor
0,108	0,034	Squalene-hopene cyclase inhibitor
0,105	0,031	Xylan 1,4-beta-xylosidase inhibitor
0,203	0,130	Gamma-glutamyltransferase inhibitor
0,186	0,112	Histidinol dehydrogenase inhibitor
0,155	0,082	Aspartoacylase inhibitor
0,120	0,047	UDP-glucose 4-epimerase inhibitor
0,123	0,050	Pantothenase inhibitor
0,140	0,067	Phenylpyruvate decarboxylase inhibitor

0,153	0,081	Adenylate cyclase stimulant
0,165	0,092	Antihelmintic (Fasciola)
0,186	0,114	Ferredoxin hydrogenase inhibitor
0,107	0,035	Galactosylgalactosylglucosylceramidase inhibitor
0,114	0,042	Globoside alpha-N-acetylgalactosaminyltransferase inhibitor
0,244	0,172	Superoxide dismutase inhibitor
0,115	0,043	Lysine decarboxylase inhibitor
0,109	0,037	3-Isopropylmalate dehydrogenase inhibitor
0,096	0,024	CYP4F12 substrate
0,121	0,050	Chondroitin 6-sulfotransferase inhibitor
0,134	0,062	O-acetylhomoserine aminocarboxypropyltransferase inhibitor
0,134	0,062	Interferon gamma antagonist
0,136	0,065	Interferon antagonist
0,147	0,076	Aldehyde dehydrogenase (NADP+) inhibitor
0,170	0,099	Indoleacetaldoxime dehydratase inhibitor
0,131	0,060	Isovaleryl-CoA dehydrogenase inhibitor
0,151	0,081	Pyridoxine 4-oxidase inhibitor
0,111	0,041	Cell wall synthesis inhibitor
0,141	0,071	2-Oxoglutarate decarboxylase inhibitor
0,120	0,051	Leucine transaminase inhibitor
0,226	0,156	4-Hydroxyproline epimerase inhibitor
0,252	0,182	N-hydroxyarylamine O-acetyltransferase inhibitor
0,187	0,118	Peroxidase substrate
0,132	0,063	Glucan endo-1,3-alpha-glucosidase inhibitor
0,157	0,088	CDP-4-dehydro-6-deoxyglucose reductase inhibitor
0,122	0,052	UGT2B11 substrate
0,160	0,091	Glycine C-acetyltransferase inhibitor
0,099	0,030	Long-chain-fatty-acid-CoA ligase inhibitor
0,117	0,048	D-glutamate oxidase inhibitor
0,102	0,034	Homospermidine synthase inhibitor
0,097	0,028	Alpha-1,6-mannosyl-glycoprotein 6-beta-N-acetylglucosaminyltransferase inhibitor
0,129	0,061	NAD(P)+ transhydrogenase (B-specific) inhibitor
0,128	0,060	Neuropsin inhibitor
0,160	0,092	Stromelysin 2 inhibitor
0,158	0,091	Hydroxymethylglutaryl-CoA lyase inhibitor
0,160	0,092	Arylacetonitrilase inhibitor
0,159	0,092	Dolichol kinase inhibitor

0,072	0,005	Antimitotic, Podophyllotoxin-like
0,285	0,218	Taurine dehydrogenase inhibitor
0,101	0,035	[hydroxymethylglutaryl-CoA reductase (NADPH)] kinase inhibitor
0,139	0,072	CYP4B substrate
0,124	0,058	L-lysine 6-transaminase inhibitor
0,221	0,154	Analgesic stimulant
0,087	0,020	3-Alpha-hydroxysteroid dehydrogenase (B-specific) inhibitor
0,198	0,132	Cathepsin T inhibitor
0,131	0,065	(S)-2-hydroxy-acid oxidase inhibitor
0,128	0,062	Iduronate-2-sulfatase inhibitor
0,124	0,058	Glycoprotein 3-alpha-L-fucosyltransferase inhibitor
0,098	0,033	6-Phosphofructo-2-kinase inhibitor
0,126	0,060	D-2-hydroxy-acid dehydrogenase inhibitor
0,179	0,114	Indolepyruvate C-methyltransferase inhibitor
0,171	0,106	Mucolytic
0,147	0,082	Aminopeptidase Y inhibitor
0,127	0,063	N4-(beta-N-acetylglucosaminyl)-L-asparaginase inhibitor
0,235	0,171	Loop diuretic
0,104	0,039	Phosphoenolpyruvate carboxykinase (diphosphate) inhibitor
0,156	0,092	CYP2D1 substrate
0,134	0,070	Antineoplastic (cervical cancer)
0,106	0,042	Monocarboxylic acid transporter 1 inhibitor
0,106	0,042	Monocarboxylic acid transporter inhibitor
0,083	0,020	ATP diphosphatase inhibitor
0,146	0,083	Plasmanylethanolamine desaturase inhibitor
0,197	0,134	Methane monooxygenase inhibitor
0,147	0,084	Tryptophan transaminase inhibitor
0,128	0,066	Antineoplastic (uterine cancer)
0,070	0,007	CYP4F8 substrate
0,144	0,081	Glucosyl transferase inhibitor
0,077	0,014	Sucrose-phosphate phosphatase inhibitor
0,105	0,042	Cysteine transaminase inhibitor
0,198	0,135	Sigma receptor agonist
0,139	0,077	Antibacterial, ophthalmic
0,091	0,028	CYP2A13 substrate
0,155	0,093	Inorganic diphosphatase inhibitor
0,139	0,077	Chitin synthase inhibitor

0,094	0,032	3-Oxoacyl-[acyl-carrier-protein] synthase inhibitor
0,115	0,053	Aspartate-ammonia ligase (ADP-forming) inhibitor
0,247	0,185	Antiinflammatory, ophthalmic
0,129	0,068	Pantoate 4-dehydrogenase inhibitor
0,152	0,091	N-methyl-2-oxoglutaramate hydrolase inhibitor
0,140	0,079	Adenylate kinase inhibitor
0,120	0,059	L-galactonolactone oxidase inhibitor
0,093	0,032	Amylo-alpha-1,6-glucosidase inhibitor
0,122	0,061	D-lactate dehydrogenase (cytochrome) inhibitor
0,099	0,038	(R)-aminopropanol dehydrogenase inhibitor
0,150	0,090	L-glucuronate reductase inhibitor
0,130	0,070	CYP4B1 substrate
0,088	0,028	Peptidoglycan beta-N-acetylmuramidase inhibitor
0,185	0,126	Tankyrase inhibitor
0,098	0,039	Malate dehydrogenase (NADP+) inhibitor
0,128	0,069	Pyruvate dehydrogenase inhibitor
0,090	0,031	4a-Hydroxytetrahydrobiopterin dehydratase inhibitor
0,109	0,050	Leucine-tRNA ligase inhibitor
0,149	0,090	Polynucleotide 5'-hydroxy-kinase inhibitor
0,140	0,081	Peptidylamidoglycolate lyase inhibitor
0,125	0,066	Carnitine 3-dehydrogenase inhibitor
0,148	0,089	Cytochrome-b5 reductase substrate
0,101	0,043	Retinol O-fatty-acyltransferase inhibitor
0,095	0,036	Epoxide hydrolase substrate
0,101	0,042	Cellobiose phosphorylase inhibitor
0,138	0,080	Methylaspartate ammonia-lyase inhibitor
0,111	0,052	Adenylylsulphatase inhibitor
0,081	0,022	1-Alkyl-2-acetylglycerol O-acyltransferase inhibitor
0,223	0,165	Antiviral (Influenza)
0,189	0,131	Acetylserotonin O-methyltransferase inhibitor
0,086	0,029	Aldose 1-epimerase inhibitor
0,136	0,079	HIV-2 reverse transcriptase inhibitor
0,089	0,032	Steroid sulfotransferase inhibitor
0,118	0,060	Ribonuclease inhibitor
0,118	0,061	Aldehyde dehydrogenase [NAD(P)+] inhibitor
0,187	0,130	1-Alkylglycerophosphocholine O-acetyltransferase inhibitor
0,112	0,055	Aspartate transaminase inhibitor

0,097	0,040	Argininosuccinate lyase inhibitor
0,147	0,090	Benzoylformate decarboxylase inhibitor
0,097	0,040	Ribulose-bisphosphate carboxylase inhibitor
0,187	0,130	Antibacterial
0,116	0,059	Cholate-CoA ligase inhibitor
0,145	0,088	Brachyurin inhibitor
0,115	0,059	Pyrimidine-deoxynucleoside 2'-dioxygenase inhibitor
0,103	0,046	UDP-N-acetylglucosamine diphosphorylase inhibitor
0,195	0,139	X-methyl-His dipeptidase inhibitor
0,193	0,137	Acidifying agent non gastric
0,102	0,046	5-Oxoprolinase (ATP-hydrolysing) inhibitor
0,155	0,099	3-Demethylubiquinone-9 3-O-methyltransferase inhibitor
0,119	0,063	Diacylglycerol cholinephosphotransferase inhibitor
0,119	0,063	NADH dehydrogenase inhibitor
0,146	0,090	Acetate kinase inhibitor
0,130	0,075	Ethanolaminephosphotransferase inhibitor
0,078	0,023	Mannosidase inhibitor
0,088	0,033	Aspartate 1-decarboxylase inhibitor
0,139	0,084	Oligopeptidase B inhibitor
0,072	0,017	Phosphatidylinositol 3-kinase alpha inhibitor
0,096	0,042	L-lysine oxidase inhibitor
0,071	0,017	Biotin-[methylmalonyl-CoA-carboxytransferase] ligase inhibitor
0,121	0,067	Sulfate adenylyltransferase (ADP) inhibitor
0,187	0,133	Prostaglandin-E2 9-reductase inhibitor
0,087	0,033	Lactate-malate transhydrogenase inhibitor
0,105	0,051	Inositol-polyphosphate 5-phosphatase inhibitor
0,169	0,116	Aminomuconate-semialdehyde dehydrogenase inhibitor
0,198	0,145	Chloride channel activator
0,107	0,053	Dihydrouracil oxidase inhibitor
0,086	0,033	3-Dehydroquinate synthase inhibitor
0,115	0,062	Alkanal monooxygenase (FMN-linked) inhibitor
0,086	0,033	mRNA (guanine-N7-)-methyltransferase inhibitor
0,122	0,069	Glycogen (starch) synthase inhibitor
0,155	0,103	Guanidinoacetate kinase inhibitor
0,124	0,072	Skin whitener
0,070	0,017	Prostaglandin-I synthase inhibitor
0,267	0,215	Intermittent claudication treatment

0,073	0,021	Glucan 1,4-alpha-glucosidase inhibitor
0,190	0,138	Glutaminyl-peptide cyclotransferase inhibitor
0,095	0,044	Phenylalanine-tRNA ligase inhibitor
0,106	0,055	Acyloxyacyl hydrolase inhibitor
0,116	0,066	Anabolic
0,088	0,038	Glutaminyl-tRNA synthase (glutamine-hydrolysing) inhibitor
0,085	0,035	CYP2C2 substrate
0,070	0,020	Amylosucrase inhibitor
0,098	0,049	Galactosylceramide sulfotransferase inhibitor
0,116	0,067	Cysteine desulfurase inhibitor
0,106	0,056	tRNA (cytosine-5-)-methyltransferase inhibitor
0,101	0,051	Benzaldehyde dehydrogenase (NAD+) inhibitor
0,143	0,094	Hydroxymethylbilane synthase inhibitor
0,199	0,150	CYP2C6 substrate
0,108	0,060	Allantoicase inhibitor
0,119	0,070	Beta lactamase inhibitor
0,112	0,064	Glycerol-3-phosphate O-acyltransferase inhibitor
0,091	0,043	2-Methylcitrate synthase inhibitor
0,109	0,061	DNA-3-methyladenine glycosylase II inhibitor
0,124	0,076	Aryl-alcohol dehydrogenase inhibitor
0,098	0,050	Deoxyribonuclease (pyrimidine dimer) inhibitor
0,081	0,033	Carnitine O-octanoyltransferase inhibitor
0,127	0,079	Sulfur dioxygenase inhibitor
0,077	0,029	Beta galactosidase inhibitor
0,202	0,154	NADPH-ferrihemoprotein reductase inhibitor
0,058	0,010	Sucrose phosphorylase inhibitor
0,102	0,054	Mannonate dehydratase inhibitor
0,095	0,047	Saccharopine dehydrogenase (NAD+, L-glutamate-forming) inhibitor
0,070	0,022	UGT2B4E substrate
0,070	0,022	UGT2B30 substrate
0,070	0,022	UGT2B19 substrate
0,070	0,022	UGT2B23 substrate
0,070	0,022	UGT2B4D substrate
0,114	0,067	Aspartate kinase inhibitor
0,134	0,086	(R,R)-butanediol dehydrogenase inhibitor
0,192	0,145	Antineoplastic (brain cancer)
0,208	0,160	FMO1 substrate

0,126	0,078	Protein-tyrosine sulfotransferase inhibitor
0,083	0,035	Pyruvate, phosphate dikinase inhibitor
0,115	0,068	5 Hydroxytryptamine 4A antagonist
0,078	0,032	[acetyl-CoA carboxylase] kinase inhibitor
0,070	0,023	Glucan 1,3-alpha-glucosidase inhibitor
0,136	0,089	Glutamate synthase (ferredoxin) inhibitor
0,191	0,144	Trimethylamine dehydrogenase inhibitor
0,072	0,025	D-lactate-2-sulfatase inhibitor
0,171	0,124	Pitrilysin inhibitor
0,135	0,089	N-Acyl-D-amino-acid deacylase inhibitor
0,185	0,139	L-threonine 3-dehydrogenase inhibitor
0,137	0,091	3,4-Dihydroxy-9,10-secoandrosta-1,3,5(10)-triene-9,17-dione 4,5-dioxygenase inhibitor
0,084	0,038	4-Hydroxy-4-methyl-2-oxoglutarate aldolase inhibitor
0,064	0,018	D(-)-tartrate dehydratase inhibitor
0,086	0,041	Glycerophosphocholine phosphodiesterase inhibitor
0,082	0,037	3(or 17)alpha-hydroxysteroid dehydrogenase inhibitor
0,080	0,035	Lactaldehyde dehydrogenase inhibitor
0,143	0,099	Tyrosine kinase stimulant
0,114	0,070	CYP19 inhibitor
0,087	0,043	Aspartate-semialdehyde dehydrogenase inhibitor
0,072	0,029	Acidifying agent gastric
0,127	0,084	Cysteine synthase inhibitor
0,071	0,028	CYP24 inhibitor
0,136	0,093	(S)-3-hydroxyacid ester dehydrogenase inhibitor
0,239	0,196	CYP19A1 expression inhibitor
0,129	0,086	Candidapepsin inhibitor
0,090	0,047	2-Oxoaldehyde dehydrogenase (NAD+) inhibitor
0,084	0,041	ATP deaminase inhibitor
0,092	0,049	Scyllo-inosamine-4-phosphate amidinotransferase inhibitor
0,205	0,162	3C-like protease (Human coronavirus) inhibitor
0,088	0,046	Deoxycytidine kinase inhibitor
0,110	0,068	Glucose 1-dehydrogenase inhibitor
0,085	0,043	Polyamine biosynthesis inhibitor
0,085	0,044	Cyanate hydratase inhibitor
0,067	0,025	3'(2'),5'-Bisphosphate nucleotidase inhibitor
0,066	0,025	Aromatase inhibitor
0,151	0,110	Cyclopentanone monooxygenase inhibitor

0,103	0,062	Phosphatidate cytidylyltransferase inhibitor
0,124	0,083	Endoglycosylceramidase inhibitor
0,093	0,053	UDP-N-acetylglucosamine 2-epimerase inhibitor
0,106	0,065	Site-specific DNA-methyltransferase (adenine-specific) inhibitor
0,068	0,028	D-Serine ammonia-lyase inhibitor
0,055	0,015	Tartrate decarboxylase inhibitor
0,253	0,213	Oxidoreductase inhibitor
0,086	0,046	Alpha,alpha-trehalose-phosphate synthase (UDP-forming) inhibitor
0,101	0,062	Ribitol 2-dehydrogenase inhibitor
0,105	0,067	Adenosylmethionine-8-amino-7-oxononanoate transaminase inhibitor
0,044	0,005	Progesterone receptor B antagonist
0,098	0,059	Thiamine-phosphate diphosphorylase inhibitor
0,097	0,058	Glycerol dehydratase inhibitor
0,156	0,117	Thermitase inhibitor
0,090	0,051	CYP26 substrate
0,094	0,056	Acetoacetate decarboxylase inhibitor
0,099	0,061	Cytosole dipeptidase inhibitor
0,071	0,033	Corticotropin releasing factor antagonist
0,208	0,170	Antiprotozoal (Coccidial)
0,175	0,137	Antiprotozoal
0,128	0,090	Tryptophanyl aminopeptidase inhibitor
0,142	0,104	4-Chlorophenylacetate 3,4-dioxygenase inhibitor
0,168	0,130	Diamine N-acetyltransferase inhibitor
0,126	0,088	3-Hydroxyphenylacetate 6-hydroxylase inhibitor
0,135	0,097	Pyruvate carboxylase inhibitor
0,122	0,084	Acylglycerone-phosphate reductase inhibitor
0,061	0,023	CYP17 substrate
0,061	0,024	Na+ K+ transporting ATPase inhibitor
0,134	0,097	4-Hydroxybenzoate nonaprenyltransferase inhibitor
0,174	0,137	O-aminophenol oxidase inhibitor
0,089	0,053	Necroptosis inhibitor
0,121	0,084	Gingipain R inhibitor
0,074	0,037	Glutathione reductase stimulant
0,074	0,037	Glutathione reductase (NADPH) stimulant
0,121	0,084	Galactoside O-acetyltransferase inhibitor
0,119	0,084	Arylalkyl acylamidase inhibitor
0,107	0,071	Aspartyl aminopeptidase inhibitor

0,150	0,114	Ferredoxin-nitrite reductase inhibitor
0,103	0,068	Alanine transaminase inhibitor
0,103	0,068	Malyl-CoA lyase inhibitor
0,100	0,065	Glycerol-3-phosphate dehydrogenase (NAD+) inhibitor
0,101	0,065	Glycoprotein-fucosylgalactoside alpha-N-acetylgalactosaminyltransferase inhibitor
0,065	0,030	Hydroxylysine kinase inhibitor
0,096	0,062	Alpha,alpha-trehalase inhibitor
0,081	0,046	Phosphoribulokinase inhibitor
0,257	0,223	Mannotetraose 2-alpha-N-acetylglucosaminyltransferase inhibitor
0,105	0,070	Squalene epoxidase inhibitor
0,133	0,098	Heat shock protein agonist
0,061	0,027	Propionate CoA-transferase inhibitor
0,073	0,039	Dextransucrase inhibitor
0,197	0,163	DNA synthesis inhibitor
0,073	0,039	Deoxyhypusine synthase inhibitor
0,070	0,036	Malate synthase inhibitor
0,079	0,045	3-Dehydroquinate dehydratase inhibitor
0,112	0,078	Narcotic antagonist
0,231	0,198	Polarisation stimulant
0,077	0,044	N-acetylglucosaminyldiphosphoundecaprenol N-acetyl-beta-D-mannosaminyltransferase inhibitor
0,087	0,054	Guanidinopropionase inhibitor
0,166	0,133	Severe acute respiratory syndrome treatment
0,047	0,014	Thymidine phosphorylase inhibitor
0,078	0,045	Dihydrolipoamide S-acetyltransferase inhibitor
0,063	0,031	CMP-KDO synthase inhibitor
0,077	0,044	Hydroperoxide dehydratase inhibitor
0,096	0,064	Isocitrate dehydrogenase (NAD+) inhibitor
0,035	0,003	3-Oxoacyl-[acyl-carrier-protein] synthase II inhibitor
0,251	0,219	Insulin promoter
0,062	0,030	Biotin-[propionyl-CoA-carboxylase (ATP-hydrolysing)] ligase inhibitor
0,097	0,065	Malate dehydrogenase (oxaloacetate-decarboxylating) inhibitor
0,154	0,123	Nicotinic alpha4beta2 receptor antagonist
0,099	0,067	Tyrosine-protein kinase TYRO 10 inhibitor
0,098	0,066	Glutamate (mGluR6) antagonist
0,210	0,178	Pyroglutamyl-peptidase II inhibitor
0,081	0,050	S-methyl-5-thioribose kinase inhibitor
0,213	0,182	Membrane integrity antagonist

0,074	0,042	Glycerol kinase inhibitor
0,125	0,094	Procollagen C-endopeptidase inhibitor
0,116	0,085	Cathepsin H inhibitor
0,102	0,071	Cerevisin inhibitor
0,133	0,103	Tryptophan alpha,beta-oxidase inhibitor
0,062	0,031	Glycine N-choloyltransferase inhibitor
0,107	0,077	CYP3C substrate
0,075	0,044	L-aminoadipate-semialdehyde dehydrogenase inhibitor
0,109	0,079	Glucosamine-6-phosphate deaminase inhibitor
0,105	0,074	Decylcitrate synthase inhibitor
0,065	0,035	Alpha-1,6-mannosyl-glycoprotein 2-beta-N-acetylglucosaminyltransferase inhibitor
0,063	0,033	Deoxyadenosine kinase inhibitor
0,079	0,050	N-acetylglucosamine-6-phosphate deacetylase inhibitor
0,100	0,070	D-Ornithine 4,5-aminomutase inhibitor
0,128	0,099	Lombricine kinase inhibitor
0,101	0,071	Diiodophenylpyruvate reductase inhibitor
0,136	0,107	Tryptophan dimethylallyltransferase inhibitor
0,050	0,021	Methylaspartate mutase inhibitor
0,072	0,043	UGT2B7H substrate
0,072	0,043	UGT2B7Y substrate
0,155	0,126	Sodium channel blocker class Ib
0,190	0,161	Monoamine uptake inhibitor
0,191	0,162	4-Hydroxyphenylacetate 3-monooxygenase inhibitor
0,082	0,054	Neolactotetraosylceramide alpha-2,3-sialyltransferase inhibitor
0,127	0,098	Peptide-tryptophan 2,3-dioxygenase inhibitor
0,114	0,086	3-Isopropylmalate dehydratase inhibitor
0,073	0,045	Glutamate (mGluR8) agonist
0,042	0,014	Homoisocitrate dehydrogenase inhibitor
0,082	0,054	Oligo-1,6-glucosidase inhibitor
0,089	0,061	CTP synthase inhibitor
0,103	0,075	D-cysteine desulfhydrase inhibitor
0,060	0,032	Sucrose alpha-glucosidase inhibitor
0,091	0,062	Astringent
0,049	0,021	Orotidylate decarboxylase inhibitor
0,103	0,075	Glucosamine-1-phosphate N-acetyltransferase inhibitor
0,074	0,047	Serine-tRNA ligase inhibitor
0,161	0,134	Corticosteroid side-chain-isomerase inhibitor

0,088	0,060	D-malate dehydrogenase (decarboxylating) inhibitor
0,066	0,039	Quinate 5-dehydrogenase inhibitor
0,070	0,043	3-Hydroxydecanoyl-[acyl-carrier-protein] dehydratase inhibitor
0,058	0,031	CYP24 substrate
0,067	0,040	GMP synthase inhibitor
0,082	0,056	Succinate-hydroxymethylglutarate CoA-transferase inhibitor
0,118	0,092	Homogentisate 1,2-dioxygenase inhibitor
0,040	0,014	Transaldolase inhibitor
0,070	0,043	Formimidoylglutamase inhibitor
0,132	0,105	Restless leg syndrome treatment
0,066	0,039	Thioether S-methyltransferase inhibitor
0,168	0,142	N-methylhydantoinase (ATP-hydrolysing) inhibitor
0,109	0,083	Phenylacetaldehyde dehydrogenase inhibitor
0,066	0,040	Phosphoglucomutase inhibitor
0,112	0,086	Acylphosphatase inhibitor
0,119	0,093	Succinic dehydrogenase inhibitor
0,065	0,039	Oxaloacetate tautomerase inhibitor
0,093	0,067	Formaldehyde dehydrogenase inhibitor
0,053	0,027	CYP3A11 substrate
0,058	0,033	Uracil phosphoribosyltransferase inhibitor
0,048	0,022	Androst-4-ene-3,17-dione monooxygenase inhibitor
0,140	0,114	Choline kinase inhibitor
0,071	0,046	Isocitrate dehydrogenase (NADP+) inhibitor
0,075	0,050	Maleate hydratase inhibitor
0,098	0,073	Sarcosine oxidase inhibitor
0,039	0,013	Uridine phosphorylase inhibitor
0,046	0,021	Isopentenyl-diphosphate DELTA-isomerase inhibitor
0,103	0,078	Asparaginase inhibitor
0,050	0,025	NADPH oxidoreductase inhibitor
0,075	0,050	ACK1 kinase inhibitor
0,084	0,059	CMP-N-acylneuraminate phosphodiesterase inhibitor
0,099	0,075	Aspartate racemase inhibitor
0,123	0,099	Narcolepsy treatment
0,081	0,057	Muconate cycloisomerase inhibitor
0,097	0,074	2-Nitrophenol 2-monooxygenase inhibitor
0,077	0,054	D-alanine transaminase inhibitor
0,104	0,081	Acetolactate decarboxylase inhibitor

0,083	0,061	Diguanidinobutanase inhibitor
0,090	0,068	Vascular endothelial growth factor antagonist
0,057	0,035	Aspartate ammonia-lyase inhibitor
0,102	0,080	Dethiobiotin synthase inhibitor
0,115	0,092	FMN reductase inhibitor
0,068	0,046	L-lactate dehydrogenase (cytochrome) inhibitor
0,067	0,044	UDP-glucose 6-dehydrogenase inhibitor
0,070	0,047	Nicotinic receptor alpha7 subunit antagonist
0,088	0,066	CYP2B4 substrate
0,080	0,058	tRNA (guanine-N2-)-methyltransferase inhibitor
0,103	0,081	Pyruvate dehydrogenase (lipoamide) inhibitor
0,101	0,080	Serine-phosphoethanolamine synthase inhibitor
0,079	0,058	SULT2A1 substrate
0,152	0,131	Expectorant
0,089	0,069	Orotate reductase (NADPH) inhibitor
0,185	0,165	Monodehydroascorbate reductase (NADH) inhibitor
0,109	0,089	Thiol S-methyltransferase inhibitor
0,043	0,022	Dichloromethane dehalogenase inhibitor
0,171	0,150	TRPA1 agonist
0,128	0,108	Glycerol dehydrogenase (NADP+) inhibitor
0,191	0,171	Immunomodulator
0,151	0,131	Gastrointestinal motility stimulant
0,086	0,067	Capillary fragility treatment
0,143	0,124	Laxative
0,053	0,033	AMP nucleosidase inhibitor
0,079	0,059	Cystathionine beta-lyase inhibitor
0,152	0,133	Histone acetyltransferase inhibitor
0,049	0,029	N-acetylglucosaminyldiphosphodolichol N-acetylglucosaminyltransferase inhibitor
0,135	0,115	Arylamine N-acetyltransferase inhibitor
0,074	0,055	Alpha glucosidase inhibitor
0,096	0,077	Aryl-aldehyde dehydrogenase (NADP+) inhibitor
0,039	0,020	Membrane-oligosaccharide glycerophosphotransferase inhibitor
0,252	0,234	Mucositis treatment
0,084	0,066	Diaminopropionate ammonia-lyase inhibitor
0,131	0,113	Quisqualate antagonist
0,159	0,141	Renal tissue kallikrein inhibitor
0,041	0,023	Phosphoglycerate phosphatase inhibitor

0,077	0,059	[myelin basic protein]-arginine N-methyltransferase inhibitor
0,063	0,045	Beta-1,4-mannosyl-glycoprotein 4-beta-N-acetylglucosaminyltransferase inhibitor
0,058	0,040	Plus-end-directed kinesin ATPase inhibitor
0,050	0,033	Anhydrotetracycline monooxygenase inhibitor
0,183	0,166	Calcium channel activator
0,045	0,028	Thymidine-triphosphatase inhibitor
0,057	0,040	Carbamoyl-serine ammonia-lyase inhibitor
0,092	0,075	Ornithine-oxo-acid transaminase inhibitor
0,158	0,142	Ribonucleoside triphosphate reductase inhibitor
0,073	0,056	Urocanate hydratase inhibitor
0,025	0,008	Progesterone receptor B1 antagonist
0,062	0,046	UDP-N-acetylmuramoylalanine-D-glutamate ligase inhibitor
0,097	0,081	Adenine deaminase inhibitor
0,122	0,106	Corticosteroid antagonist
0,076	0,060	Cushing's syndrome treatment
0,072	0,056	Carbamoyl-phosphate synthase (ammonia) inhibitor
0,070	0,054	Propanediol dehydratase inhibitor
0,119	0,103	Guanidinoacetate N-methyltransferase inhibitor
0,091	0,075	CMP-N-acetylneuraminate monooxygenase inhibitor
0,077	0,062	Guanylate kinase inhibitor
0,112	0,097	Cancer procoagulant inhibitor
0,104	0,089	Malate-CoA ligase inhibitor
0,060	0,045	Methylthioadenosine nucleosidase inhibitor
0,037	0,022	Glucose-6-phosphate isomerase inhibitor
0,104	0,090	Lysine 2-monooxygenase inhibitor
0,088	0,073	Triose-phosphate isomerase inhibitor
0,178	0,163	Antiparkinsonian, rigidity relieving
0,169	0,155	Dactylysin inhibitor
0,081	0,067	UDP-N-acetylglucosamine 1-carboxyvinyltransferase inhibitor
0,178	0,164	Antitoxic
0,156	0,141	D-amino-acid dehydrogenase inhibitor
0,053	0,039	Glutamate (mGluR1) agonist
0,030	0,016	Alpha glucosidase I inhibitor
0,126	0,112	Tryptophanamidase inhibitor
0,038	0,024	Endo-beta-N-acetylglucosaminidase inhibitor
0,064	0,051	4-Hydroxy-2-oxoglutarate aldolase inhibitor
0,058	0,045	Androgen antagonist

0,103	0,089	Cysteine-S-conjugate beta-lyase inhibitor
0,114	0,101	Isoquinoline 1-oxidoreductase inhibitor
0,080	0,066	Haloalkane dehalogenase inhibitor
0,128	0,115	Acetylspermidine deacetylase inhibitor
0,076	0,064	Carboxylate reductase inhibitor
0,100	0,087	Arginine deiminase inhibitor
0,059	0,047	Asparagine-oxo-acid transaminase inhibitor
0,224	0,212	Cancer associated disorders treatment
0,043	0,031	2-Ethylmalate synthase inhibitor
0,030	0,018	Phosphopyruvate hydratase inhibitor
0,094	0,082	Sulfate adenylyltransferase inhibitor
0,066	0,054	Cellulose synthase (UDP-forming) inhibitor
0,096	0,084	Photinus-luciferin 4-monooxygenase (ATP-hydrolysing) inhibitor
0,086	0,074	Agaritine gamma-glutamyltransferase inhibitor
0,078	0,067	Threonine ammonia-lyase inhibitor
0,092	0,081	Putrescine oxidase inhibitor
0,075	0,064	Gastricsin inhibitor
0,094	0,083	Xanthommatin reductase inhibitor
0,087	0,076	Streptopain inhibitor
0,048	0,037	Nucleoside deoxyribosyltransferase inhibitor
0,080	0,069	Nucleotide diphosphatase inhibitor
0,195	0,184	Aldehyde oxidase inhibitor
0,116	0,105	Dolichyl-phosphate beta-D-mannosyltransferase inhibitor
0,024	0,013	Dopamine D2B antagonist
0,054	0,044	Malate dehydrogenase (decarboxylating) inhibitor
0,100	0,090	Venombin A inhibitor
0,055	0,045	1-Phosphofructokinase inhibitor
0,053	0,043	Assemblin inhibitor
0,117	0,107	Phenylalanine racemase (ATP-hydrolysing) inhibitor
0,110	0,100	Polyamine oxidase inhibitor
0,052	0,043	Protein N-acetylglucosaminyltransferase inhibitor
0,051	0,042	Aldehyde dehydrogenase 1A2 inhibitor
0,127	0,118	UGT2B7 substrate
0,106	0,096	Prolactin inhibitor
0,018	0,009	Antiprotozoal activity enhancer
0,098	0,089	Coagulant
0,074	0,065	Isocitrate lyase inhibitor

0,075	0,066	[acyl-carrier-protein] S-malonyltransferase inhibitor
0,116	0,107	Quinoline-4-carboxylate 2-oxidoreductase inhibitor
0,090	0,081	Antineoplastic (squamous cell carcinoma)
0,056	0,047	Altronate dehydratase inhibitor
0,048	0,040	Tubulin GTPase inhibitor
0,043	0,035	Glucose-1-phosphate adenylyltransferase inhibitor
0,016	0,008	Kanamycin kinase inhibitor
0,049	0,041	D-Alanine-poly(phosphoribitol) ligase inhibitor
0,042	0,034	Fructose 5-dehydrogenase (NADP+) inhibitor
0,026	0,018	Tartrate dehydrogenase inhibitor
0,084	0,076	Neuropeptide FF2 agonist
0,084	0,076	Neuropeptide FF agonist
0,095	0,087	Pyridoxine 5-dehydrogenase inhibitor
0,039	0,032	Glucose-1-phosphatase inhibitor
0,108	0,101	Saccharopine dehydrogenase (NADP+, L-glutamate-forming) inhibitor
0,022	0,015	Cortisone alpha-reductase inhibitor
0,090	0,083	Glutamine-tRNA ligase inhibitor
0,142	0,135	Antinaupathic
0,070	0,063	4-Pyridoxolactonase inhibitor
0,207	0,200	Nicotinate dehydrogenase inhibitor
0,149	0,142	Beta-carotene 15,15'-monooxygenase inhibitor
0,068	0,061	1-Pyrroline-5-carboxylate dehydrogenase inhibitor
0,050	0,043	Chalcone isomerase inhibitor
0,135	0,128	Atrolysin A inhibitor
0,037	0,030	Kainate receptor agonist
0,069	0,063	Thiosulfate sulfurtransferase inhibitor
0,081	0,074	Glycine transaminase inhibitor
0,050	0,044	Pyruvate, water dikinase inhibitor
0,114	0,108	UGT2B15 substrate
0,069	0,063	Serine-glyoxylate transaminase inhibitor
0,119	0,113	N-Carbamoyl-D-amino acid hydrolase inhibitor
0,109	0,103	Protein-glutamate methylesterase inhibitor
0,012	0,006	Prostaglandin EP12 antagonist
0,080	0,075	Glycine hydroxymethyltransferase inhibitor
0,036	0,030	Glucosylceramidase inhibitor
0,038	0,033	Strombine dehydrogenase inhibitor
0,067	0,062	Aminolevulinate transaminase inhibitor

0,038	0,033	Sodium-dependent vitamin C transporter 2 inhibitor
0,088	0,083	Phosphoglycerate mutase inhibitor
0,043	0,038	Polyphosphate kinase inhibitor
0,129	0,125	Glucuronate isomerase inhibitor
0,087	0,083	Chloramphenicol O-acetyltransferase inhibitor
0,033	0,029	Ribose-5-phosphate adenylyltransferase inhibitor
0,054	0,050	Urate transporter 1 inhibitor
0,042	0,038	Carboxy-cis,cis-muconate cyclase inhibitor
0,065	0,061	Phosphoglycolate phosphatase inhibitor
0,058	0,054	Dihydrodipicolinate synthase inhibitor
0,073	0,069	Carboxypeptidase M inhibitor
0,033	0,029	RNA agonist
0,024	0,020	Dolichyldiphosphatase inhibitor
0,182	0,178	Choline-phosphate cytidylyltransferase inhibitor
0,043	0,040	Bis(5'-nucleosyl)-tetraphosphatase (symmetrical) inhibitor
0,032	0,029	Diphosphate-serine phosphotransferase inhibitor
0,037	0,034	Adenylate cyclase I inhibitor
0,024	0,021	Cathepsin S inhibitor
0,045	0,041	Glutamate uptake inhibitor
0,053	0,050	Aryl hydrocarbon receptor antagonist
0,102	0,100	Heat shock protein 70 agonist
0,016	0,013	CYP11B1 substrate
0,034	0,032	Phosphoserine transaminase inhibitor
0,096	0,094	Reductase inhibitor
0,111	0,109	Diphosphomevalonate decarboxylase inhibitor
0,036	0,034	[Isocitrate dehydrogenase (NADP+)] kinase inhibitor
0,074	0,072	Alcohol oxidase inhibitor
0,123	0,121	Phosphatidate phosphatase inhibitor
0,098	0,096	Deoxycytidylate 5-hydroxymethyltransferase inhibitor
0,058	0,056	Citrate (Si)-synthase inhibitor
0,143	0,142	Histidinol-phosphatase inhibitor
0,222	0,220	CYP3A5 substrate
0,027	0,025	Glutamate (mGluR5a) agonist
0,027	0,025	Glutamate (mGluR1a) agonist
0,058	0,057	Fumarylacetoacetase inhibitor
0,161	0,160	Hydroxylamine reductase inhibitor
0,088	0,087	Lysyl endopeptidase inhibitor

0,093	0,093	Estrone sulfotransferase inhibitor
0,123	0,122	MDM2 inhibitor
0,058	0,058	NMN nucleosidase inhibitor
0,044	0,044	Mannosyl-oligosaccharide glucosidase inhibitor
0,140	0,140	UGT1A1 substrate

Complex 3 All

All Pa>Pi Pa>0,3 Pa>0,7

Pa	Pi	Activity
0,850	0,001	Prostaglandin E1 antagonist
0,836	0,004	Ophthalmic drug
0,824	0,003	Antiglaucomic
0,831	0,023	Phobic disorders treatment
0,799	0,011	Nicotinic alpha6beta3beta4alpha5 receptor antagonist
0,767	0,002	Gestagen antagonist
0,760	0,013	5-O-(4-coumaroyl)-D-quinate 3'-monooxygenase inhibitor
0,778	0,031	Testosterone 17beta-dehydrogenase (NADP+) inhibitor
0,760	0,016	Nicotinic alpha2beta2 receptor antagonist
0,760	0,029	Acrocylindropepsin inhibitor
0,760	0,029	Saccharopepsin inhibitor
0,760	0,029	Chymosin inhibitor
0,720	0,008	Glucan 1,4-alpha-maltotriohydrolase inhibitor
0,671	0,005	Antineoplastic (non-Hodgkin's lymphoma)
0,696	0,034	Sugar-phosphatase inhibitor
0,683	0,022	Alkylacetylglycerophosphatase inhibitor
0,689	0,028	Acylcarnitine hydrolase inhibitor
0,665	0,008	Insulin promoter
0,702	0,050	CYP2J substrate
0,642	0,005	Imidazoline receptor agonist
0,677	0,045	Polyporopepsin inhibitor
0,696	0,070	Ubiquinol-cytochrome-c reductase inhibitor
0,657	0,037	Glutamyl endopeptidase II inhibitor
0,646	0,030	Ribulose-phosphate 3-epimerase inhibitor

0,683	0,069	Aspulvinone dimethylallyltransferase inhibitor
0,649	0,047	CYP2J2 substrate
0,635	0,039	NADPH peroxidase inhibitor
0,618	0,024	Cytoprotectant
0,616	0,023	27-Hydroxycholesterol 7alpha-monooxygenase inhibitor
0,624	0,033	Complement factor D inhibitor
0,595	0,004	Pediculicide
0,592	0,011	Dolichyl-diphosphooligosaccharide-protein glycotransferase inhibitor
0,579	0,005	Antineoplastic (ovarian cancer)
0,584	0,029	Chloride peroxidase inhibitor
0,595	0,046	Alkenylglycerophosphocholine hydrolase inhibitor
0,596	0,051	TP53 expression enhancer
0,550	0,005	CYP2B11 substrate
0,560	0,019	Mannan endo-1,4-beta-mannosidase inhibitor
0,572	0,033	Gastrin inhibitor
0,563	0,027	L-glutamate oxidase inhibitor
0,596	0,066	Glycosylphosphatidylinositol phospholipase D inhibitor
0,550	0,020	CYP2A4 substrate
0,574	0,061	Phosphatase inhibitor
0,513	0,009	Imidazoline I1 receptor agonist
0,546	0,046	Platelet aggregation stimulant
0,537	0,044	JAK2 expression inhibitor
0,549	0,060	Omptin inhibitor
0,518	0,033	Antipruritic, allergic
0,530	0,048	UDP-N-acetylglucosamine 4-epimerase inhibitor
0,528	0,047	Glucan endo-1,6-beta-glucosidase inhibitor
0,536	0,056	Lysase inhibitor
0,522	0,043	Pterin deaminase inhibitor
0,531	0,052	Macrophage colony stimulating factor agonist
0,502	0,027	Glyoxylate reductase inhibitor
0,493	0,019	Albendazole monooxygenase inhibitor
0,521	0,049	Glucan endo-1,3-beta-D-glucosidase inhibitor
0,521	0,049	Venombin AB inhibitor
0,498	0,031	Pullulanase inhibitor
0,489	0,022	Cutinase inhibitor
0,484	0,018	Dementia treatment
0,474	0,009	Acaricide

0,523	0,058	Antidyskinetic
0,498	0,033	CYP2B5 substrate
0,510	0,052	Limulus clotting factor B inhibitor
0,491	0,038	3-Hydroxybenzoate 6-monooxygenase inhibitor
0,492	0,039	Respiratory analeptic
0,505	0,058	Carboxypeptidase Taq inhibitor
0,475	0,028	Peptidoglycan glycosyltransferase inhibitor
0,508	0,061	Electron-transferring-flavoprotein dehydrogenase inhibitor
0,494	0,047	Feruloyl esterase inhibitor
0,479	0,033	IgA-specific serine endopeptidase inhibitor
0,522	0,079	Pseudolysin inhibitor
0,476	0,034	All-trans-retinyl-palmitate hydrolase inhibitor
0,455	0,013	RNA directed DNA polymerase inhibitor
0,488	0,046	Exoribonuclease II inhibitor
0,520	0,084	Kidney function stimulant
0,445	0,010	Endo-1,3(4)-beta-glucanase inhibitor
0,485	0,051	1,4-Lactonase inhibitor
0,503	0,071	Phthalate 4,5-dioxygenase inhibitor
0,472	0,045	Cyclic AMP agonist
0,496	0,069	Platelet adhesion inhibitor
0,463	0,039	TNF expression inhibitor
0,445	0,020	Hematopoietic inhibitor
0,459	0,035	Na+-transporting two-sector ATPase inhibitor
0,485	0,062	GST A substrate
0,543	0,121	Membrane permeability inhibitor
0,453	0,031	Aspergillopepsin I inhibitor
0,469	0,051	Hydrogen dehydrogenase inhibitor
0,462	0,046	Gluconate 5-dehydrogenase inhibitor
0,466	0,050	Trimethylamine-oxide aldolase inhibitor
0,430	0,017	Oxidizing agent
0,442	0,031	Cardiovascular analeptic
0,424	0,012	Sweetener
0,480	0,069	Phospholipid-translocating ATPase inhibitor
0,448	0,041	Acetylesterase inhibitor
0,487	0,083	Acetylcholine neuromuscular blocking agent
0,438	0,037	Simian immunodeficiency virus proteinase inhibitor
0,463	0,063	Phosphatidylcholine-retinol O-acyltransferase inhibitor

0,411	0,013	Uterine relaxant
0,398	0,002	Transactivator transcription protein inhibitor
0,490	0,096	Fusarinine-C ornithinesterase inhibitor
0,497	0,103	Membrane integrity agonist
0,418	0,025	Xylan endo-1,3-beta-xylosidase inhibitor
0,451	0,060	Arginine 2-monooxygenase inhibitor
0,412	0,024	Taurine-2-oxoglutarate transaminase inhibitor
0,435	0,048	Alkenylglycerophosphoethanolamine hydrolase inhibitor
0,409	0,022	Sorbitol-6-phosphate 2-dehydrogenase inhibitor
0,469	0,083	Protein-disulfide reductase (glutathione) inhibitor
0,426	0,041	Limulus clotting factor C inhibitor
0,458	0,074	Thioredoxin inhibitor
0,388	0,007	Insecticide
0,453	0,072	Alopecia treatment
0,413	0,033	Sulfite oxidase inhibitor
0,394	0,015	Antipruritic, non-allergic
0,410	0,032	Glucan 1,4-alpha-maltotetraohydrolase inhibitor
0,432	0,056	Cl--transporting ATPase inhibitor
0,425	0,048	Analeptic
0,423	0,048	Acetylgalactosaminyl-O-glycosyl-glycoprotein beta-1,3-N-acetylglucosaminyltransferase inhibitor
0,404	0,030	H+-exporting ATPase inhibitor
0,404	0,032	Poly(beta-D-mannuronate) lyase inhibitor
0,467	0,095	CYP3A2 substrate
0,411	0,040	EIF4E expression inhibitor
0,400	0,030	Glycolate dehydrogenase inhibitor
0,393	0,023	CDP-diacylglycerol-glycerol-3-phosphate 3-phosphatidyltransferase inhibitor
0,396	0,027	Shikimate O-hydroxycinnamoyltransferase inhibitor
0,433	0,065	Fatty-acyl-CoA synthase inhibitor
0,423	0,055	Formaldehyde transketolase inhibitor
0,414	0,048	Cyclohexanone monooxygenase inhibitor
0,430	0,064	Fragilysin inhibitor
0,416	0,051	N-acetylneuraminate 7-O(or 9-O)-acetyltransferase inhibitor
0,417	0,054	Antipruritic
0,422	0,058	Apyrase inhibitor
0,365	0,003	Antineoplastic (bone cancer)
0,434	0,072	Lysine 2,3-aminomutase inhibitor
0,416	0,055	CYP2A8 substrate

0,373	0,012	Vascular dementia treatment
0,362	0,004	CYP2B18 substrate
0,425	0,068	Dehydro-L-gulonate decarboxylase inhibitor
0,407	0,051	Thymidylate 5'-phosphatase inhibitor
0,362	0,006	GST T substrate
0,362	0,006	GST T1-1 substrate
0,357	0,001	Prostaglandin EP2 agonist
0,358	0,002	Interleukin 10 antagonist
0,360	0,006	Osteoarthritis treatment
0,393	0,038	Antiviral (Herpes)
0,445	0,091	Ovulation inhibitor
0,383	0,030	Clavaminate synthase inhibitor
0,360	0,007	Alpha-pinene-oxide decyclase inhibitor
0,436	0,084	Antiviral (Picornavirus)
0,386	0,035	CYP7 inhibitor
0,359	0,007	Ligase inhibitor
0,376	0,025	Myeloblastin inhibitor
0,368	0,018	2-Haloacid dehalogenase (configuration-inverting) inhibitor
0,460	0,112	Calcium channel (voltage-sensitive) activator
0,396	0,050	GABA aminotransferase inhibitor
0,408	0,065	CYP2D15 substrate
0,358	0,015	Maltose-transporting ATPase inhibitor
0,410	0,068	CYP2D16 substrate
0,412	0,072	Vasoprotector
0,379	0,039	2-Haloacid dehalogenase inhibitor
0,386	0,047	Creatininase inhibitor
0,377	0,038	Undecaprenyldiphospho-muramoylpentapeptide beta-N-acetylglucosaminyltransferase inhibitor
0,388	0,050	Malate oxidase inhibitor
0,418	0,081	CYP3A1 substrate
0,437	0,101	CYP2C12 substrate
0,430	0,096	NADPH-cytochrome-c2 reductase inhibitor
0,375	0,041	Dextranase inhibitor
0,364	0,031	Yeast ribonuclease inhibitor
0,369	0,035	DELTA14-sterol reductase inhibitor
0,357	0,024	Sclerosant
0,374	0,042	Mannose isomerase inhibitor
0,367	0,037	Fructan beta-fructosidase inhibitor

0,414	0,084	Leukopoiesis stimulant
0,367	0,038	Cyclomaltodextrinase inhibitor
0,410	0,082	Erythropoiesis stimulant
0,350	0,024	Styrene-oxide isomerase inhibitor
0,332	0,007	Antineoplastic, alkylator
0,382	0,058	Mucinaminylserine mucinaminidase inhibitor
0,348	0,025	Phosphoenolpyruvate mutase inhibitor
0,340	0,018	Undecaprenyl-diphosphatase inhibitor
0,393	0,070	Polyamine-transporting ATPase inhibitor
0,370	0,048	Nitrite reductase (NO-forming) inhibitor
0,424	0,103	Chlordecone reductase inhibitor
0,381	0,060	Glycerol-3-phosphate oxidase inhibitor
0,410	0,091	Antiseborrheic
0,371	0,054	CYP2D2 inhibitor
0,405	0,089	Neurotransmitter antagonist
0,345	0,030	D-alanine 2-hydroxymethyltransferase inhibitor
0,413	0,099	5 Hydroxytryptamine uptake stimulant
0,389	0,075	Beta-adrenergic receptor kinase inhibitor
0,389	0,075	G-protein-coupled receptor kinase inhibitor
0,368	0,054	Steroid N-acetylglucosaminyltransferase inhibitor
0,357	0,044	2-Hydroxymuconate-semialdehyde hydrolase inhibitor
0,381	0,068	Phenol O-methyltransferase inhibitor
0,378	0,066	Antimyopathies
0,398	0,089	(R)-6-hydroxynicotine oxidase inhibitor
0,352	0,043	Opioid kappa 3 receptor antagonist
0,361	0,053	Phosphopantothenoylcysteine decarboxylase inhibitor
0,387	0,080	Ecdysone 20-monooxygenase inhibitor
0,338	0,031	Hyaluronic acid agonist
0,339	0,034	GST M substrate
0,369	0,064	Tpr proteinase (Porphyromonas gingivalis) inhibitor
0,372	0,068	(S)-6-hydroxynicotine oxidase inhibitor
0,374	0,071	Adenomatous polyposis treatment
0,344	0,042	Glyoxylate oxidase inhibitor
0,338	0,037	Carminative
0,357	0,056	N-formylmethionyl-peptidase inhibitor
0,379	0,079	Linoleate diol synthase inhibitor
0,372	0,072	Membrane permeability enhancer

0,300	0,002	Prostaglandin D2 agonist
0,383	0,086	4-Nitrophenol 2-monooxygenase inhibitor
0,389	0,092	Histamine release stimulant
0,325	0,028	Licheninase inhibitor
0,382	0,086	Fibrolase inhibitor
0,297	0,004	Insulin secretagoues
0,352	0,059	Peptide-N4-(N-acetyl-beta-glucosaminyl)asparagine amidase inhibitor
0,371	0,078	Aspartate-phenylpyruvate transaminase inhibitor
0,379	0,086	Dimethylargininase inhibitor
0,389	0,097	Aminobutyraldehyde dehydrogenase inhibitor
0,397	0,105	Antineoplastic
0,318	0,026	Fumarate reductase (NADH) inhibitor
0,329	0,038	CYP2B10 substrate
0,324	0,035	Oryzin inhibitor
0,329	0,040	2,3,4,5-Tetrahydropyridine-2,6-dicarboxylate N-succinyltransferase inhibitor
0,365	0,078	Manganese peroxidase inhibitor
0,365	0,081	Pro-opiomelanocortin converting enzyme inhibitor
0,347	0,064	Phosphoinositide 5-phosphatase inhibitor
0,331	0,048	CYP2A2 substrate
0,374	0,091	CYP2A1 substrate
0,324	0,042	Quinoprotein glucose dehydrogenase inhibitor
0,327	0,044	DNA synthesis inhibitor
0,319	0,037	Chaperonin ATPase inhibitor
0,359	0,080	Caspase 8 stimulant
0,346	0,067	Prostaglandin-A1 DELTA-isomerase inhibitor
0,309	0,031	Galactolipase inhibitor
0,306	0,030	Bisphosphoglycerate mutase inhibitor
0,358	0,082	CYP4A11 substrate
0,337	0,062	Lysostaphin inhibitor
0,351	0,076	Histidine N-acetyltransferase inhibitor
0,336	0,062	tRNA-pseudouridine synthase I inhibitor
0,334	0,061	3-Phytase inhibitor
0,303	0,031	Vitamin-K-epoxide reductase (warfarin-insensitive) inhibitor
0,305	0,033	Aspergillopepsin II inhibitor
0,322	0,051	Hydroxylamine reductase (NADH) inhibitor
0,301	0,030	Antineoplastic (small cell lung cancer)
0,348	0,081	Hydroxylamine oxidase inhibitor

0,303	0,037	Alpha-N-acetylglucosaminidase inhibitor
0,272	0,005	Cytokine release inhibitor
0,307	0,042	Steroid synthesis inhibitor
0,350	0,086	Sphinganine kinase inhibitor
0,332	0,069	RELA expression inhibitor
0,288	0,025	3-Chloro-D-alanine dehydrochlorinase inhibitor
0,296	0,035	Alkylglycerophosphoethanolamine phosphodiesterase inhibitor
0,353	0,091	Rubredoxin-NAD+ reductase inhibitor
0,282	0,022	2-Dehydropantoate aldolase inhibitor
0,354	0,095	Pancreatic elastase inhibitor
0,327	0,068	Alcohol dehydrogenase (acceptor) inhibitor
0,320	0,061	CYP2A5 substrate
0,293	0,036	Polyneuridine-aldehyde esterase inhibitor
0,283	0,026	Hydroxysteroid dehydrogenase inhibitor
0,344	0,087	Centromere associated protein inhibitor
0,321	0,067	3-Cyanoalanine hydratase inhibitor
0,304	0,050	CYP2G1 substrate
0,293	0,040	Antihelmintic
0,378	0,126	Antinociceptive
0,301	0,049	CYP2C29 substrate
0,254	0,002	Prostaglandin E2 agonist
0,321	0,070	Glutamine-phenylpyruvate transaminase inhibitor
0,329	0,079	Leukopoiesis inhibitor
0,303	0,053	Transketolase inhibitor
0,291	0,041	N-Acyl-D-aspartate deacylase inhibitor
0,299	0,050	Linoleoyl-CoA desaturase inhibitor
0,330	0,080	Methylamine-glutamate N-methyltransferase inhibitor
0,291	0,043	Protein-Npi-phosphohistidine-sugar phosphotransferase inhibitor
0,374	0,126	Thromboxane B2 antagonist
0,304	0,056	Antihypercholesterolemic
0,358	0,111	2-Dehydropantoate 2-reductase inhibitor
0,326	0,079	CYP2F1 substrate
0,286	0,040	Poly(alpha-L-guluronate) lyase inhibitor
0,335	0,089	Cytochrome P450 stimulant
0,328	0,082	(R)-Pantolactone dehydrogenase (flavin) inhibitor
0,265	0,021	Galacturan 1,4-alpha-galacturonidase inhibitor
0,319	0,075	ADP-thymidine kinase inhibitor

0,335	0,092	Glucose oxidase inhibitor
0,286	0,042	Pseudouridylate synthase inhibitor
0,315	0,072	Phosphatidylinositol diacylglycerol-lyase inhibitor
0,261	0,019	Deoxyribonuclease I inhibitor
0,319	0,077	Chenodeoxycholoyltaurine hydrolase inhibitor
0,246	0,006	4-Carboxymethyl-4-methylbutenolide mutase inhibitor
0,258	0,018	Melanin inhibitor
0,268	0,028	Alpha-glucuronidase inhibitor
0,335	0,097	Endopeptidase So inhibitor
0,309	0,071	Chitosanase inhibitor
0,304	0,066	Cytostatic
0,298	0,061	Lysyl oxidase inhibitor
0,319	0,082	Fucosterol-epoxide lyase inhibitor
0,282	0,046	Horrilysin inhibitor
0,294	0,058	Antiparasitic
0,329	0,094	CYP2C9 inducer
0,247	0,012	Arachidonic acid antagonist
0,263	0,028	Tropinesterase inhibitor
0,291	0,057	Opheline kinase inhibitor
0,291	0,057	Taurocyamine kinase inhibitor
0,309	0,076	Adenylyl-sulfate reductase inhibitor
0,276	0,044	Dopamine release stimulant
0,304	0,071	N-acylmannosamine kinase inhibitor
0,278	0,046	Antiperistaltic
0,334	0,103	Gonadotropin antagonist
0,308	0,077	CYP3A7 substrate
0,334	0,103	Biotinidase inhibitor
0,294	0,063	Antineoplastic (pancreatic cancer)
0,289	0,059	NF-E2-related factor 2 stimulant
0,328	0,100	Malate dehydrogenase (acceptor) inhibitor
0,258	0,030	Rhizopuspepsin inhibitor
0,282	0,054	Adrenaline release stimulant
0,291	0,064	Long-chain-aldehyde dehydrogenase inhibitor
0,328	0,101	S-formylglutathione hydrolase inhibitor
0,247	0,020	Antihypotensive
0,282	0,055	Levanase inhibitor
0,299	0,075	D-lactaldehyde dehydrogenase inhibitor

0,288	0,063	Glycerol-3-phosphate dehydrogenase inhibitor
0,316	0,092	Sulfite dehydrogenase inhibitor
0,358	0,134	Octopamine antagonist
0,272	0,049	2-Oxoaldehyde dehydrogenase (NADP+) inhibitor
0,271	0,048	UGT2B28 substrate
0,298	0,075	Isopenicillin-N epimerase inhibitor
0,265	0,043	Anthranilate-CoA ligase inhibitor
0,295	0,073	Peptide alpha-N-acetyltransferase inhibitor
0,280	0,059	Bisphosphoglycerate phosphatase inhibitor
0,355	0,134	Fructose 5-dehydrogenase inhibitor
0,280	0,060	Dipeptidase E inhibitor
0,289	0,069	Glutamate-tRNA ligase inhibitor
0,235	0,015	Trehalose-phosphatase inhibitor
0,282	0,063	Endothelial growth factor antagonist
0,256	0,037	D-xylulose reductase inhibitor
0,268	0,050	2-Hydroxy-3-oxoadipate synthase inhibitor
0,250	0,033	1,4-Alpha-glucan branching enzyme inhibitor
0,262	0,045	Mannitol-1-phosphatase inhibitor
0,307	0,090	Plastoquinol-plastocyanin reductase inhibitor
0,289	0,072	Pyruvate decarboxylase inhibitor
0,278	0,062	Salicylate 1-monooxygenase inhibitor
0,245	0,029	BRAF expression inhibitor
0,309	0,095	MMP9 expression inhibitor
0,278	0,064	MAP kinase kinase 4 inhibitor
0,270	0,056	Benzaldehyde dehydrogenase (NADP+) inhibitor
0,242	0,028	GABA B receptor agonist
0,289	0,075	N-benzyloxycarbonylglycine hydrolase inhibitor
0,305	0,092	Peptidyl-dipeptidase Dcp inhibitor
0,263	0,050	Aldosterone antagonist
0,266	0,053	Flavin-containing monooxygenase inhibitor
0,297	0,084	Enteropeptidase inhibitor
0,230	0,017	Alpha-N-acetylgalactosaminidase inhibitor
0,275	0,062	Gluconolactonase inhibitor
0,258	0,046	N-acetyllactosaminide beta-1,3-N-acetylglucosaminyltransferase inhibitor
0,278	0,066	NADH kinase inhibitor
0,248	0,037	NAD+ synthase (glutamine-hydrolysing) inhibitor
0,262	0,052	Glutarate-semialdehyde dehydrogenase inhibitor

0,338	0,130	Pin1 inhibitor
0,223	0,016	(R)-limonene 6-monooxygenase inhibitor
0,313	0,106	Lipoprotein lipase inhibitor
0,222	0,015	Antiemphysemic
0,210	0,003	Lipase inhibitor
0,251	0,044	Dynein ATPase inhibitor
0,312	0,106	5 Hydroxytryptamine release stimulant
0,320	0,115	Nitrate reductase (cytochrome) inhibitor
0,251	0,046	Cellulose 1,4-beta-cellobiosidase inhibitor
0,245	0,041	Aureolysin inhibitor
0,286	0,081	Methylumbelliferyl-acetate deacetylase inhibitor
0,303	0,099	Cyanoalanine nitrilase inhibitor
0,233	0,030	Alkene monooxygenase inhibitor
0,252	0,051	Prunasin beta-glucosidase inhibitor
0,268	0,067	Thiamine pyridinylase inhibitor
0,265	0,065	Morphine 6-dehydrogenase inhibitor
0,257	0,057	N-hydroxy-2-acetamidofluorene reductase inhibitor
0,222	0,022	Riboflavin phosphotransferase inhibitor
0,291	0,091	Arylsulfate sulfotransferase inhibitor
0,232	0,032	Aldehyde dehydrogenase inhibitor
0,227	0,028	Ferredoxin-NADP+ reductase inhibitor
0,262	0,063	Transcription factor NF kappa A inhibitor
0,210	0,011	CYP3A5 inducer
0,241	0,042	Aryldialkylphosphatase inhibitor
0,264	0,066	Ethanolamine-phosphate cytidylyltransferase inhibitor
0,237	0,039	Inulinase inhibitor
0,284	0,086	P-benzoquinone reductase (NADPH) inhibitor
0,338	0,140	Oxygen scavenger
0,251	0,053	Alpha-1,6-mannosyl-glycoprotein 4-beta-N-acetylglucosaminyltransferase inhibitor
0,339	0,142	MAP kinase stimulant
0,375	0,180	Antineurotic
0,244	0,049	Polygalacturonase inhibitor
0,251	0,056	Pappalysin-1 inhibitor
0,258	0,062	Carboxypeptidase D inhibitor
0,275	0,079	Arylmalonate decarboxylase inhibitor
0,225	0,030	Glucan 1,6-alpha-glucosidase inhibitor
0,272	0,077	Opioid dependency treatment

0,274	0,080	Thiosulfate dehydrogenase inhibitor
0,245	0,051	3-Methylbutanal reductase inhibitor
0,235	0,042	Glucan 1,4-beta-glucosidase inhibitor
0,227	0,034	Glycerol-1-phosphatase inhibitor
0,235	0,042	Lactaldehyde reductase inhibitor
0,221	0,028	2,2-Dialkylglycine decarboxylase (pyruvate) inhibitor
0,262	0,070	S-alkylcysteine lyase inhibitor
0,220	0,028	Pancreatic endopeptidase E inhibitor
0,273	0,081	Aspergillus nuclease S1 inhibitor
0,236	0,045	Snapalysin inhibitor
0,244	0,053	Glycine dehydrogenase (decarboxylating) inhibitor
0,239	0,049	Cyclooxygenase substrate
0,213	0,024	Estradiol 17alpha-dehydrogenase inhibitor
0,318	0,129	RNA-directed RNA polymerase inhibitor
0,238	0,049	4-Chlorobenzoyl-CoA dehalogenase inhibitor
0,283	0,094	Chitinase inhibitor
0,298	0,110	Leukotriene-C4 synthase inhibitor
0,225	0,037	Aspartate-tRNA ligase inhibitor
0,243	0,056	GABA C receptor agonist
0,257	0,072	NOS2 expression inhibitor
0,262	0,077	Antibacterial
0,218	0,033	Allantoate deiminase inhibitor
0,222	0,037	Bile-salt sulfotransferase inhibitor
0,251	0,068	Cyclohexyl-isocyanide hydratase inhibitor
0,222	0,039	Cycloartenol synthase inhibitor
0,209	0,027	Carnitine dehydratase inhibitor
0,254	0,072	Thiamine-triphosphatase inhibitor
0,239	0,057	Crotonoyl-[acyl-carrier-protein] hydratase inhibitor
0,242	0,059	Ornithine cyclodeaminase inhibitor
0,268	0,087	4-Coumarate-CoA ligase inhibitor
0,287	0,107	Myc inhibitor
0,193	0,013	Succinate dehydrogenase inhibitor
0,244	0,064	N-acetyllactosamine synthase inhibitor
0,256	0,077	Arylesterase inhibitor
0,212	0,032	L-iduronidase inhibitor
0,238	0,059	N-(long-chain-acyl)ethanolamine deacylase inhibitor
0,216	0,038	Cyclamate sulfohydrolase inhibitor

0,251	0,072	Glutathione dehydrogenase (ascorbate) inhibitor
0,315	0,136	Nicotine dehydrogenase inhibitor
0,252	0,075	Coccolysin inhibitor
0,303	0,125	Apoptosis agonist
0,290	0,113	CYP3A4 inducer
0,209	0,033	Glycopeptide alpha-N-acetylgalactosaminidase inhibitor
0,209	0,033	Glycerone-phosphate O-acyltransferase inhibitor
0,227	0,050	Sedoheptulose-bisphosphatase inhibitor
0,181	0,004	Glycine receptor antagonist
0,251	0,075	Aspartate-ammonia ligase inhibitor
0,226	0,050	Guanosine-3',5'-bis(diphosphate) 3'-diphosphatase inhibitor
0,235	0,059	Ubiquitin thiolesterase inhibitor
0,197	0,022	Interferon antagonist
0,280	0,105	HMOX1 expression enhancer
0,225	0,050	IgA-specific metalloendopeptidase inhibitor
0,235	0,060	Procollagen N-endopeptidase inhibitor
0,299	0,125	Amine dehydrogenase inhibitor
0,282	0,108	CYP3A inducer
0,209	0,036	Retinyl-palmitate esterase inhibitor
0,210	0,037	Acetylenecarboxylate hydratase inhibitor
0,176	0,004	Anesthetic inhalation
0,270	0,099	Ferredoxin-NAD+ reductase inhibitor
0,270	0,099	Naphthalene 1,2-dioxygenase inhibitor
0,290	0,119	Antihelmintic (Nematodes)
0,190	0,020	Interferon gamma antagonist
0,223	0,052	2,3-Dihydroxyindole 2,3-dioxygenase inhibitor
0,252	0,081	Carbon-monoxide dehydrogenase inhibitor
0,267	0,097	Para amino benzoic acid antagonist
0,248	0,078	Cholestanetriol 26-monooxygenase inhibitor
0,270	0,101	Nitrite reductase [NAD(P)H] inhibitor
0,219	0,050	Tetrahydroxynaphthalene reductase inhibitor
0,171	0,003	Prostaglandin EP1 antagonist
0,247	0,079	Phosphatidylcholine-sterol O-acyltransferase inhibitor
0,199	0,033	Valine-tRNA ligase inhibitor
0,183	0,017	Demethylsterigmatocystin 6-O-methyltransferase inhibitor
0,216	0,050	Sterol 3-beta-glucosyltransferase inhibitor
0,256	0,090	Antiviral (Poxvirus)

0,210	0,044	Alkylator
0,284	0,118	NAD(P)+-arginine ADP-ribosyltransferase inhibitor
0,197	0,031	Protein-glucosylgalactosylhydroxylysine glucosidase inhibitor
0,266	0,101	4-Hydroxymandelate oxidase inhibitor
0,263	0,099	DNA-(apurinic or apyrimidinic site) lyase inhibitor
0,225	0,061	Rhodotorulapepsin inhibitor
0,193	0,028	Pectin lyase inhibitor
0,293	0,128	Polarisation stimulant
0,223	0,058	D-threo-aldose 1-dehydrogenase inhibitor
0,207	0,044	Nitric oxide scavenger
0,204	0,041	Phosphoenolpyruvate-protein phosphotransferase inhibitor
0,195	0,032	Glucuronolactone reductase inhibitor
0,232	0,070	Di-trans,poly-cis-decaprenylcistransferase inhibitor
0,240	0,079	Cell wall biosynthesis inhibitor
0,237	0,076	Lactose synthase inhibitor
0,318	0,157	2-Hydroxyquinoline 8-monooxygenase inhibitor
0,180	0,020	Isoamylase inhibitor
0,198	0,039	Antineoplastic (lymphocytic leukemia)
0,231	0,073	Dimethylmaleate hydratase inhibitor
0,244	0,086	Succinate-semialdehyde dehydrogenase [NAD(P)+] inhibitor
0,204	0,047	Antirickettsial
0,218	0,061	Tauropine dehydrogenase inhibitor
0,210	0,054	Leucine dehydrogenase inhibitor
0,243	0,087	Antiviral (CMV)
0,285	0,130	Glutathione thiolesterase inhibitor
0,188	0,033	Gaucher disease treatment
0,234	0,080	[acyl-carrier-protein] S-acetyltransferase inhibitor
0,197	0,043	Dolichyl-phosphatase inhibitor
0,255	0,102	Urethanase inhibitor
0,204	0,051	Farnesyltranstransferase inhibitor
0,194	0,042	Ganglioside galactosyltransferase inhibitor
0,192	0,039	Thioredoxin reductase inhibitor
0,207	0,055	Alpha-amylase inhibitor
0,290	0,138	Spermidine dehydrogenase inhibitor
0,171	0,019	Dry eye syndrome treatment
0,236	0,084	CYP2C3 substrate
0,222	0,072	Glycerol 2-dehydrogenase (NADP+) inhibitor

0,227	0,076	Nardilysin inhibitor
0,226	0,076	Gamma-D-Glutamyl-meso-diaminopimelate peptidase inhibitor
0,217	0,068	UGT2B17 substrate
0,276	0,127	CYP3A3 substrate
0,205	0,056	Serratia marcescens nuclease inhibitor
0,202	0,053	Protein synthesis inhibitor
0,198	0,049	Choline-sulfatase inhibitor
0,277	0,128	CYP2E1 inducer
0,197	0,049	Trans-pentaprenyltranstransferase inhibitor
0,182	0,034	Deoxyribose-phosphate aldolase inhibitor
0,212	0,064	Peptidyl-dipeptidase B inhibitor
0,190	0,042	Saccharolysin inhibitor
0,187	0,039	Protein synthesis stimulant
0,185	0,039	3-Ketovalidoxylamine C-N-lyase inhibitor
0,175	0,028	Choloylglycine hydrolase inhibitor
0,191	0,044	Mannan endo-1,6-alpha-mannosidase inhibitor
0,302	0,155	Antihypoxic
0,271	0,124	Bilirubin oxidase inhibitor
0,226	0,081	Meprin B inhibitor
0,170	0,024	Aconitate decarboxylase inhibitor
0,252	0,106	Tyrosine 3 hydroxylase inhibitor
0,252	0,107	Formate-dihydrofolate ligase inhibitor
0,244	0,099	Aldehyde dehydrogenase (pyrroloquinoline-quinone) inhibitor
0,206	0,061	(S)-3-amino-2-methylpropionate transaminase inhibitor
0,274	0,130	Gamma-guanidinobutyraldehyde dehydrogenase inhibitor
0,189	0,044	Testosterone 17beta-dehydrogenase inhibitor
0,161	0,016	Shab potassium channel blocker
0,195	0,050	Vascular adhesion protein 1 inhibitor
0,245	0,101	AR expression inhibitor
0,195	0,052	Granzyme A inhibitor
0,197	0,054	Gamma-butyrobetaine dioxygenase inhibitor
0,307	0,164	Preneoplastic conditions treatment
0,170	0,027	Pregnane X receptor agonist
0,186	0,043	Glutamate (mGluR5) agonist
0,226	0,084	DNA ligase (ATP) inhibitor
0,186	0,044	Polar-amino-acid-transporting ATPase inhibitor
0,182	0,041	Allantoinase inhibitor

0,146	0,004	Prostaglandin EP2 antagonist
0,168	0,028	Glyceraldehyde-3-phosphate dehydrogenase (phosphorylating) inhibitor
0,177	0,037	Xylose isomerase inhibitor
0,224	0,085	Glycine amidinotransferase inhibitor
0,247	0,108	Antifungal
0,254	0,115	Antiinflammatory, intestinal
0,214	0,075	CYP2A3 substrate
0,242	0,103	Leukotriene-B4 20-monooxygenase inhibitor
0,238	0,100	Phosphatidylserine decarboxylase inhibitor
0,192	0,054	ADP-ribosylarginine hydrolase inhibitor
0,150	0,012	Fatty-acyl-ethyl-ester synthase inhibitor
0,220	0,082	Proliferative diseases treatment
0,184	0,046	N-acetylneuraminate synthase inhibitor
0,194	0,056	N6-methyl-lysine oxidase inhibitor
0,246	0,108	Methylenetetrahydrofolate reductase (NADPH) inhibitor
0,198	0,061	2,4-Dichlorophenol 6-monooxygenase inhibitor
0,189	0,053	Laminaribiose phosphorylase inhibitor
0,172	0,036	Glucarate dehydratase inhibitor
0,231	0,095	DNA-3-methyladenine glycosylase I inhibitor
0,186	0,050	Glutamin-(asparagin-)ase inhibitor
0,302	0,166	Sulfur reductase inhibitor
0,146	0,011	Imidazoleglycerol-phosphate dehydratase inhibitor
0,175	0,040	Cholesterol oxidase inhibitor
0,144	0,010	Acetylcholine M3 receptor agonist
0,165	0,031	Trimethyllysine dioxygenase inhibitor
0,179	0,046	Alpha-N-arabinofuranosidase inhibitor
0,341	0,208	Mucomembranous protector
0,233	0,100	Hepatoprotectant
0,238	0,105	Gingipain K inhibitor
0,241	0,109	Cytochrome-b5 reductase inhibitor
0,237	0,104	Cis-1,2-dihydro-1,2-dihydroxynaphthalene dehydrogenase inhibitor
0,172	0,040	Asparagine-tRNA ligase inhibitor
0,183	0,051	Galactokinase inhibitor
0,260	0,128	Loop diuretic
0,287	0,155	Caspase 3 stimulant
0,174	0,043	N-(5-amino-5-carboxypentanoyl)-L-cysteinyl-D-valine synthase inhibitor
0,277	0,146	N-hydroxyarylamine O-acetyltransferase inhibitor

0,146	0,016	Mycodextranase inhibitor
0,192	0,061	UGT1A5 substrate
0,265	0,134	Mitochondrial processing peptidase inhibitor
0,193	0,063	Tentoxilysin inhibitor
0,205	0,075	Serine-pyruvate transaminase inhibitor
0,220	0,090	Prolyl aminopeptidase inhibitor
0,232	0,103	Indanol dehydrogenase inhibitor
0,161	0,031	4-Alpha-glucanotransferase inhibitor
0,191	0,061	Isopenicillin-N synthase inhibitor
0,162	0,033	(S)-carnitine 3-dehydrogenase inhibitor
0,243	0,114	4-Methoxybenzoate monooxygenase (O-demethylating) inhibitor
0,232	0,104	Acidifying agent non gastric
0,170	0,041	Acyl-lysine deacylase inhibitor
0,273	0,145	Menopausal disorders treatment
0,239	0,112	ICAM1 expression inhibitor
0,265	0,138	Retinoic acid metabolism inhibitor
0,203	0,077	Cyclopropane-fatty-acyl-phospholipid synthase inhibitor
0,200	0,073	Signal peptidase I inhibitor
0,316	0,190	Taurine dehydrogenase inhibitor
0,167	0,041	Serine 3-dehydrogenase inhibitor
0,141	0,016	Gentamicin 2''-nucleotidyltransferase inhibitor
0,191	0,066	CDP-4-dehydro-6-deoxyglucose reductase inhibitor
0,158	0,033	Creatinase inhibitor
0,193	0,068	Antihelmintic (Fasciola)
0,188	0,063	Glyoxylate reductase (NADP+) inhibitor
0,188	0,064	Sphinganine-1-phosphate aldolase inhibitor
0,224	0,099	CYP2B substrate
0,233	0,110	Sigma receptor agonist
0,151	0,028	Thermomycolin inhibitor
0,136	0,013	Alpha glucosidase inhibitor
0,190	0,067	3-Hydroxy-4-oxoquinoline 2,4-dioxygenase inhibitor
0,166	0,043	Selenocysteine lyase inhibitor
0,150	0,027	DELTA24-sterol reductase inhibitor
0,216	0,093	Uroporphyrinogen-III synthase inhibitor
0,129	0,007	Progesterone agonist
0,197	0,075	Antihematotoxic
0,168	0,047	Valine decarboxylase inhibitor

0,131	0,009	Osmotic diuretic
0,216	0,094	Antifibrinolytic
0,181	0,059	GST M1-1 substrate
0,189	0,067	Glutamate 5-kinase inhibitor
0,211	0,091	Peroxidase substrate
0,261	0,141	Cholesterol antagonist
0,183	0,063	N-acetylneuraminate 4-O-acetyltransferase inhibitor
0,148	0,028	Homocysteine desulfhydrase inhibitor
0,141	0,021	Phosphatidylinositol 3-kinase stimulant
0,175	0,055	Galactose oxidase inhibitor
0,246	0,126	Alkane 1-monooxygenase inhibitor
0,147	0,027	Secretase alpha stimulant
0,147	0,027	Secretase stimulant
0,215	0,096	Oxytocic
0,167	0,048	Pectate lyase inhibitor
0,136	0,018	High-mannose-oligosaccharide beta-1,4-N-acetylglucosaminyltransferase inhibitor
0,151	0,033	Choline dehydrogenase inhibitor
0,206	0,088	Bothrolysin inhibitor
0,162	0,043	Methionine decarboxylase inhibitor
0,175	0,057	3-Carboxyethylcatechol 2,3-dioxygenase inhibitor
0,164	0,046	CDP-diacylglycerol-inositol 3-phosphatidyltransferase inhibitor
0,161	0,043	Glutamate-1-semialdehyde 2,1-aminomutase inhibitor
0,204	0,086	Aminomuconate-semialdehyde dehydrogenase inhibitor
0,144	0,026	Beta-amylase inhibitor
0,159	0,041	Carboxymethylenebutenolidase inhibitor
0,200	0,084	Retinal dehydrogenase inhibitor
0,172	0,056	Methyltransferase substrate
0,146	0,030	2-Dehydropantolactone reductase (A-specific) inhibitor
0,127	0,012	Alpha,alpha-trehalose phosphorylase inhibitor
0,190	0,075	CYP4A2 substrate
0,221	0,106	Methane monooxygenase inhibitor
0,183	0,068	Alkylglycerone-phosphate synthase inhibitor
0,218	0,104	UGT2B4 substrate
0,237	0,123	Analgesic stimulant
0,194	0,080	2,6-Dihydroxypyridine 3-monooxygenase inhibitor
0,146	0,033	Nicotinamide-nucleotide adenylyltransferase inhibitor
0,158	0,046	Dihydrouracil dehydrogenase (NAD+) inhibitor

0,251	0,138	Antiviral (Adenovirus)
0,176	0,063	Vomilenine glucosyltransferase inhibitor
0,148	0,036	D-arabinonolactone oxidase inhibitor
0,133	0,020	Alternansucrase inhibitor
0,191	0,079	Phosphatidylglycerophosphatase inhibitor
0,180	0,068	Mitochondrial intermediate peptidase inhibitor
0,176	0,065	tRNA nucleotidyltransferase inhibitor
0,178	0,067	Lipoxygenase substrate
0,142	0,031	Glutamine-pyruvate transaminase inhibitor
0,143	0,032	Valine dehydrogenase (NADP+) inhibitor
0,177	0,067	Lactate 2-monooxygenase inhibitor
0,182	0,071	CYP2D1 substrate
0,167	0,057	Geranylgeranyl-diphosphate geranylgeranyltransferase inhibitor
0,218	0,108	Prostaglandin-E2 9-reductase inhibitor
0,221	0,112	Cathepsin T inhibitor
0,152	0,043	Endo-1,4-beta-xylanase inhibitor
0,243	0,134	4-Hydroxyproline epimerase inhibitor
0,178	0,069	Carnosine synthase inhibitor
0,175	0,066	UGT2B9 substrate
0,255	0,146	Membrane integrity antagonist
0,187	0,078	UGT2B10 substrate
0,165	0,057	SMN2 expression enhancer
0,131	0,023	Homoserine O-succinyltransferase inhibitor
0,176	0,067	Brachyurin inhibitor
0,134	0,025	Tagaturonate reductase inhibitor
0,134	0,025	Fructuronate reductase inhibitor
0,130	0,022	Amino-acid racemase inhibitor
0,123	0,015	Heme oxygenase inhibitor
0,187	0,080	2-Enoate reductase inhibitor
0,117	0,010	Inositol 1,4,5-triphosphate receptor antagonist
0,133	0,026	Hair growth stimulant
0,135	0,029	Rhodopsin kinase inhibitor
0,149	0,043	Phosphonoacetate hydrolase inhibitor
0,146	0,039	1-Aminocyclopropane-1-carboxylate deaminase inhibitor
0,201	0,094	Methanol dehydrogenase inhibitor
0,140	0,033	Glucan 1,3-beta-glucosidase inhibitor
0,224	0,118	4-Nitrophenylphosphatase inhibitor

0,261	0,155	Superoxide dismutase inhibitor
0,200	0,094	Carnitinamidase inhibitor
0,180	0,074	N-acetyl-gamma-glutamyl-phosphate reductase inhibitor
0,221	0,115	ATP phosphoribosyltransferase inhibitor
0,160	0,055	3-Oxoadipate enol-lactonase inhibitor
0,135	0,030	Deoxycytidine deaminase inhibitor
0,140	0,036	Malonate-semialdehyde dehydrogenase inhibitor
0,198	0,094	Hyponitrite reductase inhibitor
0,223	0,119	CYP2C6 substrate
0,148	0,044	Glycosylphosphatidylinositol diacylglycerol-lyase inhibitor
0,123	0,019	Ca2+-transporting ATPase inhibitor
0,217	0,113	Cyclic AMP modulator
0,141	0,037	Deoxyribonuclease X inhibitor
0,180	0,076	Rhamnulose-1-phosphate aldolase inhibitor
0,186	0,083	Phenylalanine(histidine) transaminase inhibitor
0,133	0,030	Isomaltulose synthase inhibitor
0,224	0,121	Monoamine uptake inhibitor
0,177	0,074	Dolichol kinase inhibitor
0,130	0,027	Ribose-5-phosphate-ammonia ligase inhibitor
0,170	0,067	3'-Nucleotidase inhibitor
0,200	0,097	Bontoxilysin inhibitor
0,181	0,079	CYP4F2 substrate
0,177	0,075	2,4-Diaminopentanoate dehydrogenase inhibitor
0,177	0,075	3-Hydroxybutyryl-CoA dehydrogenase inhibitor
0,177	0,075	Lysine 6-dehydrogenase inhibitor
0,168	0,066	Allantoin racemase inhibitor
0,163	0,061	Glucosyl transferase inhibitor
0,148	0,046	Beta-mannosidase inhibitor
0,129	0,027	Arabinose isomerase inhibitor
0,157	0,056	Chitin synthase inhibitor
0,229	0,128	RNA synthesis inhibitor
0,115	0,014	Arabinogalactan endo-1,4-beta-galactosidase inhibitor
0,150	0,049	2-Oxoisovalerate dehydrogenase (acylating) inhibitor
0,229	0,128	Venom exonuclease inhibitor
0,185	0,085	Gallate decarboxylase inhibitor
0,174	0,074	Aldehyde dehydrogenase 2 substrate
0,139	0,040	Fructose-2,6-bisphosphate 6-phosphatase inhibitor

0,149	0,049	Antineoplastic antimetabolite
0,166	0,067	Calcium-sensing receptor agonist
0,155	0,056	Hepatocyte nuclear factor antagonist
0,155	0,056	Hepatocyte nuclear factor 4 alpha antagonist
0,111	0,013	D-lactate-2-sulfatase inhibitor
0,260	0,161	CYP19A1 expression inhibitor
0,145	0,047	Urolithiasis treatment
0,123	0,026	2-Methyleneglutarate mutase inhibitor
0,146	0,050	Agmatinase inhibitor
0,190	0,095	Alanine-tRNA ligase inhibitor
0,213	0,117	X-methyl-His dipeptidase inhibitor
0,160	0,065	Homoserine dehydrogenase inhibitor
0,111	0,015	Alpha,alpha-phosphotrehalase inhibitor
0,215	0,120	Myosin ATPase inhibitor
0,147	0,052	D-lactate dehydrogenase inhibitor
0,136	0,040	Cytochrome-c3 hydrogenase inhibitor
0,142	0,047	Lactaldehyde reductase (NADPH) inhibitor
0,152	0,057	Diisopropyl-fluorophosphatase inhibitor
0,138	0,044	Omega-amidase inhibitor
0,142	0,048	CYP19 substrate
0,158	0,064	Cerebroside-sulfatase inhibitor
0,132	0,039	Sorbose dehydrogenase inhibitor
0,101	0,008	Glutamate (mGluR3) agonist
0,193	0,100	Nucleoside oxidase (H2O2-forming) inhibitor
0,175	0,082	6-Carboxyhexanoate-CoA ligase inhibitor
0,175	0,082	Triacetate-lactonase inhibitor
0,175	0,082	Homoaconitate hydratase inhibitor
0,175	0,082	Biotin-CoA ligase inhibitor
0,158	0,066	Lipotropic
0,177	0,085	Camphor 1,2-monooxygenase inhibitor
0,132	0,040	Gluconate dehydratase inhibitor
0,165	0,073	Pyruvate dehydrogenase (cytochrome) inhibitor
0,165	0,073	Trans-2-enoyl-CoA reductase (NAD+) inhibitor
0,169	0,077	Antimycoplasmal
0,176	0,084	Metallocarboxypeptidase D inhibitor
0,158	0,067	Gly-X carboxypeptidase inhibitor
0,127	0,036	1,5-Anhydro-D-fructose reductase inhibitor

0,117	0,027	2-Methylcitrate dehydratase inhibitor
0,175	0,084	Guanidinoacetate kinase inhibitor
0,194	0,103	Alcohol dehydrogenase [NAD(P)+] inhibitor
0,129	0,039	Glycerophosphocholine cholinephosphodiesterase inhibitor
0,284	0,194	Mucositis treatment
0,115	0,026	Inositol-3-phosphate synthase inhibitor
0,170	0,081	Inorganic diphosphatase inhibitor
0,150	0,061	Iron-cytochrome-c reductase inhibitor
0,151	0,062	Cellulase inhibitor
0,219	0,130	NADPH-ferrihemoprotein reductase inhibitor
0,112	0,024	11-Cis-retinyl-palmitate hydrolase inhibitor
0,119	0,030	Phosphatidylglycerol-membrane-oligosaccharide glycerophosphotransferase inhibitor
0,129	0,040	Aerobactin synthase inhibitor
0,137	0,049	2-Aminoadipate transaminase inhibitor
0,127	0,039	2-Aminoethylphosphonate-pyruvate transaminase inhibitor
0,139	0,051	UDP-N-acetylglucosamine-dolichyl-phosphate N-acetylglucosaminephosphotransferase inhibitor
0,096	0,008	Lipocortins synthesis agonist
0,151	0,063	Phosphoinositide phospholipase C inhibitor
0,138	0,051	Fructose-2,6-bisphosphate 2-phosphatase inhibitor
0,121	0,034	Ketohexokinase inhibitor
0,125	0,038	Beta-D-fucosidase inhibitor
0,177	0,091	Nicotinic alpha4beta2 receptor antagonist
0,130	0,044	Poly(3-hydroxybutyrate) depolymerase inhibitor
0,117	0,031	Beta-N-acetylgalactosaminidase inhibitor
0,189	0,103	Acyl-CoA oxidase inhibitor
0,117	0,031	CYP4F substrate
0,120	0,035	Proline dehydrogenase inhibitor
0,115	0,029	L-fuconate dehydratase inhibitor
0,133	0,047	Diacylglycerol kinase inhibitor
0,144	0,059	Allophanate hydrolase inhibitor
0,132	0,047	Lysozyme inhibitor
0,143	0,058	Haloacetate dehalogenase inhibitor
0,143	0,058	Prepilin peptidase inhibitor
0,152	0,067	UGT2B18 substrate
0,151	0,066	Alpha-Methylacyl-CoA racemase inhibitor
0,107	0,022	Alpha,alpha-trehalose phosphorylase (configuration-retaining) inhibitor

0,154	0,070	3-Aminobutyryl-CoA ammonia-lyase inhibitor
0,116	0,032	Ribonuclease U2 inhibitor
0,121	0,037	Bis(5'-adenosyl)-triphosphatase inhibitor
0,148	0,065	Protein-tyrosine sulfotransferase inhibitor
0,120	0,037	2-Hydroxy-3-oxopropionate reductase inhibitor
0,144	0,061	Glutathione S-transferase substrate
0,101	0,018	Chitin deacetylase inhibitor
0,135	0,053	NAD+ kinase inhibitor
0,147	0,065	CYP4B substrate
0,155	0,074	Penicillopepsin inhibitor
0,095	0,013	Prostaglandin agonist
0,135	0,054	Poly(ADP-ribose) glycohydrolase inhibitor
0,163	0,082	6-Pyruvoyltetrahydropterin synthase inhibitor
0,159	0,077	Polynucleotide 5'-hydroxy-kinase inhibitor
0,120	0,038	Dichloromuconate cycloisomerase inhibitor
0,108	0,026	Alpha-L-fucosidase inhibitor
0,140	0,059	Excitatory amino acid transporter EAAC1 inhibitor
0,137	0,055	Mevalonate kinase inhibitor
0,126	0,045	5 Hydroxytryptamine 4A antagonist
0,156	0,076	Phenylacetate-CoA ligase inhibitor
0,116	0,035	3-Oxosteroid 1-dehydrogenase inhibitor
0,149	0,068	Aldehyde ferredoxin oxidoreductase inhibitor
0,109	0,029	Alpha-L-rhamnosidase inhibitor
0,181	0,101	Sulfite reductase inhibitor
0,166	0,086	Penicillin amidase inhibitor
0,156	0,075	Adenylate cyclase stimulant
0,160	0,079	Antidiabetic (type 2)
0,163	0,083	4-Hydroxyglutamate transaminase inhibitor
0,165	0,084	Picornain 3C inhibitor
0,173	0,093	Urate-ribonucleotide phosphorylase inhibitor
0,142	0,062	Galactose 1-dehydrogenase (NADP+) inhibitor
0,142	0,062	Beta-alanine-pyruvate transaminase inhibitor
0,134	0,054	Diacylglycerol cholinephosphotransferase inhibitor
0,145	0,065	Antibacterial, ophthalmic
0,154	0,074	2-Acylglycerol O-acyltransferase inhibitor
0,165	0,085	Microtubule formation inhibitor
0,090	0,010	Aldehyde dehydrogenase 1A2 inhibitor

0,200	0,121	Glutaminyl-peptide cyclotransferase inhibitor
0,095	0,016	NMDA receptor polyamine site agonist
0,136	0,057	ATP adenylyltransferase inhibitor
0,104	0,025	2-Dehydro-3-deoxy-phosphogluconate aldolase inhibitor
0,088	0,010	Elastase 1 inhibitor
0,270	0,191	Oxidoreductase inhibitor
0,141	0,062	8-Amino-7-oxononanoate synthase inhibitor
0,136	0,057	CYP26A substrate
0,114	0,035	Gluconokinase inhibitor
0,141	0,062	GABA A inverse agonist
0,141	0,062	GABA inverse agonist
0,130	0,052	NADH dehydrogenase inhibitor
0,115	0,037	Cell wall synthesis inhibitor
0,142	0,064	Thiamine-phosphate kinase inhibitor
0,103	0,025	(S)-2-Methylmalate dehydratase inhibitor
0,150	0,072	Oligopeptidase B inhibitor
0,122	0,044	NMDA receptor polyamine site antagonist
0,161	0,083	Creatinine deaminase inhibitor
0,157	0,079	Allyl-alcohol dehydrogenase inhibitor
0,126	0,048	Heparan-alpha-glucosaminide N-acetyltransferase inhibitor
0,127	0,050	Guanidinodeoxy-scyllo-inositol-4-phosphatase inhibitor
0,138	0,061	Biotin carboxylase inhibitor
0,190	0,112	Aromatic-hydroxylamine O-acetyltransferase inhibitor
0,141	0,063	Aminocarboxymuconate-semialdehyde decarboxylase inhibitor
0,178	0,101	Dihydroxy-acid dehydratase inhibitor
0,205	0,128	CYP2C10 substrate
0,164	0,087	Acetylornithine deacetylase inhibitor
0,125	0,049	Galactoside 2-alpha-L-fucosyltransferase inhibitor
0,140	0,065	Gingipain R inhibitor
0,112	0,037	2-Dehydro-3-deoxy-L-arabinonate dehydratase inhibitor
0,186	0,111	Histidinol dehydrogenase inhibitor
0,181	0,106	Opine dehydrogenase inhibitor
0,164	0,090	Glutathione peroxidase inhibitor
0,115	0,041	Glyceraldehyde-3-phosphate dehydrogenase (NADP+) inhibitor
0,203	0,129	Gamma-glutamyltransferase inhibitor
0,137	0,063	CYP4B1 substrate
0,105	0,031	Xylan 1,4-beta-xylosidase inhibitor

0,188	0,114	O-aminophenol oxidase inhibitor
0,155	0,082	Aspartoacylase inhibitor
0,134	0,061	(S)-2-hydroxy-acid oxidase inhibitor
0,155	0,083	4-Hydroxybenzoate nonaprenyltransferase inhibitor
0,123	0,050	Pantothenase inhibitor
0,140	0,067	Phenylpyruvate decarboxylase inhibitor
0,204	0,131	Chloride channel activator
0,186	0,114	Ferredoxin hydrogenase inhibitor
0,308	0,236	Antiviral (Rhinovirus)
0,107	0,035	Galactosylgalactosylglucosylceramidase inhibitor
0,114	0,042	Globoside alpha-N-acetylgalactosaminyltransferase inhibitor
0,115	0,043	Lysine decarboxylase inhibitor
0,109	0,037	3-Isopropylmalate dehydrogenase inhibitor
0,096	0,024	CYP4F12 substrate
0,121	0,050	Chondroitin 6-sulfotransferase inhibitor
0,134	0,062	O-acetylhomoserine aminocarboxypropyltransferase inhibitor
0,147	0,076	Aldehyde dehydrogenase (NADP+) inhibitor
0,170	0,099	Indoleacetaldoxime dehydratase inhibitor
0,131	0,060	Isovaleryl-CoA dehydrogenase inhibitor
0,075	0,005	Prostaglandin EP4 antagonist
0,074	0,003	Beta glucosidase inhibitor
0,151	0,081	Pyridoxine 4-oxidase inhibitor
0,160	0,090	Antineoplastic (renal cancer)
0,168	0,099	Thermitase inhibitor
0,141	0,071	2-Oxoglutarate decarboxylase inhibitor
0,120	0,051	Leucine transaminase inhibitor
0,117	0,048	Adenylylsulphatase inhibitor
0,202	0,133	Antiparkinsonian, rigidity relieving
0,132	0,063	Glucan endo-1,3-alpha-glucosidase inhibitor
0,128	0,059	Myocardial infarction treatment
0,122	0,052	UGT2B11 substrate
0,160	0,091	Glycine C-acetyltransferase inhibitor
0,117	0,048	D-glutamate oxidase inhibitor
0,102	0,034	Homospermidine synthase inhibitor
0,097	0,028	Alpha-1,6-mannosyl-glycoprotein 6-beta-N-acetylglucosaminyltransferase inhibitor
0,115	0,046	Phosphofructokinase-1 inhibitor
0,129	0,061	NAD(P)+ transhydrogenase (B-specific) inhibitor

0,128	0,060	Neuropsin inhibitor
0,160	0,092	Stromelysin 2 inhibitor
0,171	0,104	Sodium channel blocker class Ib
0,158	0,091	Hydroxymethylglutaryl-CoA lyase inhibitor
0,203	0,135	CYP2C11 substrate
0,125	0,058	CYP19 inhibitor
0,160	0,092	Arylacetonitrilase inhibitor
0,072	0,005	Antimitotic, Podophyllotoxin-like
0,124	0,058	L-lysine 6-transaminase inhibitor
0,087	0,020	3-Alpha-hydroxysteroid dehydrogenase (B-specific) inhibitor
0,219	0,153	Aldehyde oxidase inhibitor
0,194	0,128	CYP2B2 substrate
0,128	0,062	Iduronate-2-sulfatase inhibitor
0,124	0,058	Glycoprotein 3-alpha-L-fucosyltransferase inhibitor
0,179	0,114	D-amino-acid dehydrogenase inhibitor
0,126	0,060	D-2-hydroxy-acid dehydrogenase inhibitor
0,143	0,078	Restless leg syndrome treatment
0,183	0,118	Renal tissue kallikrein inhibitor
0,179	0,114	Indolepyruvate C-methyltransferase inhibitor
0,111	0,047	Leucine-tRNA ligase inhibitor
0,147	0,082	Aminopeptidase Y inhibitor
0,127	0,063	N4-(beta-N-acetylglucosaminyl)-L-asparaginase inhibitor
0,104	0,039	Phosphoenolpyruvate carboxykinase (diphosphate) inhibitor
0,203	0,139	Calcium channel activator
0,205	0,141	4-Hydroxyphenylacetate 3-monooxygenase inhibitor
0,106	0,042	Monocarboxylic acid transporter 1 inhibitor
0,106	0,042	Monocarboxylic acid transporter inhibitor
0,116	0,052	DNA nucleotidylexotransferase inhibitor
0,083	0,020	ATP diphosphatase inhibitor
0,146	0,083	Plasmanylethanolamine desaturase inhibitor
0,117	0,053	Interleukin 2 antagonist
0,147	0,084	Tryptophan transaminase inhibitor
0,070	0,007	CYP4F8 substrate
0,104	0,042	N-acetylneuraminate lyase inhibitor
0,137	0,074	Antineoplastic (gastric cancer)
0,077	0,014	Sucrose-phosphate phosphatase inhibitor
0,105	0,042	Cysteine transaminase inhibitor

0,172	0,110	Ribonucleoside triphosphate reductase inhibitor
0,091	0,028	CYP2A13 substrate
0,120	0,058	Ribonuclease inhibitor
0,115	0,053	Aspartate-ammonia ligase (ADP-forming) inhibitor
0,129	0,068	Pantoate 4-dehydrogenase inhibitor
0,152	0,091	N-methyl-2-oxoglutaramate hydrolase inhibitor
0,120	0,059	Anabolic
0,120	0,059	L-galactonolactone oxidase inhibitor
0,142	0,081	Ferrochelatase inhibitor
0,093	0,032	Amylo-alpha-1,6-glucosidase inhibitor
0,103	0,043	Galactosylceramide sulfotransferase inhibitor
0,122	0,061	D-lactate dehydrogenase (cytochrome) inhibitor
0,295	0,235	CYP2C8 inhibitor
0,163	0,103	Antinaupathic
0,088	0,028	Peptidoglycan beta-N-acetylmuramidase inhibitor
0,185	0,126	Tankyrase inhibitor
0,098	0,039	Malate dehydrogenase (NADP+) inhibitor
0,084	0,024	Alpha-mannosidase inhibitor
0,125	0,066	Contraceptive
0,119	0,060	CYP3C substrate
0,090	0,031	4a-Hydroxytetrahydrobiopterin dehydratase inhibitor
0,096	0,037	Aspartate-semialdehyde dehydrogenase inhibitor
0,140	0,081	Peptidylamidoglycolate lyase inhibitor
0,125	0,066	Carnitine 3-dehydrogenase inhibitor
0,148	0,089	Cytochrome-b5 reductase substrate
0,101	0,043	Retinol O-fatty-acyltransferase inhibitor
0,116	0,057	Alanine transaminase inhibitor
0,101	0,042	Cellobiose phosphorylase inhibitor
0,175	0,117	Glutaminase inhibitor
0,081	0,022	1-Alkyl-2-acetylglycerol O-acyltransferase inhibitor
0,189	0,131	Acetylserotonin O-methyltransferase inhibitor
0,086	0,029	Aldose 1-epimerase inhibitor
0,136	0,079	HIV-2 reverse transcriptase inhibitor
0,089	0,032	Steroid sulfotransferase inhibitor
0,118	0,061	Aldehyde dehydrogenase [NAD(P)+] inhibitor
0,112	0,055	Aspartate transaminase inhibitor
0,147	0,090	Benzoylformate decarboxylase inhibitor

0,097	0,040	Ribulose-bisphosphate carboxylase inhibitor
0,116	0,059	Cholate-CoA ligase inhibitor
0,092	0,035	3-Oxoacyl-[acyl-carrier-protein] synthase inhibitor
0,293	0,236	Acute neurologic disorders treatment
0,118	0,062	Aspartate kinase inhibitor
0,115	0,059	Pyrimidine-deoxynucleoside 2'-dioxygenase inhibitor
0,103	0,046	UDP-N-acetylglucosamine diphosphorylase inhibitor
0,102	0,046	5-Oxoprolinase (ATP-hydrolysing) inhibitor
0,155	0,099	3-Demethylubiquinone-9 3-O-methyltransferase inhibitor
0,101	0,045	3-Deoxy-8-phosphooctulonate synthase inhibitor
0,146	0,090	Acetate kinase inhibitor
0,276	0,220	Fibrinolytic
0,110	0,054	UDP-glucose 4-epimerase inhibitor
0,086	0,031	Hydroperoxide dehydratase inhibitor
0,130	0,075	Ethanolaminephosphotransferase inhibitor
0,088	0,033	Aspartate 1-decarboxylase inhibitor
0,145	0,090	Potassium channel intermediate-conductance Ca-activated activator
0,145	0,090	K(Ca) 3.1 channel activator
0,137	0,083	Heat shock protein agonist
0,096	0,042	L-lysine oxidase inhibitor
0,071	0,017	Biotin-[methylmalonyl-CoA-carboxytransferase] ligase inhibitor
0,095	0,041	Necroptosis inhibitor
0,121	0,067	Sulfate adenylyltransferase (ADP) inhibitor
0,087	0,033	Lactate-malate transhydrogenase inhibitor
0,105	0,051	Inositol-polyphosphate 5-phosphatase inhibitor
0,125	0,071	Cathepsin H inhibitor
0,086	0,033	3-Dehydroquinate synthase inhibitor
0,123	0,070	Adenylate cyclase inhibitor
0,090	0,037	Polyamine biosynthesis inhibitor
0,086	0,033	mRNA (guanine-N7-)-methyltransferase inhibitor
0,122	0,069	Glycogen (starch) synthase inhibitor
0,073	0,021	Glucan 1,4-alpha-glucosidase inhibitor
0,208	0,156	3C-like protease (Human coronavirus) inhibitor
0,106	0,054	Tyrosine-protein kinase TYRO 10 inhibitor
0,054	0,002	Prostaglandin EP12 antagonist
0,094	0,042	(R)-aminopropanol dehydrogenase inhibitor
0,090	0,039	Epoxide hydrolase substrate

0,223	0,172	Nicotinate dehydrogenase inhibitor
0,088	0,038	Glutaminyl-tRNA synthase (glutamine-hydrolysing) inhibitor
0,133	0,082	Quinoline-4-carboxylate 2-oxidoreductase inhibitor
0,085	0,035	CYP2C2 substrate
0,070	0,020	Amylosucrase inhibitor
0,201	0,151	Choline-phosphate cytidylyltransferase inhibitor
0,116	0,067	Cysteine desulfurase inhibitor
0,106	0,056	tRNA (cytosine-5-)-methyltransferase inhibitor
0,143	0,094	Hydroxymethylbilane synthase inhibitor
0,224	0,174	Antithrombotic
0,207	0,158	Farnesoid X receptor antagonist
0,074	0,024	CYP24 inhibitor
0,100	0,050	D-proline reductase (dithiol) inhibitor
0,174	0,124	Thiol oxidase inhibitor
0,068	0,019	Prostaglandin-I synthase inhibitor
0,108	0,060	Allantoicase inhibitor
0,112	0,064	Glycerol-3-phosphate O-acyltransferase inhibitor
0,075	0,026	Corticotropin releasing factor antagonist
0,094	0,045	Phenylalanine-tRNA ligase inhibitor
0,096	0,047	Squalene-hopene cyclase inhibitor
0,266	0,217	Intermittent claudication treatment
0,091	0,043	2-Methylcitrate synthase inhibitor
0,109	0,061	DNA-3-methyladenine glycosylase II inhibitor
0,098	0,050	Deoxyribonuclease (pyrimidine dimer) inhibitor
0,127	0,079	Sulfur dioxygenase inhibitor
0,058	0,010	Sucrose phosphorylase inhibitor
0,102	0,054	Mannonate dehydratase inhibitor
0,151	0,103	Threonine aldolase inhibitor
0,095	0,047	Saccharopine dehydrogenase (NAD+, L-glutamate-forming) inhibitor
0,070	0,022	UGT2B19 substrate
0,070	0,022	UGT2B30 substrate
0,070	0,022	UGT2B4E substrate
0,070	0,022	UGT2B4D substrate
0,070	0,022	UGT2B23 substrate
0,134	0,086	(R,R)-butanediol dehydrogenase inhibitor
0,088	0,040	Estrogen agonist
0,092	0,045	[hydroxymethylglutaryl-CoA reductase (NADPH)] kinase inhibitor

0,208	0,160	FMO1 substrate
0,084	0,037	Glycerol kinase inhibitor
0,078	0,032	[acetyl-CoA carboxylase] kinase inhibitor
0,070	0,023	Glucan 1,3-alpha-glucosidase inhibitor
0,052	0,005	Glucocorticoid agonist
0,191	0,144	Trimethylamine dehydrogenase inhibitor
0,171	0,124	Pitrilysin inhibitor
0,288	0,242	Diabetic neuropathy treatment
0,135	0,089	N-Acyl-D-amino-acid deacylase inhibitor
0,185	0,139	L-threonine 3-dehydrogenase inhibitor
0,084	0,038	Beta-glucosidase inhibitor
0,115	0,069	Glutamate release inhibitor
0,137	0,091	3,4-Dihydroxy-9,10-secoandrosta-1,3,5(10)-triene-9,17-dione 4,5-dioxygenase inhibitor
0,084	0,038	4-Hydroxy-4-methyl-2-oxoglutarate aldolase inhibitor
0,064	0,018	D(-)-tartrate dehydratase inhibitor
0,086	0,041	Glycerophosphocholine phosphodiesterase inhibitor
0,126	0,081	Homogentisate 1,2-dioxygenase inhibitor
0,227	0,182	Beta glucuronidase inhibitor
0,082	0,037	3(or 17)alpha-hydroxysteroid dehydrogenase inhibitor
0,089	0,044	Deoxycytidine kinase inhibitor
0,080	0,035	Lactaldehyde dehydrogenase inhibitor
0,238	0,193	CYP3A5 substrate
0,211	0,166	Antiprotozoal (Coccidial)
0,143	0,099	Tyrosine kinase stimulant
0,072	0,029	Acidifying agent gastric
0,080	0,037	ACK1 kinase inhibitor
0,127	0,084	Cysteine synthase inhibitor
0,137	0,094	Pyruvate carboxylase inhibitor
0,136	0,093	(S)-3-hydroxyacid ester dehydrogenase inhibitor
0,150	0,107	Atrolysin A inhibitor
0,129	0,086	Candidapepsin inhibitor
0,090	0,047	2-Oxoaldehyde dehydrogenase (NAD+) inhibitor
0,084	0,041	ATP deaminase inhibitor
0,092	0,049	Scyllo-inosamine-4-phosphate amidinotransferase inhibitor
0,102	0,060	Dihydrouracil oxidase inhibitor
0,110	0,068	Glucose 1-dehydrogenase inhibitor
0,101	0,060	Triose-phosphate isomerase inhibitor

0,085	0,044	Cyanate hydratase inhibitor
0,088	0,047	D-alanine transaminase inhibitor
0,180	0,138	Endopeptidase La inhibitor
0,067	0,025	3'(2'),5'-Bisphosphate nucleotidase inhibitor
0,151	0,110	Cyclopentanone monooxygenase inhibitor
0,103	0,062	Phosphatidate cytidylyltransferase inhibitor
0,124	0,083	Endoglycosylceramidase inhibitor
0,073	0,032	3-Methyl-2-oxobutanoate dehydrogenase (lipoamide) inhibitor
0,105	0,064	Glutamate N-acetyltransferase inhibitor
0,158	0,117	Gastrointestinal motility stimulant
0,093	0,053	UDP-N-acetylglucosamine 2-epimerase inhibitor
0,106	0,065	Site-specific DNA-methyltransferase (adenine-specific) inhibitor
0,070	0,030	Mannosidase inhibitor
0,068	0,028	D-Serine ammonia-lyase inhibitor
0,055	0,015	Tartrate decarboxylase inhibitor
0,154	0,114	CTGF expression inhibitor
0,144	0,105	UGT2B7 substrate
0,086	0,046	Alpha,alpha-trehalose-phosphate synthase (UDP-forming) inhibitor
0,069	0,030	Carbamoyl-serine ammonia-lyase inhibitor
0,101	0,062	Ribitol 2-dehydrogenase inhibitor
0,105	0,067	Adenosylmethionine-8-amino-7-oxononanoate transaminase inhibitor
0,098	0,059	Thiamine-phosphate diphosphorylase inhibitor
0,097	0,058	Glycerol dehydratase inhibitor
0,096	0,057	Benzaldehyde dehydrogenase (NAD+) inhibitor
0,054	0,015	Dichloromethane dehalogenase inhibitor
0,090	0,051	CYP26 substrate
0,094	0,056	Acetoacetate decarboxylase inhibitor
0,061	0,023	Na+ K+ transporting ATPase inhibitor
0,099	0,061	Cytosole dipeptidase inhibitor
0,120	0,082	Choline acetyltransferase stimulant
0,081	0,043	6-Phosphofructo-2-kinase inhibitor
0,128	0,090	Tryptophanyl aminopeptidase inhibitor
0,142	0,104	4-Chlorophenylacetate 3,4-dioxygenase inhibitor
0,168	0,130	Diamine N-acetyltransferase inhibitor
0,126	0,088	3-Hydroxyphenylacetate 6-hydroxylase inhibitor
0,122	0,084	Acylglycerone-phosphate reductase inhibitor
0,132	0,095	Glutamate synthase (ferredoxin) inhibitor

0,061	0,023	CYP17 substrate
0,119	0,082	Xaa-Pro dipeptidyl-peptidase inhibitor
0,096	0,059	Glycine transaminase inhibitor
0,074	0,037	Glutathione reductase (NADPH) stimulant
0,074	0,037	Glutathione reductase stimulant
0,121	0,084	Galactoside O-acetyltransferase inhibitor
0,105	0,069	Squalene epoxidase inhibitor
0,119	0,084	Arylalkyl acylamidase inhibitor
0,107	0,071	Aspartyl aminopeptidase inhibitor
0,150	0,114	Ferredoxin-nitrite reductase inhibitor
0,103	0,068	Malyl-CoA lyase inhibitor
0,100	0,065	Glycerol-3-phosphate dehydrogenase (NAD+) inhibitor
0,101	0,065	Glycoprotein-fucosylgalactoside alpha-N-acetylgalactosaminyltransferase inhibitor
0,110	0,075	Venombin A inhibitor
0,065	0,030	Hydroxylysine kinase inhibitor
0,173	0,138	Hydroxylamine reductase inhibitor
0,096	0,062	Alpha,alpha-trehalase inhibitor
0,081	0,046	Phosphoribulokinase inhibitor
0,061	0,026	CYP24 substrate
0,257	0,223	Mannotetraose 2-alpha-N-acetylglucosaminyltransferase inhibitor
0,061	0,027	Propionate CoA-transferase inhibitor
0,074	0,040	UGT2B7H substrate
0,074	0,040	UGT2B7Y substrate
0,073	0,039	Dextransucrase inhibitor
0,073	0,039	Deoxyhypusine synthase inhibitor
0,079	0,045	3-Dehydroquinate dehydratase inhibitor
0,097	0,064	CMP-N-acetylneuraminate monooxygenase inhibitor
0,128	0,095	Adenylate kinase inhibitor
0,077	0,044	N-acetylglucosaminyldiphosphoundecaprenol N-acetyl-beta-D-mannosaminyltransferase inhibitor
0,087	0,054	Guanidinopropionase inhibitor
0,118	0,085	Acid-sensing ion channel blocker
0,066	0,034	Beta galactosidase inhibitor
0,078	0,045	Dihydrolipoamide S-acetyltransferase inhibitor
0,063	0,031	CMP-KDO synthase inhibitor
0,116	0,084	Creatine kinase inhibitor
0,062	0,030	Biotin-[propionyl-CoA-carboxylase (ATP-hydrolysing)] ligase inhibitor
0,097	0,065	Malate dehydrogenase (oxaloacetate-decarboxylating) inhibitor

0,210	0,178	Pyroglutamyl-peptidase II inhibitor
0,081	0,050	S-methyl-5-thioribose kinase inhibitor
0,132	0,101	TH expression enhancer
0,106	0,075	Adenylate cyclase V inhibitor
0,125	0,094	Procollagen C-endopeptidase inhibitor
0,119	0,088	Cancer procoagulant inhibitor
0,102	0,071	Cerevisin inhibitor
0,203	0,172	CYP4A substrate
0,135	0,104	Acetylspermidine deacetylase inhibitor
0,133	0,103	Tryptophan alpha,beta-oxidase inhibitor
0,062	0,031	Glycine N-choloyltransferase inhibitor
0,075	0,044	L-aminoadipate-semialdehyde dehydrogenase inhibitor
0,164	0,134	Thiopurine S-methyltransferase inhibitor
0,109	0,079	Glucosamine-6-phosphate deaminase inhibitor
0,105	0,074	Decylcitrate synthase inhibitor
0,071	0,041	Alpha 3 adrenoreceptor agonist
0,034	0,004	3-Oxoacyl-[acyl-carrier-protein] synthase II inhibitor
0,069	0,039	UDP-glucose 6-dehydrogenase inhibitor
0,065	0,035	Alpha-1,6-mannosyl-glycoprotein 2-beta-N-acetylglucosaminyltransferase inhibitor
0,054	0,025	Anhydrotetracycline monooxygenase inhibitor
0,063	0,033	Deoxyadenosine kinase inhibitor
0,079	0,050	N-acetylglucosamine-6-phosphate deacetylase inhibitor
0,100	0,070	D-Ornithine 4,5-aminomutase inhibitor
0,128	0,099	Lombricine kinase inhibitor
0,101	0,071	Diiodophenylpyruvate reductase inhibitor
0,136	0,107	Tryptophan dimethylallyltransferase inhibitor
0,132	0,102	Gametolysin inhibitor
0,167	0,138	CYP1A2 inducer
0,078	0,049	Carbamoyl-phosphate synthase (ammonia) inhibitor
0,081	0,052	Serine-glyoxylate transaminase inhibitor
0,082	0,054	Neolactotetraosylceramide alpha-2,3-sialyltransferase inhibitor
0,127	0,098	Peptide-tryptophan 2,3-dioxygenase inhibitor
0,113	0,085	Aryl-alcohol dehydrogenase inhibitor
0,114	0,086	3-Isopropylmalate dehydratase inhibitor
0,158	0,130	Mucolytic
0,073	0,045	Glutamate (mGluR8) agonist
0,042	0,014	Homoisocitrate dehydrogenase inhibitor

0,082	0,054	Oligo-1,6-glucosidase inhibitor
0,089	0,061	CTP synthase inhibitor
0,103	0,075	D-cysteine desulfhydrase inhibitor
0,091	0,062	Astringent
0,067	0,039	Methylthioadenosine nucleosidase inhibitor
0,049	0,021	Orotidylate decarboxylase inhibitor
0,103	0,075	Glucosamine-1-phosphate N-acetyltransferase inhibitor
0,074	0,047	Serine-tRNA ligase inhibitor
0,161	0,134	Corticosteroid side-chain-isomerase inhibitor
0,088	0,060	D-malate dehydrogenase (decarboxylating) inhibitor
0,044	0,016	Thymidine phosphorylase inhibitor
0,066	0,039	Quinate 5-dehydrogenase inhibitor
0,112	0,085	Pyruvate dehydrogenase inhibitor
0,060	0,032	Glutamate (mGluR1) agonist
0,040	0,013	Uridine phosphorylase inhibitor
0,082	0,056	Succinate-hydroxymethylglutarate CoA-transferase inhibitor
0,040	0,014	Transaldolase inhibitor
0,070	0,043	Formimidoylglutamase inhibitor
0,066	0,039	Thioether S-methyltransferase inhibitor
0,168	0,142	N-methylhydantoinase (ATP-hydrolysing) inhibitor
0,109	0,083	Phenylacetaldehyde dehydrogenase inhibitor
0,079	0,053	Argininosuccinate lyase inhibitor
0,066	0,040	Phosphoglucomutase inhibitor
0,112	0,086	Acylphosphatase inhibitor
0,119	0,093	Succinic dehydrogenase inhibitor
0,065	0,039	Oxaloacetate tautomerase inhibitor
0,093	0,067	Formaldehyde dehydrogenase inhibitor
0,053	0,027	CYP3A11 substrate
0,048	0,022	Androst-4-ene-3,17-dione monooxygenase inhibitor
0,236	0,211	Antiinflammatory, ophthalmic
0,166	0,141	Diuretic
0,140	0,114	Choline kinase inhibitor
0,071	0,046	Isocitrate dehydrogenase (NADP+) inhibitor
0,075	0,050	Maleate hydratase inhibitor
0,098	0,073	Sarcosine oxidase inhibitor
0,145	0,120	Laxative
0,050	0,025	NADPH oxidoreductase inhibitor

0,136	0,112	Antimutagenic
0,102	0,078	Carboxylesterase inhibitor
0,114	0,089	Lanosterol 14 alpha demethylase inhibitor
0,115	0,090	Polyamine oxidase inhibitor
0,084	0,059	CMP-N-acylneuraminate phosphodiesterase inhibitor
0,088	0,063	Neuropeptide FF agonist
0,088	0,063	Neuropeptide FF2 agonist
0,099	0,075	Aspartate racemase inhibitor
0,192	0,168	CYP2C18 substrate
0,141	0,117	Contraceptive female
0,081	0,057	Muconate cycloisomerase inhibitor
0,097	0,074	2-Nitrophenol 2-monooxygenase inhibitor
0,071	0,048	Carnitine O-octanoyltransferase inhibitor
0,104	0,081	Acetolactate decarboxylase inhibitor
0,217	0,195	Antiprotozoal (Amoeba)
0,083	0,061	Diguanidinobutanase inhibitor
0,122	0,099	Shikimate 5-dehydrogenase inhibitor
0,210	0,188	CYP17 inhibitor
0,179	0,156	Saluretic
0,102	0,080	Dethiobiotin synthase inhibitor
0,115	0,092	FMN reductase inhibitor
0,068	0,046	L-lactate dehydrogenase (cytochrome) inhibitor
0,080	0,058	tRNA (guanine-N2-)-methyltransferase inhibitor
0,108	0,087	Skin whitener
0,192	0,171	Antimetastatic
0,103	0,082	Coagulant
0,120	0,099	Liver X receptor antagonist
0,101	0,080	Serine-phosphoethanolamine synthase inhibitor
0,079	0,058	SULT2A1 substrate
0,092	0,072	Aminoacylase inhibitor
0,089	0,069	Orotate reductase (NADPH) inhibitor
0,185	0,165	Monodehydroascorbate reductase (NADH) inhibitor
0,115	0,095	Beta-cyclopiazonate dehydrogenase inhibitor
0,064	0,044	GMP synthase inhibitor
0,107	0,086	Narcotic antagonist
0,109	0,089	Thiol S-methyltransferase inhibitor
0,171	0,150	TRPA1 agonist

0,128	0,108	Glycerol dehydrogenase (NADP+) inhibitor
0,130	0,110	Phosphatidate phosphatase inhibitor
0,086	0,067	Capillary fragility treatment
0,079	0,059	Cystathionine beta-lyase inhibitor
0,050	0,030	Carboxy-cis,cis-muconate cyclase inhibitor
0,049	0,029	N-acetylglucosaminyldiphosphodolichol N-acetylglucosaminyltransferase inhibitor
0,153	0,133	Fibromyalgia syndrome treatment
0,025	0,006	Calcineurin inhibitor
0,183	0,164	Antineoplastic (brain cancer)
0,096	0,077	Aryl-aldehyde dehydrogenase (NADP+) inhibitor
0,039	0,020	Membrane-oligosaccharide glycerophosphotransferase inhibitor
0,106	0,087	Beta lactamase inhibitor
0,093	0,074	Glutamate (mGluR6) antagonist
0,084	0,066	Diaminopropionate ammonia-lyase inhibitor
0,131	0,113	Quisqualate antagonist
0,041	0,023	Phosphoglycerate phosphatase inhibitor
0,077	0,059	[myelin basic protein]-arginine N-methyltransferase inhibitor
0,063	0,045	Beta-1,4-mannosyl-glycoprotein 4-beta-N-acetylglucosaminyltransferase inhibitor
0,073	0,055	Aminolevulinate transaminase inhibitor
0,058	0,040	Plus-end-directed kinesin ATPase inhibitor
0,076	0,059	Cushing's syndrome treatment
0,045	0,028	Thymidine-triphosphatase inhibitor
0,077	0,060	Anticholelithogenic
0,092	0,075	Ornithine-oxo-acid transaminase inhibitor
0,024	0,008	Gestagen-like
0,118	0,102	Tardive dyskinesia treatment
0,096	0,080	Lysyl endopeptidase inhibitor
0,097	0,081	Adenine deaminase inhibitor
0,115	0,099	Acetyl-CoA C-acyltransferase inhibitor
0,176	0,160	Antiprotozoal (Trichomonas)
0,050	0,034	Glutamate uptake inhibitor
0,122	0,106	Corticosteroid antagonist
0,070	0,054	Propanediol dehydratase inhibitor
0,119	0,103	Guanidinoacetate N-methyltransferase inhibitor
0,082	0,067	Nucleotide diphosphatase inhibitor
0,061	0,045	Malate synthase inhibitor
0,160	0,145	Severe acute respiratory syndrome treatment

0,042	0,026	Adenylate cyclase I inhibitor
0,104	0,089	Malate-CoA ligase inhibitor
0,037	0,022	Glucose-6-phosphate isomerase inhibitor
0,104	0,090	Lysine 2-monooxygenase inhibitor
0,169	0,155	Dactylysin inhibitor
0,225	0,211	Cancer associated disorders treatment
0,101	0,087	Adenylyl-sulfate kinase inhibitor
0,105	0,091	Heat shock protein 70 agonist
0,030	0,016	Alpha glucosidase I inhibitor
0,126	0,112	Tryptophanamidase inhibitor
0,038	0,024	Endo-beta-N-acetylglucosaminidase inhibitor
0,107	0,094	Inositol 1,4,5-trisphosphate 3-kinase inhibitor
0,103	0,089	Cysteine-S-conjugate beta-lyase inhibitor
0,114	0,101	Isoquinoline 1-oxidoreductase inhibitor
0,117	0,104	UGT2B15 substrate
0,080	0,066	Haloalkane dehalogenase inhibitor
0,172	0,159	5 Hydroxytryptamine 2B agonist
0,044	0,031	Glutamate (mGluR group II) agonist
0,137	0,124	Aryl hydrocarbon receptor agonist
0,076	0,064	Carboxylate reductase inhibitor
0,061	0,048	Hydroxyquinol 1,2-dioxygenase inhibitor
0,100	0,087	Arginine deiminase inhibitor
0,059	0,047	Asparagine-oxo-acid transaminase inhibitor
0,178	0,165	Antitoxic
0,042	0,030	Glutamate (mGluR2) agonist
0,043	0,031	2-Ethylmalate synthase inhibitor
0,119	0,107	Phenol 2-monooxygenase inhibitor
0,030	0,018	Phosphopyruvate hydratase inhibitor
0,094	0,082	Sulfate adenylyltransferase inhibitor
0,066	0,054	Cellulose synthase (UDP-forming) inhibitor
0,086	0,074	Agaritine gamma-glutamyltransferase inhibitor
0,078	0,067	Threonine ammonia-lyase inhibitor
0,092	0,081	Putrescine oxidase inhibitor
0,053	0,042	Sucrose alpha-glucosidase inhibitor
0,064	0,053	Nicotinic receptor alpha7 subunit antagonist
0,094	0,083	Xanthommatin reductase inhibitor
0,038	0,027	Isopentenyl-diphosphate DELTA-isomerase inhibitor

0,048	0,037	Nucleoside deoxyribosyltransferase inhibitor
0,041	0,030	Methylaspartate mutase inhibitor
0,050	0,039	Aspartate ammonia-lyase inhibitor
0,122	0,112	Tripeptidyl-peptidase I inhibitor
0,116	0,105	Dolichyl-phosphate beta-D-mannosyltransferase inhibitor
0,126	0,116	Oxalate oxidase inhibitor
0,131	0,121	Arylamine N-acetyltransferase inhibitor
0,087	0,077	Isocitrate dehydrogenase (NAD+) inhibitor
0,126	0,116	Nucleoside-diphosphatase inhibitor
0,150	0,139	Histone acetyltransferase inhibitor
0,038	0,027	Glucosylceramidase inhibitor
0,163	0,153	1-Alkylglycerophosphocholine O-acetyltransferase inhibitor
0,205	0,195	Immunosuppressant
0,055	0,045	1-Phosphofructokinase inhibitor
0,053	0,043	Assemblin inhibitor
0,052	0,043	Protein N-acetylglucosaminyltransferase inhibitor
0,083	0,073	Acyloxyacyl hydrolase inhibitor
0,066	0,057	Sphingosine kinase inhibitor
0,018	0,009	Antiprotozoal activity enhancer
0,074	0,065	Isocitrate lyase inhibitor
0,075	0,066	[acyl-carrier-protein] S-malonyltransferase inhibitor
0,078	0,069	UDP-N-acetylglucosamine 1-carboxyvinyltransferase inhibitor
0,056	0,047	Altronate dehydratase inhibitor
0,138	0,129	CYP1B substrate
0,048	0,040	Tubulin GTPase inhibitor
0,080	0,072	Interleukin 1a antagonist
0,064	0,056	Betaine-aldehyde dehydrogenase inhibitor
0,043	0,035	Glucose-1-phosphate adenylyltransferase inhibitor
0,118	0,110	Bacterial efflux pump inhibitor
0,088	0,080	Phosphoserine phosphatase inhibitor
0,016	0,008	Kanamycin kinase inhibitor
0,072	0,064	Scytalone dehydratase inhibitor
0,049	0,041	D-Alanine-poly(phosphoribitol) ligase inhibitor
0,042	0,034	Fructose 5-dehydrogenase (NADP+) inhibitor
0,026	0,018	Tartrate dehydrogenase inhibitor
0,095	0,087	Pyridoxine 5-dehydrogenase inhibitor
0,039	0,032	Glucose-1-phosphatase inhibitor

0,025	0,017	Corticosteroid-like
0,108	0,101	Saccharopine dehydrogenase (NADP+, L-glutamate-forming) inhibitor
0,022	0,015	Cortisone alpha-reductase inhibitor
0,090	0,083	Glutamine-tRNA ligase inhibitor
0,047	0,040	AMP nucleosidase inhibitor
0,179	0,172	Phospholipase C inhibitor
0,070	0,063	4-Pyridoxolactonase inhibitor
0,149	0,142	Beta-carotene 15,15'-monooxygenase inhibitor
0,068	0,061	1-Pyrroline-5-carboxylate dehydrogenase inhibitor
0,050	0,043	Chalcone isomerase inhibitor
0,106	0,100	Cholesterol synthesis inhibitor
0,123	0,117	Hypoxia inducible factor 1 alpha inhibitor
0,069	0,063	Thiosulfate sulfurtransferase inhibitor
0,050	0,044	Pyruvate, water dikinase inhibitor
0,119	0,113	N-Carbamoyl-D-amino acid hydrolase inhibitor
0,124	0,118	L-glucuronate reductase inhibitor
0,111	0,106	Acylaminoacyl-peptidase inhibitor
0,038	0,033	Strombine dehydrogenase inhibitor
0,037	0,031	GABA B receptor antagonist
0,079	0,074	CYP2B4 substrate
0,128	0,123	Envelysin inhibitor
0,049	0,044	Uracil phosphoribosyltransferase inhibitor
0,095	0,090	Estrone sulfotransferase inhibitor
0,043	0,038	Polyphosphate kinase inhibitor
0,104	0,099	Prolactin inhibitor
0,129	0,125	Glucuronate isomerase inhibitor
0,087	0,083	Chloramphenicol O-acetyltransferase inhibitor
0,043	0,038	Pregnane X receptor antagonist
0,108	0,104	Aldehyde dehydrogenase (NAD+) inhibitor
0,033	0,029	Ribose-5-phosphate adenylyltransferase inhibitor
0,065	0,061	Phosphoglycolate phosphatase inhibitor
0,073	0,069	Carboxypeptidase M inhibitor
0,033	0,029	RNA agonist
0,024	0,020	Dolichyldiphosphatase inhibitor
0,096	0,092	Asparaginase inhibitor
0,022	0,018	Dopamine D2B antagonist
0,032	0,029	Diphosphate-serine phosphotransferase inhibitor

0,097	0,094	Alkanal monooxygenase (FMN-linked) inhibitor
0,016	0,013	CYP11B1 substrate
0,021	0,019	CYP27A substrate
0,034	0,032	Phosphoserine transaminase inhibitor
0,053	0,050	Aryl hydrocarbon receptor antagonist
0,111	0,109	Diphosphomevalonate decarboxylase inhibitor
0,036	0,034	[Isocitrate dehydrogenase (NADP+)] kinase inhibitor
0,037	0,035	Sodium-dependent vitamin C transporter 2 inhibitor
0,074	0,072	Alcohol oxidase inhibitor
0,167	0,166	Diabetic nephropathy treatment
0,098	0,096	Deoxycytidylate 5-hydroxymethyltransferase inhibitor
0,058	0,056	Citrate (Si)-synthase inhibitor
0,143	0,142	Histidinol-phosphatase inhibitor
0,022	0,020	Sigma 3 receptor antagonist
0,027	0,025	Glutamate (mGluR1a) agonist
0,027	0,025	Glutamate (mGluR5a) agonist
0,058	0,057	Fumarylacetoacetase inhibitor
0,060	0,059	3-Hydroxydecanoyl-[acyl-carrier-protein] dehydratase inhibitor
0,058	0,058	NMN nucleosidase inhibitor
0,044	0,044	Mannosyl-oligosaccharide glucosidase inhibitor

Complex 4 All

◉ All ○ Pa>Pi ○ Pa>0,3 ○ Pa>0,7

Pa	Pi	Activity
0,799	0,034	Phobic disorders treatment
0,731	0,003	Antineoplastic (non-Hodgkin's lymphoma)
0,733	0,008	Phosphatase inhibitor
0,752	0,038	Testosterone 17beta-dehydrogenase (NADP+) inhibitor
0,733	0,025	Nicotinic alpha6beta3beta4alpha5 receptor antagonist
0,731	0,036	Chymosin inhibitor
0,731	0,036	Acrocylindropepsin inhibitor
0,731	0,036	Saccharopepsin inhibitor
0,693	0,009	Antiasthmatic

0,692	0,010	Glucan 1,4-alpha-maltotriohydrolase inhibitor
0,692	0,024	5-O-(4-coumaroyl)-D-quinate 3'-monooxygenase inhibitor
0,667	0,033	Nicotinic alpha2beta2 receptor antagonist
0,663	0,041	Sugar-phosphatase inhibitor
0,654	0,033	Acylcarnitine hydrolase inhibitor
0,647	0,027	Alkylacetylglycerophosphatase inhibitor
0,613	0,038	Ribulose-phosphate 3-epimerase inhibitor
0,579	0,016	Antipruritic, allergic
0,644	0,082	Aspulvinone dimethylallyltransferase inhibitor
0,567	0,013	Dolichyl-diphosphooligosaccharide-protein glycotransferase inhibitor
0,628	0,076	CYP2J substrate
0,554	0,009	Imidazoline receptor agonist
0,580	0,043	Antidyskinetic
0,604	0,067	Polyporopepsin inhibitor
0,581	0,060	Glutamyl endopeptidase II inhibitor
0,538	0,027	Antipruritic
0,545	0,038	27-Hydroxycholesterol 7alpha-monooxygenase inhibitor
0,519	0,015	Rhinitis treatment
0,528	0,024	Mannan endo-1,4-beta-mannosidase inhibitor
0,558	0,055	Antineoplastic
0,606	0,105	Ubiquinol-cytochrome-c reductase inhibitor
0,520	0,026	CYP2A4 substrate
0,568	0,074	CYP2J2 substrate
0,503	0,010	Antidiabetic (type 2)
0,536	0,055	Alkenylglycerophosphocholine hydrolase inhibitor
0,536	0,057	Complement factor D inhibitor
0,498	0,028	Antiallergic
0,479	0,011	RNA directed DNA polymerase inhibitor
0,529	0,061	NADPH peroxidase inhibitor
0,523	0,057	Platelet aggregation stimulant
0,494	0,033	Dermatologic
0,470	0,011	DNA polymerase I inhibitor
0,478	0,020	Dementia treatment
0,504	0,051	Chloride peroxidase inhibitor
0,467	0,026	Membrane permeability enhancer
0,502	0,064	Macrophage colony stimulating factor agonist
0,493	0,059	UDP-N-acetylglucosamine 4-epimerase inhibitor

0,492	0,060	Glucan endo-1,3-beta-D-glucosidase inhibitor
0,469	0,037	Acetylesterase inhibitor
0,465	0,034	Glyoxylate reductase inhibitor
0,441	0,010	Antiviral (Hepatitis B)
0,455	0,025	Albendazole monooxygenase inhibitor
0,479	0,054	Pterin deaminase inhibitor
0,434	0,014	Imidazoline I1 receptor agonist
0,480	0,063	Limulus clotting factor B inhibitor
0,474	0,058	Glucan endo-1,6-beta-glucosidase inhibitor
0,449	0,034	Peptidoglycan glycosyltransferase inhibitor
0,456	0,041	CYP2B5 substrate
0,423	0,011	Antipruritic, non-allergic
0,457	0,044	Antiviral (Rhinovirus)
0,422	0,011	Opioid dependency treatment
0,437	0,026	Cutinase inhibitor
0,470	0,061	Phosphatidylcholine-retinol O-acyltransferase inhibitor
0,455	0,047	L-glutamate oxidase inhibitor
0,484	0,079	Platelet adhesion inhibitor
0,476	0,075	Electron-transferring-flavoprotein dehydrogenase inhibitor
0,404	0,011	Chronic obstructive pulmonary disease treatment
0,434	0,042	All-trans-retinyl-palmitate hydrolase inhibitor
0,448	0,057	Cyclic AMP agonist
0,447	0,056	Feruloyl esterase inhibitor
0,454	0,063	1,4-Lactonase inhibitor
0,461	0,070	Carboxypeptidase Taq inhibitor
0,430	0,039	Pullulanase inhibitor
0,444	0,055	Exoribonuclease II inhibitor
0,417	0,028	Phosphatidylinositol diacylglycerol-lyase inhibitor
0,474	0,089	Omptin inhibitor
0,398	0,013	Endo-1,3(4)-beta-glucanase inhibitor
0,435	0,051	Apyrase inhibitor
0,460	0,076	Lysase inhibitor
0,405	0,021	Prostate cancer treatment
0,440	0,060	Hydrogen dehydrogenase inhibitor
0,485	0,105	Glycosylphosphatidylinositol phospholipase D inhibitor
0,486	0,107	Kidney function stimulant
0,437	0,061	Trimethylamine-oxide aldolase inhibitor

0,432	0,058	CYP3A5 substrate
0,446	0,074	Venombin AB inhibitor
0,417	0,045	Simian immunodeficiency virus proteinase inhibitor
0,441	0,071	JAK2 expression inhibitor
0,420	0,056	Gluconate 5-dehydrogenase inhibitor
0,385	0,024	Oxidizing agent
0,418	0,058	CYP2D15 substrate
0,415	0,059	3-Hydroxybenzoate 6-monooxygenase inhibitor
0,357	0,002	Glucocorticoid agonist
0,391	0,040	Antiviral (Herpes)
0,364	0,014	Vascular dementia treatment
0,376	0,028	Sorbitol-6-phosphate 2-dehydrogenase inhibitor
0,394	0,046	Malate oxidase inhibitor
0,379	0,031	Taurine-2-oxoglutarate transaminase inhibitor
0,411	0,063	Immunosuppressant
0,496	0,151	Membrane permeability inhibitor
0,459	0,115	Fusarinine-C ornithinesterase inhibitor
0,401	0,057	Alkenylglycerophosphoethanolamine hydrolase inhibitor
0,373	0,030	Xylan endo-1,3-beta-xylosidase inhibitor
0,402	0,060	Acetylgalactosaminyl-O-glycosyl-glycoprotein beta-1,3-N-acetylglucosaminyltransferase inhibitor
0,426	0,089	Alopecia treatment
0,401	0,064	Adenomatous polyposis treatment
0,386	0,049	Aspergillopepsin I inhibitor
0,455	0,120	Pseudolysin inhibitor
0,362	0,028	CDP-diacylglycerol-glycerol-3-phosphate 3-phosphatidyltransferase inhibitor
0,393	0,060	Na+-transporting two-sector ATPase inhibitor
0,347	0,014	Systemic lupus erythematosus treatment
0,421	0,091	Thioredoxin inhibitor
0,369	0,040	Glucan 1,4-alpha-maltotetraohydrolase inhibitor
0,366	0,038	Glycolate dehydrogenase inhibitor
0,367	0,039	Poly(beta-D-mannuronate) lyase inhibitor
0,356	0,033	Shikimate O-hydroxycinnamoyltransferase inhibitor
0,380	0,057	GABA aminotransferase inhibitor
0,331	0,010	Alpha-pinene-oxide decyclase inhibitor
0,382	0,063	N-acetylneuraminate 7-O(or 9-O)-acetyltransferase inhibitor
0,347	0,030	Antiinflammatory, ophthalmic
0,334	0,019	Maltose-transporting ATPase inhibitor

0,393	0,078	Fatty-acyl-CoA synthase inhibitor
0,357	0,043	Cardiovascular analeptic
0,400	0,086	Lysine 2,3-aminomutase inhibitor
0,352	0,037	Clavaminate synthase inhibitor
0,426	0,112	Phthalate 4,5-dioxygenase inhibitor
0,381	0,068	Formaldehyde transketolase inhibitor
0,391	0,079	Fragilysin inhibitor
0,402	0,091	Neurotransmitter antagonist
0,429	0,120	Acetylcholine neuromuscular blocking agent
0,333	0,029	Sclerosant
0,349	0,047	Undecaprenyldiphospho-muramoylpentapeptide beta-N-acetylglucosaminyltransferase inhibitor
0,353	0,051	CYP7 inhibitor
0,329	0,028	Galactolipase inhibitor
0,338	0,037	Yeast ribonuclease inhibitor
0,306	0,005	Alternansucrase inhibitor
0,351	0,050	Mannose isomerase inhibitor
0,385	0,085	Vasoprotector
0,407	0,108	Phospholipid-translocating ATPase inhibitor
0,411	0,113	Ovulation inhibitor
0,352	0,054	H+-transporting two-sector ATPase inhibitor
0,381	0,084	CYP2D16 substrate
0,304	0,009	Allergic rhinitis treatment
0,344	0,049	2-Haloacid dehalogenase inhibitor
0,338	0,044	CYP2B11 substrate
0,411	0,117	Protein-disulfide reductase (glutathione) inhibitor
0,340	0,047	Cyclomaltodextrinase inhibitor
0,374	0,081	Dehydro-L-gulonate decarboxylase inhibitor
0,314	0,022	Undecaprenyl-diphosphatase inhibitor
0,336	0,045	Fructan beta-fructosidase inhibitor
0,325	0,037	GST M substrate
0,297	0,011	GST T substrate
0,297	0,011	GST T1-1 substrate
0,315	0,029	Styrene-oxide isomerase inhibitor
0,357	0,071	Cyclohexanone monooxygenase inhibitor
0,398	0,113	NADPH-cytochrome-c2 reductase inhibitor
0,353	0,068	Mucinaminylserine mucinaminidase inhibitor
0,359	0,074	EIF4E expression inhibitor

0,315	0,031	Phosphoenolpyruvate mutase inhibitor
0,345	0,061	Limulus clotting factor C inhibitor
0,341	0,058	Nitrite reductase (NO-forming) inhibitor
0,383	0,101	Erythropoiesis stimulant
0,327	0,046	H+-exporting ATPase inhibitor
0,356	0,076	Thymidylate 5'-phosphatase inhibitor
0,337	0,059	IgA-specific serine endopeptidase inhibitor
0,331	0,053	Sulfite oxidase inhibitor
0,294	0,017	Ligase inhibitor
0,315	0,038	Pediculicide
0,381	0,105	Leukopoiesis stimulant
0,298	0,021	Hydroxysteroid dehydrogenase inhibitor
0,308	0,036	D-alanine 2-hydroxymethyltransferase inhibitor
0,336	0,064	Steroid N-acetylglucosaminyltransferase inhibitor
0,369	0,099	GST A substrate
0,305	0,036	Pseudouridylate synthase inhibitor
0,338	0,068	CYP2D2 inhibitor
0,334	0,067	RELA expression inhibitor
0,352	0,085	Polyamine-transporting ATPase inhibitor
0,359	0,094	Arginine 2-monooxygenase inhibitor
0,297	0,032	Fumarate reductase (NADH) inhibitor
0,354	0,090	G-protein-coupled receptor kinase inhibitor
0,354	0,090	Beta-adrenergic receptor kinase inhibitor
0,346	0,082	Phenol O-methyltransferase inhibitor
0,318	0,055	Hematopoietic inhibitor
0,311	0,050	Glyoxylate oxidase inhibitor
0,310	0,050	DNA synthesis inhibitor
0,326	0,067	Phosphopantothenoylcysteine decarboxylase inhibitor
0,400	0,140	CYP3A2 substrate
0,387	0,128	5 Hydroxytryptamine uptake stimulant
0,308	0,049	CYP2B10 substrate
0,293	0,034	Antineoplastic (small cell lung cancer)
0,264	0,007	Endometrios treatment
0,364	0,107	(R)-6-hydroxynicotine oxidase inhibitor
0,264	0,007	Arachidonic acid antagonist
0,260	0,004	Cyclin-dependent kinase 7 inhibitor
0,290	0,033	Licheninase inhibitor

0,396	0,141	Membrane integrity agonist
0,352	0,097	Ecdysone 20-monooxygenase inhibitor
0,300	0,046	Steroid synthesis inhibitor
0,324	0,070	Antidiabetic
0,312	0,058	Dextranase inhibitor
0,261	0,008	Glucan 1,3-beta-glucosidase inhibitor
0,319	0,066	Opioid kappa 3 receptor antagonist
0,278	0,025	Antimycoplasmal
0,289	0,037	Dopamine release stimulant
0,355	0,104	4-Nitrophenol 2-monooxygenase inhibitor
0,322	0,071	Peptide-N4-(N-acetyl-beta-glucosaminyl)asparagine amidase inhibitor
0,334	0,083	(S)-6-hydroxynicotine oxidase inhibitor
0,320	0,069	N-formylmethionyl-peptidase inhibitor
0,297	0,047	Carminative
0,343	0,093	Aspartate-phenylpyruvate transaminase inhibitor
0,354	0,104	Fibrolase inhibitor
0,297	0,048	2,3,4,5-Tetrahydropyridine-2,6-dicarboxylate N-succinyltransferase inhibitor
0,369	0,121	CYP2C12 substrate
0,304	0,056	Antineoplastic (pancreatic cancer)
0,363	0,116	Aminobutyraldehyde dehydrogenase inhibitor
0,341	0,095	Cl--transporting ATPase inhibitor
0,341	0,096	Caspase 8 stimulant
0,321	0,077	Phosphoinositide 5-phosphatase inhibitor
0,294	0,051	Quinoprotein glucose dehydrogenase inhibitor
0,300	0,057	DELTA14-sterol reductase inhibitor
0,345	0,103	Dimethylargininase inhibitor
0,276	0,035	Bisphosphoglycerate mutase inhibitor
0,318	0,081	Prostaglandin-A1 DELTA-isomerase inhibitor
0,334	0,097	Glycerol-3-phosphate oxidase inhibitor
0,356	0,119	Antiinflammatory
0,329	0,094	Manganese peroxidase inhibitor
0,288	0,054	Hyaluronic acid agonist
0,369	0,137	Antiviral (Picornavirus)
0,255	0,023	GABA B receptor agonist
0,334	0,103	Antimyopathies
0,323	0,093	Histidine N-acetyltransferase inhibitor
0,307	0,078	Creatininase inhibitor

0,274	0,046	Alpha-N-acetylglucosaminidase inhibitor
0,271	0,043	Alkylglycerophosphoethanolamine phosphodiesterase inhibitor
0,281	0,054	Adrenaline release stimulant
0,330	0,104	CYP4A11 substrate
0,252	0,027	2-Dehydropantoate aldolase inhibitor
0,300	0,075	tRNA-pseudouridine synthase I inhibitor
0,273	0,049	Oryzin inhibitor
0,267	0,045	Myeloblastin inhibitor
0,235	0,013	N-acetylneuraminate lyase inhibitor
0,297	0,076	Lysostaphin inhibitor
0,286	0,065	RNA synthesis inhibitor
0,292	0,072	Arylmalonate decarboxylase inhibitor
0,301	0,081	Antimetastatic
0,316	0,096	CYP2A8 substrate
0,244	0,025	Deoxyribonuclease I inhibitor
0,301	0,082	3-Cyanoalanine hydratase inhibitor
0,320	0,102	Sphinganine kinase inhibitor
0,396	0,178	Calcium channel (voltage-sensitive) activator
0,281	0,064	NF-E2-related factor 2 stimulant
0,300	0,084	Alcohol dehydrogenase (acceptor) inhibitor
0,314	0,098	Tpr proteinase (Porphyromonas gingivalis) inhibitor
0,340	0,125	Histamine release stimulant
0,276	0,061	CYP2A2 substrate
0,311	0,096	Pro-opiomelanocortin converting enzyme inhibitor
0,285	0,070	Hydroxylamine reductase (NADH) inhibitor
0,222	0,008	4-Carboxymethyl-4-methylbutenolide mutase inhibitor
0,258	0,044	Polyneuridine-aldehyde esterase inhibitor
0,340	0,128	Pin1 inhibitor
0,246	0,035	Alpha-glucuronidase inhibitor
0,236	0,025	Galacturan 1,4-alpha-galacturonidase inhibitor
0,246	0,035	Tropinesterase inhibitor
0,224	0,014	Aconitate decarboxylase inhibitor
0,271	0,061	Linoleoyl-CoA desaturase inhibitor
0,345	0,136	Cytoprotectant
0,241	0,034	Ribonucleoside triphosphate reductase inhibitor
0,259	0,052	Protein-Npi-phosphohistidine-sugar phosphotransferase inhibitor
0,351	0,145	Antinociceptive

0,243	0,037	Rhizopuspepsin inhibitor
0,256	0,050	N-Acyl-D-aspartate deacylase inhibitor
0,215	0,009	Keratoses actinic (solar) treatment
0,325	0,123	CYP3A1 substrate
0,284	0,083	Glutamine-phenylpyruvate transaminase inhibitor
0,257	0,056	Aspergillopepsin II inhibitor
0,227	0,027	2,2-Dialkylglycine decarboxylase (pyruvate) inhibitor
0,221	0,020	DNA nucleotidylexotransferase inhibitor
0,331	0,131	2-Dehydropantoate 2-reductase inhibitor
0,314	0,116	Linoleate diol synthase inhibitor
0,287	0,088	3-Phytase inhibitor
0,257	0,059	Aldosterone antagonist
0,294	0,097	Methylamine-glutamate N-methyltransferase inhibitor
0,288	0,090	Adenylyl-sulfate reductase inhibitor
0,246	0,048	Poly(alpha-L-guluronate) lyase inhibitor
0,282	0,085	Chitosanase inhibitor
0,322	0,125	Rubredoxin-NAD+ reductase inhibitor
0,309	0,113	Centromere associated protein inhibitor
0,251	0,056	Horrilysin inhibitor
0,271	0,076	2-Hydroxymuconate-semialdehyde hydrolase inhibitor
0,232	0,039	1,4-Alpha-glucan branching enzyme inhibitor
0,212	0,019	Trehalose-phosphatase inhibitor
0,302	0,109	Glucose oxidase inhibitor
0,250	0,058	UGT2B28 substrate
0,282	0,090	ADP-thymidine kinase inhibitor
0,279	0,087	N-acylmannosamine kinase inhibitor
0,286	0,094	Chenodeoxycholoyltaurine hydrolase inhibitor
0,249	0,058	Chaperonin ATPase inhibitor
0,267	0,077	CYP2C29 substrate
0,260	0,071	Dipeptidase E inhibitor
0,271	0,082	Cytostatic
0,252	0,063	Antiprotozoal (Trichomonas)
0,238	0,051	Vitamin-K-epoxide reductase (warfarin-insensitive) inhibitor
0,270	0,083	Glutamate-tRNA ligase inhibitor
0,208	0,021	Alpha-N-acetylgalactosaminidase inhibitor
0,275	0,089	D-lactaldehyde dehydrogenase inhibitor
0,282	0,097	CYP2F1 substrate

0,283	0,099	Fucosterol-epoxide lyase inhibitor
0,296	0,112	Sulfite dehydrogenase inhibitor
0,304	0,120	S-formylglutathione hydrolase inhibitor
0,343	0,159	Thromboxane B2 antagonist
0,234	0,051	Anthranilate-CoA ligase inhibitor
0,245	0,062	Antineoplastic (brain cancer)
0,237	0,055	N-acetyllactosaminide beta-1,3-N-acetylglucosaminyltransferase inhibitor
0,190	0,008	Inositol-3-phosphate synthase inhibitor
0,241	0,059	2-Oxoaldehyde dehydrogenase (NADP+) inhibitor
0,260	0,078	Long-chain-aldehyde dehydrogenase inhibitor
0,247	0,065	Levanase inhibitor
0,196	0,015	CYP3A5 inducer
0,275	0,094	Lysyl oxidase inhibitor
0,241	0,060	2-Hydroxy-3-oxoadipate synthase inhibitor
0,263	0,083	MAP kinase kinase 4 inhibitor
0,304	0,124	Biotinidase inhibitor
0,215	0,035	BRAF expression inhibitor
0,223	0,045	NAD+ synthase (glutamine-hydrolysing) inhibitor
0,187	0,008	Tardive dyskinesia treatment
0,219	0,040	3-Chloro-D-alanine dehydrochlorinase inhibitor
0,224	0,045	D-xylulose reductase inhibitor
0,279	0,100	Antisecretoric
0,233	0,054	Mannitol-1-phosphatase inhibitor
0,267	0,089	Peptide alpha-N-acetyltransferase inhibitor
0,229	0,052	Dynein ATPase inhibitor
0,203	0,026	Riboflavin phosphotransferase inhibitor
0,245	0,068	Benzaldehyde dehydrogenase (NADP+) inhibitor
0,269	0,093	Isopenicillin-N epimerase inhibitor
0,241	0,065	Flavin-containing monooxygenase inhibitor
0,232	0,057	Cellulose 1,4-beta-cellobiosidase inhibitor
0,255	0,081	Transketolase inhibitor
0,211	0,038	Alkene monooxygenase inhibitor
0,247	0,074	Salicylate 1-monooxygenase inhibitor
0,206	0,033	Ferredoxin-NADP+ reductase inhibitor
0,301	0,128	CYP2C9 inducer
0,233	0,061	3-Methylbutanal reductase inhibitor
0,182	0,010	Amylo-alpha-1,6-glucosidase inhibitor

0,246	0,075	Gluconolactonase inhibitor
0,291	0,120	Membrane integrity antagonist
0,241	0,071	Bisphosphoglycerate phosphatase inhibitor
0,232	0,062	Glutarate-semialdehyde dehydrogenase inhibitor
0,266	0,096	Antipsoriatic
0,220	0,051	Aureolysin inhibitor
0,290	0,121	Hydroxylamine oxidase inhibitor
0,305	0,137	CYP2A1 substrate
0,231	0,063	Alpha-1,6-mannosyl-glycoprotein 4-beta-N-acetylglucosaminyltransferase inhibitor
0,247	0,079	NADH kinase inhibitor
0,274	0,107	Plastoquinol-plastocyanin reductase inhibitor
0,228	0,061	Prunasin beta-glucosidase inhibitor
0,195	0,029	Estradiol 17alpha-dehydrogenase inhibitor
0,249	0,084	Glycerol-3-phosphate dehydrogenase inhibitor
0,249	0,084	Endothelial growth factor antagonist
0,261	0,097	CYP2A5 substrate
0,226	0,062	Cyclooxygenase substrate
0,232	0,069	N-hydroxy-2-acetamidofluorene reductase inhibitor
0,222	0,059	Polygalacturonase inhibitor
0,204	0,041	Glycerol-1-phosphatase inhibitor
0,167	0,004	Inositol 1,4,5-triphosphate receptor antagonist
0,206	0,043	Cycloartenol synthase inhibitor
0,187	0,025	Interferon antagonist
0,200	0,038	CYP2B18 substrate
0,268	0,106	Chitinase inhibitor
0,240	0,078	Ethanolamine-phosphate cytidylyltransferase inhibitor
0,201	0,040	Antineoplastic (renal cancer)
0,230	0,068	Pappalysin-1 inhibitor
0,263	0,102	P-benzoquinone reductase (NADPH) inhibitor
0,305	0,144	Pancreatic elastase inhibitor
0,208	0,048	Inulinase inhibitor
0,281	0,122	Cyanoalanine nitrilase inhibitor
0,179	0,020	Isoamylase inhibitor
0,268	0,109	Peptidyl-dipeptidase Dcp inhibitor
0,210	0,051	Aryldialkylphosphatase inhibitor
0,181	0,023	Interferon gamma antagonist
0,279	0,122	(R)-Pantolactone dehydrogenase (flavin) inhibitor

0,193	0,037	Glucan 1,6-alpha-glucosidase inhibitor
0,189	0,033	Carnitine dehydratase inhibitor
0,305	0,150	Chlordecone reductase inhibitor
0,201	0,046	Bile-salt sulfotransferase inhibitor
0,205	0,051	Lactaldehyde reductase inhibitor
0,244	0,090	Hepatoprotectant
0,275	0,120	MMP9 expression inhibitor
0,278	0,124	5 Hydroxytryptamine release stimulant
0,281	0,127	Leukopoiesis inhibitor
0,208	0,055	Snapalysin inhibitor
0,194	0,040	Allantoate deiminase inhibitor
0,192	0,040	Antineoplastic (ovarian cancer)
0,217	0,065	Glycine dehydrogenase (decarboxylating) inhibitor
0,198	0,046	Aspartate-tRNA ligase inhibitor
0,210	0,058	(S)-3-amino-2-methylpropionate transaminase inhibitor
0,153	0,002	Lipocortins synthesis agonist
0,234	0,083	S-alkylcysteine lyase inhibitor
0,209	0,059	Sedoheptulose-bisphosphatase inhibitor
0,201	0,051	Glucan 1,4-beta-glucosidase inhibitor
0,286	0,137	Cytochrome P450 stimulant
0,198	0,049	2-Haloacid dehalogenase (configuration-inverting) inhibitor
0,188	0,039	L-iduronidase inhibitor
0,244	0,095	Aspergillus nuclease S1 inhibitor
0,216	0,067	GABA C receptor agonist
0,285	0,136	Malate dehydrogenase (acceptor) inhibitor
0,190	0,043	Retinyl-palmitate esterase inhibitor
0,168	0,022	Demethylsterigmatocystin 6-O-methyltransferase inhibitor
0,228	0,082	Antiperistaltic
0,313	0,167	MAP kinase stimulant
0,192	0,046	Cyclamate sulfohydrolase inhibitor
0,191	0,045	Acetylenecarboxylate hydratase inhibitor
0,259	0,115	CYP3A7 substrate
0,286	0,142	Gonadotropin antagonist
0,183	0,039	Glycerone-phosphate O-acyltransferase inhibitor
0,246	0,102	4-Coumarate-CoA ligase inhibitor
0,239	0,096	Transcription factor NF kappa A inhibitor
0,196	0,053	Nitric oxide scavenger

0,211	0,069	Crotonoyl-[acyl-carrier-protein] hydratase inhibitor
0,179	0,037	Protein-glucosylgalactosylhydroxylysine glucosidase inhibitor
0,273	0,132	CYP2E1 inducer
0,218	0,078	N-acetyllactosamine synthase inhibitor
0,222	0,082	Cyclohexyl-isocyanide hydratase inhibitor
0,214	0,073	Ornithine cyclodeaminase inhibitor
0,309	0,169	Oxygen scavenger
0,243	0,103	Pyruvate decarboxylase inhibitor
0,178	0,040	Glucuronolactone reductase inhibitor
0,190	0,052	Myosin-light-chain-phosphatase inhibitor
0,246	0,109	Methylumbelliferyl-acetate deacetylase inhibitor
0,141	0,004	Alpha 3 adrenoreceptor agonist
0,205	0,068	D-threo-aldose 1-dehydrogenase inhibitor
0,228	0,091	ATPase stimulant
0,168	0,032	(R)-limonene 6-monooxygenase inhibitor
0,207	0,071	N-(long-chain-acyl)ethanolamine deacylase inhibitor
0,191	0,055	Microtubule formation inhibitor
0,216	0,080	CYP2G1 substrate
0,147	0,012	Phosphoglucomutase inhibitor
0,175	0,040	Glycopeptide alpha-N-acetylgalactosaminidase inhibitor
0,243	0,109	N-benzyloxycarbonylglycine hydrolase inhibitor
0,170	0,035	Pectin lyase inhibitor
0,175	0,040	Cholesterol oxidase inhibitor
0,265	0,132	Myc inhibitor
0,223	0,089	Glutathione dehydrogenase (ascorbate) inhibitor
0,146	0,013	Dextransucrase inhibitor
0,196	0,063	2,3-Dihydroxyindole 2,3-dioxygenase inhibitor
0,149	0,016	2-Methylcitrate dehydratase inhibitor
0,222	0,090	Aspartate-ammonia ligase inhibitor
0,205	0,072	Procollagen N-endopeptidase inhibitor
0,193	0,061	Sterol 3-beta-glucosyltransferase inhibitor
0,180	0,049	Phosphoenolpyruvate-protein phosphotransferase inhibitor
0,222	0,091	Lactose synthase inhibitor
0,195	0,064	Leucine dehydrogenase inhibitor
0,172	0,041	Gaucher disease treatment
0,194	0,063	Alkylglycerone-phosphate synthase inhibitor
0,248	0,118	Para amino benzoic acid antagonist

0,202	0,072	Tauropine dehydrogenase inhibitor
0,225	0,095	Cholestanetriol 26-monooxygenase inhibitor
0,252	0,123	Enteropeptidase inhibitor
0,153	0,024	Antitreponemal
0,165	0,036	Cocain dependency treatment
0,225	0,097	Carbon-monoxide dehydrogenase inhibitor
0,269	0,141	Endopeptidase So inhibitor
0,150	0,022	Shab potassium channel blocker
0,225	0,097	NOS2 expression inhibitor
0,161	0,034	Choloylglycine hydrolase inhibitor
0,201	0,074	Rhodotorulapepsin inhibitor
0,189	0,062	Alpha-amylase inhibitor
0,184	0,057	GST M1-1 substrate
0,265	0,139	Antiseborrheic
0,134	0,007	Anesthetic inhalation
0,175	0,050	Ganglioside galactosyltransferase inhibitor
0,316	0,192	Octopamine antagonist
0,165	0,041	Deoxyribose-phosphate aldolase inhibitor
0,173	0,049	Protein synthesis stimulant
0,226	0,102	Succinate-semialdehyde dehydrogenase [NAD(P)+] inhibitor
0,137	0,013	Thromboxane antagonist
0,207	0,083	Di-trans,poly-cis-decaprenylcistransferase inhibitor
0,170	0,046	3-Ketovalidoxylamine C-N-lyase inhibitor
0,207	0,084	Carboxypeptidase D inhibitor
0,175	0,053	Testosterone 17beta-dehydrogenase inhibitor
0,210	0,088	Morphine 6-dehydrogenase inhibitor
0,136	0,014	Fatty-acyl-ethyl-ester synthase inhibitor
0,239	0,117	Thiosulfate dehydrogenase inhibitor
0,158	0,037	Pregnane X receptor agonist
0,171	0,049	Saccharolysin inhibitor
0,241	0,119	Ferredoxin-NAD+ reductase inhibitor
0,241	0,119	Naphthalene 1,2-dioxygenase inhibitor
0,199	0,078	Peptidyl-dipeptidase B inhibitor
0,186	0,065	Serratia marcescens nuclease inhibitor
0,213	0,093	DNA ligase (ATP) inhibitor
0,174	0,054	Polar-amino-acid-transporting ATPase inhibitor
0,207	0,087	Dimethylmaleate hydratase inhibitor

0,162	0,042	Glutamate-1-semialdehyde 2,1-aminomutase inhibitor
0,136	0,017	3(or 17)beta-hydroxysteroid dehydrogenase inhibitor
0,199	0,080	Guanosine-3',5'-bis(diphosphate) 3'-diphosphatase inhibitor
0,259	0,140	NAD(P)+-arginine ADP-ribosyltransferase inhibitor
0,182	0,064	ADP-ribosylarginine hydrolase inhibitor
0,179	0,061	Farnesyltranstransferase inhibitor
0,131	0,014	Imidazoleglycerol-phosphate dehydratase inhibitor
0,151	0,034	Glyceraldehyde-3-phosphate dehydrogenase (phosphorylating) inhibitor
0,169	0,052	Dolichyl-phosphatase inhibitor
0,196	0,080	UGT2B17 substrate
0,153	0,037	Trimethyllysine dioxygenase inhibitor
0,211	0,096	Opheline kinase inhibitor
0,211	0,096	Taurocyamine kinase inhibitor
0,200	0,085	Glycerol 2-dehydrogenase (NADP+) inhibitor
0,175	0,060	Trans-pentaprenyltranstransferase inhibitor
0,186	0,071	Dependence treatment
0,210	0,096	[acyl-carrier-protein] S-acetyltransferase inhibitor
0,236	0,121	4-Methoxybenzoate monooxygenase (O-demethylating) inhibitor
0,283	0,169	RNA-directed RNA polymerase inhibitor
0,133	0,019	Mycodextranase inhibitor
0,168	0,054	Mannan endo-1,6-alpha-mannosidase inhibitor
0,169	0,056	Glutamate (mGluR5) agonist
0,186	0,073	Sweetener
0,305	0,192	Fructose 5-dehydrogenase inhibitor
0,222	0,110	Thiamine-triphosphatase inhibitor
0,227	0,115	Thiamine pyridinylase inhibitor
0,168	0,056	Thioredoxin reductase inhibitor
0,161	0,050	Allantoinase inhibitor
0,223	0,112	Calcium channel activator
0,203	0,092	Gamma-D-Glutamyl-meso-diaminopimelate peptidase inhibitor
0,154	0,043	Glucarate dehydratase inhibitor
0,215	0,105	Arylesterase inhibitor
0,211	0,102	Antifibrinolytic
0,129	0,020	Gentamicin 2''-nucleotidyltransferase inhibitor
0,142	0,034	DELTA24-sterol reductase inhibitor
0,171	0,062	Laminaribiose phosphorylase inhibitor
0,148	0,040	(S)-carnitine 3-dehydrogenase inhibitor

0,217	0,109	CYP2C3 substrate
0,153	0,045	Xylose isomerase inhibitor
0,258	0,151	CYP3A4 inducer
0,180	0,073	Tentoxilysin inhibitor
0,134	0,028	Phosphatidylinositol 3-kinase stimulant
0,157	0,052	Pancreatic endopeptidase E inhibitor
0,153	0,047	Antineoplastic antimetabolite
0,202	0,096	Meprin B inhibitor
0,161	0,056	N-acetylneuraminate synthase inhibitor
0,169	0,064	Calcium-sensing receptor agonist
0,158	0,053	Hepatocyte nuclear factor antagonist
0,158	0,053	Hepatocyte nuclear factor 4 alpha antagonist
0,138	0,034	Succinate dehydrogenase inhibitor
0,156	0,051	N-(5-amino-5-carboxypentanoyl)-L-cysteinyl-D-valine synthase inhibitor
0,205	0,101	Glycine amidinotransferase inhibitor
0,116	0,012	Osmotic diuretic
0,158	0,055	Alpha-N-arabinofuranosidase inhibitor
0,152	0,048	Asparagine-tRNA ligase inhibitor
0,224	0,121	Aldehyde dehydrogenase (pyrroloquinoline-quinone) inhibitor
0,137	0,034	Thermomycolin inhibitor
0,252	0,149	Lipoprotein lipase inhibitor
0,277	0,174	Caspase 3 stimulant
0,152	0,050	Acyl-lysine deacylase inhibitor
0,121	0,019	Alpha glucosidase inhibitor
0,113	0,011	Nav1.7 sodium channel blocker
0,324	0,223	Antineurotic
0,247	0,146	CYP3A inducer
0,123	0,021	High-mannose-oligosaccharide beta-1,4-N-acetylglucosaminyltransferase inhibitor
0,122	0,020	Ca2+-transporting ATPase inhibitor
0,139	0,038	4-Alpha-glucanotransferase inhibitor
0,161	0,060	Galactokinase inhibitor
0,173	0,072	Isopenicillin-N synthase inhibitor
0,259	0,158	Leukotriene-C4 synthase inhibitor
0,179	0,080	Antirickettsial
0,198	0,098	Alcohol dehydrogenase [NAD(P)+] inhibitor
0,174	0,074	Gamma-butyrobetaine dioxygenase inhibitor
0,138	0,038	Secretase stimulant

0,138	0,038	Secretase alpha stimulant
0,236	0,137	Antiinflammatory, intestinal
0,114	0,015	Alpha,alpha-trehalose phosphorylase inhibitor
0,252	0,153	Glutathione thiolesterase inhibitor
0,208	0,109	Phosphatidylcholine-sterol O-acyltransferase inhibitor
0,129	0,030	3-Deoxy-8-phosphooctulonate synthase inhibitor
0,107	0,008	Prostaglandin agonist
0,153	0,055	CDP-diacylglycerol-inositol 3-phosphatidyltransferase inhibitor
0,252	0,155	Gamma-guanidinobutyraldehyde dehydrogenase inhibitor
0,189	0,092	CYP2A3 substrate
0,226	0,129	Formate-dihydrofolate ligase inhibitor
0,188	0,091	Cyclopropane-fatty-acyl-phospholipid synthase inhibitor
0,263	0,166	Nitrate reductase (cytochrome) inhibitor
0,114	0,017	Acetylcholine M3 receptor agonist
0,118	0,022	NAD+ nucleosidase inhibitor
0,176	0,079	Vascular adhesion protein 1 inhibitor
0,151	0,056	Valine decarboxylase inhibitor
0,135	0,040	Choline dehydrogenase inhibitor
0,168	0,073	Lactate 2-monooxygenase inhibitor
0,245	0,149	Bilirubin oxidase inhibitor
0,171	0,076	4-Chlorobenzoyl-CoA dehalogenase inhibitor
0,135	0,040	Creatinase inhibitor
0,170	0,075	Glyoxylate reductase (NADP+) inhibitor
0,131	0,037	2-Dehydropantolactone reductase (A-specific) inhibitor
0,186	0,092	Ubiquitin thiolesterase inhibitor
0,105	0,011	Thromboxane A2 antagonist
0,143	0,050	Serine 3-dehydrogenase inhibitor
0,163	0,069	Choline-sulfatase inhibitor
0,108	0,015	Androgen agonist
0,143	0,050	Carboxymethylenebutenolidase inhibitor
0,233	0,140	Nitrite reductase [NAD(P)H] inhibitor
0,208	0,115	DNA-3-methyladenine glycosylase I inhibitor
0,167	0,075	N-acetylneuraminate 4-O-acetyltransferase inhibitor
0,227	0,135	4-Hydroxymandelate oxidase inhibitor
0,173	0,081	Antineoplastic (bladder cancer)
0,173	0,081	Glutamate 5-kinase inhibitor
0,122	0,031	Fructuronate reductase inhibitor

0,122	0,031	Tagaturonate reductase inhibitor
0,218	0,127	AR expression inhibitor
0,187	0,096	Antihematotoxic
0,134	0,044	D-arabinonolactone oxidase inhibitor
0,211	0,120	Leukotriene-B4 20-monooxygenase inhibitor
0,118	0,027	Homoserine O-succinyltransferase inhibitor
0,123	0,033	Beta-amylase inhibitor
0,220	0,130	Antiviral (Poxvirus)
0,143	0,052	Methionine decarboxylase inhibitor
0,129	0,039	Nicotinamide-nucleotide adenylyltransferase inhibitor
0,228	0,138	Arylsulfate sulfotransferase inhibitor
0,117	0,027	Amino-acid racemase inhibitor
0,212	0,123	Cis-1,2-dihydro-1,2-dihydroxynaphthalene dehydrogenase inhibitor
0,157	0,068	Homoserine dehydrogenase inhibitor
0,147	0,058	Dihydrouracil dehydrogenase (NAD+) inhibitor
0,127	0,038	Glutamine-pyruvate transaminase inhibitor
0,127	0,038	Rhodopsin kinase inhibitor
0,092	0,003	Corticosteroid-like
0,146	0,058	Pectate lyase inhibitor
0,120	0,031	Ribose-5-phosphate-ammonia ligase inhibitor
0,181	0,092	Serine-pyruvate transaminase inhibitor
0,110	0,022	Anticholelithogenic
0,157	0,069	Geranylgeranyl-diphosphate geranylgeranyltransferase inhibitor
0,207	0,120	Phosphatidylserine decarboxylase inhibitor
0,124	0,037	Deoxycytidine deaminase inhibitor
0,146	0,059	Antidiabetic (type 1)
0,104	0,017	Arabinogalactan endo-1,4-beta-galactosidase inhibitor
0,155	0,068	3-Carboxyethylcatechol 2,3-dioxygenase inhibitor
0,124	0,038	Lysine decarboxylase inhibitor
0,155	0,070	Methyltransferase substrate
0,154	0,069	Glutamin-(asparagin-)ase inhibitor
0,245	0,160	Mitochondrial processing peptidase inhibitor
0,125	0,040	Valine dehydrogenase (NADP+) inhibitor
0,140	0,055	Urolithiasis treatment
0,161	0,076	Sphinganine-1-phosphate aldolase inhibitor
0,214	0,130	Methylenetetrahydrofolate reductase (NADPH) inhibitor
0,259	0,175	Amine dehydrogenase inhibitor

0,127	0,042	Malonate-semialdehyde dehydrogenase inhibitor
0,273	0,188	Nicotine dehydrogenase inhibitor
0,196	0,112	Uroporphyrinogen-III synthase inhibitor
0,138	0,054	Glycosylphosphatidylinositol diacylglycerol-lyase inhibitor
0,107	0,022	Alpha,alpha-trehalose phosphorylase (configuration-retaining) inhibitor
0,102	0,018	Alpha,alpha-phosphotrehalase inhibitor
0,165	0,081	3-Hydroxy-4-oxoquinoline 2,4-dioxygenase inhibitor
0,173	0,089	CYP4A2 substrate
0,213	0,129	Cell wall biosynthesis inhibitor
0,116	0,033	Arabinose isomerase inhibitor
0,176	0,093	UGT2B10 substrate
0,095	0,012	17-Beta-hydroxysteroid dehydrogenase 1 inhibitor
0,200	0,117	Oxytocic
0,093	0,010	Glutamate (mGluR3) agonist
0,120	0,037	Isomaltulose synthase inhibitor
0,130	0,047	1-Aminocyclopropane-1-carboxylate deaminase inhibitor
0,252	0,169	Menopausal disorders treatment
0,117	0,035	Tyrosine-protein kinase TYRO 10 inhibitor
0,251	0,170	Polarisation stimulant
0,125	0,044	Deoxyribonuclease X inhibitor
0,218	0,137	Antiviral (CMV)
0,115	0,034	Glycine receptor antagonist
0,112	0,032	2-Methyleneglutarate mutase inhibitor
0,236	0,156	Antiviral (Adenovirus)
0,133	0,053	Phosphonoacetate hydrolase inhibitor
0,132	0,052	Endo-1,4-beta-xylanase inhibitor
0,146	0,066	Galactose oxidase inhibitor
0,174	0,094	2,6-Dihydroxypyridine 3-monooxygenase inhibitor
0,198	0,119	Coccolysin inhibitor
0,183	0,104	Bothrolysin inhibitor
0,190	0,111	Nardilysin inhibitor
0,163	0,085	UGT1A5 substrate
0,156	0,078	tRNA nucleotidyltransferase inhibitor
0,224	0,146	HMOX1 expression enhancer
0,154	0,076	Vomilenine glucosyltransferase inhibitor
0,166	0,088	Signal peptidase I inhibitor
0,204	0,126	Acidifying agent non gastric

0,199	0,121	Anticarcinogenic
0,116	0,038	Beta amyloid protein antagonist
0,157	0,080	Mitochondrial intermediate peptidase inhibitor
0,144	0,067	Antibacterial, ophthalmic
0,141	0,065	3-Oxoadipate enol-lactonase inhibitor
0,134	0,058	CYP19 substrate
0,109	0,033	Heme oxygenase inhibitor
0,128	0,053	Diacylglycerol kinase inhibitor
0,154	0,078	IgA-specific metalloendopeptidase inhibitor
0,137	0,061	D-lactate dehydrogenase inhibitor
0,154	0,078	Tryptophan transaminase inhibitor
0,169	0,094	2-Enoate reductase inhibitor
0,123	0,049	Fructose-2,6-bisphosphate 6-phosphatase inhibitor
0,135	0,060	Glycogen (starch) synthase inhibitor
0,151	0,077	SMN2 expression enhancer
0,277	0,204	HIF1A expression inhibitor
0,125	0,052	Antineoplastic, alkylator
0,167	0,094	CYP4F2 substrate
0,103	0,030	11-Cis-retinyl-palmitate hydrolase inhibitor
0,133	0,060	2-Oxoisovalerate dehydrogenase (acylating) inhibitor
0,113	0,041	Ketohexokinase inhibitor
0,120	0,047	Sorbose dehydrogenase inhibitor
0,173	0,101	Choleretic
0,128	0,056	Lactaldehyde reductase (NADPH) inhibitor
0,108	0,036	Phosphatidylglycerol-membrane-oligosaccharide glycerophosphotransferase inhibitor
0,221	0,150	Antiprotozoal (Coccidial)
0,119	0,047	Gluconate dehydratase inhibitor
0,189	0,117	Tankyrase inhibitor
0,218	0,147	ICAM1 expression inhibitor
0,160	0,089	N-acetyl-gamma-glutamyl-phosphate reductase inhibitor
0,128	0,057	NAD+ kinase inhibitor
0,120	0,049	Cytochrome-c3 hydrogenase inhibitor
0,109	0,038	CYP4F substrate
0,109	0,038	Beta-N-acetylgalactosaminidase inhibitor
0,108	0,038	Ribonuclease U2 inhibitor
0,202	0,133	Indanol dehydrogenase inhibitor
0,118	0,049	Aerobactin synthase inhibitor

0,214	0,145	Cytochrome-b5 reductase inhibitor
0,163	0,094	Phosphatidylglycerophosphatase inhibitor
0,125	0,056	Beta-mannosidase inhibitor
0,149	0,080	3'-Nucleotidase inhibitor
0,216	0,148	Alkane 1-monooxygenase inhibitor
0,114	0,046	2-Aminoethylphosphonate-pyruvate transaminase inhibitor
0,139	0,070	Antineoplastic (gastric cancer)
0,150	0,082	Allantoin racemase inhibitor
0,123	0,055	Glutamate release inhibitor
0,193	0,125	Antibacterial
0,159	0,091	Rhamnulose-1-phosphate aldolase inhibitor
0,157	0,090	2,4-Diaminopentanoate dehydrogenase inhibitor
0,157	0,090	3-Hydroxybutyryl-CoA dehydrogenase inhibitor
0,157	0,090	Lysine 6-dehydrogenase inhibitor
0,125	0,058	2-Aminoadipate transaminase inhibitor
0,090	0,023	Chitin deacetylase inhibitor
0,177	0,110	Hyponitrite reductase inhibitor
0,111	0,044	1,5-Anhydro-D-fructose reductase inhibitor
0,142	0,075	Dry eye syndrome treatment
0,110	0,043	2-Hydroxy-3-oxopropionate reductase inhibitor
0,137	0,070	UGT1A10 substrate
0,119	0,053	Omega-amidase inhibitor
0,110	0,044	Bis(5'-adenosyl)-triphosphatase inhibitor
0,128	0,061	UDP-N-acetylglucosamine-dolichyl-phosphate N-acetylglucosaminephosphotransferase inhibitor
0,182	0,116	Bontoxilysin inhibitor
0,165	0,099	Phenylalanine(histidine) transaminase inhibitor
0,142	0,076	UGT2B18 substrate
0,125	0,060	Fructose-2,6-bisphosphate 2-phosphatase inhibitor
0,096	0,030	2-Dehydro-3-deoxy-phosphogluconate aldolase inhibitor
0,112	0,047	Glycerophosphocholine cholinephosphodiesterase inhibitor
0,178	0,113	Carnitinamidase inhibitor
0,158	0,093	Dolichol kinase inhibitor
0,076	0,011	Microtubule stabilization
0,085	0,020	NMDA receptor polyamine site agonist
0,101	0,036	L-fuconate dehydratase inhibitor
0,136	0,072	Iron-cytochrome-c reductase inhibitor
0,145	0,081	Melanin inhibitor

0,133	0,069	Diisopropyl-fluorophosphatase inhibitor
0,178	0,114	Methanol dehydrogenase inhibitor
0,286	0,222	TP53 expression enhancer
0,106	0,042	Gluconokinase inhibitor
0,145	0,082	Granzyme A inhibitor
0,094	0,031	(S)-2-Methylmalate dehydratase inhibitor
0,113	0,049	Homocysteine desulfhydrase inhibitor
0,106	0,043	Proline dehydrogenase inhibitor
0,138	0,076	Phosphoinositide phospholipase C inhibitor
0,069	0,006	Protein phosphatase 2A inhibitor
0,117	0,055	Lysozyme inhibitor
0,136	0,074	Cellulase inhibitor
0,210	0,149	DNA-(apurinic or apyrimidinic site) lyase inhibitor
0,203	0,141	4-Nitrophenylphosphatase inhibitor
0,107	0,046	Dichloromuconate cycloisomerase inhibitor
0,202	0,141	Myosin ATPase inhibitor
0,094	0,034	Alpha-L-fucosidase inhibitor
0,131	0,070	Allophanate hydrolase inhibitor
0,130	0,070	Selenocysteine lyase inhibitor
0,163	0,103	Gallate decarboxylase inhibitor
0,136	0,076	Cerebroside-sulfatase inhibitor
0,212	0,152	Venom exonuclease inhibitor
0,161	0,101	Metallocarboxypeptidase D inhibitor
0,147	0,088	Pyruvate dehydrogenase (cytochrome) inhibitor
0,147	0,088	Trans-2-enoyl-CoA reductase (NAD+) inhibitor
0,089	0,030	CYP4F12 substrate
0,212	0,152	Urethanase inhibitor
0,135	0,076	GABA inverse agonist
0,135	0,076	GABA A inverse agonist
0,101	0,042	3-Oxosteroid 1-dehydrogenase inhibitor
0,109	0,050	Globoside alpha-N-acetylgalactosaminyltransferase inhibitor
0,113	0,054	NMDA receptor polyamine site antagonist
0,066	0,007	Antimitotic, Podophyllotoxin-like
0,146	0,088	N6-methyl-lysine oxidase inhibitor
0,064	0,006	Dipeptidyl peptidase IV inhibitor
0,102	0,044	2-Dehydro-3-deoxy-L-arabinonate dehydratase inhibitor
0,128	0,070	Haloacetate dehalogenase inhibitor

0,128	0,070	Prepilin peptidase inhibitor
0,085	0,027	Oxaloacetate tautomerase inhibitor
0,104	0,046	Beta-D-fucosidase inhibitor
0,128	0,070	Agmatinase inhibitor
0,139	0,081	Gly-X carboxypeptidase inhibitor
0,083	0,026	Deoxyhypusine synthase inhibitor
0,082	0,025	3-Alpha-hydroxysteroid dehydrogenase (B-specific) inhibitor
0,094	0,037	Epoxide hydrolase substrate
0,091	0,034	Alpha-1,6-mannosyl-glycoprotein 6-beta-N-acetylglucosaminyltransferase inhibitor
0,135	0,078	Lipotropic
0,155	0,098	Homoaconitate hydratase inhibitor
0,155	0,098	6-Carboxyhexanoate-CoA ligase inhibitor
0,155	0,098	Biotin-CoA ligase inhibitor
0,155	0,098	Triacetate-lactonase inhibitor
0,091	0,034	Alpha-L-rhamnosidase inhibitor
0,067	0,011	11-Beta-hydroxysteroid dehydrogenase inhibitor
0,121	0,065	Poly(ADP-ribose) glycohydrolase inhibitor
0,126	0,070	CYP26A substrate
0,132	0,076	Valine-tRNA ligase inhibitor
0,188	0,132	UGT2B4 substrate
0,110	0,054	Poly(3-hydroxybutyrate) depolymerase inhibitor
0,271	0,215	2-Hydroxyquinoline 8-monooxygenase inhibitor
0,115	0,060	Guanidinodeoxy-scyllo-inositol-4-phosphatase inhibitor
0,157	0,102	Camphor 1,2-monooxygenase inhibitor
0,128	0,073	Galactose 1-dehydrogenase (NADP+) inhibitor
0,128	0,073	Beta-alanine-pyruvate transaminase inhibitor
0,147	0,092	UGT2B9 substrate
0,111	0,056	Heparan-alpha-glucosaminide N-acetyltransferase inhibitor
0,133	0,078	CYP4B substrate
0,148	0,094	Carnosine synthase inhibitor
0,121	0,066	Mevalonate kinase inhibitor
0,217	0,163	Analgesic stimulant
0,095	0,042	Galactosylgalactosylglucosylceramidase inhibitor
0,062	0,009	CYP4F8 substrate
0,067	0,013	Actin depolymerization stimulant
0,112	0,059	Galactoside 2-alpha-L-fucosyltransferase inhibitor
0,077	0,023	ATP diphosphatase inhibitor

0,200	0,147	Cyclic AMP modulator
0,070	0,017	Sucrose-phosphate phosphatase inhibitor
0,111	0,059	Chondroitin 6-sulfotransferase inhibitor
0,138	0,085	3-Aminobutyryl-CoA ammonia-lyase inhibitor
0,248	0,196	Spermidine dehydrogenase inhibitor
0,071	0,019	Amylosucrase inhibitor
0,064	0,012	11-Beta-hydroxysteroid dehydrogenase 1 inhibitor
0,138	0,086	Penicillopepsin inhibitor
0,098	0,046	Contraceptive male
0,094	0,041	Homospermidine synthase inhibitor
0,174	0,122	Acyl-CoA oxidase inhibitor
0,165	0,113	Alanine-tRNA ligase inhibitor
0,142	0,090	Tetrahydroxynaphthalene reductase inhibitor
0,114	0,063	UGT2B11 substrate
0,079	0,028	Alpha-mannosidase inhibitor
0,125	0,074	Biotin carboxylase inhibitor
0,133	0,083	Aldehyde ferredoxin oxidoreductase inhibitor
0,118	0,068	ATP adenylyltransferase inhibitor
0,179	0,128	Peroxidase substrate
0,137	0,087	2-Acylglycerol O-acyltransferase inhibitor
0,096	0,045	3-Isopropylmalate dehydrogenase inhibitor
0,110	0,060	Pantothenase inhibitor
0,125	0,075	CYP4B1 substrate
0,103	0,053	Cell wall synthesis inhibitor
0,129	0,079	Alpha-Methylacyl-CoA racemase inhibitor
0,124	0,075	8-Amino-7-oxononanoate synthase inhibitor
0,173	0,123	Retinal dehydrogenase inhibitor
0,192	0,143	X-methyl-His dipeptidase inhibitor
0,122	0,073	Excitatory amino acid transporter EAAC1 inhibitor
0,104	0,055	Glyceraldehyde-3-phosphate dehydrogenase (NADP+) inhibitor
0,175	0,126	Histidinol dehydrogenase inhibitor
0,087	0,038	Xylan 1,4-beta-xylosidase inhibitor
0,241	0,193	CYP19A1 expression inhibitor
0,124	0,076	Thiamine-phosphate kinase inhibitor
0,161	0,113	Urate-ribonucleotide phosphorylase inhibitor
0,161	0,113	Nicotinic alpha4beta2 receptor antagonist
0,121	0,073	O-acetylhomoserine aminocarboxypropyltransferase inhibitor

0,152	0,105	Aldehyde dehydrogenase 2 substrate
0,081	0,034	Peptidoglycan beta-N-acetylmuramidase inhibitor
0,085	0,038	Steroid sulfotransferase inhibitor
0,073	0,027	1-Alkyl-2-acetylglycerol O-acyltransferase inhibitor
0,119	0,073	NAD(P)+ transhydrogenase (B-specific) inhibitor
0,107	0,061	Leucine transaminase inhibitor
0,144	0,098	6-Pyruvoyltetrahydropterin synthase inhibitor
0,116	0,070	Glycoprotein 3-alpha-L-fucosyltransferase inhibitor
0,084	0,038	Gynecological disorders treatment
0,080	0,034	CYP2A13 substrate
0,132	0,087	Chitin synthase inhibitor
0,099	0,054	Phosphofructokinase-1 inhibitor
0,103	0,058	D-glutamate oxidase inhibitor
0,123	0,078	Aminocarboxymuconate-semialdehyde decarboxylase inhibitor
0,124	0,079	Phenylpyruvate decarboxylase inhibitor
0,223	0,178	Antineoplastic (solid tumors)
0,150	0,105	Penicillin amidase inhibitor
0,136	0,092	Glucosyl transferase inhibitor
0,141	0,097	Lipoxygenase substrate
0,136	0,091	Lombricine kinase inhibitor
0,099	0,055	Monocarboxylic acid transporter 1 inhibitor
0,099	0,055	Monocarboxylic acid transporter inhibitor
0,091	0,046	[hydroxymethylglutaryl-CoA reductase (NADPH)] kinase inhibitor
0,167	0,122	Nucleoside oxidase (H2O2-forming) inhibitor
0,115	0,071	Neuropsin inhibitor
0,116	0,072	Isovaleryl-CoA dehydrogenase inhibitor
0,134	0,089	Phenylacetate-CoA ligase inhibitor
0,091	0,047	Phosphoenolpyruvate carboxykinase (diphosphate) inhibitor
0,122	0,078	(S)-2-hydroxy-acid oxidase inhibitor
0,250	0,206	Antihypoxic
0,144	0,100	4-Hydroxyglutamate transaminase inhibitor
0,093	0,049	Cysteine transaminase inhibitor
0,143	0,100	Creatinine deaminase inhibitor
0,149	0,106	Glutathione peroxidase inhibitor
0,083	0,040	mRNA (guanine-N7-)-methyltransferase inhibitor
0,228	0,185	Antihelmintic (Nematodes)
0,064	0,021	Biotin-[methylmalonyl-CoA-carboxytransferase] ligase inhibitor

0,082	0,039	4a-Hydroxytetrahydrobiopterin dehydratase inhibitor
0,077	0,034	Aldose 1-epimerase inhibitor
0,145	0,103	Acaricide
0,117	0,075	Glucan endo-1,3-alpha-glucosidase inhibitor
0,116	0,073	DNA directed DNA polymerase inhibitor
0,225	0,184	CYP3A3 substrate
0,100	0,058	Isocitrate dehydrogenase (NAD+) inhibitor
0,178	0,136	Aromatic-hydroxylamine O-acetyltransferase inhibitor
0,150	0,109	Antihelmintic (Fasciola)
0,071	0,029	CYP24 inhibitor
0,138	0,097	Pyridoxine 4-oxidase inhibitor
0,138	0,096	Allyl-alcohol dehydrogenase inhibitor
0,089	0,047	Ribulose-bisphosphate carboxylase inhibitor
0,144	0,103	Adenylate cyclase stimulant
0,114	0,073	Iduronate-2-sulfatase inhibitor
0,087	0,046	Malate dehydrogenase (NADP+) inhibitor
0,168	0,127	Antihypotensive
0,102	0,062	Leucine-tRNA ligase inhibitor
0,109	0,069	L-lysine 6-transaminase inhibitor
0,140	0,100	2,4-Dichlorophenol 6-monooxygenase inhibitor
0,103	0,063	Aspartate-ammonia ligase (ADP-forming) inhibitor
0,137	0,097	Aspartoacylase inhibitor
0,079	0,039	Aspartate 1-decarboxylase inhibitor
0,256	0,216	Preneoplastic conditions treatment
0,079	0,039	Lactate-malate transhydrogenase inhibitor
0,144	0,105	Acetylornithine deacetylase inhibitor
0,065	0,026	Glucan 1,4-alpha-glucosidase inhibitor
0,160	0,121	Sulfite reductase inhibitor
0,051	0,013	Sucrose phosphorylase inhibitor
0,064	0,025	Prostaglandin-I synthase inhibitor
0,113	0,074	N4-(beta-N-acetylglucosaminyl)-L-asparaginase inhibitor
0,089	0,051	Cellobiose phosphorylase inhibitor
0,212	0,174	Tyrosine 3 hydroxylase inhibitor
0,065	0,027	UGT2B4E substrate
0,065	0,027	UGT2B23 substrate
0,065	0,027	UGT2B19 substrate
0,065	0,027	UGT2B30 substrate

0,065	0,027	UGT2B4D substrate
0,100	0,061	Adenylylsulphatase inhibitor
0,088	0,050	L-lysine oxidase inhibitor
0,168	0,130	Diamine N-acetyltransferase inhibitor
0,158	0,120	Dihydroxy-acid dehydratase inhibitor
0,111	0,073	D-2-hydroxy-acid dehydrogenase inhibitor
0,089	0,051	Retinol O-fatty-acyltransferase inhibitor
0,053	0,016	Dipeptidyl peptidase II inhibitor
0,079	0,042	CYP2C2 substrate
0,063	0,026	Aromatase inhibitor
0,146	0,109	Hydroxymethylglutaryl-CoA lyase inhibitor
0,146	0,110	Glycine C-acetyltransferase inhibitor
0,101	0,065	Aminoacylase inhibitor
0,112	0,076	Anabolic
0,109	0,073	Ribonuclease inhibitor
0,173	0,137	Prolyl aminopeptidase inhibitor
0,055	0,019	Methylaspartate mutase inhibitor
0,127	0,091	Aldehyde dehydrogenase (NADP+) inhibitor
0,188	0,153	Gingipain K inhibitor
0,082	0,047	Cushing's syndrome treatment
0,135	0,100	Protein synthesis inhibitor
0,061	0,026	Dipeptidyl peptidase inhibitor
0,063	0,028	Glucan 1,3-alpha-glucosidase inhibitor
0,184	0,149	Glutaminyl-peptide cyclotransferase inhibitor
0,075	0,041	3-Dehydroquinate synthase inhibitor
0,059	0,025	CYP17 substrate
0,114	0,080	Pantoate 4-dehydrogenase inhibitor
0,120	0,086	2-Oxoglutarate decarboxylase inhibitor
0,078	0,044	Glutaminyl-tRNA synthase (glutamine-hydrolysing) inhibitor
0,091	0,057	UDP-N-acetylglucosamine diphosphorylase inhibitor
0,160	0,126	Opine dehydrogenase inhibitor
0,071	0,037	[acetyl-CoA carboxylase] kinase inhibitor
0,258	0,224	Sulfur reductase inhibitor
0,056	0,022	D(-)-tartrate dehydratase inhibitor
0,085	0,051	2-Methylcitrate synthase inhibitor
0,063	0,030	D-lactate-2-sulfatase inhibitor
0,138	0,105	CDP-4-dehydro-6-deoxyglucose reductase inhibitor

0,153	0,120	Indoleacetaldoxime dehydratase inhibitor
0,052	0,018	Orotidylate decarboxylase inhibitor
0,053	0,019	NADPH oxidoreductase inhibitor
0,092	0,059	Galactosylceramide sulfotransferase inhibitor
0,114	0,081	Glutathione S-transferase substrate
0,083	0,050	Argininosuccinate lyase inhibitor
0,104	0,072	L-galactonolactone oxidase inhibitor
0,105	0,073	Aldehyde dehydrogenase [NAD(P)+] inhibitor
0,139	0,107	Polynucleotide 5'-hydroxy-kinase inhibitor
0,085	0,053	Deoxycytidine kinase inhibitor
0,103	0,072	Pyrimidine-deoxynucleoside 2'-dioxygenase inhibitor
0,108	0,076	NADH dehydrogenase inhibitor
0,089	0,057	Squalene-hopene cyclase inhibitor
0,059	0,028	Na+ K+ transporting ATPase inhibitor
0,088	0,057	5-Oxoprolinase (ATP-hydrolysing) inhibitor
0,106	0,075	D-lactate dehydrogenase (cytochrome) inhibitor
0,102	0,071	Cholate-CoA ligase inhibitor
0,087	0,056	Saccharopine dehydrogenase (NAD+, L-glutamate-forming) inhibitor
0,061	0,030	3'(2'),5'-Bisphosphate nucleotidase inhibitor
0,138	0,107	Cytochrome-b5 reductase substrate
0,134	0,104	Histamine agonist
0,049	0,018	Tartrate decarboxylase inhibitor
0,035	0,004	Prostaglandin EP2 agonist
0,097	0,067	tRNA (cytosine-5-)-methyltransferase inhibitor
0,090	0,059	D-proline reductase (dithiol) inhibitor
0,124	0,094	HIV-2 reverse transcriptase inhibitor
0,153	0,123	Antineoplastic (thyroid cancer)
0,065	0,035	Mannosidase inhibitor
0,064	0,034	Acidifying agent gastric
0,136	0,107	Benzoylformate decarboxylase inhibitor
0,167	0,137	Ferredoxin hydrogenase inhibitor
0,074	0,045	3(or 17)alpha-hydroxysteroid dehydrogenase inhibitor
0,072	0,042	L-lactate dehydrogenase (cytochrome) inhibitor
0,148	0,119	Stromelysin 2 inhibitor
0,072	0,043	Lactaldehyde dehydrogenase inhibitor
0,128	0,099	Oligopeptidase B inhibitor
0,137	0,108	N-methyl-2-oxoglutaramate hydrolase inhibitor

0,094	0,066	Aspartate transaminase inhibitor
0,109	0,081	Carnitine 3-dehydrogenase inhibitor
0,066	0,038	Androgen antagonist
0,107	0,078	CYP19 inhibitor
0,106	0,078	Aspartate kinase inhibitor
0,139	0,111	Inorganic diphosphatase inhibitor
0,087	0,059	Deoxyribonuclease (pyrimidine dimer) inhibitor
0,058	0,031	CYP24 substrate
0,125	0,098	Aminopeptidase Y inhibitor
0,082	0,054	Alpha,alpha-trehalose-phosphate synthase (UDP-forming) inhibitor
0,073	0,046	4-Hydroxy-4-methyl-2-oxoglutarate aldolase inhibitor
0,134	0,107	Acetate kinase inhibitor
0,101	0,074	Diacylglycerol cholinephosphotransferase inhibitor
0,062	0,035	Prolyl endopeptidase inhibitor
0,075	0,049	3-Dehydroquinate dehydratase inhibitor
0,076	0,050	Glycerophosphocholine phosphodiesterase inhibitor
0,060	0,033	D-Serine ammonia-lyase inhibitor
0,075	0,049	ATP deaminase inhibitor
0,113	0,087	Ethanolaminephosphotransferase inhibitor
0,125	0,100	Peptide-tryptophan 2,3-dioxygenase inhibitor
0,076	0,051	Aspartate-semialdehyde dehydrogenase inhibitor
0,078	0,053	Cyanate hydratase inhibitor
0,083	0,058	Scyllo-inosamine-4-phosphate amidinotransferase inhibitor
0,106	0,081	Sulfate adenylyltransferase (ADP) inhibitor
0,124	0,099	Ferrochelatase inhibitor
0,100	0,075	Glycerol-3-phosphate O-acyltransferase inhibitor
0,178	0,153	Sigma receptor agonist
0,091	0,066	Mannonate dehydratase inhibitor
0,099	0,074	DNA-3-methyladenine glycosylase II inhibitor
0,160	0,136	Indolepyruvate C-methyltransferase inhibitor
0,176	0,152	Acetylserotonin O-methyltransferase inhibitor
0,122	0,098	Peptidylamidoglycolate lyase inhibitor
0,103	0,079	Cysteine desulfurase inhibitor
0,047	0,023	Electron transport complex I inhibitor
0,180	0,156	CYP2B2 substrate
0,124	0,100	Plasmanylethanolamine desaturase inhibitor
0,058	0,035	Hydroxylysine kinase inhibitor

0,027	0,005	NADH oxidase inhibitor
0,138	0,116	3-Demethylubiquinone-9 3-O-methyltransferase inhibitor
0,045	0,022	Glucocorticoid antagonist
0,077	0,054	Polyamine biosynthesis inhibitor
0,058	0,036	Biotin-[propionyl-CoA-carboxylase (ATP-hydrolysing)] ligase inhibitor
0,094	0,072	Allantoicase inhibitor
0,083	0,061	Inositol-polyphosphate 5-phosphatase inhibitor
0,054	0,032	Propionate CoA-transferase inhibitor
0,248	0,226	Insulin promoter
0,069	0,048	Glutathione reductase stimulant
0,069	0,048	Glutathione reductase (NADPH) stimulant
0,234	0,213	N-hydroxyarylamine O-acetyltransferase inhibitor
0,066	0,045	Dipeptidyl peptidase I inhibitor
0,078	0,057	2-Oxoaldehyde dehydrogenase (NAD+) inhibitor
0,084	0,063	CYP26 substrate
0,108	0,087	5 Hydroxytryptamine 4A antagonist
0,180	0,159	Methane monooxygenase inhibitor
0,071	0,051	6-Phosphofructo-2-kinase inhibitor
0,126	0,105	Brachyurin inhibitor
0,060	0,040	Deoxyadenosine kinase inhibitor
0,195	0,175	FMO1 substrate
0,037	0,017	Homoisocitrate dehydrogenase inhibitor
0,058	0,038	Glycine N-choloyltransferase inhibitor
0,083	0,064	UDP-N-acetylglucosamine 2-epimerase inhibitor
0,092	0,073	Dihydrouracil oxidase inhibitor
0,056	0,037	CMP-KDO synthase inhibitor
0,095	0,075	Glutamate N-acetyltransferase inhibitor
0,131	0,112	Arylacetonitrilase inhibitor
0,036	0,017	Transaldolase inhibitor
0,141	0,122	Guanidinoacetate kinase inhibitor
0,175	0,157	Cathepsin T inhibitor
0,206	0,187	4-Hydroxyproline epimerase inhibitor
0,169	0,151	Endopeptidase La inhibitor
0,089	0,071	Thiamine-phosphate diphosphorylase inhibitor
0,092	0,074	UDP-glucose 4-epimerase inhibitor
0,093	0,075	Chloramphenicol O-acetyltransferase inhibitor
0,072	0,054	Phosphoribulokinase inhibitor

0,072	0,054	N-acetylglucosaminyldiphosphoundecaprenol N-acetyl-beta-D-mannosaminyltransferase inhibitor
0,096	0,078	Site-specific DNA-methyltransferase (adenine-specific) inhibitor
0,071	0,054	Dihydrolipoamide S-acetyltransferase inhibitor
0,059	0,042	Acetylcholine muscarinic agonist
0,060	0,042	Alpha-1,6-mannosyl-glycoprotein 2-beta-N-acetylglucosaminyltransferase inhibitor
0,112	0,094	Cathepsin H inhibitor
0,066	0,048	Corticotropin releasing factor antagonist
0,044	0,026	Androst-4-ene-3,17-dione monooxygenase inhibitor
0,090	0,073	Cytosole dipeptidase inhibitor
0,111	0,094	Sulfur dioxygenase inhibitor
0,109	0,092	Protein-tyrosine sulfotransferase inhibitor
0,030	0,014	Phenylethanolamine N methyltransferase inhibitor
0,129	0,113	Hydroxymethylbilane synthase inhibitor
0,114	0,098	Endoglycosylceramidase inhibitor
0,123	0,107	3,4-Dihydroxy-9,10-secoandrosta-1,3,5(10)-triene-9,17-dione 4,5-dioxygenase inhibitor
0,051	0,035	HCV NS3/NS4A protease inhibitor
0,068	0,052	UGT2B7Y substrate
0,068	0,052	UGT2B7H substrate
0,129	0,113	Restless leg syndrome treatment
0,132	0,116	CYP2D1 substrate
0,094	0,078	Glycoprotein-fucosylgalactoside alpha-N-acetylgalactosaminyltransferase inhibitor
0,119	0,103	(R,R)-butanediol dehydrogenase inhibitor
0,048	0,032	Thymidine kinase 1 inhibitor
0,071	0,056	Hydroperoxide dehydratase inhibitor
0,074	0,059	S-methyl-5-thioribose kinase inhibitor
0,085	0,070	Benzaldehyde dehydrogenase (NAD+) inhibitor
0,081	0,066	Acetoacetate decarboxylase inhibitor
0,215	0,200	Retinoic acid metabolism inhibitor
0,084	0,069	Necroptosis inhibitor
0,115	0,100	Cysteine synthase inhibitor
0,085	0,070	Glycerol dehydratase inhibitor
0,079	0,064	Guanidinopropionase inhibitor
0,048	0,034	CYP3A11 substrate
0,112	0,098	Antitussive, narcotic
0,089	0,075	Ribitol 2-dehydrogenase inhibitor
0,076	0,062	Cystathionine beta-lyase inhibitor

0,063	0,049	Glycerol kinase inhibitor
0,095	0,081	Malyl-CoA lyase inhibitor
0,067	0,053	L-aminoadipate-semialdehyde dehydrogenase inhibitor
0,095	0,081	Glucose 1-dehydrogenase inhibitor
0,121	0,107	Adenylate kinase inhibitor
0,119	0,105	N-Acyl-D-amino-acid deacylase inhibitor
0,069	0,055	Serine-tRNA ligase inhibitor
0,104	0,090	Contraceptive
0,066	0,052	Glutamate (mGluR8) agonist
0,105	0,091	Transcription factor NF kappa B inhibitor
0,060	0,047	Thioether S-methyltransferase inhibitor
0,115	0,102	Candidapepsin inhibitor
0,111	0,098	Cancer procoagulant inhibitor
0,054	0,041	Assemblin inhibitor
0,029	0,016	Hexokinase stimulant
0,058	0,046	Quinate 5-dehydrogenase inhibitor
0,150	0,138	Aminomuconate-semialdehyde dehydrogenase inhibitor
0,086	0,074	Phosphatidate cytidylyltransferase inhibitor
0,218	0,206	Loop diuretic
0,132	0,120	Tyrosine kinase stimulant
0,035	0,024	Membrane-oligosaccharide glycerophosphotransferase inhibitor
0,075	0,064	(R)-aminopropanol dehydrogenase inhibitor
0,121	0,109	(S)-3-hydroxyacid ester dehydrogenase inhibitor
0,132	0,121	Threonine aldolase inhibitor
0,019	0,008	Glutamate (mGluR2) antagonist
0,217	0,206	Superoxide dismutase inhibitor
0,088	0,077	Glycerol-3-phosphate dehydrogenase (NAD+) inhibitor
0,112	0,101	Galactoside O-acetyltransferase inhibitor
0,090	0,080	Adenosylmethionine-8-amino-7-oxononanoate transaminase inhibitor
0,105	0,094	Pyruvate dehydrogenase inhibitor
0,082	0,072	Alpha,alpha-trehalase inhibitor
0,062	0,052	Formimidoylglutamase inhibitor
0,070	0,060	N-acetylglucosamine-6-phosphate deacetylase inhibitor
0,074	0,064	Neolactotetraosylceramide alpha-2,3-sialyltransferase inhibitor
0,038	0,028	Dichloromethane dehalogenase inhibitor
0,063	0,053	UDP-glucose 6-dehydrogenase inhibitor
0,038	0,028	Isopentenyl-diphosphate DELTA-isomerase inhibitor

0,019	0,010	Gestagen-like
0,117	0,108	Liver X receptor antagonist
0,045	0,036	N-acetylglucosaminyldiphosphodolichol N-acetylglucosaminyltransferase inhibitor
0,187	0,178	Chloride channel activator
0,085	0,076	Triose-phosphate isomerase inhibitor
0,181	0,171	Monodehydroascorbate reductase (NADH) inhibitor
0,098	0,089	Decylcitrate synthase inhibitor
0,036	0,028	Phosphoglycerate phosphatase inhibitor
0,187	0,178	NADPH-ferrihemoprotein reductase inhibitor
0,058	0,050	Beta-glucosidase inhibitor
0,172	0,164	L-threonine 3-dehydrogenase inhibitor
0,165	0,157	Prostaglandin-E2 9-reductase inhibitor
0,085	0,077	Malate dehydrogenase (oxaloacetate-decarboxylating) inhibitor
0,166	0,158	Immunostimulant
0,074	0,066	Succinate-hydroxymethylglutarate CoA-transferase inhibitor
0,092	0,085	Aspartyl aminopeptidase inhibitor
0,031	0,023	Nociceptin (N/OFQ) receptor antagonist
0,107	0,100	Acylglycerone-phosphate reductase inhibitor
0,112	0,104	3-Hydroxyphenylacetate 6-hydroxylase inhibitor
0,097	0,090	Squalene epoxidase inhibitor
0,016	0,009	Ether-a-go-go potassium channel blocker
0,016	0,009	Ether-a-go-go potassium channel 1 blocker
0,081	0,074	Astringent
0,101	0,094	Skin whitener
0,115	0,109	Tryptophanyl aminopeptidase inhibitor
0,026	0,020	Alpha glucosidase I inhibitor
0,022	0,015	11-Beta-hydroxysteroid dehydrogenase 2 inhibitor
0,033	0,026	Glucose-6-phosphate isomerase inhibitor
0,091	0,084	Cerevisin inhibitor
0,123	0,116	Pyruvate carboxylase inhibitor
0,152	0,146	Pitrilysin inhibitor
0,144	0,138	Thermitase inhibitor
0,080	0,073	CTP synthase inhibitor
0,061	0,055	Isocitrate dehydrogenase (NADP+) inhibitor
0,088	0,082	Alanine transaminase inhibitor
0,047	0,041	Prostacyclin agonist
0,089	0,083	Diiodophenylpyruvate reductase inhibitor

0,066	0,060	Maleate hydratase inhibitor
0,172	0,166	ATP phosphoribosyltransferase inhibitor
0,077	0,071	D-malate dehydrogenase (decarboxylating) inhibitor
0,107	0,101	Gingipain R inhibitor
0,054	0,048	Plus-end-directed kinesin ATPase inhibitor
0,049	0,044	Beta galactosidase inhibitor
0,040	0,035	Thymidine-triphosphatase inhibitor
0,031	0,026	Sodium/glucose cotransporter 1 inhibitor
0,027	0,022	Phosphopyruvate hydratase inhibitor
0,089	0,084	D-Ornithine 4,5-aminomutase inhibitor
0,049	0,045	Uracil phosphoribosyltransferase inhibitor
0,105	0,101	Inositol 1,4,5-trisphosphate 3-kinase inhibitor
0,072	0,068	tRNA (guanine-N2-)-methyltransferase inhibitor
0,014	0,010	Kanamycin kinase inhibitor
0,135	0,131	Cyclopentanone monooxygenase inhibitor
0,019	0,015	Glutamate (mGluR3) antagonist
0,057	0,054	Beta-1,4-mannosyl-glycoprotein 4-beta-N-acetylglucosaminyltransferase inhibitor
0,073	0,069	CMP-N-acylneuraminate phosphodiesterase inhibitor
0,046	0,043	Anhydrotetracycline monooxygenase inhibitor
0,075	0,071	SULT2A1 substrate
0,072	0,068	Muconate cycloisomerase inhibitor
0,091	0,088	Carboxylesterase inhibitor
0,016	0,012	Antiprotozoal activity enhancer
0,050	0,047	Carbamoyl-serine ammonia-lyase inhibitor
0,074	0,072	Diguanidinobutanase inhibitor
0,069	0,066	ACK1 kinase inhibitor
0,087	0,084	Ornithine-oxo-acid transaminase inhibitor
0,109	0,107	Homogentisate 1,2-dioxygenase inhibitor
0,012	0,009	Actin polymerization inhibitor
0,033	0,031	Endo-beta-N-acetylglucosaminidase inhibitor
0,068	0,065	Ribonucleoside diphosphate reductase inhibitor
0,105	0,102	Arylalkyl acylamidase inhibitor
0,091	0,089	D-cysteine desulfhydrase inhibitor
0,067	0,065	Oligo-1,6-glucosidase inhibitor
0,092	0,090	Glucosamine-1-phosphate N-acetyltransferase inhibitor
0,140	0,138	CTGF expression inhibitor
0,095	0,093	Glucosamine-6-phosphate deaminase inhibitor

0,038	0,036	2-Ethylmalate synthase inhibitor
0,071	0,070	[myelin basic protein]-arginine N-methyltransferase inhibitor
0,123	0,122	UGT2B7 substrate
0,097	0,095	CYP3C substrate
0,066	0,065	D-alanine transaminase inhibitor
0,123	0,122	Tryptophan alpha,beta-oxidase inhibitor
0,126	0,125	Heat shock protein agonist
0,020	0,019	Cortisone alpha-reductase inhibitor
0,023	0,022	Tartrate dehydrogenase inhibitor
0,109	0,108	Acid-sensing ion channel blocker
0,088	0,088	2-Nitrophenol 2-monooxygenase inhibitor
0,079	0,079	Capillary fragility treatment
0,125	0,125	4-Chlorophenylacetate 3,4-dioxygenase inhibitor
0,116	0,115	4-Hydroxybenzoate nonaprenyltransferase inhibitor
0,123	0,123	Picornain 3C inhibitor
0,047	0,047	Glutamate (mGluR1) agonist
0,195	0,194	Farnesoid X receptor antagonist
0,057	0,057	GMP synthase inhibitor

Complex 5 All

All Pa>Pi Pa>0,3 Pa>0,7

Pa	Pi	Activity
0,847	0,018	Phobic disorders treatment
0,829	0,009	Antineoplastic
0,832	0,018	Testosterone 17beta-dehydrogenase (NADP+) inhibitor
0,816	0,032	CYP2C12 substrate
0,786	0,015	Acylcarnitine hydrolase inhibitor
0,790	0,023	Saccharopepsin inhibitor
0,790	0,023	Chymosin inhibitor
0,790	0,023	Acrocylindropepsin inhibitor
0,774	0,024	Antiseborrheic
0,754	0,014	Alkylacetylglycerophosphatase inhibitor
0,757	0,024	Alkenylglycerophosphocholine hydrolase inhibitor

0,745	0,013	JAK2 expression inhibitor
0,733	0,003	Antineoplastic (non-Hodgkin's lymphoma)
0,746	0,018	Beta-adrenergic receptor kinase inhibitor
0,746	0,018	G-protein-coupled receptor kinase inhibitor
0,753	0,030	Antieczematic
0,727	0,004	Imidazoline receptor agonist
0,764	0,044	Aspulvinone dimethylallyltransferase inhibitor
0,730	0,018	Lysase inhibitor
0,717	0,008	Glucan 1,4-alpha-maltotriohydrolase inhibitor
0,744	0,036	CYP2J substrate
0,702	0,013	27-Hydroxycholesterol 7alpha-monooxygenase inhibitor
0,729	0,058	Ubiquinol-cytochrome-c reductase inhibitor
0,703	0,032	CYP2J2 substrate
0,701	0,034	Nicotinic alpha6beta3beta4alpha5 receptor antagonist
0,679	0,012	Vasoprotector
0,665	0,008	CYP2A4 substrate
0,686	0,036	Sugar-phosphatase inhibitor
0,673	0,029	5-O-(4-coumaroyl)-D-quinate 3'-monooxygenase inhibitor
0,667	0,024	HIF1A expression inhibitor
0,637	0,003	Dementia treatment
0,692	0,060	Mucomembranous protector
0,655	0,024	Phosphatase inhibitor
0,637	0,011	Cardiovascular analeptic
0,633	0,008	Dolichyl-diphosphooligosaccharide-protein glycotransferase inhibitor
0,628	0,005	Antineoplastic (ovarian cancer)
0,625	0,009	H+-exporting ATPase inhibitor
0,651	0,036	TP53 expression enhancer
0,668	0,062	CDP-glycerol glycerophosphotransferase inhibitor
0,637	0,032	Ribulose-phosphate 3-epimerase inhibitor
0,619	0,021	Respiratory analeptic
0,620	0,023	CYP3A2 substrate
0,654	0,060	Membrane permeability inhibitor
0,646	0,054	Polyporopepsin inhibitor
0,601	0,011	Platelet adhesion inhibitor
0,601	0,012	Carminative
0,615	0,035	Antidyskinetic
0,606	0,026	Apoptosis agonist

0,598	0,022	Vasodilator, peripheral
0,596	0,020	CYP3A1 substrate
0,588	0,014	CYP3A3 substrate
0,598	0,025	CYP3A5 substrate
0,632	0,061	Antineurotic
0,601	0,030	Cytoprotectant
0,593	0,025	Acetylcholine neuromuscular blocking agent
0,582	0,016	Neurotransmitter antagonist
0,579	0,015	Antiviral (Influenza)
0,572	0,008	Antimetastatic
0,623	0,059	CYP2H substrate
0,608	0,046	Nicotinic alpha2beta2 receptor antagonist
0,570	0,013	Antineoplastic (breast cancer)
0,584	0,027	Membrane integrity antagonist
0,574	0,021	CYP3A4 inducer
0,557	0,007	Imidazoline I1 receptor agonist
0,571	0,025	Ovulation inhibitor
0,577	0,033	Glucan endo-1,3-beta-D-glucosidase inhibitor
0,565	0,021	CYP3A inducer
0,552	0,013	Peptidoglycan glycosyltransferase inhibitor
0,556	0,021	CYP2A2 substrate
0,563	0,028	Antiviral (Picornavirus)
0,543	0,009	Antineoplastic (solid tumors)
0,556	0,022	CYP2A1 substrate
0,555	0,021	Antipruritic, allergic
0,565	0,036	Gastrin inhibitor
0,557	0,037	Feruloyl esterase inhibitor
0,545	0,028	Linoleate diol synthase inhibitor
0,538	0,026	Alkenylglycerophosphoethanolamine hydrolase inhibitor
0,531	0,020	Cyclic AMP agonist
0,521	0,011	Myc inhibitor
0,512	0,005	Antineoplastic (brain cancer)
0,519	0,014	Antihelmintic (Nematodes)
0,519	0,014	Caspase 8 stimulant
0,512	0,009	Endothelial growth factor antagonist
0,530	0,032	CYP2C8 substrate
0,511	0,013	Antineoplastic (lung cancer)

0,521	0,025	Antinociceptive
0,505	0,011	CYP3A7 substrate
0,510	0,018	CYP2D2 inhibitor
0,543	0,053	CYP3A4 substrate
0,492	0,005	Antineoplastic (pancreatic cancer)
0,500	0,015	Membrane permeability enhancer
0,519	0,034	Cholesterol antagonist
0,535	0,052	CYP3A substrate
0,511	0,030	Analeptic
0,518	0,039	Immunosuppressant
0,483	0,005	Antineoplastic (small cell lung cancer)
0,509	0,031	CYP2B5 substrate
0,562	0,084	Membrane integrity agonist
0,530	0,053	Platelet aggregation stimulant
0,513	0,039	Adenomatous polyposis treatment
0,521	0,051	UDP-N-acetylglucosamine 4-epimerase inhibitor
0,493	0,023	Acetylgalactosaminyl-O-glycosyl-glycoprotein beta-1,3-N-acetylglucosaminyltransferase inhibitor
0,495	0,026	Steroid N-acetylglucosaminyltransferase inhibitor
0,498	0,030	TNF expression inhibitor
0,485	0,018	CYP2A11 substrate
0,481	0,017	CYP2C9 inducer
0,477	0,012	Prostate cancer treatment
0,467	0,004	Vascular dementia treatment
0,508	0,047	Alopecia treatment
0,493	0,032	Histamine release stimulant
0,485	0,030	Caspase 3 stimulant
0,479	0,026	CYP2D15 substrate
0,470	0,019	P-glycoprotein substrate
0,515	0,065	Complement factor D inhibitor
0,471	0,026	CYP17 inhibitor
0,483	0,038	Antipruritic
0,471	0,031	AR expression inhibitor
0,470	0,033	Cyclohexanone monooxygenase inhibitor
0,489	0,055	Glucan endo-1,6-beta-glucosidase inhibitor
0,481	0,046	Ecdysone 20-monooxygenase inhibitor
0,502	0,068	Protein-disulfide reductase (glutathione) inhibitor
0,441	0,010	Endo-1,3(4)-beta-glucanase inhibitor

0,460	0,029	Gonadotropin antagonist
0,516	0,086	Glutamyl endopeptidase II inhibitor
0,513	0,084	Fusarinine-C ornithinesterase inhibitor
0,466	0,039	Antiviral (Rhinovirus)
0,491	0,065	General pump inhibitor
0,464	0,039	Mannan endo-1,4-beta-mannosidase inhibitor
0,452	0,028	Alcohol dehydrogenase (acceptor) inhibitor
0,436	0,014	Pediculicide
0,448	0,026	N-acylmannosamine kinase inhibitor
0,454	0,032	Antiprotozoal (Leishmania)
0,440	0,022	DELTA14-sterol reductase inhibitor
0,433	0,016	CYP2B11 substrate
0,456	0,040	Antisecretoric
0,426	0,010	Antineoplastic (melanoma)
0,497	0,082	Nicotinic alpha4beta4 receptor agonist
0,452	0,037	MMP9 expression inhibitor
0,496	0,081	Calcium channel (voltage-sensitive) activator
0,476	0,062	Chloride peroxidase inhibitor
0,437	0,023	RELA expression inhibitor
0,432	0,018	Vanilloid 1 agonist
0,445	0,034	Rubredoxin-NAD+ reductase inhibitor
0,461	0,051	Exoribonuclease II inhibitor
0,452	0,042	Apyrase inhibitor
0,446	0,037	Pullulanase inhibitor
0,426	0,017	Oxidizing agent
0,462	0,054	Erythropoiesis stimulant
0,439	0,031	Leukopoiesis inhibitor
0,481	0,074	NADPH peroxidase inhibitor
0,437	0,030	Dextranase inhibitor
0,427	0,022	CYP7 inhibitor
0,414	0,009	CYP2D1 substrate
0,411	0,008	Nicotinic alpha4beta2 receptor antagonist
0,425	0,023	CYP2A5 substrate
0,417	0,015	Antiprotozoal (Coccidial)
0,410	0,010	Steroid synthesis inhibitor
0,436	0,038	Bilirubin oxidase inhibitor
0,479	0,082	Oxidoreductase inhibitor

0,432	0,036	Menopausal disorders treatment
0,396	0,005	Alpha-pinene-oxide decyclase inhibitor
0,407	0,017	RNA directed DNA polymerase inhibitor
0,428	0,043	Glyoxylate reductase inhibitor
0,464	0,081	Macrophage colony stimulating factor agonist
0,394	0,016	CYP2B10 substrate
0,402	0,027	Hydroxylamine reductase (NADH) inhibitor
0,380	0,006	Antineoplastic (renal cancer)
0,482	0,109	Kidney function stimulant
0,412	0,041	Aspergillopepsin I inhibitor
0,427	0,058	Polarisation stimulant
0,391	0,023	CDP-diacylglycerol-glycerol-3-phosphate 3-phosphatidyltransferase inhibitor
0,409	0,043	Phosphoinositide 5-phosphatase inhibitor
0,401	0,035	Antiviral (Herpes)
0,407	0,041	Cytostatic
0,382	0,017	Opioid dependency treatment
0,489	0,125	Fibrinolytic
0,408	0,044	Glycerol-3-phosphate oxidase inhibitor
0,381	0,017	Antipruritic, non-allergic
0,408	0,045	GABA aminotransferase inhibitor
0,405	0,044	EIF4E expression inhibitor
0,373	0,013	Glucan 1,6-alpha-glucosidase inhibitor
0,435	0,076	GST A substrate
0,375	0,017	Alkylglycerophosphoethanolamine phosphodiesterase inhibitor
0,373	0,017	Acaricide
0,430	0,075	1,4-Lactonase inhibitor
0,364	0,010	Microtubule formation inhibitor
0,401	0,050	All-trans-retinyl-palmitate hydrolase inhibitor
0,374	0,023	CYP2C6 substrate
0,400	0,050	Antifungal
0,403	0,053	Cytochrome P450 stimulant
0,376	0,026	Protein-Npi-phosphohistidine-sugar phosphotransferase inhibitor
0,364	0,015	Maltose-transporting ATPase inhibitor
0,376	0,026	GST M substrate
0,445	0,097	Chlordecone reductase inhibitor
0,381	0,033	UGT2B4 substrate
0,359	0,012	MAP kinase kinase 4 inhibitor

0,363	0,016	UGT2B17 substrate
0,403	0,056	Na+-transporting two-sector ATPase inhibitor
0,424	0,078	Leukopoiesis stimulant
0,369	0,023	Antitussive
0,384	0,038	Phosphatidylinositol diacylglycerol-lyase inhibitor
0,414	0,070	4-Nitrophenol 2-monooxygenase inhibitor
0,404	0,061	Formaldehyde transketolase inhibitor
0,411	0,069	CYP2C substrate
0,396	0,054	Simian immunodeficiency virus proteinase inhibitor
0,407	0,065	Aldehyde oxidase inhibitor
0,382	0,041	Albendazole monooxygenase inhibitor
0,412	0,073	Trimethylamine-oxide aldolase inhibitor
0,366	0,027	RNA synthesis inhibitor
0,355	0,016	Antiperistaltic
0,407	0,070	Lipoprotein lipase inhibitor
0,397	0,060	HMOX1 expression enhancer
0,371	0,035	CYP2G1 substrate
0,393	0,058	N-acetylneuraminate 7-O(or 9-O)-acetyltransferase inhibitor
0,390	0,057	Antimyopathies
0,421	0,089	Limulus clotting factor B inhibitor
0,343	0,012	Hydroxysteroid dehydrogenase inhibitor
0,343	0,013	Cycloartenol synthase inhibitor
0,370	0,041	Undecaprenyldiphospho-muramoylpentapeptide beta-N-acetylglucosaminyltransferase inhibitor
0,353	0,025	Sclerosant
0,375	0,049	CYP2C11 substrate
0,332	0,006	Retinyl-palmitate esterase inhibitor
0,383	0,058	Prostaglandin-E2 9-reductase inhibitor
0,357	0,031	Sorbitol-6-phosphate 2-dehydrogenase inhibitor
0,412	0,087	Lipid metabolism regulator
0,378	0,055	Malate oxidase inhibitor
0,448	0,125	Pseudolysin inhibitor
0,398	0,075	Beta glucuronidase inhibitor
0,334	0,012	Antineoplastic (liver cancer)
0,410	0,090	Venombin AB inhibitor
0,338	0,017	Antineoplastic (non-small cell lung cancer)
0,360	0,040	Hepatoprotectant
0,365	0,045	Mannose isomerase inhibitor

0,387	0,070	3-Hydroxybenzoate 6-monooxygenase inhibitor
0,356	0,040	Opioid kappa 3 receptor antagonist
0,341	0,026	Thiamine-triphosphatase inhibitor
0,439	0,126	Glycosylphosphatidylinositol phospholipase D inhibitor
0,368	0,055	Sulfite dehydrogenase inhibitor
0,354	0,040	Fructan beta-fructosidase inhibitor
0,395	0,083	Pterin deaminase inhibitor
0,342	0,030	DNA ligase (ATP) inhibitor
0,332	0,020	Antimitotic
0,341	0,033	N-(long-chain-acyl)ethanolamine deacylase inhibitor
0,333	0,025	Vitamin-K-epoxide reductase (warfarin-insensitive) inhibitor
0,381	0,073	Insulin promoter
0,370	0,063	Plastoquinol-plastocyanin reductase inhibitor
0,312	0,005	Nitric oxide scavenger
0,350	0,043	Cyclomaltodextrinase inhibitor
0,395	0,088	Lysine 2,3-aminomutase inhibitor
0,331	0,026	UGT2B28 substrate
0,395	0,089	Pin1 inhibitor
0,329	0,024	Fumarate reductase (NADH) inhibitor
0,354	0,049	4-Coumarate-CoA ligase inhibitor
0,375	0,070	CYP2D6 substrate
0,372	0,068	CYP2D substrate
0,369	0,066	Transcription factor stimulant
0,369	0,066	Transcription factor NF kappa B stimulant
0,335	0,032	Hyaluronic acid agonist
0,385	0,083	Hydrogen dehydrogenase inhibitor
0,338	0,037	DNA polymerase I inhibitor
0,412	0,111	Electron-transferring-flavoprotein dehydrogenase inhibitor
0,371	0,070	L-glutamate oxidase inhibitor
0,321	0,020	Sterol 3-beta-glucosyltransferase inhibitor
0,463	0,162	Nootropic
0,311	0,012	UGT1A5 substrate
0,305	0,006	Antineoplastic (gastric cancer)
0,343	0,044	Glycerol-3-phosphate dehydrogenase inhibitor
0,338	0,041	Clavaminate synthase inhibitor
0,339	0,043	Gestagen antagonist
0,305	0,009	Deoxyribonuclease I inhibitor

0,361	0,066	CYP2A substrate
0,310	0,015	Insecticide
0,344	0,049	Eye irritation, inactive
0,300	0,006	Antineoplastic (thyroid cancer)
0,384	0,090	Fibrinogen receptor antagonist
0,324	0,031	CYP2A3 substrate
0,307	0,014	Ligase inhibitor
0,391	0,099	Phosphatidylcholine-retinol O-acyltransferase inhibitor
0,319	0,027	Dopamine release stimulant
0,339	0,047	Hematopoietic inhibitor
0,323	0,032	Calcium channel activator
0,300	0,009	Antineoplastic, alkylator
0,330	0,039	Proliferative diseases treatment
0,346	0,055	D-lactaldehyde dehydrogenase inhibitor
0,328	0,038	Shikimate O-hydroxycinnamoyltransferase inhibitor
0,344	0,054	Formate-dihydrofolate ligase inhibitor
0,317	0,029	Licheninase inhibitor
0,301	0,015	Antineoplastic (endocrine cancer)
0,315	0,028	Protein synthesis inhibitor
0,327	0,041	Morphine 6-dehydrogenase inhibitor
0,317	0,031	Antineoplastic alkaloid
0,366	0,081	Pro-opiomelanocortin converting enzyme inhibitor
0,325	0,040	Antitoxic
0,296	0,011	Transactivator transcription protein inhibitor
0,408	0,123	Omptin inhibitor
0,357	0,073	NAD(P)+-arginine ADP-ribosyltransferase inhibitor
0,367	0,084	CYP2C9 substrate
0,317	0,035	Flavin-containing monooxygenase inhibitor
0,298	0,018	Aldosterone antagonist
0,331	0,051	Para amino benzoic acid antagonist
0,353	0,073	CYP2C19 inducer
0,350	0,070	Mucinaminylserine mucinaminidase inhibitor
0,315	0,035	Adrenaline release stimulant
0,294	0,014	ATPase inhibitor
0,324	0,045	DNA synthesis inhibitor
0,341	0,062	Sigma receptor agonist
0,337	0,059	Anesthetic general

0,312	0,035	Chemopreventive
0,349	0,071	CYP2E1 inducer
0,380	0,103	Carboxypeptidase Taq inhibitor
0,354	0,078	Thymidylate 5'-phosphatase inhibitor
0,290	0,013	Antineoplastic (squamous cell carcinoma)
0,357	0,082	CYP4A11 substrate
0,341	0,066	CYP19A1 expression inhibitor
0,292	0,018	Glycerone-phosphate O-acyltransferase inhibitor
0,387	0,114	Thromboxane B2 antagonist
0,336	0,063	Phosphopantothenoylcysteine decarboxylase inhibitor
0,307	0,035	Antineoplastic (colorectal cancer)
0,318	0,046	NF-E2-related factor 2 stimulant
0,285	0,013	GST T substrate
0,285	0,013	GST T1-1 substrate
0,297	0,025	Undecaprenyl-diphosphatase inhibitor
0,354	0,083	Malate dehydrogenase (acceptor) inhibitor
0,289	0,020	Tropinesterase inhibitor
0,317	0,047	Cholestanetriol 26-monooxygenase inhibitor
0,302	0,033	Sweetener
0,319	0,050	Antiparasitic
0,385	0,117	Mannotetraose 2-alpha-N-acetylglucosaminyltransferase inhibitor
0,311	0,043	Cutinase inhibitor
0,316	0,049	Taurine-2-oxoglutarate transaminase inhibitor
0,277	0,010	Interferon antagonist
0,299	0,033	Antineoplastic (colon cancer)
0,279	0,014	UGT2B10 substrate
0,348	0,084	Centromere associated protein inhibitor
0,358	0,095	Fragilysin inhibitor
0,273	0,010	Protein synthesis stimulant
0,351	0,089	5 Hydroxytryptamine release stimulant
0,276	0,015	Melanin inhibitor
0,294	0,034	Retinoprotector
0,307	0,048	ICAM1 expression inhibitor
0,277	0,017	Antineoplastic (carcinoma)
0,294	0,034	Galactolipase inhibitor
0,373	0,114	MAP kinase stimulant
0,345	0,086	Manganese peroxidase inhibitor

0,281	0,024	Alkylator
0,340	0,083	Gluconate 5-dehydrogenase inhibitor
0,294	0,038	Aspergillopepsin II inhibitor
0,265	0,009	Interferon gamma antagonist
0,276	0,022	CYP2A10 substrate
0,299	0,045	NOS2 expression inhibitor
0,370	0,116	Preneoplastic conditions treatment
0,298	0,044	Xylan endo-1,3-beta-xylosidase inhibitor
0,292	0,039	Glycine dehydrogenase (decarboxylating) inhibitor
0,265	0,014	Estradiol 17alpha-dehydrogenase inhibitor
0,330	0,078	Acetylesterase inhibitor
0,277	0,025	Cyclooxygenase substrate
0,315	0,064	Antinephritic
0,319	0,068	3-Cyanoalanine hydratase inhibitor
0,290	0,040	Alpha-N-acetylglucosaminidase inhibitor
0,272	0,024	Bile-salt sulfotransferase inhibitor
0,263	0,015	4-Alpha-glucanotransferase inhibitor
0,304	0,056	Anticarcinogenic
0,276	0,029	Antineoplastic (lymphoma)
0,266	0,019	Gastrointestinal motility stimulant
0,270	0,024	Cholesterol oxidase inhibitor
0,304	0,057	Antiviral (Poxvirus)
0,343	0,097	Fatty-acyl-CoA synthase inhibitor
0,349	0,103	CYP2D16 substrate
0,322	0,077	CYP2B6 substrate
0,289	0,044	Signal peptidase I inhibitor
0,250	0,005	Pregnane X receptor agonist
0,318	0,073	Peptide-N4-(N-acetyl-beta-glucosaminyl)asparagine amidase inhibitor
0,269	0,025	Rhizopuspepsin inhibitor
0,314	0,070	Nitrite reductase (NO-forming) inhibitor
0,329	0,086	Intermittent claudication treatment
0,302	0,059	Glycolate dehydrogenase inhibitor
0,341	0,099	Biotinidase inhibitor
0,266	0,025	Antitussive, narcotic
0,289	0,048	Analgesic stimulant
0,299	0,058	Poly(beta-D-mannuronate) lyase inhibitor
0,263	0,023	UGT2B9 substrate

0,326	0,086	Antithrombotic
0,242	0,003	Anesthetic inhalation
0,252	0,014	Antirickettsial
0,320	0,082	CYP2A6 substrate
0,247	0,010	Cyclooxygenase 1 substrate
0,282	0,045	Antihelmintic
0,288	0,052	Yeast ribonuclease inhibitor
0,315	0,078	Radioprotector
0,377	0,140	5 Hydroxytryptamine uptake stimulant
0,271	0,037	Bisphosphoglycerate mutase inhibitor
0,256	0,022	Galacturan 1,4-alpha-galacturonidase inhibitor
0,294	0,061	Glucan 1,4-alpha-maltotetraohydrolase inhibitor
0,288	0,055	Ethanolamine-phosphate cytidylyltransferase inhibitor
0,296	0,063	Lysyl oxidase inhibitor
0,365	0,135	NADPH-cytochrome-c2 reductase inhibitor
0,336	0,106	Arginine 2-monooxygenase inhibitor
0,260	0,030	Antimycoplasmal
0,260	0,031	Cholesterol synthesis inhibitor
0,341	0,112	Radiosensitizer
0,259	0,030	Vanilloid agonist
0,255	0,027	Antineoplastic (bone cancer)
0,326	0,098	Sphinganine kinase inhibitor
0,278	0,050	Raynaud's phenomenon treatment
0,305	0,078	H+-transporting two-sector ATPase inhibitor
0,272	0,045	Phospholipase A1 inhibitor
0,316	0,090	(R)-Pantolactone dehydrogenase (flavin) inhibitor
0,330	0,105	Interleukin 2 agonist
0,255	0,031	CYP4F2 substrate
0,310	0,085	CYP2F1 substrate
0,246	0,021	UGT2B18 substrate
0,331	0,108	Alcohol O-acetyltransferase inhibitor
0,250	0,028	CYP2C8 inducer
0,308	0,086	Mediator release inhibitor
0,290	0,069	Sulfite oxidase inhibitor
0,295	0,074	IgA-specific serine endopeptidase inhibitor
0,354	0,134	Fructose 5-dehydrogenase inhibitor
0,302	0,083	Chitinase inhibitor

0,330	0,111	Nucleotide metabolism regulator
0,356	0,137	Octopamine antagonist
0,352	0,133	Thioredoxin inhibitor
0,254	0,036	Interleukin 6 antagonist
0,284	0,066	Antibacterial
0,245	0,027	GABA B receptor agonist
0,242	0,025	Glucosyl transferase inhibitor
0,299	0,082	Adenylyl-sulfate reductase inhibitor
0,249	0,033	1,4-Alpha-glucan branching enzyme inhibitor
0,275	0,060	Spasmolytic, Papaverin-like
0,219	0,005	Interleukin 10 antagonist
0,297	0,083	Antiinflammatory, ophthalmic
0,290	0,077	UGT1A9 substrate
0,291	0,078	Antiinflammatory, intestinal
0,290	0,076	Antiprotozoal (Amoeba)
0,335	0,121	Spasmolytic, urinary
0,257	0,044	UGT2B7 substrate
0,286	0,073	4-Nitrophenylphosphatase inhibitor
0,233	0,021	Chitin synthase inhibitor
0,254	0,042	Styrene-oxide isomerase inhibitor
0,311	0,100	Phenol O-methyltransferase inhibitor
0,235	0,023	Endoglycosylceramidase inhibitor
0,237	0,026	Testosterone 17beta-dehydrogenase inhibitor
0,267	0,058	Transcription factor NF kappa A inhibitor
0,300	0,090	Fucosterol-epoxide lyase inhibitor
0,282	0,073	Thiosulfate dehydrogenase inhibitor
0,344	0,135	Oxygen scavenger
0,366	0,157	Phthalate 4,5-dioxygenase inhibitor
0,256	0,047	Phosphoenolpyruvate mutase inhibitor
0,267	0,059	Levanase inhibitor
0,255	0,047	N-acetyllactosaminide beta-1,3-N-acetylglucosaminyltransferase inhibitor
0,292	0,085	ADP-thymidine kinase inhibitor
0,280	0,073	2-Haloacid dehalogenase inhibitor
0,232	0,026	CYP4B substrate
0,297	0,091	Antiviral (Adenovirus)
0,241	0,035	CYP1B substrate
0,301	0,096	1-Acylglycerol-3-phosphate O-acyltransferase inhibitor

0,280	0,075	Cytochrome-b5 reductase inhibitor
0,212	0,009	4-Carboxymethyl-4-methylbutenolide mutase inhibitor
0,302	0,100	Antiinfective
0,263	0,060	2,3,4,5-Tetrahydropyridine-2,6-dicarboxylate N-succinyltransferase inhibitor
0,230	0,028	Antihypotensive
0,306	0,104	Histidine N-acetyltransferase inhibitor
0,306	0,105	Calcium regulator
0,257	0,055	Chaperonin ATPase inhibitor
0,247	0,047	Anthranilate-CoA ligase inhibitor
0,211	0,011	CYP3A5 inducer
0,309	0,109	Cyclic AMP phosphodiesterase inhibitor
0,226	0,026	Arachidonic acid antagonist
0,271	0,073	CYP2C29 substrate
0,249	0,051	UGT1A4 substrate
0,244	0,047	Dynein ATPase inhibitor
0,281	0,085	Chitosanase inhibitor
0,250	0,053	Polyribonucleotide nucleotidyltransferase inhibitor
0,244	0,049	Polygalacturonase inhibitor
0,348	0,153	Phospholipid-translocating ATPase inhibitor
0,224	0,029	Ferredoxin-NADP+ reductase inhibitor
0,211	0,016	Hypoxia inducible factor 1 alpha inhibitor
0,222	0,028	Protein kinase stimulant
0,279	0,085	tRNA-pseudouridine synthase I inhibitor
0,328	0,133	2-Dehydropantoate 2-reductase inhibitor
0,212	0,017	Choloylglycine hydrolase inhibitor
0,218	0,024	Adenylate cyclase inhibitor
0,217	0,023	Contraceptive female
0,232	0,039	UGT2B15 substrate
0,266	0,074	Antiviral (Influenza A)
0,243	0,051	Cellulose 1,4-beta-cellobiosidase inhibitor
0,209	0,017	Corticosteroid antagonist
0,249	0,058	Oxytocic
0,215	0,023	Riboflavin phosphotransferase inhibitor
0,230	0,039	Lymphocytopoiesis inhibitor
0,243	0,052	D-alanine 2-hydroxymethyltransferase inhibitor
0,277	0,086	Limulus clotting factor C inhibitor
0,242	0,053	Myeloblastin inhibitor

0,274	0,086	Lysostaphin inhibitor
0,228	0,039	Mucolytic
0,300	0,111	Prion diseases treatment
0,226	0,037	Antihematotoxic
0,239	0,051	Polyneuridine-aldehyde esterase inhibitor
0,244	0,056	Alpha-1,6-mannosyl-glycoprotein 4-beta-N-acetylglucosaminyltransferase inhibitor
0,304	0,116	Vasodilator, coronary
0,341	0,153	Glyceryl-ether monooxygenase inhibitor
0,298	0,111	Muscular dystrophy treatment
0,317	0,130	RNA-directed RNA polymerase inhibitor
0,250	0,063	Cyclic AMP modulator
0,200	0,014	Adenylate cyclase stimulant
0,279	0,093	Nitrite reductase [NAD(P)H] inhibitor
0,232	0,045	Expectorant
0,229	0,043	Glucan 1,4-beta-glucosidase inhibitor
0,294	0,108	Cyanoalanine nitrilase inhibitor
0,216	0,031	L-iduronidase inhibitor
0,277	0,093	Methylenetetrahydrofolate reductase (NADPH) inhibitor
0,189	0,004	Phosphatidylinositol 3-kinase stimulant
0,222	0,038	Antineoplastic (glioblastoma multiforme)
0,200	0,016	Alpha-L-rhamnosidase inhibitor
0,245	0,060	CYP2B2 substrate
0,279	0,095	Creatininase inhibitor
0,208	0,024	CYP4B1 substrate
0,258	0,075	Phospholipase C inhibitor
0,227	0,044	Lipotropic
0,279	0,097	Enteropeptidase inhibitor
0,211	0,029	Glutamate (mGluR5) agonist
0,248	0,066	Lactose synthase inhibitor
0,277	0,096	3-Phytase inhibitor
0,350	0,170	CYP2C8 inhibitor
0,250	0,070	Beta-carotene 15,15'-monooxygenase inhibitor
0,250	0,071	Glyoxylate oxidase inhibitor
0,241	0,063	Oryzin inhibitor
0,293	0,115	Glucose oxidase inhibitor
0,181	0,003	Inositol 1,4,5-triphosphate receptor antagonist
0,290	0,112	Dehydro-L-gulonate decarboxylase inhibitor

0,235	0,058	Choleretic
0,214	0,038	ADP-ribosylarginine hydrolase inhibitor
0,211	0,035	GST M1-1 substrate
0,298	0,122	Superoxide dismutase inhibitor
0,300	0,125	Cl--transporting ATPase inhibitor
0,183	0,009	Galactosylceramidase inhibitor
0,241	0,067	Peroxidase substrate
0,230	0,057	Anesthetic
0,188	0,015	CYP19 substrate
0,225	0,052	P-glycoprotein inhibitor
0,202	0,030	Protein-glucosylgalactosylhydroxylysine glucosidase inhibitor
0,288	0,116	MAP3K5 inhibitor
0,205	0,033	Thioredoxin reductase inhibitor
0,233	0,062	CYP1A1 inhibitor
0,234	0,064	ATP-binding cassette A1 stimulant
0,220	0,050	Alpha-amylase inhibitor
0,222	0,052	Sedoheptulose-bisphosphatase inhibitor
0,260	0,091	Glutamate-tRNA ligase inhibitor
0,248	0,079	3C-like protease (Human coronavirus) inhibitor
0,215	0,046	2-Acylglycerol O-acyltransferase inhibitor
0,243	0,075	Cell wall biosynthesis inhibitor
0,296	0,128	Amine dehydrogenase inhibitor
0,187	0,019	Isoamylase inhibitor
0,252	0,085	CYP1A1 substrate
0,211	0,043	Laxative
0,218	0,051	Severe acute respiratory syndrome treatment
0,179	0,013	Antitreponemal
0,196	0,030	SMN2 expression enhancer
0,247	0,081	Antiviral (CMV)
0,252	0,086	Indanol dehydrogenase inhibitor
0,184	0,019	Guanylate cyclase stimulant
0,240	0,075	Quinoprotein glucose dehydrogenase inhibitor
0,227	0,063	Ubiquitin thiolesterase inhibitor
0,229	0,064	3-Methylbutanal reductase inhibitor
0,307	0,144	Nicotine dehydrogenase inhibitor
0,239	0,076	Antiprotozoal (Trichomonas)
0,201	0,038	2-Dehydropantoate aldolase inhibitor

0,232	0,070	Aminomuconate-semialdehyde dehydrogenase inhibitor
0,204	0,041	Phosphoenolpyruvate-protein phosphotransferase inhibitor
0,177	0,014	UGT2B11 substrate
0,222	0,059	D-threo-aldose 1-dehydrogenase inhibitor
0,293	0,131	Aspartate-phenylpyruvate transaminase inhibitor
0,263	0,102	P-benzoquinone reductase (NADPH) inhibitor
0,235	0,074	UGT1A6 substrate
0,202	0,040	Antineoplastic (cervical cancer)
0,249	0,089	2-Hydroxymuconate-semialdehyde hydrolase inhibitor
0,202	0,042	Glycerol-1-phosphatase inhibitor
0,239	0,079	Glutathione dehydrogenase (ascorbate) inhibitor
0,234	0,074	Bisphosphoglycerate phosphatase inhibitor
0,229	0,070	Pseudouridylate synthase inhibitor
0,284	0,125	Leukotriene-C4 synthase inhibitor
0,165	0,007	Acetylcholine M3 receptor agonist
0,170	0,012	Shab potassium channel blocker
0,281	0,123	Polyamine-transporting ATPase inhibitor
0,200	0,043	Alkene monooxygenase inhibitor
0,237	0,080	Linoleoyl-CoA desaturase inhibitor
0,240	0,083	CYP2E1 substrate
0,210	0,053	Growth stimulant
0,207	0,050	Menstruation disorders treatment
0,228	0,072	1,2-alpha-L-fucosidase inhibitor
0,220	0,063	Rhodotorulapepsin inhibitor
0,207	0,051	Alpha-glucuronidase inhibitor
0,258	0,102	Isopenicillin-N epimerase inhibitor
0,228	0,072	N-acetyllactosamine synthase inhibitor
0,216	0,061	Mannitol-1-phosphatase inhibitor
0,209	0,054	Plasmanylethanolamine desaturase inhibitor
0,220	0,066	Aromatic-hydroxylamine O-acetyltransferase inhibitor
0,215	0,061	3-Demethylubiquinone-9 3-O-methyltransferase inhibitor
0,225	0,071	Alcohol dehydrogenase [NAD(P)+] inhibitor
0,247	0,093	Aspergillus nuclease S1 inhibitor
0,195	0,042	Ganglioside galactosyltransferase inhibitor
0,255	0,102	N-formylmethionyl-peptidase inhibitor
0,167	0,014	DELTA24-sterol reductase inhibitor
0,177	0,024	Glycosylphosphatidylinositol diacylglycerol-lyase inhibitor

0,219	0,066	Prunasin beta-glucosidase inhibitor
0,267	0,115	Prostaglandin-A1 DELTA-isomerase inhibitor
0,301	0,148	Fibrolase inhibitor
0,188	0,037	Phosphatidate cytidylyltransferase inhibitor
0,290	0,139	Nitrate reductase (cytochrome) inhibitor
0,314	0,163	Aminobutyraldehyde dehydrogenase inhibitor
0,159	0,008	Aldose 1-epimerase inhibitor
0,237	0,087	Antihypercholesterolemic
0,209	0,059	P-glycoprotein 1 inhibitor
0,254	0,104	CYP4A substrate
0,193	0,043	Methyltransferase substrate
0,236	0,086	CYP2E substrate
0,181	0,032	Pectin lyase inhibitor
0,183	0,033	Antibiotic
0,173	0,024	Beta-glucosidase inhibitor
0,300	0,152	(R)-6-hydroxynicotine oxidase inhibitor
0,227	0,079	Antidote
0,188	0,041	Cellulase inhibitor
0,183	0,036	Skin whitener
0,185	0,038	Diacylglycerol cholinephosphotransferase inhibitor
0,235	0,089	Dipeptidase E inhibitor
0,196	0,050	Antineoplastic (bladder cancer)
0,265	0,119	(S)-6-hydroxynicotine oxidase inhibitor
0,277	0,131	Cancer associated disorders treatment
0,150	0,005	Alpha-mannosidase inhibitor
0,197	0,052	Aldehyde dehydrogenase (NADP+) inhibitor
0,208	0,063	UGT1A1 substrate
0,190	0,045	CYP2B18 substrate
0,251	0,106	4-Methoxybenzoate monooxygenase (O-demethylating) inhibitor
0,184	0,039	Valine-tRNA ligase inhibitor
0,165	0,021	Narcotic antagonist
0,198	0,054	NAD+ synthase (glutamine-hydrolysing) inhibitor
0,204	0,060	Retinol dehydrogenase inhibitor
0,171	0,028	Trehalose-phosphatase inhibitor
0,203	0,060	Inorganic diphosphatase inhibitor
0,176	0,033	CYP19 inhibitor
0,169	0,027	Antineoplastic antibiotic

0,157	0,015	Testosterone agonist
0,180	0,038	Glycopeptide alpha-N-acetylgalactosaminidase inhibitor
0,151	0,010	Imidazoleglycerol-phosphate dehydratase inhibitor
0,285	0,145	Dimethylargininase inhibitor
0,258	0,118	4-Hydroxyproline epimerase inhibitor
0,158	0,018	Secretase stimulant
0,158	0,018	Secretase alpha stimulant
0,248	0,108	Urethanase inhibitor
0,241	0,102	Aldehyde dehydrogenase (pyrroloquinoline-quinone) inhibitor
0,176	0,037	Peristaltic stimulant
0,169	0,031	Alpha-N-acetylgalactosaminidase inhibitor
0,201	0,064	Antinaupathic
0,316	0,179	Diabetic neuropathy treatment
0,243	0,106	Peptide alpha-N-acetyltransferase inhibitor
0,164	0,027	Urolithiasis treatment
0,193	0,056	Maleate isomerase inhibitor
0,170	0,034	Cocain dependency treatment
0,282	0,146	Spermidine dehydrogenase inhibitor
0,188	0,052	Stromelysin 2 inhibitor
0,197	0,062	Aureolysin inhibitor
0,206	0,071	Antiparkinsonian, tremor relieving
0,161	0,025	Tubulin antagonist
0,236	0,101	CYP2C10 substrate
0,198	0,063	4-Hydroxybenzoate nonaprenyltransferase inhibitor
0,180	0,046	Allantoate deiminase inhibitor
0,147	0,013	Epoxide hydrolase substrate
0,181	0,047	Polar-amino-acid-transporting ATPase inhibitor
0,241	0,108	Vasodilator
0,148	0,014	CYP4F substrate
0,184	0,051	Pyruvate carboxylase inhibitor
0,197	0,064	Antianorexic
0,172	0,039	Histamine agonist
0,194	0,061	Serratia marcescens nuclease inhibitor
0,235	0,103	Thiamine pyridinylase inhibitor
0,173	0,041	Gaucher disease treatment
0,268	0,135	Retinoic acid metabolism inhibitor
0,228	0,096	Long-chain-aldehyde dehydrogenase inhibitor

0,158	0,026	Demethylsterigmatocystin 6-O-methyltransferase inhibitor
0,270	0,138	Tpr proteinase (Porphyromonas gingivalis) inhibitor
0,150	0,019	Rhodopsin kinase inhibitor
0,147	0,016	Mycodextranase inhibitor
0,195	0,064	Urate-ribonucleotide phosphorylase inhibitor
0,202	0,072	Lipocortins synthesis antagonist
0,256	0,126	Inotropic
0,134	0,004	Potassium channel Kv1.3 blocker
0,146	0,016	Leucine-tRNA ligase inhibitor
0,185	0,055	Laminaribiose phosphorylase inhibitor
0,303	0,173	2-Hydroxyquinoline 8-monooxygenase inhibitor
0,244	0,115	CYP2C19 substrate
0,168	0,039	Xylose isomerase inhibitor
0,137	0,009	Osmotic diuretic
0,230	0,102	Protein-arginine deiminase inhibitor
0,210	0,082	Pappalysin-1 inhibitor
0,175	0,048	Alpha-N-arabinofuranosidase inhibitor
0,187	0,060	Aryldialkylphosphatase inhibitor
0,215	0,087	UDP-glucuronosyltransferase substrate
0,163	0,036	Cholate-CoA ligase inhibitor
0,159	0,032	Sodium/bile acid cotransporter inhibitor
0,146	0,019	Anabolic
0,200	0,073	N-Acyl-D-aspartate deacylase inhibitor
0,136	0,010	Alpha-1,6-mannosyl-glycoprotein 6-beta-N-acetylglucosaminyltransferase inhibitor
0,182	0,056	Myosin-light-chain-phosphatase inhibitor
0,136	0,010	Glutamate (mGluR1) antagonist
0,141	0,016	Gentamicin 2''-nucleotidyltransferase inhibitor
0,178	0,053	Galactokinase inhibitor
0,219	0,094	Viral entry inhibitor
0,157	0,033	DNA directed DNA polymerase inhibitor
0,217	0,092	Antifibrinolytic
0,177	0,052	Ethanolaminephosphotransferase inhibitor
0,186	0,062	CYP1B1 substrate
0,161	0,037	NAD(P)+ transhydrogenase (B-specific) inhibitor
0,233	0,110	Immunostimulant
0,129	0,006	Mannosidase inhibitor
0,159	0,036	(R)-limonene 6-monooxygenase inhibitor

0,150	0,028	Glutamate release inhibitor
0,272	0,151	S-formylglutathione hydrolase inhibitor
0,214	0,093	[acyl-carrier-protein] S-acetyltransferase inhibitor
0,152	0,031	Diacylglycerol kinase inhibitor
0,213	0,092	Salicylate 1-monooxygenase inhibitor
0,154	0,033	Glyceraldehyde-3-phosphate dehydrogenase (phosphorylating) inhibitor
0,134	0,014	Alpha glucosidase inhibitor
0,165	0,045	CDP-diacylglycerol-inositol 3-phosphatidyltransferase inhibitor
0,184	0,064	LDLR expression enhancer
0,174	0,055	Aspartate-tRNA ligase inhibitor
0,243	0,125	CYP2C18 substrate
0,153	0,034	D-arabinonolactone oxidase inhibitor
0,127	0,009	RNA-directed RNA polymerase stimulant
0,255	0,137	CYP2A8 substrate
0,199	0,082	Horrilysin inhibitor
0,179	0,062	Lactaldehyde reductase inhibitor
0,200	0,084	Polarisation inhibitor
0,198	0,081	Cyclopropane-fatty-acyl-phospholipid synthase inhibitor
0,297	0,180	Mucositis treatment
0,166	0,050	BRAF expression inhibitor
0,146	0,029	Succinate dehydrogenase inhibitor
0,133	0,017	Nicotinic receptor alpha7 subunit antagonist
0,185	0,069	UGT1A3 substrate
0,187	0,071	Glutathione peroxidase inhibitor
0,200	0,084	Diabetic nephropathy treatment
0,270	0,155	N-hydroxyarylamine O-acetyltransferase inhibitor
0,128	0,013	Ca2+-transporting ATPase inhibitor
0,185	0,070	Poly(alpha-L-guluronate) lyase inhibitor
0,127	0,012	Alpha,alpha-trehalose phosphorylase inhibitor
0,183	0,068	Vascular adhesion protein 1 inhibitor
0,128	0,013	Anticholelithogenic
0,173	0,059	3-Chloro-D-alanine dehydrochlorinase inhibitor
0,131	0,017	Alpha-L-fucosidase inhibitor
0,181	0,067	Gamma-butyrobetaine dioxygenase inhibitor
0,173	0,060	2-Haloacid dehalogenase (configuration-inverting) inhibitor
0,284	0,171	Pancreatic elastase inhibitor
0,133	0,020	Alternansucrase inhibitor

0,235	0,122	Spasmolytic
0,238	0,125	4-Hydroxymandelate oxidase inhibitor
0,196	0,085	Guanosine-3',5'-bis(diphosphate) 3'-diphosphatase inhibitor
0,161	0,050	UGT1A7 substrate
0,162	0,051	3-Ketovalidoxylamine C-N-lyase inhibitor
0,181	0,071	4-Chlorobenzoyl-CoA dehalogenase inhibitor
0,157	0,048	Contraceptive
0,118	0,008	Mannosyl-oligosaccharide 1,2-alpha-mannosidase inhibitor
0,191	0,081	Neurotrophic factor enhancer
0,135	0,025	Cytokine release inhibitor
0,150	0,041	Glycerol-3-phosphate O-acyltransferase inhibitor
0,198	0,089	CYP1A2 inducer
0,172	0,063	D-xylulose reductase inhibitor
0,176	0,067	Lipoxygenase substrate
0,116	0,007	Prostacyclin agonist
0,228	0,119	Age-related macular degeneration treatment
0,111	0,003	Antimitotic, Podophyllotoxin-like
0,208	0,100	Prolyl aminopeptidase inhibitor
0,239	0,130	Antimycobacterial
0,142	0,034	Nicotinamide-nucleotide adenylyltransferase inhibitor
0,176	0,068	N-acetylneuraminate 4-O-acetyltransferase inhibitor
0,136	0,028	Retinol O-fatty-acyltransferase inhibitor
0,221	0,114	Lipid peroxidase inhibitor
0,194	0,087	2-Hydroxy-3-oxoadipate synthase inhibitor
0,200	0,093	Interleukin 10 agonist
0,201	0,094	UGT1A substrate
0,130	0,023	Protein kinase C stimulant
0,213	0,106	CYP2B substrate
0,202	0,095	Ceramide glucosyltransferase inhibitor
0,122	0,016	Heme oxygenase inhibitor
0,150	0,044	Endo-1,4-beta-xylanase inhibitor
0,223	0,117	Glutamine-phenylpyruvate transaminase inhibitor
0,162	0,057	HMG CoA synthase inhibitor
0,134	0,029	Beta-amylase inhibitor
0,177	0,072	UGT2B substrate
0,126	0,021	Squalene-hopene cyclase inhibitor
0,132	0,027	Squalene epoxidase inhibitor

0,249	0,145	Antiulcerative
0,130	0,026	Globoside alpha-N-acetylgalactosaminyltransferase inhibitor
0,194	0,090	GST P substrate
0,157	0,053	UGT1A8 substrate
0,130	0,027	Ribose-5-phosphate-ammonia ligase inhibitor
0,110	0,006	Glutamate (mGluR3) agonist
0,151	0,048	Carnitine dehydratase inhibitor
0,134	0,031	Deoxycytidine deaminase inhibitor
0,148	0,044	CYP26A substrate
0,189	0,086	2-Oxoaldehyde dehydrogenase (NADP+) inhibitor
0,117	0,014	Arabinogalactan endo-1,4-beta-galactosidase inhibitor
0,209	0,107	Phosphatidylcholine-sterol O-acyltransferase inhibitor
0,217	0,115	Adenosine regulator
0,163	0,061	Geranylgeranyl-diphosphate geranylgeranyltransferase inhibitor
0,215	0,113	Succinate-semialdehyde dehydrogenase [NAD(P)+] inhibitor
0,149	0,047	Transcription factor NF kappa B inhibitor
0,200	0,099	Benzaldehyde dehydrogenase (NADP+) inhibitor
0,138	0,038	Deoxyribonuclease X inhibitor
0,134	0,034	5 Hydroxytryptamine 4A antagonist
0,110	0,010	Glucose-6-phosphate translocase inhibitor
0,198	0,098	Tankyrase inhibitor
0,135	0,035	Glucan 1,3-beta-glucosidase inhibitor
0,151	0,052	2,2-Dialkylglycine decarboxylase (pyruvate) inhibitor
0,142	0,042	Tardive dyskinesia treatment
0,154	0,054	Pectate lyase inhibitor
0,238	0,139	Cardioprotectant
0,130	0,031	Isomaltulose synthase inhibitor
0,124	0,026	Phenylalanine-tRNA ligase inhibitor
0,188	0,090	Gastric antisecretory
0,106	0,008	3-Alpha-hydroxysteroid dehydrogenase (B-specific) inhibitor
0,155	0,058	VCAM1 expression inhibitor
0,153	0,057	UGT1A10 substrate
0,165	0,068	Antineoplastic (glioma)
0,115	0,018	Threonine-tRNA ligase inhibitor
0,234	0,137	Methylamine-glutamate N-methyltransferase inhibitor
0,157	0,061	Aldehyde oxidase substrate
0,157	0,061	Galactose oxidase inhibitor

0,106	0,010	1,3-Beta-glucan synthase inhibitor
0,171	0,076	2,3-Dihydroxyindole 2,3-dioxygenase inhibitor
0,211	0,115	Chloride channel activator
0,215	0,120	Transketolase inhibitor
0,229	0,134	Chenodeoxycholoyltaurine hydrolase inhibitor
0,159	0,065	SULT1E1 substrate
0,101	0,006	RNA agonist
0,182	0,087	Glutarate-semialdehyde dehydrogenase inhibitor
0,275	0,181	Antihypoxic
0,149	0,055	N-(5-amino-5-carboxypentanoyl)-L-cysteinyl-D-valine synthase inhibitor
0,173	0,079	Glycine C-acetyltransferase inhibitor
0,150	0,056	Calcium antagonist
0,127	0,034	Cholestenone 5alpha-reductase inhibitor
0,185	0,091	Skin irritation, inactive
0,161	0,068	Inulinase inhibitor
0,121	0,029	Phosphatidylglycerol-membrane-oligosaccharide glycerophosphotransferase inhibitor
0,128	0,036	Beta-D-fucosidase inhibitor
0,178	0,087	2-Enoate reductase inhibitor
0,157	0,065	Acetylenecarboxylate hydratase inhibitor
0,113	0,021	Alpha,alpha-trehalose phosphorylase (configuration-retaining) inhibitor
0,161	0,070	Cytochrome-b5 reductase substrate
0,140	0,049	Beta-mannosidase inhibitor
0,112	0,021	Fatty-acyl-ethyl-ester synthase inhibitor
0,136	0,045	Lanosterol 14 alpha demethylase inhibitor
0,168	0,077	Glyoxylate reductase (NADP+) inhibitor
0,123	0,033	Ketohexokinase inhibitor
0,103	0,013	UGT2B7H substrate
0,103	0,013	UGT2B7Y substrate
0,188	0,099	Retinal dehydrogenase inhibitor
0,100	0,011	Prostaglandin agonist
0,105	0,017	Alpha,alpha-phosphotrehalase inhibitor
0,188	0,100	N-hydroxy-2-acetamidofluorene reductase inhibitor
0,196	0,108	Gluconolactonase inhibitor
0,132	0,044	Ribonuclease inhibitor
0,113	0,025	High-mannose-oligosaccharide beta-1,4-N-acetylglucosaminyltransferase inhibitor
0,139	0,051	UDP-N-acetylglucosamine-dolichyl-phosphate N-acetylglucosaminephosphotransferase inhibitor

0,154	0,067	Cerebroside-sulfatase inhibitor
0,107	0,019	Cushing's syndrome treatment
0,166	0,078	N-methyl-2-oxoglutaramate hydrolase inhibitor
0,131	0,044	Fructose-2,6-bisphosphate 6-phosphatase inhibitor
0,118	0,031	Ribonuclease U2 inhibitor
0,148	0,061	Glucuronolactone reductase inhibitor
0,093	0,007	Actin depolymerization stimulant
0,142	0,055	Prolactin inhibitor
0,113	0,027	Sterol 24-C-methyltransferase inhibitor
0,177	0,091	Tauropine dehydrogenase inhibitor
0,119	0,033	3-Oxosteroid 1-dehydrogenase inhibitor
0,092	0,007	CYP24 inhibitor
0,125	0,039	Polynucleotide adenylyltransferase inhibitor
0,123	0,038	1,5-Anhydro-D-fructose reductase inhibitor
0,120	0,035	Glyceraldehyde-3-phosphate dehydrogenase (NADP+) inhibitor
0,104	0,019	CYP4F12 substrate
0,136	0,051	Fructose-2,6-bisphosphate 2-phosphatase inhibitor
0,104	0,019	Steroid sulfotransferase inhibitor
0,107	0,022	3(or 17)alpha-hydroxysteroid dehydrogenase inhibitor
0,100	0,016	Butyrylcholinesterase inhibitor
0,129	0,044	DNA nucleotidylexotransferase inhibitor
0,112	0,027	Inositol-3-phosphate synthase inhibitor
0,144	0,060	Glutathione S-transferase substrate
0,150	0,066	Cyclamate sulfohydrolase inhibitor
0,159	0,075	IgA-specific metalloendopeptidase inhibitor
0,195	0,111	NADH kinase inhibitor
0,146	0,063	Dolichyl-phosphatase inhibitor
0,209	0,126	Antiparkinsonian, rigidity relieving
0,158	0,075	3'-Nucleotidase inhibitor
0,109	0,027	Long-chain-fatty-acid-CoA ligase inhibitor
0,095	0,012	Hyperparathyroidism treatment
0,162	0,080	Snapalysin inhibitor
0,143	0,061	Acetyl-CoA C-acyltransferase inhibitor
0,095	0,013	Acetylcholine muscarinic agonist
0,229	0,148	CYP1A substrate
0,123	0,041	2-Dehydropantolactone reductase (A-specific) inhibitor
0,278	0,196	Sulfur reductase inhibitor

0,139	0,057	Agmatinase inhibitor
0,132	0,051	NADH dehydrogenase inhibitor
0,098	0,017	Protein 50S ribosomal subunit inhibitor
0,182	0,101	Di-trans,poly-cis-decaprenylcistransferase inhibitor
0,227	0,146	Tyrosine 3 hydroxylase inhibitor
0,096	0,015	ACK1 kinase inhibitor
0,201	0,120	Glutaminyl-peptide cyclotransferase inhibitor
0,140	0,061	Protein-S-isoprenylcysteine O-methyltransferase inhibitor
0,155	0,075	Calcium-sensing receptor agonist
0,091	0,011	UGT2B4D substrate
0,091	0,011	UGT2B4E substrate
0,091	0,011	UGT2B19 substrate
0,091	0,011	UGT2B23 substrate
0,091	0,011	UGT2B30 substrate
0,147	0,068	Anesthetic local
0,127	0,048	Heparan-alpha-glucosaminide N-acetyltransferase inhibitor
0,138	0,059	Glycogen (starch) synthase inhibitor
0,130	0,052	Trimethyllysine dioxygenase inhibitor
0,168	0,089	Leucine dehydrogenase inhibitor
0,121	0,043	Aconitate decarboxylase inhibitor
0,168	0,090	Phosphatidylglycerophosphatase inhibitor
0,117	0,039	Bis(5'-adenosyl)-triphosphatase inhibitor
0,145	0,067	Dry eye syndrome treatment
0,127	0,049	Lysozyme inhibitor
0,145	0,068	Trans-1,2-dihydrobenzene-1,2-diol dehydrogenase inhibitor
0,096	0,020	mRNA (guanine-N7-)-methyltransferase inhibitor
0,158	0,081	CYP2B1 substrate
0,113	0,036	Gluconokinase inhibitor
0,219	0,143	Peptidyl-dipeptidase Dcp inhibitor
0,108	0,032	Homoserine O-succinyltransferase inhibitor
0,238	0,162	Antiprotozoal (Trypanosoma)
0,152	0,075	Farnesyltranstransferase inhibitor
0,134	0,058	Deoxyribose-phosphate aldolase inhibitor
0,118	0,042	CMP-N-acetylneuraminate monooxygenase inhibitor
0,169	0,094	Antileukemic
0,117	0,042	GABA transporter 3 inhibitor
0,168	0,093	Antiprotozoal (Plasmodium)

0,165	0,089	Glutamate 5-kinase inhibitor
0,143	0,068	Antibacterial, ophthalmic
0,106	0,031	Xylan 1,4-beta-xylosidase inhibitor
0,115	0,040	Threonine ammonia-lyase inhibitor
0,107	0,032	Beta-N-acetylhexosaminidase inhibitor
0,183	0,108	Nucleoside oxidase (H2O2-forming) inhibitor
0,089	0,015	Glutathione reductase stimulant
0,089	0,015	Glutathione reductase (NADPH) stimulant
0,144	0,070	Phosphoinositide phospholipase C inhibitor
0,113	0,039	Oligo-1,6-glucosidase inhibitor
0,124	0,049	Galactoside 2-alpha-L-fucosyltransferase inhibitor
0,107	0,033	L-fuconate dehydratase inhibitor
0,086	0,012	Corticotropin releasing factor antagonist
0,099	0,026	Opioid mu 2 receptor antagonist
0,198	0,125	Photosensitizer
0,119	0,046	Heat shock protein antagonist
0,129	0,056	NAD+ kinase inhibitor
0,170	0,097	GABA C receptor agonist
0,115	0,042	Acetylcholine agonist
0,134	0,062	Glucosamine-6-phosphate deaminase inhibitor
0,129	0,057	Estrone sulfotransferase inhibitor
0,243	0,170	Hydroxylamine oxidase inhibitor
0,090	0,018	Androgen agonist
0,176	0,104	Carboxypeptidase D inhibitor
0,093	0,021	Steroid DELTA-isomerase inhibitor
0,129	0,058	Poly(ADP-ribose) glycohydrolase inhibitor
0,185	0,114	S-alkylcysteine lyase inhibitor
0,287	0,216	Taurine dehydrogenase inhibitor
0,297	0,226	Heat shock protein 27 antagonist
0,077	0,006	Peptidyl-prolyl cis-trans isomerase inhibitor
0,112	0,041	Cell wall synthesis inhibitor
0,077	0,006	Na+ K+ transporting ATPase inhibitor
0,239	0,168	Mitochondrial processing peptidase inhibitor
0,095	0,025	Dextransucrase inhibitor
0,099	0,029	Alpha,alpha-trehalose-phosphate synthase (UDP-forming) inhibitor
0,130	0,060	Bacterial efflux pump inhibitor
0,110	0,040	Capillary fragility treatment

0,170	0,101	GST P1-1 substrate
0,137	0,068	Saccharolysin inhibitor
0,152	0,083	N6-methyl-lysine oxidase inhibitor
0,142	0,073	Aryl sulfotransferase inhibitor
0,131	0,063	UGT2B1 substrate
0,133	0,064	D-lactate dehydrogenase inhibitor
0,073	0,004	Rotamase (FKBP) inhibitor
0,095	0,027	SULT2A1 substrate
0,133	0,066	Biotin carboxylase inhibitor
0,147	0,080	Penicillopepsin inhibitor
0,074	0,007	Sucrose phosphorylase inhibitor
0,192	0,125	Levansucrase inhibitor
0,154	0,087	Phosphatidate phosphatase inhibitor
0,247	0,180	Cell adhesion molecule inhibitor
0,103	0,037	Galactosylgalactosylglucosylceramidase inhibitor
0,143	0,077	Psychosexual dysfunction treatment
0,110	0,044	Acetylcholine M2 receptor agonist
0,118	0,052	Chondroitin 6-sulfotransferase inhibitor
0,128	0,062	ATP adenylyltransferase inhibitor
0,079	0,013	DNA directed RNA polymerase stimulant
0,122	0,057	(S)-carnitine 3-dehydrogenase inhibitor
0,079	0,014	Sucrose-phosphate phosphatase inhibitor
0,089	0,024	Protein 30S ribosomal subunit inhibitor
0,259	0,194	Mycothiol-S-conjugate amidase inhibitor
0,165	0,100	Crotonoyl-[acyl-carrier-protein] hydratase inhibitor
0,156	0,091	Arylamine N-acetyltransferase inhibitor
0,186	0,121	Hemostatic
0,098	0,034	Phosphorylase kinase inhibitor
0,168	0,104	Fibromyalgia syndrome treatment
0,095	0,031	3-Dehydroquinate dehydratase inhibitor
0,123	0,059	Glycoprotein 3-alpha-L-fucosyltransferase inhibitor
0,143	0,079	Sepsis treatment
0,111	0,048	Phosphofructokinase-1 inhibitor
0,179	0,116	Acyl-CoA oxidase inhibitor
0,221	0,158	CYP1A inhibitor
0,202	0,138	Skeletal muscle relaxant
0,133	0,070	Thiamine-phosphate kinase inhibitor

0,066	0,003	Alpha 1L adrenoreceptor antagonist
0,083	0,020	ATP diphosphatase inhibitor
0,117	0,054	Antispirochetal
0,164	0,101	CTGF expression inhibitor
0,249	0,186	Histamine release inhibitor
0,143	0,081	Shikimate 5-dehydrogenase inhibitor
0,141	0,078	3-Carboxyethylcatechol 2,3-dioxygenase inhibitor
0,205	0,142	Chloride channel blocker
0,204	0,142	Pyruvate decarboxylase inhibitor
0,175	0,113	Peptidyl-dipeptidase B inhibitor
0,112	0,049	Thermomycolin inhibitor
0,096	0,034	11-Cis-retinyl-palmitate hydrolase inhibitor
0,077	0,015	Biotin-[methylmalonyl-CoA-carboxytransferase] ligase inhibitor
0,130	0,069	Alcohol dehydrogenase substrate
0,110	0,048	Valine dehydrogenase (NADP+) inhibitor
0,096	0,034	3(or 17)beta-hydroxysteroid dehydrogenase inhibitor
0,191	0,130	L-threonine 3-dehydrogenase inhibitor
0,107	0,046	Leukocyte elastase inhibitor
0,121	0,060	Glucarate dehydratase inhibitor
0,102	0,041	Cellobiose phosphorylase inhibitor
0,125	0,065	Isovaleryl-CoA dehydrogenase inhibitor
0,109	0,048	Tyrosine-protein kinase TYRO 10 inhibitor
0,195	0,135	Arylmalonate decarboxylase inhibitor
0,081	0,021	UGT2B20 substrate
0,088	0,028	Peptidoglycan beta-N-acetylmuramidase inhibitor
0,127	0,068	Cathepsin H inhibitor
0,156	0,096	Dolichol kinase inhibitor
0,145	0,086	Homoserine dehydrogenase inhibitor
0,103	0,043	T cell inhibitor
0,092	0,033	Amylo-alpha-1,6-glucosidase inhibitor
0,098	0,039	CYP26 substrate
0,128	0,069	(S)-2-hydroxy-acid oxidase inhibitor
0,137	0,079	Hepatocyte nuclear factor antagonist
0,137	0,079	Hepatocyte nuclear factor 4 alpha antagonist
0,134	0,075	Alpha-Methylacyl-CoA racemase inhibitor
0,102	0,043	Fructuronate reductase inhibitor
0,102	0,043	Tagaturonate reductase inhibitor

0,233	0,175	Diuretic inhibitor
0,128	0,070	Pancreatic endopeptidase E inhibitor
0,188	0,130	Endopeptidase La inhibitor
0,072	0,014	CYP17 substrate
0,168	0,110	Ornithine cyclodeaminase inhibitor
0,111	0,053	Poly(3-hydroxybutyrate) depolymerase inhibitor
0,150	0,092	Isopenicillin-N synthase inhibitor
0,175	0,118	Cyclohexyl-isocyanide hydratase inhibitor
0,120	0,063	Bronchodilator
0,178	0,121	Protein-synthesizing GTPase inhibitor
0,110	0,055	Sorbose dehydrogenase inhibitor
0,100	0,044	Hair growth stimulant
0,109	0,054	Adenylylsulphatase inhibitor
0,070	0,015	CYP24 substrate
0,206	0,151	Methylumbelliferyl-acetate deacetylase inhibitor
0,073	0,018	Glycine N-choloyltransferase inhibitor
0,194	0,139	Trimethylamine dehydrogenase inhibitor
0,117	0,062	Adenylate cyclase V inhibitor
0,165	0,111	Free radical scavenger
0,206	0,152	N-benzyloxycarbonylglycine hydrolase inhibitor
0,186	0,132	Coccolysin inhibitor
0,101	0,047	UDP-N-acetylglucosamine diphosphorylase inhibitor
0,068	0,014	Microtubule stabilization
0,155	0,101	Tentoxilysin inhibitor
0,148	0,095	3-Hydroxy-4-oxoquinoline 2,4-dioxygenase inhibitor
0,126	0,073	Allantoinase inhibitor
0,185	0,133	Arylesterase inhibitor
0,158	0,106	CYP4A2 substrate
0,124	0,072	Glutamate-1-semialdehyde 2,1-aminomutase inhibitor
0,155	0,103	Procollagen N-endopeptidase inhibitor
0,074	0,021	Glucan 1,4-alpha-glucosidase inhibitor
0,208	0,156	Farnesoid X receptor antagonist
0,076	0,024	D-Serine ammonia-lyase inhibitor
0,090	0,038	Paget's disease treatment
0,071	0,019	Amylosucrase inhibitor
0,138	0,086	Phenylacetate-CoA ligase inhibitor
0,122	0,070	Acyl-lysine deacylase inhibitor

0,137	0,085	Heat shock protein agonist
0,128	0,077	Succinic dehydrogenase inhibitor
0,222	0,171	CYP1A2 substrate
0,205	0,155	DNA-(apurinic or apyrimidinic site) lyase inhibitor
0,130	0,079	Mannan endo-1,6-alpha-mannosidase inhibitor
0,103	0,052	CYP1B1 inhibitor
0,184	0,133	1-Alkylglycerophosphocholine O-acetyltransferase inhibitor
0,202	0,152	NADPH-ferrihemoprotein reductase inhibitor
0,071	0,021	Deoxyadenosine kinase inhibitor
0,108	0,058	Choline dehydrogenase inhibitor
0,100	0,050	Acetylcholine nicotinic antagonist
0,078	0,029	Protein kinase C mu inhibitor
0,201	0,152	ATPase stimulant
0,138	0,089	Trans-pentaprenyltranstransferase inhibitor
0,084	0,035	3-Dehydroquinate synthase inhibitor
0,096	0,048	Heat shock protein 90 antagonist
0,118	0,070	Sulfate adenylyltransferase (ADP) inhibitor
0,060	0,012	Androst-4-ene-3,17-dione monooxygenase inhibitor
0,158	0,110	(S)-3-amino-2-methylpropionate transaminase inhibitor
0,092	0,045	[hydroxymethylglutaryl-CoA reductase (NADPH)] kinase inhibitor
0,159	0,112	Gastritis treatment
0,054	0,006	ATPase (Vacuolar H+) inhibitor
0,094	0,046	2-Methyleneglutarate mutase inhibitor
0,097	0,050	Galactosylceramide sulfotransferase inhibitor
0,144	0,097	Polynucleotide 5'-hydroxy-kinase inhibitor
0,173	0,126	Aspartate-ammonia ligase inhibitor
0,117	0,070	Asparagine-tRNA ligase inhibitor
0,070	0,023	Antibiotic Aminoglycoside-like
0,108	0,060	Omega-amidase inhibitor
0,094	0,047	Arabinose isomerase inhibitor
0,124	0,077	Nitric oxide antagonist
0,090	0,044	Malate dehydrogenase (NADP+) inhibitor
0,196	0,150	Myosin ATPase inhibitor
0,101	0,054	Glutamine-pyruvate transaminase inhibitor
0,092	0,045	Shock treatment
0,111	0,065	Liver cirrhosis treatment
0,207	0,162	Arylsulfate sulfotransferase inhibitor

0,140	0,094	GDP-mannose 6-dehydrogenase inhibitor
0,181	0,135	Dactylysin inhibitor
0,090	0,045	Diacylglycerol O-acyltransferase inhibitor
0,180	0,135	Carbon-monoxide dehydrogenase inhibitor
0,088	0,042	Amino-acid racemase inhibitor
0,098	0,053	Inositol-polyphosphate 5-phosphatase inhibitor
0,089	0,045	2-Methylcitrate synthase inhibitor
0,191	0,146	Anti-Helicobacter pylori
0,071	0,026	Opioid mu 1 receptor antagonist
0,121	0,077	Valine decarboxylase inhibitor
0,115	0,070	Carboxymethylenebutenolidase inhibitor
0,068	0,024	Glucan 1,3-alpha-glucosidase inhibitor
0,084	0,040	Glutaminyl-tRNA synthase (glutamine-hydrolysing) inhibitor
0,101	0,057	Beta amyloid protein antagonist
0,160	0,116	Glycerol 2-dehydrogenase (NADP+) inhibitor
0,094	0,050	3-Deoxy-8-phosphooctulonate synthase inhibitor
0,103	0,059	Malonate-semialdehyde dehydrogenase inhibitor
0,064	0,020	Protein kinase C nu inhibitor
0,113	0,069	Aspartate kinase inhibitor
0,062	0,018	CYP3A11 substrate
0,088	0,045	Deoxycytidine kinase inhibitor
0,076	0,033	[acetyl-CoA carboxylase] kinase inhibitor
0,126	0,083	5 Hydroxytryptamine 2A agonist
0,182	0,139	Leukotriene-B4 20-monooxygenase inhibitor
0,166	0,123	Dimethylmaleate hydratase inhibitor
0,205	0,162	Venom exonuclease inhibitor
0,075	0,032	Malate synthase inhibitor
0,125	0,082	N-acetylneuraminate synthase inhibitor
0,085	0,043	Cyanate hydratase inhibitor
0,053	0,012	CYP4F8 substrate
0,190	0,148	Gamma-glutamyltransferase inhibitor
0,087	0,046	2-Methylcitrate dehydratase inhibitor
0,100	0,060	Creatinase inhibitor
0,117	0,077	O-acetylhomoserine aminocarboxypropyltransferase inhibitor
0,102	0,061	tRNA (cytosine-5-)-methyltransferase inhibitor
0,156	0,117	Endopeptidase inhibitor
0,065	0,026	3'(2'),5'-Bisphosphate nucleotidase inhibitor

0,130	0,091	Glutamin-(asparagin-)ase inhibitor
0,082	0,044	Propanediol dehydratase inhibitor
0,081	0,042	2-Dehydro-3-deoxy-phosphogluconate aldolase inhibitor
0,146	0,108	Hydroxymethylglutaryl-CoA lyase inhibitor
0,175	0,137	Uroporphyrinogen-III synthase inhibitor
0,101	0,063	UDP-glucose 4-epimerase inhibitor
0,068	0,030	1-Alkyl-2-acetylglycerol O-acyltransferase inhibitor
0,122	0,085	FMN reductase inhibitor
0,092	0,054	UDP-N-acetylglucosamine 2-epimerase inhibitor
0,054	0,017	NADPH oxidoreductase inhibitor
0,053	0,015	Dichloromethane dehalogenase inhibitor
0,068	0,030	NMDA receptor polyamine site agonist
0,070	0,033	Chitin deacetylase inhibitor
0,067	0,030	Lipocortins synthesis agonist
0,107	0,070	Serine 3-dehydrogenase inhibitor
0,095	0,058	Glycerophosphocholine cholinephosphodiesterase inhibitor
0,193	0,156	Monoamine uptake inhibitor
0,051	0,014	Adenylate cyclase I inhibitor
0,097	0,060	Monocarboxylic acid transporter 1 inhibitor
0,097	0,060	Monocarboxylic acid transporter inhibitor
0,083	0,046	Vitamin
0,130	0,093	Adenylate kinase inhibitor
0,118	0,082	Cholinergic
0,102	0,066	CYP1A3 substrate
0,101	0,065	Glycoprotein-fucosylgalactoside alpha-N-acetylgalactosaminyltransferase inhibitor
0,093	0,057	Beta-N-acetylgalactosaminidase inhibitor
0,118	0,082	3-Isopropylmalate dehydratase inhibitor
0,078	0,043	N-acetylglucosaminyldiphosphoundecaprenol N-acetyl-beta-D-mannosaminyltransferase inhibitor
0,110	0,075	Mevalonate kinase inhibitor
0,127	0,091	Dihydrouracil dehydrogenase (NAD+) inhibitor
0,068	0,033	Beta galactosidase inhibitor
0,106	0,071	L-galactonolactone oxidase inhibitor
0,064	0,029	CMP-KDO synthase inhibitor
0,119	0,084	Radical formation agonist
0,136	0,101	Guanylate cyclase 1 stimulant
0,110	0,076	Methionine decarboxylase inhibitor
0,078	0,044	(S)-2-Methylmalate dehydratase inhibitor

0,077	0,044	CYP2C2 substrate
0,047	0,013	Cholesterol absorption inhibitor
0,132	0,098	Choline-sulfatase inhibitor
0,050	0,017	Electron transport complex I inhibitor
0,096	0,062	Thiamine-phosphate diphosphorylase inhibitor
0,116	0,083	Nav1.3 sodium channel blocker
0,092	0,059	2-Hydroxy-3-oxopropionate reductase inhibitor
0,121	0,088	Ca2+/calmodulin-dependent kinase I inhibitor
0,063	0,031	Chalcone isomerase inhibitor
0,082	0,049	S-methyl-5-thioribose kinase inhibitor
0,034	0,002	Actin polymerization inhibitor
0,162	0,130	Gamma-D-Glutamyl-meso-diaminopimelate peptidase inhibitor
0,102	0,070	Site-specific DNA-methyltransferase (adenine-specific) inhibitor
0,077	0,046	4a-Hydroxytetrahydrobiopterin dehydratase inhibitor
0,083	0,052	2-Oxoaldehyde dehydrogenase (NAD+) inhibitor
0,184	0,153	Apoptosis antagonist
0,166	0,135	Opheline kinase inhibitor
0,166	0,135	Taurocyamine kinase inhibitor
0,094	0,063	Alpha,alpha-trehalase inhibitor
0,062	0,031	Biotin-[propionyl-CoA-carboxylase (ATP-hydrolysing)] ligase inhibitor
0,167	0,137	Ferredoxin hydrogenase inhibitor
0,115	0,085	Diisopropyl-fluorophosphatase inhibitor
0,081	0,051	Glycosylceramidase inhibitor
0,051	0,021	Pregnane X receptor antagonist
0,050	0,020	Orotidylate decarboxylase inhibitor
0,103	0,073	Glucose 1-dehydrogenase inhibitor
0,146	0,116	Uterine stimulant
0,095	0,066	Gluconate dehydratase inhibitor
0,177	0,147	Wound healing agent
0,125	0,095	Obsessive-compulsive disorder treatment
0,191	0,162	Pancreatic ribonuclease inhibitor
0,088	0,059	Alcohol oxidase inhibitor
0,055	0,026	HCV NS3/NS4A protease inhibitor
0,182	0,153	ATP phosphoribosyltransferase inhibitor
0,100	0,071	Sarcosine oxidase inhibitor
0,079	0,050	N-acetylglucosamine-6-phosphate deacetylase inhibitor
0,139	0,110	Antimutagenic

0,168	0,139	Antiviral (Hepatitis B)
0,099	0,070	Antifungal enhancer
0,089	0,061	Neuropeptide FF agonist
0,089	0,061	Neuropeptide FF2 agonist
0,136	0,107	Benzoylformate decarboxylase inhibitor
0,170	0,142	Glycine amidinotransferase inhibitor
0,098	0,070	Aerobactin synthase inhibitor
0,110	0,083	Malate-CoA ligase inhibitor
0,067	0,039	Phosphoglucomutase inhibitor
0,090	0,063	2-Aminoethylphosphonate-pyruvate transaminase inhibitor
0,075	0,048	6-Phosphofructo-2-kinase inhibitor
0,078	0,051	Nav1.7 sodium channel blocker
0,169	0,142	Nardilysin inhibitor
0,104	0,077	Phosphonoacetate hydrolase inhibitor
0,037	0,010	GRP78 expression inhibitor
0,064	0,037	Alpha-1,6-mannosyl-glycoprotein 2-beta-N-acetylglucosaminyltransferase inhibitor
0,084	0,058	Interleukin 1a antagonist
0,030	0,004	Antiprotozoal activity enhancer
0,096	0,070	Glycine receptor antagonist
0,182	0,156	Methane monooxygenase inhibitor
0,067	0,041	GMP synthase inhibitor
0,034	0,008	CYP27A1 inhibitor
0,103	0,077	Lactaldehyde reductase (NADPH) inhibitor
0,040	0,014	Postcoital contraceptive
0,135	0,110	Lactate 2-monooxygenase inhibitor
0,081	0,055	Neolactotetraosylceramide alpha-2,3-sialyltransferase inhibitor
0,101	0,076	Acetylcholine release stimulant
0,043	0,018	CYP7B substrate
0,043	0,018	CYP7B1 substrate
0,091	0,066	Mannonate dehydratase inhibitor
0,120	0,096	Diphosphomevalonate decarboxylase inhibitor
0,073	0,049	Serine-tRNA ligase inhibitor
0,058	0,034	Prostaglandin-I synthase inhibitor
0,081	0,058	Heat shock protein 70 antagonist
0,160	0,136	Meprin B inhibitor
0,039	0,015	Alpha-1,3-mannosyl-glycoprotein 2-beta-N-acetylglucosaminyltransferase inhibitor
0,065	0,041	Thioether S-methyltransferase inhibitor

0,028	0,005	Rotamase (FKBP12) inhibitor
0,132	0,109	Acetate kinase inhibitor
0,104	0,080	2-Aminoadipate transaminase inhibitor
0,191	0,167	Ferredoxin-NAD+ reductase inhibitor
0,191	0,167	Naphthalene 1,2-dioxygenase inhibitor
0,213	0,190	Nicotinate dehydrogenase inhibitor
0,111	0,088	Aryl-alcohol dehydrogenase inhibitor
0,198	0,175	Hypolipemic
0,085	0,062	2-Dehydro-3-deoxy-L-arabinonate dehydratase inhibitor
0,073	0,051	Hydroperoxide dehydratase inhibitor
0,067	0,044	UDP-glucose 6-dehydrogenase inhibitor
0,033	0,011	Cyclomaltodextrin glucanotransferase inhibitor
0,054	0,032	Antifungal (Pneumocystis)
0,080	0,058	Isocitrate lyase inhibitor
0,087	0,066	Contraceptive male
0,027	0,005	Calcineurin inhibitor
0,070	0,048	Estrogen agonist
0,094	0,073	1-Aminocyclopropane-1-carboxylate deaminase inhibitor
0,098	0,077	Sulfotransferase substrate
0,043	0,021	Glycolipid 2-alpha-mannosyltransferase inhibitor
0,111	0,089	3-Oxoadipate enol-lactonase inhibitor
0,086	0,065	Dichloromuconate cycloisomerase inhibitor
0,196	0,174	FMO1 substrate
0,073	0,052	Dihydrolipoamide S-acetyltransferase inhibitor
0,107	0,086	Heat shock protein 70 agonist
0,094	0,073	Cytochrome-c3 hydrogenase inhibitor
0,092	0,071	Homocysteine desulfhydrase inhibitor
0,071	0,050	Sphingosine kinase inhibitor
0,039	0,019	Membrane-oligosaccharide glycerophosphotransferase inhibitor
0,049	0,029	N-acetylglucosaminyldiphosphodolichol N-acetylglucosaminyltransferase inhibitor
0,218	0,198	Endopeptidase So inhibitor
0,081	0,061	CMP-N-acylneuraminate phosphodiesterase inhibitor
0,085	0,066	CTP synthase inhibitor
0,110	0,091	Protein-tyrosine sulfotransferase inhibitor
0,108	0,089	Pyruvate dehydrogenase inhibitor
0,168	0,149	O-aminophenol oxidase inhibitor
0,094	0,076	Adenosylmethionine-8-amino-7-oxononanoate transaminase inhibitor

0,092	0,073	Glycerol-3-phosphate dehydrogenase (NAD+) inhibitor
0,090	0,071	Malate dehydrogenase (oxaloacetate-decarboxylating) inhibitor
0,097	0,078	Malyl-CoA lyase inhibitor
0,098	0,080	NMDA receptor polyamine site antagonist
0,164	0,146	Diuretic
0,063	0,045	Beta-1,4-mannosyl-glycoprotein 4-beta-N-acetylglucosaminyltransferase inhibitor
0,082	0,065	Proline dehydrogenase inhibitor
0,050	0,032	UDP-N-acetylglucosamine-lysosomal-enzyme N-acetylglucosaminephosphotransferase inhibitor
0,112	0,094	Acylglycerone-phosphate reductase inhibitor
0,144	0,126	Metallocarboxypeptidase D inhibitor
0,038	0,020	Cortisol sulfotransferase inhibitor
0,232	0,215	5 Hydroxytryptamine release inhibitor
0,071	0,054	Phosphoribulokinase inhibitor
0,104	0,087	2-Oxoisovalerate dehydrogenase (acylating) inhibitor
0,106	0,089	Excitatory amino acid transporter EAAC1 inhibitor
0,114	0,097	Galactoside O-acetyltransferase inhibitor
0,101	0,085	Iduronate-2-sulfatase inhibitor
0,118	0,102	Glycine receptor agonist
0,117	0,100	Candidapepsin inhibitor
0,088	0,072	D-glutamate oxidase inhibitor
0,175	0,159	Hepatic disorders treatment
0,078	0,062	Homospermidine synthase inhibitor
0,077	0,061	N-acetylneuraminate lyase inhibitor
0,059	0,043	Shaker potassium channel blocker
0,055	0,039	Hydroxylysine kinase inhibitor
0,067	0,052	Antihypermotility
0,057	0,042	Plus-end-directed kinesin ATPase inhibitor
0,157	0,141	Thiol oxidase inhibitor
0,112	0,097	Aldehyde dehydrogenase (NAD+) inhibitor
0,060	0,045	Methylthioadenosine nucleosidase inhibitor
0,091	0,076	Glutamate (mGluR6) antagonist
0,186	0,171	Choline-phosphate cytidylyltransferase inhibitor
0,028	0,013	Cholesterol ester transfer protein antagonist
0,076	0,061	[myelin basic protein]-arginine N-methyltransferase inhibitor
0,044	0,029	Thymidine-triphosphatase inhibitor
0,108	0,093	Aminocarboxymuconate-semialdehyde decarboxylase inhibitor
0,050	0,035	Acetylcholine M1 receptor agonist

0,064	0,049	Parathyroid hormone antagonist
0,035	0,021	Methionyl aminopeptidase 2 inhibitor
0,087	0,072	Granulocyte macrophage colony stimulating factor agonist
0,084	0,069	CYP2B4 substrate
0,035	0,021	Protein phosphatase 2A inhibitor
0,025	0,011	Dopamine D2B antagonist
0,037	0,023	Glucose-6-phosphate isomerase inhibitor
0,073	0,059	Polyamine biosynthesis inhibitor
0,038	0,024	Endo-beta-N-acetylglucosaminidase inhibitor
0,142	0,128	Prenyl-diphosphatase inhibitor
0,119	0,106	Granzyme A inhibitor
0,063	0,050	Glycerol kinase inhibitor
0,165	0,152	Hydroxylamine reductase inhibitor
0,125	0,111	MDM2 inhibitor
0,099	0,086	Decylcitrate synthase inhibitor
0,132	0,119	Glutathione reductase inhibitor
0,027	0,013	Antihyperaldosteronism
0,046	0,034	Aromatase inhibitor
0,132	0,119	Antileprosy
0,042	0,029	ADAM10 endopeptidase inhibitor
0,044	0,032	Acetylcholine M4 receptor agonist
0,123	0,110	tRNA nucleotidyltransferase inhibitor
0,063	0,050	CYP2A13 substrate
0,111	0,099	Iron-cytochrome-c reductase inhibitor
0,168	0,155	TRPA1 agonist
0,139	0,127	2,6-Dihydroxypyridine 3-monooxygenase inhibitor
0,119	0,107	Vomilenine glucosyltransferase inhibitor
0,120	0,108	Sphinganine-1-phosphate aldolase inhibitor
0,046	0,034	Dopamine transporter inhibitor
0,116	0,104	HIV-2 reverse transcriptase inhibitor
0,038	0,026	Glucosylceramidase inhibitor
0,066	0,054	Cellulose synthase (UDP-forming) inhibitor
0,025	0,013	CYP27A substrate
0,104	0,093	Nav1.2 sodium channel blocker
0,022	0,011	Ketosteroid monooxygenase inhibitor
0,183	0,172	CYP2C3 substrate
0,038	0,027	Tartrate decarboxylase inhibitor

0,028	0,017	Alpha glucosidase I inhibitor
0,044	0,033	D(-)-tartrate dehydratase inhibitor
0,055	0,045	Urate transporter 1 inhibitor
0,055	0,045	1-Phosphofructokinase inhibitor
0,075	0,064	tRNA (guanine-N2-)-methyltransferase inhibitor
0,024	0,013	Alpha galactosidase inhibitor
0,066	0,055	Lactate-malate transhydrogenase inhibitor
0,136	0,126	Penicillin amidase inhibitor
0,120	0,110	Mitochondrial intermediate peptidase inhibitor
0,070	0,060	Trans-octaprenyltranstransferase inhibitor
0,358	0,348	Gluconate 2-dehydrogenase (acceptor) inhibitor
0,057	0,047	Quinate 5-dehydrogenase inhibitor
0,052	0,043	Protein N-acetylglucosaminyltransferase inhibitor
0,094	0,084	Guanidinodeoxy-scyllo-inositol-4-phosphatase inhibitor
0,064	0,055	Aspartate 1-decarboxylase inhibitor
0,021	0,012	CYP17A substrate
0,049	0,039	Tubulin GTPase inhibitor
0,078	0,069	D-malate dehydrogenase (decarboxylating) inhibitor
0,095	0,086	Photinus-luciferin 4-monooxygenase (ATP-hydrolysing) inhibitor
0,016	0,007	Kanamycin kinase inhibitor
0,020	0,012	Antibiotic Macrolide-like
0,220	0,211	CDP-diacylglycerol-serine O-phosphatidyltransferase inhibitor
0,123	0,115	Antidiabetic (type 1)
0,093	0,085	Pyrimidine-deoxynucleoside 2'-dioxygenase inhibitor
0,081	0,073	Astringent
0,052	0,043	Sucrose alpha-glucosidase inhibitor
0,049	0,041	D-lactate-2-sulfatase inhibitor
0,134	0,126	Malate dehydrogenase inhibitor
0,079	0,071	Nucleotide diphosphatase inhibitor
0,083	0,075	Aspartate transaminase inhibitor
0,104	0,096	Thiol S-methyltransferase inhibitor
0,021	0,013	CYP27 substrate
0,106	0,098	Selenocysteine lyase inhibitor
0,047	0,039	AMP nucleosidase inhibitor
0,074	0,066	3-Isopropylmalate dehydrogenase inhibitor
0,022	0,014	NMDA receptor phencyclidine site antagonist
0,029	0,021	Melatonin 2 agonist

0,069	0,061	Aspartate carbamoyltransferase inhibitor
0,074	0,066	Ribulose-bisphosphate carboxylase inhibitor
0,074	0,066	Succinate-hydroxymethylglutarate CoA-transferase inhibitor
0,010	0,002	Antibiotic Tribactam-like
0,020	0,013	Progesterone receptor B1 antagonist
0,032	0,025	Steroid-like
0,056	0,049	Beta-fructofuranosidase inhibitor
0,124	0,117	Alkylglycerone-phosphate synthase inhibitor
0,093	0,086	L-lysine 6-transaminase inhibitor
0,115	0,108	Dolichyl-phosphate beta-D-mannosyltransferase inhibitor
0,067	0,061	Carbamoyl-phosphate synthase (ammonia) inhibitor
0,016	0,009	Antiinflammatory steroid
0,125	0,119	Dependence treatment
0,073	0,066	Cysteine transaminase inhibitor
0,092	0,086	Nav1.1 sodium channel blocker
0,106	0,100	Allophanate hydrolase inhibitor
0,042	0,036	Glucose-1-phosphate adenylyltransferase inhibitor
0,120	0,114	Tetrahydroxynaphthalene reductase inhibitor
0,079	0,073	Hydroxymethylglutaryl-CoA reductase inhibitor
0,054	0,049	Androgen antagonist
0,042	0,037	Arginine-tRNA ligase inhibitor
0,059	0,053	Hot flush treatment
0,039	0,033	Glucose-1-phosphatase inhibitor
0,034	0,028	Ribose-5-phosphate adenylyltransferase inhibitor
0,046	0,040	Nucleoside deoxyribosyltransferase inhibitor
0,017	0,012	CYP11B1 substrate
0,140	0,135	Serine-pyruvate transaminase inhibitor
0,193	0,188	CYP1A2 inhibitor
0,089	0,084	Nicotinic alpha3beta4 receptor agonist
0,071	0,066	Phosphoenolpyruvate carboxykinase (diphosphate) inhibitor
0,055	0,050	Phosphodiesterase IV inhibitor
0,139	0,135	5 Hydroxytryptamine 7 agonist
0,041	0,036	Fructose 5-dehydrogenase (NADP+) inhibitor
0,043	0,038	Polyphosphate kinase inhibitor
0,049	0,045	Uracil phosphoribosyltransferase inhibitor
0,077	0,073	PRL phosphatase inhibitor
0,104	0,100	Beta-alanine-pyruvate transaminase inhibitor

0,104	0,100	Galactose 1-dehydrogenase (NADP+) inhibitor
0,041	0,037	Neuraminidase inhibitor
0,041	0,038	Chorismate mutase inhibitor
0,029	0,025	Transaldolase inhibitor
0,085	0,081	SULT1A1 substrate
0,061	0,057	4-Hydroxy-4-methyl-2-oxoglutarate aldolase inhibitor
0,029	0,026	Homoisocitrate dehydrogenase inhibitor
0,073	0,070	[acyl-carrier-protein] S-malonyltransferase inhibitor
0,073	0,070	L-lysine oxidase inhibitor
0,079	0,075	Lysine decarboxylase inhibitor
0,167	0,164	DNA-3-methyladenine glycosylase I inhibitor
0,062	0,059	Deoxyhypusine synthase inhibitor
0,169	0,167	Cis-1,2-dihydro-1,2-dihydroxynaphthalene dehydrogenase inhibitor
0,196	0,194	FMO3 substrate
0,051	0,048	Acidifying agent gastric
0,087	0,084	Pantothenase inhibitor
0,094	0,091	Dethiobiotin synthase inhibitor
0,061	0,059	Purine biosynthesis inhibitor
0,108	0,106	VEGF expression inhibitor
0,009	0,007	Vanilloid 3 antagonist
0,112	0,110	Peptidylamidoglycolate lyase inhibitor
0,065	0,063	Glycerophosphocholine phosphodiesterase inhibitor
0,102	0,101	Prepilin peptidase inhibitor
0,102	0,101	Haloacetate dehalogenase inhibitor
0,056	0,054	Prokinetic
0,059	0,057	Isocitrate dehydrogenase (NADP+) inhibitor
0,044	0,043	Glutamate uptake inhibitor
0,019	0,019	Cortisone alpha-reductase inhibitor
0,136	0,135	Aldehyde dehydrogenase 2 substrate
0,058	0,058	NMN nucleosidase inhibitor
0,018	0,017	2-Hydroxyacylsphingosine 1-beta-galactosyltransferase inhibitor
0,126	0,126	N-acetyl-gamma-glutamyl-phosphate reductase inhibitor

Table S15. Drug–induced gene expression profiles prediction (DIGEP–Pred). mRNA based, protein based, MCF-7 based,VCaP 6h based, VCaP 24h based, and combination predictionresults for complexes **1**–**5**. The name of each gene can be correlated with diseases, side-effects and biological pathways in Comparative Toxicogenomics Database (CTD), Mount Desert Island Biological Laboratory, Salisbury Cove, Maine (http://ctdbase.org/).

Complex 1

mRNA based prediction result

Pa	Pi	DownRegulation	Pa	Pi	UpRegulation
0.623	0.034	TAGLN	0.764	0.025	TNNT1
0.631	0.076	NSF	0.708	0.072	SQLE
0.670	0.118	TMEM194A	0.648	0.034	WIPI1
0.602	0.103	TRAF5	0.634	0.036	ASAH1
0.599	0.118	ADORA2B	0.600	0.024	GDPD3
0.599	0.118	CDCA4	0.635	0.065	MT1B
0.599	0.118	CENPJ	0.653	0.088	SFRP1
0.599	0.118	DNAJC9	0.574	0.040	INHBE
0.599	0.118	DONSON	0.610	0.105	CACNG4
0.599	0.118	HAUS8	0.610	0.105	CHRNE
0.599	0.118	MCMBP	0.599	0.118	PFKFB2
0.599	0.118	NUP155	0.599	0.118	SH3BGRL
0.599	0.118	PARP2	0.599	0.118	TRIM13
0.599	0.118	RMI1	0.508	0.028	LSS
0.599	0.118	SCARA3	0.527	0.049	FABP1
0.599	0.118	SLC16A14	0.510	0.033	PHYH
0.599	0.118	SUV39H2	0.523	0.050	FNDC4
0.518	0.075	SRPK2	0.551	0.080	CNTN1
0.592	0.150	TPM1	0.524	0.055	HPN
0.578	0.137	GSS	0.540	0.084	SERPINA3
0.520	0.104	CYP11A1	0.547	0.130	CASP2
0.524	0.168	TMSB15A	0.551	0.141	GAS6
			0.526	0.125	FAM49A
			0.513	0.133	PLXNA2

Protein based prediction result

Pa	Pi	DownRegulation	Pa	Pi	UpRegulation
0.737	0.075	CHEK1	0.626	0.077	NPPB
0.564	0.124	CAT	0.583	0.104	CD83

MCF7 based prediction result

Pa	Pi	DownRegulation	Pa	Pi	UpRegulation
0.579	0.065	C3orf52	0.596	0.087	CDKN1B
0.647	0.138	UBE2C	0.589	0.083	ZNF672

0.505	0.095	MRPL34	0.552	0.092	C11orf68
			0.548	0.122	KIAA0232
			0.542	0.116	RANGAP1
			0.536	0.111	STRN
			0.511	0.095	SCAND2P
			0.546	0.143	CLCF1
			0.506	0.113	CSRP2
			0.507	0.142	FOXC1
			0.508	0.154	WISP2
			0.547	0.206	CLDN9
			0.505	0.198	BIN1

VCAP_6h based prediction result

Pa	Pi	DownRegulation	Pa	Pi	UpRegulation
0.961	0.005	WDR11	0.972	0.004	B4GALT3
0.958	0.005	SLC4A1AP	0.968	0.005	MRPS34
0.940	0.019	MT1F	0.952	0.005	TTC13
0.939	0.018	MT1H	0.952	0.010	SARS2
0.933	0.020	EAPP	0.946	0.007	PMF1
0.888	0.020	SYNE2	0.933	0.008	TUBA4A
0.893	0.026	MT1P2	0.919	0.013	SUGP2
0.826	0.060	MT1X	0.908	0.017	DHPS
0.739	0.068	RABIF	0.896	0.013	ABCB8
0.716	0.054	EBAG9	0.667	0.092	DCTD
0.678	0.074	MTHFD2	0.607	0.097	DHX32
0.670	0.066	SLC25A44	0.547	0.097	POLE2
0.675	0.079	ZNF221	0.515	0.068	CASC3
0.644	0.086	PEG3	0.528	0.099	RRNAD1
0.630	0.081	ADH5	0.539	0.120	ST6GALNAC4
0.595	0.079	KIAA0485	0.580	0.162	ALG5
0.607	0.112	YWHAZ	0.519	0.114	EHMT2
0.642	0.149	C10ORF68	0.514	0.160	MIF
0.529	0.141	ROBO4	0.527	0.202	KIAA1324
0.539	0.178	CXORF56			
0.531	0.173	CETP			
0.511	0.208	MAGEA11			

VCAP_24h based prediction result

Pa	Pi	DownRegulation	Pa	Pi	UpRegulation
0.981	0.004	CBX1	0.985	0.005	POU2F1
0.978	0.005	SEC61G	0.984	0.005	ABHD8
0.977	0.005	RNASET2	0.982	0.005	GADD45GIP1
0.977	0.005	TALDO1	0.981	0.005	PLAC1
0.977	0.004	IFIT3	0.981	0.005	AMPD1
0.977	0.004	RGS4	0.981	0.005	SPEF1

0.976	0.004	UBE2E3	0.980	0.005	NUDT18
0.976	0.005	DAP3	0.980	0.005	ODZ3
0.976	0.005	GPBP1L1	0.980	0.005	B3GAT1
0.976	0.005	ATP6V1A	0.980	0.005	WRAP53
0.975	0.004	DMXL2	0.979	0.005	CD27
0.975	0.004	RALGAPB	0.979	0.005	IRGQ
0.975	0.004	C1ORF115	0.979	0.005	CCR9
0.974	0.004	DNAJC13	0.979	0.005	THSD7A
0.972	0.004	EPPK1	0.978	0.005	AK1
0.976	0.008	ZNF24	0.980	0.007	S100G
0.973	0.005	NEK4	0.978	0.005	UCN
0.972	0.004	DCUN1D1	0.979	0.006	GDF9
0.972	0.005	DLG5	0.979	0.006	GNRH2
0.972	0.005	HBS1L	0.976	0.005	COL9A1
0.971	0.005	BCL2L1	0.976	0.004	BCAM
0.971	0.004	DCTN4	0.976	0.005	ACTA1
0.971	0.005	RAB5B	0.977	0.005	WWC3
0.971	0.005	TRAFD1	0.975	0.004	HGSNAT
0.972	0.006	MRPL18	0.976	0.005	ATP5L
0.970	0.004	CLIP1	0.976	0.005	FAM86A
0.971	0.005	CLCN3	0.976	0.005	LDHC
0.970	0.005	TSPYL1	0.979	0.008	KRT85
0.970	0.005	FTO	0.976	0.005	USP19
0.968	0.005	NRBF2	0.975	0.005	PDE8B
0.968	0.005	SERINC1	0.975	0.005	CLTCL1
0.966	0.004	CLMN	0.975	0.004	TNNT1
0.965	0.004	IFT52	0.975	0.005	MYLPF
0.966	0.005	POGZ	0.975	0.005	CSNK1G2
0.966	0.005	ZNF148	0.974	0.005	CHST7
0.965	0.004	NACC2	0.976	0.006	MPZ
0.965	0.005	DBN1	0.975	0.006	ADAMTS12
0.964	0.004	CALML4	0.978	0.008	IFT74
0.963	0.004	RNF11	0.974	0.005	MEGF6
0.962	0.005	PTGES3	0.974	0.004	ITGB1BP3
0.962	0.005	RRAS2	0.974	0.005	PARD6A
0.962	0.005	HMP19	0.974	0.005	ARHGAP22
0.961	0.005	TBL1XR1	0.973	0.005	TMEM186
0.966	0.009	PPP1R12A	0.975	0.007	FAM155A
0.962	0.007	ARPC3	0.974	0.005	PPFIA2
0.960	0.005	TRAPPC10	0.975	0.007	CLDN17
0.957	0.004	EBLN2	0.972	0.004	COPS7B
0.960	0.007	SF3A1	0.972	0.005	GPR35
0.956	0.005	ASAP1	0.972	0.005	HMGB3P1
0.957	0.006	FAM13B	0.976	0.009	CENPO
0.954	0.005	NPTN	0.972	0.005	AURKB
0.954	0.005	COPS7A	0.974	0.007	LECT2
0.953	0.004	CORO1C	0.972	0.005	CTNNBIP1

0.946	0.004	FGF13	0.972	0.005	TTN
0.948	0.007	KCNJ15	0.974	0.007	CAMKMT
0.949	0.010	OGFR	0.972	0.005	PTPRCAP
0.942	0.007	RAF1	0.973	0.007	OR6A2
0.944	0.009	PRPSAP1	0.971	0.005	ZBTB48
0.942	0.008	TRAF4	0.973	0.007	CELA3B
0.945	0.011	NAAA	0.970	0.004	FSTL3
0.949	0.016	RWDD1	0.971	0.005	DEFA6
0.937	0.005	UTP14C	0.970	0.004	PNMT
0.941	0.009	PCNP	0.971	0.005	TFAP4
0.943	0.012	LSM3	0.970	0.005	VWA1
0.941	0.010	NDFIP1	0.974	0.008	C21ORF96
0.939	0.008	CNOT6	0.970	0.005	INSL6
0.947	0.018	CEP170	0.970	0.005	OXCT2
0.950	0.021	SS18L1	0.970	0.005	CPA2
0.935	0.007	WSB2	0.972	0.007	MYH3
0.932	0.005	SAR1B	0.970	0.005	FAM110B
0.930	0.005	FLNA	0.972	0.007	CAPN11
0.932	0.008	STX3	0.970	0.005	KIAA1644
0.927	0.004	AKR1B10	0.972	0.007	CLIP3
0.932	0.010	TMX2	0.969	0.004	CHST10
0.928	0.009	USP15	0.968	0.005	CLDN5
0.930	0.010	RBM23	0.968	0.005	ENDOU
0.924	0.005	HELZ	0.968	0.005	CBR4
0.924	0.005	BMI1	0.968	0.005	FARP2
0.929	0.010	TOM1L1	0.968	0.005	PROC
0.931	0.013	RALBP1	0.968	0.005	GRAP
0.926	0.010	EIF2B1	0.968	0.005	STIM1
0.922	0.008	MTMR4	0.968	0.005	CST5
0.929	0.016	C6ORF62	0.967	0.005	CYP4B1
0.919	0.005	ID2	0.967	0.005	ZSCAN16
0.920	0.012	NIPA2	0.969	0.007	RASL11B
0.913	0.006	FXYD3	0.967	0.005	CD300C
0.921	0.015	CNBP	0.966	0.005	CHRM4
0.910	0.006	SH3BGRL3	0.968	0.007	PGAM2
0.916	0.013	SYNE2	0.968	0.007	LOC100506469
0.911	0.017	SEC23IP	0.966	0.005	NKG7
0.908	0.013	RNF187	0.970	0.009	GIF
0.901	0.007	PUM2	0.966	0.006	LSM4
0.896	0.006	SLC35A5	0.969	0.009	UBOX5
0.900	0.010	MDH1	0.964	0.004	CACNA2D2
0.908	0.022	HSP90B1	0.964	0.005	PMCHL1
0.897	0.011	ELOVL2	0.964	0.005	KLHL25
0.894	0.014	CSNK1A1	0.964	0.005	MUC2
0.895	0.016	ZYX	0.966	0.007	CCL5
0.897	0.019	RNF114	0.966	0.007	ARHGEF16
0.892	0.016	HDGF	0.966	0.007	CDH19

0.889	0.013	MLH1	0.962	0.003	XAB2
0.891	0.018	TRAK1	0.963	0.005	ZBED5
0.891	0.024	PTPLB	0.965	0.007	RNF32
0.873	0.006	NUP43	0.963	0.005	ERCC2
0.882	0.020	DDX1	0.963	0.005	LRMP
0.885	0.023	COX6A1	0.962	0.005	RANBP17
0.886	0.025	MSL1	0.962	0.005	NACAD
0.877	0.018	IRS2	0.964	0.006	ALPP
0.883	0.029	UGCG	0.967	0.009	TUBAL3
0.858	0.005	KIAA0664L3	0.962	0.005	ETHE1
0.888	0.035	CTTN	0.964	0.007	PSORS1C2
0.877	0.025	PNPO	0.962	0.005	NRSN2
0.853	0.008	SNUPN	0.966	0.009	IFT140
0.863	0.026	PGS1	0.968	0.011	HSD3B2
0.873	0.037	PSMG2	0.967	0.010	NGB
0.863	0.031	SNX11	0.961	0.004	CHAC1
0.833	0.006	TOMM22	0.965	0.009	AKAP5
0.856	0.030	FXR1	0.970	0.014	GABRB1
0.854	0.029	TFG	0.961	0.005	RCC1
0.857	0.033	EIF3I	0.966	0.010	ZNF816
0.864	0.040	SLC39A6	0.969	0.013	LOC80054
0.864	0.041	TOMM20	0.962	0.006	ADCY8
0.841	0.023	MED24	0.960	0.005	ZNF629
0.849	0.032	UFD1L	0.960	0.005	STAP1
0.846	0.029	KATNB1	0.960	0.005	LOC100507424
0.828	0.026	IFNGR1	0.960	0.005	AHDC1
0.826	0.028	C1ORF216	0.959	0.004	SLC37A1
0.824	0.029	RPL36A	0.966	0.012	ALDH1B1
0.839	0.044	C14ORF109	0.963	0.010	GPR153
0.831	0.037	AASDHPPT	0.959	0.005	ARHGEF7
0.838	0.045	MICALL1	0.959	0.005	EDN1
0.823	0.031	EVL	0.964	0.010	CPS1-IT1
0.820	0.029	ABHD10	0.958	0.005	SLC9A3R2
0.824	0.033	ABHD2	0.961	0.008	ASAP3
0.821	0.033	RPLP1	0.958	0.005	SLC26A6
0.825	0.037	SORBS3	0.957	0.005	TPSB2
0.824	0.041	RNF220	0.957	0.005	SRRM1
0.807	0.025	C20ORF4	0.962	0.010	STOML1
0.817	0.036	ZFAND6	0.960	0.008	RGS9
0.811	0.030	ZNF32	0.959	0.007	KDM4D
0.836	0.056	C8ORF33	0.960	0.008	BIN1
0.805	0.032	RHOT1	0.955	0.003	PLEKHJ1
0.799	0.032	FTL	0.957	0.005	EBI3
0.794	0.028	HDAC3	0.956	0.005	DHRS1
0.815	0.053	SPCS1	0.955	0.004	TRAPPC2L
0.790	0.031	LTBR	0.960	0.009	PZP
0.797	0.039	PTS	0.959	0.009	CDH11

0.799	0.043	PSMA2	0.954	0.005	ANKRD49
0.800	0.047	ZNF500	0.957	0.008	DLK1
0.793	0.041	ATP5B	0.962	0.012	ODZ4
0.779	0.036	IARS	0.956	0.006	EPN3
0.774	0.040	UBR7	0.958	0.009	MYOM1
0.766	0.034	POMGNT1	0.958	0.009	DEFA5
0.773	0.041	RPL23A	0.958	0.010	FABP2
0.765	0.038	RPL19	0.952	0.004	ZNF606
0.764	0.037	RTF1	0.954	0.006	CCNJL
0.771	0.046	ARL6IP4	0.958	0.010	FBXL2
0.775	0.053	WIPF2	0.953	0.004	TAF15
0.768	0.048	NAPA	0.957	0.008	TNMD
0.742	0.028	POLD2	0.961	0.012	LCK
0.759	0.052	KDM2A	0.954	0.007	MATN4
0.744	0.040	VBP1	0.952	0.005	SMPDL3B
0.756	0.055	HRAS	0.955	0.009	ZNF93
0.737	0.046	PEX11A	0.951	0.005	CLPS
0.742	0.051	UBA2	0.958	0.012	RAG1
0.745	0.054	ARL1	0.954	0.009	FLJ11710
0.743	0.056	VEZF1	0.956	0.010	NPFFR1
0.738	0.054	RPS4X	0.951	0.005	HIST1H3F
0.717	0.054	SPAST	0.950	0.005	C22ORF29
0.714	0.054	SEH1L	0.950	0.005	SLC5A1
0.719	0.069	DAG1	0.954	0.010	CASQ2
0.723	0.078	TM2D3	0.949	0.005	ZBTB22
0.705	0.070	C19ORF42	0.955	0.011	PDHA2
0.695	0.067	DNAJC8	0.958	0.014	KIR3DL1
0.689	0.066	GOSR1	0.949	0.005	KIAA0182
0.694	0.079	ANAPC5	0.950	0.007	PDE6B
0.702	0.101	HSPH1	0.949	0.006	HGFAC
0.666	0.068	UBE2S	0.957	0.014	DPPA4
0.660	0.063	CASC3	0.953	0.010	GPR98
0.667	0.078	RPLP0	0.950	0.007	HIST1H1B
0.652	0.065	PSMD3	0.950	0.007	RHBDL1
0.641	0.056	LRRC59	0.948	0.005	HIST1H2AI
0.644	0.075	B3GNT1	0.951	0.009	MYL1
0.625	0.061	UBE2K	0.947	0.005	SCD5
0.661	0.098	C20ORF24	0.947	0.006	TNNC1
0.654	0.092	HNRNPA1	0.946	0.005	ZNF667
0.633	0.072	COPB2	0.947	0.006	SRD5A3
0.629	0.070	C12ORF52	0.960	0.020	OR1F1
0.650	0.093	PHB	0.945	0.005	CPA1
0.633	0.077	PHGDH	0.950	0.009	MEFV
0.630	0.079	WDR61	0.946	0.006	TCF21
0.627	0.091	EPRS	0.947	0.007	MYBPH
0.578	0.056	RHOA	0.950	0.010	LZTR1
0.627	0.120	STAU1	0.953	0.013	CD207

0.611	0.105	PGRMC1
0.610	0.104	CAPNS1
0.598	0.098	RPS10
0.601	0.101	KIAA0100
0.605	0.107	RPS2
0.564	0.071	PXN
0.568	0.081	SMARCD2
0.614	0.128	PSMA7
0.590	0.106	TMEM97
0.556	0.090	TSPAN3
0.530	0.064	TP53
0.577	0.113	TMEM93
0.575	0.114	OXA1L
0.562	0.101	H2BFS
0.583	0.125	LUC7L3
0.537	0.081	POP4
0.569	0.122	RPL13A
0.579	0.141	SET
0.552	0.114	NFKBIA
0.520	0.087	FOXO3
0.557	0.127	CSK
0.558	0.129	TMEM147
0.545	0.121	RGS2
0.557	0.142	NFE2L1
0.541	0.134	CLSTN1
0.558	0.160	N4BP2L2
0.540	0.148	CCT6A
0.535	0.147	STK25
0.529	0.144	ZNF146
0.510	0.126	COL9A3

0.946	0.007	L1TD1
0.948	0.009	LY6G6C
0.943	0.005	CRLF1
0.945	0.007	PRLH
0.945	0.007	S100A6
0.948	0.010	INS
0.942	0.005	MPDZ
0.955	0.018	NLRP1
0.958	0.020	FBXO40
0.945	0.007	TEKT2
0.949	0.012	MGC3771
0.942	0.005	IL37
0.946	0.009	NUP54
0.950	0.013	ALLC
0.948	0.012	PSKH1
0.945	0.009	TRAF2
0.955	0.020	SFRP5
0.943	0.007	C20ORF103
0.940	0.005	AP4M1
0.947	0.012	ZNF167
0.943	0.008	WRN
0.939	0.005	HIST1H2AM
0.942	0.008	MPV17
0.946	0.012	DSC1
0.945	0.011	PRKCG
0.947	0.014	ARSF
0.939	0.005	APOF
0.943	0.009	MAN2A1
0.945	0.011	GABRA4
0.947	0.014	ZNF197
0.943	0.010	CLDN15
0.945	0.013	HAPLN1
0.946	0.015	BAIAP2L2
0.941	0.010	HIST1H2AE
0.945	0.014	SRGAP2
0.939	0.008	SOX3
0.935	0.005	CRYBA2
0.941	0.010	SGSM3
0.939	0.008	HOXA7
0.935	0.005	HIGD1B
0.942	0.012	TIMM44
0.941	0.011	NCDN
0.935	0.005	NAT6
0.945	0.015	GDF2
0.947	0.017	CIDEA
0.948	0.018	SEMA6B
0.947	0.018	TCL1B

	0.943	0.014	REG1B
	0.943	0.014	ADAM11
	0.940	0.011	ZNF362
	0.943	0.014	ADA
	0.935	0.006	NGLY1
	0.933	0.005	PLEKHA8P1
	0.937	0.009	PDCD1LG2
	0.942	0.014	TNF
	0.939	0.011	OR3A3
	0.935	0.007	CELA2A
	0.943	0.015	LOC202181
	0.932	0.005	GMFG
	0.932	0.005	EPHA4
	0.931	0.005	RNF115
	0.939	0.013	CHODL
	0.940	0.014	KIR3DS1
	0.937	0.010	CD93
	0.941	0.014	GNG4
	0.935	0.009	GNA11
	0.940	0.014	KIR2DS3
	0.935	0.009	FZD10
	0.946	0.020	NRIP2
	0.950	0.025	STAB2
	0.929	0.005	RAMP2
	0.929	0.004	GNA12
	0.945	0.021	RENBP
	0.949	0.025	IL36RN
	0.931	0.008	IGF1R
	0.931	0.009	PAEP
	0.934	0.012	GPD1
	0.930	0.008	HIST1H4E
	0.935	0.013	RORC
	0.942	0.020	SAFB2
	0.931	0.009	GCG
	0.932	0.010	APEX2
	0.930	0.008	GPR19
	0.933	0.011	TASP1
	0.926	0.005	AICDA
	0.927	0.006	ACE
	0.936	0.015	GPR1
	0.935	0.014	OTC
	0.926	0.006	MMRN2
	0.929	0.008	MAP4K2
	0.934	0.015	MEP1A
	0.930	0.011	ANKRD36
	0.937	0.018	HIST1H3J
	0.931	0.012	PROX1

	0.934	0.015	HIST1H1D
	0.932	0.014	BMP5
	0.926	0.008	KCNE1L
	0.937	0.019	FSCN3
	0.926	0.008	SLMO1
	0.924	0.007	PLA2G10
	0.931	0.014	C11ORF63
	0.922	0.005	GGTLC1
	0.930	0.014	C9ORF7
	0.924	0.008	ZNF643
	0.926	0.009	ZNF324
	0.930	0.014	PKP2
	0.923	0.007	GMEB1
	0.926	0.010	MYBPC2
	0.945	0.029	ARHGDIG
	0.935	0.021	C22ORF26
	0.920	0.005	RPL13P5
	0.922	0.008	FBXL8
	0.936	0.022	PRL
	0.927	0.013	GP9
	0.928	0.015	RAB11B
	0.923	0.010	USP32
	0.926	0.014	PPY
	0.928	0.016	ADAMTS13
	0.920	0.007	CLEC11A
	0.932	0.019	INSRR
	0.921	0.009	MPO
	0.923	0.012	RXRG
	0.932	0.021	MYL2
	0.922	0.011	SAA3P
	0.919	0.008	LGI1
	0.917	0.006	GDF3
	0.934	0.024	ZNF8
	0.917	0.008	JMJD7
	0.927	0.020	RNF186
	0.928	0.020	KLRG1
	0.917	0.009	SLC9A2
	0.915	0.008	G6PC
	0.917	0.010	SLC10A2
	0.925	0.018	FRMPD1
	0.916	0.009	MMP28
	0.921	0.014	BTC
	0.921	0.015	HEG1
	0.922	0.016	CXORF27
	0.914	0.009	PPP2R5E
	0.914	0.009	SCARF1
	0.918	0.013	TMPRSS6

	0.916	0.011	ASTE1
	0.924	0.020	IL1A
	0.929	0.025	C6ORF15
	0.917	0.013	MORN1
	0.917	0.015	ALOX5AP
	0.918	0.015	LRAT
	0.915	0.013	RALA
	0.914	0.012	ZNF177
	0.906	0.005	HIST1H4C
	0.919	0.018	SCN1A
	0.921	0.019	UBR4
	0.916	0.014	VPS33A
	0.922	0.021	ESRRA
	0.920	0.019	C19ORF73
	0.920	0.019	TAS2R9
	0.907	0.007	XPO4
	0.909	0.009	ARPP21
	0.913	0.014	CACNA1S
	0.917	0.019	RERGL
	0.913	0.014	SH3TC2
	0.915	0.016	TMEM30B
	0.915	0.017	SLC35E2
	0.903	0.005	HIST1H3G
	0.919	0.021	CEBPE
	0.915	0.017	CD3G
	0.916	0.019	RPS3A
	0.909	0.012	MCPH1
	0.908	0.011	GDNF
	0.907	0.010	LRRK1
	0.917	0.020	PTPN7
	0.913	0.016	CASZ1
	0.918	0.022	ZFR2
	0.913	0.017	CYP2A13
	0.903	0.007	CNPPD1
	0.910	0.015	CAPN9
	0.914	0.018	KIR2DL4
	0.905	0.009	USP11
	0.908	0.013	MAGIX
	0.910	0.015	TRIM48
	0.920	0.025	OR2F1
	0.915	0.020	KIAA1310
	0.911	0.017	UBTD1
	0.910	0.016	GNL3LP1
	0.915	0.021	CNNM1
	0.924	0.030	NDUFB7
	0.909	0.016	GSTCD
	0.898	0.005	CCNT1

0.914	0.021	TNN
0.905	0.013	CDK20
0.913	0.021	MYH2
0.910	0.018	GPR21
0.907	0.015	CPNE7
0.908	0.016	DMRT1
0.904	0.013	C1ORF114
0.904	0.013	ZNF141
0.905	0.014	TBX21
0.899	0.008	KCNK2
0.914	0.024	ACCN1
0.911	0.021	UNC5C
0.901	0.012	FCF1
0.906	0.016	MRPL41
0.902	0.013	CFHR5
0.909	0.019	FLNC
0.908	0.019	BEGAIN
0.905	0.017	MPZL2
0.916	0.027	FGF20
0.912	0.024	TMEM59L
0.906	0.019	ANKRD1
0.899	0.012	COLEC11
0.909	0.023	RGS3
0.895	0.009	KLK14
0.910	0.024	CACNB4
0.899	0.014	KLRC3
0.910	0.025	MZB1
0.902	0.017	ACADS
0.900	0.016	LIN7A
0.909	0.026	KIR2DS2
0.902	0.019	HOXB1
0.904	0.021	ABP1
0.914	0.031	PTPRB
0.905	0.023	SSTR5
0.900	0.018	CELA3A
0.917	0.035	AQP7
0.898	0.016	NCKAP1
0.898	0.016	SLC17A2
0.908	0.027	SLCO3A1
0.913	0.031	PLA2G5
0.906	0.025	OR2S2
0.905	0.024	DNAJC4
0.898	0.017	B3GALT2
0.905	0.025	CCDC64
0.899	0.020	BACH2
0.906	0.027	ANGPTL4
0.886	0.007	SFT2D2

	0.894	0.016	KRT3
	0.902	0.024	KLHL4
	0.897	0.019	RPL3L
	0.894	0.016	CRYBB3
	0.896	0.018	TM4SF5
	0.900	0.023	TXNRD3
	0.895	0.018	PTGER1
	0.899	0.023	NPPB
	0.896	0.021	LOC400084
	0.888	0.013	EBLN2
	0.896	0.021	INSR
	0.893	0.019	IVD
	0.897	0.024	HIST1H2BB
	0.895	0.023	FLT4
	0.894	0.021	COL18A1
	0.890	0.018	FKBP6
	0.887	0.015	DDX43
	0.887	0.015	MRPL20
	0.897	0.025	RAPGEF5
	0.899	0.028	INHBC
	0.903	0.032	C1ORF38
	0.890	0.019	LRRN3
	0.881	0.011	VIP
	0.883	0.013	RGP1
	0.895	0.026	HTR1F
	0.909	0.039	LOC100288594
	0.898	0.029	GPR182
	0.892	0.023	GALNT8
	0.898	0.030	SIX3
	0.898	0.029	ECE2
	0.881	0.012	ZNF124
	0.894	0.026	ASMTL-AS1
	0.884	0.016	OVGP1
	0.892	0.025	MSTN
	0.893	0.026	SYNGR4
	0.897	0.030	TP73-AS1
	0.892	0.025	OXT
	0.890	0.024	FKSG2
	0.895	0.029	FGF14
	0.891	0.026	ITFG2
	0.886	0.020	SLC12A6
	0.889	0.024	USP27X
	0.902	0.037	RAB3D
	0.889	0.024	RFPL3-AS1
	0.898	0.033	KIR3DL3
	0.895	0.030	PYGM
	0.885	0.022	FAM164C

	0.871	0.007	CDHR1
	0.881	0.018	ARHGEF10L
	0.871	0.008	OFD1
	0.880	0.017	FAM174B
	0.887	0.024	HCG26
	0.878	0.015	DFFA
	0.888	0.026	TRPV5
	0.888	0.026	TRIM14
	0.878	0.015	FZD5
	0.879	0.017	C4ORF43
	0.885	0.023	CWH43
	0.886	0.025	RPAIN
	0.879	0.018	SEMG2
	0.877	0.016	CFB
	0.893	0.032	BRD7P3
	0.881	0.020	MMP15
	0.889	0.029	CRX
	0.893	0.034	KIAA1024
	0.888	0.028	HIST1H4A
	0.874	0.014	FKBP10
	0.884	0.024	C19ORF26
	0.881	0.021	C8ORF51
	0.887	0.028	HIST1H2AK
	0.885	0.025	NR5A1
	0.891	0.032	ESRRB
	0.874	0.016	NARS
	0.886	0.028	KCNJ1
	0.897	0.039	RHOBTB2
	0.879	0.021	LOC729799
	0.875	0.017	SGCG
	0.864	0.006	KIF18A
	0.870	0.012	PSD3
	0.882	0.025	MYH1
	0.891	0.034	PRTN3
	0.882	0.024	UHRF1BP1L
	0.882	0.025	BAIAP3
	0.874	0.018	TBC1D8
	0.870	0.014	HIST1H3C
	0.883	0.027	SLC18A1
	0.874	0.018	KIAA1614
	0.883	0.027	SP2
	0.880	0.025	TRPM8
	0.874	0.019	RETN
	0.884	0.030	FAM189A1
	0.876	0.022	CD7
	0.878	0.025	ATP4A
	0.877	0.024	ATP2A3

0.880	0.027	NUBP1
0.885	0.032	CUL2
0.887	0.034	RHBG
0.880	0.028	SLC13A4
0.879	0.028	GFRA4
0.876	0.024	GATA1
0.876	0.024	KERA
0.878	0.026	HOXA4
0.884	0.033	SNRNP70
0.881	0.029	JPH2
0.873	0.022	RUNDC2C
0.875	0.025	LY6G5C
0.876	0.026	L1CAM
0.875	0.025	SULF1
0.870	0.020	DLGAP4
0.870	0.020	CD79A
0.872	0.022	KRAS
0.874	0.025	TNFRSF10C
0.874	0.025	OR5I1
0.877	0.028	CACNA1H
0.872	0.023	WFDC1
0.872	0.024	FLJ22184
0.861	0.012	SMC5
0.883	0.035	SCAI
0.874	0.027	DPP4
0.874	0.026	CHRNB2
0.870	0.023	SYNM
0.880	0.033	GALK1
0.874	0.027	TPSAB1
0.865	0.018	PTCD1
0.871	0.025	TSPAN9
0.875	0.029	SPCS3
0.865	0.019	WSCD1
0.874	0.028	RBP3
0.861	0.014	WNT7A
0.882	0.036	LHX3
0.869	0.023	NTRK1
0.880	0.034	PKP1
0.877	0.031	PCDHA9
0.873	0.028	DOK3
0.873	0.027	P2RY6
0.869	0.024	SCN10A
0.868	0.023	MFAP3
0.870	0.026	WDR25
0.867	0.023	CORO1A
0.871	0.027	TPM3
0.880	0.037	KCNMB2

0.860	0.017	VAX2
0.868	0.026	DBF4B
0.875	0.033	MMP20
0.871	0.029	RAP1A
0.866	0.024	CNTD2
0.870	0.029	HIST1H2AB
0.867	0.027	C9ORF156
0.869	0.029	CNGA1
0.861	0.021	NEUROD1
0.870	0.030	AKAP4
0.864	0.024	C2ORF67
0.879	0.039	SNHG3
0.863	0.023	GRIK3
0.863	0.023	FOXJ1
0.862	0.023	SEMA4C
0.853	0.014	SORL1
0.873	0.034	NTN1
0.850	0.011	GADD45G
0.865	0.026	T
0.865	0.026	PCDHA10
0.870	0.032	C6ORF27
0.889	0.051	CRYBB2
0.858	0.021	HYAL3
0.861	0.024	CSRP3
0.865	0.029	CDKAL1
0.862	0.026	FGF16
0.860	0.024	MIA2
0.863	0.027	GCKR
0.880	0.045	DNASE1
0.877	0.041	CA5B
0.860	0.025	PRKY
0.859	0.024	PITPNM3
0.865	0.030	CCDC48
0.859	0.024	PAX1
0.877	0.043	MYL3
0.878	0.044	HNF4G
0.862	0.028	HIST1H3A
0.868	0.034	GLRX3
0.877	0.043	P2RX1
0.866	0.032	CYP2C19
0.858	0.024	PRSS21
0.867	0.034	KCNK13
0.873	0.039	GHRH
0.867	0.033	GLTSCR1
0.866	0.033	ALOX12B
0.859	0.027	TECTA
0.854	0.021	AQP8

	0.860	0.028	EFCAB6
	0.869	0.037	ERCC6L
	0.867	0.036	NDOR1
	0.877	0.047	FBXO42
	0.851	0.021	C10ORF92
	0.858	0.030	PDE5A
	0.868	0.040	TREX2
	0.860	0.032	SLC6A4
	0.870	0.043	APOBEC2
	0.870	0.043	HTR3A
	0.858	0.031	PRKCI
	0.852	0.025	LRRC17
	0.870	0.043	SYCP1
	0.856	0.030	VIL1
	0.858	0.031	C1ORF144
	0.840	0.014	BRIP1
	0.842	0.015	CCDC19
	0.849	0.024	PPP4R2
	0.870	0.045	C11ORF20
	0.850	0.025	EN2
	0.857	0.033	HTR6
	0.870	0.045	PCDHGB5
	0.852	0.028	FGF17
	0.843	0.019	VEGFC
	0.849	0.025	HAPLN2
	0.857	0.033	ID2B
	0.859	0.035	PRRG2
	0.857	0.033	ACTL7B
	0.856	0.032	PVRIG
	0.871	0.047	GPRC5D
	0.844	0.021	LRRC36
	0.858	0.034	KCNA4
	0.868	0.045	KLRD1
	0.861	0.038	DEFB126
	0.853	0.030	BMP3
	0.841	0.018	KCNJ15
	0.847	0.025	CDKL1
	0.858	0.036	ESPN
	0.864	0.042	ARVCF
	0.858	0.035	QPCTL
	0.844	0.022	LPA
	0.847	0.026	PRKACG
	0.855	0.034	FERMT1
	0.846	0.025	C2ORF56
	0.848	0.027	ZNF337
	0.849	0.029	FGF3
	0.843	0.023	HIST2H2AA3

	0.851	0.030	FAM120C
	0.860	0.039	SLC6A7
	0.847	0.028	RCVRN
	0.855	0.035	SCNN1G
	0.845	0.026	TAF4B
	0.856	0.037	MYCN
	0.857	0.039	IL1RL2
	0.849	0.031	MYH7
	0.838	0.021	LIPG
	0.844	0.026	KBTBD10
	0.844	0.026	CD160
	0.850	0.033	LOC729164
	0.860	0.043	MUC3A
	0.846	0.029	EXTL1
	0.841	0.025	ATP2A1
	0.848	0.032	CAV3
	0.842	0.026	ZFYVE26
	0.848	0.032	UTS2
	0.851	0.035	TPO
	0.862	0.046	HMHB1
	0.855	0.039	TRAF3IP1
	0.843	0.027	C3ORF75
	0.847	0.032	TNFAIP2
	0.831	0.016	DHX58
	0.835	0.020	HIST1H2BM
	0.851	0.037	CREB3L1
	0.847	0.033	IFNA1
	0.840	0.026	KIF26B
	0.840	0.027	ITGA9
	0.837	0.024	DYSF
	0.853	0.040	MAGOH2
	0.851	0.038	GUCA1A
	0.844	0.031	ZNF280D
	0.837	0.025	RAD54B
	0.845	0.033	FLJ13224
	0.841	0.029	DNMT3L
	0.834	0.023	HRC
	0.848	0.037	NTN3
	0.829	0.018	LEFTY2
	0.839	0.028	C20ORF195
	0.835	0.026	APLNR
	0.855	0.046	TOX
	0.844	0.035	FAM204A
	0.841	0.032	LTC4S
	0.849	0.041	HCN4
	0.842	0.034	ZNF609
	0.841	0.033	KDM6B

	0.852	0.045	CALY
	0.853	0.045	TUBB4
	0.863	0.056	PSG5
	0.845	0.038	CALB2
	0.856	0.050	KIAA1107
	0.838	0.032	HIST1H4L
	0.838	0.033	LOC51145
	0.837	0.032	HIST1H3I
	0.836	0.032	TRPC2
	0.832	0.027	P2RX3
	0.843	0.039	SLC6A16
	0.834	0.030	KRT24
	0.835	0.030	EXOSC1
	0.830	0.026	FBXW4P1
	0.842	0.037	KLRK1
	0.833	0.029	CCDC121
	0.847	0.042	TP53AIP1
	0.825	0.021	RAG2
	0.839	0.035	KLF8
	0.836	0.032	SLC26A10
	0.841	0.037	PCDHA5
	0.826	0.023	MAN1C1
	0.832	0.029	NCRNA00185
	0.834	0.031	NFASC
	0.846	0.044	RPL23AP53
	0.856	0.054	MAML3
	0.836	0.034	CLDN9
	0.853	0.052	PPP2R3A
	0.837	0.036	LAMA1
	0.852	0.051	PSPN
	0.836	0.035	PRDM16
	0.829	0.029	TTC30A
	0.853	0.054	GALR3
	0.845	0.045	TMEM80
	0.845	0.045	C11ORF16
	0.835	0.035	LCN1
	0.827	0.028	KCNC2
	0.836	0.037	ATP9B
	0.837	0.038	C6ORF10
	0.833	0.034	SIX5
	0.822	0.024	IL12B
	0.829	0.031	CINP
	0.829	0.032	HIST1H2AL
	0.841	0.044	RASL10A
	0.826	0.028	NIN
	0.821	0.024	SAGE1
	0.826	0.028	EGR4

	0.835	0.038	CA3
	0.825	0.029	FASTKD2
	0.841	0.045	IFNA16
	0.851	0.055	BTF3P11
	0.827	0.033	PSG11
	0.821	0.026	CDR1
	0.839	0.044	GPSM3
	0.841	0.046	ADORA1
	0.840	0.046	SLC9A7
	0.833	0.040	MAPK8
	0.839	0.045	CYP2W1
	0.844	0.051	NPPA
	0.809	0.015	BRD4
	0.834	0.040	ALOX12
	0.837	0.044	TNFRSF8
	0.832	0.038	TSHZ2
	0.823	0.030	MYH4
	0.815	0.023	TNFSF4
	0.822	0.029	LEP
	0.826	0.034	SLCO5A1
	0.840	0.048	GPR32
	0.841	0.049	PGK2
	0.817	0.025	UBN1
	0.831	0.040	TDRD1
	0.817	0.026	ENTPD3
	0.833	0.042	GUCY2F
	0.822	0.032	GJA3
	0.822	0.032	NAALADL1
	0.818	0.028	FOXC2
	0.812	0.022	HSD17B1
	0.809	0.020	MCF2L-AS1
	0.844	0.055	GRPR
	0.833	0.045	ANKRD53
	0.821	0.033	DNASE1L2
	0.822	0.034	IL24
	0.829	0.041	MGC4859
	0.829	0.042	MKRN3
	0.822	0.035	ETV2
	0.826	0.038	RAI1
	0.843	0.056	LOC441204
	0.828	0.042	ULK4
	0.833	0.046	TMEM143
	0.824	0.038	ZNF682
	0.820	0.034	REN
	0.816	0.030	HIST3H3
	0.826	0.040	ECE1
	0.825	0.040	UBXN1

	0.833	0.048	PRM1
	0.837	0.052	TRAV8-3
	0.825	0.041	RPLP2
	0.820	0.036	BICD1
	0.822	0.038	GNG13
	0.813	0.029	GABRR1
	0.828	0.044	PCDHB11
	0.812	0.028	MNX1
	0.831	0.048	LOC100131532
	0.822	0.039	PKDREJ
	0.832	0.049	BCL2L14
	0.843	0.060	RAPGEF1
	0.819	0.036	SOCS7
	0.822	0.039	MYOG
	0.821	0.039	DYRK2
	0.827	0.045	JMJD5
	0.820	0.038	LGR5
	0.826	0.045	TSPAN2
	0.828	0.047	NPVF
	0.824	0.043	PKI55
	0.810	0.030	CYSLTR1
	0.825	0.045	KCNK7
	0.835	0.055	FGF22
	0.813	0.034	ELP4
	0.812	0.032	ITGA1
	0.806	0.027	UCP2
	0.813	0.033	ABL2
	0.820	0.041	CD244
	0.831	0.052	TRIM46
	0.812	0.033	ATXN3L
	0.814	0.036	GALR1
	0.804	0.026	DKFZP547G183
	0.819	0.041	S1PR5
	0.829	0.051	HOXC5
	0.820	0.044	KRTAP9-9
	0.833	0.056	SLC16A8
	0.802	0.026	ATP9A
	0.807	0.031	COL5A1
	0.820	0.044	ACAP1
	0.810	0.034	OR3A1
	0.833	0.057	DEF6
	0.816	0.040	COL8A1
	0.814	0.038	DHODH
	0.806	0.030	KLRAP1
	0.811	0.036	SRSF11
	0.822	0.047	ADRB1
	0.806	0.031	C8ORF84

	0.825	0.050	TAPT1
	0.823	0.048	C9ORF53
	0.820	0.045	CELP
	0.813	0.038	NPPC
	0.806	0.032	C15ORF5
	0.833	0.059	LTK
	0.812	0.038	ZNF484
	0.814	0.040	CACNA2D1
	0.809	0.035	LSAMP
	0.800	0.027	PORCN
	0.811	0.038	PLXNA2
	0.825	0.052	NPAS1
	0.805	0.034	CYP4F2
	0.813	0.041	SMCP
	0.811	0.039	CLEC4E
	0.819	0.047	ACAA2
	0.815	0.044	ZNF442
	0.806	0.035	COL6A3
	0.803	0.032	CDX1
	0.803	0.032	ZMYND10
	0.831	0.061	CCBP2
	0.830	0.060	SPAG11B
	0.823	0.054	SLC19A3
	0.805	0.036	TNFRSF13B
	0.808	0.039	VSTM4
	0.807	0.038	PEG3
	0.807	0.038	MAP3K10
	0.813	0.044	CCDC30
	0.811	0.043	ZP2
	0.815	0.046	SATB2
	0.790	0.022	C14ORF133
	0.807	0.040	RPE65
	0.820	0.052	LGALS14
	0.826	0.058	CDSN
	0.800	0.033	POLR2J4
	0.808	0.041	CLDN11
	0.806	0.039	GPRC5B
	0.811	0.044	ANGPTL7
	0.805	0.039	KLHL23
	0.807	0.041	TMEM19
	0.805	0.040	ZNF253
	0.806	0.040	PURG
	0.807	0.041	BCMO1
	0.802	0.037	GPR132
	0.807	0.042	IL21R
	0.814	0.050	CATSPERG
	0.818	0.053	LOXL3

0.799	0.035	ZNF257
0.812	0.047	HIST1H3E
0.820	0.056	SEZ6L
0.818	0.054	STK3
0.809	0.045	PRO2012
0.791	0.028	PDZD7
0.805	0.042	PTTG2
0.800	0.038	GIPR
0.817	0.055	TGFBR1
0.799	0.038	ACTL7A
0.812	0.050	TIAL1
0.822	0.060	C11ORF41
0.790	0.028	USP53
0.810	0.049	OTUB2
0.793	0.031	C14ORF79
0.806	0.045	RIBC2
0.812	0.051	HSD17B3
0.815	0.054	EDN2
0.798	0.037	CORO7
0.817	0.057	DAZL
0.797	0.039	LRRC2
0.796	0.038	MCM3AP-AS1
0.796	0.037	CDKN2D
0.805	0.047	ATP6AP2
0.797	0.040	SPATA6
0.790	0.032	RALGAPA1
0.806	0.048	CCR8
0.806	0.049	LYZL6
0.794	0.037	SHBG
0.789	0.033	PITX2
0.807	0.051	TMEM209
0.791	0.035	KIF24
0.797	0.041	KRT36
0.806	0.050	GPR63
0.816	0.060	KCNV1
0.791	0.036	FOXD3
0.805	0.050	LMAN1L
0.800	0.046	ARSD
0.800	0.046	BPNT1
0.798	0.044	SOSTDC1
0.810	0.056	SOX15
0.784	0.030	GRRP1
0.793	0.040	C14ORF102
0.795	0.042	MEP1B
0.825	0.072	GRIK4
0.805	0.052	HHLA1
0.794	0.042	DOC2B

0.802	0.050	MAGEL2
0.803	0.051	TEX13B
0.788	0.036	PCDHGA8
0.789	0.038	SLC25A17
0.807	0.056	SCN9A
0.790	0.039	EVC
0.808	0.058	EMR3
0.788	0.038	DUSP26
0.796	0.047	UTF1
0.791	0.041	GPR124
0.814	0.065	MIR600HG
0.795	0.045	KAZALD1
0.794	0.045	NTSR2
0.790	0.041	HTR1A
0.808	0.060	ITGA4
0.788	0.039	RFXAP
0.780	0.032	CCDC87
0.804	0.057	FXYD1
0.797	0.049	ZNF528
0.799	0.052	SOX5
0.791	0.044	PLAGL2
0.791	0.044	OPRD1
0.795	0.048	ATXN7L1
0.785	0.038	WNT1
0.789	0.042	TRDN
0.796	0.050	PTPRC
0.793	0.047	ADAMTS20
0.803	0.057	DNAJC28
0.801	0.055	SCAPER
0.802	0.056	CACNA1F
0.795	0.049	GPR25
0.798	0.053	RPL35A
0.798	0.053	OR2B2
0.777	0.032	COLQ
0.788	0.044	SEMA3F
0.798	0.054	DCAF4
0.805	0.062	FZD8
0.797	0.054	CST8
0.802	0.060	BMP15
0.794	0.051	ADAM29
0.812	0.069	FAM153A
0.786	0.043	ZNF345
0.799	0.057	DUSP21
0.790	0.049	FASLG
0.793	0.051	IGH@
0.780	0.039	HAND2
0.789	0.047	ZNRF4

	0.787	0.046	INPP5D
	0.780	0.040	ZNF204P
	0.790	0.049	RNF208
	0.791	0.050	TREML2
	0.779	0.039	HIST1H4B
	0.785	0.045	TIGD6
	0.800	0.061	TLR6
	0.799	0.059	AGTR2
	0.800	0.060	MIR622
	0.795	0.055	NKX2-8
	0.784	0.045	PCDHA6
	0.778	0.040	SLC22A6
	0.796	0.057	C1ORF95
	0.789	0.050	PBRM1
	0.808	0.070	BCAT2
	0.803	0.065	GPR12
	0.806	0.069	AP2A2
	0.781	0.045	TRPC4
	0.776	0.039	EDNRA
	0.778	0.041	IKZF3
	0.788	0.052	KIAA0485
	0.791	0.055	FAM63B
	0.788	0.052	PDC
	0.790	0.054	POU4F3
	0.795	0.060	KRT84
	0.788	0.052	HOXA6
	0.780	0.045	MYEF2
	0.784	0.049	CHRNA10
	0.781	0.047	YY2
	0.773	0.038	C20ORF46
	0.779	0.044	GABRG3
	0.770	0.036	HIST1H1T
	0.780	0.046	RIC3
	0.803	0.069	EMILIN2
	0.797	0.063	RPL10
	0.791	0.057	TMEM151B
	0.782	0.048	VHL
	0.795	0.061	ST8SIA2
	0.787	0.054	SYNGR1
	0.775	0.043	IL36A
	0.773	0.040	ZNF254
	0.779	0.047	ERC2
	0.794	0.062	ANKFY1
	0.773	0.041	KRT7
	0.800	0.069	TRAF3IP3
	0.782	0.051	UCHL5
	0.799	0.068	SLC4A5

	0.774	0.043	PRDM8
	0.778	0.048	PIN1P1
	0.783	0.053	NEUROG2
	0.789	0.059	KHSRP
	0.775	0.045	RASSF8
	0.776	0.046	SP3P
	0.779	0.050	LOC257152
	0.795	0.066	SLC26A3
	0.799	0.070	DUX2
	0.769	0.040	IL19
	0.772	0.044	HIST1H2BO
	0.781	0.052	TBL1Y
	0.772	0.044	GCM1
	0.775	0.046	C7ORF54
	0.767	0.039	KIAA1704
	0.766	0.038	DDI2
	0.779	0.052	HIST1H4G
	0.772	0.045	GOLGA8A
	0.796	0.069	INSL5
	0.778	0.051	PPY2
	0.764	0.038	SH3D21
	0.785	0.058	LOC57399
	0.772	0.046	GLP2R
	0.786	0.059	INHA
	0.774	0.048	HIST1H2BN
	0.777	0.051	C8ORF60
	0.768	0.042	IL1RAPL1
	0.789	0.063	EDDM3B
	0.776	0.051	ACCN3
	0.796	0.071	FAM90A1
	0.769	0.044	RAD21L1
	0.772	0.047	FFAR3
	0.777	0.053	DIAPH3
	0.768	0.045	TONSL
	0.768	0.045	FBP2
	0.783	0.060	NIPBL
	0.771	0.048	ATP1B4
	0.758	0.035	TNIP3
	0.766	0.043	SOX18
	0.785	0.063	LOC100130741
	0.777	0.055	VPS13C
	0.775	0.052	SEC14L4
	0.758	0.036	KRT8P12
	0.776	0.054	CDX4
	0.764	0.041	PTMS
	0.768	0.046	SPTLC3
	0.768	0.046	LOC284244

	0.783	0.061	C6ORF105
	0.772	0.050	OCM2
	0.785	0.064	BIN2
	0.780	0.058	PDE6H
	0.791	0.070	NMUR1
	0.774	0.054	SNRNP200
	0.768	0.048	SCGB1D1
	0.781	0.061	PDYN
	0.769	0.050	HSF1
	0.806	0.087	CXCR1
	0.752	0.033	CCDC15
	0.774	0.055	ZNF234
	0.749	0.030	RASIP1
	0.760	0.041	ATAD5
	0.785	0.067	TAS2R3
	0.765	0.047	PLXNB2
	0.767	0.050	COX6A2
	0.778	0.061	KRT83
	0.780	0.064	SSX5
	0.781	0.065	CLEC5A
	0.749	0.033	POLI
	0.767	0.051	CXORF36
	0.757	0.041	TRA2A
	0.772	0.057	FAM76A
	0.762	0.046	HMGA1
	0.787	0.072	GCK
	0.778	0.063	WRNIP1
	0.775	0.060	FAM186A
	0.768	0.054	CASC5
	0.773	0.060	ATF6B
	0.762	0.049	MYF6
	0.777	0.064	EIF4G2
	0.770	0.057	LATS1
	0.769	0.056	ZNF74
	0.783	0.071	KCNA10
	0.757	0.045	FSCN2
	0.770	0.058	LARP7
	0.787	0.074	GK2
	0.778	0.066	SYDE1
	0.777	0.065	KIAA1751
	0.753	0.041	OVOL2
	0.775	0.064	SP4
	0.751	0.040	STK17A
	0.770	0.058	ZNF493
	0.754	0.043	BRCA2
	0.770	0.060	MYH8
	0.766	0.056	C20ORF7

	0.744	0.034	FOLR3
	0.750	0.040	SLC13A2
	0.761	0.051	BTBD18
	0.773	0.063	TAS2R8
	0.756	0.047	ZBTB6
	0.766	0.057	POMC
	0.750	0.041	PITX3
	0.757	0.048	UBE2I
	0.779	0.070	VRK3
	0.782	0.073	ITIH4
	0.758	0.050	ZNF223
	0.774	0.067	CNGB3
	0.760	0.053	TAOK2
	0.766	0.059	DOT1L
	0.778	0.072	PFKFB4
	0.775	0.069	KLK15
	0.753	0.047	LOR
	0.754	0.049	MAGEB3
	0.777	0.071	MYO3A
	0.763	0.058	GJC1
	0.759	0.055	SEMA3A
	0.766	0.061	PTH
	0.763	0.058	BTNL3
	0.751	0.047	ZNF157
	0.776	0.072	PLA2G2E
	0.770	0.067	ABCB9
	0.768	0.065	C3ORF32
	0.776	0.073	CACNG1
	0.783	0.081	GRIA1
	0.772	0.069	AVIL
	0.766	0.063	METTL19
	0.772	0.070	KIR2DS4
	0.775	0.073	GABARAPL3
	0.759	0.057	SLC13A1
	0.756	0.054	MCF2L2
	0.773	0.071	PDE3B
	0.760	0.058	DTNB
	0.745	0.044	NEUROG3
	0.768	0.066	DGKQ
	0.758	0.057	PRDM10
	0.764	0.064	HOXD12
	0.776	0.076	LINC00483
	0.745	0.045	C3ORF36
	0.743	0.042	CHML
	0.757	0.057	CARD10
	0.759	0.059	CGGBP1
	0.780	0.080	OR12D3

	0.750	0.051	OR3A2
	0.752	0.053	LHCGR
	0.767	0.068	TUBB4Q
	0.754	0.055	ZDHHC17
	0.760	0.062	CBFA2T2
	0.767	0.068	WNT3
	0.768	0.069	SDK2
	0.760	0.062	NHLH2
	0.761	0.063	MYOZ3
	0.763	0.065	PLEKHM1
	0.755	0.057	MAPK4
	0.776	0.078	RIMS1
	0.756	0.058	ROS1
	0.758	0.060	CPSF1
	0.764	0.066	C22ORF43
	0.737	0.040	P2RY10
	0.756	0.059	HGF
	0.757	0.061	AZU1
	0.757	0.061	LOC1720
	0.750	0.054	GALNT3
	0.764	0.069	METTL10
	0.753	0.058	TRIM17
	0.752	0.056	C21ORF62
	0.775	0.079	BRS3
	0.771	0.076	HCRT
	0.771	0.076	C22ORF24
	0.774	0.079	POU6F2
	0.751	0.057	NSL1
	0.760	0.066	DNAH2
	0.755	0.061	TCP11
	0.759	0.065	CYP2U1
	0.749	0.055	CNTN6
	0.752	0.059	DPH5
	0.743	0.050	GCM2
	0.752	0.059	RPL5
	0.769	0.076	MC3R
	0.752	0.060	MRPS18C
	0.752	0.060	TBC1D30
	0.759	0.067	PCDHGA11
	0.758	0.067	ZIC4
	0.745	0.054	UCP1
	0.737	0.046	WDR52
	0.751	0.060	RFC1
	0.747	0.056	EPB41L4A
	0.747	0.057	PRKD1
	0.749	0.058	KRTAP5-9
	0.745	0.055	DGKE

	0.749	0.059	CT62
	0.750	0.060	EXOC6B
	0.761	0.071	PMS2P4
	0.772	0.083	GPR3
	0.748	0.059	AGBL3
	0.758	0.068	ZNF81
	0.738	0.049	FOXL1
	0.747	0.058	CCDC33
	0.754	0.066	LOC100134713
	0.754	0.066	TSNAXIP1
	0.762	0.074	MRE11A
	0.749	0.061	MYO1A
	0.762	0.075	RPS6KA6
	0.769	0.082	LOC440792
	0.743	0.056	IQCH
	0.783	0.096	NXPH4
	0.757	0.071	TTC18
	0.754	0.068	ARL2BP
	0.742	0.056	SLC25A21
	0.755	0.069	OLFM1
	0.727	0.042	CRELD2
	0.741	0.056	ERGIC3
	0.743	0.058	PROSC
	0.744	0.059	PLK4
	0.736	0.052	LRRC8B
	0.743	0.059	OTOF
	0.755	0.071	IFNAR1
	0.762	0.079	PCDHB6
	0.738	0.054	AMELX
	0.773	0.089	ADAMTS8
	0.746	0.063	UGGT1
	0.741	0.058	CSN2
	0.749	0.066	TNFSF11
	0.752	0.069	FRAS1
	0.770	0.087	PPBPL2
	0.744	0.061	ZNF41
	0.746	0.064	PCDHGA10
	0.752	0.070	OMP
	0.761	0.079	DNAH6
	0.766	0.085	CHST4
	0.768	0.086	LRRC68
	0.748	0.067	C2ORF55
	0.734	0.053	NOL10
	0.748	0.067	ATXN2L
	0.755	0.073	GNPTAB
	0.734	0.054	DLC1
	0.720	0.040	ATG2B

	0.753	0.073	TTC12
	0.742	0.062	CFHR4
	0.746	0.067	TSGA10
	0.736	0.057	CETP
	0.742	0.064	KLHL29
	0.741	0.063	CCDC144A
	0.758	0.080	PLA2G2F
	0.737	0.059	FAM205B
	0.721	0.043	SIDT1
	0.736	0.058	LIM2
	0.741	0.063	RFPL1
	0.736	0.058	RAB9BP1
	0.736	0.058	FOXH1
	0.731	0.053	ZNF225
	0.738	0.060	SCIN
	0.732	0.055	PHF14
	0.756	0.079	JPH3
	0.753	0.076	PART1
	0.732	0.055	DPF1
	0.741	0.065	HIST1H2BL
	0.743	0.067	TCP10
	0.741	0.064	SLC25A42
	0.722	0.046	FRAT1
	0.742	0.065	TRAF3IP2
	0.749	0.073	GRIA4
	0.735	0.059	CACNA1C
	0.726	0.050	ZNF273
	0.730	0.054	GTPBP10
	0.743	0.068	PDE4B
	0.750	0.075	KCNE1
	0.737	0.063	SRSF7
	0.739	0.065	TELO2
	0.746	0.072	RAPSN
	0.744	0.070	GAN
	0.739	0.065	LAT2
	0.745	0.072	CRB1
	0.741	0.068	PEX5
	0.725	0.053	CEP97
	0.732	0.061	POU3F3
	0.752	0.081	C2ORF83
	0.751	0.081	CRYBB1
	0.746	0.076	SLC6A5
	0.723	0.053	PRR14L
	0.741	0.072	MLLT1
	0.735	0.065	POLRMT
	0.719	0.049	KRT76
	0.734	0.065	SLC7A4

	0.732	0.063	PCDHB1
	0.727	0.059	TMEM156
	0.750	0.082	GREB1L
	0.726	0.058	IFIH1
	0.739	0.072	HTR1B
	0.732	0.064	ACOXL
	0.746	0.078	FLJ20712
	0.732	0.065	OR7E24
	0.726	0.059	EFNA2
	0.719	0.052	GHSR
	0.721	0.054	MPPED2
	0.732	0.066	STAG3L4
	0.725	0.059	SENP6
	0.744	0.079	AGA
	0.743	0.077	CEACAM7
	0.733	0.067	FAM135A
	0.714	0.049	DUSP13
	0.731	0.065	PACRG
	0.719	0.054	ARHGEF10
	0.747	0.082	ADM2
	0.731	0.065	SPACA1
	0.720	0.054	GPR77
	0.721	0.056	HIST1H4D
	0.724	0.060	MYH7B
	0.725	0.061	LINC00115
	0.741	0.077	TBX4
	0.728	0.064	BPESC1
	0.723	0.060	SPO11
	0.746	0.082	TGFB2
	0.713	0.050	TIPARP
	0.717	0.054	ARHGEF38
	0.735	0.073	ZNF208
	0.727	0.065	GP6
	0.722	0.061	EPHX3
	0.729	0.068	LRIT1
	0.730	0.069	NEUROD4
	0.734	0.073	NRXN3
	0.730	0.070	ADAM20
	0.720	0.060	IFNB1
	0.734	0.074	RFX3
	0.726	0.066	HSPG2
	0.751	0.092	EXOSC2
	0.736	0.077	POM121
	0.720	0.061	PCDHGA9
	0.732	0.073	OR2B6
	0.704	0.045	HIC1
	0.729	0.071	SRY

	0.721	0.062	TULP1
	0.716	0.057	PLEK
	0.746	0.087	OBSCN
	0.723	0.064	OR7A10
	0.712	0.054	FAM46C
	0.732	0.074	ANKRD34C
	0.739	0.082	BARX1
	0.721	0.064	RBM3
	0.724	0.068	ZNF214
	0.727	0.071	NDST3
	0.725	0.069	TROVE2
	0.732	0.076	ZNF701
	0.727	0.071	PAX9
	0.734	0.078	HIST1H1A
	0.738	0.083	CNOT2
	0.712	0.057	TTLL3
	0.717	0.062	ZNF80
	0.717	0.062	HOXA2
	0.724	0.069	CNTN5
	0.736	0.081	CYSLTR2
	0.717	0.063	NR2C2
	0.710	0.056	SGCA
	0.730	0.076	ZNF132
	0.726	0.072	IL12RB1
	0.711	0.058	CSDA
	0.717	0.063	AGBL2
	0.729	0.076	FBXO4
	0.725	0.072	FGF23
	0.732	0.080	C6ORF123
	0.736	0.084	LY6G6E
	0.726	0.073	TLL1
	0.741	0.088	IFNA4
	0.729	0.077	SH2D3C
	0.712	0.059	BBS7
	0.723	0.071	SIK2
	0.724	0.072	TDRKH
	0.726	0.074	USH2A
	0.725	0.074	RPA4
	0.738	0.088	INE1
	0.720	0.069	LALBA
	0.729	0.079	EIF3F
	0.725	0.075	OR7E87P
	0.720	0.070	SCNN1A
	0.729	0.079	KCNC1
	0.728	0.079	ST8SIA3
	0.724	0.075	OBP2A
	0.724	0.075	GRK1

	0.734	0.086	GPR85
	0.714	0.066	PCDH11X
	0.721	0.073	PHLDB1
	0.724	0.076	HBEGF
	0.734	0.086	CHAT
	0.695	0.048	TRAT1
	0.717	0.070	ACCN4
	0.731	0.085	COL4A6
	0.716	0.070	PGLYRP1
	0.731	0.085	SCN8A
	0.733	0.087	ABCD2
	0.717	0.072	PRX
	0.703	0.057	SLC22A18AS
	0.721	0.076	HAUS5
	0.723	0.078	CRYGA
	0.738	0.093	GBX1
	0.706	0.061	PIWIL1
	0.734	0.089	AGRP
	0.714	0.070	SOX1
	0.712	0.068	HLA-DRB4
	0.712	0.068	MSH4
	0.732	0.089	TGM4
	0.706	0.063	HOXB8
	0.711	0.068	ANKRD7
	0.714	0.071	CEACAM1
	0.706	0.063	TPTE
	0.721	0.079	SLC14A2
	0.699	0.058	C6ORF103
	0.729	0.088	STXBP5L
	0.731	0.091	LINC00474
	0.724	0.084	RALGPS1
	0.718	0.078	TMC7
	0.713	0.073	ATOH1
	0.719	0.079	ASIP
	0.710	0.070	TP73
	0.717	0.078	CADPS
	0.723	0.084	NTNG1
	0.720	0.081	FOXN2
	0.699	0.060	ELK3
	0.717	0.079	GJA5
	0.714	0.076	NACA2
	0.717	0.079	ZNF674
	0.707	0.069	FOXB1
	0.695	0.057	ASCL2
	0.692	0.055	EIF3M
	0.720	0.083	GUCY2C
	0.705	0.069	CCDC134

	0.718	0.082	RANBP1
	0.719	0.083	AGRN
	0.736	0.101	PCDHB8
	0.721	0.086	AMELY
	0.721	0.086	PARK2
	0.722	0.087	PLEKHH3
	0.721	0.086	IQCA1
	0.722	0.088	CDH15
	0.715	0.082	KIAA1967
	0.707	0.073	GRIN2D
	0.720	0.086	HNMT
	0.710	0.076	CRTC1
	0.693	0.061	C9ORF68
	0.721	0.089	NLGN4Y
	0.696	0.064	WDR96
	0.697	0.066	AMPD3
	0.707	0.075	RPS28
	0.708	0.076	RAB40A
	0.697	0.065	IGFBP7
	0.718	0.087	OR11A1
	0.705	0.074	GUCY2D
	0.710	0.079	KDM4C
	0.692	0.062	LPHN3
	0.697	0.068	CXCL3
	0.700	0.070	MARCH6
	0.714	0.084	DAB1
	0.703	0.074	HIST1H4F
	0.712	0.084	PBOV1
	0.720	0.093	BRDT
	0.724	0.097	PRG3
	0.707	0.080	GRID2
	0.680	0.054	DCAF13
	0.710	0.083	ERCC4
	0.707	0.081	LOC100505960
	0.710	0.085	CSF2
	0.711	0.086	PEX16
	0.709	0.083	FBN1
	0.705	0.080	GLI1
	0.694	0.069	MGAT4C
	0.706	0.082	C14ORF56
	0.699	0.075	EDDM3A
	0.681	0.056	MXD1
	0.725	0.101	C19ORF50
	0.695	0.071	LCMT2
	0.704	0.080	ATP8A2
	0.726	0.102	PREX2
	0.695	0.071	GTF2A1

0.707	0.084	TRMT1
0.715	0.092	LRRC41
0.710	0.087	NPHP1
0.707	0.085	SLC8A1
0.723	0.101	PNMA2
0.720	0.099	IFNW1
0.712	0.091	KLF15
0.701	0.080	C16ORF71
0.706	0.086	PTCH2
0.718	0.098	MBL1P
0.700	0.080	CDH9
0.697	0.077	ZBTB7B
0.698	0.079	GLS
0.695	0.076	FSTL4
0.693	0.074	PHEX
0.702	0.083	RGS11
0.677	0.058	ARHGAP33
0.685	0.067	LNPEP
0.682	0.063	ZBTB7C
0.717	0.098	FAM125B
0.711	0.093	SH2D2A
0.709	0.091	MTSS1
0.696	0.078	IGKV1-5
0.694	0.077	OBFC2A
0.700	0.083	AREG
0.693	0.077	ZBTB32
0.691	0.074	WDHD1
0.672	0.056	GNRH1
0.688	0.072	KLHL28
0.710	0.094	NUP205
0.698	0.082	OR2F2
0.704	0.089	ZNF467
0.695	0.080	NYX
0.692	0.077	CHD4
0.704	0.090	NUP88
0.706	0.091	ZNF259P1
0.691	0.076	GIP
0.680	0.066	IGLV6-57
0.686	0.072	MGC13053
0.694	0.081	LOC100130354
0.687	0.074	ASB4
0.692	0.079	FOXD2
0.698	0.086	ADAM30
0.711	0.099	TBX10
0.695	0.084	DRD5
0.699	0.088	SLC25A30
0.693	0.082	THAP9

	0.685	0.075	CKAP4
	0.699	0.089	CSDC2
	0.691	0.082	TTC38
	0.656	0.047	TXNL1
	0.656	0.047	NID2
	0.688	0.079	CLUL1
	0.691	0.083	CSPG4
	0.694	0.085	MYH13
	0.680	0.072	MYB
	0.681	0.074	SLC25A16
	0.704	0.097	MYO15A
	0.691	0.084	TRPM2
	0.684	0.077	NR2E1
	0.687	0.080	IFNA10
	0.642	0.036	LCN2
	0.664	0.058	TPR
	0.715	0.110	SOX30
	0.699	0.094	SSTR4
	0.695	0.091	CDADC1
	0.701	0.097	GUCY1B2
	0.699	0.095	ADAM7
	0.686	0.082	LOC100127886
	0.679	0.075	EIF5B
	0.690	0.087	HNRNPA3P1
	0.681	0.078	CATR1
	0.684	0.081	MGC2889
	0.694	0.091	PRSS53
	0.694	0.092	NOD1
	0.684	0.082	PDE6C
	0.685	0.083	RAVER2
	0.683	0.081	TRBC2
	0.698	0.096	BAIAP2
	0.690	0.088	KIR3DX1
	0.686	0.085	CHD2
	0.688	0.088	ZBTB33
	0.678	0.079	LINC00230A
	0.692	0.093	KRTAP5-8
	0.701	0.101	KCNA1
	0.671	0.071	RPS2P45
	0.680	0.081	ZFY
	0.694	0.094	GFM1
	0.707	0.108	NMBR
	0.700	0.101	FGF8
	0.692	0.094	IL4
	0.690	0.093	RAB40AL
	0.668	0.071	RARS2
	0.697	0.100	IFNA14

	0.688	0.092	TAF13
	0.679	0.083	PCDHB17
	0.688	0.092	ZNF749
	0.680	0.085	ADARB2
	0.645	0.050	CCND2
	0.689	0.095	ADAM3A
	0.691	0.096	PCCA
	0.681	0.086	RFX1
	0.685	0.091	ADCY9
	0.675	0.081	PYHIN1
	0.684	0.090	MORC1
	0.685	0.092	TRPC7
	0.666	0.072	FER
	0.669	0.075	TMF1
	0.691	0.099	ULBP1
	0.692	0.100	SEC14L5
	0.676	0.084	LRRC3
	0.679	0.087	GPR4
	0.689	0.097	GMIP
	0.673	0.082	ACSBG2
	0.684	0.094	TIMM8A
	0.655	0.065	DNMT3A
	0.669	0.078	PRDM5
	0.693	0.103	CETN1
	0.672	0.082	SETD2
	0.686	0.096	PRAMEF11
	0.671	0.081	PRPH2
	0.697	0.107	RAB40C
	0.675	0.085	IDI2-AS1
	0.668	0.078	LRP5L
	0.668	0.079	DUOX1
	0.680	0.091	CCDC82
	0.668	0.078	SYCE1L
	0.690	0.100	ZC3H13
	0.679	0.090	CHD5
	0.678	0.089	CNOT3
	0.666	0.077	NPAS3
	0.679	0.091	CPLX2
	0.691	0.103	PNLIPRP1
	0.670	0.083	NPY6R
	0.672	0.085	ATF7IP
	0.691	0.104	IL3
	0.647	0.060	DYM
	0.670	0.083	BCL3
	0.674	0.087	TPSD1
	0.679	0.092	IFNA6
	0.679	0.093	EDA2R

	0.669	0.083	RSG1
	0.686	0.100	TEC
	0.653	0.067	GABRA6
	0.689	0.104	ACSBG1
	0.674	0.089	RLBP1
	0.664	0.079	SUV39H2
	0.667	0.082	OR1D2
	0.675	0.090	ANKRD36BP2
	0.674	0.089	CCNT2
	0.671	0.087	PKD2L2
	0.684	0.099	DLG2
	0.679	0.095	PTGDR
	0.676	0.092	ABCD1
	0.656	0.073	QSER1
	0.679	0.096	TCF20
	0.671	0.088	ZNF355P
	0.654	0.071	ZNF471
	0.669	0.086	WDR59
	0.686	0.104	MTUS2
	0.676	0.095	MS4A5
	0.670	0.090	IFNA8
	0.676	0.095	CLCA3P
	0.678	0.098	FBXO24
	0.678	0.099	TMEM144
	0.665	0.086	PHC3
	0.666	0.088	GARNL3
	0.680	0.102	C16ORF3
	0.688	0.110	IL9
	0.671	0.094	TTTY2
	0.674	0.097	ACR
	0.682	0.105	OR2C1
	0.677	0.101	OR1F2P
	0.653	0.077	NDST1
	0.675	0.100	CDS1
	0.651	0.076	ALPI
	0.677	0.103	ZNF835
	0.681	0.107	SMAD4
	0.672	0.099	KRT35
	0.657	0.084	LOC100129973
	0.677	0.104	F2RL3
	0.653	0.080	ZNF480
	0.671	0.099	SOX14
	0.664	0.094	ATP2B3
	0.672	0.102	PRR5
	0.676	0.106	KCNJ9
	0.679	0.110	CCZ1
	0.674	0.105	PRND

	0.670	0.102	GAP43
	0.665	0.097	EHMT1
	0.661	0.094	ATP10B
	0.667	0.099	EPHB1
	0.640	0.073	THOC5
	0.655	0.088	ANAPC13
	0.655	0.089	FAM182A
	0.656	0.090	SERPINB7
	0.654	0.089	TEX12
	0.672	0.107	GCNT2
	0.659	0.094	PDPN
	0.673	0.109	GAL
	0.652	0.087	C6ORF25
	0.650	0.086	C8ORF71
	0.659	0.095	BFSP2
	0.637	0.074	ANK1
	0.650	0.086	CSRNP3
	0.664	0.101	SYT12
	0.665	0.102	SEC14L3
	0.652	0.089	GALR2
	0.646	0.084	SOX21
	0.660	0.098	CDY1
	0.661	0.101	HOXD1
	0.673	0.112	KRTAP4-7
	0.659	0.099	XRCC6
	0.657	0.098	SPICE1
	0.642	0.084	TADA2A
	0.645	0.086	FARS2
	0.659	0.102	KRTAP1-3
	0.662	0.104	B3GALT5
	0.653	0.095	RFPL1-AS1
	0.665	0.108	TAS2R16
	0.671	0.114	ZBED4
	0.653	0.097	DOPEY1
	0.647	0.090	SIGLEC8
	0.655	0.099	SRRM2
	0.637	0.082	CSNK2A1
	0.648	0.093	HIPK2
	0.648	0.094	ZPBP
	0.649	0.094	IFNA2
	0.658	0.103	KANK2
	0.629	0.075	CYP19A1
	0.638	0.084	TREH
	0.659	0.105	CHRNA2
	0.655	0.101	CDON
	0.655	0.102	CTAGE1
	0.647	0.094	OLAH

	0.661	0.109	ZSCAN12
	0.664	0.112	SHOX2
	0.651	0.099	GLRA1
	0.645	0.093	PLIN2
	0.653	0.102	CAMK4
	0.660	0.109	EPHA1
	0.667	0.115	FNTB
	0.661	0.110	NSUN7
	0.637	0.086	RAB30
	0.651	0.100	MLN
	0.646	0.095	VCPIP1
	0.653	0.103	ZXDB
	0.660	0.110	HYDIN
	0.649	0.099	RNF24
	0.663	0.113	OR1E1
	0.654	0.104	CYP2B6
	0.629	0.080	KIAA0196
	0.662	0.114	GPR172B
	0.653	0.105	POU2F3
	0.651	0.103	RASSF4
	0.645	0.098	TCEB3B
	0.646	0.099	C16ORF70
	0.641	0.094	SCRT1
	0.655	0.108	MTNR1A
	0.654	0.108	PPP1R2P9
	0.650	0.105	FBXO41
	0.635	0.089	HEATR8
	0.635	0.089	PLA2R1
	0.631	0.086	CDKL2
	0.651	0.106	MINK1
	0.657	0.112	LOC100093698
	0.637	0.093	CHRNB4
	0.646	0.102	IMPG1
	0.644	0.100	S1PR2
	0.646	0.102	ERC1
	0.659	0.115	OR2W1
	0.649	0.105	POU3F4
	0.648	0.104	SERPINB9
	0.649	0.105	GSC2
	0.666	0.123	IL11
	0.634	0.092	KRT31
	0.643	0.100	PRKAB1
	0.656	0.114	LOC100131825
	0.631	0.090	SLC4A10
	0.629	0.088	PDK3
	0.646	0.105	SLC2A4
	0.650	0.109	FOXE3

	0.636	0.095	POU2F2
	0.641	0.100	CTDSPL
	0.639	0.098	PCLO
	0.632	0.092	PTGIS
	0.625	0.085	TMX4
	0.644	0.104	FAM13C
	0.648	0.108	FAM49B
	0.649	0.109	VAV2
	0.662	0.123	P2RY4
	0.639	0.100	CRYBA1
	0.638	0.099	KIAA0087
	0.641	0.102	KCNH1
	0.652	0.113	LPAR4
	0.642	0.104	AKT3
	0.645	0.106	GJB4
	0.626	0.088	MAP3K9
	0.641	0.103	PRM2
	0.638	0.100	PIGC
	0.631	0.093	IKZF2
	0.646	0.109	LGALS13
	0.650	0.114	LOC100128640
	0.641	0.105	STAMBPL1
	0.653	0.118	IFNA7
	0.641	0.106	HSDL2
	0.640	0.105	NOX3
	0.654	0.121	MTMR10
	0.642	0.109	CCDC40
	0.647	0.114	NR4A1
	0.631	0.098	WWTR1
	0.619	0.086	OTUD4
	0.626	0.093	C22ORF31
	0.640	0.108	CMA1
	0.634	0.101	ILF3
	0.636	0.104	PAPOLB
	0.617	0.085	CCDC70
	0.631	0.099	FN3K
	0.632	0.101	MCF2L
	0.615	0.084	GFRA3
	0.635	0.104	PBX2
	0.635	0.104	TGM5
	0.651	0.120	SEPT4
	0.630	0.100	GREB1
	0.659	0.129	INTS1
	0.619	0.089	HMGA2
	0.614	0.084	SLC1A3
	0.626	0.096	CCNK
	0.633	0.104	ELK1

	0.642	0.113	NCR1
	0.632	0.103	KCNJ3
	0.642	0.113	PCDHGB6
	0.632	0.103	CRYGB
	0.634	0.106	MTL5
	0.650	0.122	HOXD9
	0.645	0.118	LMAN2
	0.633	0.106	SLC39A2
	0.639	0.112	COLEC10
	0.621	0.096	OR10H2
	0.597	0.072	TMOD2
	0.643	0.118	OR1A2
	0.632	0.108	ZNF550
	0.626	0.102	IFNA5
	0.638	0.113	RAB11FIP3
	0.626	0.103	C11ORF1
	0.632	0.109	ARMC9
	0.643	0.120	TFPI
	0.622	0.099	VPS13B
	0.633	0.111	OR2J3
	0.630	0.110	EXOG
	0.637	0.117	CALCR
	0.629	0.109	LIPE
	0.634	0.115	CCT8L2
	0.636	0.117	IFNA17
	0.629	0.110	MTNR1B
	0.627	0.108	TRPC5
	0.623	0.105	RTDR1
	0.634	0.115	HRH3
	0.585	0.067	RAB21
	0.618	0.099	OR10C1
	0.620	0.102	DPY19L1
	0.625	0.108	DPYSL4
	0.619	0.102	FAM75C2
	0.630	0.114	WNT2B
	0.620	0.104	CNR2
	0.619	0.103	NCBP1
	0.618	0.102	FGF6
	0.613	0.097	UPF1
	0.620	0.104	POU3F1
	0.632	0.117	ST3GAL4
	0.635	0.120	CRYBA4
	0.631	0.117	ANKS1B
	0.614	0.100	CABYR
	0.636	0.121	IL1R1
	0.623	0.108	SPRY4
	0.617	0.102	OTUD7B

	0.620	0.106	C10ORF137
	0.623	0.110	PRPS1L1
	0.618	0.105	LOC100507284
	0.621	0.108	YES1
	0.614	0.102	EXPH5
	0.632	0.120	EZR
	0.627	0.115	CBX2
	0.619	0.108	GPR31
	0.625	0.114	C8ORF39
	0.613	0.103	TFAP2B
	0.614	0.104	OR2J2
	0.634	0.124	RGS1
	0.624	0.115	GOLGA6A
	0.621	0.113	IL26
	0.620	0.112	LOC100131298
	0.611	0.103	EPS15L1
	0.622	0.115	GART
	0.628	0.121	GSPT1
	0.614	0.106	KIDINS220
	0.630	0.123	PMFBP1
	0.626	0.119	LSS
	0.627	0.120	GSK3A
	0.613	0.107	SYT2
	0.612	0.107	NAG18
	0.607	0.102	RAX
	0.621	0.116	GJA9
	0.621	0.115	TAS2R14
	0.625	0.120	MAGEC3
	0.624	0.119	MYOF
	0.623	0.119	CD80
	0.619	0.115	OR2H1
	0.615	0.111	DKFZP686O1327
	0.625	0.120	KISS1
	0.605	0.101	AKIRIN2-AS1
	0.613	0.109	AURKA
	0.612	0.109	PARVB
	0.607	0.104	TNFRSF10D
	0.613	0.109	B4GALT6
	0.630	0.126	ILVBL
	0.611	0.108	PTPRN2
	0.629	0.126	C7ORF69
	0.614	0.113	SHH
	0.622	0.120	LILRP2
	0.624	0.122	ADAM18
	0.605	0.104	CNGA3
	0.617	0.116	DGCR2
	0.612	0.111	MAPK8IP1

	0.610	0.109	IGHV5-78
	0.621	0.120	COL19A1
	0.623	0.123	OR7A17
	0.618	0.118	DUS2L
	0.601	0.101	PHF20L1
	0.631	0.131	GPR50
	0.619	0.119	ORC5
	0.619	0.120	SIVA1
	0.583	0.084	CASP3
	0.602	0.103	SLC35F5
	0.617	0.120	MED6
	0.618	0.120	LRRC37BP1
	0.614	0.117	PQLC3
	0.612	0.115	DDX4
	0.609	0.113	PCDHA3
	0.628	0.132	SPAG8
	0.623	0.127	KIAA0509
	0.619	0.124	WSCD2
	0.616	0.121	ABCC6
	0.613	0.118	ICA1
	0.607	0.113	PPEF2
	0.610	0.117	BTN2A3P
	0.600	0.107	RIMS2
	0.592	0.098	COL1A2
	0.608	0.115	AANAT
	0.598	0.105	MPP2
	0.610	0.118	LAMA2
	0.611	0.119	MOS
	0.622	0.130	NCR3
	0.613	0.122	KDSR
	0.607	0.115	CASR
	0.616	0.125	MAF
	0.619	0.128	IFT27
	0.603	0.113	MKRN2
	0.621	0.131	SLC12A5
	0.601	0.113	LRRC23
	0.609	0.121	CNTLN
	0.602	0.114	ADAM8
	0.604	0.116	ADCK4
	0.606	0.119	TNNI3K
	0.594	0.107	XCR1
	0.600	0.113	GRIK5
	0.615	0.128	SPN
	0.618	0.132	LOC100130331
	0.588	0.101	RCHY1
	0.602	0.116	ZNF549
	0.599	0.113	TOP3B

	0.609	0.124	CER1
	0.603	0.118	PLAG1
	0.596	0.111	PPIAL4A
	0.613	0.128	ADRA1B
	0.613	0.128	ACTR2
	0.595	0.110	CRISP1
	0.606	0.122	TEX13A
	0.617	0.134	OSM
	0.607	0.123	TAS2R13
	0.612	0.129	RASAL2
	0.619	0.136	MYO15B
	0.598	0.115	PDE3A
	0.613	0.130	DOPEY2
	0.609	0.127	ZNF221
	0.614	0.132	AMOT
	0.606	0.124	ARFRP1
	0.608	0.126	CG030
	0.607	0.125	POFUT1
	0.603	0.121	CHRNA6
	0.613	0.132	SCUBE3
	0.607	0.127	F12
	0.594	0.114	HTR2A
	0.609	0.130	SNTG2
	0.598	0.118	HBP1
	0.614	0.135	CHRM5
	0.620	0.141	OR7E19P
	0.586	0.108	MEOX2
	0.601	0.125	ETV3
	0.602	0.125	ZBTB44
	0.603	0.128	HCG4B
	0.581	0.105	CSN3
	0.596	0.121	TXNDC3
	0.580	0.104	YIF1B
	0.594	0.119	EPM2A
	0.601	0.126	PDPR
	0.593	0.118	GAST
	0.601	0.127	PEX5L
	0.598	0.124	PRDM9
	0.603	0.129	SHMT1
	0.593	0.120	PPYR1
	0.593	0.121	NEU3
	0.592	0.119	GP2
	0.584	0.113	SEMA3D
	0.602	0.130	ALX4
	0.587	0.116	LBX1
	0.588	0.117	AFF4
	0.596	0.126	MYO5A

	0.593	0.123	PSG4
	0.599	0.129	GPR161
	0.600	0.131	OPN1SW
	0.589	0.120	PDE12
	0.596	0.127	TAL1
	0.599	0.131	COL6A2
	0.603	0.135	ATXN8OS
	0.591	0.123	KRT38
	0.581	0.113	HCFC1
	0.596	0.129	REG1P
	0.583	0.115	DEC1
	0.590	0.123	VN1R1
	0.580	0.114	GPR97
	0.586	0.121	C15ORF39
	0.600	0.135	TAAR2
	0.596	0.131	ZNF695
	0.588	0.124	AGAP2
	0.589	0.124	ISG20L2
	0.541	0.077	CEBPA
	0.591	0.128	SLC24A2
	0.597	0.133	HTR7P1
	0.586	0.123	RUNX1T1
	0.590	0.127	OR10J1
	0.603	0.141	APC2
	0.598	0.136	ZER1
	0.588	0.127	ADAMTS6
	0.589	0.129	TRHR
	0.583	0.123	ZNF343
	0.585	0.125	HDAC11
	0.583	0.123	ANKRD26
	0.593	0.134	GABRA1
	0.573	0.114	FAM120A
	0.586	0.127	CEP152
	0.594	0.135	GLRA3
	0.578	0.120	SLC5A5
	0.599	0.140	PCSK2
	0.538	0.079	KLHL9
	0.589	0.131	KLRC4
	0.588	0.130	HERC4
	0.590	0.133	EHD2
	0.586	0.130	SCN3B
	0.592	0.136	SLC4A7
	0.585	0.129	KIF1A
	0.591	0.135	CLOCK
	0.581	0.126	PDCD6
	0.598	0.143	MICAL2
	0.586	0.131	KIAA1467

	0.579	0.125	P2RX6
	0.578	0.124	SERPINB2
	0.581	0.127	KAZN
	0.593	0.140	ATG10
	0.579	0.126	PNLIPRP2
	0.589	0.137	MSX2
	0.582	0.131	FSHR
	0.583	0.133	FGF18
	0.574	0.124	RBFOX2
	0.594	0.144	HOXD3
	0.588	0.138	TIAM2
	0.517	0.067	STXBP2
	0.574	0.125	PP13
	0.588	0.140	LAMB4
	0.565	0.116	FSHB
	0.564	0.116	ZCWPW1
	0.585	0.137	AHCYL2
	0.567	0.120	HABP4
	0.577	0.130	SDHD
	0.588	0.141	PRELP
	0.571	0.124	ASH1L
	0.582	0.134	BPY2
	0.580	0.133	DKFZP434C153
	0.575	0.128	C3ORF51
	0.575	0.128	UBR2
	0.570	0.123	CLTA
	0.576	0.131	CNTFR
	0.578	0.132	IL22
	0.566	0.121	KCNMA1
	0.564	0.119	GGA3
	0.572	0.128	MYH15
	0.581	0.138	SPTB
	0.572	0.128	PAX5
	0.567	0.125	ANP32C
	0.593	0.150	MC5R
	0.584	0.142	PLCB1
	0.568	0.126	FOXA2
	0.558	0.117	LRRC37A4
	0.561	0.120	UBA6
	0.574	0.133	GSG1
	0.568	0.127	ELL
	0.577	0.136	PSG3
	0.573	0.133	GPR75
	0.567	0.127	CHRM2
	0.562	0.122	SLC25A31
	0.586	0.147	TNFRSF9
	0.565	0.126	SCN7A

	0.552	0.113	MGAT5
	0.552	0.113	MBNL1
	0.574	0.135	EFHC2
	0.574	0.135	HTR4
	0.583	0.145	ZMPSTE24
	0.576	0.137	RRH
	0.542	0.106	CREB1
	0.585	0.148	RNF5
	0.563	0.127	ZNF688
	0.579	0.143	ADARB1
	0.577	0.141	C4ORF6
	0.575	0.139	PPP2R2B
	0.560	0.126	PDAP1
	0.572	0.137	SLC5A2
	0.561	0.126	UBQLN3
	0.562	0.128	TAF7L
	0.558	0.125	POU1F1
	0.571	0.137	CYP2C8
	0.578	0.145	NIPAL3
	0.570	0.137	SLC28A2
	0.578	0.145	POLG
	0.585	0.152	RBMS2
	0.570	0.137	AURKC
	0.583	0.151	TSKS
	0.573	0.140	ELAC1
	0.564	0.132	ALK
	0.561	0.129	DENND1C
	0.578	0.146	EYA3
	0.552	0.121	RXFP3
	0.582	0.152	OBSL1
	0.555	0.125	HNRPDL
	0.568	0.138	KCNC3
	0.562	0.132	NLRP3
	0.570	0.141	KRIT1
	0.522	0.093	MKNK1
	0.574	0.145	LMF1
	0.565	0.137	TAS2R7
	0.551	0.124	IGF2R
	0.567	0.141	CTAGE11P
	0.573	0.147	PI4K2A
	0.568	0.142	PLSCR2
	0.571	0.145	ZNF277
	0.555	0.129	SLC2A1
	0.559	0.134	ARHGAP24
	0.576	0.151	NEBL
	0.541	0.116	FLJ10038
	0.562	0.139	GPR68

	0.569	0.146	SLCO1A2
	0.551	0.128	C15ORF34
	0.545	0.122	ZNF780B
	0.555	0.133	TAF4
	0.551	0.128	GJC2
	0.571	0.149	KIAA1654
	0.561	0.139	PRDM2
	0.543	0.121	PCDH7
	0.564	0.143	ARID1A
	0.551	0.131	EBF2
	0.567	0.146	TIMM17A
	0.572	0.152	GCNT4
	0.557	0.137	CHIA
	0.556	0.137	CEACAM4
	0.567	0.148	FRMD8
	0.551	0.132	CD5
	0.557	0.139	ADAMTS7
	0.545	0.127	LDHAL6B
	0.549	0.132	ULK2
	0.550	0.134	ADCY1
	0.562	0.146	ZCCHC11
	0.545	0.130	POM121L9P
	0.544	0.129	UBAP2L
	0.550	0.135	OR1G1
	0.559	0.144	RGS14
	0.549	0.135	MMP11
	0.560	0.147	CNGB1
	0.550	0.137	RAPGEF3
	0.554	0.141	MYL4
	0.566	0.154	CA1
	0.541	0.129	LOC730227
	0.543	0.131	CPT1A
	0.546	0.135	GRM1
	0.538	0.127	REPS1
	0.561	0.150	PBXIP1
	0.549	0.138	TCF25
	0.546	0.136	AKAP9
	0.558	0.148	RQCD1
	0.551	0.142	DES
	0.547	0.137	TSHR
	0.538	0.129	C7ORF58
	0.553	0.143	NPBWR2
	0.541	0.132	PTEN
	0.543	0.137	SEMA7A
	0.535	0.128	SLC16A3
	0.554	0.148	HIC2
	0.567	0.161	FEZ1

	0.559	0.153	SYN2
	0.559	0.154	KRTAP2-4
	0.544	0.139	IFNA21
	0.527	0.122	KLHL11
	0.546	0.141	GNAT1
	0.525	0.120	TRIM2
	0.544	0.139	CELF3
	0.544	0.140	CTNS
	0.546	0.141	KCNA2
	0.537	0.133	HOXC11
	0.548	0.144	GGA1
	0.541	0.137	CHRND
	0.543	0.140	PPP1R3A
	0.539	0.136	HNRNPU
	0.541	0.137	AFF1
	0.549	0.147	HNRNPD
	0.549	0.148	CC2D1A
	0.539	0.138	RDH8
	0.551	0.150	ABCB11
	0.539	0.139	PTENP1
	0.531	0.131	ZC3H15
	0.546	0.146	ACRV1
	0.550	0.150	B3GALT1
	0.528	0.129	TLX2
	0.548	0.149	KCNE2
	0.539	0.141	RAPGEF2
	0.553	0.155	TRPC6
	0.513	0.115	TFAP2A
	0.513	0.115	UBE2L3
	0.508	0.110	HOMER2
	0.548	0.151	GPX5
	0.548	0.151	MED18
	0.535	0.138	PMAIP1
	0.553	0.156	ASMT
	0.533	0.136	PARD6B
	0.533	0.136	OR7C1
	0.549	0.152	A4GNT
	0.547	0.151	C1QL1
	0.544	0.148	PVT1
	0.516	0.120	COL3A1
	0.546	0.151	VPS13A
	0.542	0.147	RNASE1
	0.538	0.143	TAF6L
	0.541	0.146	GABRB2
	0.540	0.145	HSD3B1
	0.526	0.132	CCDC28B
	0.533	0.139	KCNV2

	0.530	0.136	SPINLW1
	0.544	0.152	KCNJ6
	0.535	0.143	SLC9A3
	0.532	0.141	KCNH6
	0.525	0.134	TCP10L
	0.540	0.149	BMP10
	0.535	0.144	CXORF57
	0.543	0.153	BSN
	0.540	0.151	TRADD
	0.547	0.159	LTBP4
	0.545	0.157	ST3GAL6
	0.544	0.156	ELAC2
	0.540	0.153	ST14
	0.533	0.146	GPR137
	0.534	0.147	BAZ2B
	0.520	0.133	ZNF192
	0.542	0.157	C13ORF44
	0.540	0.154	DMC1
	0.538	0.152	DMPK
	0.533	0.148	MLNR
	0.523	0.138	LOC100129648
	0.534	0.150	FUT3
	0.545	0.161	TMEM206
	0.528	0.144	FKBP1A
	0.520	0.136	BTG4
	0.536	0.152	LPAL2
	0.536	0.152	ELOVL5
	0.544	0.161	TRPM1
	0.530	0.148	NEB
	0.529	0.146	SPPL2B
	0.535	0.153	TNFRSF4
	0.532	0.150	MMP27
	0.526	0.145	FOXE1
	0.543	0.163	KIAA0748
	0.526	0.146	LUZP4
	0.541	0.161	C15ORF2
	0.531	0.152	GRIP1
	0.523	0.144	ICOSLG
	0.544	0.165	NOVA2
	0.524	0.145	DNM2
	0.542	0.164	DLX4
	0.535	0.158	MYO7B
	0.529	0.151	INVS
	0.525	0.148	TSHB
	0.515	0.138	TAAR5
	0.531	0.157	SCN11A
	0.525	0.151	GABRA2

	0.535	0.160	UST
	0.529	0.155	BCL2L11
	0.528	0.154	PCDHGA1
	0.517	0.143	PDE11A
	0.529	0.156	PIAS2
	0.518	0.146	GML
	0.521	0.149	PAK7
	0.527	0.154	C4ORF10
	0.529	0.157	NR5A2
	0.506	0.135	SAFB
	0.528	0.158	PSG2
	0.522	0.152	MUC8
	0.522	0.153	DTX3
	0.519	0.151	WAS
	0.520	0.151	TP63
	0.527	0.158	RYR2
	0.502	0.133	ABCF1
	0.522	0.154	TTLL4
	0.516	0.147	C14ORF162
	0.523	0.154	KLK10
	0.520	0.152	C17ORF88
	0.514	0.146	EHD1
	0.518	0.151	DISC1
	0.520	0.152	MIP
	0.518	0.151	PER2
	0.525	0.159	DNAJC16
	0.523	0.157	ZBTB7A
	0.529	0.163	FKBP8
	0.503	0.138	RRP8
	0.510	0.145	KIR2DS1
	0.522	0.157	ZNF335
	0.508	0.144	GNAT3
	0.509	0.145	ADAMTSL4
	0.505	0.140	LENEP
	0.532	0.169	KIAA1045
	0.510	0.146	LAG3
	0.511	0.148	USP29
	0.514	0.152	TACSTD2
	0.521	0.159	ZNF440
	0.509	0.148	MCFD2
	0.520	0.159	RAP2A
	0.516	0.155	AKAP1
	0.516	0.156	OGG1
	0.510	0.150	SH3GL3
	0.516	0.156	PCDH11Y
	0.513	0.154	CHRD
	0.515	0.156	TEX15

	0.514	0.155	KIAA0195
	0.518	0.161	KIAA1609
	0.510	0.153	RBMY2FP
	0.526	0.170	SLC22A13
	0.515	0.159	FP588
	0.517	0.161	PAX4
	0.513	0.158	SVEP1
	0.512	0.157	NR2E3
	0.512	0.157	PYGO1
	0.507	0.152	GGTLC2
	0.503	0.148	KHK
	0.502	0.148	SLC22A14
	0.525	0.171	TBC1D2B
	0.508	0.155	HNF1B
	0.509	0.156	NAA11
	0.518	0.167	AHI1
	0.505	0.153	IQCK
	0.508	0.157	DIMT1
	0.513	0.162	CNKSR2
	0.510	0.160	NOTCH3
	0.511	0.161	LDB3
	0.512	0.162	LOC100508797
	0.511	0.162	APOL5
	0.504	0.157	KIAA1456
	0.501	0.154	HR44
	0.506	0.161	KIF2A
	0.521	0.176	SLC22A17
	0.506	0.162	ZNF154
	0.511	0.167	BRF1
	0.503	0.159	RNF185
	0.508	0.165	RASGRP2
	0.505	0.164	ZNF280A
	0.505	0.164	LOC647070
	0.501	0.161	EFNA5
	0.507	0.167	ANK2
	0.501	0.161	SLIT3
	0.504	0.165	ARL4C
	0.501	0.163	PRKAR2A
	0.505	0.169	PCDHA2
	0.502	0.167	TAS2R4
	0.508	0.174	HPCAL4
	0.504	0.173	FLT1
	0.501	0.171	CCHCR1
	0.504	0.177	PCYT1B
	0.501	0.178	CSNK1G1

Combination prediction result

Name	Overlap count	Up	Down	Result
INS	4	2	2	
ABHD10	3	1	2	
AFF4	3	2	1	
AURKA	3	2	1	
CAMKK2	3	2	1	
CAT	3	2	1	
CETP	3	1	2	
DCAF17	3	1	2	
DIAPH2	3	1	2	
DPF1	3	1	2	
FBXO42	3	2	1	
FKBP5	3	2	1	
GPR19	3	1	2	
ID1	3	3	0	
IGF2R	3	3	0	
KRT3	3	1	2	
MAP3K9	3	2	1	
MCMBP	3	1	2	
MDM2	3	1	2	
MXD1	3	2	1	
PAN2	3	2	1	
POLG2	3	1	2	
RARG	3	3	0	
REN	3	2	1	
SCD5	3	2	1	
SLC6A4	3	1	2	
SP2	3	2	1	
SUV39H2	3	1	2	
TNNT1	3	2	1	
TPSB2	3	1	2	
TRIM13	3	3	0	
UBR7	3	0	3	
ZBTB43	3	1	2	
AASDHPPT	2	0	2	
ABP1	2	2	0	
ADH5	2	0	2	
AKAP9	2	2	0	
AMACR	2	2	0	
ANXA1	2	0	2	
ARFGEF2	2	2	0	
ARID1A	2	2	0	
ATF6	2	2	0	
ATMIN	2	0	2	
ATP9B	2	2	0	
BAIAP2	2	2	0	

BAZ2A	2	2	0	
BAZ2B	2	2	0	
BIN1	2	2	0	
C1ORF183	2	0	2	
CAB39	2	0	2	
CASP8	2	0	2	
CBR3	2	2	0	
CBX7	2	0	2	
CC2D1A	2	2	0	
CCND2	2	2	0	
CCNT1	2	2	0	
CCNT2	2	2	0	
CDK5R1	2	2	0	
CELP	2	2	0	
CEP152	2	2	0	
CHIC2	2	2	0	
CHRNE	2	2	0	
CLCN2	2	0	2	
CLDN15	2	2	0	
CLDN9	2	2	0	
CLIC3	2	0	2	
CREB1	2	2	0	
CREB3L1	2	2	0	
CRKL	2	2	0	
CSNK2A1	2	2	0	
CYP19A1	2	2	0	
DDR1	2	2	0	
DLX4	2	2	0	
DMPK	2	2	0	
DPYSL4	2	2	0	
DYSF	2	2	0	
EBAG9	2	0	2	
ECI2	2	0	2	
EDN1	2	2	0	
EDNRA	2	2	0	
EFNA3	2	0	2	
EHD1	2	2	0	
EHHADH	2	0	2	
ELAVL1	2	0	2	
ELK1	2	2	0	
ELK4	2	2	0	
EPHA4	2	2	0	
EPRS	2	0	2	
ERC1	2	2	0	
ETAA1	2	0	2	
EVL	2	0	2	
EWSR1	2	2	0	
FAM172A	2	0	2	

FDXR	2	2	0	
FHL2	2	2	0	
FOXC1	2	2	0	
FRMD4B	2	2	0	
GAL	2	2	0	
GATA1	2	2	0	
GGA3	2	2	0	
GMEB1	2	2	0	
GPX1	2	2	0	
GRB10	2	0	2	
H6PD	2	2	0	
HEG1	2	2	0	
HIC2	2	2	0	
HIST1H1D	2	2	0	
HIST1H1T	2	2	0	
HIST1H2AM	2	2	0	
HIST1H2BN	2	2	0	
HIST1H2BO	2	2	0	
HIST1H4D	2	2	0	
HIST1H4H	2	2	0	
HK2	2	0	2	
HMOX1	2	2	0	
HP	2	0	2	
HUS1	2	2	0	
IER3	2	0	2	
IL11	2	2	0	
IL12B	2	2	0	
IL1RN	2	2	0	
IRGQ	2	2	0	
KATNB1	2	0	2	
KDM4C	2	2	0	
KDM5B	2	2	0	
KIAA0753	2	2	0	
KIAA1324	2	2	0	
KIF18A	2	2	0	
KLK13	2	2	0	
KLK2	2	2	0	
KRT5	2	0	2	
LSS	2	2	0	
MAP3K5	2	0	2	
MARCH6	2	2	0	
MAST4	2	0	2	
MAU2	2	2	0	
MEAF6	2	2	0	
MKNK1	2	2	0	
MSX2	2	2	0	
NCLN	2	2	0	
NIPA2	2	0	2	

NIPBL	2	2	0	
NPPB	2	2	0	
NR2C2	2	2	0	
NR4A1	2	2	0	
NR5A1	2	2	0	
ODF1	2	0	2	
OGFR	2	0	2	
OGT	2	2	0	
OLFM1	2	2	0	
OPA3	2	0	2	
OR2B6	2	2	0	
OVOL1	2	2	0	
OVOL2	2	2	0	
PARD6B	2	2	0	
PARP2	2	0	2	
PER3	2	0	2	
PHLPP1	2	2	0	
PHYH	2	2	0	
PIK3R3	2	2	0	
PLAGL2	2	2	0	
PLEKHM1	2	2	0	
PLLP	2	2	0	
PLXNA2	2	2	0	
PMF1	2	2	0	
PRDM10	2	2	0	
PRL	2	2	0	
PROC	2	2	0	
PRPSAP1	2	0	2	
PSG9	2	2	0	
PSMA6	2	2	0	
PTMS	2	2	0	
PTPN21	2	2	0	
PWP1	2	0	2	
RALGAPA1	2	2	0	
RALGAPB	2	0	2	
RANBP17	2	2	0	
RCHY1	2	2	0	
RETSAT	2	0	2	
RHOT1	2	0	2	
RNF24	2	2	0	
RTF1	2	0	2	
SKIL	2	2	0	
SLC25A4	2	2	0	
SLC2A4	2	2	0	
SLC39A9	2	2	0	
SLC4A7	2	2	0	
SLC4A8	2	0	2	
SLC6A14	2	2	0	

SMARCD2	2	0	2	
SMC5	2	2	0	
SORBS1	2	2	0	
SOX15	2	2	0	
SPATA20	2	2	0	
SPR	2	2	0	
SPRED2	2	2	0	
SRRM2	2	2	0	
SRSF5	2	2	0	
SS18L1	2	0	2	
ST3GAL4	2	2	0	
STK17A	2	2	0	
STK3	2	2	0	
STRN	2	2	0	
SYNE2	2	0	2	
SYNJ2	2	2	0	
TBC1D1	2	2	0	
TCF25	2	2	0	
TCF3	2	2	0	
TFAP2A	2	2	0	
TFCP2L1	2	0	2	
TGFB2	2	2	0	
THBS1	2	2	0	
TLE4	2	2	0	
TMEM97	2	0	2	
TMF1	2	2	0	
TMPRSS2	2	2	0	
TNFAIP2	2	2	0	
TOMM20	2	0	2	
TRA2A	2	2	0	
TSC22D2	2	2	0	
TSPAN5	2	0	2	
UBAP2L	2	2	0	
UBE2C	2	0	2	
UBR4	2	2	0	
UCN	2	2	0	
UHRF1BP1L	2	2	0	
VCPIP1	2	2	0	
VIM	2	0	2	
VRK3	2	2	0	
WISP2	2	2	0	
WSB2	2	0	2	
WWTR1	2	2	0	
XIAP	2	2	0	
XPO4	2	2	0	
ZDHHC6	2	0	2	
ZFP30	2	0	2	
ZFP36L1	2	2	0	

ZFX	2	2	0	
ZNF324	2	2	0	
ZNF335	2	2	0	
ZNF442	2	2	0	
ZNF500	2	0	2	
ZNF609	2	2	0	
ZNF682	2	2	0	
ZNF750	2	2	0	

Complex 2

mRNA based prediction result

Pa	Pi	DownRegulation	Pa	Pi	UpRegulation
0.843	0.020	TRAF5	0.863	0.035	SFRP1
0.790	0.056	TPM1	0.828	0.039	SQLE
0.758	0.065	ADORA2B	0.758	0.065	PFKFB2
0.758	0.065	CDCA4	0.758	0.065	SH3BGRL
0.758	0.065	CENPJ	0.758	0.065	TRIM13
0.758	0.065	DNAJC9	0.718	0.051	ST3GAL1
0.758	0.065	DONSON	0.669	0.015	COX5B
0.758	0.065	HAUS8	0.696	0.068	UGT2B15
0.758	0.065	MCMBP	0.664	0.041	CALM1
0.758	0.065	NUP155	0.693	0.083	ALDOA
0.758	0.065	PARP2	0.673	0.067	KRT6A
0.758	0.065	RMI1	0.688	0.084	CACNG4
0.758	0.065	SCARA3	0.688	0.084	CHRNE
0.758	0.065	SLC16A14	0.650	0.055	WISP2
0.758	0.065	SUV39H2	0.688	0.109	GFRA1
0.739	0.065	GSS	0.660	0.081	EXOSC4
0.696	0.056	NSF	0.643	0.071	FADS1
0.674	0.063	OSBP2	0.619	0.058	SCGB2A1
0.687	0.078	CAPRIN1	0.616	0.064	CYTH1
0.674	0.082	PLEC	0.573	0.041	PSMD6
0.637	0.048	ABCC5	0.619	0.096	KRT34
0.689	0.107	C9ORF40	0.539	0.035	NDUFB2
0.597	0.039	SLTM	0.603	0.105	GAS6
0.597	0.039	ZBTB43	0.590	0.093	PTGER4
0.658	0.101	RPRM	0.572	0.079	CHEK1
0.605	0.063	B4GALT5	0.507	0.015	RARG
0.626	0.085	ERCC2	0.614	0.126	HIVEP1
0.634	0.095	MFAP4	0.618	0.136	PPP1CB
0.643	0.106	CXCL2	0.614	0.138	C1ORF63
0.617	0.087	CXCR7	0.537	0.063	GAL
0.614	0.085	PPP1CA	0.505	0.031	UGT2B17
0.591	0.065	PRIMA1	0.545	0.075	SPATA20
0.571	0.053	EFNA3	0.592	0.131	DNAJB14
0.562	0.053	SRPK2	0.568	0.107	MT1B
0.636	0.128	TMSB15A	0.564	0.108	LINC00467
0.592	0.085	TNIK	0.563	0.109	KCNH2
0.575	0.071	TBL1XR1	0.563	0.109	PLLP
0.533	0.034	CAB39	0.563	0.109	SLC25A4
0.569	0.073	PGM2L1	0.563	0.109	ZYX
0.578	0.092	MYNN	0.561	0.118	CASP2
0.567	0.086	L1CAM	0.503	0.074	IL1RN
0.536	0.057	ILF2	0.503	0.088	PDS5B
0.620	0.143	HIG2	0.556	0.142	OLFM1
0.561	0.094	LGALS1	0.548	0.137	GATA1

0.522	0.059	STK39	0.535	0.129	MGST1
0.541	0.087	DIO2	0.532	0.128	OLFML3
0.568	0.115	RARB	0.504	0.115	HMMR
0.563	0.109	MAST4	0.535	0.157	SLC4A11
0.563	0.109	MSH5	0.514	0.152	WT1
0.563	0.109	TREX1	0.525	0.162	TFF3
0.581	0.130	SMARCC1	0.509	0.148	CREB3L1
0.551	0.104	RBM12	0.509	0.148	RNASEL
0.550	0.104	DSE	0.525	0.169	KIAA0247
0.514	0.070	UBE2M	0.511	0.165	PLK3
0.538	0.094	LRP6	0.515	0.185	GGH
0.557	0.114	PPIH	0.515	0.185	STMN1
0.534	0.096	POC1A	0.513	0.185	ABHD2
0.540	0.110	TANC1	0.526	0.199	IL10RB
0.515	0.088	ARNT2	0.511	0.187	AURKA
0.541	0.116	COL5A2	0.524	0.212	UBE2C
0.616	0.191	TBC1D9			
0.514	0.089	OSR2			
0.564	0.141	NPM1			
0.528	0.112	NDE1			
0.514	0.109	SCAMP1			
0.572	0.171	H1FX			
0.526	0.130	HPS5			
0.536	0.150	IVNS1ABP			
0.531	0.149	MAPK8			
0.505	0.131	SYMPK			
0.514	0.145	FKBP5			
0.517	0.149	ACSL3			
0.506	0.144	ABCA13			
0.506	0.144	ABCC13			
0.506	0.144	TMEM59L			
0.548	0.198	WDR4			
0.530	0.207	IKBKAP			

Protein based prediction result

Pa	Pi	DownRegulation	Pa	Pi	UpRegulation
0.586	0.154	PPARA	0.596	0.095	CD83
0.509	0.153	VIM			

MCF7 based prediction result

Pa	Pi	DownRegulation	Pa	Pi	UpRegulation
0.793	0.021	FOXM1	0.833	0.013	C11orf68
0.792	0.068	UBE2C	0.810	0.029	CLDN9
0.731	0.023	H2AFJ	0.800	0.020	ZNF672
0.705	0.015	BBS4	0.796	0.037	CLCF1

0.682	0.038	SUCO	0.763	0.005	STRN
0.686	0.042	SBF1	0.767	0.015	CDKN1B
0.668	0.029	C3orf52	0.761	0.028	ABCB6
0.627	0.012	ACVR2B	0.759	0.033	BIN1
0.627	0.012	BID	0.747	0.028	CBR3
0.627	0.012	ERGIC2	0.724	0.020	RANGAP1
0.634	0.024	MRPL34	0.730	0.045	KIAA0232
0.618	0.038	FKBPL	0.723	0.040	FOXC1
0.617	0.038	SFN	0.726	0.047	KIAA0430
0.632	0.054	KIF18B	0.655	0.032	RAB3A
0.582	0.020	LRIG1	0.627	0.012	ALAD
0.630	0.068	FZD2	0.627	0.012	CLEC16A
0.622	0.065	PLK1	0.627	0.012	FBXL18
0.605	0.055	ESPL1	0.627	0.012	ZC2HC1A
0.575	0.033	MAST4	0.659	0.050	GPRC5C
0.559	0.025	NEMF	0.661	0.055	WISP2
0.550	0.018	MREG	0.615	0.011	MARCH8
0.570	0.043	ZNF22	0.615	0.011	WIPI2
0.557	0.030	FGFR2	0.633	0.030	TRIM23
0.575	0.054	OGFR	0.614	0.014	PHLPP1
0.581	0.062	NREP	0.617	0.020	PIK3IP1
0.564	0.046	METTL21B	0.624	0.031	LYPD3
0.554	0.040	FAM111A	0.701	0.126	PTMS
0.540	0.026	SMCO4	0.585	0.013	MTUS1
0.553	0.042	TNS3	0.584	0.012	ANGEL2
0.592	0.082	ZNF85	0.588	0.017	ANKZF1
0.595	0.087	DEK	0.598	0.029	SCAND2P
0.565	0.059	PCBP1	0.613	0.050	RAB40B
0.534	0.029	HGSNAT	0.593	0.034	GCLC
0.551	0.049	CGGBP1	0.585	0.027	ZNF200
0.566	0.068	NFE2L3	0.601	0.044	STX3
0.578	0.080	ARL4C	0.641	0.093	CHD3
0.560	0.063	FLJ10038	0.585	0.038	CLIP1
0.518	0.021	GMCL1	0.566	0.020	ATXN7
0.544	0.048	ALOX12P2	0.572	0.026	SORBS1
0.574	0.082	TNNT1	0.579	0.041	FBXL5
0.568	0.076	PANK2	0.546	0.017	CDS2
0.546	0.057	CDKN2C	0.593	0.067	SLC4A7
0.562	0.075	HMMR	0.555	0.029	NPL
0.530	0.044	SPDL1	0.563	0.042	MINOS1P1
0.533	0.049	FAM172A	0.577	0.056	ZNF222
0.526	0.042	AP1AR	0.616	0.098	BTG2
0.548	0.065	KCNG1	0.550	0.033	FBXO5
0.530	0.049	ASUN	0.533	0.018	SPR
0.520	0.040	HEATR6	0.530	0.017	PPP3CC
0.529	0.052	THAP4	0.545	0.032	PRNP
0.514	0.038	MTSS1	0.560	0.048	TFE3

0.512	0.037	NINL
0.521	0.050	BCL2
0.550	0.079	STC2
0.520	0.051	DBP
0.530	0.062	MMACHC
0.520	0.052	URB2
0.512	0.046	CD3EAP
0.509	0.045	ACOX3
0.557	0.093	CYP26A1
0.514	0.050	THG1L
0.511	0.049	NAT1
0.546	0.085	MARCH8
0.530	0.070	MAGEF1
0.511	0.055	RIBC2
0.502	0.046	FAM134A
0.545	0.091	RERE
0.512	0.060	ARMCX1
0.503	0.051	MYD88
0.520	0.069	KIAA0125
0.502	0.051	TRAF3IP2
0.512	0.062	JADE2
0.503	0.053	DBF4
0.505	0.056	HSPBAP1
0.515	0.069	FUT4
0.510	0.067	PDZK1
0.507	0.066	UMPS
0.523	0.082	PSMD12
0.529	0.088	PRRC1
0.530	0.093	ZC3HAV1
0.533	0.103	PUS3
0.562	0.134	GTSE1
0.512	0.085	ZC3H13
0.503	0.078	ARHGEF3
0.547	0.123	PTPN18
0.518	0.096	SIDT1
0.561	0.140	SFSWAP
0.524	0.104	SEC22A
0.507	0.091	TCAF1
0.514	0.099	USP46
0.523	0.111	MYBL1
0.504	0.096	PIK3R1
0.527	0.120	AURKA
0.510	0.110	CHAF1A
0.514	0.121	TGS1
0.560	0.169	C2CD2
0.503	0.116	ADRA2C
0.511	0.125	CPTP

0.572	0.062	PIGA
0.574	0.067	INTS6
0.560	0.053	GABARAPL1
0.534	0.032	LINC00963
0.559	0.057	VAMP2
0.551	0.049	SRGAP3
0.546	0.046	ZFAND5
0.549	0.050	NIPBL
0.592	0.093	IFIT2
0.593	0.097	ZFP36L1
0.529	0.034	PPP3R1
0.522	0.028	LSM14A
0.537	0.045	STK17A
0.582	0.095	PGM1
0.549	0.062	DNAJB2
0.540	0.057	PROCR
0.517	0.034	COQ10B
0.539	0.057	SCYL3
0.526	0.044	OSR2
0.581	0.102	CC2D1A
0.523	0.045	TCEB3-AS1
0.513	0.035	MTMR9
0.508	0.032	KDM6A
0.518	0.044	ZNF654
0.535	0.061	SPAG9
0.537	0.064	HEG1
0.502	0.029	ZHX2
0.502	0.029	PTPN21
0.549	0.077	ZNF217
0.508	0.038	SAMD4A
0.539	0.069	ATG12
0.507	0.041	HIST1H4H
0.517	0.054	PSG9
0.521	0.058	DIP2C
0.569	0.109	BNIP3L
0.509	0.051	MAP3K14
0.508	0.051	RGS17
0.529	0.072	HIST2H2BE
0.510	0.055	WDR47
0.516	0.064	ZNF211
0.534	0.082	ID3
0.512	0.062	TNFAIP2
0.511	0.063	NR4A1
0.539	0.092	CSRP2
0.511	0.064	LDLR
0.543	0.097	KCTD5
0.519	0.073	NCLN

0.541	0.155	FAM208B	0.502	0.057	EFNA1
0.511	0.172	RNF4	0.519	0.076	ADM
			0.526	0.083	HMOX1
			0.534	0.098	SNHG17
			0.502	0.067	MCL1
			0.548	0.120	FOSL1
			0.526	0.099	NKX3-1
			0.584	0.158	KLF2
			0.515	0.091	PBXIP1
			0.513	0.096	NFKBIB
			0.542	0.128	DUSP1
			0.577	0.163	MSRB2
			0.533	0.121	KLF10
			0.505	0.094	ELF3
			0.522	0.115	IER5
			0.547	0.144	PLA2G3
			0.502	0.100	MSANTD2
			0.523	0.122	IFIT5
			0.502	0.109	BHLHE40

VCAP_6h based prediction result

Pa	Pi	DownRegulation	Pa	Pi	UpRegulation
0.855	0.024	ZNF221	0.841	0.030	SCMH1
0.819	0.044	CACNA1B	0.816	0.048	SLC38A2
0.801	0.048	SLC12A6	0.813	0.052	AKIP1
0.792	0.052	KCNC2	0.758	0.040	MIS12
0.778	0.051	NPLOC4	0.781	0.084	BCS1L
0.769	0.046	DYNC1H1	0.750	0.062	SP3
0.770	0.057	BACH2	0.789	0.104	SMG5
0.723	0.017	HP	0.742	0.065	KPNA2
0.767	0.069	C10ORF68	0.749	0.083	SNRPB2
0.749	0.060	CPM	0.745	0.080	MRPL4
0.770	0.086	LRRC8E	0.702	0.048	UNC50
0.762	0.081	ADM	0.730	0.081	ZDHHC4
0.724	0.055	PCYOX1	0.723	0.080	WDR74
0.734	0.066	CXORF56	0.672	0.030	CBR3
0.722	0.057	KANK2	0.737	0.099	TIMM9
0.715	0.052	HIBCH	0.730	0.094	HGSNAT
0.746	0.091	ASTN1	0.716	0.087	SNX17
0.736	0.081	LASP1	0.701	0.074	PALLD
0.750	0.096	CLDN7	0.687	0.065	ISOC1
0.737	0.085	ZNF23	0.699	0.082	RAB9A
0.686	0.034	CENPC1	0.674	0.065	MRPL48
0.749	0.098	MGC12488	0.677	0.069	PCTP
0.729	0.080	KIAA0664L3	0.686	0.085	ACVR2A

0.734	0.087	CLCN2	0.685	0.086	FUBP3
0.743	0.099	SPIN1	0.690	0.093	NDUFS3
0.698	0.060	SNIP1	0.688	0.096	CTSK
0.704	0.073	GTF2H2B	0.675	0.087	PDCD10
0.670	0.042	C20ORF46	0.679	0.091	CTCF
0.711	0.092	SLC6A4	0.691	0.118	BABAM1
0.700	0.091	DSC3	0.648	0.075	TXN2
0.660	0.052	ROBO4	0.637	0.065	DNAJC7
0.690	0.083	OASL	0.637	0.066	FAM59A
0.662	0.055	GABRA2	0.642	0.071	BFAR
0.672	0.070	PRR5L	0.632	0.067	ZNF580
0.697	0.101	ACSL4	0.633	0.069	NCAPD3
0.702	0.106	TMOD3	0.637	0.074	POLE3
0.683	0.088	LOC646934	0.671	0.109	RAD23A
0.678	0.086	CXXC1	0.697	0.138	CAD
0.690	0.098	ST8SIA1	0.637	0.079	TACC2
0.657	0.066	HSPB2	0.637	0.084	MFAP1
0.744	0.156	DCAF17	0.628	0.075	NSMCE4A
0.676	0.091	PCP4	0.635	0.082	DHX32
0.681	0.096	TSPYL4	0.613	0.061	TOR3A
0.645	0.060	KAZALD1	0.658	0.106	MDK
0.710	0.125	PFN2	0.702	0.153	MCAT
0.667	0.084	C3	0.626	0.078	MRPL24
0.657	0.075	ABCC5	0.647	0.101	FANCG
0.689	0.107	KLKB1	0.642	0.097	PCID2
0.656	0.076	FXC1	0.653	0.117	MMP7
0.671	0.092	FAM46C	0.657	0.122	TGDS
0.633	0.055	HIST1H1D	0.647	0.115	KIAA1324
0.671	0.096	MGLL	0.602	0.074	HDHD1
0.686	0.112	MAGEA11	0.663	0.136	PRMT1
0.658	0.085	MYOC	0.656	0.131	HNRNPA0
0.708	0.135	COL6A3	0.620	0.096	TDRD7
0.670	0.098	GFRA2	0.633	0.111	GPATCH2
0.658	0.087	LHPP	0.601	0.081	SCO2
0.642	0.074	SERPINI1	0.648	0.128	MRPL35
0.633	0.067	HIVEP3	0.581	0.063	CNNM4
0.625	0.060	LIPC	0.606	0.090	GUCY1A3
0.689	0.124	FRG1	0.649	0.134	PSMD7
0.657	0.092	ABCA12	0.543	0.028	ABCF3
0.604	0.040	SH3BP1	0.599	0.085	PTPN9
0.638	0.074	COMMD3	0.613	0.100	ALAS1
0.645	0.082	ADH7	0.633	0.123	TMEM123
0.672	0.111	TGM3	0.569	0.064	LIMA1
0.627	0.070	UPF2	0.610	0.109	RAP1B
0.623	0.069	TLR2	0.611	0.112	STRN4
0.620	0.067	TPP1	0.654	0.156	SSRP1
0.656	0.103	C1ORF114	0.618	0.119	TMEM93

0.623	0.071	KIAA0485
0.631	0.080	EPHB2
0.621	0.070	TCOF1
0.665	0.118	CETP
0.654	0.108	TMEM30B
0.605	0.060	CEACAM8
0.694	0.153	EBF2
0.665	0.124	SELL
0.627	0.088	STX7
0.655	0.116	APOBEC3G
0.656	0.118	ECHDC3
0.674	0.136	APOBEC3C
0.631	0.094	ICAM4
0.614	0.077	ULBP2
0.632	0.097	KPNA5
0.663	0.129	LRRC6
0.612	0.078	DNMT3B
0.598	0.065	DDX43
0.637	0.105	INSL4
0.630	0.097	KRT5
0.614	0.082	TLR8
0.615	0.084	CGREF1
0.601	0.075	WNT11
0.640	0.116	KIF13B
0.596	0.075	RPL11
0.608	0.087	RDH16
0.624	0.104	ATAT1
0.588	0.071	LTBP2
0.581	0.066	BAGE
0.615	0.102	GABRB3
0.622	0.108	BMPR2
0.587	0.073	APOM
0.586	0.073	LRIG1
0.619	0.109	LHX2
0.652	0.142	FAM168A
0.601	0.092	IQGAP2
0.659	0.150	SERPINB7
0.658	0.150	AZU1
0.622	0.116	ATP1B3
0.602	0.097	HYAL3
0.609	0.103	MEG3
0.637	0.133	SYNPO
0.577	0.072	DUSP2
0.587	0.083	UNC93B1
0.639	0.135	C4ORF29
0.618	0.115	ODF1
0.653	0.151	LAMA5
0.584	0.087	NTHL1
0.612	0.116	RSAD2
0.592	0.097	IRX4
0.598	0.103	TIMM44
0.653	0.159	ETFA
0.587	0.093	UQCRFS1
0.565	0.071	DHCR24
0.549	0.056	DLD
0.635	0.143	C1ORF216
0.584	0.094	COQ9
0.568	0.079	TMSB15A
0.599	0.113	CENPA
0.618	0.133	PODXL2
0.600	0.118	FJX1
0.569	0.089	LAP3
0.631	0.152	USP21
0.573	0.095	MX1
0.577	0.100	AUH
0.567	0.091	SH3GLB2
0.571	0.095	CEP70
0.559	0.084	RHBDF1
0.588	0.115	GREM1
0.614	0.142	NCAPH
0.570	0.100	GBAS
0.588	0.119	DPYSL2
0.596	0.127	TAF11
0.545	0.076	ASS1
0.548	0.080	RAD21
0.606	0.139	CCT5
0.558	0.093	FCER1A
0.589	0.125	G6PD
0.521	0.058	IQGAP1
0.554	0.093	OIP5
0.565	0.107	RAB4A
0.569	0.111	TNFAIP8
0.589	0.131	FBXL6
0.549	0.092	XAF1
0.589	0.132	ZNF12
0.549	0.093	SCFD1
0.539	0.083	ADCY7
0.582	0.126	DERL1
0.560	0.105	UBE2Q1
0.542	0.087	LSR
0.597	0.144	UBXN8
0.572	0.120	FASTKD1
0.557	0.107	FAM193B
0.529	0.081	OAS3

0.605	0.103	SLIT2
0.599	0.097	ZXDC
0.581	0.081	FZD8
0.590	0.091	PER2
0.606	0.107	DDX6
0.591	0.091	CTSL2
0.620	0.121	UTY
0.612	0.113	POLQ
0.630	0.131	CORO1C
0.588	0.091	SLPI
0.645	0.147	PTH1R
0.576	0.079	USP14
0.577	0.082	TWF2
0.646	0.151	CRYGD
0.569	0.074	WIPF2
0.683	0.188	SERPIND1
0.565	0.071	POLR3D
0.610	0.115	ATP5J
0.575	0.082	TFCP2L1
0.577	0.085	FMNL1
0.571	0.079	PLTP
0.565	0.073	DUX1
0.611	0.120	PURA
0.589	0.098	LEF1
0.598	0.108	FAH
0.604	0.113	OXT
0.633	0.143	C11ORF16
0.590	0.100	MIS18BP1
0.579	0.090	PLA2G2D
0.568	0.079	KIAA0754
0.607	0.118	SSR3
0.546	0.058	PSG7
0.570	0.082	KDSR
0.572	0.085	NKX2-2
0.583	0.095	ZNF711
0.597	0.110	CYBRD1
0.600	0.113	EIF4H
0.595	0.109	PKNOX1
0.634	0.148	MRPL24
0.559	0.074	RAB11FIP1
0.609	0.125	CPEB3
0.540	0.056	FPGS
0.565	0.081	SLC13A2
0.566	0.084	PEX13
0.605	0.123	FYB
0.567	0.086	ZNF473
0.548	0.068	KLRC3

0.546	0.100	PTPRE
0.543	0.096	HSD17B6
0.596	0.151	ILF2
0.556	0.112	RABGGTA
0.563	0.120	RDBP
0.616	0.174	ECSIT
0.536	0.094	CLP1
0.530	0.089	PIK3CA
0.581	0.140	KIAA0101
0.570	0.130	GATAD2A
0.543	0.104	TGFBI
0.579	0.141	TIMM13
0.569	0.132	MTMR2
0.533	0.096	GRB7
0.540	0.104	HNRNPC
0.536	0.102	IL10RB
0.541	0.107	IFI44L
0.531	0.098	VPS37C
0.510	0.077	PUF60
0.578	0.146	GALNS
0.575	0.143	MRPL16
0.569	0.138	BET1
0.503	0.075	ATP11B
0.597	0.172	PTGES2
0.531	0.107	SERPINH1
0.525	0.102	IFIT3
0.522	0.101	STAG2
0.533	0.114	IFIT1
0.554	0.136	ARPP21
0.540	0.123	MTCP1NB
0.544	0.127	KIAA1598
0.516	0.099	TBPL1
0.553	0.137	RAB32
0.514	0.101	NUSAP1
0.540	0.126	GPD1L
0.546	0.132	SLC29A3
0.553	0.140	ADAR
0.517	0.107	POLE2
0.537	0.129	C1QTNF1
0.574	0.167	PRC1
0.521	0.115	XKR8
0.532	0.127	FAM110B
0.549	0.145	ANXA2
0.578	0.174	NQO2
0.553	0.150	AKR7A3
0.524	0.121	DIEXF
0.530	0.127	NDUFB3

0.551	0.071	CD244	0.551	0.148	TRAPPC2L
0.596	0.117	PFKP	0.587	0.187	UNG
0.584	0.105	MBD5	0.531	0.132	FAAH
0.552	0.074	MPV17	0.502	0.103	PTPRC
0.598	0.119	ERLIN2	0.520	0.123	FAM127B
0.600	0.122	HCP5	0.532	0.135	TRIP4
0.601	0.124	NUBP1	0.512	0.118	LAMTOR2
0.637	0.162	HOOK1	0.535	0.142	COPZ1
0.587	0.112	MALL	0.530	0.138	DDX54
0.560	0.086	HLA-DMB	0.512	0.122	MCM6
0.637	0.163	ZNF556	0.517	0.130	TPM4
0.594	0.121	PAQR6	0.549	0.164	YARS2
0.580	0.106	PDK4	0.512	0.128	GTF3C1
0.617	0.144	RAMP3	0.563	0.180	GABRP
0.595	0.122	LGSN	0.528	0.146	CLEC4A
0.568	0.095	SLC26A6	0.542	0.162	GMEB2
0.597	0.125	STOM	0.524	0.144	SLCO4A1
0.535	0.063	SERPINC1	0.519	0.140	MSH2
0.611	0.140	CD247	0.529	0.149	CLK3
0.511	0.039	FOXN2	0.541	0.162	DCTPP1
0.598	0.128	P2RY13	0.519	0.140	UQCRC2
0.591	0.121	VAV3	0.521	0.142	SPDEF
0.606	0.137	KCNK15	0.505	0.127	PEX2
0.554	0.085	CRTAM	0.513	0.136	TMED9
0.554	0.085	PER3	0.556	0.179	SPECC1L
0.552	0.085	B2M	0.509	0.133	PSMA6
0.586	0.120	ASH2L	0.533	0.158	BLOC1S1
0.552	0.087	INPP5B	0.527	0.152	TSR1
0.547	0.082	NOP56	0.523	0.149	ARHGEF5
0.538	0.074	LPA	0.515	0.140	UBE2D3
0.535	0.072	GLI3	0.504	0.131	TEAD4
0.612	0.149	CCL18	0.507	0.135	NIF3L1
0.612	0.149	FNDC3B	0.532	0.160	VASP
0.577	0.115	FIP1L1	0.524	0.156	RAP2C
0.583	0.121	LOC399904	0.527	0.162	POLD2
0.586	0.124	ASAP3	0.553	0.191	TIMM10
0.602	0.141	CCR6	0.506	0.146	CCDC51
0.560	0.100	LRMP	0.554	0.195	C19ORF60
0.582	0.123	LOC220594	0.509	0.150	IFI6
0.550	0.091	TEAD1	0.531	0.172	LRRC47
0.613	0.155	KCNS3	0.519	0.161	NKRF
0.551	0.095	MLF1	0.501	0.149	TM4SF1
0.510	0.054	CYP4F2	0.523	0.171	CNOT6
0.562	0.106	RPS3	0.520	0.168	INTS7
0.543	0.088	MXD1	0.503	0.152	BBS4
0.546	0.090	PGM1	0.537	0.188	SDHAP1
0.553	0.098	CTSW	0.528	0.179	TNFRSF1A

0.563	0.108	ZNF552
0.546	0.091	NAV2
0.579	0.126	SCYL2
0.566	0.114	REPS2
0.568	0.116	TBCC
0.553	0.101	PAK3
0.613	0.162	HHAT
0.589	0.138	WDR52
0.566	0.116	NADSYN1
0.552	0.102	GABPA
0.568	0.118	TLK1
0.529	0.081	AFF4
0.516	0.068	ZNF835
0.579	0.131	COL7A1
0.568	0.120	HBQ1
0.549	0.102	CALU
0.560	0.113	ZNF516
0.589	0.142	ZMYM5
0.573	0.126	MUC16
0.509	0.063	NRG2
0.577	0.131	KRT3
0.578	0.133	HAND1
0.576	0.131	LOC100127972
0.585	0.140	TMEM140
0.535	0.090	SNAPC1
0.516	0.072	CRYGC
0.599	0.156	MAGEA6
0.578	0.136	TMEM50B
0.502	0.061	CLIC3
0.516	0.075	FRK
0.560	0.119	PAAF1
0.520	0.079	SNORA21
0.573	0.133	MSN
0.549	0.109	PPWD1
0.558	0.117	ALS2CL
0.595	0.155	ALAS2
0.553	0.113	RPS25
0.545	0.105	KIAA1109
0.551	0.112	BAMBI
0.544	0.104	GALNT11
0.521	0.082	TPI1
0.586	0.148	PNPLA2
0.512	0.073	DHX38
0.603	0.165	NME4
0.625	0.188	KIAA1024
0.569	0.132	MCC
0.590	0.153	SNTA1

0.535	0.186	AKR1A1
0.530	0.184	HDGF
0.501	0.157	WAC
0.519	0.175	PRPSAP2
0.539	0.197	PLEKHM2
0.536	0.196	CHI3L2
0.530	0.190	MMD
0.525	0.187	CCDC109B
0.506	0.168	AES
0.508	0.172	ZFP36L2
0.502	0.168	CERS6
0.512	0.184	DAD1
0.516	0.190	SUMO2
0.520	0.197	OLA1
0.505	0.187	MCM7
0.517	0.206	RWDD2A
0.519	0.209	CYC1
0.504	0.196	DOCK1
0.508	0.201	MOSC1

0.607	0.170	ACSM3
0.526	0.090	PPFIA1
0.598	0.163	ZNF253
0.539	0.104	PARL
0.539	0.105	GP1BB
0.556	0.122	A4GALT
0.579	0.146	CCR10
0.515	0.083	SNRPD2
0.588	0.156	C14ORF135
0.607	0.175	CLSTN2
0.564	0.132	FCN3
0.540	0.108	AATF
0.551	0.119	CFD
0.548	0.116	CLDN4
0.571	0.139	ELAVL2
0.549	0.118	GOLGA3
0.562	0.131	PIGT
0.579	0.149	IZUMO4
0.516	0.086	RFC4
0.543	0.113	S1PR1
0.554	0.125	TMEM8A
0.541	0.112	OSMR
0.546	0.117	DNM1
0.514	0.086	FOLR2
0.537	0.109	GPR88
0.502	0.075	POLG2
0.549	0.123	YTHDC1
0.532	0.106	RPL27
0.568	0.143	ATAD3A
0.523	0.099	IER3IP1
0.557	0.134	USP47
0.559	0.136	TMEM214
0.538	0.115	MED23
0.506	0.083	ZMAT3
0.600	0.177	CCL21
0.516	0.094	DNM3
0.535	0.113	GVINP1
0.554	0.132	TPBG
0.546	0.124	HTR3A
0.502	0.080	DHRS2
0.548	0.128	ITGA10
0.515	0.095	TAF4B
0.546	0.126	TM7SF2
0.550	0.133	HIST1H4D
0.513	0.097	ONECUT2
0.519	0.103	NTN3
0.507	0.091	MAST4

0.519	0.105	KRT8P12	
0.585	0.170	KIAA0513	
0.548	0.134	EPYC	
0.539	0.126	TFF1	
0.542	0.129	SHOC2	
0.589	0.176	ART3	
0.510	0.098	ARPC1A	
0.530	0.119	ZNF136	
0.536	0.125	OXA1L	
0.558	0.147	NACAP1	
0.563	0.152	TPPP	
0.550	0.140	MYOG	
0.573	0.163	INS	
0.514	0.104	SON	
0.561	0.152	RAB11FIP5	
0.502	0.093	ARMC6	
0.511	0.102	PIP	
0.566	0.157	NPTX1	
0.559	0.151	PROX1	
0.519	0.111	ACSL1	
0.522	0.114	STATH	
0.547	0.141	SATB2	
0.534	0.129	ENPP1	
0.514	0.109	HIST1H4B	
0.555	0.150	MYLK3	
0.579	0.175	C2ORF49	
0.521	0.118	AASS	
0.539	0.136	RPL6	
0.554	0.150	F9	
0.546	0.144	SUZ12P	
0.525	0.124	URGCP	
0.581	0.180	SUSD4	
0.536	0.136	SMTN	
0.527	0.127	C7ORF44	
0.559	0.160	MAT1A	
0.547	0.148	LINC00312	
0.538	0.139	PPAT	
0.578	0.180	KCTD7	
0.509	0.111	RPL3	
0.571	0.173	GSTCD	
0.534	0.136	NR4A1	
0.574	0.177	STAT5B	
0.565	0.169	HOXB5	
0.545	0.148	RUSC2	
0.503	0.107	ASAP1-IT1	
0.522	0.126	STMN1	
0.523	0.127	ACAP2	

0.547	0.151	DDHD2	
0.519	0.126	LSAMP	
0.542	0.149	COL2A1	
0.504	0.112	EMX2	
0.555	0.163	PFKFB4	
0.517	0.125	CEP76	
0.548	0.156	RNF25	
0.506	0.115	GPKOW	
0.510	0.119	LSM3	
0.513	0.123	MAGED1	
0.564	0.176	MST4	
0.543	0.155	HLA-DOB	
0.512	0.125	PCDHA3	
0.539	0.151	EPM2AIP1	
0.505	0.117	SPP1	
0.551	0.164	BBC3	
0.519	0.132	FICD	
0.558	0.171	TRAV8-3	
0.523	0.136	SP140	
0.531	0.144	SSH1	
0.530	0.144	EHD1	
0.531	0.145	TPST1	
0.535	0.150	ITIH2	
0.511	0.128	ITK	
0.522	0.139	GORASP1	
0.528	0.146	ABCA3	
0.513	0.131	TSPYL1	
0.545	0.165	HIST1H2AJ	
0.518	0.138	DERL2	
0.501	0.121	RPL34	
0.519	0.140	SNRNP200	
0.561	0.183	SCAF8	
0.527	0.149	SSTR3	
0.543	0.165	TNFRSF13B	
0.514	0.136	POLD2	
0.551	0.173	DIO1	
0.524	0.148	CLK2	
0.520	0.144	WRB	
0.563	0.187	AMN	
0.520	0.144	NFYA	
0.511	0.136	SSFA2	
0.537	0.164	PIK3CG	
0.533	0.160	TBKBP1	
0.525	0.152	B4GALNT1	
0.501	0.128	APOBEC3F	
0.508	0.136	NDUFV2	
0.528	0.155	PRTN3	

0.518	0.146	C7ORF10
0.523	0.151	RPL17
0.512	0.141	PPP2R5D
0.546	0.176	C2CD2
0.523	0.153	CTSA
0.517	0.148	ADAMTS5
0.520	0.151	RPL37
0.519	0.151	GTF2A1
0.528	0.162	FAM164A
0.551	0.185	HLA-DMA
0.520	0.155	NDP
0.521	0.156	NIT2
0.508	0.143	REM1
0.521	0.157	HAS1
0.535	0.172	TESK2
0.516	0.152	RAPGEF2
0.535	0.171	DDX5
0.533	0.169	SSBP1
0.519	0.156	TYRP1
0.517	0.154	DCUN1D1
0.507	0.144	HAUS3
0.504	0.142	FABP5
0.526	0.164	C8ORF44
0.532	0.169	STAT2
0.501	0.139	LPHN3
0.545	0.185	C4ORF34
0.540	0.180	PHTF2
0.529	0.169	DRG1
0.508	0.148	MAX
0.514	0.155	MIR22HG
0.520	0.161	SLC17A1
0.515	0.157	TBC1D1
0.524	0.166	BNIP3L
0.525	0.169	DYNLT1
0.522	0.166	NEK1
0.538	0.182	TNFSF9
0.510	0.156	STARD13
0.501	0.148	EDNRB
0.504	0.151	GPR171
0.531	0.178	EAF2
0.501	0.148	PINK1
0.515	0.163	POLM
0.504	0.153	RUFY2
0.550	0.199	C14ORF45
0.535	0.185	ANKZF1
0.535	0.184	DNASE1L2
0.503	0.154	KRT34

0.509	0.161	TPP2	
0.507	0.161	PPARG	
0.510	0.164	ZBED5	
0.546	0.201	KCNE4	
0.505	0.159	SETX	
0.519	0.174	PAX6	
0.505	0.161	LGALS9	
0.516	0.172	ATF7IP2	
0.544	0.201	RBFOX1	
0.522	0.179	ZCCHC14	
0.518	0.176	B4GALT2	
0.521	0.180	FKSG49	
0.503	0.163	NUP43	
0.503	0.164	PTPN2	
0.501	0.163	TASP1	
0.501	0.163	C2ORF18	
0.501	0.164	DICER1	
0.523	0.187	SEC22A	
0.504	0.167	NDST3	
0.514	0.179	LRRC20	
0.510	0.175	AKAP12	
0.503	0.168	ZNF528	
0.514	0.181	GFPT2	
0.512	0.180	HECTD3	
0.522	0.190	PSD4	
0.503	0.171	HERC6	
0.503	0.172	ALPL	
0.502	0.173	ATP6V1H	
0.509	0.180	SIPA1	
0.528	0.200	DHRS9	
0.514	0.187	ANXA10	
0.511	0.187	MEIS3P1	
0.513	0.189	MAGEA5	
0.513	0.190	SCAMP1	
0.528	0.206	LMBRD1	
0.505	0.184	TSFM	
0.508	0.188	PRKCH	
0.509	0.189	RPL23AP53	
0.504	0.184	TCHH	
0.510	0.192	MICAL2	
0.511	0.193	WIPF1	
0.504	0.186	IL2RG	
0.515	0.200	POR	
0.536	0.227	CCNJ	
0.522	0.217	PHF21A	
0.508	0.216	PDK1	
0.502	0.212	CCL16	

0.510	0.230	GNG12

VCAP_24h based prediction result

Pa	Pi	DownRegulation	Pa	Pi	UpRegulation
0.850	0.038	DLL3	0.816	0.039	SF3B5
0.845	0.037	ELANE	0.823	0.056	NDUFAB1
0.838	0.036	TRAT1	0.792	0.042	FAM57A
0.803	0.020	TSGA10	0.776	0.051	SLC50A1
0.779	0.026	SERHL2	0.785	0.062	POP7
0.776	0.028	DPF1	0.759	0.048	MRTO4
0.795	0.048	DNAJC13	0.767	0.061	SOAT1
0.802	0.055	SMR3B	0.735	0.053	EXOSC4
0.796	0.051	DKKL1	0.723	0.060	DPAGT1
0.786	0.042	CLCA4	0.727	0.066	SPEF1
0.838	0.098	DIO1	0.726	0.066	TOLLIP
0.798	0.067	DSG3	0.684	0.027	NME1
0.778	0.047	SH3D21	0.695	0.042	TIMM9
0.770	0.040	GNA14	0.701	0.052	GADD45G
0.770	0.041	AKR1B10	0.706	0.076	NDUFV1
0.752	0.027	ARL14	0.712	0.083	POLR3K
0.767	0.045	TBKBP1	0.694	0.066	GOLPH3
0.748	0.028	NRN1	0.691	0.064	PLA2G4A
0.772	0.055	CALML4	0.679	0.057	DBI
0.760	0.047	ELF3	0.680	0.058	OSBPL9
0.791	0.082	CCK	0.689	0.068	SPHK1
0.776	0.066	ERAP2	0.674	0.057	CYB561D2
0.739	0.048	TMEM100	0.670	0.056	F5
0.726	0.038	CRADD	0.664	0.054	LRRC6
0.731	0.043	C1ORF61	0.699	0.090	MCMBP
0.750	0.067	FAM5C	0.680	0.071	TRAF2
0.716	0.036	LARS	0.662	0.055	C14ORF135
0.730	0.051	EHHADH	0.671	0.064	CDC6
0.725	0.046	LOC220594	0.696	0.090	ATP5G3
0.714	0.043	EFNA3	0.665	0.062	GOLPH3L
0.730	0.062	FGF13	0.654	0.052	USP47
0.742	0.080	APH1B	0.649	0.048	NCAPG
0.704	0.042	REG3A	0.668	0.067	FADD
0.745	0.083	AKTIP	0.665	0.064	MLPH
0.723	0.063	EPHX3	0.676	0.080	NCOA3
0.711	0.064	DPEP1	0.666	0.071	CGA
0.686	0.039	CHKB	0.643	0.048	CDR2L
0.697	0.052	LOC100507009	0.716	0.122	ASS1
0.719	0.076	ADIPOR1	0.627	0.033	RPIA
0.718	0.077	DCUN1D2	0.688	0.094	TRMT12
0.683	0.046	SLC35A5	0.688	0.095	THOP1

0.699	0.063	ENPP2	0.668	0.075	GPATCH1
0.701	0.066	PANX1	0.682	0.091	ZMPSTE24
0.713	0.079	PAMR1	0.645	0.058	VEGFC
0.682	0.049	LRRC8D	0.649	0.062	CCDC19
0.694	0.064	FOXG1	0.668	0.083	UBE2D3
0.690	0.063	TPK1	0.684	0.099	CRISPLD2
0.730	0.103	PIEZO2	0.654	0.071	RPS8
0.690	0.063	LACTB2	0.691	0.108	NDUFB2
0.687	0.061	ISL1	0.650	0.069	C17ORF85
0.671	0.052	PDGFD	0.649	0.069	HSD17B1
0.698	0.083	GABARAPL1	0.669	0.089	RFPL2
0.684	0.070	AHNAK	0.659	0.080	SEPHS2
0.683	0.071	C16ORF88	0.637	0.065	EIF3L
0.690	0.078	RSBN1	0.657	0.087	BOLA1
0.675	0.063	LINC00341	0.660	0.091	PTCD3
0.675	0.064	NUDT2	0.618	0.049	NXT1
0.697	0.087	SLC26A4	0.635	0.067	FAM69A
0.670	0.060	SATB1	0.662	0.095	OAS1
0.669	0.060	AKR1C3	0.649	0.084	S100PBP
0.669	0.064	AQR	0.654	0.091	TMEM184B
0.704	0.098	CAPN3	0.629	0.066	NAT10
0.686	0.081	TMED7	0.648	0.086	TNFSF4
0.691	0.086	C12ORF48	0.667	0.106	ACAD8
0.695	0.090	DYNC1H1	0.657	0.096	SOX12
0.672	0.069	ECH1	0.629	0.068	RANBP6
0.671	0.068	C15ORF44	0.627	0.067	CXCL13
0.668	0.065	WFS1	0.634	0.074	ACTR3
0.655	0.053	MORC3	0.668	0.108	TMEM231
0.655	0.055	IER3IP1	0.618	0.058	C8ORF55
0.655	0.056	INS	0.638	0.079	EIF2C3
0.670	0.071	CTRB2	0.667	0.108	TUBA1B
0.658	0.060	TGIF1	0.649	0.090	C5ORF22
0.716	0.121	ALAS2	0.662	0.103	SRSF4
0.670	0.075	KCNG1	0.641	0.083	TARP
0.682	0.088	CCHCR1	0.639	0.082	GABPA
0.641	0.047	ARID5B	0.625	0.068	FRAT2
0.679	0.085	SLC4A1AP	0.648	0.091	ADRA2A
0.648	0.057	TCEA2	0.640	0.083	NUCB2
0.692	0.107	EVPL	0.628	0.072	PC
0.624	0.038	AZI1	0.661	0.105	SFT2D2
0.697	0.113	MYLK3	0.662	0.107	MAP4K2
0.652	0.071	PMP22	0.613	0.058	GSTO1
0.650	0.070	C6ORF145	0.648	0.094	CNIH4
0.666	0.086	UBAP2	0.617	0.063	CD2AP
0.648	0.068	ARHGAP4	0.644	0.090	HBXIP
0.659	0.079	AIFM1	0.651	0.098	ISL1
0.653	0.074	MLH1	0.637	0.085	GPAA1

0.635	0.057	METTL22	0.635	0.084	XBP1
0.669	0.092	ADCY7	0.632	0.081	TIPARP
0.659	0.083	RABEP2	0.625	0.075	CTSC
0.638	0.063	UQCC	0.622	0.072	SEPT8
0.654	0.078	PVRL2	0.626	0.077	NIT2
0.633	0.059	SLC35A1	0.654	0.106	TMEM14B
0.646	0.071	ABCD4	0.637	0.090	CTR9
0.629	0.056	ZNF710	0.619	0.073	GALE
0.670	0.097	SMA4	0.625	0.082	C14ORF133
0.654	0.082	YEATS4	0.596	0.053	RPP25
0.682	0.110	ACADS	0.641	0.098	AP3S1
0.667	0.096	FTO	0.643	0.102	MAD2L1
0.684	0.114	KIAA1024	0.633	0.092	PDE9A
0.646	0.077	PDS5A	0.613	0.072	PIP
0.652	0.084	SCFD1	0.626	0.086	NHP2
0.660	0.094	KRT3	0.627	0.087	ZNF629
0.624	0.057	NHEJ1	0.640	0.101	CDT1
0.654	0.088	MAP6D1	0.641	0.102	TBCB
0.640	0.074	OXA1L	0.619	0.081	FAM106A
0.615	0.050	FAM110B	0.623	0.085	MT1P2
0.692	0.127	UGT8	0.620	0.082	FBL
0.640	0.074	DYNC1LI1	0.628	0.092	DKFZP547G183
0.666	0.101	PLA2G15	0.606	0.070	CCND2
0.626	0.062	POT1	0.590	0.056	HOMER2
0.676	0.112	HTN1	0.631	0.097	HPCAL1
0.625	0.062	KCNIP1	0.623	0.090	MALT1
0.658	0.095	PIGL	0.640	0.108	KIF18A
0.638	0.075	RSL24D1	0.615	0.084	ZRSR2
0.636	0.074	COL17A1	0.612	0.081	FAM179B
0.615	0.053	MEP1A	0.643	0.112	U2AF1
0.631	0.070	PNKP	0.631	0.100	TNFRSF13B
0.646	0.085	COL9A3	0.581	0.053	PLK1
0.648	0.087	ACTR10	0.621	0.094	FLJ13197
0.668	0.107	MAP1B	0.595	0.068	PABPC1
0.654	0.094	CLDN18	0.637	0.110	PES1
0.642	0.082	SERPINE2	0.593	0.067	C21ORF91
0.663	0.104	TNFRSF1B	0.571	0.045	TCFL5
0.643	0.084	TMEM161A	0.593	0.068	CHIC2
0.644	0.085	FAM171A1	0.590	0.066	FAM168B
0.613	0.055	AARS	0.620	0.096	CSNK1G3
0.638	0.081	OPA1	0.605	0.083	ICAM2
0.607	0.050	APOBEC1	0.587	0.066	ATG2B
0.652	0.095	MAGEB2	0.616	0.095	FZD5
0.641	0.086	ASPM	0.627	0.106	DDA1
0.641	0.086	SDAD1	0.605	0.084	SLC13A2
0.655	0.101	HDHD1	0.612	0.091	EIF2C2
0.636	0.083	TBCCD1	0.603	0.083	NME7

0.632	0.080	UBE2S	0.599	0.079	PRMT1
0.641	0.089	MEIS1	0.612	0.092	EFHC1
0.627	0.076	DCAF6	0.607	0.088	FAM171A1
0.608	0.058	PFKM	0.603	0.086	RBM23
0.653	0.104	SLC35C2	0.594	0.078	KCTD3
0.721	0.172	GATA4	0.597	0.082	MRPL17
0.628	0.080	TMBIM4	0.597	0.082	ITGAV
0.619	0.072	ETHE1	0.633	0.119	THYN1
0.640	0.093	PTCRA	0.626	0.114	FSTL3
0.677	0.130	S100A3	0.605	0.093	SH3GL1
0.599	0.055	LMO7	0.595	0.083	VAPB
0.635	0.092	PTCD3	0.618	0.108	DCTPP1
0.624	0.080	PIPOX	0.621	0.111	LRRK1
0.625	0.082	PGRMC2	0.568	0.058	VPS26A
0.632	0.090	VSNL1	0.591	0.082	FAM178A
0.614	0.071	MTMR11	0.623	0.115	MYH10
0.622	0.080	USP3	0.620	0.113	LRRC17
0.652	0.112	ANO3	0.608	0.101	CRNKL1
0.659	0.119	RWDD3	0.599	0.093	PLA1A
0.608	0.070	NT5DC2	0.639	0.133	ATP5O
0.674	0.136	AQP6	0.592	0.087	MRPL12
0.682	0.145	C2ORF44	0.632	0.129	OBFC2B
0.606	0.069	EXOSC5	0.596	0.093	FAM118A
0.610	0.075	TUFT1	0.601	0.098	CCNO
0.608	0.074	CRIP1	0.631	0.129	ANXA3
0.648	0.114	ATAD2B	0.619	0.119	PAX6
0.628	0.095	ABTB2	0.604	0.105	MAN1C1
0.610	0.079	CD96	0.627	0.129	RABGGTA
0.649	0.119	MX2	0.574	0.076	CCT6A
0.618	0.088	SGTA	0.554	0.057	RSRC1
0.620	0.091	CDC14B	0.586	0.090	SETMAR
0.593	0.065	PRG4	0.592	0.095	PROC
0.614	0.086	LOC100506469	0.594	0.098	GBP1
0.646	0.119	SLC43A3	0.593	0.097	CCL21
0.610	0.083	GAP43	0.596	0.100	FAM50B
0.588	0.062	HDAC5	0.564	0.068	NOLC1
0.648	0.122	CCDC41	0.580	0.084	PDE1A
0.588	0.065	ERN2	0.571	0.075	CAD
0.603	0.080	HDAC7	0.586	0.090	FKBP10
0.613	0.091	TSPAN1	0.582	0.087	TKT
0.621	0.099	FLRT3	0.562	0.067	LCN2
0.648	0.128	MED13L	0.596	0.101	HEXB
0.617	0.098	TNS3	0.601	0.108	CARHSP1
0.622	0.105	CCNG2	0.606	0.113	NR2C2
0.612	0.096	IARS	0.606	0.114	PPA1
0.628	0.113	ZNF22	0.564	0.072	KLHL9
0.615	0.100	ETFDH	0.566	0.074	ATAD3A

0.569	0.054	PPCDC	0.580	0.089	FZD10
0.602	0.087	ABCA3	0.573	0.082	CHL1
0.581	0.067	NOL6	0.639	0.148	TPST2
0.591	0.077	LOC100286895	0.603	0.113	C2ORF28
0.613	0.099	FEM1B	0.580	0.091	SIDT1
0.613	0.100	PARP4	0.609	0.120	MRPL4
0.613	0.100	FZD7	0.595	0.106	QSER1
0.632	0.119	OPA3	0.607	0.118	IL21R
0.585	0.073	GPA33	0.572	0.085	METTL18
0.602	0.090	ADAM10	0.577	0.091	EPM2AIP1
0.610	0.099	EPRS	0.593	0.107	NFE2L1
0.606	0.096	ZNF12	0.606	0.120	MAK
0.627	0.117	RAB28	0.612	0.127	IQGAP2
0.600	0.091	PHGDH	0.599	0.114	PPP4R2
0.575	0.067	PLCD1	0.569	0.084	MFAP1
0.598	0.090	FAIM2	0.598	0.113	IGKV3-20
0.591	0.084	IFNGR1	0.583	0.098	UBE2A
0.595	0.088	ZG16	0.558	0.073	DERL2
0.590	0.083	RNF41	0.610	0.127	UBN1
0.597	0.090	GTPBP3	0.571	0.088	LHX1
0.612	0.105	IL17RB	0.554	0.072	ORMDL2
0.611	0.105	DERL1	0.571	0.089	KEAP1
0.611	0.106	CD3EAP	0.594	0.113	FBXL8
0.612	0.106	RMND1	0.626	0.147	CSTF3
0.594	0.089	TOM1L1	0.600	0.121	AKR1A1
0.587	0.082	PYROXD1	0.578	0.098	PILRB
0.651	0.147	EDEM1	0.567	0.088	BRMS1
0.636	0.132	BEX1	0.566	0.087	SPAG1
0.640	0.137	PNRC1	0.591	0.113	INSM1
0.626	0.123	SLC48A1	0.619	0.142	DEXI
0.627	0.124	FAM55C	0.590	0.113	SFXN3
0.577	0.074	IREB2	0.615	0.139	SLCO4A1
0.597	0.094	UNC5B	0.585	0.109	PRRX1
0.642	0.139	HBD	0.576	0.101	DAD1
0.581	0.079	ITGA6	0.605	0.130	ACAP1
0.596	0.094	DAK	0.601	0.127	RETN
0.595	0.094	RPS9	0.578	0.104	LINC00342
0.596	0.096	PIAS1	0.546	0.072	CTDSP1
0.577	0.077	F8	0.564	0.091	ICAM3
0.537	0.037	TRIM17	0.564	0.091	COPS6
0.616	0.116	GNAL	0.577	0.105	TM9SF3
0.605	0.105	LTK	0.596	0.124	DYNLT1
0.581	0.083	C10ORF88	0.570	0.098	SCGB1D2
0.589	0.092	RGS2	0.580	0.108	C7ORF28B
0.610	0.113	PLEKHA1	0.606	0.134	PSMC1
0.593	0.096	NCKAP1	0.573	0.102	CSTF1
0.567	0.070	C9ORF82	0.602	0.131	C1ORF106

0.606	0.110	CKAP5	0.575	0.105	BRE
0.587	0.091	SNX15	0.604	0.133	CYP27B1
0.617	0.121	SMARCC2	0.541	0.071	RDH14
0.602	0.106	LOC283683	0.556	0.086	RRAGA
0.582	0.087	FADD	0.587	0.117	ZBBX
0.619	0.124	PFDN5	0.567	0.098	CCDC15
0.590	0.095	KIAA0922	0.596	0.127	CD38
0.594	0.100	IFITM3	0.535	0.067	RPL34
0.603	0.110	LHPP	0.564	0.096	CRNN
0.588	0.095	MARK4	0.565	0.097	MMADHC
0.628	0.135	CDKN2AIP	0.589	0.121	C6ORF120
0.584	0.092	LOC100129361	0.588	0.121	TSSC1
0.603	0.111	C5AR1	0.569	0.103	KIFC1
0.605	0.113	PLS3	0.586	0.120	CCNB1
0.596	0.104	VAT1	0.553	0.087	INPP1
0.548	0.057	CERS4	0.566	0.102	GRRP1
0.555	0.064	DNMT1	0.576	0.113	XAB2
0.601	0.110	SLC18A2	0.572	0.108	ACOT8
0.565	0.075	DNAJA4	0.565	0.102	PTRH2
0.582	0.092	FADS3	0.582	0.119	TTC30A
0.565	0.076	LRRC59	0.574	0.112	KIF3B
0.613	0.124	PTGIR	0.565	0.103	TUBBP5
0.609	0.120	SLC7A10	0.586	0.124	C3
0.584	0.097	RPL28	0.583	0.121	HIST1H4B
0.629	0.143	CEPT1	0.573	0.112	PFKP
0.585	0.099	NECAP2	0.584	0.123	LARS2
0.577	0.091	RHOT1	0.569	0.108	MLXIPL
0.578	0.092	MAST2	0.598	0.137	TRIM9
0.591	0.105	FLNA	0.562	0.102	MYBL2
0.578	0.093	CEACAM6	0.586	0.126	PDE5A
0.547	0.063	TINF2	0.589	0.129	TMSB10
0.577	0.093	GNB2L1	0.574	0.115	CORO2A
0.588	0.104	STMN1	0.583	0.125	ZSCAN16
0.575	0.091	NIF3L1	0.547	0.090	NID2
0.584	0.101	KRT5	0.570	0.113	SLMO2
0.582	0.099	C4ORF27	0.513	0.056	CDC20
0.556	0.074	DRAM1	0.561	0.105	ZNF10
0.599	0.117	HMGCS2	0.562	0.105	ERG
0.583	0.103	CITED2	0.562	0.106	EMID1
0.572	0.092	BCORL1	0.572	0.116	HELLS
0.607	0.127	PINK1	0.542	0.086	CLEC11A
0.589	0.110	C13ORF27	0.586	0.130	ZBED1
0.606	0.127	TFF2	0.589	0.133	FOXH1
0.572	0.093	RPH3AL	0.565	0.110	PPP6R3
0.560	0.081	TPMT	0.572	0.117	PROS1
0.577	0.098	AASS	0.565	0.110	PRRC2B
0.583	0.105	KIAA0355	0.623	0.169	IMPDH2

0.549	0.071	NSMAF	0.549	0.094	BTBD3
0.600	0.122	SNX24	0.550	0.096	PSMA6
0.592	0.114	SMARCD1	0.571	0.117	PTX3
0.601	0.124	EAF2	0.563	0.109	GOLGA4
0.600	0.123	IRS2	0.575	0.122	ID1
0.562	0.084	GTF2E2	0.569	0.117	UBB
0.581	0.104	COPB2	0.565	0.113	LIMS3
0.588	0.111	LYN	0.550	0.098	MOGS
0.565	0.089	AGXT2L1	0.582	0.131	ATP5L
0.588	0.113	CST3	0.593	0.143	OFD1
0.546	0.071	SMYD5	0.586	0.136	CHMP4A
0.564	0.089	ATP11A	0.574	0.123	AKR1D1
0.556	0.081	IER3	0.565	0.115	EIF3M
0.576	0.102	TMEM41B	0.532	0.083	PELO
0.581	0.107	PCTP	0.570	0.121	CHAC1
0.602	0.128	SALL1	0.576	0.128	TULP1
0.587	0.113	TNRC6B	0.561	0.113	ZNF124
0.586	0.112	CLSTN1	0.569	0.121	PIAS3
0.589	0.115	PDE8B	0.580	0.133	GOLGA8A
0.553	0.080	SLC41A3	0.567	0.121	ZMIZ1
0.614	0.141	BDNF	0.576	0.130	IVNS1ABP
0.553	0.081	PPIE	0.577	0.132	FJX1
0.556	0.086	EMP1	0.554	0.109	RRP8
0.566	0.095	SGPP1	0.541	0.096	TDO2
0.571	0.101	FJX1	0.540	0.094	ORC1
0.617	0.146	YPEL5	0.552	0.107	BASP1
0.559	0.089	MAP1S	0.562	0.118	COQ4
0.539	0.069	FOXF2	0.556	0.111	PUS7
0.558	0.089	SPEG	0.559	0.115	DGKZ
0.579	0.109	PSPH	0.566	0.122	ZNF337
0.590	0.121	PDK4	0.555	0.112	TPR
0.567	0.098	EEF1B2	0.576	0.133	FGF17
0.595	0.126	SLTM	0.605	0.162	SNRPE
0.537	0.069	LPPR3	0.554	0.112	PRSS8
0.571	0.103	BAG4	0.564	0.122	C15ORF5
0.534	0.067	RHOA	0.559	0.118	TSPAN13
0.552	0.085	TLR2	0.570	0.129	SLC9A3R2
0.562	0.096	USP11	0.556	0.116	CPEB3
0.560	0.093	IMPACT	0.550	0.109	CCL5
0.567	0.101	NENF	0.538	0.099	MORF4L2
0.590	0.124	SMARCE1	0.546	0.107	BATF3
0.553	0.087	TCTN2	0.550	0.111	PPP2R3C
0.584	0.119	PGRMC1	0.557	0.119	MAP3K11
0.592	0.127	LGR4	0.545	0.108	RAI14
0.568	0.103	LIN7A	0.545	0.108	CENPF
0.571	0.106	TUSC3	0.548	0.111	SLC26A6
0.559	0.094	TNFRSF12A	0.526	0.091	PLP2

0.562	0.098	RPS6KA3	0.565	0.130	DNAL4
0.551	0.088	UGT2A3	0.556	0.121	MT1H
0.564	0.101	C9ORF40	0.545	0.111	GIMAP6
0.581	0.118	OAZ2	0.543	0.109	NIP7
0.580	0.117	CDK5RAP2	0.556	0.123	G3BP2
0.579	0.117	RALBP1	0.562	0.130	UBXN1
0.529	0.067	LYZ	0.558	0.127	DLGAP5
0.589	0.128	SPRR1A	0.539	0.107	ANKRD36
0.606	0.145	TUBGCP4	0.547	0.116	TMPRSS2
0.536	0.075	DHRS3	0.550	0.120	FANCI
0.560	0.100	CAD	0.552	0.122	NUMA1
0.566	0.105	UIMC1	0.526	0.096	CADPS2
0.601	0.141	HR	0.563	0.134	SEMG1
0.570	0.110	SLC29A3	0.555	0.126	PNMA1
0.575	0.116	ITGAX	0.558	0.130	SLC22A18AS
0.565	0.106	GRB10	0.552	0.124	SORL1
0.552	0.093	CTCF	0.543	0.115	ASL
0.565	0.107	TERF2IP	0.520	0.092	IFI44
0.607	0.149	AHCTF1	0.570	0.142	ZFAND1
0.575	0.118	YLPM1	0.551	0.124	DDI2
0.545	0.088	PCMTD2	0.535	0.109	IGFBP4
0.608	0.152	GLCE	0.529	0.104	KDELC1
0.620	0.164	IGKV4-1	0.547	0.123	GTF2A2
0.580	0.124	DENND4B	0.528	0.105	DZANK1
0.600	0.144	CD1A	0.572	0.148	TPX2
0.584	0.130	CHMP1A	0.562	0.138	TTLL3
0.556	0.101	B3GNT1	0.542	0.119	LTA4H
0.567	0.113	STRN3	0.544	0.121	FMO1
0.546	0.091	ARHGAP15	0.548	0.125	KRT8P12
0.581	0.128	FAM114A1	0.533	0.110	SAT1
0.570	0.117	RUVBL1	0.527	0.105	PISD
0.575	0.122	ATP5B	0.551	0.129	CLK2
0.565	0.113	SCMH1	0.548	0.127	IRF1
0.557	0.105	WRN	0.555	0.135	AURKA
0.522	0.070	ODAM	0.519	0.099	PLOD2
0.588	0.137	RRAS2	0.539	0.119	CNDP2
0.561	0.109	LTBR	0.557	0.138	JTB
0.562	0.110	ASAP1	0.538	0.119	TNNC1
0.553	0.101	ATP6V0A1	0.541	0.122	DUSP13
0.570	0.118	TMEM97	0.565	0.147	PRR7
0.544	0.093	MECR	0.517	0.100	TMEM185B
0.564	0.114	U2AF1	0.535	0.118	ELAVL3
0.586	0.136	GSTM4	0.547	0.132	HDGF
0.560	0.110	CHERP	0.538	0.123	RRP7A
0.551	0.101	CIDEB	0.536	0.121	MXD1
0.551	0.102	OSGEP	0.544	0.129	C11ORF58
0.565	0.116	ACTA1	0.540	0.126	UAP1L1

0.557	0.109	DPYSL2	0.524	0.109	ESF1
0.530	0.083	FOXO3	0.533	0.118	KIAA0355
0.549	0.102	NAT2	0.553	0.138	FOXC2
0.594	0.147	L1TD1	0.533	0.118	ZNF165
0.554	0.108	RPL23	0.518	0.103	ASPM
0.546	0.100	ABCA8	0.559	0.146	IST1
0.548	0.102	APPL1	0.522	0.110	SLC12A1
0.562	0.117	UBE2D2	0.535	0.123	PALMD
0.574	0.129	RB1CC1	0.573	0.161	GPX1
0.564	0.119	UBAP1	0.524	0.113	MRPS31
0.547	0.102	MTCP1NB	0.548	0.137	WDR59
0.542	0.097	DOCK4	0.540	0.129	PSD3
0.577	0.133	HOXD11	0.518	0.107	HYMAI
0.548	0.104	CYP3A5	0.515	0.104	SCAMP3
0.565	0.121	RCE1	0.549	0.139	ABHD10
0.558	0.115	DUSP8	0.525	0.115	CIB1
0.546	0.102	MORF4L2	0.541	0.131	DFNA5
0.571	0.127	MTUS1	0.526	0.117	VAX2
0.562	0.120	FAM82A2	0.542	0.133	MYC
0.574	0.132	TAF1C	0.518	0.110	ZNF185
0.601	0.159	YIPF5	0.532	0.124	LIMK2
0.608	0.166	LOC100127972	0.559	0.151	HIST1H2BM
0.556	0.114	C6ORF108	0.542	0.134	ARFIP1
0.544	0.102	ZNF219	0.531	0.124	BTN3A3
0.576	0.135	AP3M2	0.515	0.109	NDUFB11
0.587	0.146	TEX261	0.560	0.155	MARCH5
0.548	0.108	SERPINI1	0.550	0.145	MEIS2
0.549	0.109	HAUS6	0.539	0.134	LEFTY2
0.555	0.116	CNOT6	0.529	0.123	POLI
0.573	0.134	SEC61G	0.544	0.140	C22ORF29
0.518	0.079	ALPK3	0.532	0.129	LOC644450
0.602	0.165	AQP9	0.547	0.144	RPP14
0.550	0.114	LAD1	0.533	0.130	NFS1
0.530	0.094	POF1B	0.530	0.127	ARNT2
0.552	0.116	ADIPOR2	0.528	0.125	IFITM3
0.568	0.133	KARS	0.528	0.126	IFIT1
0.556	0.121	HHIPL2	0.529	0.128	POGK
0.528	0.093	SLC6A4	0.535	0.134	ZNF573
0.550	0.115	ZNF358	0.551	0.150	ZBTB48
0.555	0.121	ATXN2	0.507	0.107	ZNF268
0.556	0.121	MYO1C	0.518	0.119	FOXM1
0.558	0.124	APBA2	0.542	0.144	PODXL2
0.536	0.103	RNF44	0.539	0.141	PTPRK
0.556	0.123	MAK16	0.541	0.143	TENC1
0.558	0.125	COL11A1	0.573	0.175	CCR9
0.558	0.126	ADAM17	0.533	0.136	MALL
0.526	0.094	ELOVL2	0.514	0.118	ASCC1

0.552	0.120	MOB3B
0.549	0.118	MAPT
0.522	0.091	JAK1
0.565	0.134	KCTD5
0.572	0.142	CUL4A
0.531	0.101	UBE2K
0.574	0.144	CRYL1
0.572	0.142	C16ORF7
0.537	0.107	C20ORF4
0.546	0.115	PSMG2
0.590	0.161	ABR
0.533	0.104	PPL
0.538	0.109	SEC24B
0.562	0.133	TTC33
0.551	0.123	RALGPS1
0.524	0.095	GATAD1
0.565	0.138	MTMR4
0.541	0.114	MYL12A
0.544	0.117	RCBTB1
0.536	0.110	ABCB4
0.542	0.116	NDUFAF4
0.569	0.143	CSDE1
0.515	0.088	PPP2R5A
0.520	0.094	DCAF17
0.543	0.118	PLP1
0.553	0.127	GPRC5A
0.518	0.093	ALAS1
0.550	0.124	CREBL2
0.540	0.115	MFF
0.528	0.104	RPL29
0.528	0.103	SPINK4
0.548	0.124	UGGT2
0.571	0.147	LINC00472
0.530	0.106	CDO1
0.528	0.105	RPS8
0.566	0.143	PLEKHB1
0.523	0.099	RTN4
0.544	0.121	COL6A2
0.529	0.107	SREK1IP1
0.544	0.123	ODF2
0.571	0.150	CACNB3
0.522	0.101	CSGALNACT2
0.535	0.114	HSPA13
0.541	0.121	PIM1
0.521	0.101	BRIX1
0.564	0.144	TPM2
0.549	0.129	PRPH

0.527	0.130	IDH3B
0.524	0.128	PAF1
0.536	0.140	HSPA8
0.542	0.147	ARPC5
0.512	0.117	PHF10
0.521	0.125	CFB
0.515	0.121	SLC25A37
0.513	0.119	TBP
0.502	0.108	TXNL1
0.528	0.134	UCP2
0.508	0.115	CASP3
0.528	0.135	CSN1S1
0.509	0.116	SSB
0.544	0.154	TRMT1
0.510	0.120	DYM
0.564	0.174	SNRPB2
0.533	0.144	SMTN
0.507	0.117	CENPM
0.538	0.149	CENPB
0.516	0.127	PFDN2
0.513	0.125	SIX1
0.533	0.144	CDK18
0.554	0.166	ITM2B
0.525	0.137	NUP43
0.539	0.151	PCDHA10
0.517	0.130	FRAT1
0.533	0.146	PCDHGA9
0.519	0.132	COL6A3
0.504	0.117	ITFG1
0.515	0.129	GNA12
0.528	0.143	DYSF
0.511	0.126	TRIM36
0.520	0.135	CCNE2
0.546	0.162	IL36A
0.529	0.145	DAAM1
0.530	0.146	TPRKB
0.527	0.143	GPR20
0.504	0.120	TFAP2A
0.515	0.132	C14ORF109
0.531	0.147	GIMAP4
0.534	0.151	EPHB1
0.530	0.148	ZNF204P
0.501	0.120	SEPX1
0.534	0.153	PLK4
0.515	0.135	ARL8B
0.542	0.162	HAPLN1
0.516	0.136	FAM46C

0.540	0.121	RPS29	0.535	0.157	SLC9A2
0.523	0.103	HMGCR	0.529	0.152	TNN
0.560	0.141	IRF5	0.519	0.142	CLN6
0.505	0.086	RRP1B	0.512	0.135	FER
0.515	0.096	CETP	0.523	0.146	COLQ
0.540	0.121	C12ORF10	0.517	0.140	TMEM156
0.542	0.124	GSN	0.543	0.167	SF3A2
0.526	0.108	APTX	0.515	0.139	TGFBRAP1
0.571	0.153	ODF1	0.534	0.159	ATP6V0E2
0.539	0.122	NFKBIA	0.509	0.134	P2RY10
0.596	0.179	ZNF124	0.528	0.154	GCM1
0.555	0.139	SLC9A5	0.524	0.150	SLC37A1
0.557	0.140	CCNJ	0.510	0.137	BCLAF1
0.559	0.143	RIPK4	0.507	0.134	KIF3A
0.520	0.103	PRRG1	0.529	0.156	COL5A1
0.549	0.133	HERC2	0.520	0.148	GYPE
0.542	0.126	PCP4	0.540	0.169	TNMD
0.559	0.144	TBL1XR1	0.511	0.140	KIAA1661
0.599	0.184	PHLDA3	0.526	0.155	HIST1H1B
0.538	0.123	RPL9	0.523	0.152	FGF3
0.526	0.111	TRAP1	0.521	0.150	PER3
0.552	0.137	NEFM	0.506	0.135	NADSYN1
0.505	0.091	ABHD3	0.514	0.144	MTIF2
0.528	0.114	RPS7	0.522	0.152	CLDN8
0.587	0.173	CRY2	0.524	0.154	ZNF783
0.514	0.100	RPS3	0.522	0.153	ETV1
0.566	0.152	EPB49	0.525	0.157	PAX1
0.557	0.145	ROD1	0.521	0.153	EFCAB1
0.551	0.138	PLXNA1	0.511	0.143	C20ORF46
0.593	0.181	RHBG	0.509	0.141	RIPK4
0.553	0.143	VCAN	0.529	0.161	NEUROG3
0.524	0.114	TFCP2	0.506	0.139	REC8
0.594	0.184	CBY1	0.510	0.142	PLAGL1
0.549	0.140	RPS6	0.503	0.135	PORCN
0.503	0.095	CEP192	0.515	0.148	WDR52
0.535	0.126	GDAP1L1	0.508	0.141	LOC390940
0.540	0.131	MAN2B2	0.502	0.135	SH3D21
0.535	0.127	C9ORF46	0.521	0.155	CYP39A1
0.527	0.119	H2BFS	0.506	0.141	C1ORF54
0.522	0.115	MYLK	0.517	0.152	HSPA4
0.512	0.105	WSCD1	0.520	0.156	FABP5
0.556	0.149	PHF16	0.527	0.162	CNPPD1
0.515	0.109	TCEA1	0.517	0.152	IDO1
0.527	0.122	PDIA4	0.529	0.165	REPIN1
0.570	0.165	SOSTDC1	0.504	0.141	CPNE7
0.559	0.154	ENOX2	0.512	0.149	PKN2
0.547	0.142	BCL7A	0.531	0.168	IL24

0.522	0.118	RPL13	0.520	0.157	ESRP2
0.556	0.152	RASSF4	0.528	0.165	PDE4B
0.525	0.121	ZFAND5	0.511	0.150	SATB2
0.510	0.107	BRF2	0.503	0.142	OTUD4
0.610	0.207	SH3BP1	0.503	0.142	MKKS
0.514	0.111	RPL36A	0.518	0.158	CKB
0.520	0.118	DNAJB9	0.520	0.160	NAT6
0.537	0.135	CHST2	0.520	0.160	SCGB2A1
0.508	0.106	SNX6	0.512	0.153	ICA1
0.517	0.116	ARFIP2	0.519	0.160	SLC13A4
0.572	0.171	MARCH8	0.532	0.173	FBN1
0.549	0.149	ATP6V0D1	0.511	0.152	RPS14P3
0.536	0.137	NFKBIL1	0.514	0.155	HOXB1
0.525	0.125	ANXA7	0.522	0.163	ARHGEF7
0.531	0.132	DTWD1	0.505	0.148	ANKRD12
0.524	0.125	OSBPL1A	0.507	0.149	DDIT4
0.564	0.166	MAP1LC3C	0.510	0.153	FBXW4P1
0.539	0.141	BBC3	0.511	0.153	TMEM30B
0.581	0.183	SMCHD1	0.507	0.150	SRPX
0.524	0.127	CYR61	0.501	0.145	RPS2P45
0.538	0.141	CHMP3	0.535	0.179	SCARF1
0.564	0.167	HNRNPH2	0.514	0.159	HDAC6
0.574	0.178	DNMBP	0.501	0.146	EYA4
0.533	0.136	WDR3	0.506	0.151	HIST1H2BC
0.555	0.159	MUC5AC	0.502	0.148	C19ORF2
0.567	0.172	PCYOX1L	0.505	0.151	TRIM33
0.533	0.138	IDH3G	0.508	0.155	XK
0.512	0.117	HOXC10	0.503	0.150	GPR97
0.518	0.123	USP14	0.522	0.170	FFAR3
0.551	0.156	HIF1AN	0.524	0.173	HIST1H1D
0.538	0.143	ARHGEF2	0.516	0.164	LAMTOR2
0.508	0.114	TRAPPC6A	0.514	0.162	KCNC3
0.519	0.125	SERPINA4	0.517	0.166	SIX5
0.518	0.124	NFYB	0.507	0.155	ZNF280D
0.506	0.112	RPL6	0.514	0.163	ITPR1
0.570	0.176	AOAH	0.513	0.162	SUV39H2
0.544	0.150	MEIS3P1	0.520	0.169	MCM5
0.539	0.145	SEMA6A	0.509	0.158	PRKACG
0.536	0.143	METTL2B	0.515	0.165	SH3BGRL3
0.520	0.127	SH3BGRL3	0.510	0.161	FGF23
0.535	0.142	DCTN4	0.501	0.153	RRAD
0.541	0.149	ACSBG1	0.513	0.165	EPHB4
0.540	0.148	STXBP2	0.502	0.155	UCP1
0.541	0.149	FXYD3	0.506	0.158	MGC12488
0.536	0.144	CLIC3	0.515	0.168	PTBP2
0.506	0.114	CLEC11A	0.501	0.154	UBA7
0.509	0.117	DMBT1	0.510	0.163	MPV17

0.521	0.130	CCNC
0.524	0.132	ZC3H14
0.551	0.160	CD80
0.535	0.144	TMSB10
0.504	0.114	PRPF8
0.517	0.127	CAMTA1
0.544	0.154	CIC
0.515	0.126	WDR74
0.522	0.133	LIAS
0.542	0.154	BTBD1
0.519	0.132	BBS7
0.541	0.154	FERMT1
0.523	0.137	SLC22A18
0.509	0.124	GIMAP5
0.514	0.129	F9
0.528	0.142	ST8SIA5
0.545	0.160	MAGEA12
0.535	0.150	PPM1A
0.529	0.143	VCAM1
0.552	0.168	ARFGAP3
0.512	0.127	KLHDC2
0.530	0.145	APOL1
0.508	0.124	EIF2B3
0.543	0.159	CASP2
0.512	0.129	NSUN5P2
0.527	0.144	DLG1
0.522	0.138	STX10
0.557	0.175	EPB41L2
0.528	0.145	NANOG
0.543	0.161	TAC3
0.508	0.126	HPRT1
0.539	0.157	STC1
0.510	0.128	DMRT1
0.530	0.148	PAK2
0.529	0.148	RPS12
0.566	0.185	FAM8A1
0.520	0.140	PHB
0.537	0.158	IRF6
0.503	0.124	MSL3
0.504	0.125	ANPEP
0.516	0.137	POLD2
0.530	0.151	STK25
0.528	0.149	TMBIM6
0.561	0.182	MITF
0.528	0.150	SCML2
0.519	0.141	GTF3C3
0.521	0.144	SULT1C2

0.518	0.172	CYP11B2
0.509	0.163	ARAP1
0.505	0.159	USP32
0.520	0.174	C1ORF38
0.521	0.176	TCN2
0.509	0.164	DCN
0.502	0.158	WNT7A
0.501	0.157	IMMT
0.518	0.176	PAGE4
0.506	0.164	RAPGEF3
0.503	0.161	CEP170
0.503	0.162	FAM164C
0.501	0.160	TWSG1
0.526	0.185	MTCH2
0.521	0.181	DACH1
0.506	0.166	TYMS
0.506	0.167	MAPRE3
0.506	0.168	PDSS2
0.504	0.166	C10ORF92
0.503	0.165	TTC38
0.508	0.171	KIF24
0.518	0.184	HMGN3
0.519	0.185	ACTA1
0.530	0.197	TRIM5
0.507	0.175	ALPP
0.502	0.173	ASTE1
0.511	0.184	IGLL3P
0.505	0.178	PRR16
0.501	0.175	CDH11
0.501	0.176	RASIP1
0.506	0.184	SMPD3
0.502	0.183	ACTL7A
0.511	0.193	DCPS
0.505	0.189	H2AFZ
0.505	0.191	ECM1
0.504	0.191	ZNF32
0.501	0.190	IRAK1
0.511	0.216	ILF2
0.505	0.231	TRIM37

0.515	0.138	C1ORF183
0.503	0.126	ERF
0.534	0.157	ZNF430
0.528	0.152	MTMR6
0.505	0.129	ETV4
0.514	0.138	NSDHL
0.526	0.151	PNPLA4
0.565	0.190	C17ORF48
0.510	0.135	GTF2A2
0.519	0.144	ACADL
0.521	0.147	ABCB7
0.512	0.138	HIP1R
0.509	0.135	PON3
0.532	0.159	NRSN2
0.534	0.160	SYBU
0.513	0.140	ATMIN
0.519	0.146	ZKSCAN5
0.508	0.135	CD55
0.530	0.157	SLC30A3
0.540	0.167	OFD1
0.535	0.162	RAP2C
0.520	0.148	TNK1
0.523	0.150	CUTC
0.506	0.133	CLCN5
0.523	0.151	SETD1A
0.501	0.129	SAV1
0.505	0.134	FAM86B1
0.514	0.143	FAM60A
0.520	0.149	KIAA0664L3
0.521	0.150	F13A1
0.547	0.177	CUX1
0.502	0.133	PLEKHM2
0.519	0.151	RWDD2B
0.521	0.153	SPINT1
0.504	0.136	TCL1B
0.502	0.135	CCNF
0.523	0.156	ALKBH4
0.501	0.134	COL21A1
0.560	0.194	MOK
0.524	0.158	SUGP2
0.504	0.138	IQCB1
0.502	0.136	DDX31
0.545	0.179	SLC25A24
0.515	0.150	CGA
0.515	0.151	SCAMP4
0.509	0.146	ABCG2
0.506	0.144	RPS13

0.501	0.140	BAHD1
0.503	0.142	RPH3A
0.565	0.205	ECM2
0.531	0.171	MTMR14
0.502	0.142	CA2
0.504	0.144	SNRNP40
0.526	0.169	SUN2
0.506	0.148	MAX
0.536	0.179	CD19
0.501	0.144	SCG5
0.527	0.170	LMF2
0.503	0.147	RAD23B
0.512	0.155	NPL
0.504	0.147	RUNDC3A
0.511	0.155	OPHN1
0.507	0.153	TMEM45A
0.517	0.163	CA11
0.519	0.165	PTPN2
0.533	0.179	MTHFS
0.501	0.148	SYNE2
0.501	0.149	TBC1D17
0.514	0.163	WBP2
0.515	0.164	C3ORF18
0.508	0.158	MOBP
0.502	0.153	RPL21
0.505	0.156	PGM3
0.514	0.167	ZNF142
0.523	0.177	MPZ
0.532	0.186	DUOX1
0.505	0.159	TMCO3
0.511	0.167	MUDENG
0.517	0.173	ARMC1
0.524	0.180	HEY2
0.515	0.173	KRT33A
0.534	0.192	NRGN
0.525	0.184	ZNHIT6
0.509	0.168	CDCP1
0.518	0.177	CLASP1
0.514	0.174	CD27
0.514	0.174	NBR1
0.505	0.165	PCDH8
0.505	0.166	ATG4A
0.503	0.164	WNT5A
0.502	0.164	CPT1A
0.502	0.164	TPRA1
0.510	0.172	PRSS1
0.501	0.163	SUCLG1

0.511	0.176	ALDH5A1	
0.518	0.183	PDGFRL	
0.512	0.178	RAB5B	
0.517	0.185	AIM2	
0.501	0.169	RPL13A	
0.511	0.180	TMOD3	
0.502	0.171	SLC16A4	
0.502	0.171	ZFAND6	
0.545	0.215	NTRK2	
0.505	0.175	SCGB2A2	
0.508	0.180	COQ9	
0.512	0.185	FAU	
0.501	0.177	ZNF529	
0.503	0.179	CCDC85C	
0.505	0.182	HLA-DOB	
0.505	0.184	ASXL1	
0.501	0.181	PPME1	
0.518	0.201	RALGAPB	
0.501	0.183	KIFC3	
0.505	0.193	ENDOD1	
0.524	0.213	HRASLS	
0.509	0.206	GNL1	
0.508	0.212	EPHB6	
0.501	0.213	CA10	

Combination prediction result

Name	Overlap count	Up	Down	Result
ARMCX5	6	3	3	
CHST15	6	2	4	
DEK	6	2	4	
FBXO42	6	3	3	
FKBP5	6	3	3	
GDPD5	6	4	2	
H2AFJ	6	3	3	
IVNS1ABP	6	2	4	
KIAA0753	6	3	3	
PDS5B	6	4	2	
PLEC	6	2	4	
POLI	6	3	3	
PTPN2	6	3	3	
SLC12A2	6	3	3	
TACC1	6	4	2	
TRIM33	6	3	3	
UBE2C	6	3	3	
ZNF45	6	3	3	

AASDHPPT	5	2	3	
ABHD10	5	2	3	
ABHD2	5	3	2	
ABHD3	5	3	2	
ANKZF1	5	2	3	
APPL1	5	2	3	
ASNA1	5	3	2	
ATF6	5	3	2	
BBX	5	2	3	
BCL7C	5	2	3	
BCLAF1	5	3	2	
CAT	5	3	2	
CBR4	5	2	3	
CCNO	5	3	2	
CEP57	5	2	3	
CLCN3	5	2	3	
CLINT1	5	3	2	
COQ9	5	2	3	
CSNK2A1	5	3	2	
CTSB	5	2	3	
DDX52	5	3	2	
DIO2	5	2	3	
DLG1	5	3	2	
DOCK4	5	2	3	
DYRK2	5	2	3	
EAF2	5	2	3	
EFNA3	5	2	3	
EIF5B	5	2	3	
EP300	5	4	1	
FDXR	5	4	1	
FHOD1	5	2	3	
FJX1	5	2	3	
FSCN1	5	3	2	
FTL	5	2	3	
G3BP2	5	3	2	
GABARAPL1	5	3	2	
GABPA	5	3	2	
GALNT6	5	2	3	
GOLGA4	5	3	2	
GOSR1	5	2	3	
GRSF1	5	2	3	
HIBCH	5	2	3	
HIST1H3G	5	3	2	
HSD17B1	5	3	2	
ID1	5	4	1	
IL6ST	5	3	2	
IVL	5	3	2	
KRAS	5	2	3	

KRT10	5	3	2	
KRT18	5	4	1	
LIAS	5	2	3	
MAFB	5	2	3	
MAP3K9	5	3	2	
MED6	5	3	2	
METTL1	5	2	3	
MRPS31	5	2	3	
MTMR4	5	2	3	
MUC5AC	5	3	2	
N4BP2L2	5	2	3	
NACA	5	2	3	
NDE1	5	2	3	
NFKBIL1	5	2	3	
NPPB	5	3	2	
NR3C1	5	2	3	
NUBP1	5	2	3	
OAS1	5	2	3	
OPA1	5	2	3	
PCBP2	5	2	3	
PEX13	5	3	2	
PLAU	5	3	2	
PLSCR1	5	2	3	
PPME1	5	3	2	
PRPH	5	3	2	
PSMD3	5	2	3	
RAB2A	5	2	3	
RANGAP1	5	3	2	
RAPGEF2	5	3	2	
RBM12B	5	2	3	
RGS10	5	3	2	
RIT1	5	3	2	
RNF6	5	2	3	
SAFB2	5	3	2	
SAP30	5	2	3	
SF3A1	5	3	2	
SFTPC	5	3	2	
SIDT1	5	3	2	
SLC25A37	5	2	3	
SLC39A4	5	3	2	
SLC39A8	5	3	2	
SMCHD1	5	2	3	
SPATA5L1	5	2	3	
SREK1	5	3	2	
SSH1	5	2	3	
STK17A	5	3	2	
STK39	5	2	3	
STRN3	5	3	2	

TBC1D1	5	3	2	
THBS1	5	3	2	
TIPRL	5	2	3	
TLK1	5	2	3	
TMPRSS2	5	4	1	
TNFRSF1A	5	3	2	
TOPORS	5	3	2	
TPRA1	5	3	2	
TTI1	5	2	3	
TUBA4A	5	3	2	
UBE2D3	5	2	3	
UCP2	5	2	3	
UNKL	5	3	2	
ZFAND5	5	2	3	
ZNF148	5	3	2	
ZNF184	5	2	3	
ZNF22	5	2	3	
ZNF236	5	3	2	
ZNF239	5	2	3	
ZNF277	5	3	2	
ZNF32	5	2	3	
ABCA11P	4	3	1	
ABCB6	4	3	1	
ABCG1	4	2	2	
ACAP1	4	2	2	
ACD	4	1	3	
ACSL3	4	1	3	
ACTR3	4	2	2	
ACTR3B	4	1	3	
ACTR6	4	1	3	
ADARB1	4	2	2	
ADH1C	4	2	2	
ADNP2	4	2	2	
ADO	4	1	3	
ADORA2B	4	2	2	
AFF1	4	2	2	
AFF4	4	3	1	
AHNAK	4	1	3	
AIMP1	4	1	3	
AIP	4	2	2	
AK2	4	1	3	
AKAP8	4	2	2	
AKIP1	4	1	3	
AKR1A1	4	2	2	
ALAD	4	2	2	
ALDH3B1	4	1	3	
ANKH	4	2	2	
ANKRD12	4	3	1	

ANKRD27	4	2	2	
ANKRD49	4	2	2	
ANO1	4	2	2	
ANPEP	4	2	2	
ANXA2P3	4	2	2	
AP1M2	4	2	2	
AP1S2	4	2	2	
APLNR	4	2	2	
APLP2	4	3	1	
APOE	4	2	2	
APPBP2	4	2	2	
APTX	4	1	3	
ARCN1	4	2	2	
ARFGAP2	4	2	2	
ARFIP1	4	3	1	
ARFIP2	4	2	2	
ARMC6	4	2	2	
ARNT2	4	1	3	
ARPC3	4	2	2	
ASXL1	4	2	2	
ATG4A	4	2	2	
ATG5	4	2	2	
ATIC	4	1	3	
ATP13A2	4	2	2	
ATP5C1	4	3	1	
ATP5O	4	2	2	
ATXN7	4	3	1	
AURKA	4	2	2	
B4GALT3	4	3	1	
BBS7	4	2	2	
BCAS1	4	2	2	
BCL2L1	4	1	3	
BLVRA	4	3	1	
BMI1	4	2	2	
BMPR1A	4	2	2	
BNIP1	4	2	2	
BPIFA1	4	2	2	
BRD9	4	3	1	
BRE	4	2	2	
BRF2	4	2	2	
BTG1	4	3	1	
C14ORF45	4	2	2	
C15ORF5	4	2	2	
C1ORF115	4	2	2	
C1RL	4	2	2	
C3	4	3	1	
C4ORF29	4	2	2	
C5ORF22	4	2	2	

C8ORF4	4	2	2	
C9ORF116	4	2	2	
C9ORF40	4	1	3	
CABYR	4	2	2	
CACNA2D3	4	2	2	
CACYBP	4	1	3	
CAD	4	2	2	
CALM3	4	2	2	
CAMKK2	4	3	1	
CAND1	4	1	3	
CASC3	4	2	2	
CASP6	4	2	2	
CBLL1	4	3	1	
CBR3	4	3	1	
CCDC101	4	1	3	
CCHCR1	4	2	2	
CCL2	4	2	2	
CCNA2	4	2	2	
CCNE2	4	3	1	
CCNG2	4	2	2	
CCNL1	4	2	2	
CCNT1	4	3	1	
CCNT2	4	3	1	
CCT6A	4	1	3	
CD22	4	2	2	
CDC16	4	1	3	
CDC42EP2	4	1	3	
CDCA4	4	2	2	
CDCP1	4	2	2	
CDK16	4	3	1	
CDK17	4	3	1	
CDK19	4	3	1	
CDR1	4	2	2	
CDR2L	4	2	2	
CEP290	4	3	1	
CEP63	4	1	3	
CETP	4	2	2	
CFDP1	4	2	2	
CGGBP1	4	3	1	
CHAF1A	4	3	1	
CHFR	4	2	2	
CHKB	4	2	2	
CHMP1B	4	2	2	
CIAPIN1	4	1	3	
CITED2	4	2	2	
CLASRP	4	2	2	
CLDN15	4	2	2	
CLEC11A	4	2	2	

CLMN	4	2	2	
CNN2	4	2	2	
CNPPD1	4	2	2	
COIL	4	2	2	
COL3A1	4	2	2	
COL5A1	4	2	2	
COL6A2	4	2	2	
COL6A3	4	2	2	
COPS7B	4	2	2	
CORO1C	4	3	1	
COX5B	4	2	2	
CPSF7	4	2	2	
CPT1A	4	2	2	
CRBN	4	2	2	
CREB1	4	3	1	
CREB5	4	2	2	
CREBL2	4	1	3	
CRNKL1	4	1	3	
CRYZ	4	1	3	
CSE1L	4	2	2	
CSRP2	4	2	2	
CSTF3	4	3	1	
CTAGE5	4	2	2	
CTGF	4	2	2	
CTR9	4	2	2	
CTSD	4	1	3	
CTSL2	4	1	3	
CTTN	4	3	1	
CUL4B	4	2	2	
CUZD1	4	1	3	
CYB5R1	4	2	2	
CYFIP2	4	3	1	
CYP11B2	4	3	1	
CYP19A1	4	3	1	
CYP20A1	4	2	2	
CYP26A1	4	2	2	
CYP26B1	4	2	2	
CYR61	4	2	2	
DAZAP2	4	2	2	
DBN1	4	2	2	
DCAF17	4	2	2	
DCP1A	4	2	2	
DCP2	4	1	3	
DCUN1D2	4	2	2	
DDIT4	4	3	1	
DDX1	4	2	2	
DDX19A	4	1	3	
DDX43	4	3	1	

DENND2D	4	2	2	
DERL2	4	2	2	
DHX40	4	1	3	
DIAPH2	4	2	2	
DICER1	4	2	2	
DIEXF	4	1	3	
DIO1	4	2	2	
DIP2C	4	2	2	
DIXDC1	4	2	2	
DLAT	4	2	2	
DLG5	4	2	2	
DNAJA2	4	2	2	
DNAJB12	4	2	2	
DNAJB2	4	1	3	
DNAJB4	4	2	2	
DNASE2	4	1	3	
DNASE2B	4	2	2	
DNMT1	4	1	3	
DPF1	4	2	2	
DR1	4	3	1	
DSC2	4	2	2	
DSE	4	1	3	
DSG2	4	2	2	
DTX4	4	2	2	
DUS4L	4	1	3	
DUSP1	4	3	1	
DUSP3	4	2	2	
DYNC1H1	4	2	2	
ECHDC2	4	2	2	
ECHDC3	4	2	2	
ECM1	4	2	2	
EED	4	2	2	
EEF1B2	4	2	2	
EFHC1	4	2	2	
EHBP1	4	2	2	
EHD1	4	3	1	
EHHADH	4	2	2	
EIF2AK2	4	2	2	
EIF3A	4	2	2	
ELAVL1	4	2	2	
ELF2	4	3	1	
ELF3	4	2	2	
ELK4	4	3	1	
EMG1	4	2	2	
ENGASE	4	3	1	
ENO3	4	2	2	
ENOX2	4	1	3	
ERCC2	4	1	3	

ERCC5	4	1	3	
ERI2	4	1	3	
ETAA1	4	1	3	
ETS2	4	1	3	
ETV4	4	2	2	
EXOG	4	3	1	
FAIM	4	2	2	
FAM102A	4	1	3	
FAM110B	4	2	2	
FAM168B	4	3	1	
FAM171A1	4	2	2	
FAM179B	4	2	2	
FAM46C	4	2	2	
FAM55C	4	2	2	
FASTKD1	4	1	3	
FAU	4	2	2	
FBXL12	4	3	1	
FEM1B	4	1	3	
FKBP11	4	2	2	
FLJ10038	4	2	2	
FLRT3	4	2	2	
FLT3	4	2	2	
FNDC3B	4	1	3	
FOSL1	4	3	1	
FRAT2	4	3	1	
FRYL	4	2	2	
FUBP1	4	1	3	
FUT3	4	2	2	
GAA	4	3	1	
GADD45G	4	2	2	
GALE	4	2	2	
GART	4	2	2	
GATAD1	4	2	2	
GCLM	4	3	1	
GLUD2	4	2	2	
GMEB2	4	2	2	
GNA11	4	2	2	
GNG12	4	2	2	
GNMT	4	3	1	
GRB10	4	1	3	
GSN	4	1	3	
GSTM1	4	2	2	
H1FX	4	2	2	
H2AFY2	4	2	2	
HDGF	4	2	2	
HEATR6	4	1	3	
HEG1	4	3	1	
HERC2	4	2	2	

HERPUD1	4	2	2	
HGSNAT	4	2	2	
HIST1H1E	4	2	2	
HIST1H2AM	4	3	1	
HIST1H2BE	4	3	1	
HIST1H2BF	4	2	2	
HIST1H4E	4	3	1	
HIST1H4J	4	3	1	
HIST2H2AA3	4	3	1	
HJURP	4	3	1	
HK1	4	3	1	
HLA-E	4	2	2	
HMGCR	4	2	2	
HN1L	4	2	2	
HNRNPA0	4	3	1	
HNRNPA3	4	1	3	
HPN	4	3	1	
HSDL2	4	2	2	
HSF2	4	3	1	
ID3	4	3	1	
IER3	4	2	2	
IER5	4	2	2	
IFIH1	4	2	2	
IFIT1	4	3	1	
IFIT3	4	2	2	
IFIT5	4	2	2	
IFT46	4	1	3	
IFT88	4	2	2	
IGFLR1	4	2	2	
IKBKAP	4	1	3	
IL11	4	3	1	
IL1R1	4	2	2	
IL24	4	2	2	
ILF2	4	2	2	
IMPACT	4	1	3	
INHBB	4	1	3	
INTS3	4	2	2	
IPPK	4	2	2	
IRAK1	4	2	2	
IRGQ	4	3	1	
ISL1	4	2	2	
IST1	4	3	1	
ITGA5	4	1	3	
ITGAV	4	1	3	
ITM2B	4	2	2	
ITPR2	4	2	2	
JAK2	4	2	2	
KAZALD1	4	2	2	

KBTBD2	4	2	2	
KCNIP1	4	2	2	
KCNN4	4	2	2	
KCTD9	4	2	2	
KDELR3	4	1	3	
KIAA0182	4	2	2	
KIAA0430	4	2	2	
KIAA0485	4	3	1	
KIAA0664L3	4	2	2	
KIAA1024	4	2	2	
KIAA1324	4	2	2	
KIF13B	4	2	2	
KIF1B	4	2	2	
KLHL9	4	2	2	
KLK7	4	3	1	
KPNA1	4	2	2	
KPNA6	4	2	2	
KRCC1	4	1	3	
KRT19	4	2	2	
KRT3	4	2	2	
KRT8	4	3	1	
KRT8P12	4	2	2	
LAD1	4	2	2	
LANCL1	4	2	2	
LGALS1	4	2	2	
LOC399491	4	2	2	
LOC81691	4	1	3	
LPCAT1	4	2	2	
LRCH4	4	2	2	
LRIG1	4	2	2	
LRP6	4	3	1	
LRRC41	4	3	1	
LRRC8D	4	2	2	
LRRC8E	4	3	1	
LSM14A	4	1	3	
M6PR	4	2	2	
MAGOHB	4	2	2	
MAP1S	4	2	2	
MAP2K5	4	2	2	
MAP3K5	4	1	3	
MAP3K7	4	1	3	
MAP7D1	4	2	2	
MAPK9	4	2	2	
MARCH8	4	2	2	
MAST4	4	0	4	
MAX	4	1	3	
MCL1	4	1	3	
MCM9	4	2	2	

MCMBP	4	2	2	
MED13	4	2	2	
MED13L	4	2	2	
MED4	4	2	2	
MEIS1	4	2	2	
MEPCE	4	1	3	
MET	4	3	1	
METTL9	4	2	2	
MEX3C	4	2	2	
MFAP1	4	2	2	
MICAL2	4	2	2	
MIS12	4	2	2	
MKRN1	4	2	2	
MLEC	4	2	2	
MLF1	4	1	3	
MLPH	4	1	3	
MMADHC	4	2	2	
MORC2	4	1	3	
MREG	4	1	3	
MRPL15	4	1	3	
MRPL16	4	1	3	
MRPL28	4	2	2	
MRPS27	4	2	2	
MRS2	4	1	3	
MRTO4	4	2	2	
MSRB2	4	2	2	
MT1H	4	2	2	
MT1P2	4	2	2	
MTIF2	4	2	2	
MTMR6	4	1	3	
MUTYH	4	1	3	
MXD1	4	3	1	
MYBL1	4	2	2	
NAP1L2	4	2	2	
NCAPG	4	3	1	
NCKAP1	4	2	2	
NCOA1	4	1	3	
NEBL	4	2	2	
NEDD9	4	2	2	
NEMF	4	2	2	
NFAT5	4	2	2	
NFE2L1	4	2	2	
NFE2L2	4	2	2	
NFIC	4	2	2	
NFX1	4	2	2	
NFYA	4	2	2	
NINJ1	4	2	2	
NKX2-2	4	2	2	

NME1	4	2	2	
NME7	4	2	2	
NR1D2	4	2	2	
NR2C2	4	3	1	
NR4A1	4	3	1	
NR4A2	4	2	2	
NSMAF	4	1	3	
NSUN5	4	1	3	
NTAN1	4	1	3	
NTHL1	4	1	3	
NXT1	4	2	2	
OAZ2	4	1	3	
OBFC1	4	1	3	
ODF2	4	2	2	
OGFR	4	2	2	
OPHN1	4	2	2	
OSBPL1A	4	1	3	
OTUD4	4	2	2	
OXSM	4	2	2	
OXTR	4	1	3	
PAAF1	4	1	3	
PALLD	4	2	2	
PAPOLA	4	2	2	
PAQR3	4	1	3	
PARP2	4	0	4	
PARP4	4	1	3	
PATZ1	4	3	1	
PCGF3	4	2	2	
PCIF1	4	3	1	
PCMTD2	4	1	3	
PCP4	4	2	2	
PCTP	4	2	2	
PDCD5	4	1	3	
PDCD6	4	2	2	
PDE3B	4	2	2	
PDHA1	4	1	3	
PDK4	4	2	2	
PDLIM2	4	3	1	
PDLIM5	4	3	1	
PDP1	4	2	2	
PER2	4	2	2	
PES1	4	2	2	
PFKM	4	2	2	
PGM1	4	3	1	
PGRMC1	4	2	2	
PHB	4	2	2	
PHF10	4	1	3	
PHTF1	4	2	2	

PID1	4	2	2	
PIGA	4	2	2	
PIK3R1	4	1	3	
PIK3R3	4	3	1	
PIK3R4	4	1	3	
PIKFYVE	4	1	3	
PIP	4	2	2	
PLAUR	4	1	3	
PLEKHB1	4	1	3	
PLK1	4	1	3	
PLK3	4	2	2	
PLXNA3	4	2	2	
PNLIP	4	2	2	
PNMA1	4	2	2	
PNPO	4	2	2	
POLG2	4	1	3	
POLR2I	4	2	2	
POPDC3	4	2	2	
POT1	4	1	3	
PP14571	4	2	2	
PPAP2B	4	2	2	
PPAT	4	3	1	
PPL	4	2	2	
PPM1A	4	2	2	
PPM1E	4	3	1	
PPP1R10	4	1	3	
PPP1R12A	4	2	2	
PPP2R5C	4	3	1	
PPP3CC	4	2	2	
PPP6R1	4	1	3	
PPWD1	4	2	2	
PRCP	4	2	2	
PRDM10	4	3	1	
PROCR	4	3	1	
PRPF4B	4	2	2	
PRPF6	4	3	1	
PRPS1	4	3	1	
PRRG1	4	2	2	
PSMD7	4	1	3	
PSME4	4	1	3	
PSPH	4	1	3	
PTBP2	4	2	2	
PTCD3	4	2	2	
PTCRA	4	2	2	
PTMA	4	2	2	
PTPN11	4	2	2	
PTPN12	4	3	1	
PTPN9	4	1	3	

PTX3	4	2	2	
PUM2	4	2	2	
PUS7	4	2	2	
PWP1	4	2	2	
PWP2	4	1	3	
PYROXD1	4	1	3	
QRICH1	4	2	2	
RAB14	4	3	1	
RAB20	4	2	2	
RAB40B	4	2	2	
RAB5C	4	2	2	
RAD23A	4	2	2	
RAD51D	4	3	1	
RAD9A	4	2	2	
RALGAPB	4	1	3	
RAMP3	4	2	2	
RASL11B	4	2	2	
RBL2	4	1	3	
RBM12	4	1	3	
RBM4	4	2	2	
RBM5	4	2	2	
RCHY1	4	3	1	
RCOR1	4	3	1	
RCOR3	4	2	2	
REEP1	4	3	1	
REG1B	4	2	2	
REPS2	4	2	2	
RETSAT	4	2	2	
RIBC2	4	3	1	
RIOK2	4	1	3	
RNF111	4	3	1	
RNF113A	4	1	3	
RNF138	4	2	2	
RNF14	4	1	3	
RNF24	4	3	1	
RNFT2	4	1	3	
ROD1	4	2	2	
RPL13	4	2	2	
RPP14	4	3	1	
RRAGD	4	2	2	
RRP1	4	1	3	
RRP7A	4	2	2	
RTF1	4	1	3	
RUFY2	4	2	2	
RWDD3	4	1	3	
SAMM50	4	2	2	
SAR1A	4	2	2	
SCD5	4	2	2	

SCNN1A	4	3	1	
SDHAF1	4	2	2	
SEC22A	4	2	2	
SEC61A2	4	2	2	
SEC62	4	2	2	
SENP2	4	2	2	
SENP6	4	2	2	
SERPINB1	4	2	2	
SETD1B	4	1	3	
SETD2	4	3	1	
SETD5	4	2	2	
SETD6	4	1	3	
SFXN3	4	2	2	
SGPP1	4	2	2	
SH3BGRL	4	2	2	
SH3D21	4	2	2	
SHOC2	4	2	2	
SIRT7	4	2	2	
SLBP	4	2	2	
SLC14A1	4	2	2	
SLC15A2	4	4	0	
SLC33A1	4	1	3	
SLC35E1	4	2	2	
SLC35E3	4	2	2	
SLC4A2	4	2	2	
SLC4A7	4	3	1	
SLC6A14	4	2	2	
SLC6A6	4	2	2	
SLCO3A1	4	2	2	
SLCO4A1	4	3	1	
SMAD1	4	2	2	
SMYD3	4	2	2	
SNN	4	1	3	
SNRPB2	4	2	2	
SORL1	4	2	2	
SOX4	4	1	3	
SP2	4	3	1	
SPAG16	4	2	2	
SPAST	4	2	2	
SPATA2L	4	2	2	
SPATA6	4	2	2	
SRSF5	4	3	1	
SRSF6	4	1	3	
ST20	4	2	2	
STAG2	4	2	2	
STAP2	4	2	2	
STARD7	4	1	3	
STEAP1	4	2	2	

STK19	4	2	2	
STK3	4	4	0	
STX18	4	1	3	
SUGP2	4	2	2	
SULT1A2	4	2	2	
SURF1	4	1	3	
SYK	4	2	2	
SYNJ2	4	3	1	
TAC1	4	2	2	
TAF4B	4	3	1	
TAF5	4	1	3	
TAGLN	4	2	2	
TAOK2	4	2	2	
TBC1D5	4	2	2	
TBCC	4	2	2	
TBL1X	4	2	2	
TBL1XR1	4	1	3	
TCEA2	4	1	3	
TCEB1	4	2	2	
TCERG1	4	2	2	
TCF25	4	3	1	
TCHH	4	3	1	
TDP2	4	1	3	
TEAD1	4	2	2	
TERF1	4	1	3	
TERF2IP	4	2	2	
TFDP1	4	2	2	
THAP1	4	2	2	
THBD	4	1	3	
TIA1	4	1	3	
TIAL1	4	2	2	
TIMP3	4	2	2	
TIPARP	4	2	2	
TJAP1	4	2	2	
TMBIM4	4	2	2	
TMEM100	4	2	2	
TMEM185B	4	2	2	
TMEM194A	4	2	2	
TMEM222	4	3	1	
TMEM30A	4	2	2	
TMEM50B	4	2	2	
TMF1	4	3	1	
TMPRSS11D	4	2	2	
TMSB10	4	2	2	
TMX1	4	3	1	
TNFAIP2	4	3	1	
TOR1AIP1	4	3	1	
TP53I3	4	1	3	

TPP2	4	1	3	
TPRKB	4	2	2	
TRAF4	4	2	2	
TRAF5	4	1	3	
TRAPPC8	4	2	2	
TRIM13	4	4	0	
TRIM27	4	1	3	
TROVE2	4	2	2	
TSC22D1	4	2	2	
TSN	4	1	3	
TTC4	4	2	2	
TUBB	4	2	2	
TUBGCP3	4	1	3	
TUSC3	4	1	3	
TXNL4B	4	2	2	
U2AF1	4	2	2	
UBAP2L	4	3	1	
UBE2H	4	2	2	
UBE2M	4	2	2	
UBR2	4	3	1	
UBXN4	4	2	2	
UFL1	4	2	2	
UQCRFS1	4	2	2	
USP46	4	2	2	
USP47	4	3	1	
USP9X	4	2	2	
UTP14A	4	2	2	
VAMP4	4	2	2	
VAV1	4	3	1	
VAV3	4	1	3	
VCL	4	3	1	
VIP	4	2	2	
VPS33B	4	2	2	
WAC	4	2	2	
WDR37	4	1	3	
WDR47	4	2	2	
WDR5B	4	1	3	
WDR70	4	2	2	
WDR77	4	2	2	
WDTC1	4	2	2	
WISP2	4	3	1	
WRB	4	1	3	
WSB2	4	1	3	
YAF2	4	2	2	
YIPF6	4	2	2	
YTHDC1	4	2	2	
ZBED1	4	2	2	
ZBTB10	4	1	3	

ZBTB43	4	1	3	
ZEB2	4	2	2	
ZFAND1	4	2	2	
ZFX	4	3	1	
ZKSCAN4	4	2	2	
ZNF146	4	2	2	
ZNF165	4	2	2	
ZNF189	4	1	3	
ZNF222	4	2	2	
ZNF23	4	2	2	
ZNF302	4	2	2	
ZNF318	4	2	2	
ZNF337	4	1	3	
ZNF384	4	2	2	
ZNF419	4	3	1	
ZNF473	4	1	3	
ZNF512B	4	2	2	
ZNF529	4	2	2	
ZNF551	4	2	2	
ZNF586	4	2	2	
ZNF646	4	2	2	
ZNF673	4	2	2	
ZNF7	4	1	3	
ZNF783	4	3	1	
ZNF787	4	2	2	
AADAC	3	1	2	
AAGAB	3	1	2	
AARS	3	0	3	
AASS	3	1	2	
AATF	3	2	1	
ABAT	3	2	1	
ABCB8	3	2	1	
ABCF2	3	1	2	
ABCF3	3	1	2	
ABHD11	3	2	1	
ABHD5	3	1	2	
ABLIM1	3	0	3	
ABT1	3	1	2	
ACADL	3	2	1	
ACADS	3	2	1	
ACAP2	3	1	2	
ACAT1	3	1	2	
ACBD3	3	1	2	
ACIN1	3	2	1	
ACOT7	3	2	1	
ACOT8	3	2	1	
ACOT9	3	1	2	
ACOX1	3	2	1	

ACOX3	3	2	1	
ACP1	3	1	2	
ACP2	3	1	2	
ACP5	3	2	1	
ACSBG1	3	1	2	
ACSL4	3	2	1	
ACSL5	3	2	1	
ACSS3	3	1	2	
ACTA1	3	2	1	
ACTN4	3	2	1	
ACTR2	3	2	1	
ACVR1	3	1	2	
ACVR2A	3	2	1	
ADAM10	3	1	2	
ADAM8	3	2	1	
ADAMTS13	3	2	1	
ADCK3	3	1	2	
ADCY6	3	2	1	
ADCY7	3	1	2	
ADCY9	3	2	1	
ADH5	3	0	3	
ADIPOR1	3	1	2	
ADIPOR2	3	1	2	
ADM	3	2	1	
ADRA2C	3	1	2	
ADSL	3	1	2	
AEBP1	3	2	1	
AFTPH	3	2	1	
AGER	3	2	1	
AGGF1	3	1	2	
AGK	3	1	2	
AGPAT3	3	2	1	
AGT	3	2	1	
AGXT2L1	3	1	2	
AHCTF1	3	1	2	
AHCY	3	2	1	
AHCYL2	3	3	0	
AHI1	3	2	1	
AHSA1	3	0	3	
AHSG	3	2	1	
AIFM1	3	1	2	
AIM2	3	2	1	
AKAP10	3	2	1	
AKAP12	3	2	1	
AKAP13	3	3	0	
AKAP9	3	3	0	
AKIRIN1	3	1	2	
AKR1B1	3	1	2	

AKR1B10	3	2	1	
AKTIP	3	1	2	
ALAS1	3	1	2	
ALCAM	3	2	1	
ALDH3A2	3	2	1	
ALDH4A1	3	2	1	
ALDH6A1	3	1	2	
ALG12	3	2	1	
ALG13	3	1	2	
ALG3	3	1	2	
ALKBH1	3	2	1	
ALMS1	3	0	3	
ALOX5AP	3	2	1	
ALPK3	3	2	1	
ALS2CL	3	1	2	
AMD1	3	2	1	
AMOTL2	3	1	2	
ANAPC10	3	2	1	
ANGEL2	3	2	1	
ANKHD1	3	1	2	
ANKMY2	3	2	1	
ANKRA2	3	2	1	
ANKRD10	3	2	1	
ANKRD17	3	2	1	
ANKRD26	3	2	1	
ANKRD28	3	2	1	
ANKRD36	3	2	1	
ANKRD57	3	1	2	
ANKRD6	3	2	1	
ANO3	3	2	1	
ANXA1	3	1	2	
ANXA2	3	2	1	
ANXA2P2	3	1	2	
AOX1	3	1	2	
AP1AR	3	2	1	
AP3M2	3	1	2	
AP3S1	3	2	1	
APAF1	3	2	1	
APBA2	3	1	2	
APEH	3	2	1	
APEX2	3	2	1	
APH1B	3	1	2	
API5	3	1	2	
APOBEC3B	3	2	1	
APOC1	3	3	0	
APOC2	3	2	1	
APOD	3	2	1	
APOH	3	1	2	

APOL1	3	1	2	
APOL2	3	1	2	
APOLD1	3	1	2	
APOOL	3	1	2	
AQP4	3	2	1	
AQP9	3	1	2	
AQR	3	1	2	
ARAP1	3	2	1	
ARC	3	1	2	
ARF6	3	2	1	
ARFGEF1	3	2	1	
ARFGEF2	3	3	0	
ARGLU1	3	1	2	
ARHGAP17	3	1	2	
ARHGAP19	3	1	2	
ARHGAP35	3	3	0	
ARHGAP4	3	1	2	
ARHGDIA	3	1	2	
ARHGEF12	3	2	1	
ARHGEF2	3	0	3	
ARHGEF26	3	1	2	
ARHGEF3	3	2	1	
ARHGEF7	3	3	0	
ARID1A	3	3	0	
ARID3A	3	3	0	
ARID4A	3	2	1	
ARID5B	3	2	1	
ARIH1	3	2	1	
ARIH2	3	1	2	
ARL1	3	0	3	
ARL2BP	3	2	1	
ARL4C	3	2	1	
ARL6IP4	3	1	2	
ARMC8	3	1	2	
ARNT	3	3	0	
ARNTL	3	1	2	
ARPP21	3	2	1	
ARRB1	3	2	1	
ASAP1-IT1	3	1	2	
ASB1	3	2	1	
ASB6	3	1	2	
ASB9	3	2	1	
ASCC1	3	1	2	
ASCC2	3	2	1	
ASCC3	3	2	1	
ASH1L	3	3	0	
ASH2L	3	1	2	
ASPH	3	2	1	

ASTE1	3	1	2	
ASTN2	3	1	2	
ASXL2	3	2	1	
ATAD2B	3	2	1	
ATAD3A	3	2	1	
ATF2	3	2	1	
ATMIN	3	0	3	
ATP1B1	3	2	1	
ATP2A2	3	1	2	
ATP5G3	3	1	2	
ATP6V0A2	3	2	1	
ATP6V0C	3	2	1	
ATP6V0E2	3	2	1	
ATP6V1A	3	1	2	
ATP6V1C1	3	2	1	
ATP6V1D	3	3	0	
ATP6V1H	3	1	2	
ATP9B	3	2	1	
ATRX	3	3	0	
ATXN1	3	1	2	
ATXN2L	3	2	1	
ATXN3	3	2	1	
ATXN7L3B	3	2	1	
AURKB	3	3	0	
AVL9	3	1	2	
B2M	3	1	2	
B3GAT1	3	2	1	
B3GNT3	3	1	2	
B4GALT1	3	2	1	
B4GALT2	3	2	1	
BACH2	3	2	1	
BAG2	3	2	1	
BAG3	3	1	2	
BAIAP2	3	3	0	
BAMBI	3	2	1	
BANK1	3	2	1	
BANP	3	2	1	
BAP1	3	1	2	
BASP1	3	2	1	
BATF3	3	2	1	
BAZ2A	3	3	0	
BAZ2B	3	3	0	
BBC3	3	1	2	
BBS10	3	1	2	
BBS4	3	2	1	
BCAM	3	2	1	
BCKDHA	3	2	1	
BCL10	3	2	1	

BCL11B	3	2	1	
BCL2	3	2	1	
BCL2L13	3	2	1	
BCORL1	3	2	1	
BCR	3	2	1	
BCS1L	3	1	2	
BDKRB2	3	1	2	
BFAR	3	1	2	
BICD1	3	2	1	
BICD2	3	1	2	
BID	3	2	1	
BIK	3	2	1	
BIN1	3	3	0	
BIN2	3	2	1	
BLM	3	1	2	
BLNK	3	2	1	
BNIP2	3	2	1	
BNIP3	3	1	2	
BPGM	3	0	3	
BPTF	3	3	0	
BRAP	3	2	1	
BRD4	3	1	2	
BRD7	3	1	2	
BRIP1	3	2	1	
BRIX1	3	2	1	
BRMS1	3	2	1	
BRWD1	3	1	2	
BSPRY	3	1	2	
BTAF1	3	1	2	
BTBD3	3	2	1	
BTG3	3	1	2	
BTN2A1	3	2	1	
BTN3A3	3	2	1	
BUB1	3	3	0	
BUB3	3	1	2	
BZW1	3	3	0	
C10ORF26	3	1	2	
C10ORF95	3	1	2	
C11ORF16	3	1	2	
C11ORF61	3	2	1	
C11ORF75	3	2	1	
C12ORF11	3	2	1	
C12ORF51	3	1	2	
C12ORF52	3	1	2	
C14ORF101	3	1	2	
C14ORF133	3	1	2	
C16ORF61	3	1	2	
C16ORF7	3	1	2	

C17ORF108	3	1	2	
C17ORF48	3	1	2	
C19ORF2	3	1	2	
C19ORF21	3	2	1	
C19ORF24	3	2	1	
C1GALT1	3	2	1	
C1ORF174	3	2	1	
C1ORF183	3	1	2	
C1ORF38	3	2	1	
C1ORF54	3	1	2	
C1ORF9	3	1	2	
C20ORF103	3	1	2	
C20ORF11	3	2	1	
C21ORF91	3	2	1	
C22ORF29	3	2	1	
C2CD3	3	1	2	
C2ORF18	3	1	2	
C2ORF28	3	2	1	
C2ORF47	3	2	1	
C3ORF14	3	1	2	
C3ORF18	3	2	1	
C4ORF27	3	1	2	
C4ORF34	3	1	2	
C5	3	2	1	
C6ORF120	3	1	2	
C6ORF145	3	1	2	
C6ORF47	3	1	2	
C6ORF48	3	0	3	
C7ORF42	3	2	1	
C7ORF44	3	2	1	
C7ORF68	3	2	1	
C9ORF167	3	2	1	
C9ORF91	3	1	2	
CA2	3	1	2	
CAB39	3	1	2	
CALM1	3	3	0	
CAMKMT	3	2	1	
CAMP	3	1	2	
CAMSAP1	3	2	1	
CAND2	3	2	1	
CANX	3	2	1	
CAP2	3	2	1	
CAPN9	3	3	0	
CARD10	3	2	1	
CARHSP1	3	2	1	
CASP7	3	2	1	
CASP8	3	1	2	
CASP9	3	2	1	

CAV1	3	1	2	
CBFA2T2	3	2	1	
CBFB	3	1	2	
CBLB	3	2	1	
CBY1	3	1	2	
CC2D1A	3	3	0	
CCDC15	3	1	2	
CCDC22	3	2	1	
CCDC28A	3	1	2	
CCDC85C	3	1	2	
CCDC88C	3	2	1	
CCDC90B	3	1	2	
CCDC93	3	2	1	
CCK	3	1	2	
CCL5	3	2	1	
CCNC	3	1	2	
CCNF	3	1	2	
CCNJ	3	1	2	
CCT2	3	1	2	
CCT5	3	2	1	
CD14	3	2	1	
CD164	3	2	1	
CD207	3	2	1	
CD38	3	3	0	
CD3D	3	1	2	
CD44	3	1	2	
CD47	3	3	0	
CD52	3	1	2	
CD59	3	1	2	
CD7	3	2	1	
CD80	3	1	2	
CDA	3	2	1	
CDC37	3	1	2	
CDC40	3	2	1	
CDC42EP1	3	1	2	
CDC42EP4	3	1	2	
CDH2	3	2	1	
CDH4	3	2	1	
CDK10	3	2	1	
CDK12	3	3	0	
CDK2	3	1	2	
CDK20	3	1	2	
CDK4	3	1	2	
CDK5RAP2	3	2	1	
CDK5RAP3	3	1	2	
CDK9	3	2	1	
CDKN1B	3	1	2	
CDKN2C	3	1	2	

CDT1	3	2	1	
CDV3	3	1	2	
CDYL	3	2	1	
CEACAM6	3	1	2	
CEBPD	3	2	1	
CEBPG	3	0	3	
CEP152	3	3	0	
CEP192	3	1	2	
CEP250	3	2	1	
CEP350	3	2	1	
CEP70	3	1	2	
CEP76	3	1	2	
CEPT1	3	1	2	
CER1	3	2	1	
CERK	3	2	1	
CERS6	3	1	2	
CES3	3	1	2	
CETN3	3	1	2	
CGA	3	2	1	
CHD1	3	2	1	
CHD3	3	3	0	
CHD4	3	2	1	
CHEK1	3	2	1	
CHERP	3	2	1	
CHI3L2	3	2	1	
CHL1	3	2	1	
CHMP1A	3	1	2	
CHMP2B	3	1	2	
CHPF2	3	1	2	
CHRNE	3	3	0	
CHST10	3	1	2	
CHTOP	3	2	1	
CIAO1	3	1	2	
CIB1	3	2	1	
CINP	3	2	1	
CIR1	3	1	2	
CISH	3	2	1	
CIT	3	2	1	
CKAP5	3	1	2	
CKB	3	2	1	
CKS1B	3	2	1	
CKS2	3	2	1	
CLASP2	3	2	1	
CLCF1	3	2	1	
CLDN1	3	2	1	
CLDN4	3	1	2	
CLDN7	3	2	1	
CLDN8	3	2	1	

CLEC2D	3	2	1	
CLIC3	3	1	2	
CLIP1	3	2	1	
CLNS1A	3	2	1	
CLP1	3	1	2	
CLU	3	1	2	
CMAS	3	1	2	
CNNM3	3	2	1	
CNOT6	3	2	1	
CNOT8	3	2	1	
CNPY2	3	1	2	
CNPY4	3	1	2	
CNTN1	3	2	1	
CNTNAP2	3	2	1	
COCH	3	2	1	
COG4	3	1	2	
COG5	3	1	2	
COL11A1	3	2	1	
COL17A1	3	1	2	
COL4A3BP	3	1	2	
COL5A2	3	1	2	
COL7A1	3	1	2	
COMMD3	3	1	2	
COPB2	3	1	2	
COPG	3	1	2	
COPS6	3	2	1	
COPS8	3	1	2	
COQ10B	3	1	2	
COQ4	3	2	1	
CORO2A	3	2	1	
COX16	3	2	1	
COX4I1	3	1	2	
CPA2	3	2	1	
CPD	3	1	2	
CPEB3	3	2	1	
CPNE1	3	2	1	
CPNE3	3	2	1	
CPS1	3	1	2	
CPSF1	3	2	1	
CPSF4	3	0	3	
CPT2	3	0	3	
CREB3	3	1	2	
CREBZF	3	2	1	
CRELD2	3	3	0	
CRKL	3	3	0	
CRNN	3	2	1	
CROCCP2	3	2	1	
CRP	3	2	1	

CRTAM	3	2	1	
CRTAP	3	1	2	
CRY2	3	2	1	
CRYL1	3	1	2	
CSDA	3	2	1	
CSDE1	3	2	1	
CSK	3	0	3	
CSNK1D	3	2	1	
CSNK1G2	3	2	1	
CSNK1G3	3	2	1	
CSNK2A2	3	1	2	
CSPG5	3	2	1	
CSPP1	3	2	1	
CST3	3	1	2	
CSTF1	3	2	1	
CTBP1	3	1	2	
CTDP1	3	2	1	
CTDSP2	3	2	1	
CTNNA2	3	2	1	
CTNND2	3	2	1	
CTSE	3	1	2	
CTSK	3	2	1	
CUL4A	3	1	2	
CUL5	3	1	2	
CUX1	3	2	1	
CWC25	3	2	1	
CWF19L1	3	2	1	
CXADR	3	1	2	
CXCL3	3	1	2	
CYB5R2	3	1	2	
CYBA	3	2	1	
CYBRD1	3	1	2	
CYP1B1	3	1	2	
CYP24A1	3	2	1	
CYP27B1	3	2	1	
CYP2B6	3	3	0	
CYP2C19	3	3	0	
CYP2E1	3	3	0	
CYP3A4	3	3	0	
CYP4F2	3	2	1	
CYP51A1	3	1	2	
D4S234E	3	2	1	
DACH1	3	2	1	
DAG1	3	1	2	
DAK	3	2	1	
DARS	3	1	2	
DCAF10	3	1	2	
DCAF8	3	2	1	

DCBLD2	3	2	1	
DCLRE1C	3	1	2	
DCTN4	3	1	2	
DCUN1D1	3	1	2	
DCUN1D4	3	2	1	
DDAH2	3	1	2	
DDOST	3	2	1	
DDRGK1	3	1	2	
DDX24	3	1	2	
DDX28	3	2	1	
DDX31	3	1	2	
DDX3X	3	3	0	
DDX49	3	2	1	
DDX54	3	1	2	
DDX58	3	1	2	
DEAF1	3	1	2	
DEGS1	3	3	0	
DENND4A	3	2	1	
DENND4B	3	1	2	
DENND4C	3	1	2	
DENND5A	3	1	2	
DENND5B	3	1	2	
DERA	3	2	1	
DERL1	3	1	2	
DEXI	3	2	1	
DFFA	3	2	1	
DFFB	3	0	3	
DHRS1	3	2	1	
DHRS7B	3	1	2	
DIAPH1	3	2	1	
DIS3	3	1	2	
DLC1	3	2	1	
DLD	3	2	1	
DLG4	3	1	2	
DLGAP4	3	2	1	
DLGAP5	3	2	1	
DLX2	3	2	1	
DMBT1	3	1	2	
DMP1	3	1	2	
DMXL1	3	1	2	
DNAAF2	3	1	2	
DNAJB14	3	2	1	
DNAJB6	3	2	1	
DNAJB9	3	1	2	
DNAJC10	3	1	2	
DNAJC12	3	1	2	
DNAJC13	3	2	1	
DNAJC15	3	1	2	

DNAJC24	3	0	3	
DNAJC3	3	2	1	
DNAJC7	3	1	2	
DNAJC8	3	1	2	
DNASE1L2	3	1	2	
DNASE1L3	3	1	2	
DND1	3	1	2	
DNMBP	3	1	2	
DNMT3B	3	1	2	
DOK1	3	2	1	
DOLPP1	3	2	1	
DPH5	3	1	2	
DPY19L1	3	2	1	
DPYSL2	3	1	2	
DRAM1	3	2	1	
DROSHA	3	1	2	
DSG1	3	1	2	
DST	3	2	1	
DSTN	3	2	1	
DTWD1	3	1	2	
DUSP13	3	2	1	
DUSP7	3	2	1	
DYNLT1	3	1	2	
E2F3	3	2	1	
E2F5	3	2	1	
E2F6	3	1	2	
EBAG9	3	1	2	
EBF2	3	2	1	
EBLN2	3	2	1	
EBP	3	2	1	
ECE1	3	2	1	
ECM2	3	1	2	
ECSIT	3	2	1	
EDEM2	3	1	2	
EDN1	3	3	0	
EDNRA	3	3	0	
EEA1	3	3	0	
EEF1D	3	2	1	
EFCAB2	3	1	2	
EFHD2	3	2	1	
EFNA4	3	2	1	
EFNB2	3	1	2	
EFR3A	3	2	1	
EFTUD1	3	0	3	
EGR2	3	2	1	
EGR3	3	2	1	
EHBP1L1	3	2	1	
EHD4	3	1	2	

EIF1	3	2	1	
EIF1B	3	2	1	
EIF2B2	3	2	1	
EIF2B3	3	0	3	
EIF2C1	3	2	1	
EIF2C2	3	2	1	
EIF2C3	3	2	1	
EIF2S1	3	2	1	
EIF3D	3	2	1	
EIF3L	3	2	1	
EIF3M	3	2	1	
EIF4A2	3	1	2	
EIF4A3	3	2	1	
EIF4EBP2	3	1	2	
EIF4ENIF1	3	2	1	
EIF4H	3	1	2	
ELAVL2	3	2	1	
ELAVL3	3	2	1	
ELF4	3	2	1	
ELF5	3	2	1	
ELK1	3	3	0	
ELK3	3	2	1	
ELMO2	3	1	2	
ELOVL4	3	2	1	
ELOVL6	3	2	1	
EMP2	3	2	1	
ENPEP	3	2	1	
ENPP1	3	2	1	
ENTPD2	3	2	1	
ENTPD3	3	2	1	
ENTPD6	3	2	1	
EPB49	3	1	2	
EPHA4	3	3	0	
EPHB3	3	2	1	
EPHB6	3	1	2	
EPHX3	3	2	1	
EPPK1	3	1	2	
EPS15	3	1	2	
ERC1	3	3	0	
ERCC3	3	1	2	
ERF	3	2	1	
ERGIC2	3	0	3	
ERH	3	2	1	
ERLIN1	3	1	2	
ERP44	3	1	2	
ESF1	3	2	1	
ETNK2	3	1	2	
ETV1	3	2	1	

EVL	3	1	2	
EXOC7	3	3	0	
EXOSC4	3	3	0	
EXOSC8	3	0	3	
EZR	3	2	1	
F11R	3	2	1	
F3	3	2	1	
F7	3	3	0	
FAAH	3	2	1	
FABP1	3	2	1	
FABP5	3	2	1	
FADD	3	1	2	
FAF2	3	1	2	
FAHD2A	3	1	2	
FAM107A	3	1	2	
FAM114A1	3	1	2	
FAM114A2	3	1	2	
FAM115A	3	2	1	
FAM117A	3	1	2	
FAM134B	3	1	2	
FAM158A	3	2	1	
FAM164A	3	1	2	
FAM168A	3	2	1	
FAM169A	3	2	1	
FAM172A	3	1	2	
FAM175B	3	1	2	
FAM178A	3	2	1	
FAM184A	3	1	2	
FAM189B	3	2	1	
FAM192A	3	1	2	
FAM193A	3	2	1	
FAM20B	3	2	1	
FAM30A	3	2	1	
FAM3A	3	1	2	
FAM3C	3	2	1	
FAM50B	3	1	2	
FAM57A	3	2	1	
FAM65A	3	1	2	
FAM70A	3	1	2	
FAM86C1	3	1	2	
FAM89B	3	2	1	
FANCI	3	1	2	
FARP2	3	2	1	
FARSA	3	1	2	
FASTKD2	3	2	1	
FBLN5	3	2	1	
FBN1	3	2	1	
FBP1	3	1	2	

FBRS	3	2	1	
FBXL4	3	1	2	
FBXL5	3	2	1	
FBXO2	3	1	2	
FBXO38	3	2	1	
FBXW11	3	2	1	
FBXW4	3	2	1	
FCER1A	3	1	2	
FCGR2A	3	2	1	
FECH	3	2	1	
FEZ1	3	2	1	
FGF1	3	1	2	
FGFR1OP	3	3	0	
FGFR2	3	2	1	
FICD	3	1	2	
FIP1L1	3	2	1	
FKBP14	3	1	2	
FKBP15	3	2	1	
FKSG49	3	1	2	
FLJ11710	3	2	1	
FLNC	3	2	1	
FLOT1	3	2	1	
FMNL1	3	2	1	
FMO5	3	1	2	
FN3KRP	3	2	1	
FNBP1	3	1	2	
FNBP1L	3	2	1	
FNBP4	3	1	2	
FNDC3A	3	1	2	
FNDC4	3	2	1	
FNTA	3	1	2	
FOLH1	3	2	1	
FOS	3	2	1	
FOXC1	3	2	1	
FOXF1	3	1	2	
FOXG1	3	2	1	
FOXJ1	3	2	1	
FOXM1	3	2	1	
FOXN3	3	1	2	
FPGS	3	1	2	
FPR1	3	2	1	
FSTL1	3	2	1	
FSTL3	3	3	0	
FTSJ2	3	1	2	
FTSJ3	3	1	2	
FUCA1	3	1	2	
FUT1	3	3	0	
FUT4	3	1	2	

FXC1	3	1	2	
FXN	3	1	2	
FXR1	3	1	2	
FXYD1	3	2	1	
FXYD3	3	1	2	
FXYD7	3	1	2	
FYB	3	1	2	
FYCO1	3	2	1	
FZD3	3	2	1	
FZD5	3	2	1	
FZD6	3	1	2	
FZD8	3	2	1	
GAB2	3	2	1	
GABARAPL3	3	3	0	
GABPB1	3	1	2	
GABRA2	3	2	1	
GADD45B	3	2	1	
GAL	3	3	0	
GALNS	3	1	2	
GALNT11	3	2	1	
GALNT14	3	1	2	
GANAB	3	2	1	
GAP43	3	1	2	
GAPDH	3	1	2	
GAPDHS	3	2	1	
GAPVD1	3	2	1	
GAS2	3	2	1	
GAS7	3	1	2	
GATA2	3	2	1	
GATA4	3	1	2	
GATA6	3	3	0	
GATAD2A	3	2	1	
GBE1	3	2	1	
GCA	3	2	1	
GDF15	3	2	1	
GDF2	3	2	1	
GDI2	3	2	1	
GEMIN4	3	1	2	
GFAP	3	1	2	
GFRA2	3	2	1	
GGCX	3	1	2	
GGH	3	3	0	
GGPS1	3	1	2	
GIGYF2	3	2	1	
GIMAP6	3	2	1	
GIN1	3	1	2	
GJA1	3	1	2	
GLCE	3	2	1	

GLRB	3	2	1	
GLRX2	3	0	3	
GLRX3	3	2	1	
GLRX5	3	1	2	
GLT8D1	3	0	3	
GLTP	3	1	2	
GLTSCR1	3	2	1	
GLUL	3	2	1	
GMCL1	3	1	2	
GNA12	3	1	2	
GNAI1	3	1	2	
GNAQ	3	1	2	
GNB2L1	3	1	2	
GNG7	3	1	2	
GNL3L	3	2	1	
GOLGA1	3	2	1	
GOLGA2	3	1	2	
GOLGA3	3	1	2	
GOLGA8A	3	2	1	
GOLGB1	3	1	2	
GOLIM4	3	0	3	
GORASP2	3	0	3	
GOT2	3	1	2	
GP1BB	3	2	1	
GPA33	3	1	2	
GPATCH1	3	2	1	
GPATCH3	3	2	1	
GPATCH8	3	1	2	
GPC1	3	2	1	
GPC4	3	1	2	
GPD1	3	1	2	
GPD2	3	2	1	
GPLD1	3	2	1	
GPN2	3	3	0	
GPR107	3	2	1	
GPR19	3	1	2	
GPR20	3	1	2	
GPRC5A	3	1	2	
GPRC5C	3	2	1	
GPS2	3	1	2	
GPX2	3	2	1	
GRAMD4	3	1	2	
GRB2	3	1	2	
GRB7	3	3	0	
GREB1	3	2	1	
GREM2	3	1	2	
GRHL2	3	2	1	
GRHPR	3	2	1	

GRIA1	3	2	1	
GRINA	3	2	1	
GSDMD	3	1	2	
GSK3B	3	2	1	
GSTCD	3	2	1	
GSTM2	3	2	1	
GSTO1	3	1	2	
GSTP1	3	2	1	
GTF2A1	3	2	1	
GTF2B	3	2	1	
GTF2E1	3	0	3	
GTF2H2B	3	2	1	
GTF3A	3	0	3	
GTF3C1	3	2	1	
GTPBP3	3	1	2	
GTPBP8	3	3	0	
GUCY1A3	3	1	2	
GUSBP3	3	1	2	
GYG2	3	2	1	
H2AFV	3	1	2	
H2AFZ	3	2	1	
H2BFS	3	1	2	
H3F3B	3	1	2	
HABP4	3	2	1	
HADH	3	2	1	
HADHA	3	1	2	
HAL	3	2	1	
HAND1	3	2	1	
HAS1	3	1	2	
HAUS3	3	1	2	
HAUS5	3	1	2	
HAUS6	3	2	1	
HBS1L	3	1	2	
HBZ	3	1	2	
HCCS	3	1	2	
HCP5	3	1	2	
HDDC2	3	1	2	
HDHD1	3	1	2	
HDLBP	3	1	2	
HEBP1	3	2	1	
HECTD3	3	1	2	
HERC1	3	1	2	
HERC6	3	1	2	
HEY2	3	1	2	
HFE	3	3	0	
HGD	3	2	1	
HGS	3	1	2	
HHLA3	3	1	2	

HIC1	3	2	1	
HIC2	3	3	0	
HINFP	3	2	1	
HIPK1	3	2	1	
HIST1H1D	3	2	1	
HIST1H2AI	3	2	1	
HIST1H2BC	3	1	2	
HIST1H2BH	3	2	1	
HIST1H2BI	3	2	1	
HIST1H2BK	3	3	0	
HIST1H3F	3	3	0	
HIST1H4C	3	2	1	
HIST1H4D	3	2	1	
HIST1H4H	3	3	0	
HIST3H2A	3	2	1	
HIVEP2	3	1	2	
HIVEP3	3	2	1	
HK2	3	1	2	
HLA-C	3	1	2	
HLA-DRB1	3	1	2	
HLA-F	3	1	2	
HMBOX1	3	2	1	
HMG20A	3	2	1	
HMGCS1	3	3	0	
HMGN2	3	2	1	
HMGN3	3	2	1	
HMMR	3	1	2	
HMP19	3	1	2	
HNRNPA1	3	1	2	
HNRNPL	3	1	2	
HNRNPR	3	1	2	
HNRPDL	3	2	1	
HOOK1	3	2	1	
HOXA10	3	2	1	
HOXA3	3	2	1	
HOXA7	3	3	0	
HOXB13	3	2	1	
HOXC13	3	2	1	
HP	3	1	2	
HPS5	3	0	3	
HRAS	3	1	2	
HRASLS	3	2	1	
HS2ST1	3	1	2	
HSP90B1	3	1	2	
HSPA13	3	1	2	
HSPA1L	3	2	1	
HSPA2	3	1	2	
HSPA8	3	2	1	

HSPB8	3	2	1	
HSPBAP1	3	1	2	
HTATIP2	3	1	2	
HTR1F	3	2	1	
HUS1	3	3	0	
HUWE1	3	2	1	
HYAL2	3	1	2	
HYI	3	1	2	
ICAM3	3	1	2	
ICAM4	3	1	2	
ICOS	3	1	2	
ICT1	3	2	1	
ID2	3	1	2	
IDE	3	1	2	
IDH1	3	1	2	
IDH2	3	1	2	
IDH3A	3	2	1	
IDH3B	3	2	1	
IDS	3	2	1	
IER3IP1	3	1	2	
IFI44	3	2	1	
IFI6	3	2	1	
IFIT2	3	2	1	
IFITM1	3	2	1	
IFITM3	3	1	2	
IFNAR2	3	1	2	
IFRD1	3	0	3	
IGF2R	3	3	0	
IGFBP5	3	2	1	
IGHMBP2	3	2	1	
IGKC	3	2	1	
IGKV3-20	3	2	1	
IK	3	1	2	
IKBKG	3	1	2	
IKZF5	3	2	1	
IL13RA1	3	2	1	
IL13RA2	3	2	1	
IL15	3	1	2	
IL17RB	3	2	1	
IL1R2	3	2	1	
IL20RA	3	1	2	
IL21R	3	2	1	
IL6	3	1	2	
ILF3	3	2	1	
ILKAP	3	1	2	
ILVBL	3	2	1	
INADL	3	3	0	
INCENP	3	2	1	

INF2	3	2	1	
ING1	3	2	1	
INHBA	3	2	1	
INO80B	3	1	2	
INPP1	3	2	1	
INPP5E	3	1	2	
INPP5F	3	1	2	
INS	3	1	2	
INSR	3	3	0	
INTS12	3	1	2	
IP6K1	3	2	1	
IP6K2	3	2	1	
IPCEF1	3	2	1	
IPO5	3	1	2	
IPO7	3	1	2	
IPP	3	1	2	
IREB2	3	2	1	
IRF2BP1	3	1	2	
IRF5	3	1	2	
IRS2	3	1	2	
IRX4	3	1	2	
ISG15	3	2	1	
ISOC2	3	2	1	
ITCH	3	1	2	
ITFG1	3	2	1	
ITGB6	3	1	2	
ITGB8	3	1	2	
ITGBL1	3	1	2	
ITIH2	3	2	1	
ITPKB	3	1	2	
ITSN1	3	2	1	
JMJD6	3	2	1	
KANK2	3	2	1	
KARS	3	0	3	
KAT8	3	1	2	
KATNB1	3	1	2	
KAZN	3	2	1	
KBTBD4	3	1	2	
KCMF1	3	1	2	
KCNG1	3	1	2	
KCNJ15	3	2	1	
KCNJ8	3	2	1	
KCNK1	3	1	2	
KCNK5	3	1	2	
KCTD3	3	1	2	
KDELC1	3	1	2	
KDELR1	3	2	1	
KDELR2	3	1	2	

KDM5B	3	2	1	
KDSR	3	2	1	
KEAP1	3	1	2	
KHDRBS1	3	1	2	
KHNYN	3	2	1	
KIAA0040	3	1	2	
KIAA0226	3	2	1	
KIAA0247	3	2	1	
KIAA0355	3	2	1	
KIAA0754	3	1	2	
KIAA0894	3	2	1	
KIAA0895	3	2	1	
KIAA1109	3	2	1	
KIDINS220	3	3	0	
KIF16B	3	1	2	
KIF18A	3	2	1	
KIF3A	3	2	1	
KIFAP3	3	1	2	
KIN	3	1	2	
KLC1	3	2	1	
KLF11	3	1	2	
KLF5	3	1	2	
KLHDC10	3	1	2	
KLHDC2	3	1	2	
KLHDC4	3	1	2	
KLHL20	3	2	1	
KLHL3	3	1	2	
KLK2	3	3	0	
KLK3	3	2	1	
KLKB1	3	1	2	
KMO	3	2	1	
KNG1	3	1	2	
KPNA3	3	1	2	
KPNA5	3	2	1	
KRIT1	3	1	2	
KRR1	3	0	3	
KRT14	3	1	2	
KRT16	3	0	3	
KRT17	3	1	2	
KRT34	3	2	1	
KRT5	3	1	2	
KRT6A	3	3	0	
KRT7	3	2	1	
KRT81	3	2	1	
L1CAM	3	2	1	
L1TD1	3	2	1	
LACTB2	3	0	3	
LAGE3	3	1	2	

LAMA5	3	2	1	
LAMB1	3	2	1	
LANCL2	3	1	2	
LARP4B	3	2	1	
LCMT1	3	0	3	
LCMT2	3	2	1	
LDOC1	3	2	1	
LEF1	3	2	1	
LEMD3	3	1	2	
LEPROTL1	3	1	2	
LETM1	3	2	1	
LGALS3	3	1	2	
LGALS8	3	2	1	
LGALS9	3	2	1	
LGR4	3	0	3	
LHFPL2	3	1	2	
LHPP	3	1	2	
LHX2	3	1	2	
LIG4	3	2	1	
LIMA1	3	2	1	
LIMK2	3	2	1	
LIN7A	3	1	2	
LIN7C	3	2	1	
LINC00312	3	1	2	
LINC00342	3	2	1	
LINC00472	3	2	1	
LIPA	3	2	1	
LIPT1	3	1	2	
LMCD1	3	1	2	
LMNA	3	2	1	
LMNB1	3	2	1	
LOC100127972	3	1	2	
LOC100506469	3	2	1	
LOC100508797	3	2	1	
LOC202181	3	2	1	
LOC282997	3	1	2	
LOC283683	3	1	2	
LOC390940	3	2	1	
LOX	3	1	2	
LPHN2	3	2	1	
LPIN2	3	2	1	
LPPR2	3	2	1	
LPPR3	3	2	1	
LRFN4	3	1	2	
LRIF1	3	1	2	
LRP8	3	2	1	
LRRC16A	3	1	2	
LRRC31	3	1	2	

LRRC6	3	2	1	
LRRFIP2	3	2	1	
LRRN3	3	2	1	
LSM2	3	1	2	
LSS	3	3	0	
LTB	3	1	2	
LUC7L	3	2	1	
LUC7L3	3	1	2	
LUZP1	3	1	2	
LY86	3	1	2	
LYL1	3	2	1	
LYPD1	3	2	1	
LYPD3	3	2	1	
LYPLA1	3	2	1	
LYST	3	3	0	
LZTFL1	3	1	2	
LZTR1	3	2	1	
MACF1	3	1	2	
MAD2L1	3	2	1	
MAGEA1	3	1	2	
MAGEA10	3	2	1	
MAGEA3	3	2	1	
MAGEA5	3	1	2	
MAGEA6	3	2	1	
MAGEF1	3	0	3	
MAGT1	3	1	2	
MAK16	3	0	3	
MALT1	3	1	2	
MAML1	3	2	1	
MAN1A1	3	2	1	
MANSC1	3	1	2	
MAP1B	3	1	2	
MAP1LC3B	3	2	1	
MAP2K3	3	1	2	
MAP3K1	3	2	1	
MAP3K4	3	1	2	
MAP3K8	3	2	1	
MAPK8	3	2	1	
MAPRE1	3	2	1	
MAPT	3	2	1	
MARCH6	3	2	1	
MARCKS	3	1	2	
MARK4	3	1	2	
MAST3	3	1	2	
MATK	3	2	1	
MATR3	3	1	2	
MAU2	3	3	0	
MBD1	3	2	1	

MBD5	3	1	2	
MBL2	3	3	0	
MBNL1	3	2	1	
MCOLN1	3	1	2	
MCRS1	3	1	2	
MDC1	3	1	2	
MDM1	3	1	2	
MDM2	3	2	1	
MED1	3	2	1	
MED15	3	1	2	
MED17	3	1	2	
MED18	3	2	1	
MEF2A	3	2	1	
MEG3	3	2	1	
MEGF9	3	1	2	
MEIS3P1	3	1	2	
METAP2	3	0	3	
METTL10	3	2	1	
METTL13	3	1	2	
METTL2B	3	1	2	
METTL3	3	1	2	
METTL4	3	1	2	
METTL5	3	1	2	
METTL7A	3	1	2	
MFAP3	3	3	0	
MFAP3L	3	2	1	
MFF	3	1	2	
MFSD1	3	1	2	
MFSD11	3	1	2	
MFSD6	3	2	1	
MGEA5	3	2	1	
MGLL	3	1	2	
MICALL2	3	1	2	
MICB	3	2	1	
MICU1	3	1	2	
MID1IP1	3	2	1	
MINK1	3	2	1	
MINPP1	3	1	2	
MIR22HG	3	1	2	
MITF	3	2	1	
MKKS	3	1	2	
MLANA	3	2	1	
MLC1	3	2	1	
MLH1	3	1	2	
MLL	3	2	1	
MLLT11	3	2	1	
MLXIPL	3	2	1	
MLYCD	3	1	2	

MMP19	3	2	1	
MMP2	3	2	1	
MMP3	3	1	2	
MMP9	3	1	2	
MNS1	3	2	1	
MON2	3	2	1	
MORC3	3	2	1	
MORC4	3	1	2	
MORF4L1	3	2	1	
MORF4L2	3	2	1	
MPDU1	3	2	1	
MPG	3	2	1	
MPP5	3	2	1	
MPRIP	3	1	2	
MPZ	3	2	1	
MPZL2	3	2	1	
MRC2	3	1	2	
MRE11A	3	2	1	
MRPL17	3	1	2	
MRPL18	3	0	3	
MRPL2	3	1	2	
MRPL24	3	2	1	
MRPL39	3	0	3	
MRPL4	3	2	1	
MRPL52	3	1	2	
MRPS18A	3	1	2	
MRPS18B	3	1	2	
MRPS28	3	2	1	
MRPS30	3	1	2	
MS4A1	3	2	1	
MS4A6A	3	2	1	
MSL2	3	2	1	
MSL3	3	1	2	
MSMO1	3	2	1	
MSN	3	2	1	
MST1	3	1	2	
MST1R	3	1	2	
MSX2	3	3	0	
MT1E	3	1	2	
MTCP1NB	3	1	2	
MTF2	3	3	0	
MTFR1	3	1	2	
MTHFSD	3	1	2	
MTMR11	3	1	2	
MTO1	3	1	2	
MTRR	3	2	1	
MTUS1	3	2	1	
MTX2	3	2	1	

MUC5B	3	2	1	
MUM1	3	1	2	
MUS81	3	0	3	
MUT	3	1	2	
MVD	3	2	1	
MVP	3	2	1	
MX2	3	1	2	
MYB	3	2	1	
MYBBP1A	3	1	2	
MYL6	3	2	1	
MYLPF	3	2	1	
MYO1A	3	2	1	
MYO1C	3	1	2	
MYO5C	3	1	2	
MYO9A	3	1	2	
MYRIP	3	2	1	
MZT2A	3	2	1	
N6AMT1	3	1	2	
NAA10	3	1	2	
NAA16	3	2	1	
NAAA	3	1	2	
NACC2	3	1	2	
NADSYN1	3	2	1	
NAE1	3	1	2	
NANOG	3	2	1	
NAP1L3	3	1	2	
NAP1L4	3	2	1	
NAPG	3	1	2	
NARF	3	1	2	
NARS2	3	2	1	
NAT10	3	2	1	
NAT2	3	1	2	
NBN	3	1	2	
NCAM2	3	2	1	
NCDN	3	2	1	
NCK2	3	1	2	
NCLN	3	3	0	
NCOA3	3	2	1	
NCRNA00185	3	2	1	
NDFIP1	3	1	2	
NDP	3	2	1	
NDRG1	3	2	1	
NDST3	3	2	1	
NDUFA4	3	2	1	
NDUFA8	3	1	2	
NDUFAF4	3	0	3	
NDUFB2	3	2	1	
NDUFS2	3	2	1	

NDUFV2	3	1	2	
NEDD4	3	0	3	
NEK1	3	1	2	
NEK4	3	0	3	
NEK9	3	2	1	
NELL2	3	2	1	
NEO1	3	2	1	
NETO2	3	2	1	
NF2	3	2	1	
NFATC2IP	3	1	2	
NFATC4	3	2	1	
NFIB	3	1	2	
NFKBIB	3	1	2	
NFKBIE	3	2	1	
NFS1	3	1	2	
NFYB	3	2	1	
NFYC	3	2	1	
NGF	3	1	2	
NGFRAP1	3	2	1	
NGLY1	3	2	1	
NHP2	3	2	1	
NID2	3	2	1	
NIF3L1	3	1	2	
NIPA2	3	0	3	
NIPBL	3	3	0	
NIPSNAP1	3	2	1	
NKTR	3	2	1	
NKX3-1	3	3	0	
NLE1	3	1	2	
NLGN4X	3	2	1	
NMT2	3	2	1	
NOC2L	3	2	1	
NOC3L	3	2	1	
NOL6	3	1	2	
NOL8	3	0	3	
NOP56	3	1	2	
NOS2	3	1	2	
NOTCH2	3	2	1	
NOTCH2NL	3	3	0	
NPAT	3	2	1	
NPC1L1	3	1	2	
NPEPPS	3	2	1	
NPIPL3	3	2	1	
NPL	3	2	1	
NQO2	3	2	1	
NR1H3	3	1	2	
NR1I3	3	2	1	
NR2E1	3	2	1	

NRGN	3	1	2	
NRIP1	3	2	1	
NRSN2	3	2	1	
NSDHL	3	2	1	
NSF	3	1	2	
NSMCE4A	3	1	2	
NSUN5P1	3	1	2	
NSUN6	3	2	1	
NT5E	3	2	1	
NUCB2	3	3	0	
NUDCD3	3	1	2	
NUDT13	3	1	2	
NUDT2	3	1	2	
NUDT3	3	1	2	
NUP107	3	0	3	
NUP98	3	0	3	
NUTF2	3	2	1	
NXF1	3	1	2	
NXN	3	2	1	
NXT2	3	2	1	
OAT	3	1	2	
ODAM	3	1	2	
ODC1	3	2	1	
OFD1	3	2	1	
OGFOD1	3	1	2	
OGFOD2	3	1	2	
OLFML2A	3	1	2	
OLFML3	3	2	1	
OPA3	3	1	2	
OR7E14P	3	2	1	
ORC2	3	3	0	
ORC5	3	2	1	
OS9	3	2	1	
OSBP	3	2	1	
OSBPL2	3	1	2	
OSBPL9	3	1	2	
OSGEP	3	1	2	
OSGEPL1	3	2	1	
OSGIN1	3	2	1	
OSMR	3	1	2	
OSR2	3	2	1	
OSTF1	3	2	1	
OVGP1	3	2	1	
OXA1L	3	1	2	
OXT	3	2	1	
P2RX4	3	1	2	
P2RX5	3	1	2	
P2RY2	3	1	2	

P2RY4	3	2	1	
P2RY6	3	2	1	
P4HA1	3	1	2	
P4HB	3	2	1	
PA2G4	3	1	2	
PABPC1	3	2	1	
PABPN1	3	2	1	
PAEP	3	2	1	
PAF1	3	2	1	
PAH	3	2	1	
PAIP1	3	1	2	
PAK1IP1	3	1	2	
PAK2	3	1	2	
PALB2	3	1	2	
PAM16	3	1	2	
PAN2	3	1	2	
PANK2	3	1	2	
PANX1	3	0	3	
PAQR4	3	2	1	
PAQR6	3	2	1	
PARD6B	3	3	0	
PARP12	3	1	2	
PAX6	3	2	1	
PBK	3	2	1	
PBX2	3	2	1	
PBXIP1	3	3	0	
PC	3	3	0	
PCBP3	3	2	1	
PCDH8	3	1	2	
PCDH9	3	2	1	
PCM1	3	1	2	
PCMT1	3	2	1	
PCNP	3	1	2	
PCNX	3	3	0	
PCYT2	3	2	1	
PDCD10	3	1	2	
PDCD2	3	2	1	
PDCD4	3	1	2	
PDCL	3	2	1	
PDE1A	3	2	1	
PDE4D	3	2	1	
PDE6D	3	1	2	
PDE8A	3	1	2	
PDE8B	3	2	1	
PDGFA	3	1	2	
PDGFB	3	2	1	
PDGFD	3	1	2	
PDGFRL	3	1	2	

PDK3	3	2	1	
PDXDC1	3	1	2	
PDXK	3	1	2	
PDZD2	3	3	0	
PDZK1	3	1	2	
PDZRN3	3	1	2	
PEA15	3	3	0	
PELO	3	2	1	
PER3	3	1	2	
PEX10	3	2	1	
PEX16	3	2	1	
PEX5	3	2	1	
PFAS	3	1	2	
PFDN1	3	1	2	
PFDN2	3	2	1	
PFKP	3	2	1	
PGAM1	3	2	1	
PGAM2	3	2	1	
PGM3	3	1	2	
PGRMC2	3	1	2	
PGS1	3	2	1	
PHGDH	3	0	3	
PHKB	3	2	1	
PHKG2	3	1	2	
PI3	3	2	1	
PIAS1	3	2	1	
PIAS3	3	2	1	
PIBF1	3	2	1	
PICALM	3	2	1	
PIDD	3	2	1	
PIEZO1	3	1	2	
PIEZO2	3	1	2	
PIGC	3	2	1	
PIGF	3	1	2	
PIGP	3	2	1	
PIK3C3	3	2	1	
PIK3IP1	3	2	1	
PILRB	3	1	2	
PIN1	3	1	2	
PINK1	3	1	2	
PIP4K2A	3	1	2	
PIP4K2B	3	2	1	
PIP5K1A	3	3	0	
PISD	3	2	1	
PITPNA	3	1	2	
PITPNB	3	2	1	
PKN2	3	3	0	
PKP3	3	2	1	

PLA2G2D	3	1	2	
PLAGL1	3	2	1	
PLAGL2	3	3	0	
PLCB3	3	1	2	
PLCB4	3	2	1	
PLCD1	3	1	2	
PLCXD1	3	2	1	
PLEK2	3	2	1	
PLEKHA8P1	3	2	1	
PLEKHG3	3	2	1	
PLEKHM2	3	1	2	
PLLP	3	3	0	
PLOD2	3	2	1	
PLP2	3	2	1	
PLXNA1	3	1	2	
PLXNA2	3	2	1	
PLXNC1	3	2	1	
PMEL	3	2	1	
PMP22	3	2	1	
PMS2P3	3	1	2	
PNKP	3	1	2	
PNMA2	3	2	1	
PNP	3	1	2	
PODXL	3	2	1	
POF1B	3	1	2	
POGK	3	1	2	
POGLUT1	3	0	3	
POGZ	3	2	1	
POLA1	3	2	1	
POLA2	3	1	2	
POLB	3	1	2	
POLD2	3	1	2	
POLD3	3	1	2	
POLR2A	3	3	0	
POLR2B	3	2	1	
POLR2K	3	1	2	
POLR2L	3	2	1	
POLR3G	3	1	2	
PON2	3	2	1	
POP4	3	1	2	
POP5	3	0	3	
POR	3	2	1	
POU2F1	3	2	1	
POU6F1	3	2	1	
PPAP2C	3	1	2	
PPARGC1A	3	2	1	
PPCDC	3	2	1	
PPDPF	3	1	2	

PPFIA1	3	1	2	
PPFIBP1	3	2	1	
PPM1B	3	2	1	
PPOX	3	2	1	
PPP1CB	3	2	1	
PPP1R7	3	1	2	
PPP2CB	3	1	2	
PPP2R5B	3	1	2	
PPP3R1	3	2	1	
PPRC1	3	1	2	
PQBP1	3	2	1	
PRDX3	3	2	1	
PRDX4	3	2	1	
PRDX6	3	2	1	
PRELP	3	2	1	
PREP	3	1	2	
PREPL	3	1	2	
PRIM1	3	1	2	
PRKAA1	3	2	1	
PRKAR1A	3	2	1	
PRKAR2A	3	3	0	
PRKAR2B	3	1	2	
PRKD3	3	1	2	
PRKRIR	3	2	1	
PRKY	3	2	1	
PRMT1	3	2	1	
PRMT5	3	1	2	
PRMT7	3	1	2	
PROSAPIP1	3	1	2	
PROX1	3	2	1	
PROZ	3	2	1	
PRPSAP2	3	1	2	
PRR15L	3	2	1	
PRR16	3	1	2	
PRR7	3	2	1	
PRRC1	3	0	3	
PRRC2C	3	1	2	
PRSS22	3	3	0	
PSEN1	3	1	2	
PSG7	3	2	1	
PSG9	3	3	0	
PSIP1	3	2	1	
PSMA6	3	2	1	
PSMB1	3	2	1	
PSMC1	3	1	2	
PSMC2	3	1	2	
PSMC3IP	3	1	2	
PSMD2	3	1	2	

PSMD8	3	2	1	
PSMD9	3	1	2	
PTCH1	3	2	1	
PTDSS2	3	2	1	
PTER	3	1	2	
PTGER3	3	3	0	
PTH1R	3	1	2	
PTMS	3	2	1	
PTPN21	3	3	0	
PTPN3	3	1	2	
PTPRC	3	2	1	
PTRH2	3	1	2	
PTTG1IP	3	1	2	
PURA	3	2	1	
PUS3	3	1	2	
PXMP2	3	1	2	
QKI	3	2	1	
R3HCC1	3	1	2	
RAB27B	3	1	2	
RAB28	3	2	1	
RAB32	3	2	1	
RAB3GAP1	3	2	1	
RAB4A	3	2	1	
RAB7A	3	3	0	
RAB9A	3	1	2	
RABAC1	3	2	1	
RABGAP1	3	1	2	
RABIF	3	1	2	
RAD1	3	2	1	
RAD17	3	1	2	
RAD23B	3	1	2	
RAD54B	3	2	1	
RAF1	3	1	2	
RALA	3	1	2	
RALGAPA1	3	3	0	
RAMP1	3	1	2	
RAN	3	1	2	
RANBP2	3	2	1	
RAPGEFL1	3	2	1	
RARA	3	3	0	
RARG	3	3	0	
RASA1	3	1	2	
RASAL2	3	3	0	
RB1CC1	3	1	2	
RBBP5	3	2	1	
RBFOX2	3	3	0	
RBM15B	3	1	2	
RBM23	3	2	1	

RBM25	3	1	2	
RBM34	3	2	1	
RBM38	3	2	1	
RBM41	3	2	1	
RBM4B	3	1	2	
RBM7	3	1	2	
RBX1	3	2	1	
RC3H2	3	2	1	
RCE1	3	1	2	
RCL1	3	1	2	
RDH11	3	2	1	
RDX	3	0	3	
RECQL	3	2	1	
RECQL5	3	2	1	
REL	3	2	1	
RELA	3	1	2	
RENBP	3	2	1	
RET	3	1	2	
RGS17	3	3	0	
RGS19	3	2	1	
RGS3	3	2	1	
RGS4	3	2	1	
RGS7	3	2	1	
RHBG	3	1	2	
RHOB	3	1	2	
RHOQ	3	1	2	
RHOT1	3	1	2	
RIN2	3	2	1	
RIPK2	3	0	3	
RIPK4	3	2	1	
RLN2	3	2	1	
RMND5A	3	2	1	
RMND5B	3	1	2	
RNASEH1	3	0	3	
RNASEL	3	2	1	
RNASET2	3	2	1	
RNF144A	3	1	2	
RNF146	3	2	1	
RNF19B	3	1	2	
RNF25	3	1	2	
RNF31	3	2	1	
RNF38	3	1	2	
RNF4	3	0	3	
RNF41	3	1	2	
RNGTT	3	2	1	
RNMT	3	1	2	
ROBO4	3	1	2	
RP2	3	1	2	

RPH3A	3	1	2	
RPH3AL	3	1	2	
RPL10A	3	2	1	
RPL11	3	1	2	
RPL22	3	1	2	
RPL24	3	1	2	
RPL28	3	1	2	
RPL34	3	1	2	
RPL35A	3	2	1	
RPL38	3	1	2	
RPL4	3	1	2	
RPL5	3	1	2	
RPL8	3	2	1	
RPLP2	3	1	2	
RPP25	3	2	1	
RPS14P3	3	2	1	
RPS19	3	0	3	
RPS25	3	2	1	
RPS28	3	2	1	
RPS3A	3	2	1	
RPS5	3	1	2	
RPS6KA2	3	2	1	
RPS6KA4	3	1	2	
RPS6KB1	3	3	0	
RPS8	3	1	2	
RPS9	3	1	2	
RPUSD2	3	1	2	
RRAS2	3	2	1	
RREB1	3	2	1	
RRM1	3	2	1	
RRN3	3	2	1	
RRN3P1	3	3	0	
RRP12	3	1	2	
RRP1B	3	1	2	
RRP9	3	1	2	
RSAD2	3	2	1	
RSBN1	3	1	2	
RSF1	3	3	0	
RSRC1	3	1	2	
RSRC2	3	1	2	
RTN4	3	2	1	
RUFY3	3	1	2	
RUNDC3B	3	1	2	
RWDD1	3	1	2	
RXRG	3	3	0	
RYBP	3	2	1	
S100A10	3	1	2	
S100A4	3	2	1	

S100P	3	1	2	
S100PBP	3	2	1	
SAC3D1	3	1	2	
SACM1L	3	2	1	
SACS	3	1	2	
SAGE1	3	2	1	
SAP130	3	1	2	
SAP18	3	0	3	
SAP30L	3	0	3	
SART3	3	1	2	
SATB2	3	1	2	
SAV1	3	0	3	
SBF1	3	2	1	
SBNO1	3	2	1	
SBNO2	3	1	2	
SCAF4	3	1	2	
SCAF8	3	2	1	
SCAI	3	2	1	
SCAMP1	3	1	2	
SCAMP3	3	2	1	
SCAP	3	2	1	
SCFD1	3	2	1	
SCG5	3	1	2	
SCGB1D2	3	3	0	
SCGB2A1	3	3	0	
SCGB2A2	3	1	2	
SCMH1	3	1	2	
SCN1B	3	1	2	
SCNN1B	3	2	1	
SCO2	3	1	2	
SCYL3	3	2	1	
SDAD1	3	0	3	
SDC3	3	2	1	
SDHAP1	3	2	1	
SDHB	3	2	1	
SDHC	3	2	1	
SDS	3	1	2	
SEC13	3	1	2	
SEC16A	3	2	1	
SEC23A	3	2	1	
SEC23B	3	1	2	
SEC24A	3	1	2	
SEC24D	3	2	1	
SEC31A	3	2	1	
SEC63	3	1	2	
SECTM1	3	2	1	
SEH1L	3	0	3	
SELP	3	2	1	

SEMA4C	3	2	1	
SEMA5A	3	1	2	
SEPHS2	3	1	2	
SEPT2	3	2	1	
SEPT6	3	1	2	
SEPT8	3	2	1	
SEPX1	3	2	1	
SERINC5	3	1	2	
SERPINA10	3	2	1	
SERPINA7	3	2	1	
SERPINB2	3	2	1	
SERPINB3	3	2	1	
SERPINI1	3	1	2	
SERTAD2	3	2	1	
SESN1	3	0	3	
SETD1A	3	1	2	
SETD3	3	2	1	
SETD4	3	1	2	
SETMAR	3	2	1	
SF1	3	2	1	
SF3A2	3	2	1	
SF3B1	3	1	2	
SF3B2	3	1	2	
SFMBT1	3	2	1	
SFPQ	3	2	1	
SFRP5	3	2	1	
SFSWAP	3	1	2	
SFTPA2	3	2	1	
SFTPB	3	2	1	
SGMS1	3	2	1	
SGPL1	3	1	2	
SGSH	3	3	0	
SH2D4A	3	1	2	
SH3BGRL3	3	1	2	
SH3GL2	3	2	1	
SH3TC1	3	2	1	
SIAH1	3	2	1	
SIDT2	3	1	2	
SIM2	3	2	1	
SIPA1L1	3	2	1	
SIX1	3	1	2	
SKAP2	3	1	2	
SKIL	3	3	0	
SLC12A1	3	2	1	
SLC12A7	3	2	1	
SLC16A1	3	1	2	
SLC16A6	3	2	1	
SLC16A7	3	2	1	

SLC17A9	3	2	1	
SLC18A2	3	1	2	
SLC1A1	3	1	2	
SLC1A2	3	1	2	
SLC1A5	3	1	2	
SLC25A17	3	2	1	
SLC25A20	3	2	1	
SLC25A38	3	2	1	
SLC25A4	3	3	0	
SLC25A40	3	2	1	
SLC25A44	3	1	2	
SLC25A46	3	0	3	
SLC26A2	3	1	2	
SLC26A6	3	2	1	
SLC2A10	3	1	2	
SLC2A9	3	2	1	
SLC30A3	3	2	1	
SLC30A5	3	1	2	
SLC31A2	3	2	1	
SLC35A1	3	1	2	
SLC35A2	3	2	1	
SLC35D2	3	1	2	
SLC35E2	3	1	2	
SLC35F5	3	3	0	
SLC37A1	3	2	1	
SLC38A2	3	1	2	
SLC39A9	3	3	0	
SLC45A2	3	3	0	
SLC4A1AP	3	1	2	
SLC4A4	3	2	1	
SLC4A8	3	1	2	
SLC5A3	3	2	1	
SLC5A6	3	2	1	
SLC6A4	3	1	2	
SLC7A11	3	0	3	
SLC7A6	3	2	1	
SLC9A1	3	2	1	
SLC9A6	3	0	3	
SLC9A8	3	2	1	
SLCO1B3	3	2	1	
SLIT2	3	2	1	
SLMO1	3	2	1	
SLMO2	3	1	2	
SLTM	3	0	3	
SMAD2	3	2	1	
SMAD7	3	2	1	
SMAP1	3	2	1	
SMARCA2	3	1	2	

SMARCA4	3	2	1	
SMARCA5	3	2	1	
SMARCC1	3	2	1	
SMARCE1	3	1	2	
SMC3	3	0	3	
SMC4	3	2	1	
SMC5	3	3	0	
SMG5	3	2	1	
SMG7	3	3	0	
SMOX	3	2	1	
SMUG1	3	1	2	
SMURF2	3	1	2	
SNAI2	3	2	1	
SNAP29	3	1	2	
SNAPC1	3	1	2	
SNAPC3	3	2	1	
SNCA	3	2	1	
SNORA21	3	1	2	
SNRNP70	3	2	1	
SNRPB	3	1	2	
SNRPD3	3	2	1	
SNRPE	3	2	1	
SNW1	3	2	1	
SNX16	3	3	0	
SNX19	3	1	2	
SNX24	3	1	2	
SOBP	3	2	1	
SOCS1	3	2	1	
SOCS2	3	2	1	
SOCS5	3	2	1	
SON	3	1	2	
SORBS1	3	3	0	
SP140L	3	2	1	
SPAG1	3	2	1	
SPAG9	3	3	0	
SPATA2	3	2	1	
SPATA20	3	2	1	
SPATA7	3	2	1	
SPG11	3	2	1	
SPG20	3	2	1	
SPG21	3	2	1	
SPG7	3	1	2	
SPHK1	3	2	1	
SPIN1	3	1	2	
SPINK4	3	1	2	
SPINT1	3	1	2	
SPOCK2	3	2	1	
SPOP	3	2	1	

SPRED2	3	2	1	
SPTAN1	3	2	1	
SPTSSA	3	2	1	
SQLE	3	2	1	
SQRDL	3	1	2	
SQSTM1	3	3	0	
SRC	3	2	1	
SRD5A3	3	2	1	
SRGAP2	3	2	1	
SRGAP3	3	3	0	
SRPK1	3	2	1	
SRPK2	3	1	2	
SRPR	3	1	2	
SRRD	3	2	1	
SRRM2	3	3	0	
SRSF10	3	1	2	
SRSF11	3	1	2	
SRSF4	3	2	1	
SRSF8	3	1	2	
SS18L1	3	1	2	
SS18L2	3	2	1	
SSB	3	1	2	
SSBP1	3	2	1	
SSBP3	3	2	1	
SSR3	3	1	2	
SSSCA1	3	1	2	
ST3GAL4	3	3	0	
ST3GAL6	3	2	1	
ST6GAL1	3	3	0	
ST6GALNAC4	3	2	1	
STAM2	3	2	1	
STAMBP	3	1	2	
STAP1	3	2	1	
STARD13	3	1	2	
STAT3	3	2	1	
STAT5A	3	3	0	
STAT5B	3	0	3	
STAT6	3	1	2	
STC2	3	0	3	
STEAP3	3	1	2	
STEAP4	3	2	1	
STIM1	3	2	1	
STK10	3	2	1	
STK17B	3	2	1	
STK24	3	1	2	
STK38L	3	1	2	
STMN1	3	1	2	
STMN2	3	2	1	

STOML2	3	2	1	
STRA13	3	2	1	
STRADA	3	2	1	
STRAP	3	0	3	
STRN4	3	2	1	
STX16	3	2	1	
STX1A	3	1	2	
STX3	3	2	1	
STX7	3	1	2	
STXBP2	3	1	2	
SUB1	3	1	2	
SUCLA2	3	2	1	
SUMO2	3	2	1	
SUN1	3	3	0	
SUN2	3	1	2	
SURF2	3	0	3	
SUV39H2	3	1	2	
SUV420H1	3	1	2	
SUZ12P	3	2	1	
SV2B	3	2	1	
SVIL	3	2	1	
SWAP70	3	2	1	
SYBU	3	0	3	
SYCP2	3	1	2	
SYNGR2	3	2	1	
SYNJ1	3	2	1	
SYPL1	3	1	2	
TAB2	3	2	1	
TACSTD2	3	2	1	
TAF11	3	1	2	
TAF1A	3	1	2	
TAP1	3	1	2	
TARP	3	2	1	
TASP1	3	2	1	
TATDN2	3	2	1	
TAX1BP1	3	1	2	
TBC1D10B	3	1	2	
TBC1D2B	3	2	1	
TBC1D30	3	2	1	
TBC1D4	3	1	2	
TBC1D8	3	3	0	
TBC1D9B	3	2	1	
TBCA	3	2	1	
TBCCD1	3	0	3	
TBKBP1	3	1	2	
TBP	3	2	1	
TBPL1	3	3	0	
TCEB2	3	2	1	

TCEB3	3	0	3	
TCF3	3	3	0	
TCFL5	3	1	2	
TCIRG1	3	2	1	
TCL1A	3	2	1	
TCTA	3	2	1	
TDG	3	2	1	
TDRD7	3	1	2	
TEP1	3	2	1	
TERT	3	2	1	
TESK2	3	1	2	
TEX261	3	1	2	
TEX264	3	2	1	
TFAM	3	1	2	
TFAP2A	3	3	0	
TFCP2L1	3	1	2	
TFF2	3	1	2	
TFF3	3	2	1	
TFG	3	1	2	
TFIP11	3	2	1	
TFPI	3	1	2	
TFPI2	3	2	1	
TGDS	3	1	2	
TGFB2	3	3	0	
TGFBI	3	1	2	
TGFBR2	3	0	3	
TGM2	3	3	0	
THADA	3	2	1	
THAP4	3	0	3	
THAP7	3	2	1	
THNSL1	3	1	2	
THOC1	3	1	2	
THOC2	3	1	2	
THRAP3	3	2	1	
THSD7A	3	2	1	
THUMPD1	3	1	2	
THYN1	3	2	1	
TIMM10	3	1	2	
TIMM44	3	2	1	
TIMM8B	3	2	1	
TIMM9	3	2	1	
TIMP2	3	2	1	
TINAGL1	3	2	1	
TIPIN	3	1	2	
TJP3	3	1	2	
TK2	3	1	2	
TLE4	3	3	0	
TLR2	3	1	2	

TM9SF1	3	2	1	
TMBIM1	3	1	2	
TMCO1	3	2	1	
TMCO3	3	1	2	
TMEFF1	3	2	1	
TMEM14A	3	1	2	
TMEM14B	3	2	1	
TMEM159	3	1	2	
TMEM177	3	2	1	
TMEM184B	3	1	2	
TMEM184C	3	2	1	
TMEM2	3	1	2	
TMEM231	3	2	1	
TMEM30B	3	2	1	
TMEM41B	3	1	2	
TMEM5	3	2	1	
TMEM50A	3	1	2	
TMEM70	3	1	2	
TMEM8A	3	1	2	
TMEM93	3	1	2	
TMEM97	3	1	2	
TMOD1	3	1	2	
TMOD3	3	1	2	
TMPRSS11E	3	1	2	
TNFAIP1	3	1	2	
TNFRSF10B	3	2	1	
TNFRSF11B	3	1	2	
TNFSF13	3	1	2	
TNFSF9	3	1	2	
TNIK	3	1	2	
TNPO2	3	2	1	
TNS3	3	1	2	
TOB2	3	2	1	
TOLLIP	3	2	1	
TOMM20	3	0	3	
TOMM22	3	2	1	
TOP1	3	2	1	
TOP2B	3	2	1	
TOP3A	3	3	0	
TOR1B	3	2	1	
TP53BP1	3	1	2	
TPD52	3	2	1	
TPD52L2	3	1	2	
TPM2	3	1	2	
TPO	3	2	1	
TPP1	3	2	1	
TPPP3	3	2	1	
TPR	3	2	1	

TPSB2	3	1	2	
TPST1	3	1	2	
TRA2A	3	3	0	
TRADD	3	2	1	
TRAFD1	3	1	2	
TRAM1	3	1	2	
TRAPPC10	3	1	2	
TRAPPC2	3	1	2	
TRAPPC2L	3	2	1	
TRAPPC6A	3	1	2	
TRAT1	3	2	1	
TRIM14	3	2	1	
TRIM16	3	1	2	
TRIM17	3	2	1	
TRIM2	3	2	1	
TRIM26	3	1	2	
TRIM36	3	2	1	
TRIM37	3	2	1	
TRIM38	3	1	2	
TRIM9	3	2	1	
TRIO	3	2	1	
TRIP13	3	2	1	
TRIT1	3	2	1	
TRMT112	3	2	1	
TRMT12	3	2	1	
TRMT2B	3	1	2	
TRMT61B	3	2	1	
TRMU	3	1	2	
TRNAU1AP	3	1	2	
TRPC4AP	3	1	2	
TRPV2	3	2	1	
TSC1	3	3	0	
TSC22D2	3	3	0	
TSEN2	3	1	2	
TSFM	3	2	1	
TSPAN13	3	2	1	
TSPAN6	3	1	2	
TSPAN7	3	1	2	
TSPYL5	3	1	2	
TST	3	1	2	
TTC1	3	1	2	
TTC13	3	1	2	
TTC17	3	2	1	
TTC23	3	2	1	
TTC33	3	1	2	
TTC38	3	3	0	
TTLL12	3	2	1	
TTLL4	3	2	1	

TUBB3	3	1	2	
TUFT1	3	2	1	
TUG1	3	1	2	
TULP4	3	1	2	
TUSC2	3	1	2	
TWF2	3	2	1	
TWSG1	3	2	1	
TXN2	3	2	1	
TXNIP	3	2	1	
TXNRD2	3	3	0	
TYMS	3	1	2	
TYR	3	2	1	
U2AF2	3	2	1	
U2SURP	3	1	2	
UAP1L1	3	2	1	
UBE2A	3	1	2	
UBE2B	3	2	1	
UBE2I	3	2	1	
UBE2J1	3	1	2	
UBE2Q1	3	2	1	
UBE2S	3	1	2	
UBR5	3	2	1	
UBR7	3	0	3	
UBXN1	3	2	1	
UBXN7	3	1	2	
UBXN8	3	1	2	
UCHL5	3	2	1	
UCKL1	3	2	1	
UCN	3	2	1	
UFC1	3	2	1	
UGGT1	3	2	1	
UGT2A3	3	1	2	
UGT2B15	3	2	1	
UHRF1BP1L	3	3	0	
UIMC1	3	1	2	
ULK2	3	2	1	
UMOD	3	2	1	
UNC119	3	1	2	
UNC50	3	1	2	
UNC5B	3	1	2	
UPF3A	3	0	3	
UQCC	3	2	1	
UQCRC2	3	2	1	
UQCRH	3	1	2	
USP14	3	0	3	
USP15	3	1	2	
USP16	3	1	2	
USP18	3	2	1	

USP34	3	2	1	
USP39	3	1	2	
USPL1	3	1	2	
UST	3	1	2	
UTP18	3	1	2	
UTY	3	2	1	
UXS1	3	1	2	
UXT	3	2	1	
VASP	3	2	1	
VCPIP1	3	3	0	
VDAC1	3	1	2	
VEZF1	3	1	2	
VGLL3	3	2	1	
VIL1	3	2	1	
VKORC1	3	2	1	
VOPP1	3	1	2	
VPS11	3	2	1	
VPS13D	3	2	1	
VPS4A	3	1	2	
VPS54	3	1	2	
VPS72	3	2	1	
VPS8	3	1	2	
VRK3	3	2	1	
VTI1B	3	2	1	
VTN	3	2	1	
VWA5A	3	1	2	
WAS	3	2	1	
WASL	3	2	1	
WBP11	3	1	2	
WBP4	3	2	1	
WDFY3	3	2	1	
WDR18	3	2	1	
WDR26	3	2	1	
WDR41	3	1	2	
WDR43	3	1	2	
WDR44	3	1	2	
WDR45	3	1	2	
WDR59	3	3	0	
WDR62	3	1	2	
WDR74	3	1	2	
WEE1	3	2	1	
WHSC1	3	2	1	
WIPF2	3	1	2	
WIPI1	3	2	1	
WIPI2	3	1	2	
WIZ	3	2	1	
WRAP53	3	2	1	
WRN	3	2	1	

WSB1	3	2	1	
WSCD1	3	2	1	
WWP1	3	1	2	
WWP2	3	2	1	
WWTR1	3	3	0	
XAF1	3	1	2	
XBP1	3	1	2	
XIAP	3	3	0	
XPA	3	1	2	
XPC	3	1	2	
XPNPEP2	3	2	1	
XRCC1	3	1	2	
YEATS2	3	2	1	
YIPF1	3	1	2	
YIPF2	3	1	2	
YIPF3	3	1	2	
YOD1	3	2	1	
YPEL5	3	1	2	
YTHDF3	3	1	2	
YWHAQ	3	2	1	
YY1AP1	3	2	1	
ZBED5	3	2	1	
ZBTB11	3	1	2	
ZBTB24	3	1	2	
ZBTB38	3	1	2	
ZBTB39	3	1	2	
ZBTB48	3	2	1	
ZBTB5	3	2	1	
ZC3H14	3	0	3	
ZC3H7A	3	2	1	
ZC3HAV1	3	1	2	
ZCCHC14	3	2	1	
ZCCHC2	3	1	2	
ZDHHC14	3	1	2	
ZDHHC17	3	3	0	
ZDHHC4	3	1	2	
ZDHHC6	3	1	2	
ZFAND6	3	1	2	
ZFC3H1	3	1	2	
ZFHX4	3	2	1	
ZFP106	3	1	2	
ZFP30	3	1	2	
ZFP36L1	3	3	0	
ZFR	3	2	1	
ZFYVE16	3	2	1	
ZFYVE21	3	1	2	
ZFYVE26	3	2	1	
ZG16	3	1	2	

ZGPAT	3	1	2	
ZHX2	3	2	1	
ZIC1	3	1	2	
ZKSCAN5	3	1	2	
ZMIZ1	3	2	1	
ZMYM2	3	3	0	
ZMYM5	3	2	1	
ZMYND8	3	2	1	
ZNF12	3	1	2	
ZNF133	3	1	2	
ZNF134	3	2	1	
ZNF136	3	1	2	
ZNF143	3	1	2	
ZNF185	3	2	1	
ZNF200	3	2	1	
ZNF202	3	1	2	
ZNF207	3	1	2	
ZNF211	3	2	1	
ZNF226	3	2	1	
ZNF24	3	1	2	
ZNF268	3	2	1	
ZNF271	3	1	2	
ZNF281	3	1	2	
ZNF282	3	2	1	
ZNF304	3	1	2	
ZNF324	3	3	0	
ZNF33B	3	2	1	
ZNF35	3	2	1	
ZNF354A	3	1	2	
ZNF358	3	2	1	
ZNF394	3	2	1	
ZNF467	3	3	0	
ZNF500	3	1	2	
ZNF510	3	2	1	
ZNF544	3	1	2	
ZNF573	3	2	1	
ZNF576	3	2	1	
ZNF580	3	2	1	
ZNF589	3	1	2	
ZNF592	3	2	1	
ZNF606	3	1	2	
ZNF609	3	3	0	
ZNF623	3	1	2	
ZNF652	3	1	2	
ZNF688	3	2	1	
ZNF710	3	1	2	
ZNF764	3	2	1	
ZNF83	3	1	2	

ZNF835	3	2	1	
ZNF85	3	1	2	
ZNF93	3	1	2	
ZSCAN16	3	1	2	
ZWILCH	3	1	2	
ZXDC	3	0	3	
ABCA3	2	0	2	
ABCA8	2	0	2	
ABCC2	2	2	0	
ABCC5	2	0	2	
ABCC6	2	2	0	
ABCC9	2	2	0	
ABHD8	2	2	0	
ABI2	2	2	0	
ABO	2	2	0	
ABTB2	2	0	2	
ACAD8	2	2	0	
ACCN1	2	2	0	
ACE	2	2	0	
ACE2	2	2	0	
ACPP	2	2	0	
ACSBG2	2	2	0	
ACSF2	2	0	2	
ACTL7B	2	2	0	
ACTR1A	2	0	2	
ACVR1B	2	2	0	
ADA	2	2	0	
ADAM11	2	2	0	
ADAM12	2	2	0	
ADAMTS5	2	0	2	
ADAMTS6	2	2	0	
ADAMTS7	2	2	0	
ADAMTS8	2	2	0	
ADAMTS9	2	0	2	
ADARB2	2	2	0	
ADCY10	2	2	0	
ADCY2	2	2	0	
ADCY8	2	2	0	
ADH1A	2	2	0	
ADK	2	0	2	
ADRA1D	2	2	0	
ADRB2	2	2	0	
ADRBK1	2	2	0	
ADRBK2	2	2	0	
AFM	2	2	0	
AGPAT1	2	0	2	
AGRN	2	2	0	
AHCYL1	2	2	0	

AHNAK2	2	0	2	
AHR	2	0	2	
AICDA	2	2	0	
AIF1	2	2	0	
AIMP2	2	0	2	
AKAP17A	2	2	0	
AKAP5	2	2	0	
AKAP8L	2	2	0	
AKR1C1	2	0	2	
AKT2	2	2	0	
ALAS2	2	0	2	
ALDH1B1	2	2	0	
ALDH1L1	2	0	2	
ALDH7A1	2	2	0	
ALDOA	2	2	0	
ALOX15B	2	2	0	
ALOX5	2	2	0	
ALPP	2	2	0	
ALS2CR8	2	2	0	
ALX1	2	2	0	
AMACR	2	2	0	
AMOT	2	2	0	
AMPD3	2	2	0	
ANAPC1	2	2	0	
ANAPC5	2	0	2	
ANK2	2	2	0	
ANKLE2	2	0	2	
ANKMY1	2	2	0	
ANKRD40	2	2	0	
ANKRD53	2	2	0	
ANXA6	2	0	2	
ANXA7	2	0	2	
AP1G1	2	0	2	
AP2A2	2	2	0	
AP4M1	2	2	0	
APC	2	2	0	
APIP	2	0	2	
APOA2	2	2	0	
APOF	2	2	0	
APOL5	2	2	0	
AQP3	2	0	2	
AQP5	2	0	2	
AQP7	2	2	0	
AQP8	2	2	0	
AREG	2	2	0	
ARHGAP22	2	2	0	
ARHGAP26	2	2	0	
ARHGAP33	2	2	0	

ARHGEF16	2	2	0	
ARHGEF17	2	2	0	
ARHGEF18	2	0	2	
ARHGEF4	2	2	0	
ARL4D	2	2	0	
ARL5A	2	2	0	
ARPC1A	2	0	2	
ARPC5	2	2	0	
ARPP19	2	2	0	
ARRB2	2	2	0	
ARSD	2	2	0	
ARSE	2	2	0	
ARSJ	2	2	0	
ART1	2	2	0	
ART4	2	2	0	
ARTN	2	2	0	
ASAP1	2	0	2	
ASAP2	2	0	2	
ASGR1	2	2	0	
ASIP	2	2	0	
ASMT	2	2	0	
ASNS	2	0	2	
ASNSD1	2	0	2	
ASPN	2	0	2	
ASS1	2	2	0	
ATF1	2	0	2	
ATF3	2	0	2	
ATF7IP	2	2	0	
ATG10	2	2	0	
ATG12	2	2	0	
ATG16L1	2	2	0	
ATG3	2	0	2	
ATG7	2	2	0	
ATP10B	2	2	0	
ATP11B	2	2	0	
ATP2A3	2	2	0	
ATP2B4	2	2	0	
ATP5B	2	0	2	
ATP5F1	2	2	0	
ATP6AP2	2	2	0	
ATP6V1E1	2	0	2	
ATP6V1G2	2	2	0	
ATP8A1	2	2	0	
ATR	2	0	2	
ATRN	2	0	2	
ATXN7L1	2	2	0	
AUH	2	2	0	
AVEN	2	0	2	

AVIL	2	2	0	
B3GALT2	2	2	0	
B4GALT6	2	2	0	
BACE2	2	0	2	
BARX1	2	2	0	
BAX	2	2	0	
BBIP1	2	0	2	
BCAR3	2	0	2	
BCAS2	2	0	2	
BCAS3	2	2	0	
BCCIP	2	0	2	
BCKDHB	2	0	2	
BCL2A1	2	0	2	
BCL3	2	2	0	
BEX1	2	0	2	
BICC1	2	2	0	
BIN3	2	0	2	
BLCAP	2	0	2	
BLMH	2	2	0	
BLZF1	2	2	0	
BMP1	2	2	0	
BMP10	2	2	0	
BMP5	2	2	0	
BRCA1	2	0	2	
BRD1	2	2	0	
BRD7P3	2	2	0	
BRS3	2	2	0	
BSDC1	2	0	2	
BTC	2	2	0	
BTG2	2	2	0	
BTNL3	2	2	0	
BZRAP1	2	0	2	
C10ORF137	2	2	0	
C11ORF2	2	2	0	
C11ORF49	2	2	0	
C11ORF51	2	2	0	
C11ORF73	2	0	2	
C11ORF9	2	2	0	
C12ORF10	2	0	2	
C12ORF41	2	0	2	
C12ORF43	2	0	2	
C13ORF27	2	0	2	
C14ORF132	2	2	0	
C14ORF162	2	2	0	
C14ORF56	2	2	0	
C14ORF79	2	2	0	
C15ORF29	2	0	2	
C15ORF39	2	2	0	

C16ORF62	2	2	0	
C17ORF81	2	2	0	
C18ORF10	2	2	0	
C19ORF22	2	2	0	
C19ORF50	2	2	0	
C19ORF6	2	2	0	
C19ORF60	2	2	0	
C19ORF73	2	2	0	
C19ORF80	2	2	0	
C1ORF106	2	2	0	
C1ORF144	2	2	0	
C1ORF27	2	0	2	
C1QA	2	2	0	
C2	2	2	0	
C20ORF111	2	0	2	
C20ORF195	2	2	0	
C20ORF20	2	2	0	
C20ORF24	2	0	2	
C20ORF4	2	0	2	
C21ORF2	2	2	0	
C21ORF7	2	2	0	
C21ORF96	2	2	0	
C22ORF24	2	2	0	
C22ORF26	2	2	0	
C22ORF43	2	2	0	
C2CD2	2	0	2	
C2CD2L	2	0	2	
C2ORF43	2	2	0	
C2ORF49	2	0	2	
C2ORF54	2	2	0	
C2ORF83	2	2	0	
C3ORF32	2	2	0	
C3ORF51	2	2	0	
C3ORF75	2	2	0	
C5ORF28	2	2	0	
C5ORF44	2	2	0	
C6ORF123	2	2	0	
C7ORF28B	2	2	0	
C7ORF54	2	2	0	
C7ORF58	2	2	0	
C8G	2	2	0	
C8ORF55	2	2	0	
C9ORF156	2	2	0	
C9ORF46	2	0	2	
C9ORF53	2	2	0	
C9ORF7	2	2	0	
C9ORF78	2	0	2	
C9ORF95	2	0	2	

CA1	2	2	0	
CA5B	2	2	0	
CACNA1F	2	2	0	
CACNA1G	2	2	0	
CACNA2D1	2	2	0	
CACNA2D2	2	2	0	
CACNB3	2	0	2	
CACNB4	2	2	0	
CACNG2	2	2	0	
CADM4	2	2	0	
CADPS	2	2	0	
CALCR	2	2	0	
CALD1	2	2	0	
CALHM2	2	2	0	
CALML5	2	2	0	
CAMK1G	2	2	0	
CAMK2A	2	2	0	
CAMK2G	2	2	0	
CAPN10	2	2	0	
CAPN11	2	2	0	
CAPN3	2	0	2	
CAPN6	2	2	0	
CARS	2	0	2	
CARTPT	2	2	0	
CASP10	2	2	0	
CASP3	2	2	0	
CASR	2	2	0	
CASS4	2	2	0	
CBL	2	2	0	
CBLC	2	2	0	
CBX7	2	0	2	
CCDC109B	2	2	0	
CCDC165	2	2	0	
CCDC28B	2	2	0	
CCDC30	2	2	0	
CCDC40	2	2	0	
CCDC59	2	0	2	
CCDC64	2	2	0	
CCDC85B	2	2	0	
CCL11	2	2	0	
CCL13	2	2	0	
CCL4	2	2	0	
CCNA1	2	2	0	
CCND2	2	2	0	
CCNG1	2	0	2	
CCNJL	2	2	0	
CCR7	2	0	2	
CCR8	2	2	0	

CCR9	2	2	0	
CCRL1	2	2	0	
CD163	2	2	0	
CD2AP	2	2	0	
CD300C	2	2	0	
CD302	2	0	2	
CD3EAP	2	0	2	
CD53	2	2	0	
CD68	2	2	0	
CD83	2	2	0	
CD84	2	2	0	
CD93	2	2	0	
CD97	2	2	0	
CDADC1	2	2	0	
CDC14A	2	2	0	
CDC20	2	2	0	
CDC37L1	2	0	2	
CDC42EP3	2	2	0	
CDC7	2	0	2	
CDH1	2	0	2	
CDH11	2	2	0	
CDH5	2	2	0	
CDH6	2	2	0	
CDH8	2	2	0	
CDH9	2	2	0	
CDHR1	2	2	0	
CDK11A	2	0	2	
CDK13	2	2	0	
CDK18	2	2	0	
CDK5R1	2	2	0	
CDKL1	2	2	0	
CDKL2	2	2	0	
CDKN2D	2	2	0	
CDKN3	2	0	2	
CDS1	2	2	0	
CDX1	2	2	0	
CEBPA	2	2	0	
CEBPB	2	0	2	
CEBPE	2	2	0	
CEBPZ	2	2	0	
CECR5	2	2	0	
CELA2A	2	2	0	
CELA3A	2	2	0	
CELA3B	2	2	0	
CELP	2	2	0	
CELSR1	2	2	0	
CENPE	2	2	0	
CENPM	2	2	0	

CENPO	2	2	0	
CENPQ	2	2	0	
CEP135	2	2	0	
CEP72	2	2	0	
CERS4	2	0	2	
CETN1	2	2	0	
CETN2	2	0	2	
CFB	2	2	0	
CFTR	2	2	0	
CHAT	2	2	0	
CHCHD2	2	0	2	
CHCHD3	2	2	0	
CHD8	2	2	0	
CHGB	2	2	0	
CHIC2	2	2	0	
CHML	2	2	0	
CHMP5	2	0	2	
CHMP7	2	0	2	
CHODL	2	2	0	
CHORDC1	2	0	2	
CHP	2	0	2	
CHRNA2	2	2	0	
CHRNA5	2	2	0	
CHRNA6	2	2	0	
CHST3	2	2	0	
CHST4	2	2	0	
CHST7	2	2	0	
CHSY1	2	0	2	
CIB2	2	2	0	
CIITA	2	2	0	
CIRBP	2	0	2	
CISD1	2	2	0	
CKAP2	2	0	2	
CLCN2	2	0	2	
CLDN3	2	2	0	
CLDN5	2	2	0	
CLDN9	2	2	0	
CLEC3B	2	0	2	
CLIC4	2	0	2	
CLN3	2	0	2	
CLN5	2	0	2	
CLPS	2	2	0	
CLPTM1	2	2	0	
CLTA	2	2	0	
CMA1	2	2	0	
CMTM6	2	0	2	
CNDP2	2	2	0	
CNKSR2	2	2	0	

CNN3	2	2	0	
CNNM4	2	2	0	
CNOT4	2	2	0	
CNR1	2	2	0	
COASY	2	0	2	
COBL	2	0	2	
COL10A1	2	0	2	
COL13A1	2	2	0	
COL18A1	2	2	0	
COL19A1	2	2	0	
COL1A2	2	2	0	
COL4A3	2	2	0	
COL4A4	2	2	0	
COL6A1	2	2	0	
COLEC11	2	2	0	
COLEC12	2	2	0	
COPA	2	2	0	
COPS4	2	2	0	
COQ6	2	0	2	
CORO1A	2	2	0	
CORO2B	2	2	0	
CORO7	2	2	0	
COX11	2	0	2	
COX15	2	0	2	
COX6A1	2	0	2	
COX7A1	2	0	2	
COX7B	2	0	2	
CPA1	2	2	0	
CPA3	2	2	0	
CPN1	2	2	0	
CR2	2	2	0	
CRABP1	2	2	0	
CRB1	2	2	0	
CRCP	2	2	0	
CREB3L1	2	2	0	
CRLF1	2	2	0	
CRLF3	2	0	2	
CRX	2	2	0	
CRYBB1	2	2	0	
CRYBB2	2	2	0	
CRYZL1	2	0	2	
CS	2	0	2	
CSF1	2	2	0	
CSF1R	2	2	0	
CSF2RB	2	2	0	
CSGALNACT1	2	0	2	
CSGALNACT2	2	0	2	
CSN3	2	2	0	

CSNK1A1	2	0	2	
CSNK2B	2	0	2	
CSRNP3	2	2	0	
CSRP1	2	2	0	
CST5	2	2	0	
CST6	2	0	2	
CTBP2	2	2	0	
CTNNA1	2	2	0	
CTNNBIP1	2	2	0	
CTNNBL1	2	0	2	
CTNS	2	2	0	
CTSH	2	2	0	
CTSL1	2	0	2	
CUTC	2	0	2	
CXCL10	2	2	0	
CXCL11	2	2	0	
CXCL14	2	0	2	
CXCL9	2	0	2	
CXCR4	2	2	0	
CXCR7	2	0	2	
CXORF27	2	2	0	
CXORF40A	2	0	2	
CXORF40B	2	0	2	
CXORF57	2	2	0	
CYFIP1	2	0	2	
CYP1A1	2	0	2	
CYP2B7P1	2	0	2	
CYP39A1	2	2	0	
CYP3A43	2	2	0	
CYP4A11	2	2	0	
CYP4F3	2	2	0	
CYP4F8	2	2	0	
CYTIP	2	2	0	
DAAM1	2	2	0	
DAB1	2	2	0	
DAD1	2	2	0	
DALRD3	2	0	2	
DAPK1	2	2	0	
DAXX	2	0	2	
DBF4	2	0	2	
DBF4B	2	2	0	
DBNDD1	2	2	0	
DBR1	2	2	0	
DCAF11	2	2	0	
DCAF4	2	2	0	
DCAKD	2	2	0	
DCHS1	2	2	0	
DCLK1	2	2	0	

DCTN1	2	2	0	
DCTN3	2	2	0	
DCTPP1	2	2	0	
DDB2	2	0	2	
DDC	2	2	0	
DDI2	2	2	0	
DDIT3	2	0	2	
DDR1	2	2	0	
DDX17	2	2	0	
DDX18	2	0	2	
DDX39A	2	0	2	
DDX4	2	2	0	
DDX50	2	0	2	
DDX56	2	0	2	
DECR1	2	0	2	
DEFA5	2	2	0	
DEFA6	2	2	0	
DENND1B	2	2	0	
DENND1C	2	2	0	
DEPDC1	2	2	0	
DFNA5	2	2	0	
DGCR2	2	2	0	
DGKE	2	2	0	
DHDDS	2	0	2	
DHODH	2	2	0	
DHRS9	2	0	2	
DHX35	2	2	0	
DIABLO	2	0	2	
DIAPH3	2	2	0	
DIMT1	2	2	0	
DKC1	2	0	2	
DKFZP547G183	2	2	0	
DKFZP686O1327	2	2	0	
DKK2	2	2	0	
DLG3	2	2	0	
DLGAP1	2	2	0	
DLK1	2	2	0	
DLX4	2	2	0	
DMPK	2	2	0	
DMXL2	2	2	0	
DNAAF1	2	2	0	
DNAH2	2	2	0	
DNAH3	2	2	0	
DNAI1	2	2	0	
DNAJA3	2	0	2	
DNAJC28	2	2	0	
DNAJC9	2	0	2	
DNASE1L1	2	0	2	

DNMT3A	2	2	0	
DNTT	2	2	0	
DOCK9	2	2	0	
DOHH	2	2	0	
DOPEY1	2	2	0	
DOPEY2	2	2	0	
DOT1L	2	2	0	
DPH2	2	0	2	
DPM1	2	0	2	
DPP4	2	2	0	
DPPA4	2	2	0	
DPYSL4	2	2	0	
DRD1	2	2	0	
DRD4	2	2	0	
DRD5	2	2	0	
DRG1	2	0	2	
DSCR3	2	2	0	
DSCR6	2	2	0	
DSN1	2	0	2	
DSTYK	2	2	0	
DTNA	2	2	0	
DTNB	2	2	0	
DTX2P1	2	2	0	
DUSP11	2	2	0	
DUSP12	2	0	2	
DUSP21	2	2	0	
DUX2	2	2	0	
DYM	2	2	0	
DYNC2H1	2	2	0	
DYNC2LI1	2	0	2	
DYRK1A	2	2	0	
DYSF	2	2	0	
E2F1	2	2	0	
EAPP	2	0	2	
EBI3	2	2	0	
ECI2	2	0	2	
EDA	2	2	0	
EDDM3A	2	2	0	
EDN2	2	2	0	
EEF1A1	2	0	2	
EEF1G	2	0	2	
EFNA5	2	2	0	
EGR4	2	2	0	
EHD3	2	0	2	
EHMT1	2	2	0	
EHMT2	2	0	2	
EI24	2	2	0	
EIF2AK3	2	2	0	

EIF2B1	2	0	2	
EIF2S2	2	0	2	
ELAC1	2	2	0	
ELAVL4	2	2	0	
ELL	2	2	0	
ELOVL5	2	2	0	
ELP4	2	2	0	
EMCN	2	2	0	
EMILIN1	2	2	0	
EML2	2	2	0	
EMP3	2	0	2	
EMR2	2	2	0	
EMR3	2	2	0	
EN1	2	2	0	
ENPP2	2	0	2	
ENY2	2	0	2	
EOGT	2	0	2	
EPAS1	2	2	0	
EPB41	2	2	0	
EPB41L4A	2	2	0	
EPHA1	2	2	0	
EPHA3	2	2	0	
EPHB1	2	2	0	
EPHB4	2	2	0	
EPHX2	2	2	0	
EPOR	2	2	0	
EPRS	2	0	2	
ERAL1	2	2	0	
ERAP1	2	2	0	
ERBB3	2	2	0	
ERCC6L	2	2	0	
ERMP1	2	2	0	
ERO1L	2	0	2	
ESR2	2	2	0	
ESRRA	2	2	0	
ESYT1	2	0	2	
ETFB	2	2	0	
ETFDH	2	0	2	
ETNK1	2	2	0	
ETS1	2	2	0	
ETV6	2	2	0	
ETV7	2	2	0	
EVC	2	2	0	
EWSR1	2	2	0	
EXOC1	2	0	2	
EXOSC10	2	0	2	
EXOSC2	2	2	0	
EXT2	2	2	0	

EYA3	2	2	0	
EZH1	2	2	0	
F12	2	2	0	
F8	2	0	2	
F9	2	0	2	
FABP3	2	0	2	
FABP7	2	2	0	
FAM105A	2	2	0	
FAM111A	2	0	2	
FAM118A	2	2	0	
FAM120A	2	2	0	
FAM13A	2	2	0	
FAM13B	2	2	0	
FAM149A	2	2	0	
FAM153A	2	2	0	
FAM155A	2	2	0	
FAM164C	2	2	0	
FAM189A1	2	2	0	
FAM18B1	2	0	2	
FAM193B	2	2	0	
FAM198B	2	2	0	
FAM204A	2	2	0	
FAM208B	2	0	2	
FAM48A	2	2	0	
FAM49A	2	2	0	
FAM49B	2	2	0	
FAM60A	2	0	2	
FAM63B	2	2	0	
FAM90A1	2	2	0	
FANCA	2	2	0	
FAP	2	2	0	
FAS	2	0	2	
FAT1	2	0	2	
FBL	2	2	0	
FBLN1	2	2	0	
FBXL14	2	0	2	
FBXL2	2	2	0	
FBXL6	2	2	0	
FBXL8	2	2	0	
FBXO11	2	0	2	
FBXO21	2	2	0	
FBXO34	2	0	2	
FBXO40	2	2	0	
FBXO41	2	2	0	
FBXO46	2	2	0	
FBXO9	2	0	2	
FCAR	2	2	0	
FCGRT	2	2	0	

FCHO1	2	0	2	
FDX1	2	0	2	
FER	2	2	0	
FFAR2	2	2	0	
FGB	2	2	0	
FGD2	2	2	0	
FGF13	2	0	2	
FGF14	2	2	0	
FGF18	2	2	0	
FGF22	2	2	0	
FGF9	2	2	0	
FGFR4	2	2	0	
FHL1	2	0	2	
FHL2	2	2	0	
FKBP10	2	2	0	
FKBP4	2	2	0	
FKBP6	2	2	0	
FKSG2	2	2	0	
FKTN	2	0	2	
FLAD1	2	0	2	
FLJ11292	2	2	0	
FLJ13224	2	2	0	
FLJ22184	2	2	0	
FLNB	2	0	2	
FLOT2	2	2	0	
FLT4	2	2	0	
FLVCR2	2	0	2	
FN1	2	2	0	
FOLR1	2	0	2	
FOSB	2	0	2	
FOXA2	2	2	0	
FOXD1	2	2	0	
FOXE1	2	2	0	
FOXH1	2	2	0	
FOXO1	2	0	2	
FOXO3	2	0	2	
FPR2	2	2	0	
FRMD4B	2	2	0	
FRMD8	2	2	0	
FRS2	2	2	0	
FSCN3	2	2	0	
FSD1	2	2	0	
FSTL4	2	2	0	
FTO	2	0	2	
FTSJ1	2	2	0	
FUT2	2	2	0	
FUT6	2	2	0	
FUT8	2	2	0	

FUT9	2	2	0	
FZD7	2	0	2	
FZR1	2	2	0	
G6PC	2	2	0	
GABRA1	2	2	0	
GABRA4	2	2	0	
GABRA5	2	2	0	
GABRB2	2	2	0	
GABRG3	2	2	0	
GAD2	2	2	0	
GAGE3	2	2	0	
GALC	2	2	0	
GALNT10	2	0	2	
GALNT8	2	2	0	
GAN	2	2	0	
GAS2L1	2	2	0	
GATA1	2	2	0	
GBP1	2	2	0	
GBX1	2	2	0	
GCC1	2	0	2	
GCC2	2	0	2	
GCKR	2	2	0	
GCLC	2	2	0	
GCN1L1	2	0	2	
GCNT1	2	0	2	
GCNT2	2	2	0	
GDAP1	2	2	0	
GDF3	2	2	0	
GDF9	2	2	0	
GDPD3	2	2	0	
GFOD1	2	2	0	
GFRA1	2	2	0	
GFRA3	2	2	0	
GFRA4	2	2	0	
GGA2	2	2	0	
GGA3	2	2	0	
GGTLC1	2	2	0	
GHRH	2	2	0	
GINS1	2	2	0	
GJA5	2	2	0	
GJA9	2	2	0	
GJB3	2	2	0	
GJB4	2	2	0	
GK2	2	2	0	
GLG1	2	0	2	
GLI1	2	2	0	
GLI2	2	2	0	
GLI3	2	0	2	

GLP1R	2	2	0	
GLP2R	2	2	0	
GLS	2	2	0	
GLYR1	2	2	0	
GMEB1	2	2	0	
GNAI2	2	0	2	
GNAT1	2	2	0	
GNG10	2	2	0	
GNG4	2	2	0	
GNRH2	2	2	0	
GOLGA5	2	0	2	
GOLPH3	2	2	0	
GON4L	2	2	0	
GOT1	2	0	2	
GP9	2	2	0	
GPBP1L1	2	0	2	
GPR126	2	0	2	
GPR137B	2	0	2	
GPR143	2	0	2	
GPR161	2	2	0	
GPR182	2	2	0	
GPR32	2	2	0	
GPR35	2	2	0	
GPR6	2	2	0	
GPSM3	2	2	0	
GRAMD3	2	0	2	
GRAP	2	2	0	
GRB14	2	0	2	
GREB1L	2	2	0	
GRIA2	2	2	0	
GRID2	2	2	0	
GRIK4	2	2	0	
GRIN1	2	2	0	
GRIN2C	2	2	0	
GRN	2	0	2	
GRPR	2	2	0	
GSPT1	2	2	0	
GSPT2	2	2	0	
GSS	2	0	2	
GSTT1	2	2	0	
GTF2H4	2	2	0	
GTPBP10	2	2	0	
GTSE1	2	0	2	
GUCA1B	2	2	0	
GULP1	2	2	0	
H2AFY	2	2	0	
H6PD	2	2	0	
HADHB	2	0	2	

HAPLN1	2	2	0	
HAT1	2	0	2	
HAUS7	2	0	2	
HAX1	2	0	2	
HBE1	2	2	0	
HBEGF	2	2	0	
HBP1	2	2	0	
HBXIP	2	2	0	
HCFC1R1	2	2	0	
HCFC2	2	2	0	
HCG26	2	2	0	
HCN2	2	2	0	
HDAC3	2	0	2	
HDAC6	2	2	0	
HDAC7	2	0	2	
HEATR2	2	2	0	
HEATR3	2	0	2	
HEBP2	2	2	0	
HECA	2	0	2	
HERC3	2	2	0	
HERC4	2	2	0	
HEXA	2	2	0	
HGF	2	2	0	
HGFAC	2	2	0	
HHLA1	2	2	0	
HIP1R	2	0	2	
HIPK3	2	2	0	
HIST1H1B	2	2	0	
HIST1H1T	2	2	0	
HIST1H2AE	2	2	0	
HIST1H2BB	2	2	0	
HIST1H2BG	2	2	0	
HIST1H2BL	2	2	0	
HIST1H2BM	2	2	0	
HIST1H2BN	2	2	0	
HIST1H2BO	2	2	0	
HIST1H3A	2	2	0	
HIST1H3B	2	2	0	
HIST1H3E	2	2	0	
HIST1H3I	2	2	0	
HIST1H4A	2	2	0	
HIST1H4F	2	2	0	
HIST2H2BE	2	2	0	
HIVEP1	2	2	0	
HKDC1	2	2	0	
HLA-A	2	0	2	
HLA-B	2	0	2	
HLA-DOA	2	2	0	

HLA-DOB	2	0	2	
HLA-DQB1	2	2	0	
HLF	2	2	0	
HMG20B	2	2	0	
HMGA1	2	2	0	
HMGCL	2	2	0	
HMGN1	2	0	2	
HMGN4	2	0	2	
HMHB1	2	2	0	
HMOX1	2	2	0	
HMX1	2	2	0	
HNF1A	2	2	0	
HNF1B	2	2	0	
HNF4A	2	2	0	
HNRNPD	2	2	0	
HNRNPF	2	2	0	
HNRNPH2	2	0	2	
HNRNPH3	2	0	2	
HOPX	2	2	0	
HOXA1	2	2	0	
HOXA11	2	2	0	
HOXA2	2	2	0	
HOXA6	2	2	0	
HOXB6	2	2	0	
HOXB9	2	2	0	
HOXC10	2	0	2	
HOXC6	2	0	2	
HOXD12	2	2	0	
HOXD3	2	2	0	
HPCAL4	2	2	0	
HPRT1	2	0	2	
HPS6	2	2	0	
HRH3	2	2	0	
HSBP1	2	0	2	
HSD11B1	2	2	0	
HSD17B10	2	0	2	
HSD17B7	2	2	0	
HSD3B1	2	2	0	
HSF1	2	2	0	
HSP90AB1	2	0	2	
HSPA12A	2	2	0	
HSPA14	2	0	2	
HSPA4	2	2	0	
HSPA5	2	0	2	
HSPB2	2	0	2	
HSPG2	2	2	0	
HTR2A	2	2	0	
HTR2B	2	2	0	

HTR2C	2	2	0	
HYOU1	2	2	0	
IARS	2	0	2	
ICMT	2	2	0	
ID4	2	2	0	
IFI27	2	0	2	
IFI30	2	0	2	
IFI44L	2	2	0	
IFITM2	2	2	0	
IFNA14	2	2	0	
IFNA17	2	2	0	
IFNGR1	2	0	2	
IGF1	2	2	0	
IGF1R	2	2	0	
IGFBP4	2	2	0	
IGFBP6	2	2	0	
IGKV1-5	2	2	0	
IGLV3-19	2	2	0	
IGLV6-57	2	2	0	
IGSF3	2	2	0	
IKBKB	2	0	2	
IKBKE	2	2	0	
IL10RB	2	2	0	
IL12B	2	2	0	
IL17RA	2	2	0	
IL1A	2	2	0	
IL1B	2	2	0	
IL1RL1	2	2	0	
IL1RN	2	2	0	
IL22	2	2	0	
IL23A	2	2	0	
IL26	2	2	0	
IL2RB	2	2	0	
IL32	2	2	0	
IL36G	2	2	0	
IL4	2	2	0	
IL5RA	2	2	0	
IL7	2	2	0	
IL9	2	2	0	
IMP4	2	0	2	
INE1	2	2	0	
ING3	2	2	0	
INHBC	2	2	0	
INPP4A	2	2	0	
INSL5	2	2	0	
INTS1	2	2	0	
INVS	2	2	0	
IPO13	2	2	0	

IPO9	2	2	0	
IQCA1	2	2	0	
IQCK	2	2	0	
IQSEC1	2	2	0	
IRF4	2	2	0	
IRF7	2	2	0	
IRS1	2	0	2	
IRX5	2	0	2	
ISG20L2	2	2	0	
ISLR	2	2	0	
ITGA10	2	0	2	
ITGA2B	2	2	0	
ITGA4	2	2	0	
ITGA6	2	0	2	
ITGA9	2	2	0	
ITGB1	2	2	0	
ITGB1BP3	2	2	0	
ITGB3BP	2	0	2	
ITGB4	2	2	0	
ITIH1	2	2	0	
ITIH3	2	2	0	
ITM2A	2	0	2	
ITM2C	2	2	0	
IVD	2	2	0	
JMJD5	2	2	0	
JPH3	2	2	0	
JRKL	2	0	2	
JUP	2	0	2	
KAT5	2	2	0	
KAT6A	2	2	0	
KATNA1	2	0	2	
KBTBD11	2	2	0	
KCNA1	2	2	0	
KCNA2	2	2	0	
KCNAB1	2	2	0	
KCND2	2	2	0	
KCND3	2	2	0	
KCNE2	2	2	0	
KCNH2	2	2	0	
KCNJ1	2	2	0	
KCNJ2	2	2	0	
KCNJ3	2	2	0	
KCNJ4	2	2	0	
KCNJ5	2	2	0	
KCNJ6	2	2	0	
KCNJ9	2	2	0	
KCNK2	2	2	0	
KCNMA1	2	2	0	

KCNMB2	2	2	0	
KCNN2	2	2	0	
KCNV1	2	2	0	
KCNV2	2	2	0	
KCTD20	2	2	0	
KDM1A	2	2	0	
KDM4C	2	2	0	
KDM5C	2	2	0	
KDM6A	2	2	0	
KDM6B	2	2	0	
KERA	2	2	0	
KHDRBS3	2	2	0	
KHSRP	2	2	0	
KIAA0100	2	0	2	
KIAA0125	2	0	2	
KIAA0195	2	2	0	
KIAA0196	2	2	0	
KIAA0240	2	0	2	
KIAA0284	2	2	0	
KIAA0586	2	0	2	
KIAA0649	2	0	2	
KIAA0922	2	0	2	
KIAA1033	2	2	0	
KIAA1107	2	2	0	
KIAA1279	2	0	2	
KIAA1614	2	2	0	
KIAA1661	2	2	0	
KIAA1731	2	2	0	
KIAA1751	2	2	0	
KIAA1967	2	2	0	
KIF15	2	0	2	
KIF20A	2	2	0	
KIF23	2	2	0	
KIF26B	2	2	0	
KIFC1	2	2	0	
KIR2DL4	2	2	0	
KIRREL	2	2	0	
KISS1	2	2	0	
KL	2	2	0	
KLHL11	2	2	0	
KLHL21	2	0	2	
KLHL24	2	2	0	
KLHL25	2	2	0	
KLHL29	2	2	0	
KLHL4	2	2	0	
KLK11	2	2	0	
KLK13	2	2	0	
KLK15	2	2	0	

KLK5	2	2	0	
KLK6	2	2	0	
KLK8	2	2	0	
KLRB1	2	0	2	
KRT38	2	2	0	
KRT76	2	2	0	
KRT83	2	2	0	
KRT85	2	2	0	
KRTAP5-9	2	2	0	
KTN1	2	0	2	
L3MBTL1	2	2	0	
LAMA1	2	2	0	
LAMA2	2	2	0	
LAMB3	2	0	2	
LAMTOR2	2	2	0	
LAP3	2	2	0	
LAPTM4A	2	0	2	
LARGE	2	2	0	
LARP1	2	0	2	
LATS1	2	2	0	
LBR	2	0	2	
LDLR	2	2	0	
LEP	2	2	0	
LEPREL2	2	2	0	
LGALS14	2	2	0	
LGI1	2	2	0	
LGR5	2	2	0	
LHCGR	2	2	0	
LHX1	2	2	0	
LIFR	2	2	0	
LILRA2	2	2	0	
LIMS3	2	2	0	
LIN37	2	0	2	
LINC00115	2	2	0	
LINC00474	2	2	0	
LIPE	2	2	0	
LMAN1	2	2	0	
LMF1	2	2	0	
LMF2	2	0	2	
LMTK2	2	2	0	
LOC100093698	2	2	0	
LOC100129361	2	0	2	
LOC100129648	2	2	0	
LOC100130741	2	2	0	
LOC100131298	2	2	0	
LOC100272216	2	0	2	
LOC100288594	2	2	0	
LOC100507397	2	2	0	

LOC100507424	2	2	0	
LOC220594	2	0	2	
LOC257152	2	2	0	
LOC388796	2	0	2	
LOC440792	2	2	0	
LOC441204	2	2	0	
LOC647070	2	2	0	
LOXL1	2	2	0	
LPAR3	2	2	0	
LPHN1	2	2	0	
LPIN1	2	2	0	
LRIG2	2	2	0	
LRP5L	2	2	0	
LRRC23	2	2	0	
LRRC36	2	2	0	
LRRC37A3	2	2	0	
LRRC48	2	2	0	
LRRC59	2	0	2	
LRRC8B	2	2	0	
LRRK1	2	2	0	
LRRTM2	2	2	0	
LSM3	2	0	2	
LSP1	2	2	0	
LST1	2	2	0	
LTBP3	2	0	2	
LTC4S	2	2	0	
LTN1	2	2	0	
LY6G6C	2	2	0	
LY75	2	0	2	
LY96	2	2	0	
LYN	2	0	2	
LYRM2	2	0	2	
LZTS1	2	2	0	
MAF	2	2	0	
MAFG	2	0	2	
MAG	2	2	0	
MAGEA4	2	2	0	
MAGEB1	2	2	0	
MAGEB4	2	2	0	
MAGEC2	2	2	0	
MAGEC3	2	2	0	
MAGEL2	2	2	0	
MAGOH2	2	2	0	
MAK	2	2	0	
MAN1C1	2	2	0	
MANEA	2	2	0	
MAP2K6	2	2	0	
MAP2K7	2	2	0	

MAP3K13	2	2	0	
MAP3K14	2	2	0	
MAP3K3	2	2	0	
MAP4	2	0	2	
MAP4K2	2	2	0	
MAP4K3	2	0	2	
MAP4K4	2	0	2	
MAP6D1	2	0	2	
MAP7D3	2	0	2	
MAPK1	2	2	0	
MAPK10	2	2	0	
MAPK11	2	2	0	
MAPK14	2	2	0	
MAPK3	2	2	0	
MAPK4	2	2	0	
MAPKAPK5	2	0	2	
MAPRE2	2	2	0	
MAPRE3	2	2	0	
MARK2	2	2	0	
MASP2	2	2	0	
MAT2A	2	0	2	
MATN2	2	0	2	
MATN4	2	2	0	
MBD2	2	2	0	
MBIP	2	2	0	
MBNL3	2	2	0	
MBOAT7	2	2	0	
MBTD1	2	2	0	
MBTPS2	2	2	0	
MC5R	2	2	0	
MCAM	2	2	0	
MCF2L-AS1	2	2	0	
MCHR1	2	2	0	
MCM2	2	2	0	
MCM3AP	2	2	0	
MCM4	2	2	0	
MCM5	2	2	0	
MCM6	2	2	0	
MCM7	2	2	0	
MDH1	2	0	2	
MEAF6	2	2	0	
MECP2	2	2	0	
MED14	2	2	0	
MED25	2	2	0	
MED7	2	0	2	
MEFV	2	2	0	
MEGF6	2	2	0	
MEGF8	2	2	0	

MEIS2	2	2	0	
MEOX1	2	0	2	
MEP1B	2	2	0	
METTL21D	2	0	2	
MEX3D	2	2	0	
MFGE8	2	0	2	
MFNG	2	2	0	
MGAT4A	2	2	0	
MGAT4B	2	0	2	
MGC2752	2	2	0	
MGST3	2	0	2	
MICAL1	2	0	2	
MINA	2	0	2	
MIOS	2	2	0	
MIR600HG	2	2	0	
MIR622	2	2	0	
MIS18A	2	0	2	
MKI67	2	2	0	
MKL1	2	2	0	
MLF1IP	2	2	0	
MLF2	2	2	0	
MLL2	2	2	0	
MLLT1	2	2	0	
MLLT10	2	2	0	
MLLT4	2	2	0	
MLN	2	2	0	
MLX	2	0	2	
MMACHC	2	0	2	
MMP11	2	2	0	
MNT	2	2	0	
MOAP1	2	0	2	
MORN1	2	2	0	
MOSC1	2	2	0	
MOSPD3	2	2	0	
MPHOSPH8	2	0	2	
MPP2	2	2	0	
MPPE1	2	0	2	
MRPL19	2	0	2	
MRPL34	2	0	2	
MRPL40	2	0	2	
MRPL48	2	2	0	
MRPL49	2	0	2	
MRPS12	2	2	0	
MRPS14	2	2	0	
MS4A5	2	2	0	
MSH6	2	2	0	
MSR1	2	2	0	
MSTN	2	2	0	

MT3	2	0	2	
MT4	2	2	0	
MTAP	2	2	0	
MTF1	2	2	0	
MTHFR	2	2	0	
MTHFS	2	0	2	
MTL5	2	2	0	
MTMR9	2	2	0	
MTPAP	2	2	0	
MTUS2	2	2	0	
MUC8	2	2	0	
MUDENG	2	0	2	
MUL1	2	2	0	
MVK	2	2	0	
MYBL2	2	2	0	
MYBPC2	2	2	0	
MYCBP2	2	0	2	
MYL1	2	2	0	
MYL12A	2	0	2	
MYLK	2	0	2	
MYLK3	2	0	2	
MYNN	2	0	2	
MYO10	2	0	2	
MYO15A	2	2	0	
MYO5A	2	2	0	
MYO7A	2	2	0	
MYOF	2	2	0	
MYT1	2	2	0	
N4BP2L1	2	2	0	
N4BP2L2-IT1	2	2	0	
NAALAD2	2	2	0	
NAB2	2	2	0	
NAG18	2	2	0	
NAGK	2	0	2	
NAGPA	2	2	0	
NASP	2	2	0	
NAT1	2	0	2	
NAV2	2	0	2	
NAV3	2	2	0	
NBEAL2	2	2	0	
NCAPH2	2	2	0	
NCF1C	2	2	0	
NCKAP1L	2	2	0	
NCOA2	2	2	0	
NCOR2	2	2	0	
NDST1	2	2	0	
NDUFA5	2	2	0	
NDUFAF1	2	0	2	

NDUFB7	2	2	0	
NDUFS6	2	2	0	
NECAB3	2	2	0	
NECAP1	2	2	0	
NEFH	2	2	0	
NEU1	2	0	2	
NEUROG2	2	2	0	
NEUROG3	2	2	0	
NF1	2	2	0	
NFATC3	2	2	0	
NFE2	2	2	0	
NFKB1	2	0	2	
NFKB2	2	2	0	
NFRKB	2	2	0	
NFU1	2	0	2	
NGB	2	2	0	
NHLH2	2	2	0	
NID1	2	2	0	
NIPAL2	2	2	0	
NISCH	2	0	2	
NKX3-2	2	2	0	
NLRP1	2	2	0	
NLRP3	2	2	0	
NLRX1	2	2	0	
NMB	2	0	2	
NME6	2	2	0	
NMNAT2	2	2	0	
NOD1	2	2	0	
NOL7	2	0	2	
NOP14	2	0	2	
NOP2	2	0	2	
NOTCH3	2	2	0	
NOV	2	2	0	
NPAS3	2	2	0	
NPHS1	2	2	0	
NPIP	2	0	2	
NPM1	2	0	2	
NPPA	2	2	0	
NPR3	2	0	2	
NPY2R	2	2	0	
NR0B1	2	2	0	
NR0B2	2	2	0	
NR1H2	2	2	0	
NR2E3	2	2	0	
NR2F2	2	2	0	
NR5A1	2	2	0	
NR6A1	2	2	0	
NRP1	2	2	0	

NRXN2	2	2	0	
NRXN3	2	2	0	
NSA2	2	0	2	
NSFL1C	2	2	0	
NSUN5P2	2	0	2	
NTRK2	2	0	2	
NTSR2	2	2	0	
NUAK1	2	2	0	
NUDT1	2	0	2	
NUDT11	2	2	0	
NUDT9	2	0	2	
NUFIP1	2	2	0	
NUP210	2	0	2	
NUP37	2	0	2	
NUP43	2	0	2	
NUP54	2	0	2	
NUP62	2	0	2	
NUPL2	2	0	2	
NUS1P3	2	2	0	
NUSAP1	2	2	0	
OCLN	2	2	0	
OCM2	2	2	0	
ODF1	2	0	2	
ODZ3	2	2	0	
ODZ4	2	2	0	
OGG1	2	2	0	
OGN	2	2	0	
OGT	2	2	0	
OLFM1	2	2	0	
OMP	2	2	0	
OPN1SW	2	2	0	
OPRD1	2	2	0	
OPRL1	2	2	0	
OPRM1	2	2	0	
OR1D2	2	2	0	
OR1F1	2	2	0	
OR1F2P	2	2	0	
OR2B6	2	2	0	
OR2C1	2	2	0	
OR3A2	2	2	0	
OR51E2	2	2	0	
OR7E19P	2	2	0	
OR7E24	2	2	0	
ORC1	2	2	0	
OSBPL8	2	0	2	
OSM	2	2	0	
OTC	2	2	0	
OTOF	2	2	0	

OTUD7B	2	2	0	
OVOL1	2	2	0	
OVOL2	2	2	0	
P2RX1	2	2	0	
P2RX2	2	2	0	
P2RY14	2	2	0	
PACSIN2	2	0	2	
PADI1	2	2	0	
PADI4	2	2	0	
PAFAH1B2	2	2	0	
PAGE1	2	2	0	
PALM	2	2	0	
PAMR1	2	0	2	
PARG	2	0	2	
PARP1	2	2	0	
PARP3	2	2	0	
PART1	2	2	0	
PARVB	2	2	0	
PAX1	2	2	0	
PBLD	2	0	2	
PCBD1	2	2	0	
PCBP1	2	0	2	
PCCA	2	2	0	
PCDHA10	2	2	0	
PCDHB8	2	2	0	
PCDHGA8	2	2	0	
PCDHGA9	2	2	0	
PCDHGC3	2	2	0	
PCGF2	2	2	0	
PCNT	2	2	0	
PCNXL2	2	2	0	
PCSK1	2	2	0	
PCSK7	2	0	2	
PCYOX1	2	0	2	
PCYOX1L	2	0	2	
PCYT1A	2	2	0	
PCYT1B	2	2	0	
PDCD1LG2	2	2	0	
PDE2A	2	0	2	
PDE4B	2	2	0	
PDE6B	2	2	0	
PDGFRB	2	2	0	
PDHB	2	0	2	
PDHX	2	2	0	
PDIA3	2	2	0	
PDIA6	2	2	0	
PDPK1	2	2	0	
PDYN	2	2	0	

PEX12	2	0	2	
PEX14	2	0	2	
PEX2	2	2	0	
PEX5L	2	2	0	
PFDN6	2	2	0	
PGCP	2	2	0	
PGF	2	2	0	
PHC2	2	2	0	
PHEX	2	2	0	
PHF20	2	2	0	
PHF3	2	2	0	
PHIP	2	2	0	
PHLDA1	2	2	0	
PHLDB1	2	2	0	
PHLPP1	2	2	0	
PHLPP2	2	2	0	
PI4K2A	2	2	0	
PIAS2	2	2	0	
PIGG	2	0	2	
PIGZ	2	2	0	
PIK3CD	2	2	0	
PIK3R2	2	2	0	
PIM1	2	0	2	
PIN1P1	2	2	0	
PION	2	2	0	
PIP5K1B	2	2	0	
PITX3	2	2	0	
PKD1P1	2	0	2	
PKDREJ	2	2	0	
PKI55	2	2	0	
PKNOX2	2	2	0	
PLA2G10	2	2	0	
PLA2G16	2	0	2	
PLA2G2E	2	2	0	
PLA2G3	2	2	0	
PLA2G4C	2	0	2	
PLAC1	2	2	0	
PLAG1	2	2	0	
PLCB1	2	2	0	
PLCB2	2	2	0	
PLCE1	2	2	0	
PLCG2	2	0	2	
PLD1	2	2	0	
PLEKHA1	2	0	2	
PLEKHB2	2	0	2	
PLEKHF2	2	0	2	
PLEKHM1	2	2	0	
PLIN2	2	2	0	

PLK4	2	2	0	
PLOD3	2	0	2	
PLP1	2	0	2	
PLXDC1	2	2	0	
PLXNB2	2	2	0	
PMAIP1	2	2	0	
PMF1	2	2	0	
PMM2	2	2	0	
PNLIPRP1	2	2	0	
PNMT	2	2	0	
PNN	2	0	2	
PODXL2	2	2	0	
POFUT1	2	2	0	
POFUT2	2	2	0	
POLDIP3	2	2	0	
POLH	2	2	0	
POLL	2	2	0	
POLR1C	2	0	2	
POLR3C	2	2	0	
POLR3E	2	0	2	
POLR3K	2	2	0	
POLRMT	2	2	0	
POM121	2	2	0	
POM121L2	2	2	0	
POP7	2	2	0	
PORCN	2	2	0	
POU3F1	2	2	0	
POU6F2	2	2	0	
PPAP2A	2	2	0	
PPFIA2	2	2	0	
PPIB	2	2	0	
PPIE	2	0	2	
PPIF	2	0	2	
PPP1CA	2	0	2	
PPP1R12B	2	2	0	
PPP1R13B	2	2	0	
PPP1R13L	2	2	0	
PPP1R14B	2	2	0	
PPP1R2P9	2	2	0	
PPP2R1B	2	2	0	
PPP3CB	2	2	0	
PPP4R1	2	2	0	
PPP5C	2	2	0	
PPPDE1	2	2	0	
PPT2	2	0	2	
PPY	2	2	0	
PQLC1	2	0	2	
PRDM12	2	2	0	

PRDM2	2	2	0	
PRDM5	2	2	0	
PRDX2	2	2	0	
PRG3	2	2	0	
PRKAB1	2	2	0	
PRKACG	2	2	0	
PRKCB	2	0	2	
PRKCG	2	2	0	
PRKCH	2	0	2	
PRKG1	2	2	0	
PRKX	2	2	0	
PRL	2	2	0	
PRLH	2	2	0	
PRMT3	2	0	2	
PROC	2	2	0	
PRODH2	2	2	0	
PRPF19	2	2	0	
PRPF4	2	0	2	
PRPF40A	2	0	2	
PRPH2	2	2	0	
PRR5	2	2	0	
PRRX1	2	2	0	
PRSS1	2	0	2	
PRSS21	2	2	0	
PRSS23	2	0	2	
PRSS8	2	2	0	
PRX	2	2	0	
PSAT1	2	0	2	
PSD	2	2	0	
PSG1	2	2	0	
PSG2	2	2	0	
PSG3	2	2	0	
PSG4	2	2	0	
PSG5	2	2	0	
PSMD12	2	0	2	
PSMD5	2	2	0	
PSME2	2	0	2	
PSMF1	2	2	0	
PSMG1	2	0	2	
PSMG2	2	0	2	
PTCD1	2	2	0	
PTEN	2	2	0	
PTENP1	2	2	0	
PTGER1	2	2	0	
PTGER4	2	2	0	
PTGES2	2	2	0	
PTGES3	2	0	2	
PTGIS	2	2	0	

PTK2B	2	2	0	
PTK7	2	2	0	
PTP4A2	2	0	2	
PTPN14	2	2	0	
PTPRCAP	2	2	0	
PTPRK	2	2	0	
PTPRM	2	2	0	
PTS	2	0	2	
PUF60	2	2	0	
PUM1	2	2	0	
PUS1	2	0	2	
PVR	2	2	0	
PVRL1	2	2	0	
PVRL3	2	2	0	
PXDN	2	2	0	
PYCARD	2	2	0	
PYCR1	2	0	2	
PYGL	2	2	0	
PZP	2	2	0	
QPRT	2	0	2	
QSER1	2	2	0	
QTRT1	2	2	0	
R3HDM2	2	0	2	
RAB11B	2	2	0	
RAB11FIP1	2	0	2	
RAB11FIP2	2	0	2	
RAB11FIP5	2	0	2	
RAB15	2	0	2	
RAB17	2	0	2	
RAB1A	2	0	2	
RAB21	2	2	0	
RAB22A	2	0	2	
RAB23	2	2	0	
RAB25	2	0	2	
RAB26	2	2	0	
RAB38	2	2	0	
RAB3A	2	2	0	
RAB40A	2	2	0	
RAB40C	2	2	0	
RAB5A	2	2	0	
RAB8A	2	2	0	
RABEP2	2	0	2	
RABEPK	2	0	2	
RABGGTA	2	2	0	
RAC1	2	0	2	
RAC2	2	0	2	
RAD51B	2	2	0	
RAD52	2	2	0	

RAE1	2	0	2	
RAI1	2	2	0	
RAI14	2	2	0	
RALB	2	0	2	
RALBP1	2	0	2	
RALGPS2	2	2	0	
RAMP2	2	2	0	
RAP1A	2	2	0	
RAP1GAP2	2	0	2	
RAP2A	2	2	0	
RAP2B	2	2	0	
RAPGEF5	2	2	0	
RASAL1	2	2	0	
RASGRF1	2	2	0	
RASL10A	2	2	0	
RASSF2	2	0	2	
RASSF7	2	2	0	
RB1	2	2	0	
RBBP8	2	0	2	
RBBP9	2	2	0	
RBCK1	2	0	2	
RBKS	2	0	2	
RBM10	2	0	2	
RBM28	2	0	2	
RBM3	2	2	0	
RBM47	2	2	0	
RBM6	2	0	2	
RBP4	2	2	0	
REC8	2	2	0	
RECK	2	2	0	
REPIN1	2	2	0	
RERGL	2	2	0	
RFC4	2	0	2	
RFNG	2	2	0	
RFPL1-AS1	2	2	0	
RFX1	2	2	0	
RFX5	2	0	2	
RGP1	2	2	0	
RGS1	2	2	0	
RGS12	2	2	0	
RGS14	2	2	0	
RGS5	2	2	0	
RGS9	2	2	0	
RHBDF1	2	2	0	
RHBDF2	2	2	0	
RHOBTB3	2	2	0	
RIC3	2	2	0	
RIC8B	2	0	2	

RIMBP2	2	2	0	
RIMS1	2	2	0	
RLBP1	2	2	0	
RLN1	2	2	0	
RMI1	2	0	2	
RNASEH2B	2	2	0	
RNF11	2	0	2	
RNF115	2	2	0	
RNF141	2	2	0	
RNF185	2	2	0	
RNF186	2	2	0	
RNF187	2	0	2	
RNF208	2	2	0	
RNF220	2	0	2	
RNF44	2	0	2	
RNPS1	2	2	0	
ROCK1	2	0	2	
ROPN1B	2	2	0	
RPA3	2	0	2	
RPAP2	2	2	0	
RPF1	2	0	2	
RPL12	2	0	2	
RPL13A	2	0	2	
RPL13P5	2	2	0	
RPL14	2	0	2	
RPL17	2	0	2	
RPL18	2	0	2	
RPL19	2	0	2	
RPL21	2	0	2	
RPL23A	2	0	2	
RPL27	2	0	2	
RPL27A	2	0	2	
RPL29	2	0	2	
RPL3	2	0	2	
RPL30	2	0	2	
RPL35	2	0	2	
RPL36A	2	0	2	
RPL37	2	0	2	
RPL37A	2	0	2	
RPL6	2	0	2	
RPL7A	2	0	2	
RPL9	2	0	2	
RPLP0	2	0	2	
RPLP1	2	0	2	
RPRD2	2	2	0	
RPS10	2	0	2	
RPS12	2	0	2	
RPS13	2	0	2	

RPS16	2	0	2	
RPS17	2	0	2	
RPS2	2	0	2	
RPS20	2	0	2	
RPS24	2	0	2	
RPS27A	2	0	2	
RPS29	2	0	2	
RPS3	2	0	2	
RPS4X	2	0	2	
RPS6KC1	2	0	2	
RPS7	2	0	2	
RPSA	2	0	2	
RRH	2	2	0	
RRM2	2	2	0	
RRNAD1	2	0	2	
RRP15	2	0	2	
RSAD1	2	0	2	
RSL24D1	2	0	2	
RSU1	2	2	0	
RTEL1	2	2	0	
RUNX1	2	2	0	
RUNX1T1	2	2	0	
RUNX2	2	2	0	
RUNX3	2	0	2	
RUVBL1	2	0	2	
RWDD2B	2	0	2	
RYR2	2	2	0	
S100A6	2	2	0	
S100A8	2	2	0	
S100G	2	2	0	
SAMD4A	2	2	0	
SAMSN1	2	2	0	
SAP30BP	2	0	2	
SARDH	2	2	0	
SASH1	2	0	2	
SAT1	2	2	0	
SC5DL	2	2	0	
SCAPER	2	2	0	
SCARF1	2	2	0	
SCIN	2	2	0	
SCML2	2	0	2	
SCN3B	2	2	0	
SCN8A	2	2	0	
SCNN1G	2	2	0	
SCRG1	2	2	0	
SCRN1	2	2	0	
SCTR	2	2	0	
SCUBE2	2	0	2	

SDC1	2	2	0	
SDCBP	2	2	0	
SDCCAG3	2	0	2	
SDF2L1	2	2	0	
SDF4	2	0	2	
SDHA	2	2	0	
SEC14L1	2	2	0	
SEC61G	2	0	2	
SECISBP2	2	0	2	
SEL1L3	2	0	2	
SELENBP1	2	2	0	
SELL	2	0	2	
SEMA3A	2	2	0	
SEMA4D	2	2	0	
SEMG1	2	2	0	
SENP3	2	2	0	
SERINC3	2	0	2	
SERPINA1	2	2	0	
SERPINB13	2	2	0	
SERPINB6	2	0	2	
SERPINB8	2	2	0	
SERPINB9	2	2	0	
SERPINI2	2	2	0	
SERTAD3	2	2	0	
SET	2	0	2	
SETX	2	0	2	
SF3A3	2	0	2	
SF3B3	2	2	0	
SH2D1A	2	2	0	
SH2D2A	2	2	0	
SH2D3C	2	2	0	
SH3BGR	2	2	0	
SH3BP1	2	0	2	
SH3GL1	2	2	0	
SH3GL3	2	2	0	
SH3TC2	2	2	0	
SH3YL1	2	0	2	
SHB	2	2	0	
SHMT2	2	0	2	
SHOX2	2	2	0	
SIGIRR	2	0	2	
SIGLEC1	2	2	0	
SIGLEC15	2	0	2	
SIGLEC7	2	2	0	
SIRT4	2	2	0	
SIX3	2	2	0	
SKIV2L	2	0	2	
SLC11A1	2	2	0	

SLC12A4	2	2	0	
SLC12A5	2	2	0	
SLC13A4	2	2	0	
SLC16A8	2	2	0	
SLC17A2	2	2	0	
SLC17A3	2	2	0	
SLC17A5	2	0	2	
SLC19A1	2	2	0	
SLC19A3	2	2	0	
SLC1A3	2	2	0	
SLC22A18	2	0	2	
SLC22A18AS	2	2	0	
SLC22A6	2	2	0	
SLC23A2	2	2	0	
SLC25A1	2	2	0	
SLC25A13	2	0	2	
SLC25A15	2	0	2	
SLC25A16	2	2	0	
SLC25A23	2	2	0	
SLC25A28	2	0	2	
SLC26A3	2	2	0	
SLC27A2	2	0	2	
SLC27A5	2	2	0	
SLC28A2	2	2	0	
SLC2A4	2	2	0	
SLC30A1	2	0	2	
SLC30A9	2	0	2	
SLC35A3	2	0	2	
SLC38A1	2	2	0	
SLC39A2	2	2	0	
SLC39A6	2	0	2	
SLC3A1	2	2	0	
SLC44A1	2	0	2	
SLC48A1	2	0	2	
SLC5A1	2	2	0	
SLC5A2	2	2	0	
SLC6A7	2	2	0	
SLC7A1	2	2	0	
SLC7A4	2	2	0	
SLC7A9	2	2	0	
SLC9A2	2	2	0	
SLC9A3R2	2	2	0	
SLCO1C1	2	2	0	
SLCO2A1	2	2	0	
SLIT3	2	2	0	
SLK	2	0	2	
SLPI	2	0	2	
SLURP1	2	2	0	

SMAD4	2	2	0	
SMARCA1	2	2	0	
SMARCC2	2	0	2	
SMARCD2	2	0	2	
SMARCD3	2	2	0	
SMG9	2	2	0	
SMURF1	2	2	0	
SND1	2	2	0	
SNRK	2	2	0	
SNRNP25	2	2	0	
SNRPA1	2	0	2	
SNTB2	2	2	0	
SNUPN	2	0	2	
SNX11	2	0	2	
SNX3	2	0	2	
SNX4	2	0	2	
SNX6	2	0	2	
SOAT2	2	2	0	
SOCS3	2	2	0	
SOCS7	2	2	0	
SOD1	2	0	2	
SOLH	2	2	0	
SORBS3	2	0	2	
SORCS3	2	2	0	
SOS2	2	2	0	
SOX1	2	2	0	
SOX14	2	2	0	
SOX15	2	2	0	
SOX18	2	2	0	
SOX2	2	2	0	
SOX21	2	2	0	
SOX3	2	2	0	
SOX5	2	2	0	
SP1	2	2	0	
SP110	2	0	2	
SPAG6	2	2	0	
SPAG8	2	2	0	
SPCS3	2	2	0	
SPEG	2	0	2	
SPHK2	2	2	0	
SPI1	2	2	0	
SPINK2	2	2	0	
SPINK5	2	2	0	
SPINLW1	2	2	0	
SPN	2	2	0	
SPP1	2	0	2	
SPPL2B	2	2	0	
SPR	2	2	0	

SPRY2	2	0	2	
SPTB	2	2	0	
SPTBN2	2	2	0	
SPTLC1	2	0	2	
SRCAP	2	2	0	
SREBF2	2	0	2	
SRF	2	2	0	
SRP54	2	0	2	
SRPX2	2	2	0	
SSX2IP	2	2	0	
SSX3	2	2	0	
ST18	2	2	0	
ST8SIA2	2	2	0	
ST8SIA4	2	2	0	
STAB2	2	2	0	
STAG1	2	2	0	
STAT1	2	0	2	
STK25	2	0	2	
STK4	2	2	0	
STRN	2	2	0	
STX12	2	0	2	
STX8	2	2	0	
STXBP1	2	0	2	
STXBP6	2	2	0	
STYK1	2	2	0	
SULF1	2	2	0	
SULT1E1	2	2	0	
SUPT16H	2	0	2	
SUPT6H	2	0	2	
SV2A	2	2	0	
SVEP1	2	2	0	
SYCP1	2	2	0	
SYMPK	2	0	2	
SYNCRIP	2	0	2	
SYNE1	2	2	0	
SYNE2	2	0	2	
SYNJ2BP	2	2	0	
SYNPO2L	2	2	0	
SYT5	2	2	0	
TACR1	2	2	0	
TAF1	2	2	0	
TAF12	2	0	2	
TAF15	2	2	0	
TAF1D	2	0	2	
TAF2	2	0	2	
TAF4	2	2	0	
TAF6	2	2	0	
TAF9B	2	2	0	

TAGLN3	2	2	0	
TALDO1	2	0	2	
TANC2	2	2	0	
TAPBPL	2	2	0	
TAS2R10	2	2	0	
TBC1D12	2	2	0	
TBCD	2	2	0	
TBX10	2	2	0	
TCEA1	2	0	2	
TCEAL4	2	0	2	
TCEB3B	2	2	0	
TCF12	2	2	0	
TCF7L2	2	2	0	
TCL6	2	2	0	
TCP11	2	2	0	
TCTN1	2	2	0	
TDO2	2	2	0	
TEKT2	2	2	0	
TESC	2	2	0	
TEX2	2	0	2	
TFAP2B	2	2	0	
TFB1M	2	0	2	
TFF1	2	0	2	
TFRC	2	0	2	
TGFB3	2	2	0	
TGFBR1	2	2	0	
TGIF2	2	2	0	
TGOLN2	2	2	0	
THAP3	2	2	0	
THBS2	2	2	0	
THOP1	2	2	0	
THUMPD2	2	2	0	
THY1	2	2	0	
TIMM17A	2	2	0	
TIMM17B	2	0	2	
TIMM22	2	2	0	
TIMM8A	2	2	0	
TINF2	2	0	2	
TJP1	2	0	2	
TJP2	2	0	2	
TK1	2	2	0	
TLE3	2	0	2	
TLE6	2	0	2	
TLK2	2	2	0	
TM2D3	2	0	2	
TM4SF1	2	2	0	
TM4SF5	2	2	0	
TMBIM6	2	0	2	

TMC5	2	2	0	
TMC7	2	2	0	
TMCC2	2	0	2	
TMED1	2	2	0	
TMED7	2	0	2	
TMEM104	2	0	2	
TMEM127	2	0	2	
TMEM132A	2	2	0	
TMEM135	2	2	0	
TMEM140	2	0	2	
TMEM143	2	2	0	
TMEM144	2	2	0	
TMEM147	2	0	2	
TMEM156	2	2	0	
TMEM158	2	2	0	
TMEM176A	2	2	0	
TMEM176B	2	2	0	
TMEM19	2	2	0	
TMEM22	2	2	0	
TMEM33	2	0	2	
TMEM40	2	2	0	
TMEM45A	2	0	2	
TMEM47	2	0	2	
TMEM87A	2	0	2	
TMPO	2	2	0	
TMPRSS6	2	2	0	
TMX2	2	0	2	
TMX4	2	2	0	
TNF	2	2	0	
TNFAIP8	2	2	0	
TNFRSF4	2	2	0	
TNFRSF9	2	2	0	
TNFSF11	2	2	0	
TNFSF15	2	2	0	
TNFSF4	2	2	0	
TNNI3K	2	2	0	
TNPO1	2	0	2	
TNPO3	2	0	2	
TNXB	2	2	0	
TOMM70A	2	0	2	
TONSL	2	2	0	
TOPBP1	2	0	2	
TOX	2	2	0	
TP53TG1	2	0	2	
TP63	2	2	0	
TP73-AS1	2	2	0	
TPD52L1	2	2	0	
TPK1	2	0	2	

TPM1	2	0	2	
TPSAB1	2	2	0	
TPST2	2	2	0	
TPTE	2	2	0	
TRABD	2	2	0	
TRAF3IP3	2	2	0	
TRAP1	2	0	2	
TRD@	2	2	0	
TREM2	2	2	0	
TREML2	2	2	0	
TRIL	2	2	0	
TRIM15	2	2	0	
TRIM23	2	2	0	
TRIM24	2	2	0	
TRIM3	2	2	0	
TRIM44	2	0	2	
TRIM5	2	2	0	
TRIM68	2	2	0	
TRIOBP	2	2	0	
TRIP10	2	0	2	
TRMT1	2	2	0	
TRMT1L	2	0	2	
TRPC1	2	2	0	
TRPC7	2	2	0	
TRPM1	2	2	0	
TRPM3	2	2	0	
TRPS1	2	0	2	
TSC2	2	2	0	
TSC22D3	2	2	0	
TSEN34	2	2	0	
TSHR	2	2	0	
TSHZ2	2	2	0	
TSKS	2	2	0	
TSKU	2	2	0	
TSPAN3	2	0	2	
TSPAN5	2	0	2	
TSPO	2	0	2	
TSPYL1	2	0	2	
TSPYL2	2	0	2	
TSPYL4	2	0	2	
TSSC1	2	2	0	
TTBK2	2	2	0	
TTC12	2	2	0	
TTC18	2	2	0	
TTC22	2	2	0	
TTC28	2	2	0	
TTC37	2	0	2	
TTLL5	2	2	0	

TULP2	2	2	0	
TWF1	2	2	0	
TXLNA	2	0	2	
UBA52	2	0	2	
UBA7	2	2	0	
UBAC1	2	0	2	
UBE2D1	2	2	0	
UBE2D2	2	0	2	
UBE2V2	2	0	2	
UBL4A	2	0	2	
UBL5	2	0	2	
UBN1	2	2	0	
UBOX5	2	2	0	
UBQLN2	2	0	2	
UBR4	2	2	0	
UBXN2B	2	0	2	
UCK2	2	2	0	
UCP1	2	2	0	
UGDH	2	2	0	
UNC5C	2	2	0	
UNC93A	2	2	0	
UPF3B	2	0	2	
UPK3B	2	0	2	
UQCR11	2	0	2	
URB1	2	2	0	
URB2	2	0	2	
USP27X	2	2	0	
USP29	2	2	0	
USP32	2	2	0	
USP6NL	2	0	2	
USP7	2	2	0	
UTP14C	2	0	2	
UTP20	2	2	0	
UTP6	2	0	2	
UTS2	2	2	0	
VAC14	2	2	0	
VAMP2	2	2	0	
VAMP7	2	0	2	
VANGL1	2	0	2	
VAV2	2	2	0	
VBP1	2	0	2	
VEGFC	2	2	0	
VHL	2	2	0	
VIM	2	0	2	
VIPR2	2	2	0	
VPS13B	2	2	0	
VPS28	2	2	0	
VPS37C	2	2	0	

VPS53	2	2	0	
VSTM4	2	2	0	
VSX1	2	2	0	
WARS2	2	2	0	
WASF2	2	2	0	
WDR11	2	0	2	
WDR12	2	0	2	
WDR3	2	0	2	
WDR4	2	0	2	
WDR61	2	0	2	
WDR7	2	2	0	
WFDC2	2	2	0	
WIF1	2	2	0	
WNK1	2	2	0	
WNT1	2	2	0	
WNT2B	2	2	0	
WNT5A	2	0	2	
WNT7A	2	2	0	
WRNIP1	2	2	0	
WT1	2	2	0	
WWC3	2	2	0	
XAB2	2	2	0	
XPNPEP3	2	2	0	
XPO4	2	2	0	
XPOT	2	0	2	
YEATS4	2	0	2	
YES1	2	2	0	
YKT6	2	0	2	
YTHDC2	2	2	0	
YTHDF1	2	0	2	
YWHAZ	2	2	0	
ZBBX	2	2	0	
ZBED2	2	0	2	
ZBTB1	2	2	0	
ZBTB16	2	0	2	
ZBTB17	2	2	0	
ZBTB40	2	2	0	
ZBTB44	2	2	0	
ZBTB6	2	2	0	
ZBTB7A	2	2	0	
ZC3H15	2	2	0	
ZC3H7B	2	2	0	
ZCCHC10	2	0	2	
ZCCHC11	2	2	0	
ZCCHC24	2	2	0	
ZCWPW1	2	2	0	
ZDHHC7	2	0	2	
ZEB1	2	2	0	

ZER1	2	2	0	
ZFHX3	2	2	0	
ZMAT3	2	0	2	
ZMYM3	2	2	0	
ZMYND10	2	2	0	
ZNF10	2	2	0	
ZNF137P	2	2	0	
ZNF14	2	0	2	
ZNF141	2	2	0	
ZNF175	2	0	2	
ZNF177	2	2	0	
ZNF180	2	2	0	
ZNF204P	2	2	0	
ZNF219	2	0	2	
ZNF223	2	2	0	
ZNF224	2	2	0	
ZNF259	2	0	2	
ZNF259P1	2	2	0	
ZNF273	2	2	0	
ZNF280A	2	2	0	
ZNF280B	2	2	0	
ZNF287	2	2	0	
ZNF292	2	2	0	
ZNF329	2	2	0	
ZNF335	2	2	0	
ZNF365	2	2	0	
ZNF44	2	2	0	
ZNF442	2	2	0	
ZNF460	2	2	0	
ZNF484	2	2	0	
ZNF518A	2	2	0	
ZNF536	2	2	0	
ZNF562	2	0	2	
ZNF614	2	2	0	
ZNF638	2	0	2	
ZNF643	2	2	0	
ZNF654	2	2	0	
ZNF667	2	2	0	
ZNF672	2	2	0	
ZNF682	2	2	0	
ZNF721	2	0	2	
ZNF749	2	2	0	
ZNF750	2	2	0	
ZNF79	2	2	0	
ZNF814	2	2	0	
ZNF816	2	2	0	
ZNF84	2	0	2	
ZNHIT6	2	0	2	

ZP2	2	2	0	
ZSCAN5A	2	2	0	
ZXDB	2	2	0	
ZZZ3	2	2	0	

Complex 3

mRNA based prediction result

Pa	Pi	DownRegulation
0.739	0.052	TRAF5
0.656	0.037	CYP11A1
0.677	0.107	TPM1
0.629	0.109	ADORA2B
0.629	0.109	CDCA4
0.629	0.109	CENPJ
0.629	0.109	DNAJC9
0.629	0.109	DONSON
0.629	0.109	HAUS8
0.629	0.109	MCMBP
0.629	0.109	NUP155
0.629	0.109	PARP2
0.629	0.109	RMI1
0.629	0.109	SCARA3
0.629	0.109	SLC16A14
0.629	0.109	SUV39H2
0.606	0.124	GSS
0.595	0.131	RPRM
0.564	0.116	CXCR7
0.543	0.107	ABCC5
0.536	0.115	TNIK
0.546	0.139	MFAP4
0.522	0.118	TBL1XR1
0.543	0.162	PLEC
0.504	0.139	NSF
0.537	0.172	ERCC2
0.534	0.193	CXCL2
0.507	0.191	CAPRIN1
0.538	0.226	TBC1D9

Pa	Pi	UpRegulation
0.935	0.006	PGR
0.932	0.008	TFF1
0.774	0.006	CYP19A1
0.711	0.003	RARG
0.670	0.084	SFRP1
0.587	0.005	CYP26A1
0.571	0.030	COX5B
0.614	0.089	KRT6A
0.628	0.106	UGT2B15
0.629	0.109	PFKFB2
0.629	0.109	SH3BGRL
0.629	0.109	TRIM13
0.615	0.095	SQLE
0.611	0.140	GFRA1
0.516	0.059	PSMD6
0.548	0.092	CALM1
0.566	0.129	ST3GAL1
0.542	0.125	CYTH1
0.529	0.137	CACNG4
0.529	0.137	CHRNE
0.519	0.128	PTGER4
0.546	0.157	ALDOA
0.537	0.163	HIVEP1
0.505	0.152	FADS1
0.502	0.154	EXOSC4

Protein based prediction result

Pa	Pi	DownRegulation
0.769	0.005	IFNG
0.519	0.145	VIM
0.528	0.186	PPARA

Pa	Pi	UpRegulation
0.807	0.004	CYP3A4
0.804	0.005	CYP3A7
0.758	0.003	CYP2B6
0.561	0.122	CD83

MCF7 based prediction result

Pa	Pi	DownRegulation
0.621	0.026	BBS4

Pa	Pi	UpRegulation
0.687	0.025	STRN

0.589	0.037	MRPL34	0.694	0.035	CDKN1B
0.620	0.088	FOXM1	0.699	0.042	C11orf68
0.536	0.046	ACVR2B	0.667	0.051	ZNF672
0.536	0.046	BID	0.651	0.119	CLDN9
0.536	0.046	ERGIC2	0.617	0.087	KIAA0430
0.517	0.033	MREG	0.628	0.109	CLCF1
0.582	0.101	SBF1	0.542	0.026	PHLPP1
0.532	0.054	H2AFJ	0.557	0.051	SCAND2P
0.537	0.097	SFN	0.519	0.022	ANGEL2
0.518	0.117	SUCO	0.602	0.108	BIN1
			0.536	0.046	ALAD
			0.536	0.046	CLEC16A
			0.536	0.046	FBXL18
			0.536	0.046	ZC2HC1A
			0.532	0.046	ZNF200
			0.545	0.063	RAB3A
			0.510	0.029	MTUS1
			0.510	0.031	MARCH8
			0.510	0.031	WIPI2
			0.551	0.076	STX3
			0.570	0.101	RANGAP1
			0.508	0.060	MINOS1P1
			0.556	0.109	FOXC1
			0.528	0.084	CBR3
			0.511	0.072	PIK3IP1
			0.517	0.083	LYPD3
			0.508	0.075	GCLC
			0.553	0.120	KIAA0232
			0.518	0.099	TRIM23
			0.502	0.117	PIGA

VCAP_6h based prediction result

Pa	Pi	DownRegulation	Pa	Pi	UpRegulation
0.995	0.001	SIN3B	0.997	0.003	OXSR1
0.996	0.004	TUBB6	0.996	0.003	PIK3R3
0.768	0.043	ZNF221	0.996	0.003	AMDHD2
0.687	0.027	HP	0.506	0.040	ABCF3
0.581	0.155	CETP	0.547	0.089	CBR3
0.540	0.129	OXT	0.536	0.180	C1ORF216
0.519	0.169	NUBP1	0.507	0.158	BABAM1

VCAP_24h based prediction result

Pa	Pi	DownRegulation	Pa	Pi	UpRegulation
0.997	0.001	STX10	0.997	0.001	EPHX2
0.997	0.001	TMEM2	0.997	0.001	CLDN7

0.997	0.001	FLAD1
0.996	0.001	ABCG1
0.996	0.002	LSM7
0.997	0.002	TERF2IP
0.996	0.002	EDF1
0.997	0.003	SMC3
0.997	0.003	SKIV2L
0.996	0.003	STK25
0.996	0.003	CSNK1A1
0.994	0.001	ODAM
0.994	0.001	GABPB1
0.996	0.003	PSME2
0.993	0.001	PGRMC2
0.555	0.110	COL9A3
0.525	0.106	CD96

0.997	0.001	CLDN3
0.997	0.001	IGLV1-40
0.997	0.001	C2
0.997	0.002	IGKV3-20
0.997	0.002	CD7
0.997	0.001	KIAA0664L3
0.997	0.002	COL6A3
0.997	0.002	TRAT1
0.997	0.002	SIVA1
0.997	0.002	IGKV1-5
0.997	0.002	IL1R1
0.997	0.002	TCF25
0.997	0.002	SLC4A4
0.997	0.002	NR4A1
0.997	0.002	FBXL12
0.996	0.002	ARPP21
0.997	0.002	PLP2
0.996	0.002	GLUL
0.997	0.003	RPA2
0.995	0.001	GMFB
0.995	0.001	RPP14
0.997	0.003	PRKAG2
0.995	0.001	SPOCK2
0.995	0.002	CD3G
0.996	0.003	COL11A1
0.996	0.003	PHKB
0.992	0.001	PID1
0.521	0.101	HOMER2

Combination prediction result

Name	Overlap count	Up	Down	Result
CBR3	3	3	0	
CDCA4	3	2	1	
CRBN	3	1	2	
CTSB	3	1	2	
DCAF17	3	1	2	
ELAVL1	3	1	2	
FKBP5	3	3	0	
GALNT6	3	1	2	
PDZK1	3	1	2	
RETSAT	3	1	2	
STK39	3	1	2	
UBE2C	3	2	1	
ZNF302	3	2	1	
AHCY	2	2	0	
AK2	2	0	2	

ANXA1	2	0	2	
C8ORF4	2	2	0	
CCR7	2	0	2	
CEACAM6	2	0	2	
CEP70	2	0	2	
CETP	2	0	2	
CHFR	2	2	0	
CHIC2	2	2	0	
CHST15	2	0	2	
CLCN2	2	0	2	
CLIC3	2	0	2	
DENND5B	2	0	2	
DIAPH2	2	0	2	
DLD	2	2	0	
DSE	2	0	2	
EHHADH	2	0	2	
ETAA1	2	0	2	
ETFB	2	2	0	
FBXL12	2	2	0	
FLAD1	2	0	2	
FNDC3B	2	0	2	
FOXD1	2	2	0	
GGH	2	2	0	
GJA1	2	0	2	
GNMT	2	2	0	
GPR19	2	0	2	
GPR20	2	0	2	
HIVEP1	2	2	0	
HMOX1	2	2	0	
ID1	2	2	0	
IER3	2	0	2	
IFIT1	2	2	0	
IKBKAP	2	0	2	
ITGB8	2	0	2	
KLF11	2	0	2	
KRT3	2	0	2	
LAMTOR2	2	2	0	
MAP3K5	2	0	2	
MAST4	2	0	2	
MCMBP	2	0	2	
MDM2	2	0	2	
MEAF6	2	2	0	
NOS2	2	0	2	
NR4A1	2	2	0	
ODF1	2	0	2	
PCMTD2	2	0	2	
PDK4	2	0	2	
PDS5B	2	2	0	

PIK3R3	2	2	0	
PLEC	2	0	2	
POLI	2	0	2	
PRDX6	2	2	0	
RARG	2	2	0	
RBM12	2	0	2	
REG1B	2	0	2	
ROBO4	2	0	2	
RPP14	2	2	0	
SECISBP2	2	0	2	
SELL	2	0	2	
SH3D21	2	0	2	
SLC25A37	2	0	2	
SLC4A8	2	0	2	
SLC6A4	2	0	2	
SPATA20	2	2	0	
SPOP	2	2	0	
SPR	2	2	0	
STK3	2	2	0	
SUV39H2	2	0	2	
TCF25	2	2	0	
TLR2	2	0	2	
TMPRSS2	2	2	0	
TOP1	2	2	0	
TPSB2	2	0	2	
TRIM13	2	2	0	
TSKU	2	2	0	
TSPAN5	2	0	2	
VAV3	2	0	2	
VIM	2	0	2	
WISP2	2	2	0	
ZBTB39	2	0	2	
ZFP30	2	0	2	

Complex 4

mRNA based prediction result

Pa	Pi	DownRegulation
0.903	0.001	CYP2S1
0.795	0.030	NSF
0.696	0.067	TRAF5
0.625	0.132	TPM1
0.558	0.096	ABCC5
0.583	0.123	ADORA2B
0.583	0.123	CDCA4
0.583	0.123	CENPJ
0.583	0.123	DNAJC9
0.583	0.123	DONSON
0.583	0.123	HAUS8
0.583	0.123	MCMBP
0.583	0.123	NUP155
0.583	0.123	PARP2
0.583	0.123	RMI1
0.583	0.123	SCARA3
0.583	0.123	SLC16A14
0.583	0.123	SUV39H2
0.562	0.145	GSS

Pa	Pi	UpRegulation
0.707	0.077	SFRP1
0.567	0.107	SQLE
0.583	0.123	PFKFB2
0.583	0.123	SH3BGRL
0.583	0.123	TRIM13
0.508	0.118	CALM1
0.525	0.187	UGT2B15

Protein based prediction result

Pa	Pi	DownRegulation
0.859	0.001	PR0.859)KCA

Pa	Pi	UpRegulation
0.585	0.103	CD83

MCF7 based prediction result

Pa	Pi	DownRegulation
0.562	0.042	BBS4
0.505	0.095	MRPL34
0.501	0.158	SBF1

Pa	Pi	UpRegulation
0.897	0.010	LPIN1
0.870	0.015	BNIP3L
0.720	0.004	MAFB
0.687	0.036	PLA2G3
0.629	0.046	KLHL24
0.628	0.046	STRN
0.635	0.059	CDKN1B
0.598	0.056	SQLE
0.581	0.073	HMGCS1
0.600	0.094	KIAA0430
0.581	0.076	CCNG2
0.532	0.039	EGR3
0.542	0.062	ALDOC

Pa	Pi	UpRegulation
0.565	0.086	C11orf68
0.568	0.094	ZNF672
0.510	0.038	SPRED2
0.616	0.149	CLDN9
0.511	0.068	WIPI1
0.501	0.060	EIF2AK3

VCAP_6h based prediction result

Pa	Pi	DownRegulation	Pa	Pi	UpRegulation
0.991	0.003	PPAN	0.990	0.004	ACADL
0.990	0.003	DBN1	0.989	0.004	FBXO21
0.990	0.004	WIZ	0.988	0.004	ETNK2
0.988	0.005	SOX4	0.986	0.003	KCTD9
0.987	0.004	NDUFA10	0.987	0.004	CCRL1
0.988	0.005	MLL4	0.986	0.005	MTFR1
0.987	0.005	CIZ1	0.985	0.003	SUMO2
0.987	0.004	ARMC6	0.985	0.004	ECM2
0.987	0.004	SCAMP1	0.984	0.004	GPR19
0.987	0.004	TMEM97	0.984	0.004	GMEB2
0.987	0.004	PEBP1	0.983	0.004	DERL1
0.984	0.002	KLHL21	0.983	0.005	APBA2
0.985	0.003	CGREF1	0.983	0.004	FZD5
0.986	0.005	RAD9A	0.983	0.005	DYNC1I1
0.984	0.005	PHGDH	0.981	0.003	MTO1
0.984	0.005	MRPS27	0.982	0.004	AIP
0.982	0.003	PTGER2	0.981	0.004	YWHAQ
0.983	0.004	PKN1	0.982	0.005	CARD9
0.983	0.004	ZDHHC4	0.981	0.004	NFE2L1
0.981	0.003	INO80B	0.981	0.004	PGM3
0.982	0.004	UPF3A	0.981	0.004	DNAJA2
0.982	0.004	ATP13A1	0.981	0.005	ARMCX3
0.982	0.004	PTPN2	0.981	0.005	TRAPPC6A
0.980	0.004	EPHB2	0.981	0.005	SNAP23
0.981	0.005	AKAP17A	0.980	0.005	GSDMB
0.980	0.004	BBX	0.980	0.005	SOCS2
0.981	0.004	CNPPD1	0.980	0.005	ARF6
0.980	0.004	TNFSF13	0.978	0.003	SLC38A2
0.980	0.005	TECR	0.978	0.004	RAP1B
0.980	0.004	ATAD3A	0.979	0.004	TYROBP
0.980	0.004	MAGED1	0.979	0.005	LPP
0.980	0.004	MPST	0.978	0.005	IPO13
0.978	0.003	MGC12488	0.979	0.005	SLCO2A1
0.978	0.003	LZTFL1	0.978	0.005	NCAPD2
0.979	0.004	B4GALT2	0.978	0.004	SORD
0.978	0.004	PTER	0.977	0.004	CYB5R4
0.979	0.004	LYRM4	0.978	0.005	KLF9

0.978	0.004	GORASP1	0.978	0.004	UBE2H
0.978	0.004	ALDH18A1	0.977	0.003	HERC2
0.978	0.003	AMFR	0.977	0.004	TMEM176B
0.978	0.004	IKBKB	0.977	0.004	RTN1
0.978	0.004	DNMT3B	0.977	0.005	TPPP3
0.978	0.005	KIAA0494	0.977	0.005	HSD17B6
0.976	0.004	ATP1B3	0.976	0.004	LIFR
0.976	0.003	TUBG2	0.977	0.005	MS4A4A
0.977	0.005	TM7SF2	0.976	0.004	KCTD15
0.977	0.004	YIPF2	0.976	0.004	ARAP3
0.977	0.004	FUT7	0.976	0.005	CORO2A
0.976	0.005	SORBS3	0.976	0.005	PEA15
0.976	0.005	C14ORF1	0.975	0.004	CST1
0.974	0.003	NDP	0.976	0.005	MORC3
0.975	0.004	SNAPC2	0.975	0.004	PTPN9
0.975	0.004	LEPROT	0.976	0.005	ANXA1
0.975	0.004	PARP4	0.975	0.004	SNAI2
0.976	0.005	WSB1	0.976	0.005	SMYD2
0.976	0.005	MRPL24	0.976	0.005	OAT
0.975	0.005	LTBP3	0.975	0.005	MOB3B
0.973	0.003	BACH2	0.976	0.005	MORF4L2
0.974	0.004	ESRRG	0.975	0.005	LAP3
0.975	0.005	RABAC1	0.975	0.005	EPS15
0.974	0.004	PRRC2B	0.975	0.005	STK17B
0.974	0.004	MRPL17	0.975	0.005	KIAA0930
0.974	0.005	ATP9A	0.975	0.005	CDH5
0.974	0.004	PWP2	0.975	0.005	PDLIM1
0.973	0.004	INPPL1	0.977	0.008	MIOS
0.973	0.005	RPL36	0.974	0.004	RNF115
0.973	0.005	NOLC1	0.975	0.005	USP15
0.971	0.003	GALNT10	0.974	0.005	ANPEP
0.970	0.003	ABCB8	0.974	0.005	PPEF1
0.970	0.003	EFNA3	0.972	0.003	NDUFB11
0.971	0.004	WDR46	0.973	0.005	CAST
0.972	0.005	MRPS31	0.973	0.005	PHOX2A
0.969	0.003	ADAM15	0.973	0.005	CLEC7A
0.971	0.005	LEPREL4	0.973	0.004	GSK3B
0.971	0.005	NAA15	0.972	0.005	G3BP2
0.969	0.003	MRPL52	0.973	0.005	MNDA
0.970	0.004	HMGA1	0.973	0.005	MMP9
0.970	0.004	RPL11	0.972	0.005	RGS16
0.970	0.004	BBC3	0.972	0.004	CD2AP
0.971	0.005	SMARCE1	0.971	0.004	OLFML2A
0.970	0.005	IP6K2	0.971	0.004	NCAPD3
0.970	0.004	RRNAD1	0.971	0.005	FAM189A2
0.969	0.004	COX15	0.971	0.005	CNOT8
0.970	0.005	CDC37	0.971	0.005	TRAPPC2

0.969	0.003	ECM2	0.971	0.005	RTF1
0.969	0.005	BRD2	0.971	0.005	PALLD
0.968	0.004	TLE3	0.971	0.005	ALKBH1
0.968	0.005	SREBF1	0.971	0.005	ZNF394
0.968	0.005	GALNT11	0.970	0.005	GPNMB
0.968	0.005	RANGAP1	0.970	0.005	TMEM204
0.967	0.005	IMPDH2	0.970	0.004	CREB5
0.967	0.004	RUFY2	0.970	0.005	CTRC
0.967	0.005	OAS1	0.969	0.004	UBE2G1
0.966	0.004	CAD	0.970	0.005	CPEB3
0.966	0.004	C9ORF91	0.970	0.005	FGD6
0.965	0.003	RPAP1	0.970	0.005	RBX1
0.965	0.004	TRAF3	0.970	0.005	FOXF2
0.968	0.007	FAM162A	0.968	0.003	ST18
0.966	0.005	KIAA0368	0.969	0.005	FAM127B
0.966	0.005	YIF1A	0.969	0.005	PSMC2
0.965	0.005	HTRA2	0.968	0.004	SLC37A1
0.965	0.005	U2AF1	0.968	0.005	SMAP1
0.964	0.004	RPH3AL	0.969	0.005	PLA2G7
0.965	0.005	PREP	0.968	0.005	CFI
0.965	0.005	GAR1	0.968	0.005	IGSF6
0.964	0.004	DNASE2	0.968	0.005	IL10RA
0.964	0.004	GDF2	0.968	0.005	TOMM22
0.962	0.003	APOBEC3F	0.966	0.002	FUBP3
0.963	0.004	FAM134C	0.967	0.005	LDB2
0.963	0.004	PNPLA6	0.967	0.005	C5ORF44
0.963	0.004	SPP1	0.967	0.005	RAPGEF5
0.963	0.005	ALG3	0.966	0.004	SALL2
0.963	0.004	FAM30A	0.969	0.007	BCL2L13
0.967	0.008	MLEC	0.972	0.011	EDEM3
0.962	0.004	PEMT	0.966	0.005	LOC100507397
0.962	0.005	SLC30A1	0.965	0.004	NUP153
0.962	0.004	FOXO4	0.965	0.005	PRLH
0.961	0.004	RPL6	0.965	0.005	NEK2
0.960	0.003	CES1P1	0.965	0.005	DDX3X
0.961	0.005	MECR	0.964	0.004	CSRP2
0.960	0.005	LEF1	0.963	0.004	BNIP2
0.960	0.004	PITRM1	0.964	0.005	DDX19A
0.962	0.006	PSD3	0.964	0.005	CLEC4A
0.961	0.006	DALRD3	0.963	0.004	C18ORF8
0.959	0.005	VCP	0.961	0.004	ZNF189
0.958	0.005	COL5A1	0.967	0.010	CYB561
0.959	0.006	PRKCSH	0.961	0.005	PNOC
0.958	0.005	TERF1	0.961	0.005	INTS7
0.958	0.005	EHMT2	0.961	0.005	FGL2
0.957	0.004	GMDS	0.961	0.005	ZNF137P
0.957	0.005	C9ORF40	0.960	0.004	CHMP2B

0.954	0.003	TMEM214	0.959	0.005	PTPRO
0.959	0.008	RIC8A	0.959	0.005	HNRNPC
0.962	0.012	SRSF1	0.959	0.005	TMEM176A
0.953	0.004	THADA	0.959	0.005	COPS2
0.954	0.005	POLD2	0.959	0.005	CHN2
0.952	0.003	RAB11FIP5	0.958	0.004	C1ORF115
0.952	0.006	TSEN34	0.958	0.005	NDUFA6
0.955	0.009	EXOSC10	0.958	0.005	CREBL2
0.952	0.007	SRSF11	0.958	0.006	RAC3
0.948	0.004	PDCD6IP	0.959	0.006	TGM4
0.948	0.005	ST7	0.957	0.005	CES2
0.947	0.006	NKTR	0.957	0.005	C1ORF21
0.945	0.005	EHD1	0.957	0.004	UBE2D3
0.944	0.004	OXSM	0.957	0.005	PPP1R16B
0.944	0.004	ARHGEF1	0.954	0.003	SNX10
0.956	0.016	C21ORF33	0.955	0.005	SUMO1
0.943	0.005	KRR1	0.954	0.005	WLS
0.947	0.009	SRSF7	0.954	0.005	KIAA1462
0.942	0.005	NIT2	0.953	0.004	CDCP1
0.942	0.005	METTL21D	0.953	0.005	WARS
0.943	0.008	TSPAN4	0.953	0.005	SLC27A3
0.953	0.018	RHEB	0.952	0.005	CYTIP
0.938	0.005	TLE6	0.956	0.009	TM6SF1
0.938	0.005	UBE2C	0.956	0.010	ANAPC10
0.934	0.004	SETD5	0.954	0.008	CFD
0.940	0.010	IP6K1	0.956	0.010	USP10
0.935	0.005	EIF3E	0.951	0.005	SNX16
0.934	0.004	CRYL1	0.949	0.005	SEMG1
0.939	0.009	GAA	0.949	0.006	HYI
0.937	0.008	C10ORF2	0.952	0.009	WDR44
0.939	0.013	C12ORF10	0.949	0.006	TBPL1
0.934	0.010	H2BFS	0.949	0.007	SLC5A6
0.932	0.009	GORASP2	0.945	0.004	SCGB1D2
0.929	0.006	TBL3	0.949	0.008	CCNH
0.931	0.009	RUVBL2	0.947	0.008	PCGF3
0.929	0.008	ILK	0.945	0.006	ELF2
0.926	0.006	STRN4	0.944	0.005	TRIM22
0.924	0.005	AFG3L2	0.943	0.004	PGAM2
0.923	0.005	NPAS1	0.943	0.005	SERPINI2
0.924	0.009	EEF1B2	0.945	0.008	RANBP10
0.919	0.004	NELF	0.948	0.011	SH3BP5
0.924	0.010	GPD1L	0.942	0.005	HPSE
0.925	0.012	CD151	0.942	0.006	STAM
0.921	0.008	SEC61A1	0.940	0.005	NFKBIL1
0.932	0.020	SH3GLB2	0.943	0.008	ITGA8
0.927	0.015	NHP2	0.939	0.005	ITM2B
0.925	0.013	DEAF1	0.937	0.005	SEPT10

0.920	0.009	COBRA1	0.941	0.009	FAM198B
0.919	0.008	LYPD1	0.943	0.013	PRPF4B
0.933	0.023	KAZN	0.932	0.005	KCNJ8
0.921	0.013	PPP3CB	0.931	0.005	KATNB1
0.919	0.012	UBE2M	0.932	0.007	UBE2N
0.917	0.011	AK2	0.934	0.010	ZNF22
0.915	0.013	ACTR2	0.931	0.007	C4ORF41
0.911	0.009	PPP1CA	0.933	0.010	SLC4A4
0.909	0.008	SRPRB	0.930	0.007	CD93
0.906	0.006	GNB5	0.933	0.012	CPA2
0.909	0.010	SDAD1	0.931	0.012	SH3GLB1
0.904	0.005	WDR48	0.934	0.015	NEDD9
0.901	0.004	ARMCX6	0.922	0.006	APOLD1
0.907	0.011	HEXIM1	0.924	0.008	SPG11
0.909	0.015	FBXO7	0.926	0.011	MARK4
0.900	0.005	LUC7L2	0.920	0.005	GDF2
0.906	0.012	HSD17B10	0.923	0.009	CSTF2T
0.909	0.016	KIF2C	0.923	0.009	BHLHB9
0.901	0.013	FAM57A	0.920	0.010	CAV1
0.910	0.024	ATP6V0E1	0.921	0.012	FOXF1
0.901	0.016	EEF1G	0.923	0.015	CDH1
0.892	0.007	SEC24A	0.920	0.012	LRRC16A
0.902	0.018	NGFRAP1	0.920	0.013	FILIP1L
0.899	0.018	C19ORF22	0.921	0.014	HNRNPD
0.895	0.020	NDUFV1	0.920	0.014	AOAH
0.881	0.007	RPL35	0.916	0.010	ATG5
0.886	0.011	SAC3D1	0.911	0.006	YWHAZ
0.887	0.013	ZNF302	0.919	0.015	UBR2
0.897	0.025	SLC35E1	0.909	0.008	VWA1
0.883	0.015	RSAD1	0.912	0.011	EDC3
0.885	0.018	MLST8	0.906	0.006	WDR45L
0.877	0.010	RUFY1	0.908	0.008	SPDEF
0.898	0.032	RCC1	0.907	0.009	LGI1
0.881	0.018	CHD1L	0.904	0.008	LPPR4
0.882	0.019	DNPEP	0.903	0.008	CREB3L1
0.880	0.018	ATP5G1	0.900	0.005	NIT1
0.878	0.018	LSM4	0.900	0.005	KIAA1324
0.877	0.021	FAM50A	0.904	0.009	KAL1
0.873	0.017	BANF1	0.909	0.014	KRI1
0.877	0.022	PLOD3	0.907	0.015	GTSE1
0.873	0.018	HSPBP1	0.899	0.008	CD300C
0.864	0.011	C2CD2	0.900	0.010	OMD
0.865	0.014	COX17	0.898	0.009	EMCN
0.866	0.020	KCTD3	0.902	0.014	OR51E2
0.867	0.028	TUFM	0.898	0.010	ZMYM1
0.844	0.005	KIAA0754	0.907	0.019	RAB9A
0.859	0.020	PRMT1	0.893	0.009	RAPGEF4

0.846	0.016	BST2	0.900	0.017	ESRRG
0.846	0.017	TPX2	0.899	0.015	CAB39L
0.857	0.029	RNASEH1	0.892	0.009	SLC33A1
0.853	0.029	ARPC4	0.896	0.013	APPBP2
0.856	0.032	SMARCD2	0.897	0.018	DLG2
0.839	0.016	PRDX4	0.903	0.025	CLPX
0.834	0.013	PRAF2	0.909	0.031	ZNF318
0.840	0.020	PPT1	0.894	0.019	UBXN4
0.846	0.026	GOLGA2	0.892	0.018	GNRH2
0.842	0.025	SIGMAR1	0.885	0.011	F11R
0.840	0.024	FIBP	0.878	0.005	CCNL1
0.841	0.026	NME1	0.888	0.016	FAM188A
0.833	0.019	NIP7	0.891	0.021	SEC23A
0.835	0.022	PDSS2	0.881	0.012	ITIH1
0.844	0.050	MAP3K7	0.876	0.008	ARL15
0.823	0.035	MTHFD2	0.890	0.022	ALDH5A1
0.818	0.033	ENDOG	0.885	0.018	UEVLD
0.803	0.019	KPTN	0.885	0.020	RBM12
0.810	0.028	RPL15	0.879	0.016	SEC24D
0.811	0.029	UBE2V2	0.878	0.018	ETFDH
0.812	0.032	GPI	0.875	0.015	PAQR3
0.802	0.036	RNASEH2A	0.873	0.016	CDK2
0.811	0.048	CACNA1B	0.872	0.015	HHEX
0.803	0.040	PPP2R4	0.870	0.013	STX4
0.799	0.038	ARF5	0.879	0.023	TOX4
0.789	0.028	C11ORF2	0.871	0.016	PIBF1
0.807	0.047	BRD3	0.876	0.022	CIAO1
0.784	0.027	HIST1H2BF	0.869	0.016	APP
0.797	0.042	CCT3	0.869	0.015	LIN7C
0.789	0.036	DNTTIP2	0.872	0.020	CDH4
0.806	0.054	U2AF2	0.868	0.016	PPM1B
0.799	0.048	PEX10	0.866	0.017	NR2E1
0.782	0.031	CELF1	0.869	0.020	SERPINA1
0.802	0.051	STAMBP	0.869	0.021	UHRF1BP1L
0.767	0.022	SRM	0.854	0.008	BMX
0.780	0.035	TROAP	0.860	0.017	NUP98
0.796	0.051	NUP210	0.855	0.012	GC
0.775	0.032	GLT25D1	0.867	0.025	AP1S2
0.787	0.049	MAN1B1	0.852	0.011	PWP1
0.770	0.038	CDCA3	0.865	0.025	HMX1
0.762	0.032	SKIV2L	0.858	0.018	CLEC1A
0.756	0.027	MICU1	0.858	0.021	CEP170
0.783	0.053	TM9SF1	0.863	0.028	MYO1B
0.762	0.033	NCOA3	0.850	0.017	HIPK1
0.784	0.055	SNX6	0.844	0.011	SERHL2
0.778	0.052	SDCCAG3	0.859	0.026	C18ORF10
0.748	0.024	PSMG1	0.851	0.020	F13A1

0.775	0.052	CHEK2	0.848	0.022	C1ORF38
0.769	0.048	TRIM28	0.849	0.023	C8ORF60
0.761	0.040	ZNF544	0.836	0.014	CPA3
0.746	0.036	HRAS	0.861	0.039	COPS8
0.757	0.048	IPO5	0.834	0.014	EVI2A
0.773	0.065	STK25	0.848	0.029	SPARCL1
0.744	0.038	TPD52	0.851	0.034	LANCL2
0.760	0.054	CCT6A	0.844	0.027	CANX
0.759	0.053	DSC3	0.847	0.030	C4BPB
0.760	0.058	BCS1L	0.847	0.030	OFD1
0.751	0.052	TUSC2	0.839	0.026	CAMSAP2
0.752	0.056	BAG5	0.828	0.018	IL13RA1
0.750	0.055	SLC35B1	0.842	0.035	PEX7
0.764	0.075	RPS28	0.842	0.036	CALD1
0.724	0.039	MRTO4	0.849	0.043	VPS41
0.720	0.035	KIAA0100	0.839	0.035	GDPD5
0.758	0.082	SELL	0.830	0.027	ZNF223
0.737	0.062	RUVBL1	0.826	0.024	APLNR
0.723	0.048	EBP	0.823	0.023	GIMAP6
0.726	0.053	DYNLRB1	0.822	0.023	NOTCH4
0.732	0.063	ACSL4	0.832	0.033	STMN4
0.724	0.064	SCD	0.839	0.040	EDN3
0.730	0.069	MBD3	0.821	0.023	OSBPL3
0.698	0.050	PPIE	0.831	0.034	PDE4DIP
0.693	0.047	POLR2H	0.814	0.018	GRM3
0.708	0.067	MZT2B	0.839	0.045	MEF2A
0.708	0.068	TNPO1	0.814	0.019	ZNF557
0.678	0.041	UBE2S	0.819	0.026	KLHL2
0.690	0.058	KARS	0.809	0.020	KIAA0556
0.689	0.063	KEAP1	0.817	0.031	VRK2
0.675	0.052	TXLNA	0.808	0.024	CLIC5
0.690	0.072	ZNF221	0.805	0.024	CDKL3
0.684	0.078	KPNB1	0.802	0.020	FEZF2
0.654	0.051	MTFR1	0.816	0.035	FCER1G
0.678	0.077	UTP6	0.810	0.035	ITIH3
0.662	0.063	PYCR1	0.801	0.029	ENTPD1
0.656	0.059	STMN1	0.803	0.031	ZNF204P
0.664	0.068	GNPDA1	0.795	0.023	GABRA5
0.651	0.058	SNRPD2	0.797	0.027	NECAB2
0.667	0.076	PRMT5	0.813	0.045	ZC3H7A
0.658	0.071	SEH1L	0.792	0.025	GDAP1L1
0.682	0.096	RAB11A	0.790	0.024	BET1L
0.671	0.087	DHX30	0.808	0.043	SKAP2
0.648	0.067	SFXN1	0.803	0.037	AQP9
0.653	0.076	RPS9	0.783	0.020	ZNF276
0.641	0.072	CCNA2	0.796	0.033	JMJD6
0.648	0.082	TRAM1	0.795	0.034	CASP4

0.645	0.079	RPS2	0.800	0.038	TGFB1
0.666	0.101	SP110	0.806	0.046	EGFR
0.622	0.057	USP14	0.781	0.023	MARCH1
0.668	0.106	ZC3H14	0.776	0.018	TCIRG1
0.644	0.089	COPB1	0.786	0.030	ASGR2
0.603	0.058	GNB2L1	0.789	0.034	CSF3R
0.638	0.093	A4GALT	0.789	0.035	ACSM1
0.652	0.108	CXORF56	0.788	0.034	BDH2
0.610	0.066	NOP56	0.785	0.032	SERPINB9
0.625	0.089	DDX49	0.796	0.044	RAB6B
0.653	0.118	DIAPH2	0.786	0.035	FEM1B
0.563	0.035	CSDA	0.786	0.037	ADAMTS13
0.591	0.064	HP	0.795	0.046	ST20
0.620	0.106	YWHAB	0.804	0.056	ZNF536
0.607	0.097	VEGFB	0.765	0.017	CCDC102B
0.583	0.078	TFCP2L1	0.762	0.017	SLC2A10
0.607	0.110	FAM134A	0.787	0.042	CDH11
0.585	0.090	PNP	0.789	0.049	PRTN3
0.609	0.114	SCRIB	0.775	0.040	TTLL7
0.561	0.066	GLI3	0.755	0.022	PTPRK
0.598	0.106	CENPC1	0.788	0.056	NFATC2IP
0.594	0.104	TMED9	0.776	0.046	MOSPD2
0.601	0.122	SYNCRIP	0.764	0.034	EFR3A
0.601	0.124	ASNA1	0.777	0.049	CNPY3
0.571	0.097	FARSA	0.759	0.031	CLDND1
0.577	0.107	TMEM160	0.773	0.045	RNF111
0.606	0.135	ADM	0.755	0.028	PDHX
0.571	0.103	EDF1	0.758	0.031	CACNG3
0.583	0.116	IMP4	0.771	0.048	TMOD3
0.577	0.115	BABAM1	0.813	0.091	ZFAND1
0.549	0.088	CRTAM	0.760	0.040	SPAST
0.569	0.108	GSS	0.752	0.035	ANKRA2
0.547	0.090	MAT2A	0.749	0.033	KCNV1
0.579	0.124	TCEA2	0.750	0.035	HBEGF
0.564	0.113	GHITM	0.751	0.036	CADPS
0.532	0.083	DUX1	0.765	0.052	PPP2R5A
0.527	0.088	CDKN2A	0.762	0.053	MDFIC
0.515	0.082	SH3BP1	0.740	0.032	FAM49A
0.517	0.085	BAGE	0.729	0.021	PDE5A
0.546	0.117	RPL22	0.761	0.054	PLP2
0.541	0.112	APEH	0.741	0.041	CD38
0.543	0.121	EIF3G	0.756	0.057	CD48
0.525	0.105	FMNL1	0.743	0.048	GABRG2
0.518	0.105	XBP1	0.753	0.058	NUMB
0.521	0.119	CLPX	0.730	0.043	CEBPE
0.531	0.130	FADS1	0.755	0.069	CLCN3
0.517	0.119	RNMT	0.724	0.039	TREML2

0.522	0.123	ELOVL6
0.565	0.174	KCNC2
0.521	0.130	MGAT4B
0.503	0.116	LIPC
0.522	0.137	DNAJC9
0.547	0.167	CETP
0.515	0.139	PHB
0.504	0.131	ATP6V0B
0.509	0.139	EIF4E
0.510	0.142	GTF3A
0.515	0.162	WDR44
0.718	0.033	COPB2
0.727	0.043	PITX3
0.725	0.042	KCNQ1DN
0.727	0.048	AAK1
0.721	0.042	TMEM144
0.741	0.064	SCAF4
0.743	0.068	HABP4
0.724	0.052	PHIP
0.717	0.044	DUS4L
0.704	0.032	GAS8
0.711	0.039	RFPL1-AS1
0.719	0.047	WDR78
0.721	0.050	NFE2L2
0.725	0.056	PRKX
0.720	0.053	POLE2
0.729	0.064	LPXN
0.719	0.058	SLC38A6
0.687	0.026	KIF20A
0.703	0.047	KIAA1107
0.696	0.042	LMBRD1
0.717	0.068	AGRN
0.705	0.057	OR7E47P
0.701	0.057	SLC16A5
0.707	0.068	LY86
0.686	0.051	ADAT1
0.693	0.062	THRAP3
0.698	0.072	TRIM14
0.677	0.052	FPR3
0.691	0.070	PDE4B
0.690	0.069	EMR2
0.681	0.063	RBM15B
0.687	0.070	HSPA4
0.682	0.067	WIZ
0.661	0.049	CNKSR2
0.675	0.063	PTPRF
0.658	0.052	ZNF589
0.665	0.060	FRMPD4
0.665	0.060	NUDT21
0.652	0.047	PRR15L
0.676	0.078	WTAP
0.668	0.073	CASP1
0.683	0.089	TWF1
0.655	0.065	HIST2H2BE
0.684	0.094	CCR1
0.653	0.069	KALRN
0.648	0.065	AQR
0.645	0.063	INTS1

	0.655	0.073	CLEC5A
	0.639	0.058	MTM1
	0.602	0.021	ABCF3
	0.655	0.075	TNFAIP1
	0.664	0.084	THAP3
	0.666	0.087	CD68
	0.690	0.111	PSMD7
	0.648	0.071	POU4F1
	0.654	0.080	ZNF770
	0.639	0.065	PIAS1
	0.646	0.075	TBX10
	0.634	0.065	CA4
	0.649	0.082	ATP6V1C1
	0.606	0.039	HK1
	0.647	0.080	EPS8L1
	0.643	0.081	CST2
	0.633	0.071	F2
	0.692	0.133	NGFRAP1
	0.654	0.099	HK2
	0.644	0.093	SLC4A7
	0.655	0.105	KPNA2
	0.639	0.092	MON2
	0.649	0.107	EIF4E2
	0.626	0.087	FAM179B
	0.637	0.101	B4GALT1
	0.614	0.080	PEX11B
	0.622	0.090	YIPF4
	0.588	0.072	PCYT2
	0.621	0.107	SMARCA5
	0.602	0.089	HPX
	0.608	0.095	HOMER3
	0.591	0.081	ST3GAL2
	0.614	0.104	MPZL1
	0.609	0.099	CTBS
	0.631	0.122	PNISR
	0.587	0.078	PEX5L
	0.606	0.098	TROVE2
	0.580	0.077	AQP1
	0.590	0.091	SERTAD2
	0.597	0.099	HAP1
	0.600	0.103	P2RY1
	0.598	0.102	TCF12
	0.605	0.109	SEPT6
	0.602	0.106	SSBP1
	0.594	0.098	ACAP2
	0.592	0.099	DIDO1
	0.547	0.056	INPP1

	0.593	0.104	CCL2
	0.574	0.089	LOC254896
	0.584	0.103	SLC35A2
	0.577	0.096	HAND2
	0.577	0.098	RAB28
	0.585	0.106	GDF11
	0.560	0.081	MCM6
	0.591	0.114	LINC00342
	0.586	0.115	CD44
	0.576	0.105	FOLR3
	0.563	0.104	N4BP1
	0.566	0.111	NARFL
	0.544	0.091	RAB7A
	0.547	0.095	SIM2
	0.559	0.107	TRPC6
	0.546	0.097	CALCRL
	0.544	0.096	FAM105A
	0.568	0.121	PPP2CB
	0.554	0.109	NFYA
	0.551	0.107	SCN3B
	0.543	0.101	MUC8
	0.554	0.118	PAPOLA
	0.548	0.119	EIF5A
	0.531	0.107	SEPT4
	0.514	0.090	TMSB15A
	0.552	0.131	SOCS5
	0.542	0.126	MMP14
	0.539	0.127	IL11
	0.533	0.122	BMP2K
	0.527	0.118	SLC25A14
	0.522	0.114	CASC1
	0.535	0.130	CDKN2C
	0.545	0.140	ALG13
	0.525	0.124	CCR2
	0.530	0.132	TMEM5
	0.520	0.122	OGFR
	0.520	0.123	ACO2
	0.523	0.131	KDM6B
	0.547	0.156	FAM13B
	0.514	0.123	CNTN2
	0.505	0.116	MOBP
	0.513	0.129	ZCCHC11
	0.515	0.133	GRB10
	0.504	0.130	FGF1
	0.504	0.130	PIGB
	0.506	0.133	PTMS
	0.501	0.129	NPPA

0.503	0.133	PHKB
0.504	0.135	C5AR1
0.508	0.140	HUWE1
0.521	0.154	TMEM93
0.501	0.144	PEX1
0.511	0.155	DDIT4
0.522	0.168	CXADR
0.507	0.159	TGOLN2

VCAP_24h based prediction result

Pa	Pi	DownRegulation	Pa	Pi	UpRegulation
0.995	0.004	SPAG7	0.996	0.004	USP46
0.991	0.003	HAX1	0.995	0.004	DZIP3
0.991	0.004	EIF3I	0.994	0.004	CPT1A
0.991	0.004	RANGAP1	0.994	0.004	SLC23A2
0.990	0.004	SPATS2	0.993	0.004	TCL1B
0.991	0.005	GGPS1	0.994	0.005	DENND2D
0.990	0.004	ZNF259	0.993	0.005	NRG2
0.991	0.005	UQCRB	0.993	0.004	FRK
0.990	0.004	GRSF1	0.992	0.004	CLCN7
0.989	0.004	PFDN5	0.993	0.004	PRIM2
0.990	0.005	TMEM97	0.992	0.004	SH3D21
0.988	0.004	C16ORF88	0.992	0.004	CD164
0.988	0.003	SMCHD1	0.992	0.005	PLCH1
0.989	0.005	SKIV2L	0.992	0.004	TMF1
0.988	0.004	LMF2	0.992	0.004	USH1C
0.988	0.004	PSPH	0.992	0.005	CADM4
0.987	0.003	RAB7L1	0.991	0.003	PREP
0.988	0.005	HNRNPH3	0.992	0.005	PDIA5
0.987	0.004	RAP2C	0.991	0.004	HSD3B2
0.988	0.004	NUP98	0.991	0.005	PART1
0.988	0.004	NPIP	0.991	0.004	PLEC
0.987	0.004	DAZAP2	0.991	0.004	GRSF1
0.987	0.004	NBPF10	0.991	0.004	DYRK2
0.988	0.004	PDIA4	0.990	0.004	GOLGA4
0.988	0.004	PPIF	0.990	0.004	FAM171A1
0.987	0.004	WWP1	0.990	0.004	ETAA1
0.987	0.004	GRK6	0.990	0.004	NCRNA00185
0.987	0.005	TIAL1	0.990	0.004	SCN9A
0.987	0.005	SYPL1	0.990	0.004	ABHD2
0.986	0.004	ZFAND1	0.990	0.004	CDSN
0.987	0.005	STAU1	0.989	0.003	RNF126P1
0.986	0.004	DYNC1LI1	0.990	0.004	RAP1A
0.986	0.004	HSP90AB1	0.990	0.004	RAB3B
0.986	0.005	SLC4A2	0.990	0.004	RIBC2
0.984	0.003	MYBL1	0.990	0.005	POU3F1

0.984	0.003	MCL1	0.990	0.004	KHSRP
0.986	0.005	MLLT11	0.990	0.004	PIAS3
0.985	0.005	PSMB9	0.990	0.004	UFL1
0.985	0.004	MSL1	0.990	0.005	DLG3
0.984	0.004	NAAA	0.990	0.005	PCYT1B
0.983	0.003	FGFR4	0.990	0.004	NKX3-1
0.982	0.002	RIT1	0.990	0.004	CGA
0.983	0.004	NEK4	0.990	0.004	PPOX
0.982	0.004	MTUS1	0.990	0.005	RAB30
0.981	0.003	RAD54L2	0.989	0.004	FAM86A
0.982	0.004	IL6ST	0.989	0.004	UGT2B17
0.982	0.004	PHF10	0.990	0.005	CAPN10
0.982	0.004	GPBP1L1	0.989	0.004	GAB2
0.982	0.004	GADD45B	0.990	0.005	ITGB1
0.983	0.005	RNF220	0.990	0.005	GGA2
0.982	0.004	PLS3	0.990	0.005	UBA6
0.980	0.003	STAT5B	0.990	0.005	SCUBE3
0.982	0.004	TALDO1	0.988	0.003	SUMO4
0.981	0.004	BST2	0.988	0.003	KLHL25
0.982	0.005	TCTN3	0.989	0.005	ARHGAP44
0.982	0.005	C12ORF43	0.989	0.005	LMF1
0.981	0.004	P2RX5	0.989	0.004	AASS
0.981	0.005	DCTN6	0.989	0.004	U2AF2
0.980	0.003	TSPAN7	0.987	0.003	ABHD11
0.980	0.004	IFIT3	0.989	0.005	MALT1
0.981	0.005	PPP1R12A	0.987	0.004	NAT6
0.981	0.005	KDM2A	0.988	0.004	EHHADH
0.981	0.004	GAP43	0.988	0.005	PAX5
0.981	0.004	YARS	0.987	0.003	ETV4
0.981	0.004	STK17A	0.987	0.003	IQGAP2
0.981	0.005	SCAMP4	0.988	0.005	LPHN3
0.981	0.005	TRAM2	0.988	0.005	CHML
0.981	0.005	MAX	0.988	0.005	PDS5B
0.980	0.004	HERC1	0.987	0.004	NUDT18
0.980	0.005	LIMA1	0.988	0.005	LTK
0.980	0.004	TTC4	0.987	0.004	CHL1
0.980	0.005	CCDC93	0.987	0.004	RPS6KA2
0.980	0.004	GOLGA2	0.987	0.004	HAPLN1
0.980	0.004	CDK10	0.988	0.005	ST8SIA4
0.980	0.004	PAK2	0.986	0.004	ATP5L
0.980	0.005	BFAR	0.987	0.004	MYO1A
0.979	0.004	TTI2	0.987	0.005	ATP5C1
0.980	0.004	UBXN7	0.987	0.004	FAM188A
0.980	0.005	RAB14	0.986	0.004	PIGZ
0.978	0.003	NRGN	0.987	0.005	PCCA
0.980	0.005	ING1	0.987	0.005	PQBP1
0.980	0.004	GFER	0.986	0.004	POF1B

0.980	0.005	PTP4A2	0.986	0.004	NPPB
0.979	0.004	ZNF189	0.987	0.005	ATP10B
0.980	0.005	SNRPC	0.986	0.005	TTC30A
0.979	0.004	FAM193A	0.986	0.005	UMPS
0.980	0.005	NBL1	0.985	0.003	RASSF7
0.979	0.004	CALCOCO2	0.985	0.003	SLC12A7
0.979	0.004	KDELR3	0.987	0.005	YTHDC2
0.980	0.005	UBAP2	0.986	0.005	CD24
0.979	0.004	CLDN18	0.986	0.005	FGF5
0.979	0.004	ACTR1A	0.986	0.005	LRP5L
0.979	0.005	LSG1	0.986	0.005	NPFFR1
0.978	0.003	UGP2	0.986	0.005	ITPR1
0.978	0.003	MS4A4A	0.985	0.005	GNA11
0.979	0.005	PFDN1	0.985	0.005	SEC31A
0.979	0.005	SRP54	0.985	0.005	ZCWPW1
0.978	0.004	RASSF4	0.983	0.003	TBCA
0.977	0.003	TNFSF9	0.983	0.003	TSPAN1
0.979	0.005	RABIF	0.984	0.004	UTF1
0.976	0.002	DSG3	0.985	0.005	AMBP
0.978	0.004	PTMS	0.985	0.005	NR2F6
0.979	0.005	APBA2	0.984	0.005	ARHGAP33
0.978	0.004	VAMP8	0.984	0.004	FAM174B
0.978	0.004	PRSS3	0.985	0.005	CALM1
0.978	0.005	LIAS	0.984	0.004	C9ORF167
0.977	0.004	CTSB	0.984	0.005	KDM4C
0.978	0.004	POMT1	0.984	0.005	ZFHX3
0.978	0.004	UIMC1	0.984	0.005	NCOA2
0.978	0.005	ITM2B	0.984	0.005	ANGPTL7
0.979	0.005	CCNG2	0.984	0.004	IDH2
0.978	0.004	MT3	0.983	0.004	MNS1
0.979	0.005	TTF1	0.983	0.004	LAP3
0.978	0.005	CIAO1	0.984	0.005	COL11A1
0.977	0.004	SFRP4	0.984	0.005	OSBPL10
0.978	0.005	LSM14A	0.984	0.005	IL17RC
0.978	0.004	NUDT4	0.983	0.005	FBXO21
0.978	0.005	DIAPH1	0.982	0.003	HERC2
0.978	0.004	SPATA2	0.983	0.004	IRGQ
0.978	0.005	PEX14	0.983	0.004	FSCN1
0.978	0.005	COPS8	0.983	0.005	ARID1A
0.978	0.005	TCEA1	0.983	0.005	F5
0.977	0.004	MEGF9	0.982	0.004	GPATCH1
0.978	0.005	SHQ1	0.982	0.004	QRSL1
0.977	0.004	PRKD2	0.980	0.002	ADPGK
0.977	0.004	DVL3	0.982	0.004	KIAA0894
0.977	0.004	FUCA1	0.983	0.005	B3GAT1
0.977	0.004	PLEKHA1	0.982	0.004	EXT2
0.977	0.005	M6PR	0.982	0.005	CEP57

0.977	0.005	OBFC2B	0.982	0.005	CCL23
0.978	0.005	DDA1	0.982	0.004	RBL1
0.978	0.005	BANP	0.982	0.004	ZDHHC13
0.978	0.005	COMMD9	0.982	0.005	HIST1H1D
0.977	0.005	ELMO2	0.982	0.005	ZEB2
0.976	0.004	PPAP2C	0.982	0.004	INPP1
0.977	0.004	C6ORF64	0.982	0.004	ACTA1
0.976	0.004	REEP1	0.981	0.004	UFC1
0.977	0.005	SRPR	0.981	0.004	PDE8B
0.976	0.003	IGLV2-23	0.981	0.004	LOC399491
0.977	0.004	AGPAT1	0.982	0.005	DDC
0.976	0.004	TSPYL2	0.981	0.004	KLK5
0.977	0.005	GCC2	0.980	0.004	ANPEP
0.974	0.002	F13A1	0.982	0.005	CPS1-IT1
0.978	0.005	PPP6C	0.981	0.005	IL1A
0.976	0.004	FZD7	0.981	0.005	NAP1L2
0.977	0.005	ISCU	0.981	0.005	P4HB
0.976	0.004	CRNN	0.981	0.005	NUS1P3
0.977	0.005	UBAP1	0.981	0.005	SRSF3
0.976	0.004	BCKDHB	0.981	0.005	TYRP1
0.977	0.005	MRPL18	0.980	0.004	CLN6
0.977	0.005	TUBGCP4	0.981	0.005	LCK
0.976	0.004	ATP6V1E1	0.980	0.004	RYR1
0.976	0.004	PGM3	0.981	0.005	MFSD6
0.976	0.004	UGT2A3	0.980	0.004	FARP2
0.976	0.004	PCSK7	0.980	0.004	WARS2
0.976	0.005	TRAM1	0.981	0.005	ZMAT5
0.976	0.004	RGL2	0.980	0.005	ARHGEF16
0.976	0.004	DCAF10	0.981	0.005	NCF4
0.976	0.005	DLX2	0.980	0.004	COL9A1
0.976	0.005	NECAP2	0.980	0.004	NLGN4X
0.975	0.004	FAM8A1	0.980	0.005	LOC202181
0.976	0.005	PRR7	0.980	0.005	KLC2
0.975	0.004	CD7	0.982	0.006	FAM153A
0.974	0.002	SERHL2	0.980	0.005	C19ORF40
0.976	0.005	DERL2	0.980	0.005	LPHN1
0.976	0.004	MUM1	0.980	0.005	AZI2
0.975	0.004	CHMP3	0.980	0.005	ULK2
0.976	0.004	NKIRAS2	0.979	0.004	DAAM1
0.977	0.006	C12ORF48	0.979	0.004	LRIG1
0.976	0.005	NXF1	0.980	0.005	HEXA
0.976	0.005	E2F6	0.979	0.004	TMEM126B
0.976	0.005	RBMS1	0.980	0.005	ATP9A
0.974	0.003	TNFRSF12A	0.979	0.004	MEGF6
0.975	0.004	C1QTNF1	0.980	0.005	TMEM151B
0.976	0.005	STK19	0.979	0.004	MPV17
0.975	0.004	CLK3	0.980	0.005	KLK7

0.976	0.005	IDH3G	0.979	0.005	ZHX2
0.976	0.005	TRAP1	0.980	0.005	FOXH1
0.975	0.004	CD82	0.979	0.005	ECHDC3
0.975	0.005	C1ORF216	0.979	0.005	TAGLN2
0.974	0.004	FBXO34	0.979	0.005	KLK8
0.974	0.004	FAM102A	0.978	0.004	DNMT3B
0.975	0.004	C9ORF114	0.979	0.005	GAN
0.975	0.005	C9ORF78	0.979	0.005	NUP43
0.974	0.005	ZNF529	0.979	0.005	FOXJ1
0.973	0.003	SALL1	0.980	0.005	SORBS1
0.973	0.004	C1ORF61	0.977	0.003	BRMS1
0.974	0.005	DUS4L	0.980	0.006	KRT85
0.973	0.004	ZDHHC18	0.979	0.005	PROC
0.973	0.004	SCMH1	0.979	0.005	ICOSLG
0.974	0.005	AKAP8	0.979	0.005	CCDC165
0.974	0.005	TNPO1	0.979	0.005	MAP2K7
0.973	0.004	DBN1	0.979	0.005	TOMM22
0.973	0.005	ATG13	0.978	0.004	TRMT61B
0.974	0.005	RPL29	0.978	0.004	KHDRBS3
0.973	0.004	COL6A2	0.978	0.004	ZNF226
0.973	0.004	BAP1	0.978	0.005	C8ORF60
0.973	0.005	CNPY3	0.978	0.004	GLTPD1
0.973	0.005	PKD1P1	0.978	0.004	FAM173A
0.973	0.005	RPS19	0.978	0.005	GNG4
0.972	0.004	NEU1	0.978	0.005	PPAP2A
0.972	0.004	TNKS2	0.978	0.004	ZNF33B
0.971	0.003	C16ORF7	0.977	0.004	GOLGA8H
0.973	0.005	CARS	0.978	0.005	CHRNB2
0.971	0.004	TMPRSS11D	0.978	0.005	PSMA2
0.971	0.004	DRAM1	0.978	0.004	FYCO1
0.976	0.008	DDX41	0.978	0.005	PPP6R1
0.972	0.005	RPP30	0.977	0.004	RUFY2
0.972	0.004	CLEC11A	0.978	0.005	DLG5
0.972	0.005	NFYB	0.977	0.004	DHRS1
0.971	0.004	LOC220594	0.978	0.005	LSAMP
0.971	0.004	MST1	0.978	0.005	EXOG
0.971	0.004	TMEM45A	0.979	0.006	ERN2
0.971	0.004	FERMT1	0.976	0.003	POP7
0.971	0.004	RAF1	0.977	0.005	CYP24A1
0.973	0.006	MLX	0.978	0.005	CLDN3
0.971	0.005	PSMD6	0.977	0.004	PEPD
0.970	0.004	ENOX2	0.978	0.005	ATG2B
0.971	0.005	IMMT	0.977	0.005	TREX1
0.969	0.003	TMEM140	0.977	0.004	C14ORF169
0.970	0.004	CREB3	0.977	0.004	NDN
0.970	0.004	S100A14	0.977	0.004	MAGED1
0.970	0.004	ERF	0.977	0.005	GMIP

0.970	0.004	ANXA10	0.977	0.004	ZNF358
0.970	0.005	RTN4	0.976	0.004	PAXIP1
0.970	0.004	FUT4	0.977	0.004	POGK
0.969	0.004	KIAA0319L	0.977	0.005	NEBL
0.969	0.004	TRAF2	0.977	0.005	PPM1F
0.970	0.005	FOLR1	0.977	0.004	CXCL13
0.967	0.003	SERPINI1	0.977	0.004	ZBTB38
0.969	0.005	RHCG	0.977	0.005	PDHX
0.968	0.003	TEAD3	0.976	0.004	KDELC1
0.969	0.005	DIEXF	0.976	0.005	EIF4H
0.969	0.004	TEX261	0.977	0.005	PROX1
0.969	0.005	LMO4	0.976	0.004	ENTPD6
0.968	0.004	CASP2	0.977	0.005	PDIA4
0.969	0.005	TRAK1	0.976	0.005	FLG
0.967	0.003	TMEM183A	0.975	0.004	PPIP5K2
0.968	0.004	MRC2	0.976	0.005	TM9SF1
0.968	0.004	STRADA	0.976	0.005	NBAS
0.968	0.004	SPHK1	0.976	0.005	FCGR2A
0.968	0.004	GCNT1	0.976	0.005	SPATA2L
0.967	0.003	APOL1	0.976	0.005	SMARCE1
0.968	0.004	FBXO11	0.976	0.004	TEAD1
0.967	0.004	DLG4	0.976	0.005	NASP
0.969	0.006	BICD2	0.976	0.004	PC
0.971	0.008	KIAA0913	0.976	0.005	PRPS1
0.967	0.004	CNPY4	0.976	0.004	DYNC2H1
0.965	0.003	SEMA3G	0.976	0.005	LY6G6C
0.967	0.004	MEOX1	0.976	0.005	FABP6
0.967	0.004	RAB25	0.976	0.005	FAM189A1
0.969	0.007	BRP44L	0.976	0.005	CADPS2
0.966	0.004	MGST3	0.977	0.006	TLL2
0.966	0.005	THUMPD1	0.976	0.005	CIDEA
0.966	0.004	GABARAPL1	0.975	0.003	UBE2M
0.967	0.005	ERGIC2	0.976	0.005	LDOC1
0.966	0.004	UBXN6	0.978	0.007	MAN1A2
0.966	0.005	MYO1C	0.976	0.005	MANEA
0.965	0.005	CDK9	0.977	0.006	CNTN1
0.965	0.004	BCKDHA	0.976	0.005	ARVCF
0.966	0.006	LOC399491	0.975	0.004	PRRC2B
0.968	0.008	MUC5AC	0.976	0.005	CHODL
0.964	0.004	CEP192	0.975	0.005	SLC5A1
0.965	0.005	ZNF500	0.975	0.005	RPL23AP32
0.965	0.005	RPL27A	0.975	0.004	ALOX15B
0.964	0.005	TMEM33	0.980	0.009	M6PR
0.964	0.005	RPS10	0.975	0.005	RHOT1
0.963	0.005	MAT2A	0.975	0.005	CSTF1
0.961	0.004	MAML1	0.975	0.004	CTSC
0.967	0.010	SPTLC1	0.975	0.005	INF2

0.961	0.005	ZNF238	0.978	0.008	OR1F1
0.961	0.005	HDLBP	0.975	0.005	RGS9
0.961	0.005	RPL12	0.975	0.005	PAQR4
0.960	0.005	EIF2S2	0.977	0.007	DCN
0.961	0.005	KIAA0649	0.975	0.004	SF1
0.965	0.009	PALLD	0.975	0.004	BOLA1
0.960	0.004	FLOT1	0.975	0.005	HIST1H1B
0.959	0.004	HIF1AN	0.975	0.005	PDZRN4
0.961	0.006	ANAPC2	0.975	0.005	GPR153
0.959	0.004	TMEM57	0.975	0.005	MCFD2
0.959	0.005	RPL23A	0.976	0.006	ANK1
0.958	0.005	AIFM1	0.976	0.006	RAB2A
0.959	0.006	RPL13A	0.974	0.005	SMC2
0.957	0.005	RPL36A	0.974	0.005	TMEM176A
0.957	0.005	TUBD1	0.977	0.007	RDH11
0.957	0.005	HMGN1	0.974	0.005	GGT1
0.958	0.006	VEZF1	0.974	0.005	TMEM59L
0.956	0.005	LPGAT1	0.974	0.004	PARN
0.959	0.008	UQCC	0.974	0.005	C14ORF79
0.955	0.004	USP39	0.973	0.004	FN3KRP
0.955	0.005	CACYBP	0.974	0.005	MTSS1
0.954	0.003	CUTC	0.974	0.005	FLJ22184
0.959	0.009	H3F3B	0.973	0.004	SLCO4A1
0.955	0.005	DERL1	0.974	0.005	BDNF
0.955	0.005	RAD23B	0.973	0.004	KCTD3
0.956	0.007	MRPL40	0.973	0.005	APOC1
0.956	0.007	AIMP2	0.973	0.005	VPREB3
0.954	0.005	CGRRF1	0.972	0.005	FZD6
0.952	0.004	ADAM17	0.973	0.005	D4S234E
0.957	0.008	ATP6V1H	0.972	0.005	PMEL
0.952	0.004	C10ORF26	0.973	0.005	PHF20L1
0.952	0.005	ARCN1	0.972	0.004	EGFL6
0.952	0.005	MAPT	0.972	0.005	NUBP1
0.964	0.017	BRD2	0.974	0.007	PPP2R1B
0.951	0.005	SERPINB6	0.972	0.005	PROZ
0.951	0.005	ATP5B	0.974	0.007	SH3GL3
0.950	0.005	RIPK2	0.973	0.006	DNASE1L2
0.950	0.005	KIF18A	0.972	0.005	CSNK2A2
0.950	0.005	BAG6	0.972	0.005	PCBP3
0.947	0.004	EMD	0.971	0.004	LOC100506168
0.948	0.005	SLBP	0.971	0.005	DMXL1
0.947	0.005	MLH1	0.975	0.008	TAPBPL
0.954	0.013	TGOLN2	0.971	0.005	PAF1
0.946	0.005	TOR3A	0.972	0.005	PSKH1
0.946	0.005	SHMT2	0.971	0.005	MUC2
0.950	0.010	SNRNP27	0.971	0.004	ZNF180
0.947	0.006	MARS	0.971	0.005	ARHGAP17

0.948	0.009	DUSP3	0.972	0.006	NUMA1
0.946	0.007	SNX11	0.970	0.004	GTF3C1
0.947	0.008	SUMO3	0.971	0.005	ESPN
0.943	0.005	SIGIRR	0.970	0.005	LPHN2
0.943	0.007	LAMP1	0.971	0.005	DLG1
0.942	0.006	UBE2D3	0.971	0.005	ARSJ
0.941	0.005	TAF5	0.968	0.003	NDUFAB1
0.940	0.005	TIMM17B	0.971	0.006	SCN1A
0.940	0.005	SNX3	0.974	0.008	GIF
0.940	0.005	UCHL3	0.970	0.005	PER3
0.939	0.005	LOC388796	0.973	0.008	HTR1F
0.942	0.009	CDR2L	0.970	0.005	ARHGEF17
0.938	0.005	CHMP7	0.969	0.004	PTPRU
0.936	0.007	TRIM44	0.971	0.006	PRDX2
0.934	0.005	SH3BGRL3	0.969	0.004	SLC22A3
0.938	0.009	MYL6B	0.970	0.006	FAM86C1
0.938	0.009	RANGRF	0.969	0.005	SLC35A1
0.936	0.010	MTX2	0.968	0.005	CORO7
0.937	0.011	FUBP1	0.971	0.008	MAP3K8
0.930	0.004	WFS1	0.968	0.005	TELO2
0.929	0.005	CBFB	0.968	0.005	IBTK
0.932	0.008	ABCF2	0.966	0.003	GPX1
0.934	0.012	PDCD4	0.968	0.005	AQP7
0.927	0.005	ADH5	0.967	0.005	IGF2R
0.926	0.005	FAM171A1	0.968	0.005	C3ORF18
0.929	0.008	RPL22	0.968	0.005	RB1
0.927	0.006	UBA52	0.967	0.005	CHD9
0.932	0.012	UBE2D2	0.967	0.005	VPS33B
0.925	0.005	GTF2A2	0.967	0.005	HOXA7
0.931	0.011	SOX4	0.968	0.006	SCAPER
0.930	0.011	TMEM161A	0.969	0.007	ROD1
0.923	0.005	C9ORF40	0.967	0.005	PPP1CB
0.929	0.013	FAU	0.967	0.005	XBP1
0.921	0.005	MRPL16	0.967	0.005	CALCB
0.921	0.005	MORC2	0.967	0.005	BAIAP3
0.936	0.022	PSMG2	0.966	0.004	ANKRD49
0.918	0.004	WNT5A	0.966	0.004	GADD45G
0.928	0.015	ZNF207	0.966	0.005	ZC3H4
0.920	0.008	MAK16	0.966	0.005	TUBB4
0.925	0.013	KPNA6	0.969	0.007	TNFAIP2
0.922	0.014	PIGG	0.966	0.005	SF3B3
0.917	0.009	SMARCE1	0.966	0.005	ZNF93
0.927	0.020	DOM3Z	0.970	0.008	TUBAL3
0.917	0.012	TMBIM6	0.966	0.005	PLAC1
0.907	0.004	CNPPD1	0.966	0.005	FASTKD2
0.912	0.010	ASPM	0.965	0.005	SCD5
0.908	0.007	C4ORF27	0.964	0.004	RPN1

0.908	0.008	DDX50	0.966	0.005	PRODH2
0.912	0.012	HLA-C	0.964	0.004	AP4M1
0.908	0.010	LSM7	0.967	0.007	FAM125B
0.906	0.010	MRPS18B	0.965	0.005	GNAZ
0.908	0.012	BSDC1	0.964	0.004	CDC42BPB
0.905	0.009	LEPROTL1	0.968	0.008	NPPA
0.908	0.012	TXLNA	0.967	0.007	CXCR4
0.899	0.005	PGRMC2	0.964	0.004	ACADL
0.898	0.005	PFKM	0.965	0.005	GNB2
0.908	0.015	STK25	0.969	0.010	MUC3A
0.897	0.005	PARP4	0.967	0.008	MASP2
0.906	0.014	WDTC1	0.967	0.008	TAF9B
0.914	0.023	TOMM20	0.968	0.009	VIL1
0.899	0.008	CKAP5	0.964	0.005	CDCP1
0.904	0.013	NUDT9	0.965	0.006	HN1L
0.901	0.013	YKT6	0.967	0.008	PLAG1
0.892	0.004	APPL1	0.964	0.005	VAPB
0.893	0.006	SMYD5	0.964	0.005	SLC17A6
0.916	0.031	TTC3	0.964	0.005	NEFH
0.898	0.014	RPL35	0.964	0.005	PSD3
0.900	0.017	SORBS3	0.964	0.005	RAPGEF2
0.895	0.012	PTCD3	0.964	0.005	RSU1
0.892	0.009	WDR18	0.963	0.005	ERCC6L
0.897	0.014	RPS27A	0.963	0.005	TRAF3IP3
0.895	0.013	PTPN3	0.966	0.008	CAPN6
0.901	0.019	ATG4B	0.965	0.007	CAPN5
0.897	0.016	PGRMC1	0.962	0.004	SETD3
0.895	0.014	FAM32A	0.963	0.005	FAM70A
0.890	0.009	UBL4A	0.962	0.005	SRRM1
0.899	0.019	PHB	0.962	0.005	ETS1
0.894	0.015	VBP1	0.962	0.004	SSB
0.891	0.013	RPL30	0.962	0.005	ZNF512B
0.883	0.005	CCDC22	0.962	0.005	TIPARP
0.896	0.017	EIF3G	0.962	0.005	MTHFSD
0.892	0.014	CCNB1IP1	0.961	0.004	CISH
0.893	0.017	MAP4	0.963	0.006	TBC1D9B
0.880	0.007	GNG12	0.964	0.007	MEFV
0.880	0.008	ZNF576	0.965	0.009	ATXN3
0.887	0.015	LRRC59	0.965	0.008	SLC6A7
0.878	0.007	EPB41L2	0.967	0.011	RIMBP2
0.882	0.012	XPO7	0.965	0.009	STOML1
0.893	0.023	PTPN2	0.961	0.005	ARF6
0.883	0.014	NFKBIA	0.961	0.005	TCL1A
0.884	0.019	AQP3	0.961	0.005	LOC100129250
0.882	0.017	NUDT1	0.961	0.005	RLN1
0.883	0.019	STMN1	0.963	0.007	MS4A1
0.886	0.024	SUGP2	0.961	0.005	UBR2

0.884	0.022	LUC7L3	0.961	0.005	SOX3
0.879	0.018	CCNF	0.960	0.005	IVNS1ABP
0.875	0.015	FAM60A	0.960	0.005	FAM63B
0.872	0.014	IARS	0.960	0.005	FBXL4
0.867	0.012	SF3A3	0.964	0.009	PTPN21
0.873	0.019	RPL7A	0.961	0.007	CEBPD
0.874	0.021	FNTA	0.965	0.010	ADRBK2
0.870	0.017	TSPAN3	0.959	0.005	DDIT4
0.870	0.018	NCK1	0.969	0.015	C19ORF2
0.876	0.024	PUS7	0.960	0.007	PIN1P1
0.867	0.016	TCEA2	0.959	0.005	C22ORF29
0.860	0.009	COG4	0.961	0.007	TRIM48
0.859	0.010	EXOSC5	0.958	0.005	HIST1H2AE
0.866	0.016	PTS	0.960	0.007	LASP1
0.865	0.016	FTL	0.957	0.004	BPTF
0.878	0.029	XRCC5	0.965	0.012	DACH1
0.865	0.017	MAST2	0.957	0.004	ENOPH1
0.868	0.021	CERS6	0.956	0.004	DUSP12
0.856	0.012	STX4	0.960	0.008	INSRR
0.862	0.021	ATP6V0E1	0.959	0.007	IL1R2
0.858	0.018	RPL9	0.961	0.009	DNAJC4
0.842	0.008	PTGES2	0.958	0.005	MYO1D
0.850	0.021	ABHD10	0.961	0.008	IL18
0.850	0.023	RPS12	0.959	0.007	C2ORF67
0.852	0.027	RPS4X	0.957	0.005	TOPORS
0.844	0.022	CSK	0.961	0.009	IPW
0.848	0.027	UBE2C	0.957	0.005	POGZ
0.841	0.022	C20ORF111	0.958	0.006	MMP15
0.845	0.029	PSMA2	0.960	0.009	RELN
0.835	0.023	TMEM93	0.956	0.004	EIF2S1
0.830	0.020	RPS13	0.960	0.009	BMP5
0.840	0.031	MED13	0.956	0.005	MIS18BP1
0.822	0.017	LOC100129361	0.957	0.006	DPF1
0.832	0.028	RPLP0	0.958	0.008	CDKN2C
0.827	0.023	EEF1A1	0.954	0.003	SEMA4D
0.832	0.031	TCEB1	0.954	0.004	CRP
0.830	0.028	RPSA	0.955	0.005	EIF3L
0.814	0.014	PLK1	0.955	0.005	TMEM100
0.841	0.041	NFE2L1	0.954	0.005	TMEM132A
0.809	0.013	RRP9	0.958	0.009	WDR25
0.817	0.022	RUFY1	0.958	0.009	ACSBG2
0.830	0.037	SURF1	0.953	0.005	PTCD1
0.822	0.029	RPL23	0.955	0.007	UGT2B28
0.832	0.039	CPT2	0.953	0.005	UBTD1
0.819	0.026	HLA-F	0.953	0.005	IDH3A
0.803	0.014	RAB4A	0.952	0.004	FBXL8
0.817	0.029	MMADHC	0.952	0.004	PGS1

0.810	0.028	TBC1D9B
0.814	0.034	SLC25A46
0.806	0.027	ILKAP
0.805	0.026	SNRNP40
0.810	0.033	PEX11A
0.797	0.024	OXA1L
0.815	0.044	LRRFIP1
0.799	0.030	PSMB8
0.800	0.033	RHOT1
0.801	0.034	RBM6
0.788	0.021	TP53
0.811	0.046	ANAPC5
0.788	0.027	ISOC2
0.794	0.033	EEF1B2
0.784	0.027	RPS29
0.809	0.054	MPDZ
0.780	0.025	RPL6
0.779	0.027	MTCP1NB
0.777	0.025	SESN1
0.789	0.038	NUP62
0.770	0.020	FJX1
0.774	0.027	TCTA
0.771	0.026	PPIE
0.787	0.044	ARL6IP4
0.789	0.048	RPL17
0.772	0.035	DDX56
0.800	0.065	C8ORF33
0.790	0.057	THOC2
0.775	0.043	BLVRA
0.770	0.039	UBE2S
0.763	0.038	BNIP3
0.779	0.054	NKTR
0.766	0.046	RPL38
0.752	0.035	RPL24
0.758	0.045	SMARCA4
0.729	0.018	RRP1
0.736	0.027	HAUS7
0.743	0.035	SNX6
0.743	0.036	DUSP6
0.745	0.050	UQCR11
0.733	0.045	EEF1G
0.756	0.068	HMOX2
0.730	0.047	ECH1
0.741	0.060	HNRNPA1
0.719	0.048	GADD45A
0.732	0.066	PAM16
0.726	0.060	RPS20
0.952	0.004	RFPL2
0.953	0.006	SEC24D
0.952	0.005	SLC25A37
0.957	0.010	INPP4A
0.956	0.010	CASP6
0.950	0.004	C11ORF58
0.960	0.014	PKNOX2
0.954	0.008	ANP32A-IT1
0.955	0.009	RNF186
0.955	0.009	CASP10
0.950	0.004	CKB
0.953	0.008	ANKRD36
0.950	0.005	DNMT3L
0.953	0.007	SMG7
0.951	0.006	FLJ13197
0.951	0.006	NDUFA5
0.956	0.011	RNF24
0.952	0.007	EMR2
0.948	0.004	ACAD8
0.954	0.010	ZNF132
0.949	0.005	SIM1
0.949	0.005	TMEM22
0.958	0.014	ARHGDIA
0.957	0.013	LOC440792
0.952	0.009	TMPO
0.953	0.009	TPD52
0.947	0.004	ITM2B
0.955	0.012	GLI1
0.950	0.007	FOXC2
0.948	0.005	GYG2
0.948	0.005	RXRG
0.957	0.014	CSF2
0.953	0.011	DSC1
0.952	0.010	RREB1
0.947	0.006	ZNF177
0.945	0.004	GMFG
0.946	0.005	EPHA4
0.950	0.009	SLC1A6
0.949	0.008	TPO
0.951	0.010	NMT2
0.947	0.006	L1TD1
0.948	0.008	ZNF493
0.945	0.005	MIOS
0.947	0.007	CD47
0.944	0.004	PSMA6
0.945	0.005	AP1AR
0.945	0.005	SNRPN

0.719	0.059	PDCL	0.951	0.012	FLJ20712
0.720	0.062	SMNDC1	0.950	0.011	NADSYN1
0.702	0.049	RPL13	0.944	0.005	CDX1
0.693	0.041	FLNB	0.945	0.006	EAF2
0.702	0.054	RPS9	0.944	0.005	ZNF43
0.692	0.045	GTF2E2	0.948	0.009	SNAPC3
0.686	0.041	NCK2	0.947	0.009	TTC28
0.686	0.051	PSMD3	0.950	0.012	HNF4G
0.695	0.061	CLSTN1	0.947	0.010	CTNNA1
0.705	0.072	STARD7	0.942	0.005	C4ORF29
0.685	0.053	DCK	0.942	0.005	EFHC1
0.705	0.074	NACA	0.948	0.011	HPS4
0.683	0.053	NMT1	0.945	0.009	KDELR2
0.692	0.066	RPS16	0.947	0.011	TRIM17
0.678	0.055	MAPK9	0.952	0.016	NUCB1
0.682	0.062	CHERP	0.949	0.013	RASL10A
0.664	0.046	NT5DC2	0.946	0.010	INVS
0.676	0.058	PARP2	0.943	0.007	CYFIP2
0.670	0.071	YTHDF1	0.944	0.009	FUT7
0.651	0.053	GEMIN4	0.945	0.010	ACAA2
0.653	0.059	ARFIP2	0.941	0.007	MARK3
0.673	0.081	TSGA10	0.938	0.003	JTB
0.668	0.081	RPS2	0.946	0.012	DZIP1
0.649	0.062	CHKB	0.947	0.013	APC
0.656	0.075	AURKA	0.943	0.009	SCAI
0.633	0.055	GALE	0.941	0.007	SLC9A2
0.651	0.074	CHN1	0.938	0.004	HERPUD1
0.666	0.092	LINC00472	0.945	0.011	KIR2DL1
0.638	0.064	FBXO7	0.945	0.011	PTPN7
0.668	0.098	ATIC	0.953	0.020	GRPR
0.597	0.027	C22ORF36	0.938	0.005	PGF
0.641	0.077	H2BFS	0.941	0.008	CCDC121
0.667	0.110	GAPVD1	0.938	0.005	TCIRG1
0.640	0.083	UBA2	0.939	0.007	HIST3H3
0.605	0.056	FOXO3	0.937	0.005	GMFB
0.627	0.079	PHGDH	0.937	0.005	CITED2
0.640	0.096	DAG1	0.937	0.005	RECQL
0.633	0.089	RPL14	0.946	0.014	ACRV1
0.659	0.116	ZNHIT6	0.941	0.009	EPHA7
0.635	0.093	EHHADH	0.940	0.009	ARAP1
0.617	0.086	PDS5A	0.936	0.005	YEATS4
0.606	0.076	MRPS27	0.935	0.005	HAUS6
0.608	0.086	NENF	0.944	0.013	CDH11
0.588	0.067	ARHGAP15	0.946	0.015	LCN1
0.594	0.085	PLCB3	0.941	0.010	TRIL
0.601	0.105	EPRS	0.935	0.004	TCEB1
0.605	0.111	ATP5G3	0.935	0.005	DBNDD1

0.532	0.038	TRIM17	0.936	0.006	TEP1
0.591	0.100	COL9A3	0.933	0.004	TMPRSS2
0.572	0.083	UBQLN2	0.936	0.007	HLA-DPB1
0.590	0.103	PDCD5	0.939	0.010	LRRC48
0.558	0.077	PRG4	0.933	0.005	PLEKHA8P1
0.555	0.078	DNAJA4	0.940	0.012	DUS2L
0.566	0.090	CLCN5	0.946	0.018	HOXC5
0.548	0.075	PPP2R5A	0.936	0.009	CAPN9
0.577	0.110	PSAT1	0.931	0.003	CTNNBL1
0.576	0.116	CAPNS1	0.931	0.005	DYNLT1
0.595	0.135	PSMA7	0.941	0.015	NAALAD2
0.554	0.096	RNMT	0.942	0.017	FNTB
0.606	0.152	SOD3	0.932	0.007	UBN1
0.557	0.106	DSG2	0.939	0.013	HOXA3
0.550	0.100	RBM15B	0.940	0.015	INA
0.554	0.106	LOC100286895	0.941	0.016	CADM3
0.573	0.127	PPP1R7	0.935	0.011	NEURL
0.539	0.102	CALM3	0.941	0.017	POU2F1
0.514	0.080	APLNR	0.938	0.014	MCM4
0.525	0.094	IER3	0.933	0.010	CREB3L1
0.527	0.098	HERPUD1	0.930	0.007	NVL
0.530	0.102	TMEM5	0.929	0.007	MAGI1
0.552	0.128	MBNL1	0.932	0.009	PRKCI
0.527	0.106	CTNNAL1	0.930	0.008	PPP4R2
0.525	0.105	CEACAM6	0.939	0.017	GUCY2C
0.555	0.138	NUDCD3	0.930	0.008	MOSPD3
0.573	0.156	MPZ	0.926	0.005	NUCB2
0.517	0.101	SLC25A13	0.935	0.014	CD300C
0.536	0.121	PLP1	0.930	0.009	LOC729799
0.527	0.116	WASF3	0.933	0.013	EXOC7
0.550	0.144	UNKL	0.928	0.008	NGLY1
0.525	0.119	KLHDC2	0.926	0.006	LGI1
0.556	0.151	KIAA1024	0.933	0.014	SRSF7
0.523	0.126	S100A13	0.925	0.006	NEDD4L
0.517	0.123	UBR7	0.925	0.007	SRD5A2
0.529	0.137	KRT5	0.932	0.014	GLCE
0.526	0.135	KEAP1	0.925	0.008	HIST1H2BM
0.577	0.188	LOC100127972	0.931	0.013	CSRNP3
0.542	0.155	KDELR2	0.922	0.004	OR51E2
0.503	0.123	XPNPEP1	0.930	0.012	PYGL
0.510	0.135	SLC26A4	0.932	0.014	ATXN7
0.508	0.136	ANXA7	0.929	0.011	GLTSCR1
0.515	0.148	TSN	0.935	0.018	ARRB1
0.519	0.155	NISCH	0.924	0.008	CHST15
0.539	0.179	CD19	0.942	0.026	DMBT1
0.514	0.164	SET	0.926	0.009	PEG3
0.508	0.165	PDK4	0.935	0.019	ILVBL

	0.932	0.016	SATB2
	0.933	0.017	PDGFB
	0.932	0.016	AIM2
	0.920	0.005	PTBP2
	0.926	0.011	ELK4
	0.927	0.012	MBIP
	0.922	0.007	ICAM3
	0.932	0.018	LAT2
	0.928	0.014	GALNT8
	0.930	0.016	MYCN
	0.926	0.013	S100A3
	0.931	0.017	HNF4A
	0.923	0.010	ANKRD17
	0.918	0.005	XPO4
	0.924	0.012	MAN2A1
	0.918	0.006	ANXA2P3
	0.922	0.010	NCKAP1
	0.929	0.017	A4GALT
	0.927	0.016	KCNA4
	0.928	0.017	ACCN1
	0.922	0.012	PDE5A
	0.926	0.016	PPIL2
	0.920	0.011	TMEM231
	0.918	0.009	LRRC8E
	0.929	0.021	SLC16A8
	0.913	0.005	ZNF124
	0.912	0.005	UBE2D3
	0.920	0.013	CHRNA4
	0.922	0.016	ZBTB1
	0.915	0.009	RRAGA
	0.912	0.007	KLK14
	0.923	0.018	TSPAN9
	0.919	0.014	PBX1
	0.925	0.020	SLC26A3
	0.908	0.003	MFAP1
	0.918	0.013	NUP133
	0.923	0.020	GPR182
	0.922	0.021	SPRYD7
	0.908	0.007	WSCD1
	0.905	0.004	TMEM135
	0.913	0.013	PPP1R12A
	0.923	0.023	IFT27
	0.919	0.019	MET
	0.921	0.022	CNGB3
	0.916	0.017	BCLAF1
	0.914	0.016	TAS2R7
	0.914	0.016	PCDHB11

	0.910	0.012	KIF3B
	0.917	0.019	NPIPL3
	0.915	0.018	GPLD1
	0.918	0.021	GAS2L1
	0.917	0.020	PKP2
	0.908	0.012	ATXN7L1
	0.911	0.015	BMP1
	0.910	0.015	GPRC5B
	0.902	0.007	SMC5
	0.899	0.004	CD2AP
	0.900	0.006	FOS
	0.921	0.028	AMPD1
	0.910	0.016	HIST1H3I
	0.912	0.018	SKP2
	0.905	0.012	ZNF185
	0.901	0.009	CCL11
	0.902	0.010	ERG
	0.916	0.025	PAQR5
	0.909	0.017	BRE
	0.911	0.019	ADORA1
	0.900	0.010	B3GALNT1
	0.912	0.022	LINC00483
	0.903	0.013	CDKL1
	0.899	0.010	ALDH7A1
	0.910	0.021	SH2D3C
	0.904	0.017	TYR
	0.902	0.015	KIAA0564
	0.908	0.021	STAG3L4
	0.903	0.016	NTRK1
	0.910	0.023	KAZN
	0.900	0.014	RPH3A
	0.913	0.027	CPLX2
	0.911	0.026	ANGPTL4
	0.898	0.013	LINC00342
	0.895	0.010	DSC2
	0.899	0.015	UCP2
	0.900	0.016	CLDN15
	0.896	0.013	KIAA1109
	0.888	0.005	PRSS8
	0.887	0.005	PPP2R3C
	0.905	0.024	SPAST
	0.897	0.016	NRXN2
	0.899	0.019	RUNX2
	0.892	0.013	PAX6
	0.905	0.025	MLLT10
	0.887	0.008	FAM172A
	0.897	0.018	PRDM2

	0.896	0.018	CACNA2D1
	0.904	0.026	EIF5A
	0.894	0.016	ZFYVE26
	0.897	0.019	NFASC
	0.897	0.020	CHRNA10
	0.901	0.024	C6ORF105
	0.907	0.030	PRKAR1A
	0.890	0.014	FKBP4
	0.896	0.020	TRRAP
	0.897	0.021	DLL3
	0.900	0.025	PEX5
	0.897	0.022	IL24
	0.879	0.005	KIF18A
	0.880	0.006	FKBP1B
	0.892	0.020	DCAF17
	0.892	0.020	TSPAN2
	0.886	0.014	HK1
	0.888	0.016	TERT
	0.891	0.020	AFF2
	0.898	0.027	HNRNPH1
	0.894	0.023	GCK
	0.887	0.017	RAB27B
	0.893	0.024	THBS1
	0.899	0.030	VPS4B
	0.889	0.021	TRPC4
	0.889	0.022	CFHR4
	0.890	0.024	CA3
	0.876	0.011	JUNB
	0.880	0.015	BTN3A3
	0.879	0.016	KCTD9
	0.886	0.022	ZFY
	0.894	0.032	HCRT
	0.896	0.035	NCR3
	0.896	0.035	EMILIN2
	0.880	0.019	SLC45A2
	0.878	0.018	WDHD1
	0.884	0.024	HGD
	0.869	0.009	SMAP1
	0.877	0.019	CCDC134
	0.898	0.040	STXBP5L
	0.883	0.025	HOXD3
	0.888	0.031	CCDC30
	0.879	0.021	RAX
	0.879	0.024	PLA2G10
	0.885	0.030	PSORS1C2
	0.881	0.027	KLF8
	0.869	0.016	PGLYRP1

0.873	0.019	LEP
0.873	0.019	VSTM4
0.882	0.029	ZNF536
0.870	0.018	DKFZP547G183
0.874	0.022	CTBP2
0.873	0.022	ALDH3B2
0.866	0.015	HOPX
0.869	0.019	SEC22A
0.860	0.010	RTN4
0.876	0.026	MAPK10
0.869	0.020	RAD23B
0.879	0.030	SPACA1
0.863	0.014	FAM179B
0.883	0.034	GUCY1B2
0.874	0.025	SLC25A16
0.864	0.015	IL12B
0.870	0.022	MED6
0.886	0.039	FAM198B
0.860	0.014	TOLLIP
0.868	0.022	RAB4A
0.874	0.028	ARFIP1
0.866	0.019	ITPR2
0.865	0.019	SPTLC3
0.872	0.026	ADARB2
0.871	0.025	FAM204A
0.871	0.026	FLJ13224
0.866	0.021	KLRC4
0.872	0.028	SYT5
0.853	0.010	LHFPL2
0.879	0.036	TGFB2
0.867	0.023	SLC2A4
0.881	0.039	BAIAP2
0.866	0.024	SEC14L4
0.863	0.023	CNTN5
0.868	0.028	GJA5
0.860	0.020	SLC26A4
0.864	0.024	SH3TC2
0.844	0.005	PGD
0.865	0.026	B3GALT5
0.861	0.022	HIST1H1T
0.867	0.028	TPM3
0.859	0.020	MGAT4A
0.861	0.023	PCDHGA10
0.860	0.022	SLC13A2
0.859	0.021	MKNK1
0.869	0.031	ATP9B
0.858	0.022	PCDHGA3

	0.863	0.027	FOXN2
	0.868	0.032	CD1D
	0.864	0.029	AGPAT3
	0.854	0.019	NID2
	0.855	0.020	MAK
	0.845	0.011	RIT1
	0.862	0.029	ARFGEF2
	0.868	0.036	UBE2L6
	0.864	0.032	BAIAP2L2
	0.861	0.030	PRSS12
	0.852	0.020	TCN2
	0.855	0.024	PLXNA2
	0.859	0.029	TCHH
	0.861	0.031	FGD2
	0.854	0.024	MDM2
	0.856	0.027	ZNF214
	0.870	0.040	AVIL
	0.853	0.025	PLK4
	0.852	0.024	STAT3
	0.855	0.027	TPD52L1
	0.856	0.030	LOC257152
	0.855	0.028	PURG
	0.871	0.048	BCAT2
	0.853	0.030	DAPK1
	0.856	0.033	CHD4
	0.857	0.034	SCNN1G
	0.857	0.035	QPCTL
	0.828	0.006	TRIM36
	0.840	0.020	MYBL2
	0.838	0.018	RASIP1
	0.851	0.031	L3MBTL1
	0.845	0.026	POU3F3
	0.846	0.028	PTK2B
	0.839	0.021	TNIP3
	0.838	0.020	SPTBN4
	0.853	0.035	CEACAM7
	0.859	0.042	CELF3
	0.838	0.022	SPG21
	0.847	0.031	TMC7
	0.839	0.024	GYPE
	0.838	0.023	C8ORF84
	0.839	0.024	HMGA2
	0.848	0.033	EBF2
	0.840	0.026	CACNA1C
	0.837	0.025	ERGIC3
	0.841	0.031	PTGER1
	0.844	0.034	LIN7C

	0.835	0.025	SCGB1D1
	0.851	0.042	TAGLN3
	0.843	0.034	HIST1H1A
	0.834	0.026	CYP7A1
	0.828	0.021	HYMAI
	0.821	0.014	BRD4
	0.841	0.034	PAR5
	0.833	0.028	TMEM212
	0.845	0.040	ARHGAP24
	0.837	0.032	SOCS7
	0.835	0.033	ARID3A
	0.820	0.019	KIAA0284
	0.836	0.034	EFCAB6
	0.842	0.041	DCAF4
	0.822	0.022	KCNJ15
	0.833	0.033	NKX2-5
	0.834	0.034	POU2F3
	0.838	0.039	RUFY3
	0.836	0.037	GPR85
	0.838	0.039	MYO1B
	0.830	0.032	FAM20B
	0.825	0.028	MCTP2
	0.832	0.034	UPF1
	0.821	0.025	TRA2A
	0.820	0.024	SEPT8
	0.828	0.032	OR7E24
	0.840	0.045	IRS4
	0.817	0.022	PDZD7
	0.831	0.037	KLHL28
	0.833	0.039	CXCL3
	0.821	0.029	ZNF204P
	0.824	0.032	SRSF11
	0.821	0.030	STAMBPL1
	0.819	0.028	LOR
	0.820	0.030	ITGA1
	0.830	0.040	YWHAE
	0.827	0.037	LOC729164
	0.829	0.040	TCTN1
	0.828	0.038	ZNF688
	0.798	0.009	DDA1
	0.826	0.037	CER1
	0.835	0.047	TMCO1
	0.833	0.045	PMP22
	0.824	0.036	CD40
	0.821	0.034	PAX1
	0.820	0.033	POU2F2
	0.818	0.033	GALNT3

	0.819	0.033	FGFR4
	0.808	0.023	USP53
	0.828	0.043	NR5A2
	0.789	0.005	NCAPG
	0.820	0.036	MAPRE3
	0.826	0.042	PP13
	0.836	0.052	PLA2G2F
	0.807	0.023	FSCN2
	0.819	0.036	DERA
	0.813	0.030	GABRG3
	0.810	0.028	LRRC37A4
	0.831	0.051	SEZ6L
	0.816	0.036	HIST1H2BN
	0.809	0.030	HCCS
	0.805	0.026	OR7E14P
	0.812	0.033	ALS2CR8
	0.818	0.039	AHCYL2
	0.821	0.043	LRRC1
	0.808	0.031	VGLL3
	0.819	0.043	VN1R1
	0.827	0.052	PKP1
	0.819	0.044	KRIT1
	0.799	0.025	GNRH1
	0.808	0.035	LBX1
	0.817	0.044	TECTA
	0.806	0.033	CLASP2
	0.800	0.028	BRCA2
	0.813	0.041	CRB1
	0.819	0.048	RAB28
	0.802	0.031	BBS7
	0.800	0.029	MLANA
	0.790	0.020	SEPX1
	0.834	0.065	NR0B1
	0.816	0.047	SLC6A16
	0.803	0.034	ZBTB6
	0.813	0.045	HNRNPD
	0.807	0.039	FOXD2
	0.802	0.035	DOPEY1
	0.810	0.043	ARSD
	0.795	0.028	LIMK2
	0.804	0.037	HOXB8
	0.809	0.044	KCNC1
	0.807	0.042	EPHB1
	0.805	0.040	ICA1
	0.812	0.048	MAGEC3
	0.799	0.035	C11ORF63
	0.800	0.037	ELP4

	0.820	0.057	ZBED4
	0.802	0.039	RFC1
	0.798	0.036	FHL2
	0.816	0.054	TRIM15
	0.798	0.036	CEP97
	0.802	0.040	SEMA3F
	0.805	0.044	RAB14
	0.817	0.056	SLC4A7
	0.816	0.055	C1ORF68
	0.789	0.029	EFCAB1
	0.808	0.047	ZFP2
	0.804	0.045	MINK1
	0.795	0.037	RPS2P45
	0.820	0.062	TTPA
	0.792	0.036	EMX2
	0.796	0.039	TRDN
	0.800	0.044	PYHIN1
	0.789	0.033	GPR77
	0.795	0.039	LCMT2
	0.802	0.047	PVR
	0.790	0.034	LNPEP
	0.790	0.035	SNX13
	0.801	0.047	PBOV1
	0.786	0.033	NFATC4
	0.795	0.042	AFF4
	0.810	0.057	DEDD
	0.820	0.068	FSCN3
	0.798	0.046	PLIN2
	0.790	0.039	EVC
	0.795	0.044	SIK2
	0.782	0.031	SAGE1
	0.758	0.007	ORMDL2
	0.788	0.037	GPR97
	0.807	0.056	FAM115A
	0.786	0.038	BMP4
	0.797	0.049	SOX14
	0.793	0.045	ATOH1
	0.804	0.057	WIZ
	0.791	0.044	COL6A1
	0.793	0.046	SPICE1
	0.800	0.054	NR4A1
	0.802	0.057	PDPR
	0.802	0.058	GOLGA6A
	0.791	0.048	CEACAM1
	0.789	0.046	NOX3
	0.792	0.051	TGM5
	0.781	0.040	AGBL2

	0.782	0.043	GRIN2D
	0.807	0.068	AHI1
	0.785	0.046	PCDHB1
	0.772	0.033	SMG1
	0.782	0.045	NCBP1
	0.792	0.055	NONO
	0.774	0.037	CCDC70
	0.769	0.033	TGFB3
	0.783	0.047	TSHR
	0.793	0.060	MT4
	0.784	0.051	ADRA1B
	0.786	0.053	CD80
	0.773	0.041	TTLL3
	0.776	0.044	PTPRN2
	0.781	0.049	IGHV5-78
	0.800	0.069	IFNA17
	0.772	0.041	IMPAD1
	0.770	0.039	SMC4
	0.771	0.041	COG5
	0.788	0.059	SEC14L5
	0.777	0.048	UBQLN3
	0.780	0.052	TMEM5
	0.777	0.050	AATK
	0.802	0.075	CRCP
	0.766	0.039	FER
	0.784	0.057	HNRNPUL2
	0.775	0.049	DENND4A
	0.773	0.047	SETD2
	0.765	0.041	TMX4
	0.791	0.068	ATXN8OS
	0.779	0.057	CACNB4
	0.759	0.038	ZBTB7C
	0.774	0.054	MYO5A
	0.782	0.063	PRKDC
	0.772	0.053	POLR3G
	0.756	0.037	EIF3M
	0.775	0.058	GGA1
	0.768	0.051	DEC1
	0.769	0.053	SHH
	0.757	0.042	SYK
	0.763	0.047	PKD2L2
	0.774	0.059	VPREB1
	0.754	0.039	CCDC87
	0.763	0.049	C16ORF70
	0.772	0.058	CADPS
	0.781	0.067	MRE11A
	0.762	0.048	HNRPDL

	0.775	0.061	CAMKMT
	0.759	0.046	PDE3A
	0.773	0.060	KRTAP1-3
	0.778	0.065	TP53I11
	0.759	0.047	DCT
	0.771	0.060	SCN7A
	0.765	0.053	KIF2A
	0.780	0.069	COL4A6
	0.747	0.036	COL3A1
	0.758	0.047	EXPH5
	0.778	0.067	TUBGCP5
	0.719	0.008	HSD17B11
	0.766	0.056	CLMN
	0.758	0.048	TRADD
	0.757	0.047	VPS72
	0.761	0.052	TCF3
	0.764	0.055	LMAN1
	0.775	0.065	TMEM144
	0.757	0.047	NOV
	0.749	0.040	DDX17
	0.770	0.061	SERPINB9
	0.760	0.052	PDCD6
	0.746	0.039	ICMT
	0.756	0.050	EFNA2
	0.765	0.061	EP300
	0.750	0.047	RCHY1
	0.771	0.069	EYA3
	0.765	0.063	VSNL1
	0.744	0.043	SLC22A18AS
	0.751	0.050	XCR1
	0.748	0.048	PDK3
	0.759	0.060	HRH3
	0.768	0.071	CLEC5A
	0.752	0.055	ARMC9
	0.758	0.061	H2AFX
	0.743	0.047	MPPED2
	0.763	0.067	TFAP2C
	0.751	0.055	ATP12A
	0.744	0.052	ZNF480
	0.732	0.041	HIST1H2BK
	0.744	0.054	MEOX2
	0.748	0.058	ZNF695
	0.735	0.045	RHOA
	0.740	0.050	TRIM33
	0.749	0.060	PRPF31
	0.745	0.056	TAF4
	0.753	0.065	PER2

	0.747	0.059	PHC3
	0.745	0.058	SAP30
	0.743	0.057	PPEF2
	0.746	0.060	PRX
	0.748	0.064	HCRTR1
	0.748	0.064	ALDH3A2
	0.745	0.062	GFRA2
	0.748	0.066	DKFZP686O1327
	0.754	0.072	RP2
	0.746	0.065	UBAP2L
	0.744	0.064	CEP152
	0.742	0.062	CC2D1A
	0.735	0.055	FUT3
	0.737	0.058	DENND1C
	0.734	0.055	POLR2J4
	0.732	0.054	CCNE2
	0.760	0.082	CCBP2
	0.741	0.065	HSD3B1
	0.735	0.058	PBX2
	0.729	0.053	KLHL11
	0.753	0.077	TAPT1
	0.752	0.077	PCDHGC3
	0.730	0.055	TRANK1
	0.752	0.079	TMEM206
	0.738	0.065	HTR1D
	0.743	0.072	HCN2
	0.734	0.063	C15ORF39
	0.732	0.061	FOXN1
	0.728	0.057	WDR59
	0.749	0.079	MICAL2
	0.716	0.045	PLP2
	0.742	0.072	TSNAXIP1
	0.738	0.067	MAF
	0.727	0.058	IREB2
	0.727	0.058	SLC15A2
	0.714	0.046	LRP4
	0.718	0.050	PRKX
	0.712	0.045	SIDT1
	0.730	0.063	TAOK2
	0.740	0.075	GABRA2
	0.733	0.068	HS3ST3B1
	0.725	0.060	HLA-DRB6
	0.723	0.059	E2F1
	0.736	0.072	POU3F4
	0.732	0.069	FGF23
	0.734	0.071	ALK
	0.736	0.075	LARP4B

	0.730	0.070	PTPRC
	0.685	0.025	UGDH
	0.731	0.073	SDHD
	0.719	0.061	SENP6
	0.738	0.080	EXTL3
	0.712	0.055	ETV1
	0.732	0.076	FSHR
	0.734	0.078	SPN
	0.726	0.074	AQP4
	0.727	0.075	RBMS2
	0.721	0.071	SMCP
	0.713	0.066	FUT2
	0.734	0.088	NUP205
	0.709	0.063	MYB
	0.711	0.067	WWTR1
	0.712	0.069	ADAMTSL4
	0.709	0.067	DDR1
	0.685	0.045	PLA2G15
	0.699	0.061	PDS5A
	0.714	0.077	KHK
	0.722	0.085	LEF1
	0.658	0.021	DERL2
	0.661	0.025	TCFL5
	0.707	0.071	TOM1
	0.658	0.022	HOMER2
	0.714	0.078	LOC51145
	0.723	0.088	MCF2
	0.722	0.088	HRK
	0.704	0.071	NXT2
	0.717	0.085	HBS1L
	0.704	0.072	OLAH
	0.703	0.073	NPY6R
	0.722	0.091	PGCP
	0.712	0.082	GRIN2C
	0.708	0.080	NOTCH3
	0.705	0.079	PDK4
	0.680	0.055	MBOAT2
	0.706	0.081	HIPK3
	0.705	0.080	ARHGAP6
	0.700	0.076	STRN
	0.709	0.085	HOXD1
	0.704	0.081	FOXD3
	0.700	0.077	TSSK1B
	0.707	0.084	ZNF3
	0.707	0.085	ZNF280B
	0.698	0.078	HLF
	0.696	0.076	BCL2

	0.694	0.076	CYLC2
	0.697	0.079	ATXN3L
	0.692	0.077	DHRS7
	0.687	0.073	FOXE1
	0.703	0.090	RASAL2
	0.697	0.086	CNGB1
	0.676	0.066	SMEK1
	0.686	0.080	TOP3A
	0.716	0.110	GRIA1
	0.680	0.079	EP400
	0.694	0.093	EHD2
	0.669	0.069	SHC1
	0.649	0.048	GABARAPL1
	0.692	0.092	FKBP1A
	0.690	0.092	GSPT1
	0.645	0.052	S100A4
	0.686	0.093	TIMM8A
	0.693	0.100	RPL10
	0.644	0.051	CISD1
	0.697	0.104	LRRC37BP1
	0.694	0.102	TLR6
	0.673	0.082	SIGLEC1
	0.677	0.087	CCNT2
	0.690	0.102	PCIF1
	0.669	0.081	POM121L9P
	0.633	0.046	TF
	0.641	0.055	CDH3
	0.673	0.087	AKAP9
	0.678	0.094	LILRA5
	0.678	0.094	SP2
	0.673	0.092	ERC2
	0.687	0.107	ELAC2
	0.668	0.090	AMACR
	0.644	0.066	IKBKAP
	0.676	0.099	RYR2
	0.683	0.106	SMAD4
	0.664	0.089	LRRC23
	0.660	0.086	CUL4B
	0.667	0.095	KIF1A
	0.651	0.079	DCUN1D4
	0.665	0.093	TACSTD2
	0.664	0.094	TUB
	0.670	0.102	HPCAL4
	0.654	0.088	NCAPH2
	0.654	0.088	NUDT7
	0.660	0.095	COL8A1
	0.623	0.059	PSMF1

	0.664	0.099	PDE11A
	0.656	0.092	CFTR
	0.658	0.096	ELOVL5
	0.617	0.055	CCNA1
	0.670	0.109	PCSK2
	0.650	0.089	TEX28
	0.653	0.092	ATF7IP
	0.668	0.109	TRPC6
	0.660	0.101	ULK4
	0.671	0.113	FLT1
	0.645	0.088	CDC42EP4
	0.665	0.108	RFPL3-AS1
	0.651	0.096	KCNH6
	0.651	0.097	SYT1
	0.656	0.102	CD36
	0.662	0.109	GUCA1B
	0.655	0.103	LOC220077
	0.648	0.096	RPL13
	0.641	0.089	DPF3
	0.604	0.053	NOLC1
	0.656	0.106	MUC8
	0.651	0.103	FOXA2
	0.649	0.101	ZNF442
	0.614	0.066	GAA
	0.647	0.100	C16ORF71
	0.632	0.088	FLJ10038
	0.651	0.108	ZBTB7A
	0.647	0.108	ABI2
	0.630	0.092	LCE2B
	0.659	0.120	AHNAK
	0.646	0.109	BCL2L11
	0.651	0.115	SLCO1A2
	0.634	0.101	PDLIM4
	0.630	0.097	SERPINB7
	0.628	0.095	EIF5B
	0.651	0.118	C6ORF10
	0.582	0.049	LOXL1
	0.612	0.081	COG4
	0.643	0.113	GPR52
	0.620	0.092	CLNS1A
	0.624	0.097	PLLP
	0.625	0.099	RUNDC3A
	0.636	0.111	IDS
	0.632	0.107	EPAG
	0.628	0.105	CTIF
	0.636	0.114	TRO
	0.639	0.118	PDE4C

	0.627	0.107	SRGAP3
	0.639	0.119	ACSBG1
	0.640	0.121	FPR2
	0.621	0.103	GALC
	0.631	0.113	SOCS1
	0.636	0.122	RS1
	0.620	0.107	PPP4R4
	0.628	0.116	P2RX1
	0.627	0.115	ARHGEF4
	0.634	0.123	RPS6KA6
	0.576	0.069	KLHL9
	0.618	0.111	PAPOLA
	0.620	0.114	MYL4
	0.625	0.121	NUP88
	0.607	0.104	NCOA3
	0.604	0.102	SLC26A2
	0.608	0.107	FOXG1
	0.616	0.116	PRKAR2A
	0.565	0.065	NME1
	0.610	0.112	NCLN
	0.611	0.114	XIAP
	0.608	0.111	FPR1
	0.620	0.125	ACTR2
	0.611	0.116	VPS13C
	0.606	0.112	CDK17
	0.589	0.100	MPZ
	0.545	0.058	WDR7
	0.614	0.128	TAS2R4
	0.608	0.122	AURKC
	0.581	0.099	ATP6V1D
	0.600	0.119	GPR37
	0.601	0.121	PQLC3
	0.610	0.130	P2RY1
	0.604	0.125	ANK2
	0.588	0.109	CD163
	0.607	0.128	TRIM3
	0.600	0.125	WISP1
	0.604	0.129	KIAA1751
	0.595	0.123	OR52A1
	0.583	0.111	RSF1
	0.585	0.114	EPS15L1
	0.591	0.123	CCL25
	0.586	0.120	KIDINS220
	0.582	0.117	MEG3
	0.587	0.125	CTNS
	0.584	0.122	BRF1
	0.585	0.125	JAK3

0.573	0.120	TCP10L
0.581	0.128	TDRKH
0.576	0.127	IGHA1
0.564	0.116	RNGTT
0.580	0.132	VENTXP1
0.579	0.131	GABRB2
0.582	0.135	SCN11A
0.567	0.120	TRGV5
0.583	0.139	CNTNAP2
0.578	0.134	NIPBL
0.570	0.128	NRXN3
0.568	0.128	PICALM
0.581	0.141	LIMK1
0.534	0.097	NFATC3
0.563	0.126	CHEK1
0.548	0.111	KIAA1324
0.568	0.132	SOCS3
0.576	0.141	PIN4
0.575	0.140	GAB1
0.562	0.129	PPARA
0.566	0.133	LYST
0.570	0.138	REPIN1
0.568	0.140	HUS1
0.562	0.135	DCHS2
0.565	0.138	VIPR2
0.565	0.139	GRIN1
0.565	0.140	MED25
0.544	0.120	GSTM2
0.544	0.122	SAFB
0.562	0.143	DRD3
0.542	0.128	PIP5K1B
0.559	0.145	ACSL6
0.561	0.147	SYDE1
0.568	0.155	FKBP15
0.565	0.152	EZR
0.550	0.137	GJB3
0.565	0.152	C11ORF9
0.554	0.142	KANK1
0.551	0.139	DTX3
0.548	0.137	MVK
0.548	0.138	ASB4
0.540	0.133	ZMYND8
0.544	0.139	CD8B
0.549	0.145	KLRD1
0.543	0.139	SIKE1
0.542	0.139	PCSK6
0.535	0.133	ANKRD12

	0.548	0.148	ESR2
	0.533	0.141	MUSK
	0.535	0.143	ZBTB44
	0.536	0.146	SMARCA1
	0.535	0.148	NEU3
	0.531	0.145	COBLL1
	0.526	0.140	CASR
	0.525	0.142	SYNJ2
	0.530	0.148	ERCC4
	0.526	0.144	ZIM2
	0.528	0.147	P2RX2
	0.524	0.142	KCNMA1
	0.529	0.148	CEACAM3
	0.526	0.145	GRM1
	0.517	0.137	HEBP2
	0.527	0.148	RUNX1T1
	0.516	0.139	CYP2D6
	0.522	0.146	IQCK
	0.507	0.131	MBNL1
	0.511	0.135	EDDM3A
	0.535	0.160	NRXN1
	0.533	0.157	HSPA12A
	0.531	0.157	DCTN1
	0.519	0.146	AKAP6
	0.512	0.141	BRP44
	0.519	0.152	CHRNE
	0.526	0.160	HNMT
	0.549	0.182	NPTN
	0.519	0.152	GLS
	0.523	0.158	SNRK
	0.514	0.151	CD22
	0.508	0.145	RBMXL2
	0.514	0.155	ADCY9
	0.523	0.165	RALGPS1
	0.508	0.153	CAMSAP1
	0.507	0.152	ELK1
	0.514	0.160	SLC25A30
	0.509	0.159	GRK4
	0.506	0.156	ST6GAL1
	0.505	0.158	MAU2
	0.522	0.176	OBSL1
	0.514	0.170	PPFIBP1
	0.509	0.169	KCND3
	0.509	0.170	NCR1
	0.504	0.171	PIGR
	0.508	0.179	MLLT4

Combination prediction result

Name	Overlap count	Up	Down	Result
ITM2B	4	2	2	
KIAA0753	4	2	2	
PDS5B	4	4	0	
RIT1	4	2	2	
SEC24D	4	3	1	
TXNL4B	4	2	2	
ACADL	3	2	1	
AFF4	3	2	1	
AIMP2	3	0	3	
ANKRA2	3	2	1	
ANXA1	3	1	2	
ARFGEF2	3	3	0	
ATP6V0E1	3	1	2	
BANP	3	2	1	
BRD2	3	1	2	
C9ORF40	3	0	3	
CBR3	3	3	0	
CCT6A	3	1	2	
CDCP1	3	2	1	
CDKN2C	3	2	1	
CEACAM6	3	1	2	
CHST15	3	1	2	
COQ9	3	2	1	
COX7A1	3	1	2	
CTSB	3	2	1	
DCAF17	3	2	1	
DHX40	3	1	2	
DIAPH2	3	1	2	
EEF1B2	3	1	2	
EHHADH	3	1	2	
FAM179B	3	2	1	
FAM188A	3	2	1	
FKBP5	3	3	0	
FUBP1	3	2	1	
GABARAPL1	3	2	1	
GFAP	3	1	2	
GLOD4	3	1	2	
HERC1	3	1	2	
HIBCH	3	1	2	
IKBKAP	3	1	2	
IL6ST	3	2	1	
KEAP1	3	1	2	
LIAS	3	1	2	
LINC00342	3	2	1	

LRRC8E	3	2	1	
MAST4	3	0	3	
MDM2	3	1	2	
MTSS1	3	2	1	
NEBL	3	2	1	
NIPBL	3	3	0	
NUBP1	3	1	2	
P2RY1	3	2	1	
PAPOLA	3	2	1	
PARP4	3	0	3	
PDK4	3	2	1	
PER3	3	1	2	
PGM3	3	1	2	
PLK1	3	1	2	
PLP2	3	2	1	
PPP1R12A	3	2	1	
PREP	3	1	2	
PTPN12	3	2	1	
RAB14	3	2	1	
RANGAP1	3	1	2	
RETSAT	3	1	2	
RNMT	3	1	2	
RREB1	3	2	1	
SH3D21	3	1	2	
SLC25A37	3	1	2	
SLC4A7	3	3	0	
SMARCE1	3	1	2	
SPG11	3	2	1	
STK39	3	1	2	
STRADA	3	2	1	
TMEM5	3	2	1	
TRIM2	3	2	1	
UBE2C	3	1	2	
UBE2D3	3	2	1	
UBE2M	3	1	2	
WDR44	3	1	2	
WIZ	3	2	1	
ZFAND1	3	2	1	
ZNF302	3	2	1	
ABHD10	2	0	2	
ACRV1	2	2	0	
ACSL3	2	0	2	
ADCY2	2	2	0	
ADH5	2	0	2	
ADO	2	0	2	
AHCYL2	2	2	0	
AK2	2	0	2	
AKAP9	2	2	0	

ALOX15B	2	2	0	
ALOX5	2	2	0	
AMACR	2	2	0	
ANKRD12	2	2	0	
ANPEP	2	2	0	
AP1S2	2	2	0	
APC	2	2	0	
APPBP2	2	2	0	
ARF6	2	2	0	
ARFIP2	2	0	2	
ARID1A	2	2	0	
ASS1	2	2	0	
ATF6	2	2	0	
ATG5	2	2	0	
ATIC	2	0	2	
ATP6V1C1	2	2	0	
ATP6V1D	2	2	0	
ATP9B	2	2	0	
ATXN7	2	2	0	
BAIAP2	2	2	0	
BNIP3	2	0	2	
BPTF	2	2	0	
BST2	2	0	2	
C6ORF47	2	0	2	
C8ORF60	2	2	0	
CACYBP	2	0	2	
CADPS	2	2	0	
CALM1	2	2	0	
CAMKK2	2	2	0	
CAPRIN1	2	0	2	
CARS	2	0	2	
CASP10	2	2	0	
CC2D1A	2	2	0	
CCNL1	2	2	0	
CD2AP	2	2	0	
CD300C	2	2	0	
CD47	2	2	0	
CD68	2	2	0	
CDC42EP4	2	2	0	
CDH11	2	2	0	
CDK12	2	2	0	
CDK4	2	0	2	
CDR2L	2	0	2	
CEBPD	2	2	0	
CEP152	2	2	0	
CERS4	2	0	2	
CHEK1	2	2	0	
CHRNE	2	2	0	

CIITA	2	2	0	
CLDN15	2	2	0	
CLEC5A	2	2	0	
CNPPD1	2	0	2	
COL4A6	2	2	0	
CPSF7	2	2	0	
CPT2	2	0	2	
CREB1	2	2	0	
CREB3	2	0	2	
CREB3L1	2	2	0	
CRKL	2	2	0	
CSNK2A2	2	2	0	
CTSL1	2	0	2	
CTTN	2	2	0	
CYP1A1	2	0	2	
CYP2E1	2	2	0	
CYTIP	2	2	0	
DBN1	2	0	2	
DDIT4	2	2	0	
DDX17	2	2	0	
DDX3X	2	2	0	
DENND2D	2	2	0	
DKK3	2	2	0	
DLG5	2	2	0	
DNAJB14	2	2	0	
DNAJB2	2	0	2	
DNAJC9	2	0	2	
DNASE2	2	0	2	
DOCK9	2	2	0	
DSG2	2	0	2	
DST	2	2	0	
EEF1G	2	0	2	
EFCAB1	2	2	0	
EFNA3	2	0	2	
EHD3	2	0	2	
EIF3G	2	0	2	
EIF5A	2	2	0	
ELF2	2	2	0	
ELK1	2	2	0	
ELK4	2	2	0	
EMR2	2	2	0	
EPHA4	2	2	0	
ERAP1	2	2	0	
ERCC2	2	0	2	
ERGIC2	2	0	2	
ETV1	2	2	0	
EXOC7	2	2	0	
EZR	2	2	0	

FAM105A	2	2	0	
FAM134A	2	0	2	
FAM198B	2	2	0	
FARSA	2	0	2	
FBXL5	2	2	0	
FBXO21	2	2	0	
FBXO7	2	0	2	
FBXW2	2	0	2	
FKBP4	2	2	0	
FOXA2	2	2	0	
FPR2	2	2	0	
FTL	2	0	2	
FZD7	2	0	2	
GALNT10	2	0	2	
GCLC	2	2	0	
GFOD1	2	2	0	
GNMT	2	2	0	
GOLGA2	2	0	2	
GOLGA3	2	0	2	
GRB7	2	2	0	
GRIN1	2	2	0	
GSK3B	2	2	0	
GSS	2	0	2	
H2BFS	2	0	2	
HECTD3	2	0	2	
HERC2	2	2	0	
HIPK1	2	2	0	
HIST1H1D	2	2	0	
HIST1H1T	2	2	0	
HK1	2	2	0	
HNRNPD	2	2	0	
HPCAL4	2	2	0	
HSD17B10	2	0	2	
HUS1	2	2	0	
ID1	2	2	0	
IER3	2	0	2	
IFIT1	2	2	0	
IGF2R	2	2	0	
IL11	2	2	0	
IL12B	2	2	0	
IL13RA1	2	2	0	
IL1R2	2	2	0	
INPP1	2	2	0	
IQCK	2	2	0	
ITGA5	2	0	2	
ITGB1	2	2	0	
ITGB4	2	2	0	
ITPK1	2	2	0	

KARS	2	0	2	
KAZALD1	2	0	2	
KCTD9	2	2	0	
KDM4C	2	2	0	
KIAA1324	2	2	0	
KLK5	2	2	0	
KLK7	2	2	0	
KRR1	2	0	2	
KRT6A	2	2	0	
LAP3	2	2	0	
LARP4B	2	2	0	
LGI1	2	2	0	
LIN7C	2	2	0	
LINS	2	2	0	
MAN1A2	2	2	0	
MAP3K5	2	0	2	
MASP2	2	2	0	
MAT2A	2	0	2	
MAU2	2	2	0	
MCL1	2	0	2	
MCMBP	2	0	2	
MEF2A	2	2	0	
MEOX1	2	0	2	
METTL21D	2	0	2	
MGAT4B	2	0	2	
MIOS	2	2	0	
MORC2	2	0	2	
MRPL34	2	0	2	
MRPL52	2	0	2	
MRPS27	2	0	2	
MSR1	2	2	0	
MUC8	2	2	0	
MUM1	2	0	2	
MVP	2	2	0	
MYB	2	2	0	
MYBL2	2	2	0	
MYLK3	2	0	2	
MYO1B	2	2	0	
NACA	2	0	2	
NDUFB2	2	2	0	
NEK4	2	0	2	
NEMF	2	0	2	
NF1	2	2	0	
NFE2L2	2	2	0	
NFYA	2	2	0	
NKTR	2	0	2	
NLRP3	2	2	0	
NOS2	2	0	2	

NPPA	2	2	0	
NPPB	2	2	0	
NR1H2	2	2	0	
NR4A1	2	2	0	
NRXN3	2	2	0	
OGT	2	2	0	
OPA3	2	0	2	
OR51E2	2	2	0	
P2RY2	2	0	2	
PANX1	2	0	2	
PARP2	2	0	2	
PCGF3	2	2	0	
PDE5A	2	2	0	
PDGFB	2	2	0	
PDHX	2	2	0	
PEA15	2	2	0	
PEX14	2	0	2	
PFKFB2	2	2	0	
PFKM	2	0	2	
PHB	2	0	2	
PHF10	2	0	2	
PHGDH	2	0	2	
PHIP	2	2	0	
PHLPP1	2	2	0	
PICALM	2	2	0	
PIEZO2	2	0	2	
PIN1	2	0	2	
PLAUR	2	0	2	
PLEKHA1	2	0	2	
PLEKHG3	2	2	0	
PLIN2	2	2	0	
PLLP	2	2	0	
PNP	2	0	2	
PPIE	2	0	2	
PPP1CA	2	0	2	
PRKAR2A	2	2	0	
PRKX	2	2	0	
PRMT5	2	0	2	
PRPF4B	2	2	0	
PRSS22	2	2	0	
PRSS23	2	0	2	
PSG9	2	2	0	
PSMA7	2	0	2	
PSMG1	2	0	2	
PSPH	2	0	2	
PTPN2	2	0	2	
PTPN21	2	2	0	
PYCR1	2	0	2	

PYGL	2	2	0	
QKI	2	2	0	
RAB28	2	2	0	
RAP1A	2	2	0	
RARG	2	2	0	
RB1	2	2	0	
RNASEH1	2	0	2	
RNF24	2	2	0	
RPL22	2	0	2	
RPL35	2	0	2	
RPL6	2	0	2	
RPS2	2	0	2	
RPS9	2	0	2	
RPUSD2	2	0	2	
RRNAD1	2	0	2	
RRP9	2	0	2	
RUFY1	2	0	2	
S100A8	2	2	0	
SAP30	2	2	0	
SCAMP1	2	0	2	
SCNN1G	2	2	0	
SDHAF1	2	0	2	
SECISBP2	2	0	2	
SELL	2	0	2	
SERPINB9	2	2	0	
SET	2	0	2	
SHC1	2	2	0	
SIGLEC7	2	2	0	
SKIV2L	2	0	2	
SLC25A13	2	0	2	
SLC25A46	2	0	2	
SLC39A4	2	2	0	
SLC45A2	2	2	0	
SMAP1	2	2	0	
SMC5	2	2	0	
SNAI2	2	2	0	
SNX16	2	2	0	
SNX6	2	0	2	
SOCS5	2	2	0	
SORBS1	2	2	0	
SORBS3	2	0	2	
SOS2	2	2	0	
SOX2	2	2	0	
SOX4	2	0	2	
SP110	2	0	2	
SPAST	2	2	0	
SPATA20	2	2	0	
SPR	2	2	0	

SPRED2	2	2	0	
SQLE	2	2	0	
SRCAP	2	2	0	
SRGAP3	2	2	0	
SSBP1	2	2	0	
STAMBP	2	0	2	
STAT3	2	2	0	
STAT5B	2	0	2	
STK25	2	0	2	
STK3	2	2	0	
STMN1	2	0	2	
STOML2	2	2	0	
STRN	2	2	0	
SUV39H2	2	0	2	
TAF4	2	2	0	
TARP	2	2	0	
TBKBP1	2	0	2	
TBL3	2	0	2	
TCEA2	2	0	2	
TCF12	2	2	0	
TCIRG1	2	2	0	
TCTA	2	0	2	
TFAP2C	2	2	0	
TFCP2L1	2	0	2	
TJP3	2	0	2	
TLE6	2	0	2	
TMEM144	2	2	0	
TMEM176A	2	2	0	
TMEM97	2	0	2	
TMPRSS2	2	2	0	
TNFAIP2	2	2	0	
TNPO1	2	0	2	
TNS3	2	0	2	
TOMM20	2	0	2	
TOMM22	2	2	0	
TOP2B	2	2	0	
TOPORS	2	2	0	
TP63	2	2	0	
TPP1	2	2	0	
TPSB2	2	0	2	
TRAM1	2	0	2	
TRPC6	2	2	0	
TSPAN3	2	0	2	
TXLNA	2	0	2	
UBE2D1	2	2	0	
UBE2G1	2	2	0	
UBE2S	2	0	2	
UBR2	2	2	0	

UBR7	2	0	2	
UFL1	2	2	0	
UGT2B17	2	2	0	
UHRF1BP1L	2	2	0	
UPF3A	2	0	2	
UQCR11	2	0	2	
USP7	2	2	0	
UTP6	2	0	2	
VPS13C	2	2	0	
WDTC1	2	0	2	
WISP2	2	2	0	
WWTR1	2	2	0	
XIAP	2	2	0	
XPO4	2	2	0	
XRCC1	2	0	2	
ZBTB39	2	0	2	
ZBTB7A	2	2	0	
ZDHHC4	2	0	2	
ZDHHC6	2	0	2	
ZHX2	2	2	0	
ZNF204P	2	2	0	
ZNF442	2	2	0	
ZNF536	2	2	0	

Complex 5

mRNA based prediction result

Pa	Pi	DownRegulation	Pa	Pi	UpRegulation
0.846	0.020	TRAF5	0.881	0.028	AURKA
0.772	0.061	ADORA2B	0.772	0.061	PFKFB2
0.772	0.061	CDCA4	0.772	0.061	SH3BGRL
0.772	0.061	CENPJ	0.772	0.061	TRIM13
0.772	0.061	DNAJC9	0.761	0.064	DNAJB14
0.772	0.061	DONSON	0.711	0.045	GAS6
0.772	0.061	HAUS8	0.724	0.075	CACNG4
0.772	0.061	MCMBP	0.724	0.075	CHRNE
0.772	0.061	NUP155	0.673	0.049	WISP2
0.772	0.061	PARP2	0.694	0.080	SFRP1
0.772	0.061	RMI1	0.686	0.109	PBK
0.772	0.061	SCARA3	0.682	0.105	PPP1CB
0.772	0.061	SLC16A14	0.599	0.024	COX5B
0.772	0.061	SUV39H2	0.634	0.090	KCNH2
0.747	0.047	CAPRIN1	0.634	0.090	PLLP
0.700	0.078	GSS	0.634	0.090	SLC25A4
0.688	0.073	CXCL2	0.634	0.090	ZYX
0.707	0.100	C9ORF40	0.608	0.067	CHEK1
0.586	0.034	CCL5	0.655	0.117	SERPINB8
0.613	0.062	POC1A	0.610	0.079	TRIOBP
0.634	0.090	MAST4	0.620	0.093	SQLE
0.634	0.090	MSH5	0.626	0.108	SLC4A11
0.634	0.090	TREX1	0.607	0.092	KRT6A
0.660	0.118	TMSB15A	0.566	0.061	HMGCR
0.686	0.159	TBC1D9	0.592	0.092	CASP2
0.615	0.097	PPIH	0.611	0.120	UGT2B15
0.609	0.106	NPM1	0.626	0.137	TRIP13
0.562	0.077	DIO2	0.543	0.060	GAL
0.620	0.135	TPM1	0.591	0.109	CMBL
0.504	0.023	HOXA10	0.578	0.104	FADS1
0.582	0.105	NCOA1	0.542	0.069	GNMT
0.600	0.125	PLEC	0.586	0.115	CREB3L1
0.592	0.119	MAPK8	0.586	0.115	RNASEL
0.510	0.041	CAB39	0.587	0.130	COL22A1
0.580	0.112	GPLD1	0.587	0.130	KLK13
0.560	0.096	TANC1	0.540	0.088	HOXA11
0.552	0.100	MYNN	0.527	0.075	ABCA1
0.557	0.112	HPS5	0.596	0.145	GFRA1
0.562	0.117	CXCR7	0.615	0.165	UBE2C
0.546	0.105	ABCC5	0.563	0.119	MCM6
0.552	0.113	CTSL2	0.563	0.119	MCM7
0.552	0.113	GPR19	0.561	0.121	CYP2S1
0.552	0.113	LFNG	0.552	0.113	CNN2

0.552	0.113	MIR20B	0.552	0.113	DYSF
0.552	0.113	NUP54	0.552	0.113	FAM110C
0.552	0.113	PRR16	0.552	0.113	MYO5B
0.552	0.113	SP4	0.552	0.113	PPFIBP2
0.572	0.142	RPRM	0.563	0.128	CDC20
0.559	0.134	YPEL5	0.603	0.172	RACGAP1
0.529	0.111	NDE1	0.534	0.120	BRMS1
0.505	0.099	INCENP	0.553	0.145	MT1L
0.550	0.157	ERCC2	0.535	0.142	WT1
0.513	0.123	HSPA6	0.520	0.150	BLM
0.512	0.147	ZBTB10	0.507	0.154	ST3GAL1
0.540	0.181	RPS19			
0.511	0.212	H1FX			

Protein based prediction result

Pa	Pi	DownRegulation	Pa	Pi	UpRegulation
0.860	0.024	CHEK1	0.672	0.051	NPPB
0.695	0.018	MDM2	0.643	0.055	AR
0.550	0.021	PRKCA	0.534	0.049	CYP3A4
			0.506	0.041	KRT18
			0.516	0.063	PRDX4
			0.516	0.165	CD83

MCF7 based prediction result

Pa	Pi	DownRegulation	Pa	Pi	UpRegulation
0.817	0.017	FOXM1	0.812	0.028	CLDN9
0.783	0.040	LTBP3	0.779	0.014	CDKN1B
0.702	0.039	PTPN18	0.784	0.021	C11orf68
0.742	0.092	UBE2C	0.783	0.025	ZNF672
0.666	0.033	H2AFJ	0.769	0.014	RANGAP1
0.671	0.040	SUCO	0.793	0.038	CLCF1
0.663	0.032	NFE2L3	0.784	0.036	MSRB2
0.709	0.085	C2CD2	0.765	0.027	ABCB6
0.674	0.052	PRRC1	0.717	0.032	PGM1
0.647	0.027	NREP	0.693	0.057	IFIT5
0.663	0.046	CCDC85B	0.669	0.044	GPRC5C
0.663	0.046	KIAA0101	0.656	0.035	SLC4A7
0.645	0.029	KCNG1	0.630	0.028	DNAJB2
0.636	0.023	BBS4	0.652	0.052	PLA2G3
0.649	0.042	ARHGEF10	0.666	0.077	BTG2
0.620	0.028	MRPL34	0.610	0.024	TROAP
0.633	0.042	DPH2	0.604	0.020	RGS17
0.604	0.021	UBIAD1	0.634	0.054	BORA
0.590	0.015	CIAO1	0.664	0.084	CHD3
0.613	0.039	OGFR	0.638	0.060	FOXC1

0.643	0.073	SFSWAP	0.610	0.033	HEG1
0.616	0.048	STC2	0.602	0.030	GCLC
0.609	0.042	SIDT1	0.633	0.063	BNIP3L
0.624	0.057	CPTP	0.589	0.020	LSM14A
0.610	0.046	CHAF1A	0.617	0.049	RAB40B
0.582	0.019	CD3EAP	0.577	0.013	ANGEL2
0.604	0.041	RELA	0.590	0.027	DIP2C
0.612	0.054	SNAPC4	0.606	0.043	TRIM23
0.591	0.037	DIEXF	0.613	0.053	ZNF217
0.625	0.074	SBF1	0.631	0.074	WISP2
0.613	0.070	LPCAT4	0.595	0.039	ZNF211
0.585	0.043	PIK3R1	0.586	0.034	SCAND2P
0.580	0.038	CHAF1B	0.566	0.015	CDS2
0.597	0.056	RBPMS	0.603	0.052	DLG3
0.574	0.035	BCL2	0.604	0.054	CSRP2
0.558	0.020	SMCO4	0.579	0.031	TRMT11
0.553	0.016	GMCL1	0.595	0.048	CLCN3
0.600	0.064	GIT2	0.584	0.039	ZNF451
0.622	0.086	ADCY9	0.593	0.050	ZNF222
0.561	0.025	HGSNAT	0.557	0.014	SPR
0.572	0.036	VDR	0.579	0.036	NIPBL
0.611	0.079	FZD2	0.594	0.055	SCML1
0.590	0.060	ZC3HAV1	0.581	0.043	GABARAPL1
0.567	0.038	PDZK1	0.575	0.040	PIK3IP1
0.570	0.041	NOP2	0.582	0.047	ID3
0.556	0.027	MYD88	0.546	0.014	UBXN4
0.585	0.057	KIAA0368	0.563	0.034	ZFAND5
0.564	0.037	METTL1	0.565	0.036	ADM
0.589	0.067	SLC12A2	0.578	0.050	TXNIP
0.564	0.043	JADE2	0.538	0.011	HSPB8
0.554	0.035	AP1AR	0.559	0.034	STYK1
0.583	0.063	FBXL14	0.566	0.042	PPM1E
0.552	0.033	THAP4	0.560	0.036	STK17A
0.592	0.074	SRSF6	0.553	0.028	PRNP
0.567	0.049	ZFYVE21	0.572	0.049	VAMP4
0.547	0.029	RIBC2	0.559	0.037	ZNF200
0.574	0.056	FLJ10038	0.573	0.052	SLC9A1
0.580	0.063	FKBPL	0.561	0.041	PTRF
0.556	0.039	ICAM3	0.561	0.043	SPAG9
0.596	0.079	PLK1	0.579	0.062	NFKBIB
0.552	0.036	RBM12	0.557	0.044	MINOS1P1
0.570	0.054	SMAD7	0.570	0.057	ZNF329
0.555	0.040	CISH	0.546	0.034	SORBS1
0.561	0.047	MMACHC	0.560	0.048	HIST2H2BE
0.555	0.042	MAST4	0.577	0.066	INTS6
0.600	0.089	EXOSC4	0.554	0.043	SIAH1
0.577	0.065	PANX1	0.543	0.036	DNAJB4

0.547	0.037	FJX1	0.526	0.020	MICB
0.559	0.050	FASTKD1	0.560	0.055	LYPD3
0.560	0.052	PSMD12	0.533	0.029	PHLPP1
0.580	0.073	C1orf174	0.517	0.014	BBX
0.530	0.023	C10orf2	0.520	0.018	PPP3CC
0.544	0.038	THG1L	0.567	0.068	PALLD
0.531	0.024	MREG	0.535	0.038	WDR47
0.541	0.035	URB2	0.552	0.056	N4BP2L2
0.550	0.045	FAM102A	0.526	0.031	UBE2H
0.535	0.031	RET	0.581	0.088	TMEM41B
0.549	0.044	ABR	0.522	0.030	OSBP
0.575	0.071	ESPL1	0.516	0.026	ATXN7
0.551	0.048	AKAP1	0.547	0.058	TFE3
0.582	0.079	HOMER3	0.514	0.027	RSRC2
0.529	0.027	ABLIM1	0.573	0.087	STRN
0.580	0.079	RERE	0.513	0.028	ADGRG6
0.558	0.057	HYAL2	0.552	0.067	DENND5A
0.516	0.019	PRKCD	0.527	0.043	EIF2AK3
0.542	0.046	TNS3	0.522	0.039	BAG2
0.522	0.026	DSCC1	0.504	0.021	BRAP
0.530	0.035	HSPBAP1	0.537	0.054	CLIP1
0.539	0.044	TSEN2	0.521	0.038	PPP3R1
0.546	0.053	FAM3C	0.512	0.029	PCYT1A
0.548	0.056	CDKN2C	0.527	0.045	RANBP9
0.544	0.052	KIAA0125	0.506	0.025	MED6
0.584	0.093	KIF18B	0.520	0.039	SLC5A3
0.540	0.049	INHBB	0.508	0.028	SLBP
0.567	0.077	IRX5	0.502	0.022	SLC39A14
0.531	0.041	PPP1R10	0.538	0.057	SCYL3
0.549	0.059	TCAF1	0.510	0.030	MTUS1
0.542	0.053	METTL21B	0.506	0.027	SQRDL
0.515	0.027	PER2	0.579	0.100	DLX2
0.532	0.047	MYB	0.521	0.043	ZNF654
0.522	0.037	CDC42EP2	0.509	0.032	KDM6A
0.564	0.079	PANK2	0.509	0.032	FERMT2
0.559	0.075	MARCH8	0.502	0.027	MTMR11
0.547	0.063	CCDC101	0.512	0.037	COQ10B
0.612	0.128	PURA	0.578	0.104	ZFP36L1
0.518	0.035	MAP6D1	0.520	0.045	BRF2
0.511	0.028	SACS	0.508	0.033	KDM5B
0.517	0.034	LRIG1	0.503	0.028	FRYL
0.522	0.039	HEATR6	0.518	0.045	MAP3K14
0.550	0.067	PCBP1	0.559	0.088	KCTD5
0.533	0.051	UMPS	0.516	0.044	HIST2H2AA3
0.527	0.045	TST	0.506	0.035	GATA3
0.523	0.042	NAT1	0.537	0.068	FBXL5
0.530	0.049	ASUN	0.509	0.041	UBE2W

0.520	0.040	FAM134A
0.541	0.061	CTPS1
0.509	0.031	GDAP2
0.541	0.063	ZNF22
0.512	0.034	SCO2
0.542	0.065	PATZ1
0.556	0.079	HMMR
0.505	0.029	LCMT2
0.542	0.066	SYNCRIP
0.508	0.034	PWP2
0.513	0.039	MTSS1
0.571	0.097	ZNF85
0.509	0.038	ZNF232
0.540	0.069	ACKR3
0.511	0.040	ASB13
0.512	0.042	MAGI1
0.525	0.055	FAM178A
0.504	0.035	KNOP1
0.508	0.040	NINL
0.519	0.054	FAM172A
0.505	0.040	DUSP7
0.509	0.045	METTL8
0.532	0.069	MAGEF1
0.530	0.066	NDUFAF4
0.502	0.040	SURF1
0.508	0.046	MBD4
0.502	0.040	HK2
0.510	0.049	APEX2
0.510	0.049	MGAT1
0.547	0.087	SFN
0.545	0.086	CDC42EP4
0.512	0.053	ZMYM6
0.507	0.048	DHRS1
0.560	0.102	NR2C2
0.540	0.083	TAF1D
0.503	0.049	ZNF544
0.502	0.051	APBB2
0.516	0.066	FAM111A
0.513	0.063	TACC1
0.511	0.061	MRPS11
0.584	0.137	RRP8
0.501	0.054	STK10
0.507	0.062	ARMCX1
0.513	0.069	UNG
0.503	0.059	SPDL1
0.503	0.059	FGFR2
0.503	0.060	DBP
0.547	0.079	STX3
0.534	0.066	ALDOC
0.549	0.082	NKX3-1
0.537	0.071	ATG12
0.510	0.046	LINC00963
0.531	0.069	NCLN
0.519	0.057	NRP1
0.524	0.064	PROCR
0.556	0.097	KIAA0485
0.510	0.051	FBXO5
0.512	0.056	PSG9
0.513	0.058	TUBA4A
0.531	0.076	PIK3R3
0.539	0.095	DMXL2
0.510	0.074	FLRT3
0.507	0.074	GATA6
0.602	0.175	PTMS
0.530	0.105	FIP1L1
0.522	0.100	PIGA
0.518	0.097	SFPQ
0.530	0.117	PPCDC
0.530	0.117	RRN3
0.505	0.093	ELF3
0.507	0.095	FAM46C
0.516	0.105	PNRC1
0.529	0.125	KLF10
0.527	0.135	KIAA0232
0.514	0.136	FOSL1
0.511	0.192	BIN1

0.517	0.074	ZC3H14
0.511	0.071	ACVR1
0.512	0.072	PCIF1
0.502	0.066	CD44
0.503	0.070	TUBA4A
0.505	0.075	ATXN1
0.538	0.111	ARL4C
0.502	0.075	USP21
0.502	0.077	FUT4
0.505	0.081	SMAD3
0.502	0.078	ATP2A2
0.502	0.079	ARHGEF3
0.525	0.102	TNNT1
0.524	0.103	ADRA2C
0.524	0.104	SEC22A
0.507	0.088	ZC3H13
0.524	0.106	C3orf52
0.507	0.097	IMP3
0.504	0.094	CCNE2
0.502	0.097	GTF3C2
0.518	0.115	ALDH1A3
0.512	0.115	PUS3
0.541	0.145	GTSE1
0.546	0.153	FAM208B
0.541	0.152	RNF4
0.507	0.118	PCBP2
0.506	0.126	ZC3H7B
0.527	0.157	ANKRD12
0.505	0.150	C2orf42

VCAP_6h based prediction result

Pa	Pi	DownRegulation	Pa	Pi	UpRegulation
0.908	0.024	BAG2	0.924	0.005	WBP2
0.909	0.030	POU3F2	0.913	0.009	ZFAND1
0.888	0.022	TRAIP	0.897	0.004	NGFRAP1
0.858	0.006	C6ORF47	0.879	0.023	CA2
0.890	0.042	ALMS1	0.874	0.022	EMG1
0.872	0.031	RPL23AP53	0.850	0.011	KIAA1324
0.846	0.013	TUT1	0.794	0.014	TMEM50A
0.862	0.034	MRPL24	0.825	0.048	GREM1
0.845	0.022	CAD	0.806	0.030	NCAPD3
0.838	0.035	KLHDC4	0.768	0.014	TMEM93
0.843	0.047	BRE	0.761	0.050	MMP3
0.802	0.008	LIPC	0.724	0.042	IRF7
0.816	0.029	BPIFA1	0.727	0.067	IPO13

0.829	0.056	CEMP1	0.692	0.050	DHX15
0.813	0.047	CACNA1B	0.678	0.047	TUBB2A
0.789	0.033	DUX1	0.703	0.074	INTS7
0.806	0.052	PCP4	0.722	0.094	MMD
0.787	0.041	CXORF56	0.729	0.108	OSTM1
0.776	0.041	ZNF221	0.750	0.132	MCAT
0.765	0.031	CTSG	0.714	0.097	TTC33
0.733	0.007	C10ORF95	0.695	0.080	PRC1
0.775	0.051	REPS2	0.671	0.066	TXN2
0.737	0.016	KLK7	0.699	0.104	MRPL4
0.724	0.015	AGPAT3	0.670	0.081	GMEB2
0.741	0.035	PCBP4	0.629	0.044	TRIM36
0.773	0.069	PROX1	0.649	0.066	BFAR
0.774	0.072	SELL	0.652	0.073	INSIG1
0.765	0.065	IPCEF1	0.655	0.077	IFI6
0.739	0.047	TBC1D1	0.632	0.057	TERF2IP
0.791	0.111	CRYGD	0.643	0.077	UNC50
0.764	0.087	FRG1	0.687	0.123	PRMT1
0.756	0.081	ROR1	0.629	0.081	SPDEF
0.722	0.050	ALDH1L1	0.669	0.122	PSMD7
0.739	0.068	ELF3	0.657	0.114	SLC38A2
0.701	0.031	AURKA	0.640	0.098	ACVR2A
0.712	0.052	UFSP2	0.623	0.093	CENPA
0.699	0.044	COL4A3BP	0.567	0.053	GRB7
0.750	0.097	MGC12488	0.604	0.097	NIF3L1
0.723	0.075	ACSL4	0.595	0.090	USP3
0.717	0.074	SSBP1	0.621	0.118	KPNA2
0.696	0.056	ADD1	0.577	0.074	PIK3CA
0.724	0.085	HLCS	0.603	0.101	CCDC91
0.697	0.061	ST7	0.611	0.108	HIST3H2A
0.698	0.064	EPHB2	0.562	0.063	LBH
0.689	0.057	LSM3	0.529	0.031	ABCF3
0.708	0.088	VAMP3	0.602	0.108	SGPP1
0.684	0.073	KCNJ12	0.570	0.081	HMGN2
0.691	0.081	TPBG	0.603	0.116	NDE1
0.709	0.103	PMS2P5	0.565	0.078	MCM6
0.673	0.069	TIPIN	0.614	0.132	BABAM1
0.691	0.090	C10ORF2	0.601	0.119	MRPL16
0.665	0.068	PPP2R2A	0.574	0.095	MX1
0.672	0.080	EYA2	0.561	0.113	PNMT
0.681	0.090	DRG1	0.532	0.086	TMSB15A
0.677	0.086	ZNF587	0.551	0.109	ARFIP2
0.635	0.054	TFCP2L1	0.610	0.172	MOSC1
0.645	0.065	IRX5	0.547	0.110	ABHD3
0.620	0.042	KIF15	0.531	0.098	CBR3
0.664	0.087	NIT2	0.557	0.125	IL32
0.698	0.123	EHBP1	0.554	0.123	ALAS1

0.636	0.061	LTBP2
0.681	0.107	ZNF253
0.660	0.087	SCAMP1
0.683	0.115	KCNS3
0.637	0.079	PAAF1
0.644	0.088	POLQ
0.611	0.054	POLR3D
0.627	0.072	CYP26B1
0.620	0.072	RRP1
0.656	0.108	KIF13B
0.655	0.108	EVI2B
0.650	0.104	CLCN2
0.621	0.081	STEAP1
0.614	0.076	ROBO4
0.642	0.109	GFRA2
0.603	0.075	WNT11
0.645	0.119	C20ORF103
0.587	0.060	CDA
0.585	0.071	C20ORF46
0.591	0.081	ZNF552
0.657	0.155	AHNAK
0.603	0.106	IKBKB
0.572	0.076	HIST1H4B
0.606	0.111	DARS
0.628	0.133	LOC100508797
0.582	0.093	PARL
0.598	0.115	PDLIM1
0.574	0.090	KIAA1109
0.594	0.116	FGL1
0.562	0.084	KIAA0754
0.597	0.124	MAP3K5
0.597	0.124	ODF1
0.558	0.087	TEAD1
0.591	0.122	ERLIN2
0.548	0.081	PPFIA1
0.568	0.104	CGREF1
0.588	0.128	WHSC1
0.568	0.111	ZNF516
0.605	0.149	SLC25A37
0.550	0.095	FMNL1
0.589	0.137	C1ORF114
0.585	0.136	P2RY13
0.562	0.113	ENPP1
0.613	0.164	ART3
0.596	0.148	C2CD2
0.549	0.106	URGCP
0.619	0.176	SERPINB7
0.570	0.145	RNF13
0.555	0.132	LMO3
0.510	0.088	GNE
0.541	0.124	SUN2
0.549	0.136	ICT1
0.541	0.132	PCTP
0.570	0.169	COPS7B
0.533	0.132	FAM114A1
0.535	0.144	SLC31A2
0.518	0.134	RECQL
0.540	0.156	AKAP8L
0.535	0.152	ARL6IP4
0.554	0.174	CTSK
0.526	0.154	RAB32
0.514	0.147	OSBPL1A
0.503	0.140	MEX3C
0.532	0.170	C7ORF68
0.522	0.162	AES
0.513	0.155	MAP1LC3B
0.507	0.151	KCTD13
0.507	0.166	SMARCA5
0.527	0.198	SCMH1
0.503	0.188	MTMR14

0.572	0.130	DCUN1D1	
0.561	0.122	LOC644450	
0.552	0.115	DNM1	
0.570	0.135	LHX2	
0.580	0.144	ADM	
0.521	0.087	ITGA2	
0.511	0.079	AMPD2	
0.532	0.101	C14ORF133	
0.508	0.078	DHRS2	
0.541	0.111	NFKB1	
0.597	0.168	PRRC2B	
0.548	0.125	A4GALT	
0.544	0.121	TLR2	
0.570	0.148	ATP13A2	
0.560	0.141	UBE2L3	
0.564	0.147	KNG1	
0.560	0.145	RIN1	
0.502	0.093	BAGE	
0.528	0.119	PPWD1	
0.567	0.159	GTF2H2B	
0.563	0.156	TP53TG5	
0.517	0.111	KIAA0146	
0.541	0.135	PTPRG	
0.522	0.117	GALNT11	
0.540	0.137	TLK1	
0.565	0.163	IL2RG	
0.560	0.162	CETP	
0.564	0.167	SEMA5A	
0.547	0.151	DDHD2	
0.520	0.126	PRRG2	
0.523	0.131	FICD	
0.542	0.154	LOC81691	
0.502	0.116	MLF1	
0.511	0.125	CBR1	
0.501	0.119	CRTAM	
0.529	0.149	B4GALNT1	
0.521	0.147	HNRPDL	
0.558	0.185	KIAA0513	
0.540	0.174	LASP1	
0.531	0.167	ZNF227	
0.511	0.148	RUFY2	
0.518	0.156	KANK2	
0.514	0.153	SERPINI1	
0.515	0.157	KPNA5	
0.521	0.162	LOC646934	
0.542	0.191	MAGEA11	
0.524	0.174	CENPC1	

0.506	0.163	MUC16
0.527	0.184	ENC1
0.507	0.164	NDP
0.536	0.194	HHAT
0.502	0.161	UPF2
0.529	0.189	KCNC2
0.538	0.200	HLA-DMA
0.502	0.178	WDR44
0.504	0.184	PIK3CG
0.502	0.185	TCHH
0.538	0.233	EBF2

VCAP_24h based prediction result

Pa	Pi	DownRegulation	Pa	Pi	UpRegulation
0.941	0.005	IFT52	0.977	0.007	NPFFR1
0.941	0.010	UBE2O	0.941	0.012	EXTL1
0.930	0.009	RAD23A	0.930	0.010	PRRX1
0.924	0.020	MRPL49	0.933	0.016	SLC12A7
0.920	0.025	CLASRP	0.919	0.011	HIST1H2AJ
0.899	0.010	MLPH	0.916	0.010	CEP170
0.890	0.005	ACTR1B	0.916	0.013	PGS1
0.901	0.029	GCN1L1	0.913	0.013	ZNF324
0.880	0.008	GDI2	0.908	0.009	C19ORF60
0.881	0.009	CLDN1	0.907	0.009	C19ORF22
0.895	0.024	RBMX2	0.905	0.009	SNX16
0.882	0.020	HIF1AN	0.907	0.013	ATP2A3
0.869	0.008	TSPAN1	0.915	0.022	OLFM1
0.883	0.024	ABR	0.909	0.016	SND1
0.875	0.019	C15ORF44	0.911	0.020	RNF31
0.884	0.031	ANO1	0.904	0.015	CSNK1G3
0.868	0.016	SYBU	0.883	0.004	CIT
0.854	0.014	GTPBP3	0.886	0.010	KLK14
0.849	0.013	EPB41L2	0.894	0.018	NAT6
0.860	0.029	HPRT1	0.895	0.022	HLF
0.863	0.033	CLASP1	0.897	0.028	TBC1D12
0.837	0.012	NUP43	0.878	0.010	PLS3
0.851	0.031	KAT8	0.883	0.017	ITM2B
0.828	0.012	TSGA10	0.874	0.008	ACOT8
0.847	0.034	RBM7	0.887	0.027	DEXI
0.844	0.039	C16ORF7	0.878	0.019	PSENEN
0.809	0.005	NRN1	0.876	0.021	MPDZ
0.851	0.049	SETD1A	0.869	0.019	CARHSP1
0.850	0.051	PIAS3	0.863	0.014	DYNLT1
0.844	0.047	CEP85	0.878	0.033	CORO2B
0.835	0.042	EIF2B1	0.864	0.019	ZRSR2
0.838	0.044	SLC26A4	0.853	0.011	CCNB1

0.817	0.025	NUP107	0.874	0.032	CYR61
0.799	0.010	EHHADH	0.879	0.039	PDK2
0.811	0.022	IMPACT	0.866	0.027	CTNND2
0.851	0.063	EDEM2	0.874	0.037	TOX
0.831	0.043	P2RY2	0.863	0.027	ZNF783
0.830	0.042	NIPA2	0.866	0.031	NCDN
0.819	0.032	MORF4L2	0.843	0.009	KIF18A
0.818	0.032	ZNF473	0.869	0.039	OGN
0.820	0.037	SHMT2	0.850	0.022	PP14571
0.811	0.029	MRPL15	0.856	0.029	PEG3
0.806	0.032	AIFM1	0.841	0.016	FAM179B
0.790	0.017	AHNAK2	0.853	0.029	TRDV3
0.782	0.011	DAK	0.857	0.034	BMP5
0.829	0.059	KIAA1024	0.863	0.044	LTK
0.798	0.035	GDAP1L1	0.845	0.027	MFAP2
0.806	0.043	ARC	0.859	0.041	RAB11B
0.785	0.022	NOC3L	0.836	0.019	HIST1H2BI
0.791	0.031	EPRS	0.866	0.051	LCK
0.800	0.041	CGA	0.843	0.029	MAPRE3
0.780	0.022	RWDD2B	0.844	0.032	IFT88
0.791	0.033	ABCA3	0.853	0.043	AICDA
0.800	0.043	CCDC41	0.845	0.038	ISG15
0.785	0.034	ELF3	0.833	0.026	PIK3R2
0.783	0.032	KRT3	0.835	0.029	AQP8
0.793	0.043	SSR3	0.821	0.016	PCTP
0.779	0.030	SDAD1	0.828	0.025	JUNB
0.785	0.036	MAPT	0.821	0.017	DBI
0.791	0.047	PCYOX1L	0.813	0.010	CENPF
0.780	0.038	CS	0.832	0.033	CHMP4A
0.786	0.049	NUBP1	0.826	0.028	RSAD2
0.780	0.044	CKAP5	0.830	0.033	OSTF1
0.773	0.038	IARS	0.830	0.034	UBE2D3
0.771	0.037	IRX5	0.811	0.015	TOX3
0.797	0.063	MAP1LC3C	0.832	0.037	DGCR2
0.764	0.031	C16ORF88	0.818	0.024	SHARPIN
0.759	0.029	HOXC10	0.820	0.026	RGS5
0.753	0.023	LARS	0.840	0.049	DHX40
0.773	0.044	EIF4H	0.816	0.026	CSF2RB
0.770	0.041	CTCF	0.801	0.014	CMAS
0.752	0.024	ARHGAP4	0.822	0.035	SCARF1
0.765	0.037	ENPP2	0.819	0.034	MEST
0.756	0.030	PFAS	0.825	0.041	IFI44L
0.739	0.014	CLIC3	0.824	0.039	CD244
0.759	0.035	VSNL1	0.807	0.024	ANXA2
0.761	0.037	TMBIM4	0.820	0.038	GP1BB
0.745	0.023	SERPINE2	0.827	0.045	QPCTL
0.757	0.037	SCMH1	0.791	0.010	ITGAV

0.846	0.127	NTRK2	0.825	0.050	HPS6
0.755	0.036	SERPINI1	0.811	0.036	WNK1
0.782	0.067	IQCE	0.807	0.033	BPTF
0.774	0.060	MGLL	0.806	0.037	MT1E
0.773	0.062	GPN3	0.787	0.020	ADM
0.733	0.024	RAB4A	0.795	0.029	MATK
0.752	0.047	NIF3L1	0.810	0.045	NEBL
0.756	0.055	RPS6	0.792	0.028	IRF7
0.750	0.051	TBKBP1	0.797	0.033	FOXC2
0.736	0.037	SESN1	0.779	0.017	CD2AP
0.738	0.039	DNAJA4	0.819	0.057	ADRBK2
0.759	0.061	RELA	0.799	0.038	EGR4
0.751	0.054	AAGAB	0.798	0.037	MAP3K9
0.731	0.035	DPF1	0.769	0.010	TPX2
0.724	0.029	PFKM	0.796	0.038	KIF26B
0.755	0.063	PIGF	0.803	0.045	PTGER1
0.755	0.064	TCTN3	0.805	0.047	POU2F1
0.754	0.065	ACADS	0.801	0.043	PPIB
0.745	0.057	SH3D21	0.800	0.046	CDON
0.748	0.060	PLEKHB1	0.848	0.095	IGFBP2
0.753	0.066	FAM5C	0.796	0.045	B3GAT1
0.750	0.064	TPD52L2	0.798	0.047	PRKG1
0.746	0.060	WNT5A	0.793	0.043	ABCA11P
0.733	0.048	MAP1S	0.794	0.045	NUP50
0.730	0.045	NLE1	0.836	0.088	PDIA3
0.763	0.081	RIN2	0.803	0.055	CYP11B2
0.742	0.061	EIF2S2	0.787	0.041	CFB
0.725	0.049	FPGS	0.770	0.024	CYB561D2
0.732	0.057	MUC4	0.757	0.011	NCAPG
0.747	0.075	PDHA1	0.798	0.056	PEX16
0.698	0.026	TINF2	0.783	0.042	TOLLIP
0.713	0.042	RPH3AL	0.778	0.039	PCDHGA9
0.695	0.025	PALB2	0.791	0.052	SMYD3
0.720	0.051	COPG	0.787	0.049	CHAC1
0.720	0.053	LACTB2	0.781	0.043	C12ORF11
0.732	0.065	ANXA3	0.778	0.041	HIST1H2AI
0.734	0.067	KCTD5	0.803	0.067	RAB38
0.732	0.067	TTF1	0.773	0.037	FER
0.728	0.063	FLOT1	0.767	0.033	MKI67
0.713	0.049	CST3	0.786	0.052	ZNF280D
0.728	0.064	ADCY7	0.778	0.045	HAPLN2
0.728	0.065	CCHCR1	0.789	0.056	ARHGEF7
0.708	0.046	TIMM17B	0.781	0.048	CRISPLD2
0.725	0.063	ASPM	0.779	0.049	APOH
0.706	0.045	VCAM1	0.768	0.040	SMC4
0.723	0.063	RPL13A	0.769	0.041	AURKA
0.743	0.083	SULT1C2	0.778	0.053	CNGB1

0.719	0.060	MAK16	0.738	0.016	KIF23
0.710	0.051	SLPI	0.764	0.043	SLC35E3
0.744	0.087	SETD1B	0.757	0.036	DPY19L4
0.736	0.079	SF3A1	0.770	0.050	TSC2
0.719	0.063	UBA52	0.748	0.028	SAT1
0.728	0.072	TMEM180	0.776	0.058	SRGAP2
0.727	0.073	DDX24	0.777	0.059	CRB1
0.686	0.033	IFITM3	0.749	0.033	FOS
0.691	0.038	ALG3	0.769	0.053	HELLS
0.677	0.024	AZI1	0.766	0.052	TRPV6
0.710	0.058	FLNA	0.780	0.067	ARVCF
0.697	0.045	LOC100286895	0.756	0.043	HBXIP
0.698	0.047	C6ORF108	0.766	0.052	GP9
0.701	0.050	PRR7	0.763	0.050	APOBEC3B
0.724	0.075	PASK	0.786	0.073	LMO3
0.730	0.083	CLIC1	0.773	0.061	C14ORF132
0.707	0.060	CEACAM6	0.746	0.035	C2ORF28
0.700	0.054	B3GNT1	0.770	0.059	ZNF467
0.720	0.074	SLC6A6	0.780	0.071	SLC26A3
0.739	0.094	DLL3	0.746	0.037	FAM69A
0.719	0.076	FABP3	0.756	0.047	CCNA2
0.697	0.054	SKIV2L2	0.787	0.080	SAFB2
0.715	0.075	FAM125B	0.774	0.068	EIF2B2
0.709	0.069	AIMP1	0.765	0.059	ZMPSTE24
0.715	0.076	PNP	0.779	0.075	TAGLN3
0.696	0.057	PPRC1	0.745	0.041	TIPARP
0.694	0.056	WDR45	0.750	0.046	PTBP2
0.713	0.075	DNASE1L1	0.760	0.059	ZBED5
0.704	0.066	RUNDC3A	0.751	0.050	CRYBA2
0.691	0.054	PRPSAP2	0.745	0.045	LPPR1
0.693	0.057	PARP4	0.755	0.056	GLI1
0.679	0.043	GALNT6	0.743	0.045	C14ORF109
0.697	0.062	C20ORF103	0.742	0.044	PDZD7
0.702	0.067	LINC00472	0.756	0.059	UBB
0.678	0.044	SGPP1	0.768	0.075	TGFB2
0.688	0.056	PDS5A	0.764	0.074	LOC100131532
0.705	0.073	CLDN18	0.736	0.046	TARP
0.678	0.046	POLD2	0.744	0.055	VPS53
0.722	0.090	DSG3	0.735	0.049	CCL11
0.704	0.073	AKR1B10	0.769	0.084	KIAA0182
0.681	0.051	DIXDC1	0.741	0.057	POU6F1
0.693	0.066	MED15	0.696	0.012	DLGAP5
0.695	0.070	RNF187	0.748	0.065	DYNC2H1
0.693	0.069	ABCG1	0.767	0.088	MZB1
0.701	0.078	MRPL16	0.746	0.068	SLC13A4
0.712	0.089	CHMP7	0.720	0.043	ICAM2
0.637	0.017	TRIM17	0.728	0.051	RCHY1

0.680	0.059	C20ORF4	0.733	0.057	AP3S1
0.706	0.087	ELANE	0.685	0.010	FOXM1
0.696	0.077	DAZAP2	0.728	0.053	SMC5
0.672	0.058	CD3EAP	0.736	0.062	TSSK2
0.691	0.077	C12ORF41	0.735	0.061	DOC2B
0.690	0.076	SLC7A10	0.730	0.058	HCCS
0.693	0.080	ZNF32	0.734	0.062	OAS1
0.671	0.058	FADS3	0.746	0.075	PDE4C
0.676	0.064	CCNB1IP1	0.734	0.064	CTBP2
0.697	0.084	DDX18	0.744	0.074	HIST1H2AB
0.651	0.039	RBM28	0.743	0.074	TCN2
0.672	0.061	ATR	0.731	0.062	MARCH6
0.680	0.069	PLEKHA1	0.710	0.042	FAM172A
0.691	0.082	SMARCC2	0.750	0.084	KIAA1310
0.669	0.061	OXA1L	0.742	0.077	DNM2
0.687	0.080	PDK4	0.717	0.054	SLC35D2
0.663	0.056	PTGES2	0.676	0.013	KIFC1
0.660	0.054	CHKB	0.710	0.050	XBP1
0.689	0.084	TPM4	0.700	0.043	SYNJ2BP
0.672	0.068	ECH1	0.720	0.062	MAK
0.686	0.083	SLC30A3	0.684	0.028	RDH14
0.675	0.073	DNAJC13	0.714	0.059	LRP5L
0.670	0.069	C1ORF61	0.713	0.058	TRA2A
0.678	0.078	U2AF1	0.724	0.070	ARL5A
0.660	0.060	DCK	0.729	0.075	HEG1
0.654	0.054	CHST15	0.670	0.017	ASPM
0.675	0.078	KDM2A	0.705	0.053	ROBO1
0.688	0.094	CUTC	0.705	0.054	GSTK1
0.711	0.120	TIMP4	0.720	0.069	RFPL2
0.668	0.077	BBS7	0.722	0.071	ZDHHC17
0.659	0.069	COPS7A	0.703	0.054	ELAVL3
0.648	0.058	TLR2	0.704	0.055	CCDC87
0.671	0.085	C19ORF42	0.681	0.033	DAD1
0.679	0.095	GNAL	0.729	0.081	CTNNA1
0.633	0.049	TMEM168	0.708	0.060	KIAA1797
0.648	0.066	LARP4	0.731	0.084	MED4
0.661	0.079	TMEM9B	0.702	0.056	CHI3L2
0.643	0.062	GAP43	0.684	0.037	KIF20A
0.632	0.052	PPL	0.718	0.071	IGLL3P
0.646	0.067	POF1B	0.705	0.058	COL5A1
0.657	0.078	SLC5A6	0.689	0.043	GALE
0.623	0.044	PIGB	0.705	0.059	TRIM33
0.632	0.054	CLCN5	0.713	0.067	ELL
0.657	0.079	PDIA4	0.714	0.069	PBX2
0.614	0.037	DCAF17	0.716	0.072	CD38
0.659	0.083	SPINT1	0.698	0.055	LHX1
0.654	0.078	ZHX2	0.709	0.066	GPRC5B

0.600	0.025	C22ORF36	0.728	0.086	NR4A1
0.649	0.074	ADAM10	0.712	0.070	CPB2
0.637	0.063	CIDEB	0.727	0.086	HS6ST1
0.656	0.082	COL9A3	0.689	0.050	F5
0.636	0.063	DMRT1	0.737	0.099	TREM2
0.665	0.092	OPHN1	0.719	0.081	CLDN5
0.643	0.075	RPS13	0.713	0.075	CYP2A13
0.639	0.072	PSMD3	0.731	0.093	MUC2
0.649	0.081	UGT2B28	0.682	0.045	HIST1H2BC
0.646	0.078	TMBIM6	0.715	0.078	LY6G6C
0.670	0.103	ZNF200	0.691	0.055	CORO2A
0.634	0.069	ABCB4	0.705	0.070	MGC12488
0.640	0.076	INTS3	0.707	0.073	ACAP1
0.644	0.082	MPPE1	0.661	0.027	FKBP1B
0.659	0.098	EIF2D	0.734	0.100	KLK5
0.658	0.098	MUC5AC	0.723	0.089	VPS41
0.618	0.058	SLC41A3	0.695	0.063	JTB
0.664	0.106	UBL4A	0.704	0.073	RAI1
0.656	0.098	PTPN11	0.693	0.063	USP32
0.624	0.066	MTMR11	0.719	0.088	SHOX2
0.632	0.080	RPL27	0.695	0.065	ANKRD12
0.622	0.071	ZNF219	0.717	0.087	NCR2
0.626	0.076	APPL1	0.703	0.075	RECQL
0.636	0.087	RPS29	0.711	0.085	ACAD8
0.608	0.058	PGAM2	0.708	0.083	SMAD2
0.606	0.058	AARS	0.683	0.058	COL4A4
0.631	0.083	DPYSL2	0.705	0.081	SUPV3L1
0.637	0.089	RUVBL1	0.703	0.080	PDLIM4
0.619	0.071	RPL13	0.694	0.071	POLR2J4
0.627	0.079	COL11A1	0.676	0.055	DDA1
0.690	0.143	SH3BP1	0.704	0.084	MVK
0.618	0.073	SCD5	0.699	0.079	TMEM212
0.668	0.124	CTSB	0.694	0.074	EN2
0.630	0.089	SERPINA4	0.689	0.069	USP9X
0.675	0.134	CA10	0.706	0.087	TCF20
0.680	0.140	CYFIP1	0.716	0.098	LOC202181
0.657	0.118	CALML4	0.699	0.082	ECE1
0.598	0.061	BRIX1	0.676	0.060	TUFT1
0.615	0.078	MSL3	0.713	0.097	AP2A2
0.630	0.094	TMCO3	0.702	0.087	MS4A1
0.658	0.122	SLC25A24	0.671	0.055	MOGS
0.606	0.071	NT5DC2	0.663	0.048	GTF2B
0.619	0.086	C2ORF56	0.689	0.074	NCAPH2
0.585	0.053	LPPR3	0.708	0.092	SCNN1G
0.626	0.094	YLPM1	0.674	0.060	ZNF646
0.584	0.052	DUSP22	0.635	0.021	HJURP
0.616	0.084	CAD	0.694	0.080	YOD1

0.613	0.083	TJP3	0.710	0.096	PNLIPRP1
0.662	0.132	RSRC1	0.691	0.077	ETV7
0.621	0.091	C1ORF183	0.689	0.076	FBXL8
0.620	0.092	LAMB3	0.690	0.078	LRRC8B
0.605	0.078	FAM60A	0.684	0.071	MARCH5
0.578	0.051	FOXF2	0.687	0.075	TM9SF1
0.582	0.056	APLNR	0.692	0.080	FGF3
0.619	0.094	VAT1	0.678	0.067	SIRT7
0.637	0.114	DDB2	0.666	0.056	SPAG1
0.607	0.084	COL6A2	0.670	0.061	MLXIPL
0.597	0.075	TRAP1	0.681	0.073	ITPR2
0.617	0.096	DKC1	0.690	0.082	PCDHA10
0.614	0.093	KCNG1	0.659	0.053	RBM12B
0.655	0.136	ALS2CL	0.687	0.083	WSCD1
0.593	0.074	EMD	0.699	0.094	TMEM40
0.638	0.120	ANO3	0.681	0.078	EXT2
0.584	0.068	WDR74	0.698	0.095	SYDE1
0.599	0.084	ARL14	0.659	0.061	POLI
0.598	0.084	GRB10	0.674	0.076	EFHC1
0.619	0.105	ARL1	0.698	0.101	OXCT2
0.646	0.131	AHCTF1	0.679	0.083	TAGLN
0.610	0.098	C7ORF42	0.652	0.058	SULT1A2
0.620	0.109	PAAF1	0.687	0.093	ARHGAP22
0.585	0.075	TCEA2	0.674	0.082	DNAJB1
0.580	0.071	ARHGAP15	0.634	0.043	NME1
0.589	0.080	RHOBTB2	0.675	0.087	FAM174B
0.644	0.135	PTGES3	0.670	0.083	COLQ
0.614	0.106	BCL7A	0.614	0.028	CNDP2
0.647	0.139	PIEZO2	0.679	0.094	NRIP2
0.615	0.109	PTPN2	0.646	0.061	EIF3L
0.599	0.094	OPA1	0.625	0.040	TUBA1A
0.587	0.082	PYROXD1	0.650	0.067	ZNF573
0.602	0.097	RSL24D1	0.664	0.084	EPS15L1
0.618	0.113	RPL12	0.669	0.089	MCPH1
0.611	0.107	POLR2D	0.657	0.077	CGA
0.593	0.088	IRAK1	0.642	0.062	TMPRSS2
0.621	0.118	TRIP10	0.674	0.094	TBC1D8
0.600	0.097	ESYT1	0.669	0.090	TTC38
0.624	0.122	RPL22	0.678	0.100	TSPYL5
0.595	0.096	MFF	0.668	0.090	CDK20
0.572	0.075	DMBT1	0.653	0.076	NHP2
0.564	0.068	NUDT3	0.677	0.101	HOXA4
0.588	0.092	HADHB	0.633	0.057	GABARAPL1
0.598	0.103	ABCG2	0.665	0.089	LOC729799
0.612	0.117	DDX54	0.658	0.082	IGF1
0.596	0.103	TMEM97	0.654	0.080	SORL1
0.603	0.110	GNA12	0.659	0.086	TBCB

0.636	0.143	BLNK	0.616	0.043	ORMDL2
0.608	0.117	MREG	0.671	0.098	SYT12
0.577	0.085	LINC00341	0.675	0.103	CEACAM7
0.552	0.061	FHL5	0.655	0.083	ZNF204P
0.565	0.074	PRG4	0.646	0.075	IRF1
0.594	0.104	RPL30	0.677	0.106	KCNMB2
0.588	0.100	PLP1	0.661	0.091	CARD10
0.586	0.098	ICAM1	0.635	0.070	CCDC19
0.643	0.156	CD19	0.615	0.050	KIF14
0.611	0.124	FXYD3	0.660	0.096	ALLC
0.588	0.102	PNMA1	0.637	0.074	FSTL1
0.623	0.138	CAPN3	0.651	0.088	PIGZ
0.551	0.069	NSMAF	0.619	0.057	C21ORF91
0.605	0.123	GSTM4	0.654	0.092	AGAP2
0.586	0.104	ANPEP	0.665	0.104	ZNF33B
0.601	0.120	RSBN1	0.591	0.029	CDC20
0.613	0.132	UNC119	0.645	0.084	SIX5
0.576	0.097	LOC100129361	0.650	0.089	C8G
0.621	0.143	FAM134B	0.606	0.046	HOMER2
0.606	0.128	PTGIR	0.658	0.098	MEIS2
0.578	0.102	RPS24	0.665	0.106	PVRIG
0.587	0.111	RPL23A	0.665	0.105	EFR3B
0.547	0.072	NFX1	0.590	0.032	CDCA8
0.585	0.113	SIM1	0.654	0.098	HEXA
0.578	0.107	NSDHL	0.639	0.083	ACPP
0.607	0.135	CPSF6	0.639	0.083	FOXJ1
0.588	0.118	SLC30A9	0.663	0.108	TCEAL2
0.582	0.113	TNS3	0.646	0.091	BTBD18
0.567	0.099	RPL9	0.599	0.045	PLK1
0.607	0.138	FAM55C	0.637	0.083	BRD4
0.561	0.093	APTX	0.654	0.100	ERV3-2
0.558	0.090	TMEM5	0.615	0.062	NID2
0.586	0.118	PGRMC1	0.635	0.083	FCGR2A
0.560	0.093	ACAT1	0.665	0.112	SIX3
0.607	0.141	EVPL	0.640	0.088	AASS
0.574	0.107	CRADD	0.655	0.103	GPR21
0.562	0.095	PIPOX	0.643	0.091	MFNG
0.596	0.130	DYNC2LI1	0.670	0.118	RNF126
0.554	0.088	POT1	0.646	0.095	PKDREJ
0.567	0.101	CHCHD8	0.641	0.091	HSPA4
0.584	0.118	ARNTL	0.641	0.091	REPIN1
0.563	0.098	CBX7	0.651	0.102	TNFRSF17
0.583	0.119	MEIS1	0.641	0.092	CHST15
0.593	0.129	MTMR4	0.658	0.110	FABP2
0.588	0.124	DLG5	0.663	0.117	MYBPH
0.597	0.134	PTMA	0.623	0.077	GOLPH3L
0.560	0.097	ABCA8	0.664	0.118	RAB3D

0.572	0.109	KIAA0922	0.623	0.078	MMADHC
0.555	0.093	NOL6	0.661	0.117	OR1F1
0.576	0.114	LAMP1	0.650	0.106	CHRNA6
0.526	0.065	PKP1	0.638	0.094	C7ORF58
0.602	0.143	SLC35C2	0.633	0.090	LNPEP
0.544	0.086	ZNF710	0.641	0.098	WDR59
0.593	0.137	CPSF7	0.630	0.089	GADD45G
0.565	0.109	DUSP8	0.637	0.096	CDKN2B
0.564	0.109	MFN2	0.609	0.069	IFI44
0.580	0.126	M6PR	0.660	0.121	OBFC2B
0.537	0.083	HDAC5	0.633	0.094	SRSF7
0.561	0.107	RNF4	0.650	0.111	SRSF4
0.563	0.110	DNAJB12	0.635	0.097	FMOD
0.593	0.141	SNN	0.635	0.097	PLIN2
0.557	0.105	TFCP2	0.632	0.095	SCFD1
0.589	0.138	LSM3	0.632	0.095	SOAT1
0.513	0.062	AOC2	0.645	0.108	PSD
0.566	0.117	MYO1C	0.632	0.096	HLA-DOA
0.533	0.084	HOXC4	0.631	0.095	LINC00115
0.563	0.115	PCTP	0.640	0.103	EFNA1
0.564	0.117	RALGPS1	0.623	0.086	RNF111
0.559	0.112	RGS2	0.638	0.103	HIST3H3
0.548	0.102	NMT1	0.635	0.100	MTCH2
0.556	0.110	RPS9	0.659	0.124	GALR3
0.508	0.063	HOXC11	0.650	0.116	LRRC8E
0.594	0.150	TAC3	0.642	0.109	GYG2
0.545	0.101	FLNB	0.651	0.119	CLOCK
0.559	0.115	RSRC2	0.637	0.106	RORC
0.561	0.118	SLC39A4	0.615	0.085	C20ORF46
0.553	0.111	SUMO3	0.619	0.090	LAD1
0.530	0.088	AKAP3	0.614	0.085	POLR2J2
0.569	0.127	PLEK2	0.643	0.115	LGI1
0.564	0.125	SAR1B	0.637	0.109	ARFRP1
0.573	0.135	ZFAND1	0.612	0.085	VNN2
0.523	0.085	PPP2R5A	0.620	0.093	TK1
0.567	0.129	LINS	0.619	0.092	CPEB3
0.519	0.081	ATG14	0.570	0.043	KCMF1
0.568	0.130	RPLP1	0.626	0.099	SLC7A4
0.548	0.111	RPL23	0.656	0.131	KCNK3
0.524	0.088	ERN2	0.636	0.111	FGF16
0.575	0.139	INPP5F	0.640	0.117	OGFR
0.551	0.115	TRPC4AP	0.643	0.120	RABGGTA
0.527	0.093	RPS3	0.599	0.076	ASCC1
0.572	0.138	CCK	0.630	0.107	PTCD3
0.546	0.113	RAF1	0.632	0.110	EBLN2
0.541	0.108	MYLK	0.633	0.112	TEKT2
0.530	0.098	C22ORF29	0.598	0.077	DYM

0.601	0.170	MYLK3	0.616	0.095	MELK
0.565	0.133	DAG1	0.633	0.112	FGFR4
0.535	0.103	TCL1B	0.622	0.101	DCN
0.551	0.120	FKSG49	0.621	0.101	CDX1
0.521	0.091	JAK1	0.621	0.102	CRP
0.553	0.123	SEL1L3	0.637	0.117	HIST1H1D
0.542	0.113	MORC2	0.625	0.106	MYH7B
0.570	0.141	GRSF1	0.613	0.095	KRT8P12
0.536	0.110	ISL1	0.599	0.080	NVL
0.546	0.120	WDR73	0.621	0.103	SLC37A1
0.553	0.127	ABHD2	0.639	0.121	NDUFS6
0.539	0.114	LILRB3	0.621	0.104	HTR1B
0.548	0.123	ITGA10	0.629	0.112	CLDN11
0.541	0.116	NFYB	0.608	0.092	PPP2R3C
0.560	0.136	VWA5A	0.639	0.124	LSM14A
0.547	0.123	STRN3	0.614	0.099	FLJ13197
0.535	0.111	KLKB1	0.634	0.120	PSMC1
0.544	0.121	ACADL	0.627	0.114	FGF18
0.568	0.146	PLA2G15	0.561	0.048	TCFL5
0.568	0.147	PIGL	0.636	0.124	C22ORF24
0.547	0.126	RPL21	0.633	0.122	GCNT2
0.503	0.083	PLK1	0.618	0.108	GPR31
0.531	0.112	GEMIN4	0.582	0.072	CIB1
0.535	0.116	CYP2B7P1	0.612	0.103	VPS13B
0.510	0.091	RRP1	0.622	0.113	IST1
0.563	0.144	WDR26	0.604	0.095	SERPIND1
0.569	0.152	UBXN6	0.608	0.100	PDE9A
0.536	0.119	CCNF	0.603	0.096	PRSS8
0.555	0.139	C10ORF95	0.626	0.119	PLA2G4A
0.515	0.099	IER3	0.621	0.114	ANXA2P3
0.553	0.137	GABPB1	0.590	0.084	SCGB1D2
0.554	0.139	RPSA	0.621	0.115	USP27X
0.530	0.114	ALDOC	0.646	0.141	MRPL22
0.542	0.129	PGRMC2	0.625	0.120	PDLIM2
0.538	0.126	WFS1	0.622	0.118	BMP3
0.562	0.150	ARPC3	0.609	0.105	SMTN
0.561	0.149	CRYL1	0.543	0.039	ATP5I
0.557	0.145	C12ORF48	0.595	0.091	KLK3
0.507	0.095	SMYD5	0.583	0.083	CRNN
0.550	0.139	ZBED1	0.616	0.116	DFFA
0.536	0.126	LIAS	0.619	0.119	TAPBPL
0.520	0.110	BCORL1	0.605	0.106	PPIL2
0.558	0.148	DCLRE1C	0.588	0.090	EAF2
0.539	0.129	SLC22A18	0.613	0.115	TUB
0.574	0.164	HNRNPH2	0.608	0.111	TTLL5
0.504	0.095	CEP192	0.588	0.092	ASL
0.546	0.137	RPS12	0.607	0.112	GAMT

0.529	0.121	C7ORF49	0.598	0.103	GOLPH3
0.521	0.113	KBTBD4	0.608	0.114	DZIP1
0.532	0.126	FAM193A	0.608	0.115	TNFSF4
0.549	0.144	ZNF142	0.617	0.123	KLF15
0.591	0.186	CBY1	0.607	0.114	ADAM11
0.553	0.148	FERMT1	0.604	0.111	BICD1
0.538	0.133	EFNA3	0.594	0.102	MBIP
0.518	0.113	RBM15B	0.616	0.124	ZNF221
0.537	0.132	MAN2B2	0.605	0.114	LOC647070
0.539	0.135	SERHL2	0.640	0.149	CLPS
0.505	0.100	SLC22A4	0.526	0.035	CDC25B
0.541	0.138	HERC2	0.629	0.138	CD3G
0.533	0.129	KIAA0100	0.587	0.098	LPHN3
0.534	0.131	DTWD1	0.602	0.113	SPTSSA
0.544	0.142	RNF220	0.596	0.108	ITIH1
0.536	0.133	ACTA1	0.569	0.082	PEX13
0.531	0.129	PMP22	0.621	0.134	TMBIM4
0.547	0.145	DSN1	0.603	0.117	ZNF180
0.566	0.164	DDX1	0.558	0.072	DERL2
0.560	0.159	MOSPD2	0.577	0.091	EFCAB2
0.530	0.130	FXN	0.605	0.120	KHSRP
0.516	0.116	CTSE	0.599	0.113	HESX1
0.544	0.145	SSB	0.612	0.127	IQGAP2
0.507	0.108	CGRRF1	0.614	0.130	MRPL42
0.527	0.128	SCG5	0.594	0.111	TFAP2B
0.553	0.154	HOXD11	0.609	0.126	SCAP
0.527	0.129	SIX2	0.610	0.127	CHRM4
0.509	0.112	CD96	0.606	0.123	SCN3B
0.549	0.154	AASDHPPT	0.601	0.119	CREB3L1
0.572	0.178	GNL1	0.610	0.128	STAG2
0.514	0.121	SPEG	0.575	0.094	FAM178A
0.531	0.137	CTSL1	0.613	0.133	FRMPD1
0.513	0.121	PTMS	0.613	0.133	NDUFAB1
0.519	0.127	RETSAT	0.562	0.083	TMEM121
0.539	0.147	NFYA	0.597	0.121	UBA7
0.539	0.149	SF3A3	0.569	0.094	BBC3
0.539	0.149	CDK5RAP2	0.590	0.115	GAS1
0.506	0.117	KLHL9	0.539	0.064	UGDH
0.577	0.188	HRASLS	0.599	0.125	ACAA2
0.558	0.170	PPP1R9A	0.602	0.128	BSN
0.576	0.189	LOC100127972	0.614	0.140	PEX2
0.552	0.165	MPZ	0.607	0.133	MTSS1
0.523	0.138	AQP3	0.608	0.135	ZBED4
0.560	0.175	DEFB1	0.584	0.111	CXXC4
0.534	0.150	SAFB2	0.592	0.119	DTX3
0.523	0.142	RPL4	0.578	0.106	TMEM66
0.506	0.125	TMEM33	0.602	0.131	LOC100508797

0.561	0.180	C2ORF44	0.566	0.094	APRT
0.510	0.129	H2BFS	0.592	0.120	MAGI1
0.519	0.140	EEF1G	0.604	0.133	C9ORF167
0.519	0.140	C4BPB	0.593	0.121	YES1
0.527	0.149	EEF1A1	0.580	0.109	SPHK1
0.501	0.122	CETP	0.539	0.068	NRIP1
0.504	0.126	RABL3	0.579	0.108	VPS35
0.523	0.145	DLG1	0.578	0.107	GBP1
0.545	0.167	GJB1	0.608	0.137	FBN1
0.545	0.168	STAT5B	0.614	0.145	IL11
0.561	0.187	E4F1	0.599	0.131	TPO
0.515	0.141	CRTAP	0.573	0.105	KIAA0196
0.529	0.155	PSMC3IP	0.587	0.120	PROSC
0.517	0.147	UBE2D2	0.587	0.120	MKRN2
0.503	0.134	SLC33A1	0.539	0.075	CRELD2
0.538	0.169	TFF2	0.574	0.110	FANCI
0.565	0.196	RHBG	0.540	0.076	CTDSP1
0.513	0.145	MOB3B	0.594	0.131	CWF19L1
0.503	0.136	GSN	0.579	0.116	PHTF1
0.509	0.143	WDR61	0.569	0.106	HIST1H2AM
0.527	0.162	RPL34	0.578	0.116	DYSF
0.518	0.153	RPS2	0.564	0.103	PITX3
0.502	0.137	ZNF358	0.571	0.111	LSM14B
0.504	0.139	FAIM2	0.599	0.139	TM4SF5
0.521	0.156	FUCA1	0.556	0.097	HELZ
0.505	0.143	PPIP5K1	0.541	0.082	PLP2
0.529	0.167	RASSF4	0.561	0.103	LHFPL2
0.514	0.153	PSMD1	0.583	0.125	SMARCA1
0.522	0.161	CAND1	0.572	0.114	CLDN8
0.522	0.162	TBL1XR1	0.608	0.151	HMGB2
0.530	0.171	PHF16	0.567	0.110	TM9SF3
0.505	0.148	DKK4	0.537	0.080	TCF15
0.501	0.146	CHERP	0.552	0.095	PSMA6
0.542	0.188	SMEK2	0.580	0.124	HIST1H4B
0.529	0.176	DCUN1D2	0.586	0.130	ZNF93
0.524	0.171	AQP5	0.568	0.113	AMPD3
0.531	0.180	ERC2	0.579	0.124	FOXD2
0.503	0.152	ZKSCAN5	0.601	0.146	LRRC68
0.531	0.181	CD7	0.570	0.116	NKX3-1
0.506	0.156	SNUPN	0.591	0.137	LIFR
0.501	0.153	RPLP0	0.575	0.121	TNFRSF13B
0.514	0.169	GPBP1L1	0.566	0.114	RNF138
0.505	0.161	UQCC	0.571	0.120	HEATR8
0.501	0.158	TGIF1	0.572	0.121	HDAC6
0.507	0.165	HSP90AB1	0.557	0.107	FUT7
0.514	0.174	RUNX3	0.564	0.114	LENEP
0.516	0.177	DIO1	0.558	0.108	ALDH1A3

0.507	0.169	MRAS
0.505	0.167	RPS4X
0.503	0.169	LOC100507009
0.513	0.180	EPB49
0.510	0.177	CPNE7
0.538	0.208	PHLDA3
0.516	0.189	HR
0.505	0.187	DNASE1L2
0.508	0.191	PKD1P1
0.520	0.204	CRYAB
0.505	0.198	MARCH8
0.504	0.237	GATA4

0.548	0.099	RANBP6
0.572	0.124	EDN2
0.581	0.133	RGS3
0.551	0.103	RPL13P5
0.580	0.132	PCDHGA1
0.570	0.123	SLC4A2
0.580	0.134	RGS11
0.541	0.096	PTTG3P
0.575	0.130	KDM6B
0.554	0.108	IFIT1
0.538	0.093	RRAGA
0.579	0.134	SLC28A2
0.571	0.128	CEACAM1
0.580	0.137	SAA3P
0.570	0.128	ADAM20
0.555	0.113	YIF1B
0.591	0.150	ATF2
0.573	0.132	SPTBN5
0.513	0.073	RND1
0.587	0.148	MUC3A
0.572	0.134	ZNF226
0.565	0.128	TRMT12
0.558	0.122	CPNE3
0.578	0.143	ZNF277
0.547	0.113	ERG
0.565	0.130	C7ORF54
0.543	0.109	NKTR
0.573	0.138	MINPP1
0.571	0.137	FKSG2
0.566	0.132	GJB4
0.567	0.134	PRLH
0.576	0.143	TROVE2
0.530	0.097	SCAMP3
0.554	0.122	TTC23
0.584	0.152	CELA3B
0.547	0.116	THRAP3
0.553	0.122	ALPI
0.552	0.121	CELP
0.518	0.088	LCN2
0.530	0.100	ZNF268
0.563	0.133	CASP6
0.573	0.143	GNG13
0.555	0.125	DENND4A
0.556	0.127	STAP1
0.589	0.160	MRPS15
0.544	0.117	PAF1
0.552	0.125	NELL2

	0.547	0.120	FZD5
	0.561	0.136	INPP4A
	0.560	0.134	C22ORF29
	0.557	0.132	IL17RA
	0.559	0.134	IQCA1
	0.556	0.132	MMP11
	0.562	0.139	APOL3
	0.530	0.107	SPTBN4
	0.575	0.152	C1ORF106
	0.547	0.125	IL1RAPL1
	0.558	0.136	CEACAM4
	0.542	0.120	MBOAT2
	0.523	0.102	EIF3D
	0.548	0.128	TSPAN1
	0.556	0.135	HOXB1
	0.549	0.128	SLC25A16
	0.563	0.145	CGGBP1
	0.565	0.147	SCAPER
	0.508	0.090	SDCBP
	0.563	0.147	AHSG
	0.552	0.137	CLSTN3
	0.535	0.120	ARHGAP33
	0.544	0.130	SEMA3F
	0.545	0.131	LAP3
	0.537	0.123	GRRP1
	0.549	0.135	UBXN1
	0.563	0.150	ATP9B
	0.534	0.121	BTN3A3
	0.556	0.143	CETN1
	0.555	0.144	GCH1
	0.574	0.163	SNRPB2
	0.542	0.131	GAS2L1
	0.552	0.142	PRKAR1A
	0.554	0.145	FAM153A
	0.549	0.141	C1ORF174
	0.544	0.135	ATP10B
	0.517	0.109	CXCL13
	0.547	0.140	NDUFB8
	0.546	0.139	CLDN15
	0.547	0.140	SPPL2B
	0.559	0.153	ACOX2
	0.561	0.154	NOD1
	0.546	0.141	KLRAP1
	0.554	0.148	MASP2
	0.557	0.152	PBXIP1
	0.542	0.138	AFF4
	0.518	0.114	CERK

	0.538	0.135	PIAS3
	0.553	0.150	PLXNA2
	0.535	0.132	FAM171A1
	0.549	0.146	CISH
	0.538	0.135	TOP3B
	0.527	0.125	SEPT8
	0.525	0.123	GIMAP6
	0.514	0.114	CSNK2A2
	0.556	0.156	ADAM7
	0.539	0.139	LGR5
	0.537	0.138	CLDN9
	0.534	0.134	IFITM1
	0.562	0.162	H2AFZ
	0.509	0.109	USP47
	0.549	0.150	OCM2
	0.536	0.138	SYNJ2
	0.509	0.111	PCP4
	0.555	0.157	PLAC8
	0.546	0.150	SGSH
	0.548	0.152	PFDN1
	0.554	0.158	LSM2
	0.523	0.128	C3ORF64
	0.526	0.131	NFKB2
	0.526	0.132	ADPGK
	0.523	0.129	HIST1H1T
	0.543	0.150	SEC24D
	0.531	0.138	CTIF
	0.531	0.138	SCNN1A
	0.504	0.111	CKS2
	0.535	0.143	CDK18
	0.544	0.152	NEUROG3
	0.542	0.152	FAM198B
	0.513	0.124	TRIM36
	0.515	0.126	FAM168B
	0.537	0.148	NT5C
	0.551	0.164	OBSL1
	0.522	0.135	F10
	0.525	0.138	ATAD5
	0.541	0.156	METTL10
	0.530	0.146	PAK7
	0.526	0.142	LANCL1
	0.538	0.156	GPR161
	0.521	0.139	RPRD1A
	0.532	0.150	MLPH
	0.519	0.137	MAPK8
	0.529	0.148	MAPK4
	0.534	0.153	FAM125B

0.536	0.157	SF3B5
0.544	0.166	ECM1
0.522	0.144	PKN2
0.528	0.150	HRH3
0.546	0.168	HOXA3
0.530	0.152	ADH1C
0.510	0.133	PCNX
0.527	0.150	DBF4B
0.519	0.142	IGH@
0.517	0.141	GHRHR
0.510	0.133	POLR2K
0.540	0.164	GALNT8
0.534	0.158	ACE
0.523	0.148	ABL2
0.530	0.156	POMC
0.534	0.159	RBM41
0.524	0.150	ZNF282
0.530	0.156	FZD6
0.505	0.132	CCDC70
0.535	0.162	MEP1A
0.535	0.162	SULF1
0.512	0.139	TAF4B
0.523	0.151	PTPRN2
0.516	0.144	ATOH1
0.541	0.169	HOXB5
0.525	0.154	TTLL3
0.524	0.154	CLDN3
0.528	0.159	RRAS2
0.523	0.154	KIR2DL4
0.503	0.138	RRP8
0.524	0.160	ARHGEF4
0.524	0.160	ZNF606
0.521	0.159	RIC3
0.515	0.153	PAX5
0.507	0.145	S100PBP
0.529	0.168	SPAG9
0.507	0.146	PPP1R13B
0.518	0.157	GPN2
0.506	0.145	CCDC134
0.522	0.162	PLEKHM1
0.521	0.161	TANK
0.541	0.181	LIMCH1
0.509	0.150	UBE2A
0.519	0.160	RASGRP2
0.517	0.158	SSX5
0.507	0.148	GPR97
0.511	0.153	EPM2A

	0.504	0.147	SRRM1
	0.525	0.168	FLT1
	0.503	0.146	NOTCH2
	0.514	0.157	NEU3
	0.514	0.157	CEP112
	0.533	0.176	HMGN3
	0.516	0.161	AMELY
	0.515	0.160	CA5B
	0.518	0.165	SLC22A6
	0.529	0.176	MTRR
	0.503	0.149	CNR1
	0.512	0.160	CWH43
	0.513	0.163	BUB1
	0.507	0.157	RIMBP2
	0.505	0.156	GNRH2
	0.513	0.164	KIAA0894
	0.504	0.157	CCDC71
	0.515	0.169	UBR2
	0.504	0.158	ZNF287
	0.519	0.174	ACTL7A
	0.504	0.160	LHB
	0.509	0.165	ADAMTS12
	0.509	0.165	ZER1
	0.513	0.171	OFD1
	0.514	0.174	SYN2
	0.516	0.179	MCMBP
	0.504	0.167	TMEM147
	0.501	0.165	FGF23
	0.508	0.172	SLC26A2
	0.514	0.180	UBN1
	0.509	0.176	C8ORF60
	0.507	0.175	PHF20
	0.501	0.171	SH3BGRL3
	0.502	0.172	EIF2C2
	0.506	0.176	ACSM1
	0.503	0.174	HPCAL1
	0.507	0.178	CLINT1
	0.502	0.174	CCDC40
	0.504	0.176	IFT27
	0.506	0.183	SLC6A7
	0.506	0.182	GIPR
	0.501	0.184	ELAVL4

Combination prediction result

Name	Overlap count	Up	Down	Result
PTPN2	6	3	3	
AURKA	5	2	3	
CCNO	5	3	2	
CEP57	5	2	3	
EAF2	5	2	3	
KIAA0753	5	2	3	
OPA1	5	2	3	
PLEC	5	1	4	
RBM23	5	3	2	
SPHK1	5	2	3	
SREK1	5	2	3	
TMPRSS2	5	4	1	
TNFRSF1A	5	3	2	
TUBA4A	5	3	2	
ZNF277	5	3	2	
ZNF32	5	2	3	
ZNF45	5	2	3	
ADM	4	3	1	
AKAP8	4	1	3	
ALKBH1	4	2	2	
ANKRD12	4	3	1	
APPL1	4	1	3	
ARMC8	4	2	2	
ARMCX5	4	1	3	
ARNT2	4	1	3	
ASPH	4	2	2	
ATG5	4	2	2	
BCL7C	4	2	2	
BCLAF1	4	2	2	
BNIP1	4	1	3	
CBR3	4	3	1	
CCL2	4	2	2	
CD22	4	2	2	
CDCA4	4	2	2	
CDT1	4	3	1	
CHST15	4	1	3	
COQ9	4	2	2	
CTSB	4	2	2	
DCAF17	4	2	2	
DERL2	4	2	2	
DHX40	4	2	2	
DNAJB12	4	2	2	
DNAJC10	4	1	3	
DOCK4	4	1	3	
EEF1B2	4	2	2	

EFNA3	4	1	3	
EHD1	4	3	1	
EIF2B2	4	3	1	
EIF5B	4	1	3	
FDXR	4	3	1	
FEM1B	4	1	3	
FKBP5	4	3	1	
FOXC1	4	2	2	
FTL	4	1	3	
FUBP1	4	1	3	
GAA	4	3	1	
GMEB2	4	2	2	
GRB10	4	1	3	
GRN	4	1	3	
GRSF1	4	1	3	
HERC1	4	2	2	
HIBCH	4	1	3	
HIST1H2BE	4	3	1	
HMGCR	4	2	2	
HSPA4	4	4	0	
ID1	4	4	0	
IER3	4	2	2	
IFIT1	4	3	1	
IGF2R	4	3	1	
ISL1	4	2	2	
ITGA6	4	1	3	
IVNS1ABP	4	1	3	
KCNIP1	4	2	2	
KIAA0664L3	4	2	2	
KIAA1324	4	2	2	
KLHL9	4	2	2	
KRAS	4	2	2	
KRT10	4	3	1	
LAD1	4	2	2	
MAP3K9	4	2	2	
MAST4	4	0	4	
MRPL16	4	1	3	
MRPS31	4	2	2	
MTMR4	4	1	3	
MYBL1	4	2	2	
NAP1L2	4	2	2	
NFKBIL1	4	1	3	
NFX1	4	2	2	
NME1	4	2	2	
NR3C1	4	2	2	
NUBP1	4	1	3	
NVL	4	3	1	
OSBPL1A	4	1	3	

PAAF1	4	1	3	
PARP2	4	0	4	
PCP4	4	2	2	
PDS5B	4	3	1	
PHB	4	1	3	
PODXL	4	2	2	
POLG2	4	1	3	
PPCDC	4	2	2	
PPME1	4	3	1	
PTCD3	4	2	2	
PTPN9	4	1	3	
PTRH2	4	1	3	
PWP1	4	2	2	
PYROXD1	4	1	3	
RAD9A	4	2	2	
RCOR1	4	3	1	
SAMM50	4	2	2	
SEC61A2	4	2	2	
SETD1B	4	1	3	
SFXN3	4	2	2	
SIDT1	4	2	2	
SLC12A2	4	2	2	
SLC25A37	4	1	3	
SLC35E3	4	1	3	
SLC39A4	4	2	2	
SPRED2	4	3	1	
SRSF5	4	2	2	
STEAP1	4	2	2	
STK39	4	2	2	
STRN3	4	2	2	
SUV420H1	4	1	3	
SYK	4	2	2	
TACC1	4	2	2	
TBC1D1	4	2	2	
TBCC	4	2	2	
TBL1XR1	4	1	3	
THBD	4	1	3	
TLK1	4	1	3	
TRIM33	4	3	1	
TRIM36	4	3	1	
TTI1	4	1	3	
TXNL4B	4	2	2	
UBE2C	4	2	2	
UBE2D3	4	2	2	
UCP2	4	2	2	
UFL1	4	2	2	
UNKL	4	2	2	
UQCRFS1	4	2	2	

VDR	4	3	1	
ZFAND1	4	2	2	
ZNF148	4	2	2	
ZNF23	4	2	2	
ZNF239	4	2	2	
ZNF337	4	1	3	
ZNF7	4	1	3	
ZNF783	4	3	1	
ZNF93	4	2	2	
AASDHPPT	3	1	2	
ABCA11P	3	2	1	
ABCC5	3	0	3	
ABCF3	3	1	2	
ABHD3	3	3	0	
ACAT1	3	1	2	
ACD	3	0	3	
ACOT9	3	1	2	
ACSL3	3	1	2	
ADARB1	3	1	2	
ADCK3	3	1	2	
ADH1C	3	1	2	
ADNP2	3	2	1	
ADO	3	1	2	
AFF1	3	1	2	
AFF4	3	2	1	
AGPAT3	3	2	1	
AGXT2L1	3	1	2	
AHSA1	3	1	2	
AIMP1	3	0	3	
AKAP17A	3	2	1	
AKIP1	3	1	2	
ALAD	3	1	2	
ALAS1	3	1	2	
ALG13	3	1	2	
ALMS1	3	0	3	
ALS2CL	3	1	2	
ANKH	3	1	2	
ANO1	3	1	2	
APOBEC3B	3	2	1	
APOE	3	3	0	
APPBP2	3	2	1	
APTX	3	0	3	
ARCN1	3	2	1	
ARFGAP2	3	3	0	
ARFIP2	3	2	1	
ARHGAP17	3	1	2	
ARHGEF26	3	1	2	
ARHGEF7	3	3	0	

ARID4A	3	2	1	
ARID5B	3	2	1	
ARL1	3	0	3	
ARMC6	3	1	2	
ASCC1	3	1	2	
ASCC3	3	2	1	
ASNA1	3	2	1	
ATF2	3	2	1	
ATG4A	3	2	1	
ATIC	3	0	3	
ATMIN	3	0	3	
ATP5C1	3	2	1	
ATP5O	3	1	2	
B4GALT1	3	2	1	
BAIAP2	3	2	1	
BANK1	3	2	1	
BBS7	3	1	2	
BCL2	3	2	1	
BICD1	3	2	1	
BLM	3	1	2	
BMI1	3	1	2	
BMPR1A	3	1	2	
BPGM	3	0	3	
BPIFA1	3	1	2	
BRE	3	1	2	
BRF2	3	1	2	
BRMS1	3	2	1	
BTG1	3	2	1	
C14ORF133	3	1	2	
C19ORF24	3	2	1	
C20ORF103	3	1	2	
C21ORF91	3	2	1	
C22ORF29	3	2	1	
C2ORF47	3	2	1	
C3	3	2	1	
C4ORF29	3	1	2	
C8ORF4	3	2	1	
CALM3	3	2	1	
CAMKK2	3	2	1	
CASP7	3	2	1	
CAT	3	2	1	
CBR4	3	0	3	
CCDC15	3	1	2	
CCHCR1	3	1	2	
CCNA2	3	2	1	
CCNE2	3	2	1	
CCNT1	3	2	1	
CCT6A	3	1	2	

CD80	3	1	2	
CDC16	3	0	3	
CDC42EP2	3	1	2	
CDC42EP4	3	1	2	
CDCP1	3	1	2	
CDK17	3	2	1	
CDR2L	3	1	2	
CEACAM6	3	1	2	
CEBPD	3	2	1	
CENPF	3	3	0	
CEP290	3	2	1	
CEP70	3	1	2	
CEP76	3	1	2	
CEPT1	3	1	2	
CETP	3	1	2	
CFLAR	3	2	1	
CGA	3	2	1	
CGGBP1	3	2	1	
CHAF1A	3	2	1	
CHD9	3	2	1	
CHERP	3	2	1	
CHKB	3	2	1	
CISH	3	2	1	
CITED2	3	2	1	
CLCN3	3	1	2	
CLMN	3	1	2	
CMAS	3	1	2	
CNOT6	3	2	1	
COCH	3	2	1	
COL17A1	3	1	2	
COL4A3BP	3	1	2	
COL5A1	3	2	1	
COPS6	3	2	1	
COPS7B	3	1	2	
CPSF7	3	2	1	
CPT1A	3	1	2	
CRBN	3	1	2	
CRCP	3	2	1	
CREBL2	3	1	2	
CRELD2	3	3	0	
CRKL	3	3	0	
CRNKL1	3	1	2	
CSDE1	3	2	1	
CSNK2A2	3	2	1	
CSRP2	3	2	1	
CTBP1	3	1	2	
CTGF	3	1	2	
CTSK	3	2	1	

CTSL2	3	1	2	
CXCL10	3	3	0	
CYB5R1	3	1	2	
CYP11B2	3	2	1	
CYP26A1	3	2	1	
CYP26B1	3	1	2	
CYP27B1	3	2	1	
CYR61	3	2	1	
DAAM1	3	3	0	
DARS	3	1	2	
DCK	3	1	2	
DCUN1D2	3	1	2	
DDX17	3	2	1	
DDX31	3	1	2	
DDX52	3	2	1	
DEGS1	3	3	0	
DEK	3	1	2	
DENND2D	3	1	2	
DENND4A	3	2	1	
DENND4B	3	1	2	
DENND4C	3	1	2	
DERA	3	2	1	
DIAPH2	3	1	2	
DIO2	3	1	2	
DIP2C	3	1	2	
DIXDC1	3	1	2	
DLAT	3	1	2	
DLD	3	2	1	
DLG1	3	2	1	
DNAJA3	3	1	2	
DNAJB9	3	1	2	
DSE	3	1	2	
DUSP12	3	1	2	
DUSP3	3	1	2	
DYRK2	3	1	2	
DZIP3	3	2	1	
EBP	3	1	2	
ECHDC3	3	2	1	
EFHD2	3	2	1	
EFNB2	3	1	2	
EGR3	3	2	1	
EIF2B3	3	0	3	
EIF2C3	3	2	1	
EIF3A	3	1	2	
EIF4ENIF1	3	1	2	
ELF2	3	3	0	
ELF3	3	1	2	
ELF5	3	2	1	

ELK4	3	2	1	
ELOVL4	3	2	1	
ENGASE	3	2	1	
ENOX2	3	0	3	
ENTPD6	3	2	1	
EP300	3	2	1	
ERCC2	3	0	3	
ERF	3	2	1	
ERI2	3	1	2	
ERN2	3	2	1	
ETAA1	3	1	2	
EXOG	3	2	1	
EXOSC4	3	2	1	
EXOSC8	3	0	3	
F11R	3	2	1	
F8	3	1	2	
F9	3	1	2	
FAAH	3	2	1	
FADD	3	1	2	
FAM117A	3	1	2	
FAM179B	3	1	2	
FAM46C	3	2	1	
FAM57A	3	1	2	
FAM86C1	3	1	2	
FANCI	3	1	2	
FASTKD1	3	0	3	
FASTKD2	3	2	1	
FBXO38	3	2	1	
FGFR2	3	2	1	
FHOD1	3	1	2	
FJX1	3	1	2	
FLJ10038	3	1	2	
FMNL1	3	2	1	
FN3KRP	3	2	1	
FOXM1	3	2	1	
FRAT2	3	2	1	
FRYL	3	2	1	
FSTL3	3	3	0	
FUT3	3	1	2	
GABARAPL1	3	3	0	
GABPA	3	2	1	
GADD45G	3	1	2	
GALE	3	1	2	
GALNT10	3	1	2	
GALNT11	3	2	1	
GALNT6	3	0	3	
GART	3	1	2	
GATA2	3	2	1	

GGH	3	3	0	
GLOD4	3	1	2	
GLRX2	3	0	3	
GLRX5	3	1	2	
GLUD2	3	1	2	
GNA11	3	1	2	
GNB2L1	3	1	2	
GNMT	3	2	1	
GORASP2	3	0	3	
GOSR1	3	1	2	
GPD1	3	1	2	
GPR19	3	1	2	
GREB1	3	2	1	
H2AFY2	3	2	1	
H2BFS	3	1	2	
HDGF	3	2	1	
HELZ	3	2	1	
HERC2	3	1	2	
HINFP	3	2	1	
HIST1H2BF	3	2	1	
HIST1H2BH	3	3	0	
HIST1H4C	3	2	1	
HIST3H2A	3	2	1	
HIVEP1	3	2	1	
HJURP	3	2	1	
HNRNPA3	3	0	3	
HOXA10	3	2	1	
HOXC13	3	1	2	
ICT1	3	2	1	
ID3	3	3	0	
IFT88	3	1	2	
IL11	3	2	1	
IL1R1	3	2	1	
IL24	3	1	2	
IL6ST	3	2	1	
INPP1	3	2	1	
INS	3	1	2	
INTS3	3	1	2	
IPPK	3	1	2	
IRGQ	3	2	1	
IRX4	3	1	2	
IRX5	3	0	3	
IST1	3	3	0	
IVL	3	1	2	
JMJD6	3	2	1	
JUNB	3	1	2	
KAZALD1	3	1	2	
KCTD3	3	1	2	

KDELR3	3	1	2	
KIAA0430	3	1	2	
KIAA0754	3	1	2	
KIAA0894	3	2	1	
KIAA1024	3	1	2	
KIAA1109	3	2	1	
KIF15	3	1	2	
KIF16B	3	1	2	
KIF18A	3	2	1	
KIF1B	3	1	2	
KIF20A	3	3	0	
KLF10	3	1	2	
KLF11	3	1	2	
KLHDC2	3	1	2	
KLK2	3	3	0	
KPNA5	3	2	1	
KRIT1	3	1	2	
KRT3	3	1	2	
KRT6A	3	3	0	
KRT8P12	3	1	2	
LCMT2	3	2	1	
LEMD3	3	1	2	
LHFPL2	3	1	2	
LIAS	3	1	2	
LIMA1	3	2	1	
LIN7C	3	2	1	
LRRC16A	3	1	2	
LRRC8D	3	1	2	
LRRC8E	3	2	1	
LSM14A	3	2	1	
LTBP1	3	1	2	
LYST	3	3	0	
MAFB	3	1	2	
MAGEF1	3	0	3	
MALT1	3	1	2	
MAP2K5	3	1	2	
MAP3K5	3	0	3	
MAP3K7	3	1	2	
MAPT	3	2	1	
MARCH6	3	2	1	
MARCH8	3	1	2	
MAX	3	1	2	
MBNL1	3	1	2	
MCL1	3	1	2	
MCMBP	3	1	2	
MDM2	3	1	2	
MED13	3	2	1	
MED13L	3	1	2	

MED15	3	1	2	
MEG3	3	2	1	
MEIS1	3	2	1	
MEPCE	3	0	3	
METAP2	3	0	3	
METTL9	3	2	1	
MFAP1	3	2	1	
MGLL	3	1	2	
MINPP1	3	2	1	
MLF1	3	0	3	
MLL	3	2	1	
MLPH	3	1	2	
MMD	3	2	1	
MMP19	3	2	1	
MNX1	3	1	2	
MORF4L2	3	2	1	
MPHOSPH10	3	1	2	
MRPL15	3	0	3	
MRPS27	3	1	2	
MRTO4	3	1	2	
MSL1	3	1	2	
MSL2	3	2	1	
MT1H	3	2	1	
MTFR1	3	2	1	
MTHFSD	3	1	2	
MTIF2	3	1	2	
MTMR11	3	1	2	
MTMR14	3	2	1	
MTMR6	3	1	2	
MTSS1	3	2	1	
MTUS1	3	1	2	
MTX2	3	1	2	
MUM1	3	1	2	
MXD1	3	2	1	
MYB	3	2	1	
MYRIP	3	2	1	
N4BP2L2	3	1	2	
NAPA	3	2	1	
NCAPG	3	2	1	
NCKAP1	3	1	2	
NDE1	3	2	1	
NEDD4	3	0	3	
NEDD9	3	2	1	
NEK1	3	1	2	
NEMF	3	1	2	
NFE2L1	3	2	1	
NFYA	3	1	2	
NIF3L1	3	1	2	

NOP56	3	1	2	
NOTCH2	3	2	1	
NR1D2	3	2	1	
NR2C2	3	2	1	
NRIP1	3	2	1	
NSDHL	3	2	1	
NSMAF	3	1	2	
NSMCE4A	3	1	2	
NTHL1	3	1	2	
NUP107	3	0	3	
NUP98	3	0	3	
NXT1	3	1	2	
OAZ2	3	1	2	
ODAM	3	1	2	
OGFR	3	1	2	
OGT	3	2	1	
OR7E14P	3	2	1	
OSBPL11	3	1	2	
OXA1L	3	1	2	
OXSM	3	1	2	
P2RX5	3	1	2	
PABPN1	3	2	1	
PAK1IP1	3	0	3	
PAN2	3	1	2	
PANX1	3	0	3	
PAPOLA	3	1	2	
PARP4	3	0	3	
PATZ1	3	2	1	
PCBP2	3	1	2	
PCIF1	3	2	1	
PCMTD2	3	0	3	
PCTP	3	2	1	
PDHA1	3	1	2	
PDK4	3	1	2	
PDLIM5	3	3	0	
PDZD2	3	3	0	
PER2	3	1	2	
PER3	3	1	2	
PEX13	3	2	1	
PHF10	3	1	2	
PID1	3	2	1	
PIEZO2	3	1	2	
PIK3R3	3	3	0	
PITPNB	3	2	1	
PLAUR	3	1	2	
PLEK2	3	2	1	
PLK1	3	1	2	
PLK4	3	3	0	

PLP2	3	2	1	
PNP	3	1	2	
POF1B	3	1	2	
POGZ	3	2	1	
POLD2	3	1	2	
POLI	3	2	1	
POLR3G	3	1	2	
POT1	3	0	3	
POU2F1	3	2	1	
PPARD	3	2	1	
PPL	3	2	1	
PPM1B	3	2	1	
PPP1R12A	3	2	1	
PPP2R5C	3	1	2	
PPRC1	3	1	2	
PPWD1	3	1	2	
PRDM10	3	2	1	
PRDM4	3	1	2	
PRIM1	3	2	1	
PRMT1	3	2	1	
PRPF4B	3	1	2	
PRR7	3	1	2	
PSMB9	3	2	1	
PSMD7	3	1	2	
PSME4	3	1	2	
PTBP2	3	2	1	
PTCRA	3	1	2	
PTMA	3	2	1	
PTMS	3	2	1	
PTPN12	3	2	1	
PTX3	3	1	2	
PUM2	3	1	2	
PURA	3	1	2	
PUS7	3	2	1	
PWP2	3	0	3	
RAB14	3	2	1	
RAB2A	3	1	2	
RAB32	3	2	1	
RAB40B	3	1	2	
RAB4A	3	2	1	
RAB5B	3	1	2	
RANBP2	3	2	1	
RANGAP1	3	1	2	
RAPGEF2	3	2	1	
RARG	3	3	0	
RBM15B	3	0	3	
RBM38	3	1	2	
RCHY1	3	2	1	

RECQL	3	2	1	
RECQL5	3	2	1	
REEP1	3	2	1	
REEP5	3	1	2	
REG1B	3	1	2	
RETSAT	3	1	2	
RGS10	3	2	1	
RGS3	3	2	1	
RHBG	3	1	2	
RIBC2	3	2	1	
RIOK2	3	1	2	
RIPK4	3	2	1	
RIT1	3	2	1	
RNF111	3	2	1	
RNF113A	3	1	2	
RNF6	3	1	2	
RPL11	3	1	2	
RPL4	3	1	2	
RPP14	3	2	1	
RPRD2	3	2	1	
RRAS2	3	2	1	
RRP1	3	0	3	
RRP7A	3	1	2	
RSAD2	3	2	1	
RSBN1	3	1	2	
RTF1	3	1	2	
RTN4	3	2	1	
RWDD3	3	0	3	
RYBP	3	2	1	
SACS	3	1	2	
SAFB2	3	2	1	
SCAMP1	3	0	3	
SCD5	3	1	2	
SCFD1	3	2	1	
SCMH1	3	1	2	
SCNN1A	3	2	1	
SDHAF1	3	2	1	
SEC24D	3	2	1	
SESN1	3	0	3	
SETD2	3	2	1	
SGPP1	3	2	1	
SH3D21	3	1	2	
SIAH1	3	2	1	
SLC16A1	3	1	2	
SLC25A13	3	0	3	
SLC26A2	3	2	1	
SLC33A1	3	0	3	
SLC37A1	3	2	1	

SLC41A3	3	1	2	
SLC4A7	3	2	1	
SLCO4A1	3	2	1	
SLMO2	3	1	2	
SMAD7	3	2	1	
SMCHD1	3	1	2	
SNAP23	3	2	1	
SNORA21	3	1	2	
SNRPB2	3	2	1	
SNX10	3	2	1	
SOCS5	3	2	1	
SOX4	3	1	2	
SPAG9	3	3	0	
SPATA2L	3	1	2	
SPATA6	3	1	2	
SPR	3	2	1	
SRSF4	3	2	1	
SSH1	3	1	2	
STAG1	3	2	1	
STAM2	3	2	1	
STAT3	3	2	1	
STAT5A	3	3	0	
STAT5B	3	0	3	
STEAP3	3	1	2	
STK17A	3	3	0	
STOML2	3	2	1	
STX3	3	2	1	
SUB1	3	1	2	
SYBU	3	0	3	
SYNJ2	3	2	1	
TAF4B	3	2	1	
TAF5	3	1	2	
TAOK2	3	1	2	
TBC1D5	3	2	1	
TBKBP1	3	1	2	
TBPL1	3	3	0	
TBX2	3	2	1	
TCF25	3	2	1	
TCF3	3	2	1	
TCIRG1	3	1	2	
TEAD1	3	1	2	
TFAP2C	3	2	1	
TGDS	3	0	3	
TGFB2	3	2	1	
THAP1	3	2	1	
THNSL1	3	1	2	
TIMM9	3	2	1	
TIPARP	3	2	1	

TMBIM4	3	1	2	
TMEM123	3	2	1	
TMEM140	3	1	2	
TMEM147	3	1	2	
TMEM177	3	2	1	
TMEM185B	3	1	2	
TMEM194A	3	1	2	
TMEM97	3	1	2	
TNFAIP2	3	2	1	
TNFRSF12A	3	1	2	
TNFSF13	3	1	2	
TOMM20	3	0	3	
TOPORS	3	2	1	
TP53I3	3	1	2	
TPST1	3	2	1	
TRA2A	3	2	1	
TRAFD1	3	1	2	
TRIM13	3	3	0	
TRIT1	3	2	1	
TSPAN6	3	1	2	
TTC23	3	2	1	
TTC4	3	1	2	
TUBB3	3	2	1	
TUFT1	3	2	1	
U2AF1	3	1	2	
U2SURP	3	1	2	
UBR2	3	2	1	
UBR5	3	2	1	
UBR7	3	0	3	
ULK1	3	1	2	
USP47	3	3	0	
USP9X	3	2	1	
UTP14A	3	1	2	
VEZF1	3	1	2	
VRK3	3	2	1	
VTN	3	2	1	
WDFY3	3	2	1	
WDTC1	3	1	2	
WIPF2	3	1	2	
WRB	3	1	2	
WWTR1	3	2	1	
XPA	3	1	2	
ZBED1	3	1	2	
ZBED5	3	2	1	
ZBTB24	3	1	2	
ZBTB43	3	1	2	
ZFP30	3	1	2	
ZFX	3	2	1	

ZIC1	3	1	2	
ZMYM2	3	3	0	
ZMYND8	3	2	1	
ZNF14	3	1	2	
ZNF165	3	1	2	
ZNF184	3	1	2	
ZNF189	3	1	2	
ZNF200	3	1	2	
ZNF22	3	0	3	
ZNF226	3	3	0	
ZNF302	3	2	1	
ZNF419	3	2	1	
ZNF473	3	0	3	
ZNF551	3	1	2	
ZXDC	3	0	3	
AARS	2	0	2	
ABCA3	2	0	2	
ABCC6	2	2	0	
ABCF2	2	0	2	
ABHD10	2	0	2	
ABHD11	2	2	0	
ABR	2	0	2	
ACOT8	2	2	0	
ACPP	2	2	0	
ACTR3B	2	0	2	
ACVR2A	2	2	0	
ADAMTS9	2	0	2	
ADCY3	2	0	2	
ADH5	2	0	2	
ADORA2B	2	0	2	
ADRBK1	2	2	0	
AFM	2	2	0	
AGGF1	2	0	2	
AGK	2	0	2	
AHCY	2	2	0	
AHCYL2	2	2	0	
AHNAK	2	0	2	
AIMP2	2	0	2	
AK2	2	0	2	
AKAP13	2	2	0	
AKAP8L	2	2	0	
AKAP9	2	2	0	
AKR1C3	2	0	2	
AKTIP	2	0	2	
ALDH1L1	2	0	2	
ALG3	2	0	2	
ALOX15B	2	2	0	
ALPP	2	2	0	

AMACR	2	2	0	
AMD1	2	2	0	
ANAPC5	2	0	2	
ANKLE2	2	0	2	
ANKRD17	2	2	0	
ANXA1	2	0	2	
AP3M2	2	0	2	
APOL2	2	0	2	
APOOL	2	0	2	
AQP3	2	0	2	
AQR	2	0	2	
ARFGEF2	2	2	0	
ARG2	2	2	0	
ARHGAP35	2	2	0	
ARHGDIA	2	0	2	
ARHGEF1	2	2	0	
ARHGEF2	2	0	2	
ARID3A	2	2	0	
ARNT	2	2	0	
ASAH1	2	0	2	
ASAP1-IT1	2	0	2	
ASH1L	2	2	0	
ASH2L	2	0	2	
ASS1	2	2	0	
ASTN2	2	0	2	
ATAD2	2	2	0	
ATF3	2	0	2	
ATF6	2	2	0	
ATF7IP	2	2	0	
ATG12	2	2	0	
ATP1B1	2	2	0	
ATP2A2	2	0	2	
ATP2B4	2	2	0	
ATP6V0C	2	2	0	
ATP6V1D	2	2	0	
ATP9B	2	2	0	
ATR	2	0	2	
ATRX	2	2	0	
ATXN1	2	0	2	
ATXN7	2	2	0	
AURKB	2	2	0	
AVL9	2	0	2	
B4GALT3	2	2	0	
BACE2	2	0	2	
BAG3	2	0	2	
BANP	2	2	0	
BAP1	2	0	2	
BAX	2	2	0	

BAZ2A	2	2	0	
BAZ2B	2	2	0	
BBS10	2	0	2	
BCL2L1	2	0	2	
BCS1L	2	0	2	
BICD2	2	0	2	
BIK	2	2	0	
BNIP2	2	2	0	
BPTF	2	2	0	
BRCA1	2	0	2	
BRD7	2	0	2	
BUB1	2	2	0	
BZW1	2	2	0	
C10ORF95	2	0	2	
C12ORF10	2	0	2	
C12ORF41	2	0	2	
C12ORF52	2	0	2	
C15ORF29	2	0	2	
C19ORF80	2	2	0	
C1ORF115	2	2	0	
C2	2	2	0	
C20ORF11	2	2	0	
C20ORF30	2	2	0	
C2CD2	2	0	2	
C6ORF47	2	0	2	
C6ORF48	2	0	2	
C7ORF68	2	2	0	
C9ORF40	2	0	2	
CACNB3	2	0	2	
CACYBP	2	0	2	
CAD	2	0	2	
CALM1	2	2	0	
CAND1	2	0	2	
CARS	2	0	2	
CASP3	2	2	0	
CASP8	2	0	2	
CAV1	2	0	2	
CBFA2T2	2	2	0	
CBL	2	2	0	
CBLB	2	2	0	
CBLL1	2	2	0	
CBX7	2	0	2	
CBY1	2	0	2	
CC2D1A	2	2	0	
CCDC101	2	0	2	
CCDC59	2	0	2	
CCDC85C	2	0	2	
CCNF	2	0	2	

CCNJ	2	0	2	
CCNL1	2	2	0	
CCNT2	2	2	0	
CD1C	2	0	2	
CD38	2	2	0	
CD3EAP	2	0	2	
CD44	2	0	2	
CD47	2	2	0	
CD79B	2	2	0	
CD83	2	2	0	
CDC20	2	2	0	
CDC7	2	0	2	
CDH15	2	2	0	
CDH5	2	2	0	
CDK12	2	2	0	
CDK4	2	0	2	
CDK5R1	2	2	0	
CDK9	2	2	0	
CDS1	2	2	0	
CEBPG	2	0	2	
CENPA	2	2	0	
CEP152	2	2	0	
CERS2	2	2	0	
CERS4	2	0	2	
CES3	2	0	2	
CETN3	2	0	2	
CHD1	2	2	0	
CHD3	2	2	0	
CHGB	2	2	0	
CHI3L2	2	2	0	
CHIC2	2	2	0	
CHMP1A	2	0	2	
CHPT1	2	0	2	
CHRNE	2	2	0	
CHST10	2	0	2	
CIAO1	2	0	2	
CIITA	2	2	0	
CISD1	2	2	0	
CKAP5	2	0	2	
CKS2	2	2	0	
CLCN2	2	0	2	
CLDN15	2	2	0	
CLDN4	2	0	2	
CLDN9	2	2	0	
CLEC3B	2	0	2	
CLIP1	2	2	0	
CLP1	2	0	2	
CNDP2	2	2	0	

CNOT4	2	2	0	
COASY	2	0	2	
COBL	2	0	2	
COG4	2	0	2	
COMMD3	2	0	2	
COMT	2	0	2	
CORO1C	2	2	0	
COX4I1	2	0	2	
COX7A1	2	0	2	
CPA1	2	2	0	
CPNE1	2	2	0	
CPNE3	2	2	0	
CPS1	2	0	2	
CPSF4	2	0	2	
CPSF6	2	0	2	
CPT2	2	0	2	
CREB3L1	2	2	0	
CRIPT	2	0	2	
CRLF3	2	0	2	
CRP	2	2	0	
CRYZL1	2	0	2	
CSE1L	2	0	2	
CSF1	2	2	0	
CSF2RB	2	2	0	
CSK	2	0	2	
CSN3	2	2	0	
CST3	2	0	2	
CTCF	2	0	2	
CTSE	2	0	2	
CUL4A	2	0	2	
CUL4B	2	2	0	
CUL5	2	0	2	
CUTC	2	0	2	
CYB5R2	2	0	2	
CYP19A1	2	0	2	
CYP1A1	2	0	2	
CYP1B1	2	0	2	
CYP24A1	2	2	0	
CYP2B6	2	2	0	
CYP2C19	2	2	0	
CYP3A4	2	2	0	
CYP4F8	2	2	0	
DAXX	2	0	2	
DCAF11	2	2	0	
DCP2	2	0	2	
DDAH2	2	0	2	
DDB2	2	0	2	
DDR1	2	2	0	

DDRGK1	2	0	2	
DDX1	2	0	2	
DDX19A	2	0	2	
DDX23	2	0	2	
DDX3X	2	2	0	
DDX50	2	0	2	
DECR1	2	0	2	
DENND1B	2	2	0	
DENND5B	2	0	2	
DERL1	2	0	2	
DFFB	2	0	2	
DHRS7B	2	0	2	
DHRS9	2	0	2	
DHX30	2	0	2	
DIEXF	2	0	2	
DIO1	2	0	2	
DIS3	2	0	2	
DLG3	2	2	0	
DLGAP4	2	2	0	
DLGAP5	2	2	0	
DLX4	2	2	0	
DMPK	2	2	0	
DMXL2	2	2	0	
DNAJB14	2	2	0	
DNAJC24	2	0	2	
DNASE1L1	2	0	2	
DNMT1	2	0	2	
DNMT3B	2	0	2	
DNPEP	2	0	2	
DOPEY1	2	2	0	
DPH2	2	0	2	
DPY19L2P2	2	2	0	
DPYSL2	2	0	2	
DPYSL4	2	2	0	
DR1	2	2	0	
DRG1	2	0	2	
DROSHA	2	0	2	
DSG2	2	0	2	
DSN1	2	0	2	
DTWD1	2	0	2	
DUS4L	2	0	2	
DUSP1	2	2	0	
DYNC1H1	2	0	2	
DYNC2LI1	2	0	2	
DYSF	2	2	0	
E2F3	2	2	0	
EAPP	2	0	2	
ECM1	2	2	0	

EDNRA	2	2	0	
EEA1	2	2	0	
EFNA1	2	2	0	
EHBP1	2	0	2	
EHD3	2	0	2	
EHHADH	2	0	2	
EID1	2	0	2	
EIF1B	2	2	0	
EIF2AK3	2	2	0	
EIF2B1	2	0	2	
EIF2S2	2	0	2	
EIF3E	2	2	0	
EIF4A3	2	2	0	
EIF4H	2	0	2	
ELK1	2	2	0	
ELL	2	2	0	
EPAS1	2	2	0	
EPHA2	2	0	2	
EPHA4	2	2	0	
EPRS	2	0	2	
ERBB3	2	2	0	
ERC1	2	2	0	
ERCC5	2	0	2	
ESRP2	2	2	0	
ETFDH	2	0	2	
ETNK2	2	0	2	
EXOC1	2	0	2	
EXOC7	2	2	0	
EXOSC10	2	0	2	
F7	2	2	0	
FAIM	2	0	2	
FAM114A2	2	0	2	
FAM127B	2	0	2	
FAM134B	2	0	2	
FAM13B	2	2	0	
FAM164A	2	0	2	
FAM168B	2	2	0	
FAM192A	2	0	2	
FAM20B	2	0	2	
FAM55C	2	0	2	
FARSA	2	0	2	
FAS	2	0	2	
FBL	2	2	0	
FBXL14	2	0	2	
FBXL6	2	2	0	
FBXO28	2	2	0	
FBXO5	2	2	0	
FBXO9	2	0	2	

FBXW2	2	0	2	
FCHO1	2	0	2	
FECH	2	0	2	
FGF9	2	2	0	
FGFR1OP	2	2	0	
FHL2	2	2	0	
FICD	2	0	2	
FIP1L1	2	2	0	
FKBP4	2	2	0	
FKSG49	2	0	2	
FLT3	2	0	2	
FN1	2	2	0	
FNDC3A	2	0	2	
FOXN3	2	0	2	
FRMD4B	2	2	0	
FSCN1	2	2	0	
FSTL1	2	2	0	
FTSJ2	2	0	2	
FTSJ3	2	0	2	
FUCA1	2	0	2	
FUT1	2	2	0	
FUT6	2	2	0	
FXN	2	0	2	
FXYD3	2	0	2	
FXYD7	2	0	2	
FZD7	2	0	2	
GADD45B	2	2	0	
GAL	2	2	0	
GALNT14	2	0	2	
GAN	2	2	0	
GAPDH	2	0	2	
GAPVD1	2	2	0	
GATA4	2	0	2	
GATA6	2	2	0	
GBP1	2	2	0	
GCLM	2	0	2	
GCN1L1	2	0	2	
GEMIN4	2	0	2	
GFAP	2	0	2	
GFPT2	2	2	0	
GGA3	2	2	0	
GGPS1	2	0	2	
GIGYF2	2	2	0	
GK	2	0	2	
GLT8D1	2	0	2	
GLUL	2	2	0	
GMCL1	2	0	2	
GMEB1	2	2	0	

GMNN	2	2	0	
GNAQ	2	0	2	
GOLGA3	2	0	2	
GOLGA4	2	2	0	
GOLGA5	2	0	2	
GOLIM4	2	0	2	
GPATCH1	2	2	0	
GPC1	2	2	0	
GPN2	2	2	0	
GPR143	2	0	2	
GPRC5A	2	0	2	
GPX1	2	2	0	
GRAMD4	2	0	2	
GRB7	2	2	0	
GSK3B	2	2	0	
GSN	2	0	2	
GSTT1	2	2	0	
GTF2B	2	2	0	
GTF2E1	2	0	2	
GTF2IRD1	2	0	2	
GTF3A	2	0	2	
GTPBP3	2	0	2	
GTSE1	2	0	2	
H2AFZ	2	2	0	
H6PD	2	2	0	
HADHA	2	0	2	
HADHB	2	0	2	
HAT1	2	0	2	
HBP1	2	2	0	
HCP5	2	0	2	
HDAC7	2	0	2	
HDDC2	2	0	2	
HDLBP	2	0	2	
HEATR2	2	2	0	
HEATR3	2	0	2	
HEATR6	2	0	2	
HEBP2	2	2	0	
HECTD3	2	0	2	
HEG1	2	2	0	
HERC6	2	0	2	
HES2	2	2	0	
HEXA	2	2	0	
HFE	2	2	0	
HIC2	2	2	0	
HIP1	2	0	2	
HIPK1	2	2	0	
HIST1H1B	2	2	0	
HIST1H1D	2	2	0	

HIST1H1E	2	2	0	
HIST1H1T	2	2	0	
HIST1H2AI	2	2	0	
HIST1H2AM	2	2	0	
HIST1H2BK	2	2	0	
HIST1H2BN	2	2	0	
HIST1H2BO	2	2	0	
HIST1H3E	2	2	0	
HIST1H3F	2	2	0	
HIST1H3G	2	2	0	
HIST1H4D	2	2	0	
HIST1H4E	2	2	0	
HIST1H4H	2	2	0	
HIST2H2BE	2	2	0	
HK2	2	0	2	
HLA-DOB	2	0	2	
HLA-DQB1	2	2	0	
HMGB2	2	2	0	
HMGCS1	2	2	0	
HMGN4	2	0	2	
HMOX1	2	2	0	
HNRNPA0	2	2	0	
HOXB13	2	2	0	
HPS5	2	0	2	
HSD17B7	2	2	0	
HSDL2	2	0	2	
HSF2	2	2	0	
HUS1	2	2	0	
IDE	2	0	2	
IDH2	2	2	0	
IDS	2	2	0	
IER3IP1	2	0	2	
IER5	2	2	0	
IFI30	2	0	2	
IFI44L	2	2	0	
IFITM3	2	0	2	
IFNAR2	2	0	2	
IFRD1	2	0	2	
IFT46	2	0	2	
IGF1R	2	2	0	
IGFBP2	2	2	0	
IKBKB	2	0	2	
IL12B	2	2	0	
IL13RA1	2	2	0	
IL13RA2	2	2	0	
IL1RN	2	2	0	
IL32	2	2	0	
IL36G	2	2	0	

IMPACT	2	0	2	
INADL	2	2	0	
ING3	2	2	0	
INHBB	2	0	2	
INO80B	2	0	2	
INPP5F	2	0	2	
INSIG1	2	2	0	
INSR	2	2	0	
IP6K1	2	2	0	
IP6K2	2	2	0	
IPO13	2	2	0	
IPO5	2	0	2	
IPP	2	0	2	
IQCE	2	0	2	
IRF5	2	0	2	
IRF7	2	2	0	
IRS1	2	0	2	
ISOC2	2	0	2	
ITGA10	2	0	2	
ITGA5	2	0	2	
ITM2B	2	2	0	
JUP	2	0	2	
KARS	2	0	2	
KAT6A	2	2	0	
KAT8	2	0	2	
KATNB1	2	0	2	
KBTBD2	2	2	0	
KCNF1	2	0	2	
KCNG1	2	0	2	
KDELR2	2	0	2	
KDM4C	2	2	0	
KDM5B	2	2	0	
KDM6A	2	2	0	
KDM6B	2	2	0	
KIAA0100	2	0	2	
KIAA0247	2	2	0	
KIAA0895	2	2	0	
KIAA1279	2	0	2	
KIDINS220	2	2	0	
KLHDC3	2	0	2	
KLHL21	2	0	2	
KLHL24	2	2	0	
KLK13	2	2	0	
KLK5	2	2	0	
KNG1	2	0	2	
KRT81	2	2	0	
LACTB2	2	0	2	
LAP3	2	2	0	

LBR	2	0	2	
LCMT1	2	0	2	
LEF1	2	2	0	
LGR4	2	0	2	
LGR5	2	2	0	
LIFR	2	2	0	
LMF1	2	2	0	
LMO3	2	2	0	
LOC100127972	2	0	2	
LOC100129361	2	0	2	
LOC283683	2	0	2	
LOC399491	2	2	0	
LOC81691	2	0	2	
LPIN2	2	2	0	
LRIG1	2	0	2	
LRP5L	2	2	0	
LRRC31	2	0	2	
LSM3	2	0	2	
LUZP1	2	0	2	
LZTFL1	2	0	2	
MACF1	2	0	2	
MAD2L1	2	2	0	
MAGEA1	2	0	2	
MAK16	2	0	2	
MAP1LC3B	2	2	0	
MAP2K4	2	0	2	
MAP3K14	2	2	0	
MAP4	2	0	2	
MAP4K2	2	2	0	
MAP4K4	2	0	2	
MAP6D1	2	0	2	
MAP7D1	2	2	0	
MAP7D3	2	0	2	
MAST3	2	0	2	
MAT2A	2	0	2	
MBL2	2	2	0	
MCM4	2	2	0	
MCM6	2	2	0	
MCM7	2	2	0	
MDM1	2	0	2	
MED6	2	2	0	
MED7	2	0	2	
MEGF9	2	0	2	
METTL13	2	0	2	
METTL2B	2	0	2	
METTL3	2	0	2	
METTL4	2	0	2	
MFAP3	2	2	0	

MFSD11	2	0	2	
MGAT4A	2	2	0	
MICALL1	2	0	2	
MICB	2	2	0	
MIOS	2	2	0	
MIS12	2	2	0	
MIS18A	2	0	2	
MKI67	2	2	0	
MMACHC	2	0	2	
MMADHC	2	2	0	
MORC2	2	0	2	
MPDU1	2	2	0	
MPHOSPH8	2	0	2	
MPP2	2	2	0	
MPP5	2	2	0	
MPPE1	2	0	2	
MPRIP	2	0	2	
MPZL2	2	2	0	
MREG	2	0	2	
MRPL19	2	0	2	
MRPL2	2	0	2	
MRPL34	2	0	2	
MRPL39	2	0	2	
MRPL52	2	0	2	
MRPS18B	2	0	2	
MRPS28	2	2	0	
MSL3	2	0	2	
MST1	2	0	2	
MTF2	2	2	0	
MTMR2	2	0	2	
MUC5B	2	2	0	
MUTYH	2	0	2	
MVP	2	2	0	
MYBL2	2	2	0	
MYLK3	2	0	2	
N4BP2L1	2	2	0	
NAAA	2	0	2	
NAALAD2	2	2	0	
NAB1	2	2	0	
NAT1	2	0	2	
NBN	2	0	2	
NCAPD2	2	2	0	
NCLN	2	2	0	
NCOA3	2	2	0	
NDUFAF1	2	0	2	
NDUFAF4	2	0	2	
NDUFB11	2	2	0	
NDUFB2	2	2	0	

NDUFS6	2	2	0	
NEK4	2	0	2	
NENF	2	0	2	
NFKB1	2	0	2	
NFKB2	2	2	0	
NFRKB	2	2	0	
NFYC	2	2	0	
NIPA2	2	0	2	
NIPBL	2	2	0	
NKX3-1	2	2	0	
NLE1	2	0	2	
NOL8	2	0	2	
NPPB	2	2	0	
NR4A1	2	2	0	
NSUN5	2	0	2	
NTAN1	2	0	2	
NTRK2	2	0	2	
NUCB2	2	2	0	
NUDCD3	2	0	2	
NUDT13	2	0	2	
NUDT2	2	0	2	
NUP62	2	0	2	
NUPL2	2	0	2	
NUSAP1	2	2	0	
ODF1	2	0	2	
OLFM1	2	2	0	
OPHN1	2	0	2	
OR2B6	2	2	0	
ORC2	2	2	0	
OSBP	2	2	0	
OVOL1	2	2	0	
OVOL2	2	2	0	
P2RY2	2	0	2	
PA2G4	2	0	2	
PALB2	2	0	2	
PALLD	2	2	0	
PARD6B	2	2	0	
PARG	2	0	2	
PBXIP1	2	2	0	
PCM1	2	0	2	
PCNX	2	2	0	
PCYT1A	2	2	0	
PCYT2	2	0	2	
PDCD5	2	0	2	
PDE8A	2	0	2	
PDGFD	2	0	2	
PDLIM2	2	2	0	
PDP1	2	2	0	

PEA15	2	2	0	
PELP1	2	0	2	
PES1	2	0	2	
PEX10	2	2	0	
PEX14	2	0	2	
PFAS	2	0	2	
PFKM	2	0	2	
PGRMC2	2	0	2	
PHGDH	2	0	2	
PIBF1	2	2	0	
PIK3IP1	2	2	0	
PIK3R1	2	0	2	
PIN1	2	0	2	
PIP4K2A	2	0	2	
PIP5K1A	2	2	0	
PJA2	2	2	0	
PKN2	2	2	0	
PLAGL2	2	2	0	
PLAU	2	2	0	
PLCB3	2	0	2	
PLD3	2	2	0	
PLEKHA1	2	0	2	
PLEKHM1	2	2	0	
PLLP	2	2	0	
PLOD2	2	2	0	
PLOD3	2	0	2	
PLXNA1	2	0	2	
PMM2	2	2	0	
PMS2P1	2	0	2	
PNKP	2	0	2	
PNMA1	2	0	2	
PNMT	2	2	0	
POLA2	2	0	2	
POLB	2	0	2	
POLD3	2	0	2	
POLM	2	2	0	
POLR2I	2	0	2	
POP5	2	0	2	
POPDC3	2	0	2	
PPAN	2	0	2	
PPAP2A	2	2	0	
PPIE	2	0	2	
PPM1E	2	2	0	
PPP1CB	2	2	0	
PPP1R12B	2	2	0	
PPP1R7	2	0	2	
PPP5C	2	2	0	
PRCP	2	2	0	

PRDM2	2	2	0	
PRDX4	2	2	0	
PRKAA1	2	2	0	
PRKD2	2	2	0	
PRMT3	2	0	2	
PRMT5	2	0	2	
PRPF4	2	0	2	
PRPS1	2	2	0	
PRRC1	2	0	2	
PRSS21	2	2	0	
PRSS22	2	2	0	
PRSS23	2	0	2	
PRSS8	2	2	0	
PSG9	2	2	0	
PSMA6	2	2	0	
PSMD2	2	0	2	
PSMD8	2	2	0	
PSMD9	2	0	2	
PSMG1	2	0	2	
PSMG2	2	0	2	
PSPH	2	0	2	
PTCH1	2	2	0	
PTGER3	2	2	0	
PTGIS	2	2	0	
PTP4A2	2	0	2	
PTPN21	2	2	0	
PTPRK	2	2	0	
PTS	2	0	2	
PUM1	2	2	0	
PUS1	2	0	2	
PXDN	2	2	0	
QKI	2	2	0	
QRICH1	2	2	0	
R3HCC1	2	0	2	
RAB23	2	2	0	
RAB27B	2	0	2	
RAB7A	2	2	0	
RABEP2	2	0	2	
RABIF	2	0	2	
RAI14	2	2	0	
RALGAPA1	2	2	0	
RALGAPB	2	0	2	
RAN	2	0	2	
RARA	2	2	0	
RARS	2	0	2	
RASAL2	2	2	0	
RBBP5	2	2	0	
RBFA	2	0	2	

RBFOX2	2	2	0	
RBM10	2	0	2	
RBM28	2	0	2	
RELA	2	0	2	
REPS2	2	0	2	
RFC4	2	0	2	
RFX5	2	0	2	
RGS17	2	2	0	
RHOB	2	0	2	
RIC8B	2	0	2	
RIPK2	2	0	2	
RMND5B	2	0	2	
RNF138	2	2	0	
RNF14	2	0	2	
RNF24	2	2	0	
RNF4	2	0	2	
RNF41	2	0	2	
RNF44	2	0	2	
RNGTT	2	2	0	
RPH3A	2	0	2	
RPH3AL	2	0	2	
RPL13	2	0	2	
RPL17	2	0	2	
RPL18	2	0	2	
RPL27	2	0	2	
RPL35	2	0	2	
RPL36A	2	0	2	
RPL37A	2	0	2	
RPL6	2	0	2	
RPS19	2	0	2	
RPS28	2	2	0	
RPS6KB1	2	2	0	
RPS6KC1	2	0	2	
RPUSD2	2	0	2	
RRN3	2	2	0	
RRN3P1	2	2	0	
RRP15	2	0	2	
RRP1B	2	0	2	
RRP9	2	0	2	
RSF1	2	2	0	
RUFY2	2	0	2	
RUNDC3B	2	0	2	
RUNX1	2	2	0	
RUVBL1	2	0	2	
RWDD2B	2	0	2	
SAP30L	2	0	2	
SAT1	2	2	0	
SCAF4	2	0	2	

SCARA3	2	0	2	
SCG5	2	0	2	
SCGB1D2	2	2	0	
SCGB2A1	2	2	0	
SCO2	2	0	2	
SDAD1	2	0	2	
SDCBP	2	2	0	
SDCCAG3	2	0	2	
SEC14L1	2	2	0	
SEC22A	2	0	2	
SEC62	2	2	0	
SEC63	2	0	2	
SECISBP2	2	0	2	
SEH1L	2	0	2	
SEMA4C	2	2	0	
SEMA5A	2	0	2	
SERINC5	2	0	2	
SERPINA1	2	2	0	
SERPINB6	2	0	2	
SERPINI1	2	0	2	
SETD6	2	0	2	
SF1	2	2	0	
SF3A3	2	0	2	
SFPQ	2	2	0	
SFTPC	2	2	0	
SGSH	2	2	0	
SH2D1A	2	2	0	
SH3BP1	2	0	2	
SH3YL1	2	0	2	
SIRT7	2	2	0	
SKIL	2	2	0	
SKIV2L	2	0	2	
SKIV2L2	2	0	2	
SLC12A7	2	2	0	
SLC14A1	2	0	2	
SLC15A2	2	2	0	
SLC1A2	2	0	2	
SLC25A1	2	2	0	
SLC25A15	2	0	2	
SLC25A4	2	2	0	
SLC25A46	2	0	2	
SLC27A2	2	0	2	
SLC2A4	2	2	0	
SLC31A2	2	2	0	
SLC35F5	2	2	0	
SLC39A6	2	0	2	
SLC39A8	2	2	0	
SLC39A9	2	2	0	

SLC44A1	2	0	2	
SLC45A2	2	2	0	
SLC48A1	2	0	2	
SLC4A8	2	0	2	
SLC7A11	2	0	2	
SLC9A6	2	0	2	
SLCO1B3	2	2	0	
SMARCA5	2	2	0	
SMARCC2	2	0	2	
SMARCD2	2	0	2	
SMARCD3	2	2	0	
SMARCE1	2	0	2	
SMC3	2	0	2	
SMC5	2	2	0	
SMG7	2	2	0	
SNAPC1	2	2	0	
SNN	2	0	2	
SNRK	2	2	0	
SNUPN	2	0	2	
SNX16	2	2	0	
SNX24	2	0	2	
SNX3	2	0	2	
SNX4	2	0	2	
SNX6	2	0	2	
SORBS1	2	2	0	
SORBS3	2	0	2	
SOX9	2	2	0	
SP1	2	2	0	
SP140L	2	2	0	
SP2	2	2	0	
SP3	2	2	0	
SPAG1	2	2	0	
SPATA20	2	2	0	
SPI1	2	2	0	
SPP1	2	0	2	
SPTSSA	2	2	0	
SRGAP3	2	2	0	
SRRM2	2	2	0	
SRSF6	2	0	2	
SRSF8	2	0	2	
SS18L2	2	2	0	
SSR1	2	0	2	
SSR3	2	0	2	
SSSCA1	2	0	2	
ST3GAL4	2	2	0	
ST6GAL1	2	2	0	
STAMBP	2	0	2	
STAT6	2	0	2	

STC2	2	0	2	
STK17B	2	2	0	
STK25	2	0	2	
STK3	2	2	0	
STK38L	2	0	2	
STRN	2	2	0	
STXBP2	2	0	2	
SUCLA2	2	2	0	
SUGP2	2	2	0	
SUMO2	2	2	0	
SUMO3	2	0	2	
SUN1	2	2	0	
SURF1	2	0	2	
SUV39H2	2	0	2	
SYNCRIP	2	0	2	
SYNJ2BP	2	2	0	
TAF12	2	0	2	
TARP	2	2	0	
TBC1D8	2	2	0	
TBC1D9B	2	2	0	
TBCCD1	2	0	2	
TBL3	2	0	2	
TCEA2	2	0	2	
TCEB3	2	0	2	
TCERG1	2	0	2	
TDG	2	2	0	
TDRD7	2	0	2	
TERF1	2	0	2	
TERF2IP	2	2	0	
TEX261	2	0	2	
TFAP2A	2	2	0	
TFB1M	2	0	2	
TFCP2L1	2	0	2	
TFDP1	2	0	2	
TFPI	2	0	2	
TFRC	2	0	2	
TGIF1	2	0	2	
TGM2	2	2	0	
TGOLN2	2	2	0	
THAP4	2	0	2	
THBS1	2	2	0	
THOC1	2	0	2	
THY1	2	2	0	
TIA1	2	0	2	
TIMM17B	2	0	2	
TIPIN	2	0	2	
TJP2	2	0	2	
TJP3	2	0	2	

TLE3	2	0	2	
TLE4	2	2	0	
TLR2	2	0	2	
TM9SF2	2	0	2	
TMCC2	2	0	2	
TMED1	2	2	0	
TMEM135	2	2	0	
TMEM30A	2	2	0	
TMEM30B	2	2	0	
TMF1	2	2	0	
TMOD3	2	0	2	
TMX2	2	0	2	
TNF	2	2	0	
TNIK	2	0	2	
TNMD	2	2	0	
TNPO1	2	0	2	
TNPO3	2	0	2	
TNS3	2	0	2	
TOB2	2	2	0	
TOMM70A	2	0	2	
TOP1	2	2	0	
TOP3A	2	2	0	
TOR1AIP1	2	2	0	
TPD52L2	2	0	2	
TPK1	2	0	2	
TPM1	2	0	2	
TPR	2	2	0	
TPRKB	2	0	2	
TPSB2	2	0	2	
TRA2B	2	0	2	
TRAF5	2	0	2	
TRAPPC2L	2	2	0	
TREM2	2	2	0	
TRIM16	2	0	2	
TRIM24	2	2	0	
TRIM26	2	0	2	
TRIM27	2	0	2	
TRIM44	2	0	2	
TRIM5	2	2	0	
TRMT1L	2	0	2	
TRMT2B	2	0	2	
TRMU	2	0	2	
TRPC1	2	2	0	
TSC1	2	2	0	
TSC22D2	2	2	0	
TSC22D3	2	2	0	
TSEN2	2	0	2	
TSEN34	2	2	0	

TSKU	2	2	0	
TSN	2	0	2	
TSPAN14	2	2	0	
TSPAN3	2	0	2	
TSPAN5	2	0	2	
TSPO	2	0	2	
TTBK2	2	2	0	
TTC27	2	0	2	
TTC38	2	2	0	
TUBB	2	0	2	
TUBGCP3	2	0	2	
TXN2	2	2	0	
TYMS	2	0	2	
UBAC1	2	0	2	
UBE2H	2	2	0	
UBE2J1	2	0	2	
UBE2L6	2	2	0	
UBE2M	2	0	2	
UBL4A	2	0	2	
UBR4	2	2	0	
UGT2A3	2	0	2	
UGT2B28	2	0	2	
UNC119	2	0	2	
UPF3A	2	0	2	
UQCC	2	0	2	
URB2	2	0	2	
USP14	2	0	2	
UST	2	0	2	
UTP18	2	0	2	
UTP6	2	0	2	
VAMP2	2	2	0	
VAMP3	2	0	2	
VAX2	2	2	0	
VCL	2	2	0	
VCP	2	2	0	
VCPIP1	2	2	0	
VDAC1	2	0	2	
VEGFC	2	2	0	
VOPP1	2	0	2	
VPS13B	2	2	0	
VPS54	2	0	2	
WAC	2	2	0	
WAS	2	2	0	
WASF3	2	0	2	
WDR11	2	0	2	
WDR12	2	0	2	
WDR3	2	0	2	
WDR41	2	0	2	

WDR44	2	0	2	
WDR59	2	2	0	
WDR61	2	0	2	
WDR62	2	0	2	
WDR7	2	2	0	
WDR74	2	0	2	
WDR77	2	0	2	
WEE1	2	2	0	
WIF1	2	2	0	
WISP2	2	2	0	
WIZ	2	2	0	
WSB2	2	0	2	
WWP1	2	0	2	
XIAP	2	2	0	
XPC	2	0	2	
XPO4	2	2	0	
XRCC1	2	0	2	
YIPF2	2	0	2	
YTHDF1	2	0	2	
YY1AP1	2	2	0	
ZBTB1	2	2	0	
ZBTB17	2	2	0	
ZBTB39	2	0	2	
ZBTB7A	2	2	0	
ZC3H14	2	0	2	
ZDHHC17	2	2	0	
ZDHHC4	2	0	2	
ZEB2	2	0	2	
ZFC3H1	2	0	2	
ZG16	2	0	2	
ZKSCAN4	2	0	2	
ZNF10	2	2	0	
ZNF175	2	0	2	
ZNF202	2	0	2	
ZNF222	2	2	0	
ZNF268	2	2	0	
ZNF324	2	2	0	
ZNF329	2	2	0	
ZNF335	2	2	0	
ZNF394	2	2	0	
ZNF442	2	2	0	
ZNF467	2	2	0	
ZNF500	2	0	2	
ZNF518A	2	2	0	
ZNF544	2	0	2	
ZNF571	2	2	0	
ZNF609	2	2	0	
ZNF668	2	0	2	

ZNF710	2	0	2	
ZNF750	2	2	0	
ZNF787	2	0	2	
ZNF83	2	0	2	
ZNF85	2	0	2	
ZNHIT2	2	0	2	
ZSCAN12	2	2	0	
